# Benchtop ^19^F Nuclear Magnetic Resonance (NMR) Spectroscopy Provides Mechanistic Insight into the Biginelli Condensation toward the Chemical Synthesis of Novel Trifluorinated Dihydro- and Tetrahydropyrimidinones as Antiproliferative Agents

**DOI:** 10.1021/acsomega.3c00290

**Published:** 2023-03-10

**Authors:** Rosie Chen, Pratyush Singh, Sarah Su, Selin Kocalar, Xina Wang, Neha Mandava, Srishti Venkatesan, Adrienne Ferguson, Aishi Rao, Emma Le, Casey Rojas, Edward Njoo

**Affiliations:** † Department of Chemistry, Biochemistry and Physics, Aspiring Scholars Directed Research Program, Fremont, California 94539, United States

## Abstract

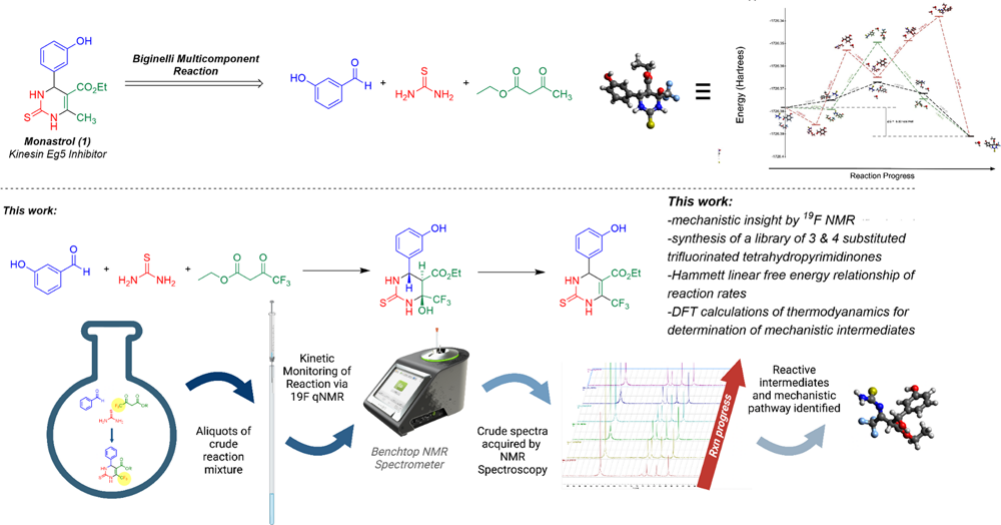

Benchtop nuclear magnetic resonance (NMR) spectroscopy has enabled the monitoring and optimization of chemical transformations while simultaneously providing kinetic, mechanistic, and structural insight into reaction pathways with quantitative precision. Moreover, benchtop NMR proton lock capabilities further allow for rapid and convenient monitoring of various organic reactions in real time, as the use of deuterated solvents is not required. The complementary role of ^19^F NMR-based kinetic monitoring in the fluorination of bioactive compounds has many benefits in the drug discovery process since fluorinated motifs additionally improve drug pharmacology. In this study, ^19^F NMR spectroscopy was utilized to monitor the synthesis of novel trifluorinated analogs of monastrol, a small molecule dihydropyrimidinone kinesin-Eg5 inhibitor, and to probe the mechanism of the Biginelli cyclocondensation, a multicomponent reaction used to synthesize dihydropyrimidinone and tetrahydropyrimidinones through a Bronsted- or Lewis-acid catalyzed cyclocondensation between ethyl acetoacetate, thiourea, and an aryl aldehyde. In the present study, a trifluorinated ketoester serves a dual purpose as being the source of the trifluoromethyl group in our fluorinated dihydropyrimidinones and as a spectroscopic handle for real-time reaction monitoring and tracking of reactive intermediates by ^19^F NMR. Further, upon extending this workflow to a diverse array of 3- and 4-substituted aryl aldehydes, we were able to derive Hammett linear free energy relationships (LFER) to determine stereoelectronic effects of *para-* and *meta-*substituted aryl aldehydes to corresponding reaction rates and mechanistic routes. In addition, we used density functional theory (DFT) calculations to corroborate our experimental results through the thermodynamic values of key intermediates in each mechanism. Finally, these studies culminate in the synthesis of a novel trifluorinated analog of monastrol and its subsequent biological evaluation *in vitro.* More broadly, we show an application of benchtop ^19^F NMR spectroscopy as an analytical tool in the real-time investigation of a mechanistically and chemically complex multicomponent reaction mixture.

## Introduction

Multicomponent reactions (MCRs) have enabled the rapid chemical synthesis of complex and diverse molecular scaffolds, and this has, in turn, accelerated the discovery of small molecules with therapeutic potential.^1,2^ The Biginelli cyclocondensation is a three-component acid-catalyzed MCR between an aldehyde, β-ketoester, and urea, which has been used to produce dihydropyrimidinone and tetrahydropyrimidinone heterocycles.^3−5^ One such dihydropyrimidinone, monastrol (**1**), was found to be a potent inhibitor of kinesin Eg5, a motor protein necessary for the assembly of mitotic spindle fibers, thereby inducing apoptosis by preventing the development of spindle bipolarity and arresting cells in mitosis.^6−8^ This discovery has inspired the synthesis of other antiproliferative dihydropyrimidinone scaffolds, including LaSOM-63 (**2**), which, unlike monastrol, induces apoptosis of glioma cells through the inhibition of ecto-5′nucleotidase/CD73 activity.^9^ The broad biological applicability of these scaffolds accessed through the Biginelli cyclocondensation highlights the utility of this MCR as a synthetic platform for the discovery of bioactive small molecules.

The Biginelli cyclocondensation proceeds through three possible mechanistic pathways: the imine route, involving initial Schiff base condensation between the aldehyde and urea; the enamine route, involving initial condensation between the urea and β-ketoester; and the Knoevenagel route, involving initial aldol condensation between the aldehyde and β-ketoester.^4^ The mechanistic investigation of the Biginelli condensation has been complicated by short-lived reactive intermediates, which are difficult, if not impossible, to isolate, as well as the possibility for multiple competing mechanistic pathways. Previously, studies by Ramos and co-workers using electrospray mass spectrometry have suggested that the imine pathway is preferred when the reaction is performed with conventional β-ketoesters.^10^

We decided to synthesize the trifluorinated counterparts of monastrol and LaSOM-63 because of the bioorthogonal nature of fluorine and the increased metabolic stability offered by the presence of carbon–fluorine bonds.^11−19^ Of note, while it has been reported that the substitution of a β-ketoester with a 4,4,4-trifluorinated ketoester can be used to prepare trifluorinated versions of these dihydropyrimidinones and tetrahydropyrimidinones through the Biginelli reaction, the operative mechanism through which the Biginelli proceeds with 4,4,4-trifluoroketoesters has not been investigated. Recent advances in benchtop nuclear magnetic resonance (NMR) spectroscopy have allowed for noncanonical applications of NMR spectroscopy as an analytical tool directly in a synthetic laboratory setting, including real-time reaction monitoring.^20−23^

Here, we utilize benchtop ^19^F NMR spectroscopy to probe the mechanism of the Biginelli cyclocondensation of trifluorinated ketoesters by tracking the production of various aryl-substituted trifluorinated tetrahydropyrimidinones and their reactive intermediates in real time. In this study, a library of various 3- and 4-substituted aromatic aldehydes not only gave access to a diverse library of 6-aryl trifluorinated tetrahydroprymidinones but also provided mechanistic insight into the Biginelli through *meta-* and *para-*Hammett linear free energy relationships (LFER) derived from ^19^F NMR spectra of crude reaction mixtures.^24,25^ While quantitative measurement of chemical species present in a reaction mixture might be possible with other means, such as high-performance liquid chromatography (HPLC), LC-MS, or GC-MS; the quantification of such species by ^19^F NMR does not require any reaction workup or further sample processing and can be done within seconds or minutes of set time points, in a manner that is not invasive to the reaction mixture and therefore allows for direct measurement of species of interest in an unadulterated setting. Moreover, the ability to track a reaction by NMR is readily adapted for flow monitoring conditions.^26,27^

Additionally, we employed computational modeling by density functional theory (DFT) calculations to further probe the thermodynamics of the Biginelli reaction involving trifluorinated ketoesters.^28,29^ Finally, the antiproliferative efficacy of a selection of our compounds was explored in cancer cells. On a broader scope, we describe a workflow that may be applied to gain mechanistic understanding or to optimize the synthesis of other fluorinated compounds with potential medicinal significance.

## Results and Discussion

**Scheme 1:**
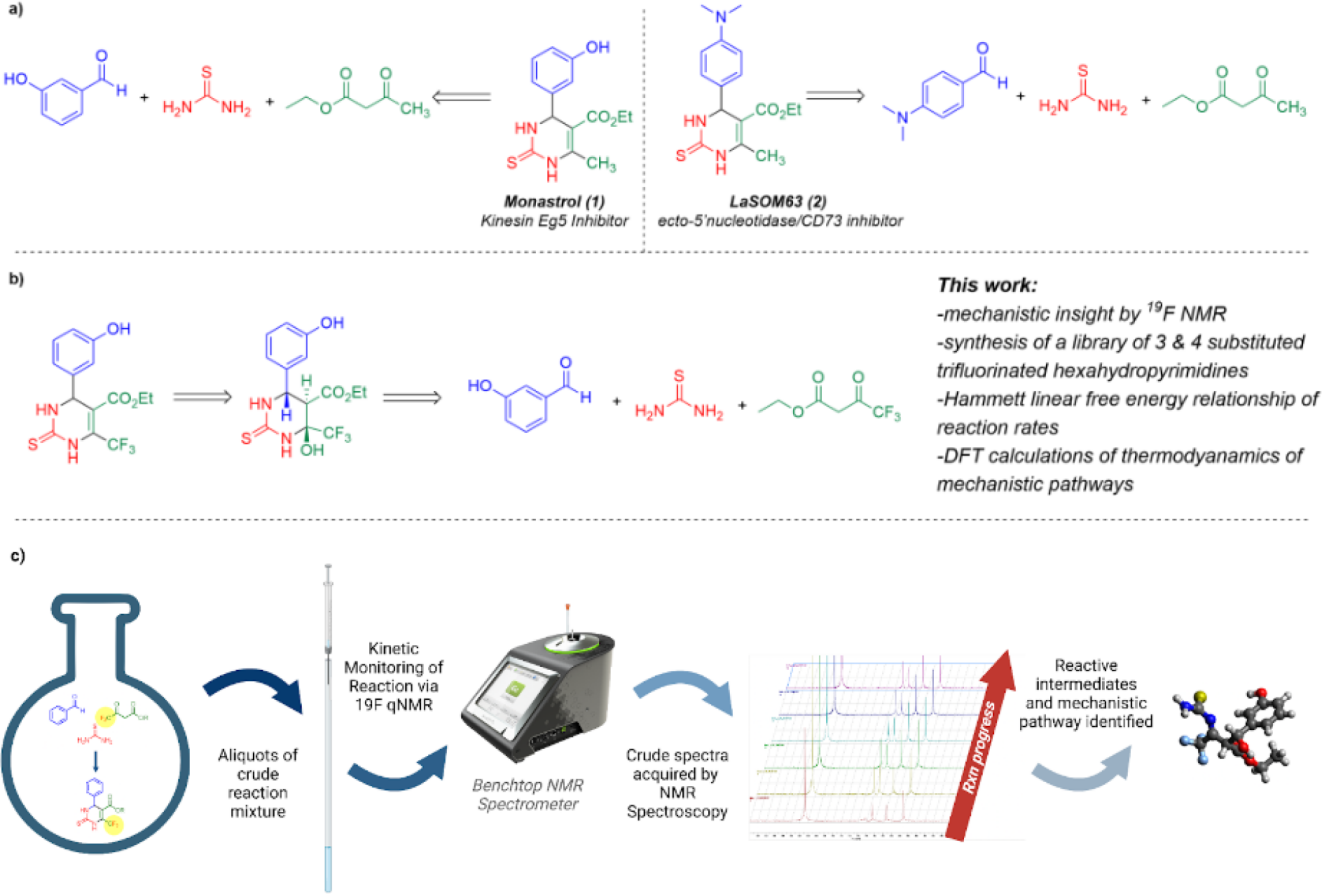
(a) Monastrol, a Reported Kinesin Eg5 Inhibitor, and an Analogous Dihydropyrimidinone, LaSOM-63, a Known Ecto-5′ Nucleotidase/CD73 Activity Inhibitor: Two Examples of Biologically Active 2,4-Dihydropyrimidinones That Can Be Prepared through the Biginelli Cyclocondensation; (b) Preparation of a Trifluorinated Analog of Monastrol through an Analogous Biginelli Cyclocondensation Involving a 4,4,4-Trifluoroketoester and the Mechanism of the Analogous Biginelli on Trifluoroketoesters by Benchtop NMR; (c) Workflow for ^19^F NMR Based Mechanistic Studies of the Biginelli Cyclocondensation, Crude Reaction Mixtures Directly Analyzed by ^19^F NMR (Benchtop NMR Stock Photo Adapted with Permission. Copyright 2022, Nanalysis Corp.)

Our interest in trifluorinated tetrahydropyrimidinones and dihydropyrimidinones was initially motivated out of a desire to prepare trifluorinated analogs of monastrol and other dihydropyrimidinones and tetrahydropyrimidinones (Scheme 1a). Given the modularity of the Biginelli reaction, we employed an ethyl 4,4,4-trifluoroacetoacetate as the origin of the trifluoromethyl group (Scheme 1b). From the onset, we found that the trifluorinated ketoester was a convenient spectroscopic handle on benchtop ^19^F nuclear magnetic resonance (NMR) spectroscopy for real-time, quantitative analysis of the rates of formation and absolute concentrations of reaction intermediates and product formation as a function of time. Since these instruments do not require deuterated solvents with proton lock capabilities, crude reaction mixtures or reaction aliquots could be directly analyzed in the instrument without further processing, and on an analytical scale, reactions could themselves be performed in standard 5 mm NMR tubes for rapid, high throughput reaction condition screening and optimization (Scheme 1c), and indeed we previously reported the use of ^19^F NMR spectroscopy for reaction condition optimization for a scalable synthesis of other fluorinated small molecules.^30^ To make this workflow quantitative, reaction mixtures were spiked with an internal standard, and for the purposes of this study, α,α,α-trifluorotoluene was selected over hexafluorobenzene as an internal standard. An initial screen of a variety of Lewis acid catalysts revealed that 8 mol % ytterbium(III) triflate in acetonitrile most efficiently catalyzed the Biginelli condensation between ethyl 4,4,4,-trifluoroacetoacetate, thiourea, and 3-hydroxybenzaldehyde to the corresponding tetrahydropyrimidinone (Table 1).^31−33^

**Table 1:** Catalyst Optimization Screening Results^a^

entry	reaction conditions	results
1	LaCl_3_, 20 mol %, MeCN, 0.33 M^c^^,^^b^	56%
2	Yb(OTf)_3_, 20 mol %, MeCN, 0.33 M^c^	49%
3	Sc(OTf)_3_, 20 mol %, MeCN, 0.33 M	negligible conversion
4	In(OTf)_3_, 20 mol %, MeCN, 0.33 M	negligible conversion
5	Yb(OTf)_3_, 2 mol %, MeCN, 0.16 M	3%
6	Yb(OTf)_3_, 4 mol %, MeCN, 0.16 M	7%
7	Yb(OTf)_3_, 6 mol %, MeCN, 0.16 M	9%
8	Yb(OTf)_3_, 8 mol %, MeCN, 0.16 M	16%
9	Yb(OTf)_3_, 10 mol %, MeCN, 0.16 M	13%
10	Yb(OTf)_3_, 12 mol %, MeCN, 0.16 M	12%
11	Yb(OTf)_3_, 14 mol %, MeCN, 0.16 M	12%
12	Yb(OTf)_3_, 8 mol %, MeCN, 0.1 M^c^	31%
13	Yb(OTf)_3_, 8 mol %, MeCN, 0.5 M^c^	41%
14	Yb(OTf)_3_, 8 mol %, MeCN, 0.75 M^c^	63%
15	Yb(OTf)_3_, 8 mol %, MeCN, 1 M^c^	73%
16	Yb(OTf)_3_, 8 mol %, EtOH, 0.16 M	16%
17	LaCl_3_, 2 mol %, EtOH, 0.16 M	15%
18	LaCl_3_, 2 mol %, DMF, 0.16 M	no conversion
19	LaCl_3_, 2 mol %, EtOH, 1 M^c^	44%
20	Yb(OTf)_3_, 8 mol %, EtOH, 1 M^c^	45%

a A catalytic system of ytterbium(III) triflate in MeCN produced the highest and most reproducible yields. While lanthanum(III) chloride had a higher yield in some cases, its poor solubility in MeCN and other organic solvents made it a less desirable choice. 8 mol % ytterbium(III) triflate was found to be the optimal catalyst loading concentration. The reaction yield increased as the reaction concentration increased, with the 1 M reaction having the highest yield of the tests. Screening of the reactions in different solvents showed either poor yield or poor solubility. Percent conversion was determined by ^19^F qNMR of the crude reaction mixture after 3 h against a trifluorotoluene (C_6_H_5_CF_3_) internal standard unless indicated otherwise. All reactions were performed at 60 °C.

b While lanthanum chloride gave comparably high yields, its limited solubility in a variety of solvent screens complicated efforts to have reproducible high throughput reaction screening.

c Isolated yield.

**Scheme 2:**
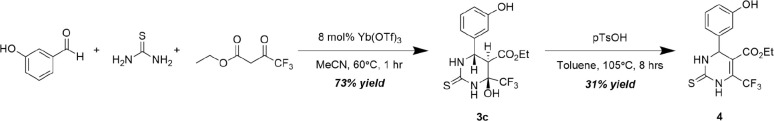
Synthesis of the Trifluorinated Tetrahydropyrimidinones through the Biginelli Cyclocondensation, a One-Pot Multicomponent Reaction That Reacts Ethyl Acetoacetate, an Aryl Aldehyde, and Thiourea to Form a Dihydropyrimidinone (Tetrahydropyrimidinone When Using Trifluoroethylacetoacetate) Subsequent dehydration of tetrahydropyrimidinone analog of trifluoromonastrol proceeds with excess p-toluenesulfonic acid in reflux with 36% yield.

As expected and in concordance with previous literature reports, the Biginelli cyclocondensation involving trifluorinated keto esters failed to undergo final dehydration into the dihydropyrimidinone, instead terminating at the tetrahydropyrimidinone, and this was ubiquitously observed across a select series of tested Lewis acid catalysts. From here, we followed a procedure utilizing excess p-toluenesulfonic acid (pTsOH, Scheme 2) in toluene established by Agbaje et al.^34^ to dehydrate the tetrahydropyrimidinone (compound **4**). Notably, a dehydrated trifluoromonastrol compound has not been previously reported in the literature.^35−37^ Other dehydration conditions screened (Table 2) did not give the desired product.

**Table 2:** Conditions Screened for the Dehydration of the Tetrahydropyrimidinone (**3C**) to Its Respective Dihydropyrimidinone (**4**), Utilizing Lewis Acids in Order to Catalyze the Dehydration of Our Hydrated Product Produced by Running the Biginelli Cyclocondensation with a Trifluorinated Ketoester

entry	reaction conditions	results
1	Yb(OTf)_3_, MeCN, reflux	no dehydration
2	H_2_SO_4_, PhMe	no dehydration, then decomposition
3	P_2_O_5_, 1,4-dioxane	no dehydration
4	P_2_O_5_, DMF	decomposition
5	3 equiv pTsOH, PhMe, reflux	31% yield

**Scheme 3:**
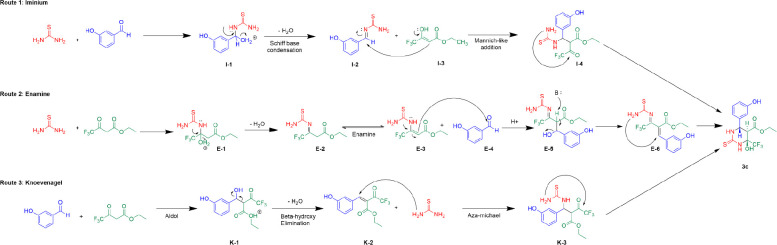
Three Possible Mechanisms of the Biginelli Multicomponent Cyclocondensation Reaction (1) The imine/iminium mechanism, wherein thiourea condenses with the benzaldehyde carbonyl, resulting in a Schiff base which undergoes subsequent addition with the ketoester. This undergoes a final 6-exo-trig cyclization to form compound **3**. (2) The enamine mechanism, where thiourea condenses with the ketone of the ketoester, forming intermediate imine **E-2**, which tautomerizes to the enamine in intermediate **E-3**. **E-3** undergoes nucleophilic addition to the benzaldehyde and undergoes a subsequent dehydration and final cyclization to form compound **3**. (3) The Knoevenagel mechanism, where the aryl aldehyde and ethylacetoacetate react first in an aldol condensation to form ketoenoate **K-2**, which reacts with the thiourea in an aza-Michael addition; this resultant intermediate **K-3** undergoes a final 6-exo-trig cyclization to form compound **3**.

**Figure 1:**
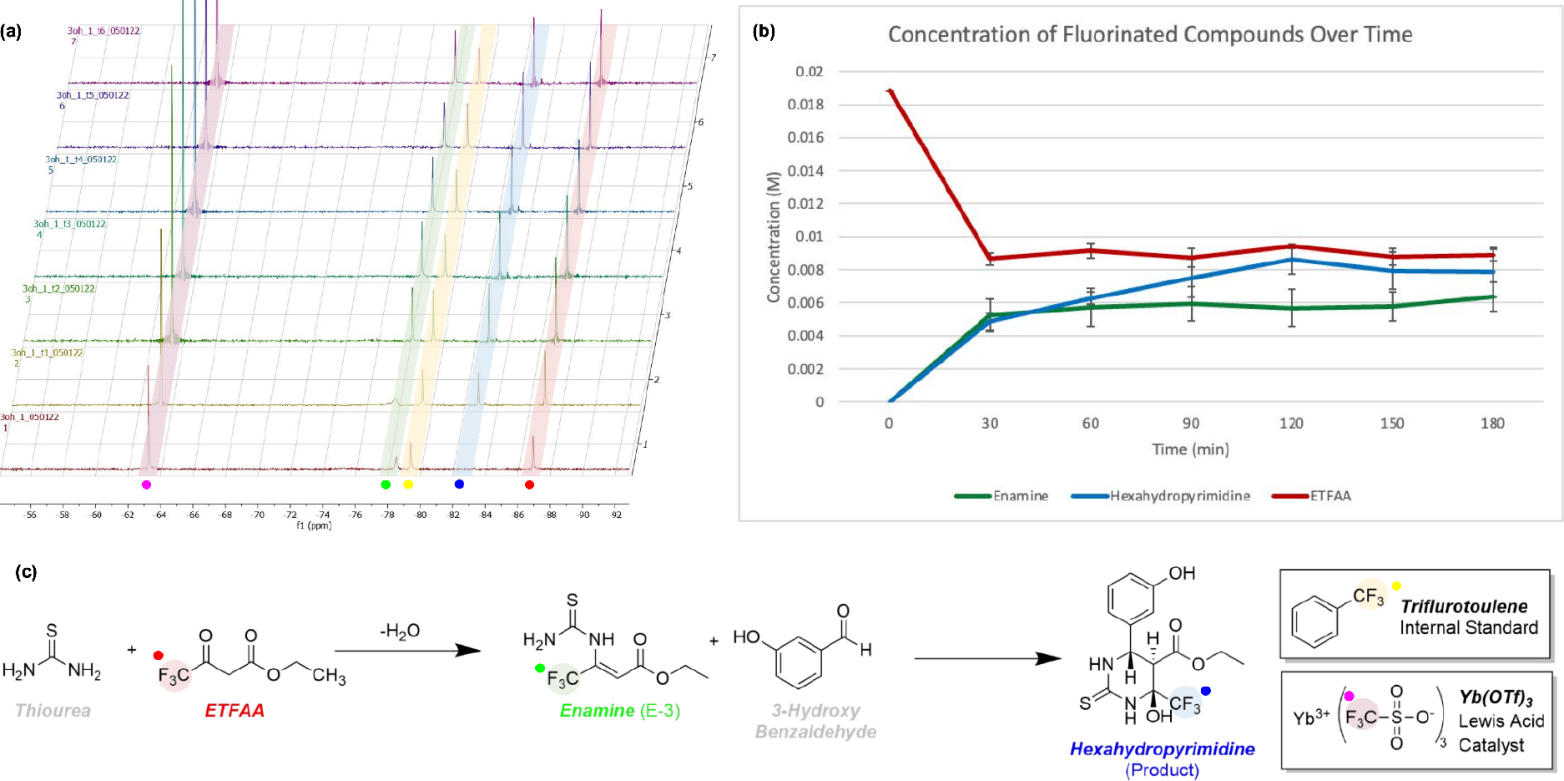
Benchtop NMR enables quantitative reaction monitoring of the Biginelli reaction. (a) Timecourse stacked ^19^F NMR spectra taken every 30 min between ethyl 4,4,4,-trifluoroacetoacetate (ETFAA; ^19^F resonance shown in red), thiourea, and 3-hydroxybenzaldehyde. (b) Monitoring the progress of this reaction is possible by qNMR integration of distinct ^19^F resonances: consumption of starting material (ETFAA, labeled in red), mechanistic intermediates (enamine intermediate, labeled in green), and product formation over time (tetrahydropyrimidinone, labeled in blue). (c) Color-coded assignments of ^19^F resonances in the Biginelli cyclocondensation between ETFAA, 3-hydroxybenzaldehyde, and thiourea en route to the preparation of a trifluorinated monastrol analog.

The synthesis of the tetrahydropyrimidinones can proceed through three possible mechanisms depending on which two reagents react first: the iminium mechanism, the enamine mechanism, and the Knoevenagel mechanism (Scheme 3). In prior studies, the synthesis of monastrol has been observed to occur through the iminium mechanism, wherein thiourea condenses with the benzaldehyde carbonyl, resulting in a Schiff base, which undergoes subsequent addition with the ketoester, which then undergoes a final 6-exo-trig cyclization to form the final tetrahydropyrimidinone. Using ^19^F NMR, we were able to observe both the tetrahydropyrimidinone product (δ = −82.73 ppm) and an unexpected reactive intermediate (δ = −78.48 ppm) on the timecourse NMR (Figure 1a). The presence of a fluorinated intermediate shows that the reaction’s primary operative mechanism was either the enamine or Knoevenagel mechanism, as the iminium intermediate has no fluorine handle. We then graphed the quantity of the tetrahydropyrimidinone product and this reaction intermediate on a concentration over time graph (Figure 1b) and assigned the appropriate chemical structures to them (Figure 1c).

**Figure 2:**
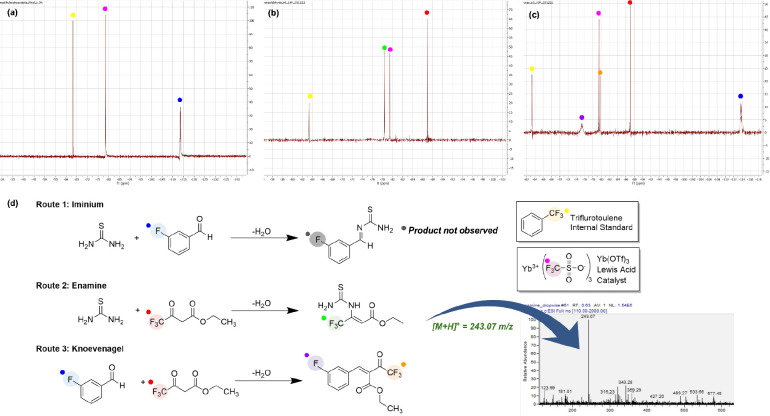
^19^F NMR spectra of the intermediates of fluorinated Biginelli reaction intermediates. (a) NMR spectra of reaction between thiourea and 3-fluorobenzaldehyde, operating through the iminium pathway. (b) NMR spectra of the reaction between thiourea and ethyl 4,4,4-trifluoroacetoacetate, forming intermediate **E-3** with a fluorine resonance at −78.50 ppm, which proceeds through the enamine pathway. (c) NMR spectra of the reaction between 3-fluorobenzaldehyde and ethyl 4,4,4-trifluoroacetoacetate, forming intermediate **K-2** with two fluorine resonances at −80.22 ppm, which proceeds through the Knoevenagel pathway. (d) Pathways pictured were operative in various kinetics experiments to reveal the production of their intermediates over time with ^19^F NMR.

In order to identify the observed singlet fluorine resonance (δ = −78.48 ppm), we performed selective experiments where the conversion of each of the intermediates in the possible mechanisms was forced and characterized. Moreover, we used 3-fluorobenzaldehyde (δ = −113.78 ppm [Ar-**F**] in CH_3_CN, q, *J* = 7.5 Hz) as one of the starting materials to determine whether or not the new intermediate would incorporate the aryl fluoride as the aryl fluoride resonance would remain unchanged if it was the enamine intermediate and would change if it were the Knoevenagel. Since the aryl fluoride was not incorporated, the identity of the peak was preliminarily established as the enamine intermediate. Its identity was further verified by ESI-MS (expected *m*/*z* = 243.04; found *m*/*z* = 243.07 [M + H]; Figure 2d) of the crude reaction mixture; no significant mass ion corresponding to tetrahydropyrimidinone **E-1** was observed. Efforts to force Schiff base condensation of thiourea and a fluorinated benzaldehyde (Figure 2a) did not result in any observable new peaks on crude ^19^F NMR. A corresponding experiment where thiourea was excluded from the reactants to drive exclusive formation of the Knoevenagel condensation product (δ = −79.77 ppm [-C**F**_**3**_] in CH_3_CN, δ = −75.35 ppm [Ar-**F**] in CH_3_CN) demonstrated that the earlier observed peak was not indeed the Knoevenagel adduct (Figure 2c). Additionally, in the absence of the ytterbium catalyst, the Knoevenagel condensation product **K-2** was the only observed intermediate, but this ultimately did not lead to any discernible production of the cyclized tetrahydropyrimidinone product even under extended reaction times. The necessity of the ytterbium(III) triflate catalyst in driving the operative mechanistic pathway might be rationalized by coordination of the metal cation with the 1,3-dicarbonyl of the fluorinated ketoester, thereby activating the α,α,α-trifluoroketone to nucleophilic attack by thiourea.

**Scheme 4:**
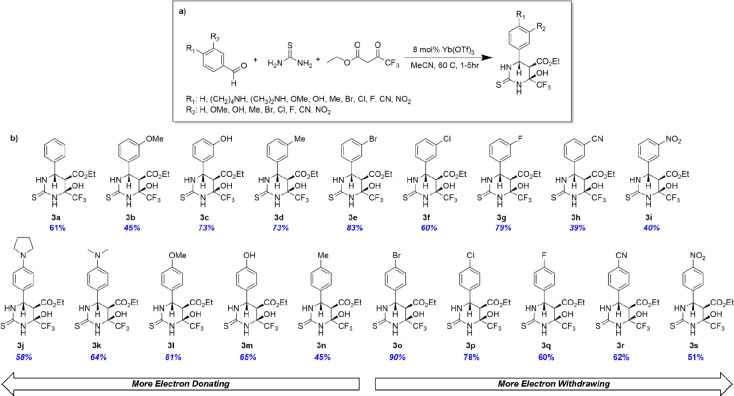
Synthesis of Trifluorinated Tetrahydropyrimidinone Analogs (a) The general synthetic procedure for synthesizing tetrahydropyrimidinone analogs. Analogs were synthesized with 8 mol % Yb(OTf)_3_ at 60°C in acetonitrile. (b) The synthesized library of meta- and para-aryl substituted tetrahydropyrimidinones with both electron-donating (left) and electron-withdrawing (right) aryl substitutions. Isolated yields are shown below each compound, for which absolute purity was established to be >95% by quantitative NMR (qNMR).

After completing the synthesis of trifluoromonastrol, we then pursued the preparation of an analogous trifluorinated dihydropyrimidinone compound with a 4-*N*,*N*-dimethylamino aryl fragment in efforts to prepare a trifluorinated analog of LaSOM-63. During this synthetic process, we observed a drastically different rate of formation of intermediates and products in comparison to the synthesis of trifluoromonastrol, as observed by time course ^19^F NMR of the crude reaction mixture. Intrigued by the difference an aryl substituent had on the reaction, we synthesized a library of trifluorinated tetrahydropyrimidinone with differing *meta-* and *para-*aryl substituents, including pyrrolidine, dimethyl, methoxy, hydroxy, methyl, bromo, chloro, fluoro, cyano, and nitro groups to investigate the role of aryl stereoelectronics on the reaction pathway (Scheme 4).

**Figure 3:**
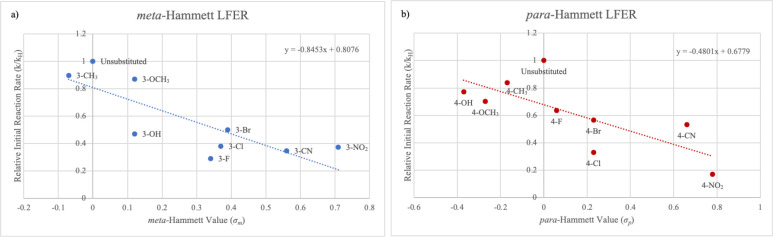
Hammett free energy relationship (LFER) plots of participation of 3- and 4-substituted aryl aldehydes in the Biginelli cyclocondensation involving ethyl 4,4,4-trifluoroacetoacetate and thiourea. (a) The LFER plots for the meta-substituted tetrahydropyrimidinones. A negative line of best fit between the meta-Hammett values (σ_m_) and the reaction rate indicates a buildup of positive charge/loss of negative charge at the benzylic center in the transition state of the rate-determining step. (b) The LFER plots for the para-substituted tetrahydropyrimidinones. A negative line of best fit between the para-Hammett values (σ_p_) and the reaction rate also indicates a buildup of positive charge/loss of negative charge at the benzylic center in the transition state of the rate-determining step. Rates of relative product formation were normalized with respect to the observed rate of formation with the unsubstituted benzaldehyde. All kinetics experiments were performed in triplicate with two operators to ensure reproducibility.

Reaction rates determined by aliquot ^19^F qNMR were then used to derive Hammett linear free energy plots (Figure 3). Upon analysis of the effect of the aryl aldehyde Hammett values on the initial rate of product formation as a function as determined by quantitative ^19^F NMR of timecourse reaction aliquots, we found a consistent negative Hammett linear free energy relationship in both 3- and 4-substituted aryl aldehyde series. This suggests that the rate-determining step results in an increase in positive charge in the transition state at the benzylic reaction center position, which we postulated might be the E1cb β-hydroxy elimination from **E-5** to **E-6**, which would proceed through partial carbocation character at the benzylic carbon.

**Figure 4:**
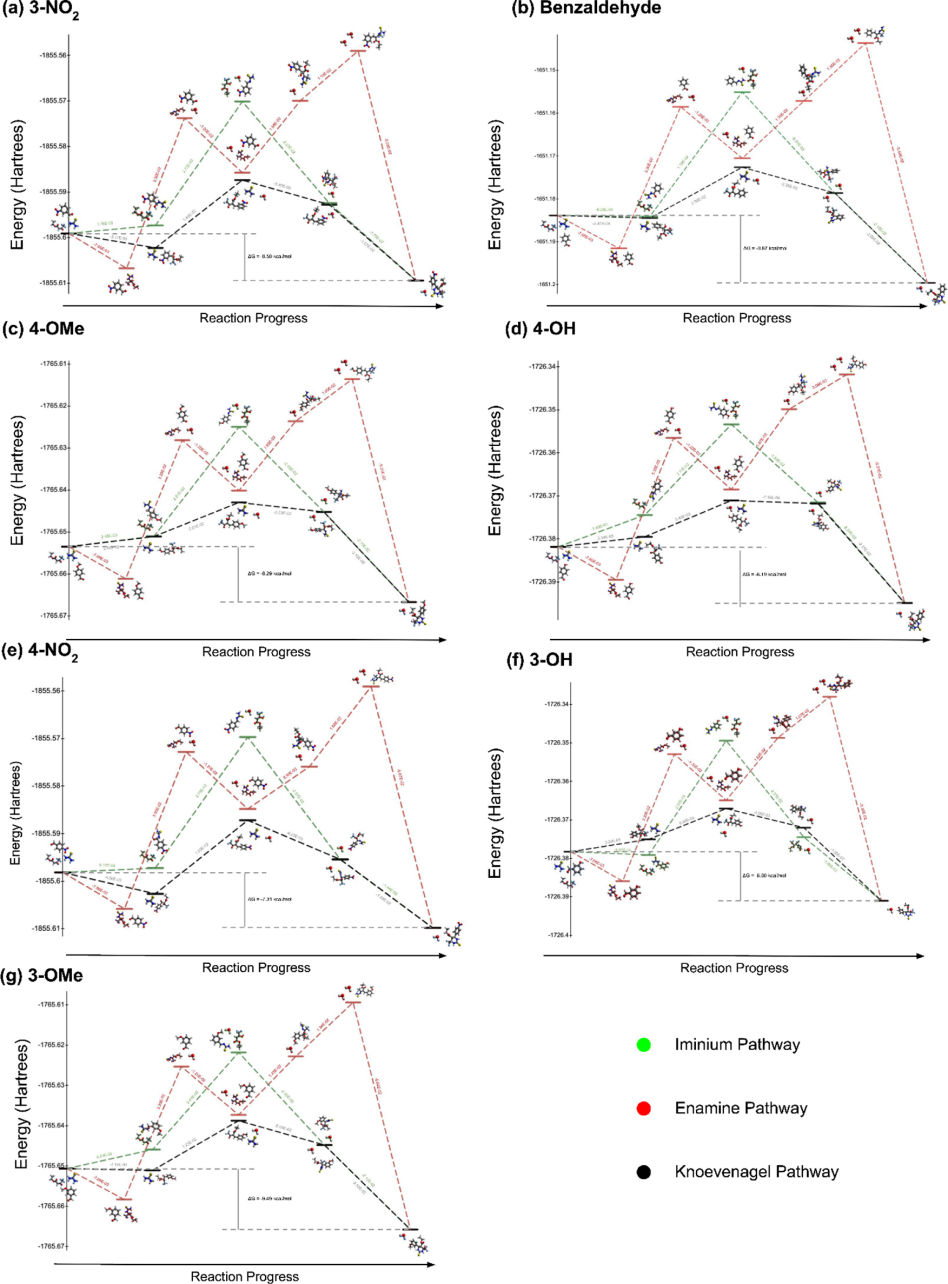
Density functional theory (DFT) calculations of the thermodynamic profiles of enamine, iminium, and Knoevenagel pathways of select 3- and 4-aryl substituents. For all aryl substituents modeled, the initiation step in the enamine mechanism was most thermodynamically favorable (−4.8 kcal/mol for 3-OH substitution). Moreover, the steep thermodynamic disfavorability of the penultimate dehydration is consistent with our Hammett linear free energy relationships, as the model corroborates the theory that the final dehydration is the rate-determining step of the reaction mechanism. DFT free energy values were calculated as a Boltzmann weighted average of conformers at the B3LYP/6-31G(d,p) levels of theory. (a) Calculated thermodynamics of mechanistic pathways en route to analog **3i** (3-NO_2_ substituent). (b) Calculated thermodynamics of mechanistic pathways en route to analog **3a** (Benzaldehyde substituent). (c) Calculated thermodynamics of mechanistic pathways en route to analog **3l** (4-OMe substituent). (d) Calculated thermodynamics of mechanistic pathways en route to analog **3m** (4-OH substituent). (e) Calculated thermodynamics of mechanistic pathways en route to analog **3s** (4-NO_2_ substituent). (f) Calculated thermodynamics of mechanistic pathways en route to analog **3c** (3-OH substituent). (g) Calculated thermodynamics of mechanistic pathways en route to analog **3b** (3-OMe substituent).

We further probed the thermodynamics of the three possible mechanistic pathways by performing density functional theory (DFT) free energy calculations on various reactive intermediates en route to the tetrahydropyrimidinone in each of the mechanistic possibilities (Figure 4). We observed that, regardless of the identity of the aryl aldehyde modeled, the initial thiourea addition to the α,α,α-trifluoroketone to form tetrahydropyrimidinone **E-1** was the most thermodynamically favorable (Δ*G* = −1.4 kcal/mol) pathway, which is consistent with our mechanistic hypothesis that the trifluorinated keto ester outcompetes the aryl aldehyde as the best electrophile, thereby committing the Biginelli cyclocondensation to the enamine route. This result, as shown by computer modeling, is consistent with our observation of the initial accumulation of the intermediate identified as the enamine (δ = −78.50 ppm in CH_3_CN), regardless of the identity of the aryl aldehyde. Moreover, we observed that the hydroxyl elimination prior to the final cyclization was highly thermodynamically unfavorable; this is consistent with the observed Hammett LFER, which pointed to this elimination as the rate-determining step. The rate of the final cyclization is dependent on the concentration of the dehydrated iminoester **E-6**, whose rate is, in turn, dependent on the equilibrium of the preceding β-hydroxy elimination step.

Moreover, we used density functional theory to probe the relative thermodynamics of the final dehydration step between monastrol and trifluoromonastrol. The Δ*G* of dehydration of the tetrahydropyrimidinone to synthesize monastrol was only 2.716 kcal/mol, while the change in energy for the dehydration of the tetrahydropyrimidinone to form trifluoromonastrol was 14.640 kcal/mol, accounting for the differences observed in synthesizing the dehydrated product.

Of the four possible diastereomers of the final tetrahydropyrimidinone, the relative stereochemistry was determined by both *J*-coupling constants of the two methine protons in the ^1^H NMR as well as by density functional theory calculations. The lowest energy diastereomer (compound **3c**), with a DFT-calculated dihedral angle of 172.8° between the two methine protons, had a predicted *J* value of 11.58 Hz, and this was consistent with our observed NMR spectra in both DMSO-*d*_6_ and methanol-*d*_4_ (see Supporting Information Figure S4). Moreover, this stereochemical assignment was consistent with previous literature reports.^34^

We measured the antiproliferative activity of monastrol, trifluoromonastrol, and its hydrated tetrahydropyrimidinone against HCT-116 cells and found that monastrol had an EC_50_ value of 0.0834 mM, and trifluoromonastrol had an EC_50_ value of 0.291 mM. Trifluoromonastrol had trivially lower antiproliferative activity in comparison to monastrol, suggesting that the addition of the trifluoromethyl group results in only a modest loss in biological potency. Future studies on trifluoromonastrol’s antiproliferative activity in other cell lines, and exploration of its ADMET properties, are currently underway.

## Conclusions

In summary, en route to the preparation of a novel trifluorinated analog of monastrol, an antiproliferative dihydropyrimidinone small molecule, we determined that the Biginelli multicomponent reaction proceeds through an enamine mechanism preferentially over the imine mechanism when a 4,4,4-trifluoro-β-ketoester is used in lieu of conventional, nonfluorinated β-ketoesters. The mechanistic investigation of this reaction was enabled by benchtop ^19^F NMR spectroscopy, with which the rates of formation and reaction of fluorinated intermediates and products could be quantified. With this workflow, we show that ytterbium(III) triflate effectively catalyzes the Biginelli cyclocondensation of 4,4,4-trifluorinated β-ketoesters, aryl aldehydes, and thiourea into tetrahydropyrimidinones and that this proceeds through initial condensation of thiourea and the trifluorinated ketoester. This stands in contrast to earlier reports of the Biginelli cyclocondensation preferring the imine mechanism pathway, and this might be rationalized by the inductive activation by the neighboring fluorine atoms on the ketoester such that this outcompetes the aryl aldehyde as a preferred electrophile for initial condensation with thiourea. Also enabled by this workflow, the relative rates of this reaction observed by timecourse ^19^F qNMR show a distinct negative Hammett linear free energy correlation, corresponding to a decrease in positive charge at the benzylic carbon in the transition state of the rate-determining step. Both of these observations were consistent with DFT calculations of reaction pathways.

Finally, this workflow has led to the rapid synthesis of a library of 19 tetrahydropyrimidinones and a novel trifluorinated analog of monastrol, which was subsequently evaluated for antiproliferative activity against nonfluorinated monastrol. On a broader level, we demonstrate the versatility of benchtop heteronuclear NMR spectroscopy as a quantitative analytical tool for the mechanistic investigation of complex reaction mixtures, and we envision this workflow might be applied to the investigation of other mechanistically complex chemical systems or to flow chemistry for real-time reaction monitoring on scale, ultimately providing access to rapid reaction optimization for medicinally relevant small molecule scaffolds. Such studies are currently underway in our laboratory.

## Materials and Methods

### General Biginelli Synthesis Procedure (on Scale)

To a reaction vial charged with a Teflon stir bar under a positive pressure nitrogen atmosphere was added ethyl 4,4,4-trifluoroacetoacetate (AK Scientific, 1.32 mmol, 193 μL), Yb(OTf)_3_ (AK Scientific, 0.2 mmol, 124 mg), thiourea (Himedia, 1 mmol, 76.1 mg), and the substituted benzaldehyde stock solution (1 mmol, N/A). The reaction was stirred at 60 °C under nitrogen and monitored by ^19^F NMR (1–5 h). Upon completion, a majority of the acetonitrile was removed under reduced pressure, and the resulting crude reaction mixture was extracted in ethyl acetate (2 × 25 mL) over brine. The organic layers were combined and dried over anhydrous magnesium sulfate.

### Purification Protocol A

The filtered product was directly concentrated *in vacuo* and recrystallized in cold acetonitrile to yield a white crystalline solid.

### Purification Protocol B

The 4-pyrrolidine and 4-dimethylamino substituted compounds did not recrystallize in acetonitrile, and so the filtered product was purified via column chromatography with a hexanes and ethyl acetate eluent (100% hexanes to 3:2 EtOAc/hexanes) to afford pure products **3j** and **3k**.

### General Biginelli Synthesis Procedure (Micro Scale)

To an NMR tube was sequentially added a solution of Yb(OTf)_3_ in acetonitrile (AK Scientific, 2M, 0.1 mL), a solution of ethyl 4,4,4-trifluoroacetoacetate in acetonitrile (AK Scientific, 26.4 M, 0.05 mL), a solution of a substituted benzaldehyde in acetonitrile (20 M, 0.05 mL), a solution of thiourea in acetonitrile (Himedia, 2M, 0.5 mL), and a solution of trifluorotoluene in acetonitrile (AK Scientific, 4M, 0.05 mL). The NMR tube headspace was flushed with nitrogen, and an initial ^19^F NMR using 16 scans and a 1.0 s scan delay was taken. The reaction mixture was placed into a 60 °C water bath, and ^19^F NMR spectra were taken in 30-minute intervals over a 3 hour period.

### Kinetics Parameters

Kinetics were run in a water bath heated to a temperature of 60 °C on a heat plate in duplicate. NMR spectra were taken with 200 ppm spectral width and a 1.0 s scan delay.

### Dehydration Procedure

To a 50 mL round-bottom flask charged with a Teflon stir bar was added ethyl(4*S*,5*R*)-4-hydroxy-6-(3-hydroxyphenyl)-2-thioxo-4-(trifluoromethyl) tetrahydropyrimidinone-5-carboxylate (2 mmol, 728.68 mg), pTsOH (AK Scientific, 6 mmol, 1.141g), and toluene. The reaction was stirred at 110 °C for 8 h and tracked by thin-layer chromatography (1:1 EtOAc/hexanes). Upon completion, a majority of the toluene was removed under reduced pressure, and the resulting crude reaction mixture was purified via column chromatography with a hexanes and ethyl acetate eluent (100% hexanes to 3:7 EtOAc/hexanes) to afford pure product.

## Supporting Information Available

Characterization and spectroscopic data for all compounds, supplementary figures, computer modeling information, and additional experimental results (PDF)
