# Supplementary material for: Benchtop 19F Nuclear Magnetic Resonance (NMR) Spectroscopy Provides Mechanistic Insight into the Biginelli Condensation toward the Chemical Synthesis of Novel Trifluorinated Dihydro- and Tetrahydropyrimidinones as Antiproliferative Agents
Source: ACS Omega. 2023 Mar 10;8(11):10545–54. doi: 10.1021/acsomega.3c00290 (PMC10034998; doi:10.1021/acsomega.3c00290)
Supplement: Supplementary file 1 — ao3c00290_si_001.pdf [file ao3c00290_si_001.pdf]

Supporting Information for
Benchtop ^{19}F nuclear magnetic resonance (NMR) spectroscopy provides mechanistic insight into the Biginelli condensation towards the chemical synthesis of novel trifluorinated dihydro- and tetrahydropyrimidinones as antiproliferative agents

Authors: Rosie Chen*, Pratyush Singh*, Sarah Su, Selin Kocalar, Xina Wang, Neha Mandava, Srishti Venkatesan, Adrienne Ferguson, Aishi Rao, Emma Le, Casey Rojas, Edward Njoo

Department of Chemistry, Biochemistry & Physics,
Aspiring Scholars Directed Research Program, Fremont, CA 94539

edward.njoo@asdrp.org

A. General Information S1
 a. Materials S1
 b. Equipment S1
 c. Computational Methods S2

B. Experimental Procedures and Supplementary Figures S3
 a. MTT Procedure S3
 b. Supplementary Figures S3

C. Spectroscopic Data S8
 a. Characterization S8
  i. 3a S8
  ii. 3b S12
  iii. 3c S16
  iv. 3d S20
  v. 3e S24
  vi. 3f S28
  vii. 3g S32
  viii. 3h S36

ix.	3i	S40
x.	3j	S44
xi.	3k	S48
xii.	3l	S52
xiii.	3m	S56
xiv.	3n	S60
xv.	3o	S64
xvi.	3p	S68
xvii.	3q	S72
xviii.	3r	S76
xix.	3s	S80
xx.	4	S84

D. *In silico* modeling S87

E. References S394

1. General Information

Materials:

Solvents used in all reactions and purification processes were ACS grade or higher and were used without additional purification, and were purchased from Fisher Chemical, Sigma Aldrich, Sierra Chemical Corp, Beantown Chemical, Stellar Chemical, JT Baker, or Acros Organics. Deuterated solvents were purchased from Cambridge Isotope Laboratories, Acros Organics, or Martek Isotopes, and were used without further purification. Solvents used in analytical methods (HPLC, LCMS) were HPLC grade (22 micron filtered). Substituted benzaldehydes were purchased from the following; 3-dimethylaminobenzaldehyde, 3-methoxybenzaldehyde, 3-hydroxybenzaldehyde, 3-methylbenzaldehyde, 3-bromobenzaldehyde, 3-chlorobenzaldehyde, 3-fluorobenzaldehyde, 3-cyanobenzaldehyde, 3-nitrobenzaldehyde, 4-pyrrolidinobenzaldehyde, 4-hydroxybenzaldehyde, 4-methylbenzaldehyde, 4-bromobenzaldehyde, 4-chlorobenzaldehyde, 4-cyanobenzaldehyde, 4-methoxybenzaldehyde and 4-nitrobenzaldehyde were all obtained from AK scientific (95% purity), whereas 4-dimethylaminobenzaldehyde was purchased from HiMedia Laboratories, LLC, 3-hydroxybenzaldehyde and 4-fluorobenzaldehyde was obtained from Beantown chemical, and Benzaldehyde (99%) was obtained from Sigma Aldrich. Ytterbium trifluoromethanesulfonate (98% purity) was purchased from AK Scientific or Tokyo Chemical Industries Chemicals. p-Toluenesulfonic acid hydrate (98% purity) was obtained from Sigma Aldrich. All reagents, catalysts, and chemicals were purchased from commercial sources and used without further purification unless otherwise stated.

Equipment:

^{1}H, ^{19}F and ^{13}C{^{1}H} NMR spectra were acquired on a Nanalysis NMReady 60Pro multinuclear benchtop nuclear magnetic resonance spectrometer and were processed on the MestreNova software package. ^{1}H and ^{13}C chemical shifts are reported in parts per million (ppm). ^{1}H chemical shifts are reported relative to the residual solvent peak ($CDCl_3$ = 7.26 ppm, CD_3CN = 1.93, DMSO-*d6* = 2.49, CD_3 = 3.49) as follows: chemical shift (δ), multiplicity (app = apparent, b = broad, s = singlet, d = doublet, t = triplet, q = quartet, m = multiplet, or combinations thereof), coupling constant(s) in Hz, integration. ^{13}C chemical shifts are reported relative to the residual solvent peak (DMSO-*d6* = 39.52).

Mass spectra were obtained using a Thermo Electron LTQ-XL linear ion trap mass spectrometer equipped with a Thermo Finnigan Surveyor reverse phase high performance liquid chromatography (LC-MS) or a Thermo Scientific DSQ single quadrupole mass spectrometer equipped with a Thermo Trace 1300 gas chromatograph (GC-MS). Infrared spectra were collected on a Thermo Scientific Nicolet iS5 Fourier transform infrared (FT-IR) spectrometer equipped with a Thermo iD5 attenuated total reflectance (ATR) assembly. UV-visible spectra were acquired on a Spectronic Genesys 5 UV-vis spectrophotometer, a BioRad SmartSpec 3000 UV-visible spectrophotometer, or a Perkin Elmer Lambda 11 UV-visible spectrophotometer.

Computational Methods:

Lowest energy conformers were first generated by molecular mechanics with a Monte Carlo conformer search algorithm with the MMFF94 force field. This was performed on the OpenBabel cheminformatics software package[1].

Further quantum mechanically rigorous structural optimization was conducted using density functional theory (DFT) on ORCA, an *ab initio* quantum mechanical molecular modeling suite, with the B3LYP functional and 6-31G(d,p) basis set using a CPCM implicit solvation model[2]. DFT calculations were performed on a Dell PowerEdge 710 server cluster with a 4 x 24 core Intel Xeon X5660 processor at 2.80GHz and 128 GB RAM.

Computer models were visualized on Avogadro, an open source molecular modeling and visualization software[3].

2. Experimental Procedures

MTT Procedure:

HCT-116 cells (European Collection of Authenticated Cell Cultures) were grown to confluence at 37 ºC with 5% CO_2. Cells were washed with 1 mL DPBS (Corning), detached with 1 mL trypsin-EDTA (Tribioscience), and were washed with 6 mL of McCoy's 5A cell media (Tribioscience). Cells were centrifuged into a pellet, and resuspended in media to 250,000 cells per mL. 100 uL of the cell mixture was aliquoted per well into a 96 well plate (Endosafe), and was treated with 1 uL of the respective compound dissolved in DMSO (Fisher Scientific, Molecular Biology grade). A negative control was established by not treating cells with any compound, and a positive control was established by treating cells with 1% DMSO. The plate was incubated for 24 hours, and a fresh mixture of thiazol blue tetrazolium bromide (AK Scientific) in DPBS (5 mg/mL) was prepared. 10 µL of the MTT solution was added to each well, and the cells were allowed to incubate for 1-4 hours, before 100 µL of DMSO was added to each well and a spectrometric reading was obtained with a Labsystems Multiskan spectrometer at 570 nm. EC_{50} values were calculated following protocols by Kemmer et al[4].

Supplementary Figures:

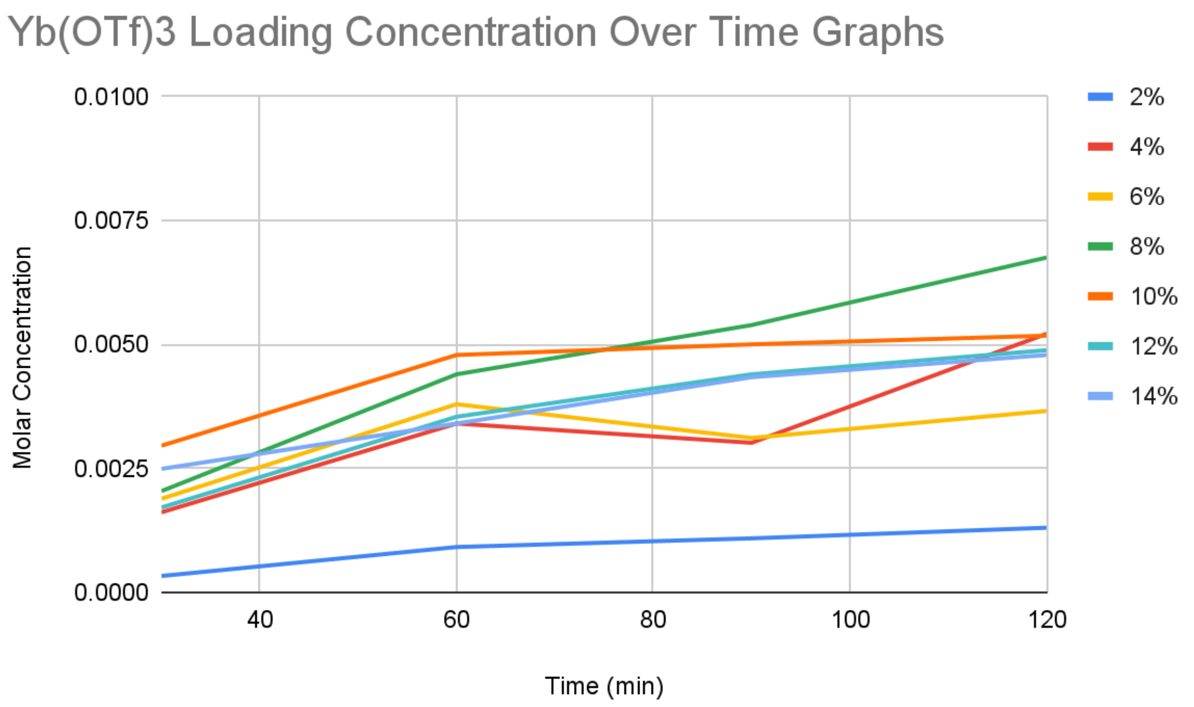

SI-1: Optimization of Catalyst Loading *Yb(OTf)$_3$ catalyst loadings of 2%-14% were tested and a 8 mol% catalyst loading was determined to be optimal. Note: The Yb(OTf)$_3$ was determined to not be a suitable internal standard due its fluctuating integration. This fluctuation of integration values was determined*

through a T1 NMR experiment to not be related to relaxation times. Trifluoro toluene was chosen as an internal standard for its stable integration and its stability to ytterbium triflate.

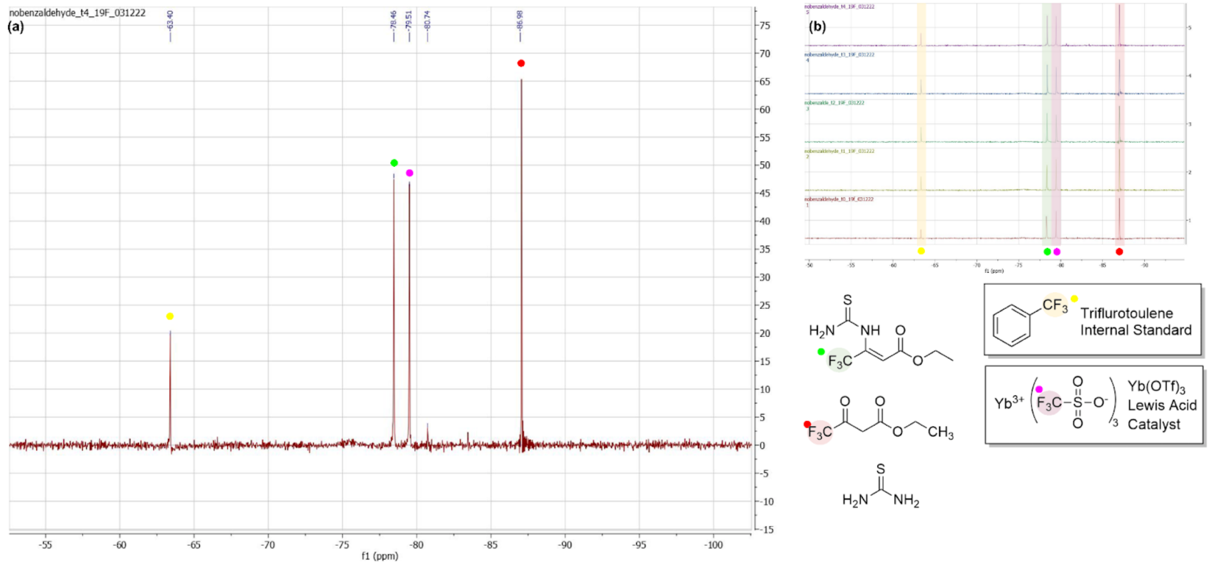

SI-2: Benchtop NMR enables quantitative reaction monitoring of the Biginelli reaction. (a) *^{19}F NMR spectra of a solution mixture of ethylacetoacetate (δ = -87.10 ppm), thiourea, ytterbium (III) triflate (δ = -79.52 ppm), and trifluorotoluene (δ = -63.40 ppm) after 120 minutes* ***(b)*** *Timecourse stacked ^{19}F NMR spectra taken every 30 minutes of a reaction between ethyl 4,4,4,-trifluoroacetoacetate (ETFAA; ^{19}F resonance shown in red) and thiourea to form intermediate* ***E-3*** *(shown in green, δ = -78.48 ppm).*

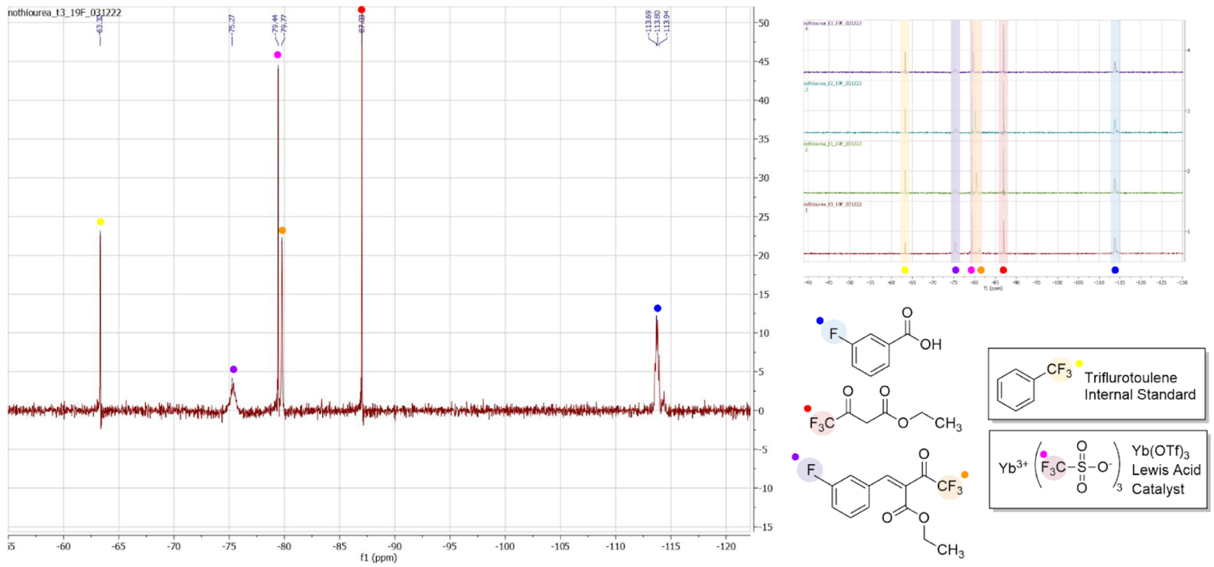

SI-3: Benchtop NMR enables quantitative reaction monitoring of the Biginelli reaction. (a) *^{19}F NMR spectra of a solution mixture of ethylacetoacetate (δ = -87.10 ppm), 3-fluorobenzaldehyde (δ = -113.16 ppm), ytterbium (III) triflate (δ = -79.52 ppm), and trifluorotoluene (δ = -63.40 ppm) after 90 minutes* ***(b)***

Timecourse stacked ^{19}F NMR spectra taken every 30 minutes of a reaction. Knovenagel product, highlighted in orange, ***K-2*** *(δ = -79.77 ppm) is formed.*

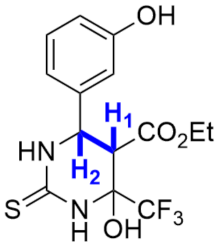 **Stereoisomer**	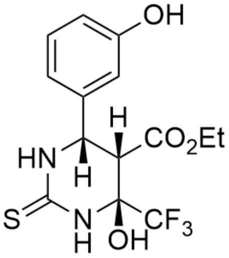	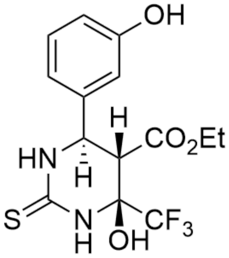	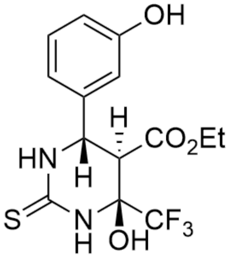	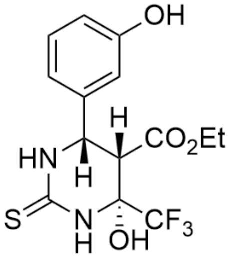
	4R,5R,6R	*4S,5R,6R*	*4R,5S,6R*	*4R,5R,6S*
**DFT Optimized Structures**	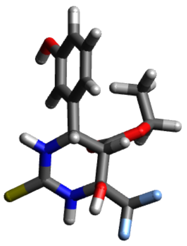	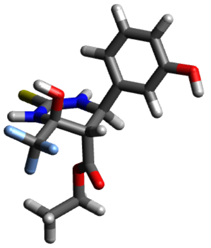	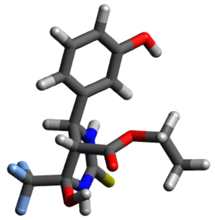	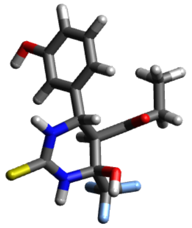
**Relative Energy (kcal/mol)**	1.33	3.98	0	4
**H_1-H_2 Dihedral Angle**	61.1º	284.2º	172.8º	56.5º
**Predicted J-Value (Hz)**	2.33	1.03	11.58	2.92

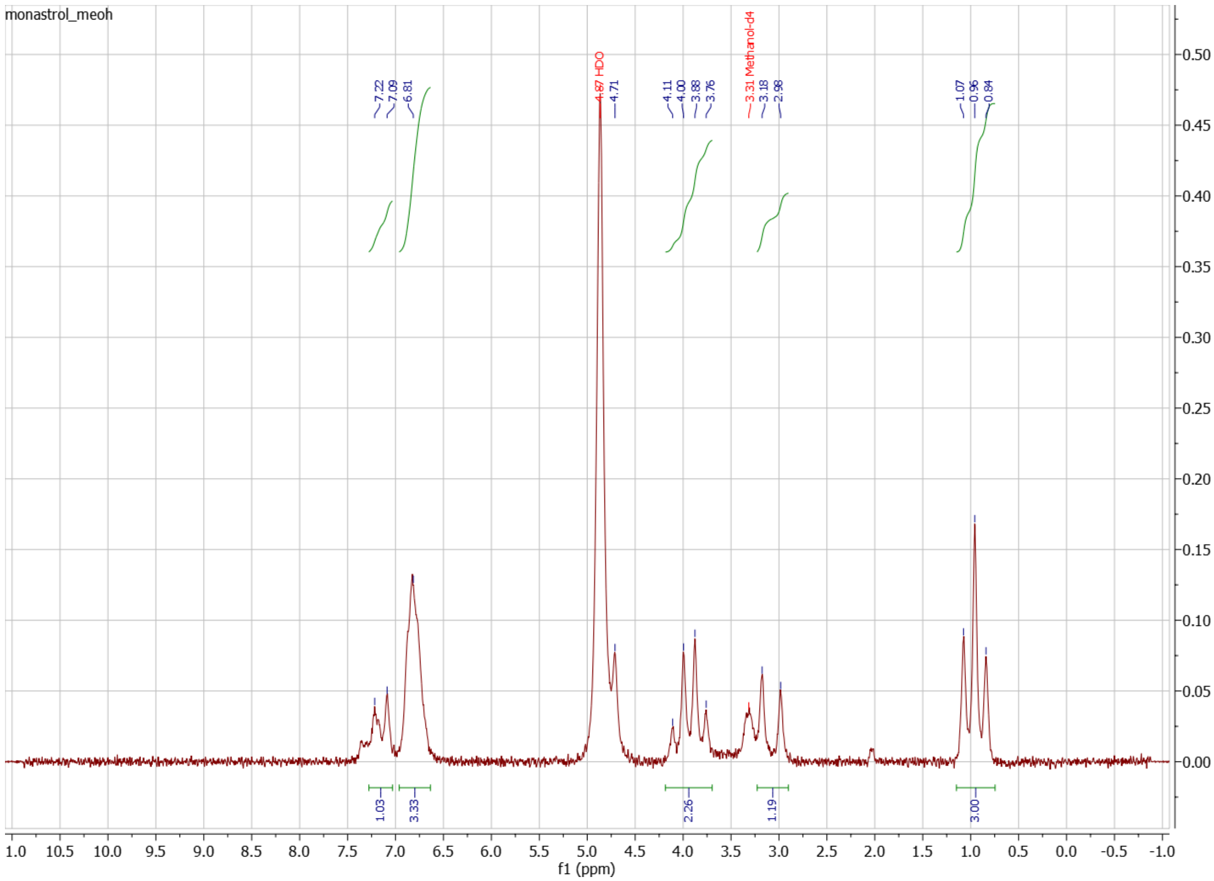

[1]***H NMR (60 MHz, Methanol-d_4)*** *δ 7.15 (d, J = 7.8 Hz, 1H), 6.81 (s, 3H), 4.71 (d, J = 11.7, 1H), 3.94 (q, J = 7.0 Hz, 2H), 3.08 (d, J = 11.7 Hz, 1H), 0.96 (t, J = 7.1 Hz, 3H).*

SI-4: Computational Modeling of Relative Free Energies and Predicted J-Values of Possible Tetrahydropyrimidinone Stereoisomers. Here, we utilized density functional theory to calculate relative single point energies of all four possible stereoisomers of the resulting tetrahydropyrimidinone of the Biginelli cyclocondensation. The lowest energy stereoisomer, with a H2-H3 dihedral of 187.2º degrees, has a predicted J-value (11.58 Hz) is consistent with those observed on our spectra (11.6 Hz in DMSO-d6, 11.7 in CD_3OD)[5]. DFT optimized structures were calculated at the B3LYP / 6-31G(d,p) levels of theory.

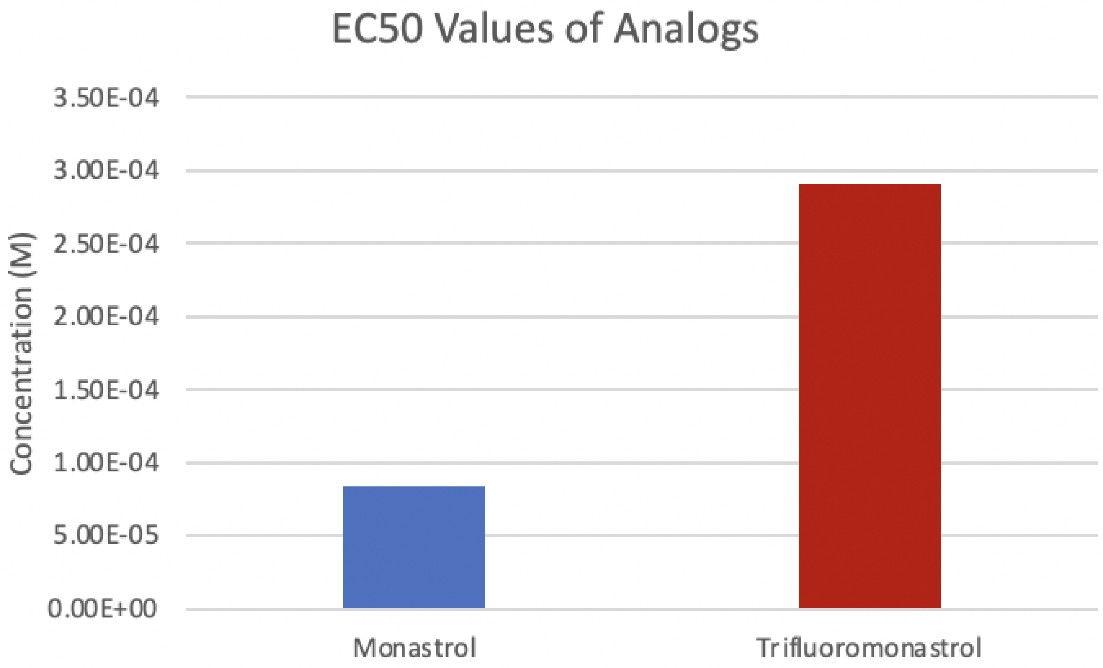

***SI-5*: Comparative cell viability of HCT cells treated with Monastrol and Trifluoromonastrol.**
Calculated EC50 values for non-fluorinated monastrol (left) and trifluorinated monastrol (right) on HCT-116 cells.

Characterization:

ethyl (4*S*,5*R*)-4-hydroxy-6-phenyl-2-thioxo-4-(trifluoromethyl)hexahydropyrimidine-5-carboxylate (Compound 3a - no substitution):

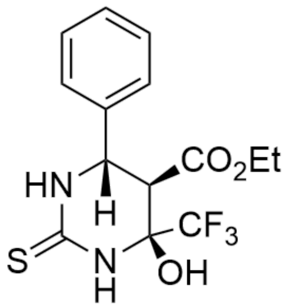

Following the general procedure yielded a white solid (212.38 mg, 60.97% qNMR isolated yield).

MW: 348.34 g/mol

Experimentalists: RC, PS

TLC R_f = 0.725 (50% EtOAc/Hex), UV active

LCMS *m/z:* Calcd for $C_{14}H_{16}F_3N_2O_3S^+$ 350.09 $[M+H]^+$, Found 350.15 $[M+H]^+$

^{1}H NMR (60 MHz, DMSO-d_6) δ 9.08 (s, 1H), 8.89 (s, 1H), 7.86 (s, 1H), 7.33 (s, 5H), 4.78 (d, J = 11.9 Hz, 1H), 3.79 (q, J = 7.2 Hz, 2H), 3.01 (d, J = 12.2 Hz, 2H), 0.80 (t, J = 7.1 Hz, 3H).

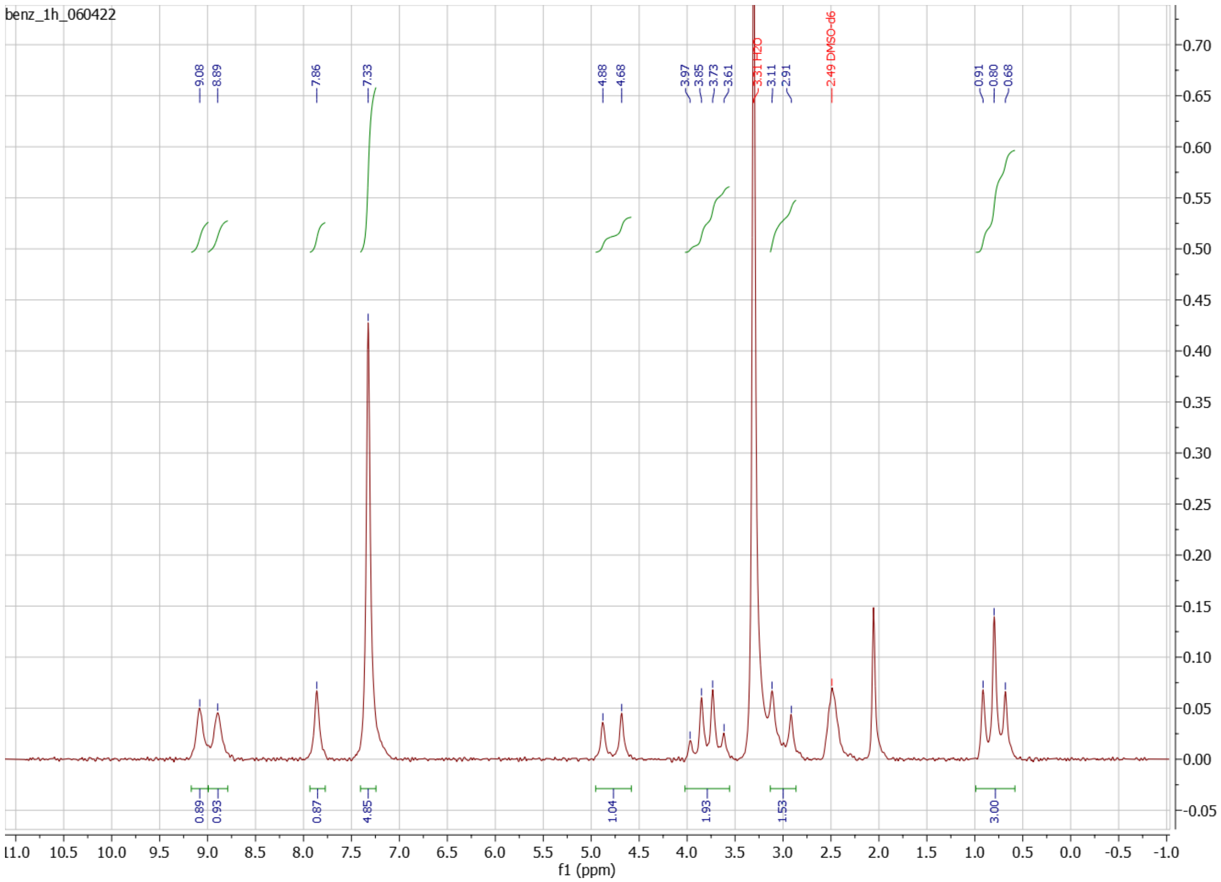

^{13}C NMR (15 MHz, DMSO-d_6) δ 177.22, 166.52, 136.95, 128.43, 128.15, 60.45, 54.33, 49.37, 13.40.

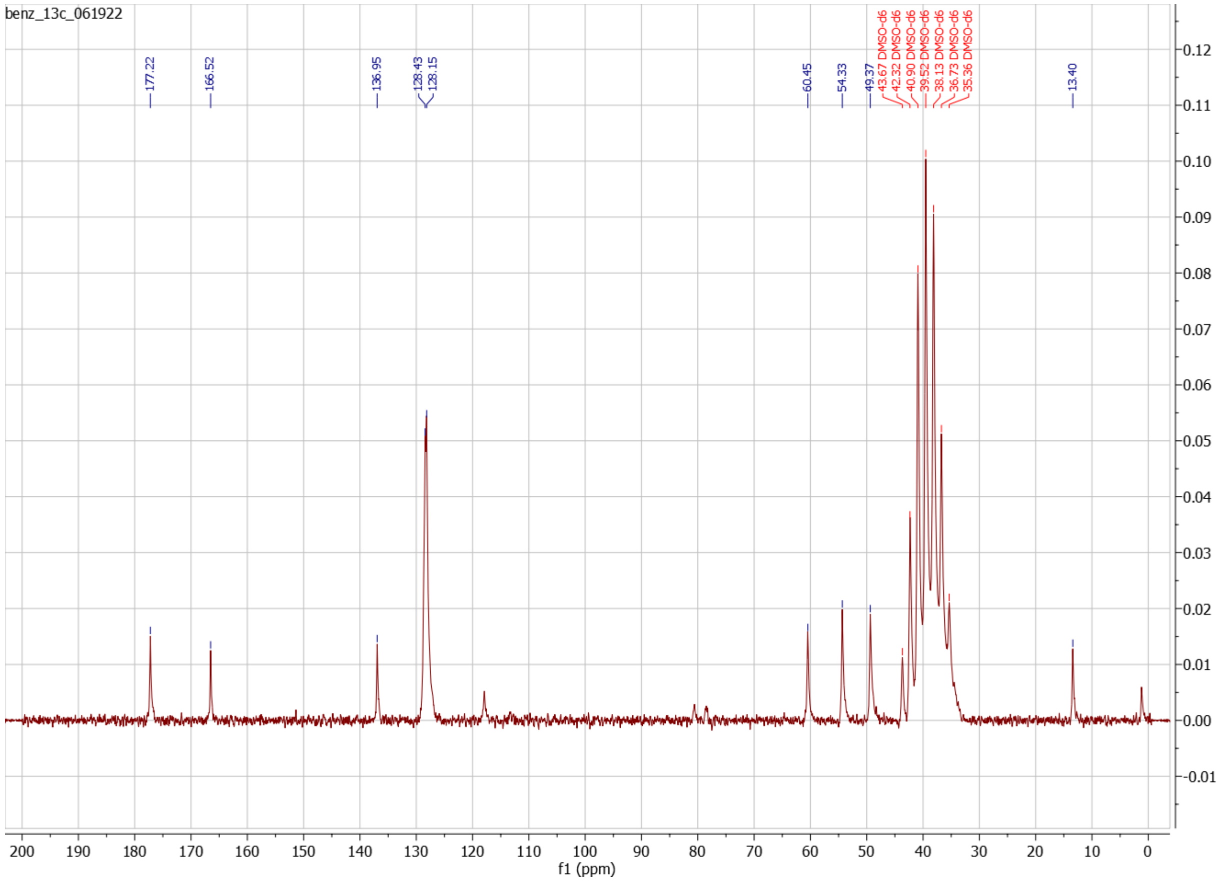

^{19}F NMR (60 MHz, DMSO-d_6) δ -80.40.

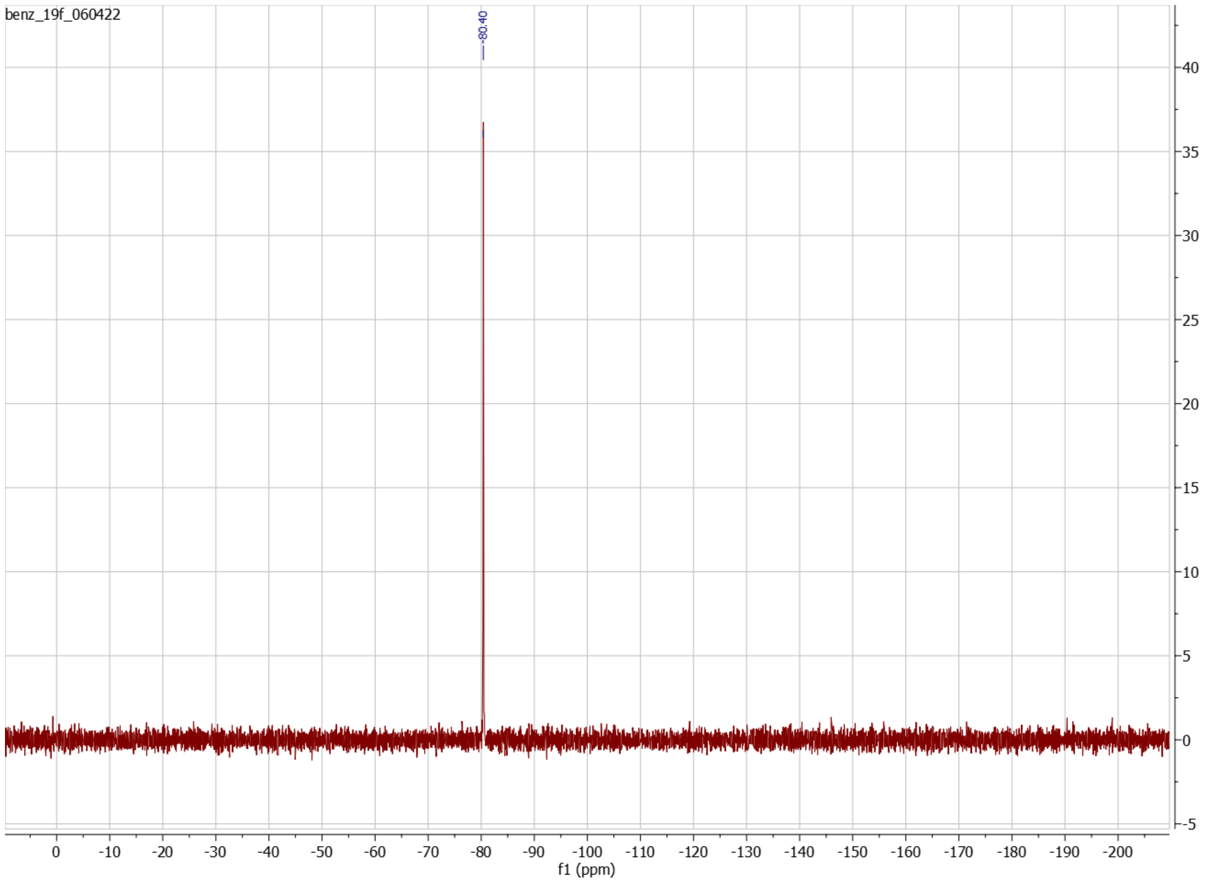

FT-IR (neat, ATR): 3201.07, 1732.56, 1556.9, 1494.95, 1457.3, 1377.52, 1344.14, 1242.82, 1191.79, 1022.73, 698.98

COSY 2D NMR (60 MHz, DMSO-d_6)

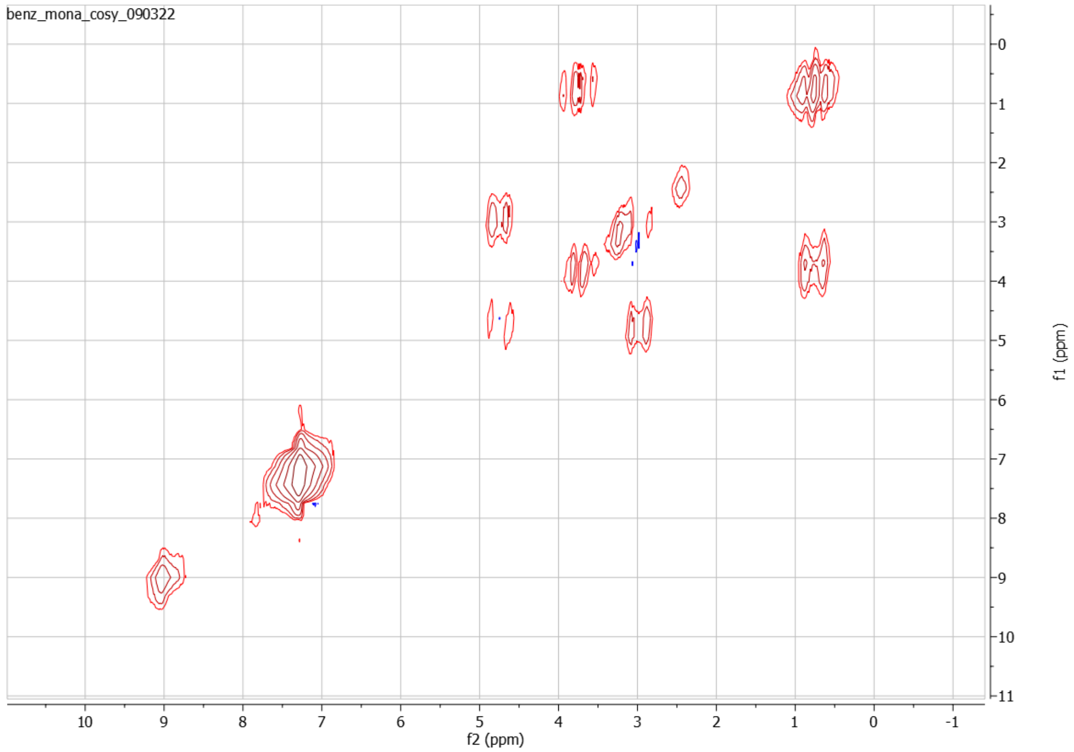

ethyl (4*S*,5*R*)-4-hydroxy-6-(3-methoxyphenyl)-2-thioxo-4-(trifluoromethyl)hexahydropyrimidine-5-carboxylate (Compound 3b - 3-methoxy substitution):

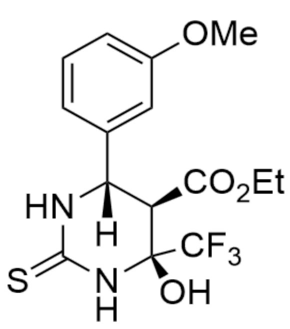

Following the general procedure yielded a white solid (170.64 mg, 45.10% qNMR isolated yield).

MW: 378.37 g/mol

Experimentalists: RC, SS

TLC R_f = 0.65 (50% EtOAc/Hex), UV active

LCMS *m/z:* Calcd for $C_{15}H_{18}F_3N_2O_4S^+$ 379.09 $[M+H]^+$, Found 379.16 $[M+H]^+$

^{1}**H NMR** (60 MHz, DMSO-d_6) δ 9.05 (s, 1H), 7.20 (d, J = 6.7 Hz, 1H), 6.87 (d, J = 5.7 Hz, 3H), 4.76 (d, J = 11.8 Hz, 1H), 3.81 (q, J = 7.1 Hz, 2H), 3.70 (s, 3H), 3.04 (d, J = 11.8 Hz, 1H), 0.84 (t, J = 7.1 Hz, 3H).

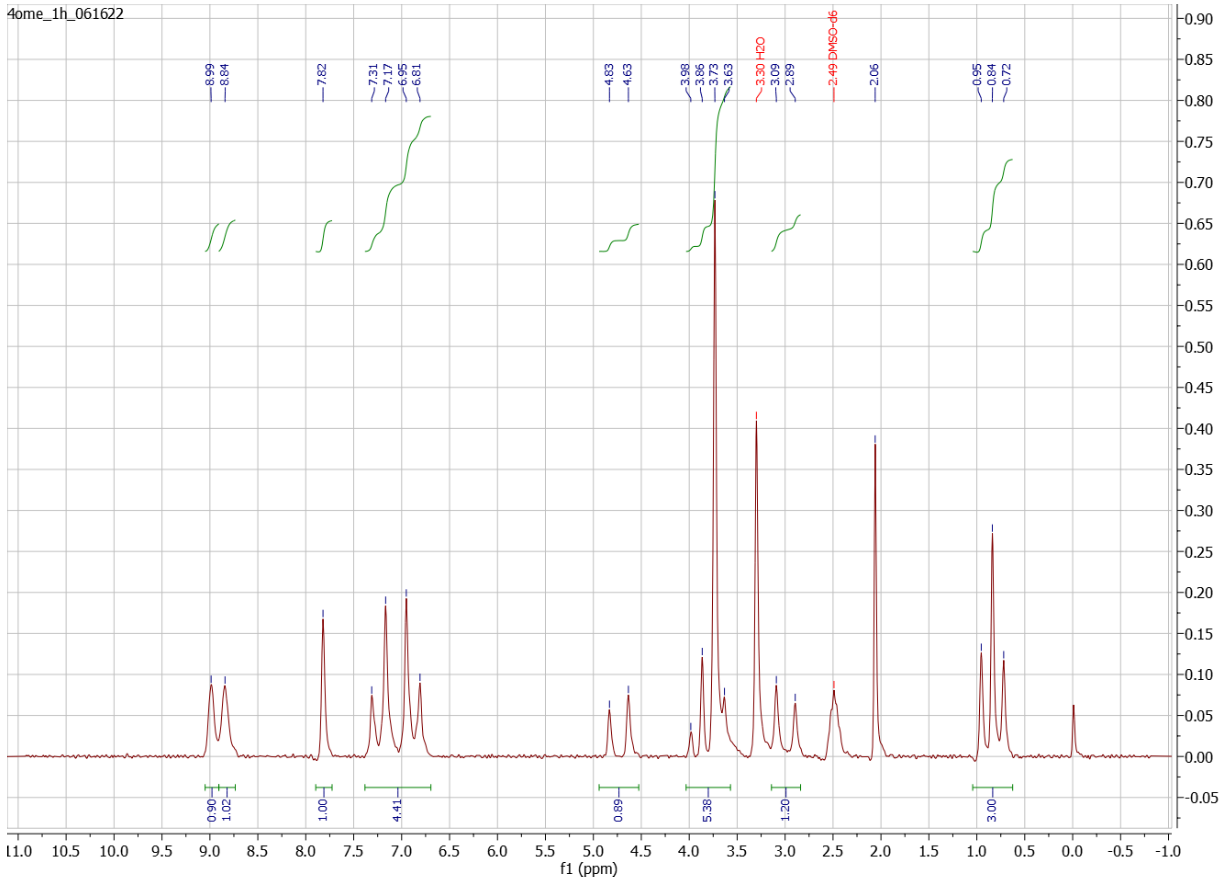

^{13}C NMR (15 MHz, DMSO-d_6) δ 177.10, 166.50, 159.11, 138.38, 129.53, 120.22, 113.80, 60.50, 55.21, 54.20, 49.09, 13.46.

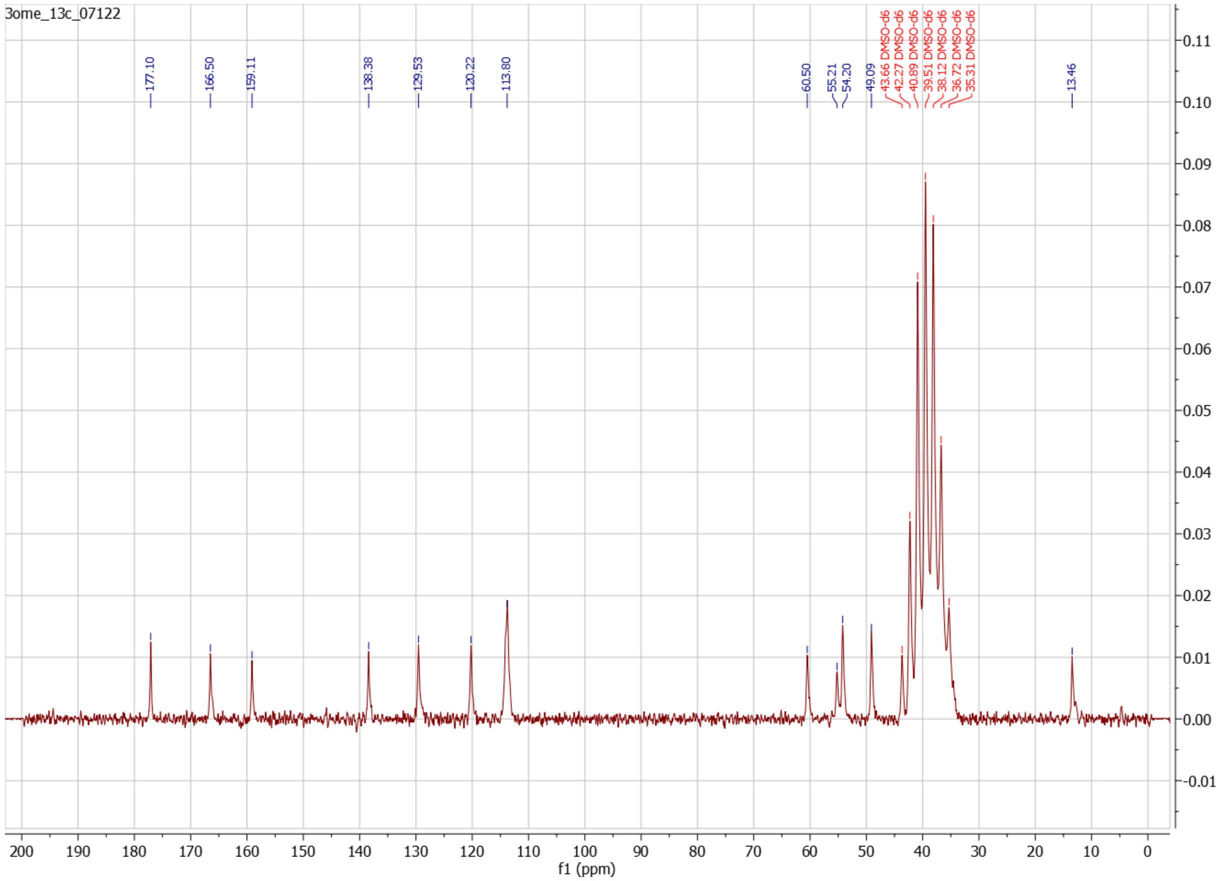

^{19}F NMR (60 MHz, DMSO-d_6) δ -80.35.

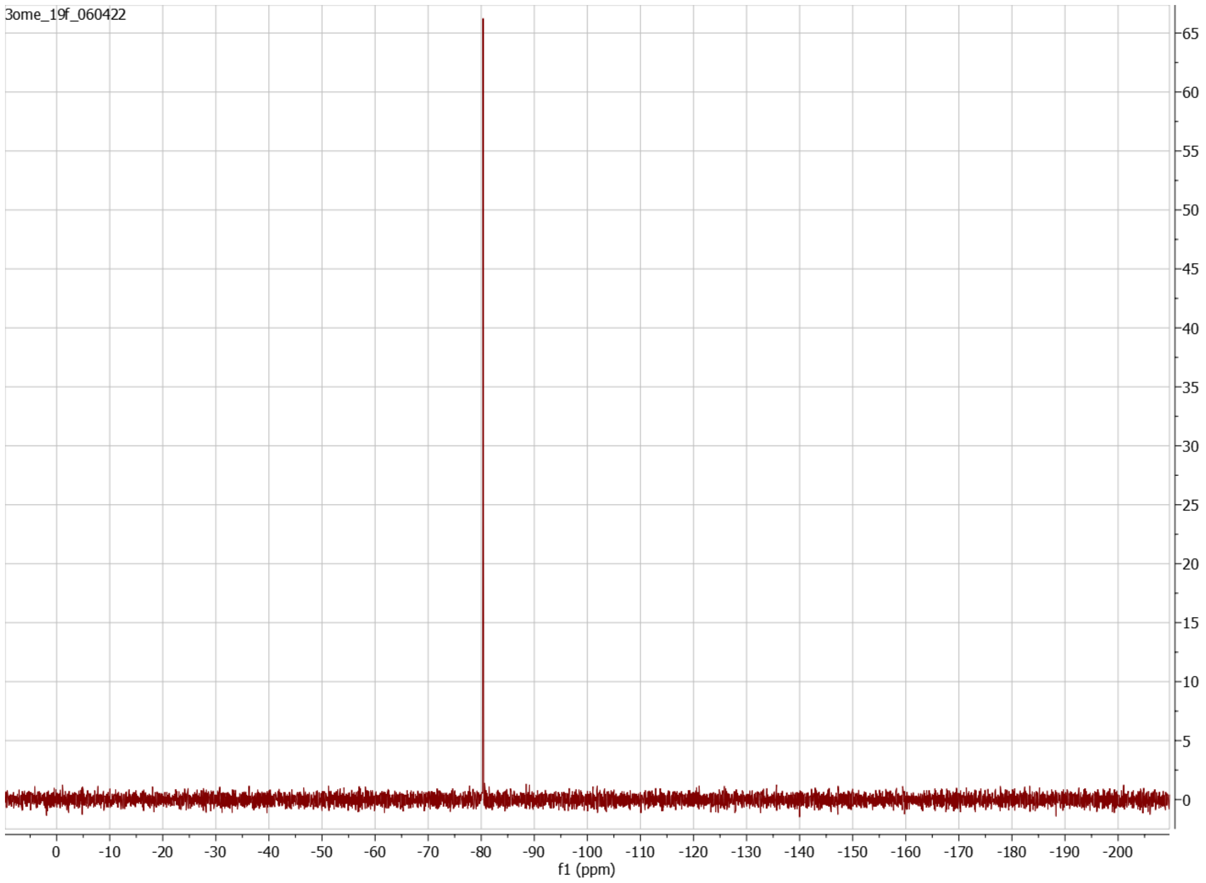

FT-IR (neat, ATR): 3207.09, 2980.57, 1740.58, 1600, 1562.94, 1505.65, 1470.91, 1434.59, 1397.57, 1373.87, 1349.48, 1326.25, 1265.1, 1193.1, 1160.47, 1137.59, 1105.54, 1083.72, 1032.87, 698.34

COSY 2D NMR (60 MHz, DMSO-d_6)

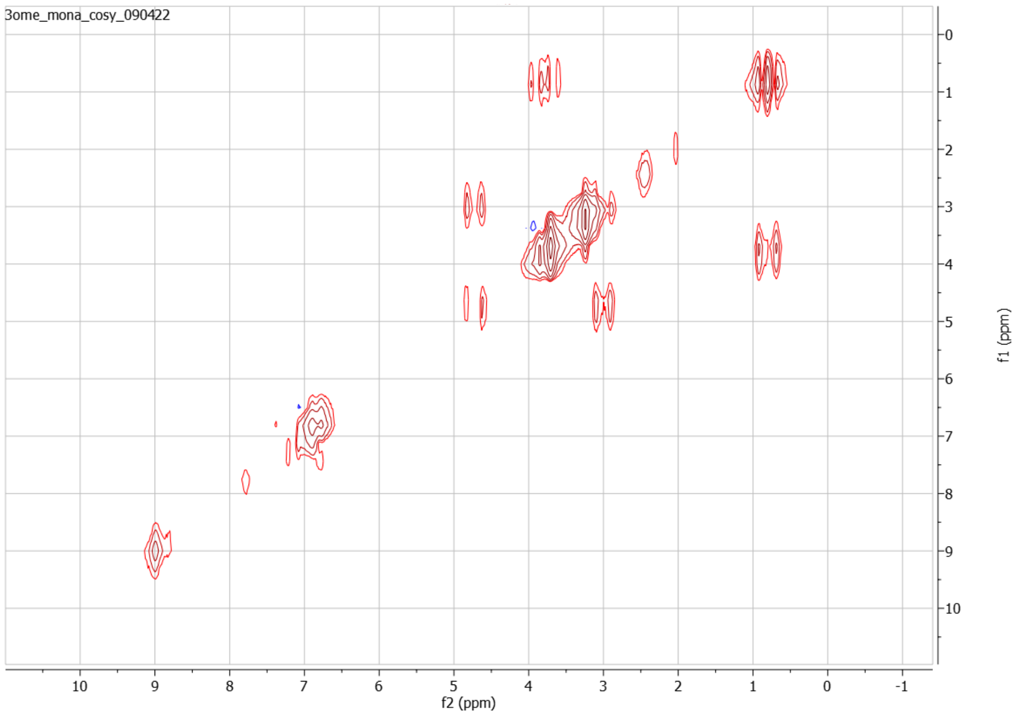

ethyl (4*S*,5*R*)-4-hydroxy-6-(3-hydroxyphenyl)-2-thioxo-4-(trifluoromethyl)hexahydropyrimidine-5-carboxylate (Compound 3c - 3-hydroxy substitution):

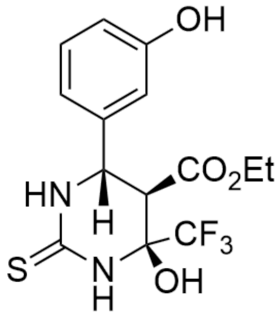

Following the general procedure yielded a white solid (266.70 mg, 73.20% qNMR isolated yield).

MW: 364.34 g/mol

Experimentalists: RC, PS

TLC R_f = 0.375 (50% EtOAc/Hex), UV active

LCMS *m/z:* Calcd for $C_{14}H_{16}F_3N_2O_4S^+$ 365.07 $[M+H]^+$, Found 365.14 $[M+H]^+$

^{1}H NMR (60 MHz, DMSO-d_6) δ 9.05 (s, 1H), 7.28-7.01 (m, 1H), 6.70 (d, J = 5.7 Hz, 3H), 4.69 (d, J = 11.9 Hz, 1H), 3.84 (q, J = 7.0 Hz, 2H), 2.90 (d, J = 11.7 Hz, 1H), 0.86 (t, J = 7.1 Hz, 3H).

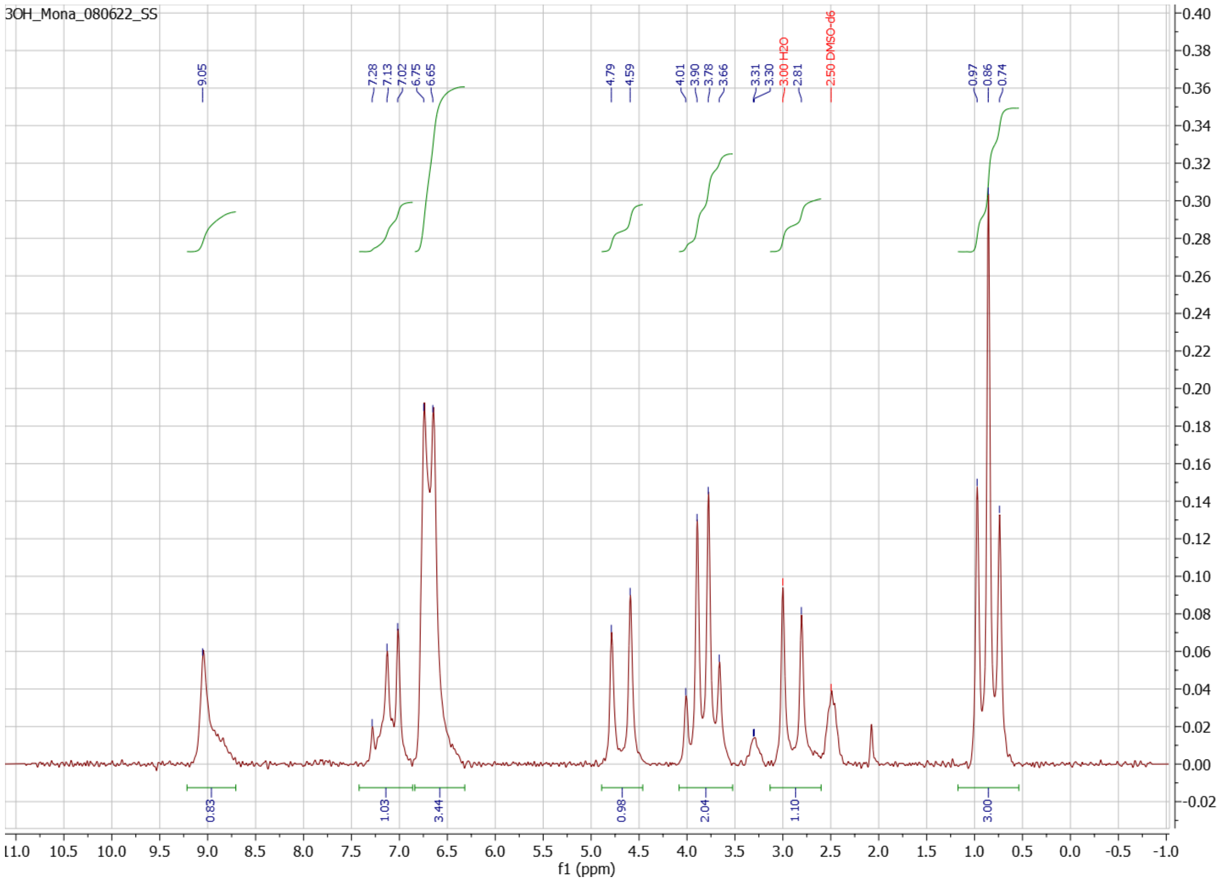

^{13}C NMR (15 MHz, DMSO-d_6) δ 177.23, 166.49, 138.51, 133.59, 132.39, 129.56, 118.46, 111.33, 60.56, 53.76, 48.54, 13.45.

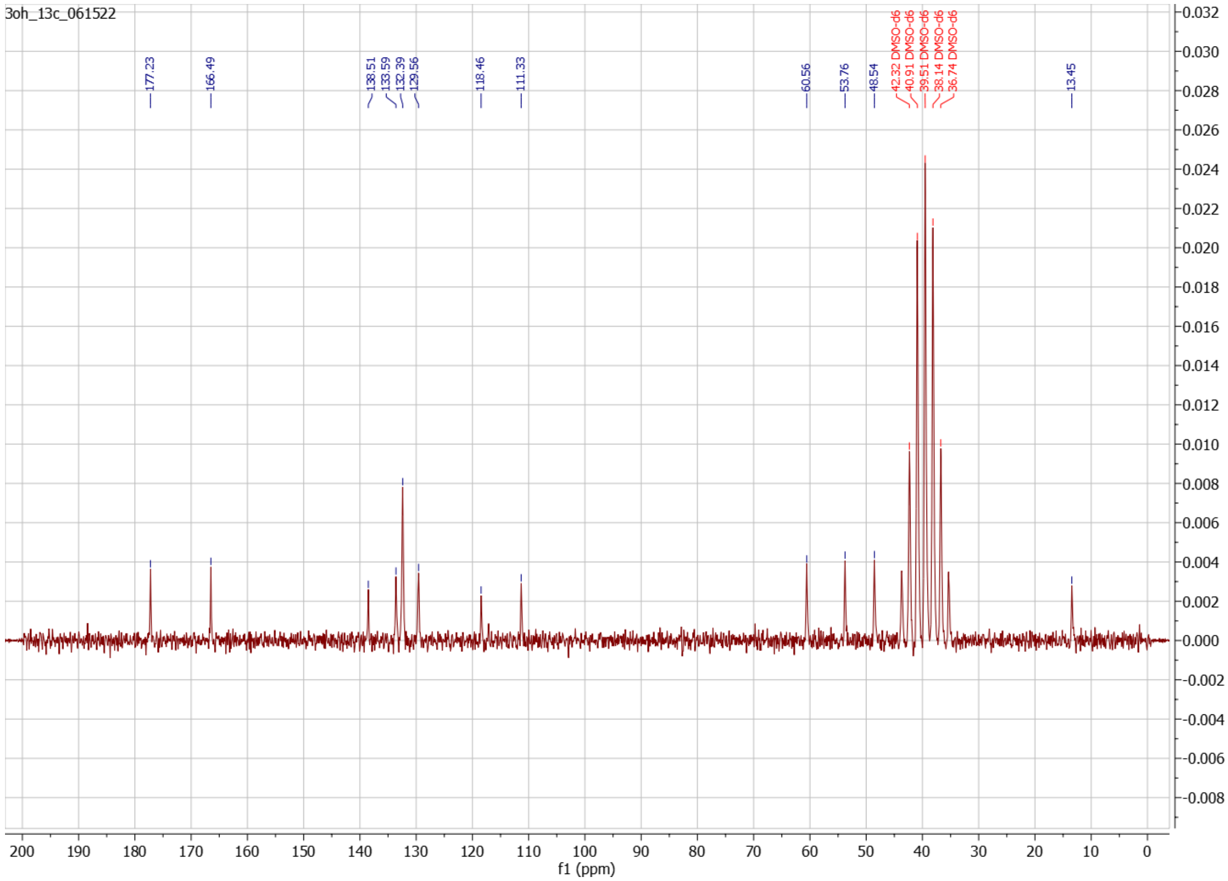

^{19}F NMR (60 MHz, DMSO-d_6) δ -80.50.

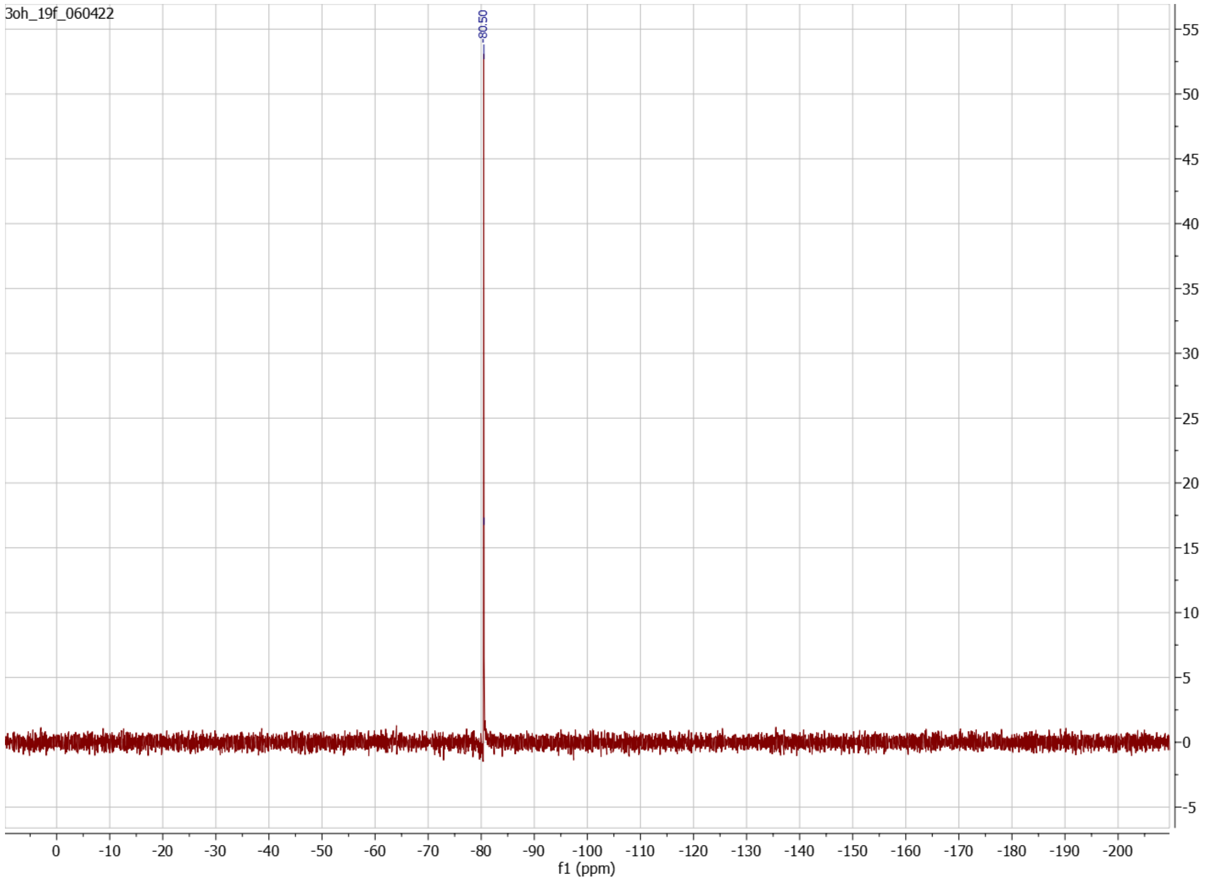

FT-IR (neat, ATR): 3306.16, 1731.9, 1715.6, 1603.35, 1557.16, 1505.2, 1487.73, 1455.7, 1377.64, 1344.73, 1313.25, 1196.43, 1022.32, 788.25, 697.89, 608.48, 595.68, 570.09, 556.14

COSY 2D NMR (60 MHz, DMSO-d_6)

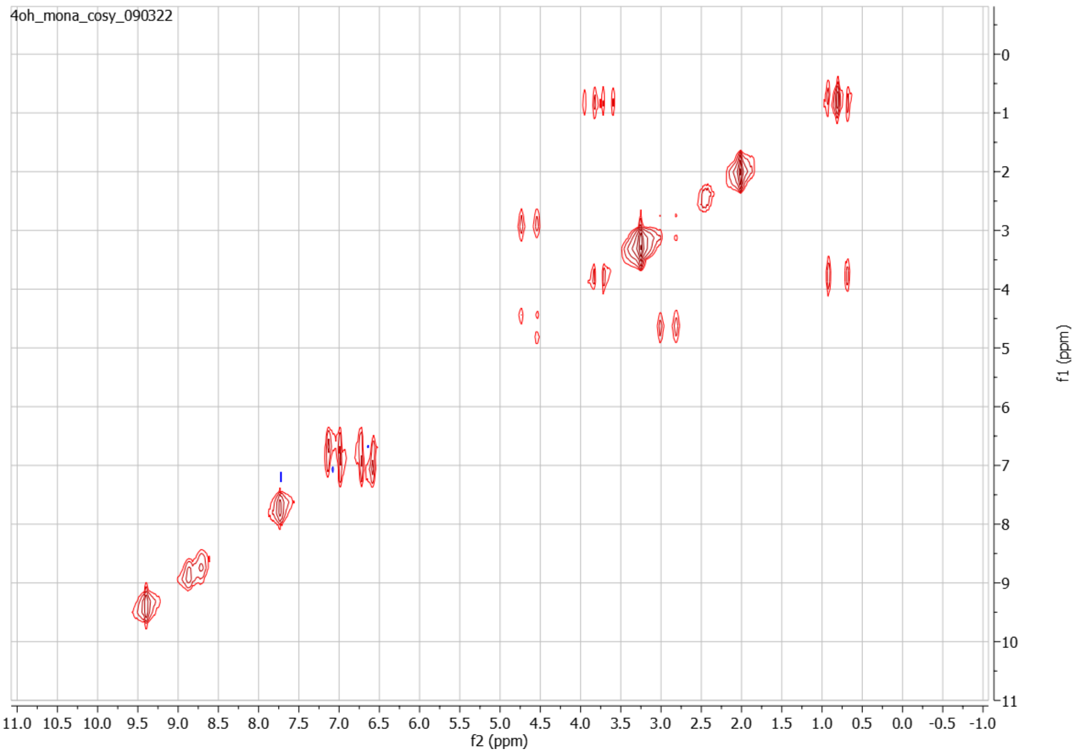

ethyl (4*S*,5*R*)-4-hydroxy-2-thioxo-6-(m-tolyl)-4-(trifluoromethyl)hexahydropyrimidine-5-carboxylate (Compound 3d - 3-methyl substitution):

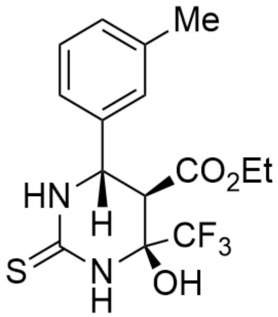

Following the general procedure yielded a white solid (263.15 mg, 72.62% qNMR isolated yield).

MW: 362.37 g/mol

Experimentalists: RC, PS

TLC R_f = 0.75 (50% EtOAc/Hex), UV active

LCMS *m/z:* Calcd for $C_{15}H_{16}F_3N_2O_3S^-$ 361.08 $[M-H]^-$, Found 361.15 $[M-H]^-$

^{1}**H NMR** (60 MHz, DMSO-d_6) δ 9.04 (s, 1H), 8.77 (bs, 1H), 7.80 (bs, 1H), 7.14 (s, 4H), 4.75 (d, J = 11.8 Hz, 1H), 3.81 (q, J = 7.1 Hz, 2H), 2.99 (d, J = 11.8 Hz, 1H), 2.30 (s, 3H), 0.82 (t, J = 7.1 Hz, 3H).

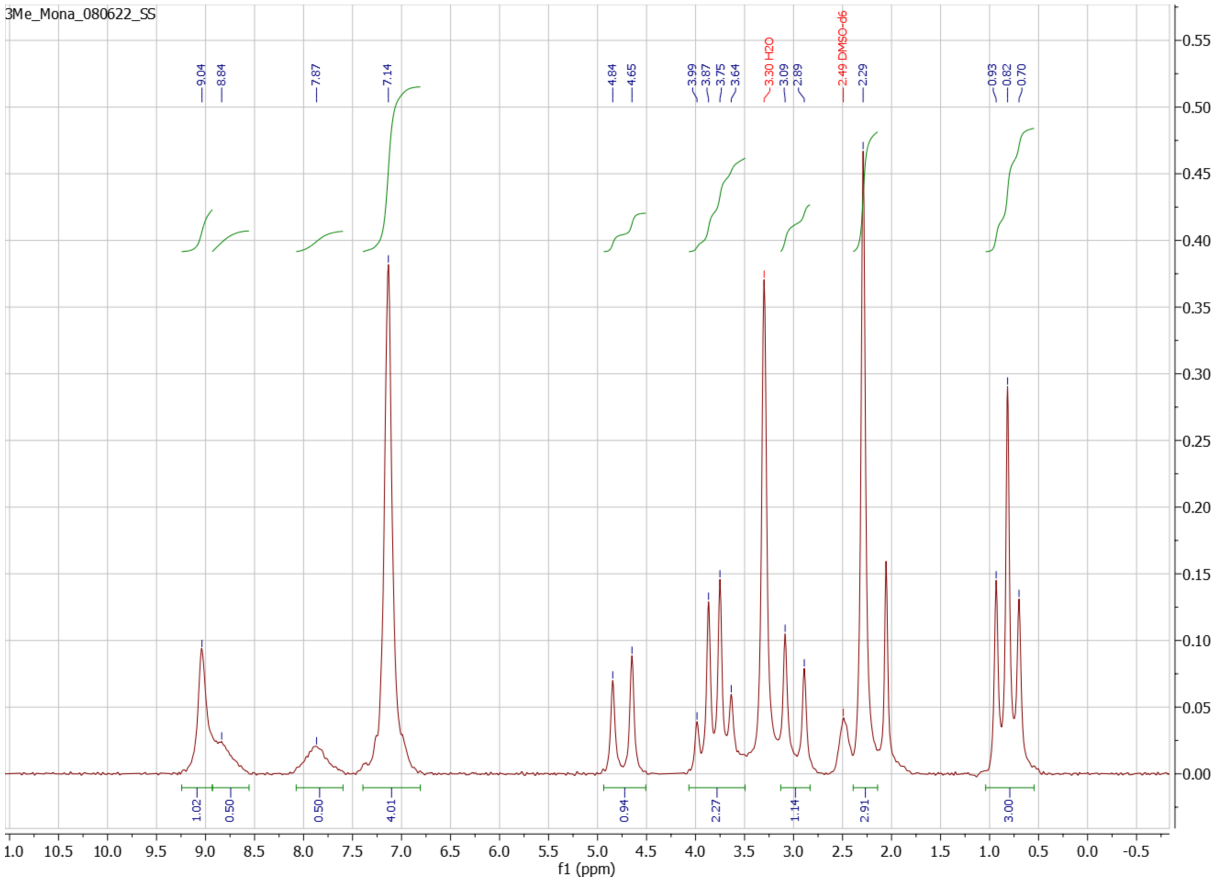

^{13}C NMR (15 MHz, DMSO-d_6) δ 177.17, 166.54, 137.57, 136.89, 129.31, 128.43, 125.24, 60.53, 54.29, 49.38, 20.92, 13.47.

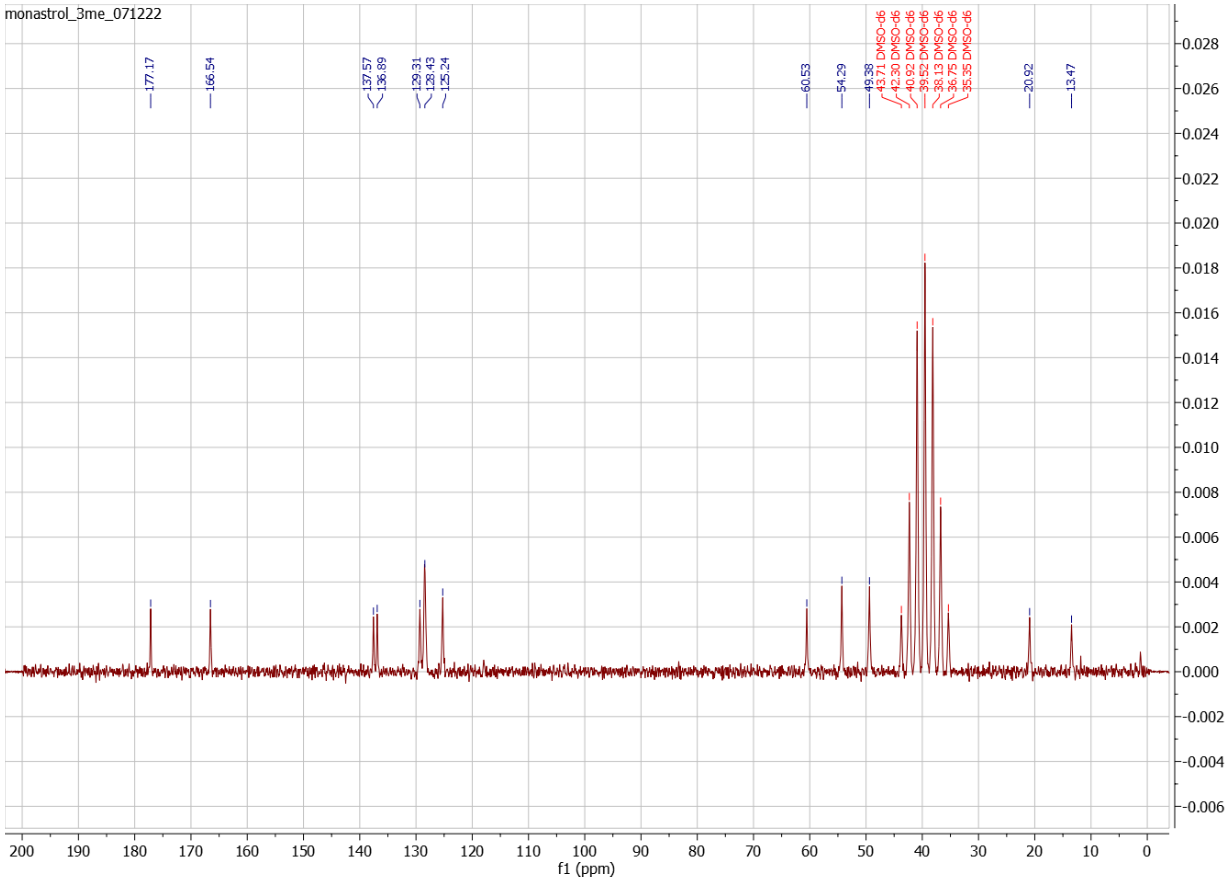

^{19}F NMR (60 MHz, DMSO-d_6) δ -80.41.

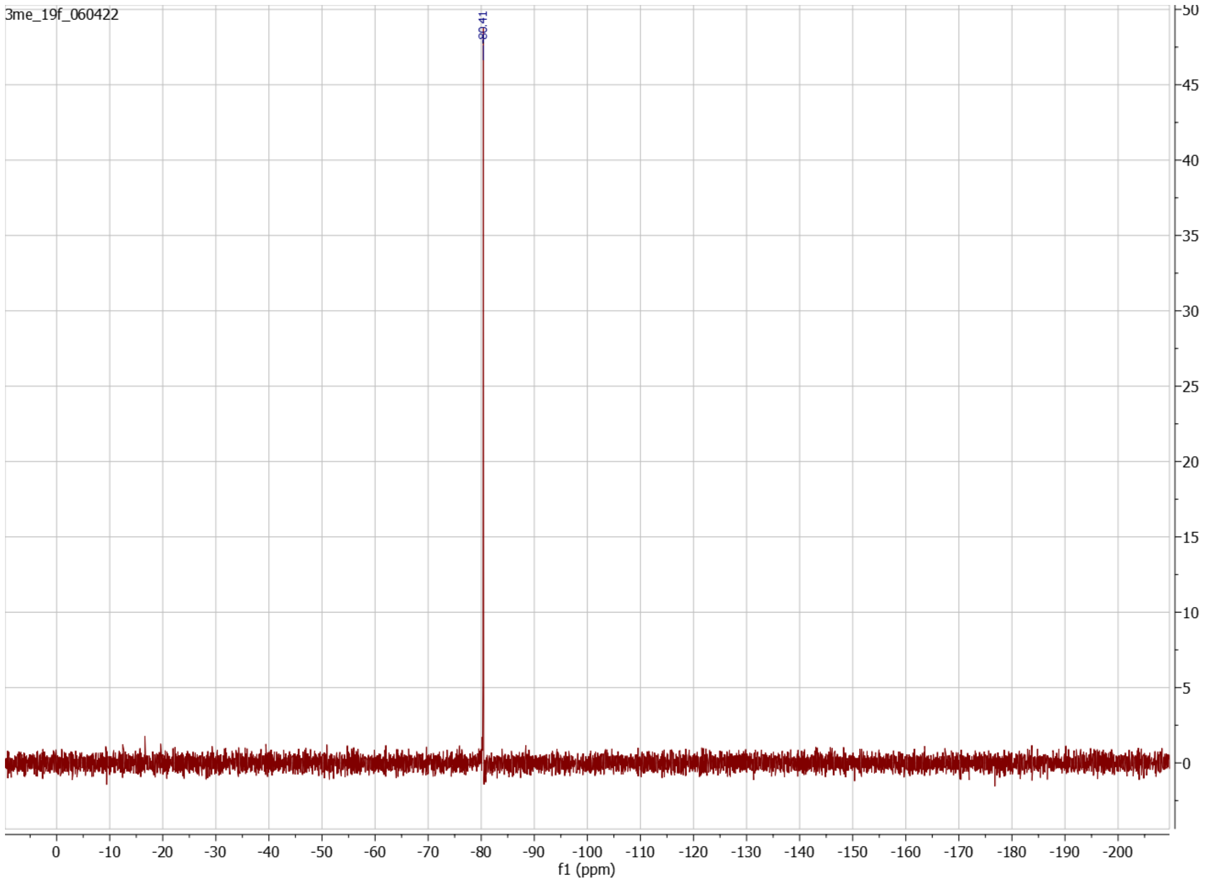

FT-IR (neat, ATR): 3190.95, 1736.08, 1555.12, 1491.69, 1377.11, 1342.81, 1193.87, 1023.25, 787.89, 702.47, 615.94, 609.39, 556.67

COSY 2D NMR (60 MHz, DMSO-d_6)

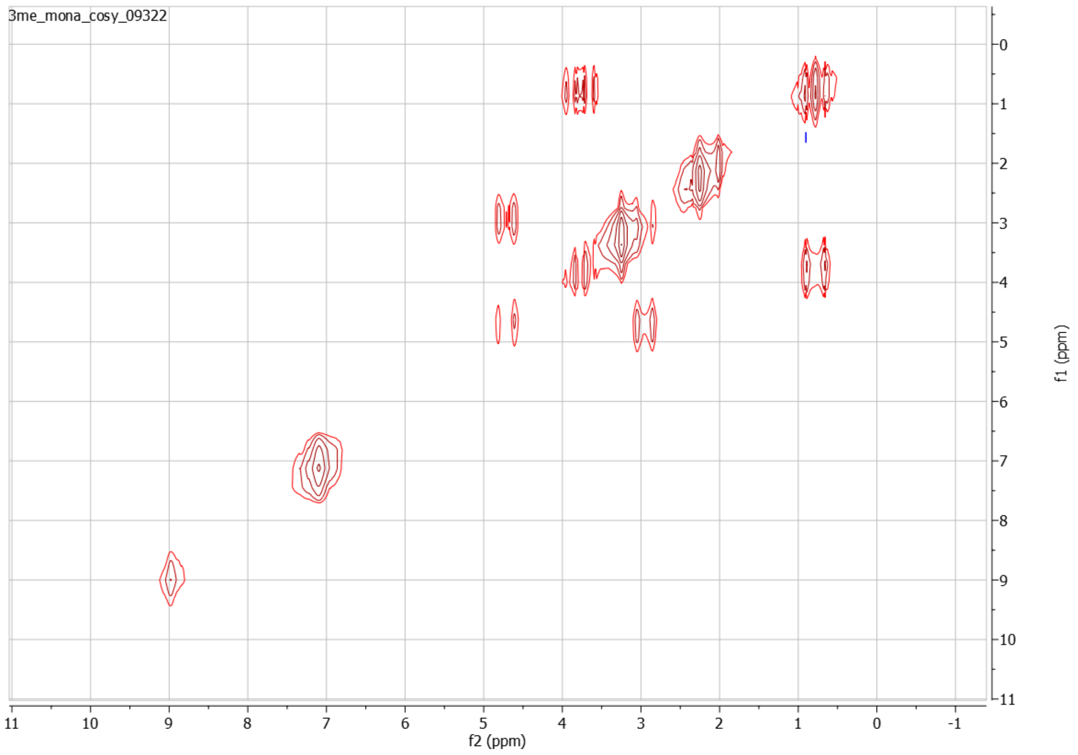

ethyl (4*S*,5*R*)-6-(3-bromophenyl)-4-hydroxy-2-thioxo-4-(trifluoromethyl)hexahydropyrimidine-5-carboxylate (Compound 3e - 3-bromo substitution):

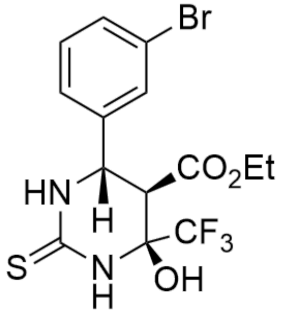

Following the general procedure yielded a white solid (353.88 mg, 82.83% qNMR isolated yield).

MW: 427.24 g/mol

Experimentalists: RC, PS

TLC R_f = 0.725 (50% EtOAc/Hex), UV active

LCMS *m/z:* Calcd for $C_{14}H_{15}BrF_3N_2O_3S^+$ 426.99 $[M+H]^+$, Found 427.08 $[M+H]^+$

^{1}H NMR (60 MHz, DMSO-d_6) δ 9.14 (s, 1H), 8.95 (bs, 1H), 7.88 (bs, 1H), 7.44 (d, J = 11.7 Hz, 4H), 4.78 (d, J = 11.8 Hz, 1H), 3.83 (q, J = 7.1 Hz, 2H), 3.11 (d, J = 11.7 Hz, 1H), 0.83 (t, J = 7.1 Hz, 3H).

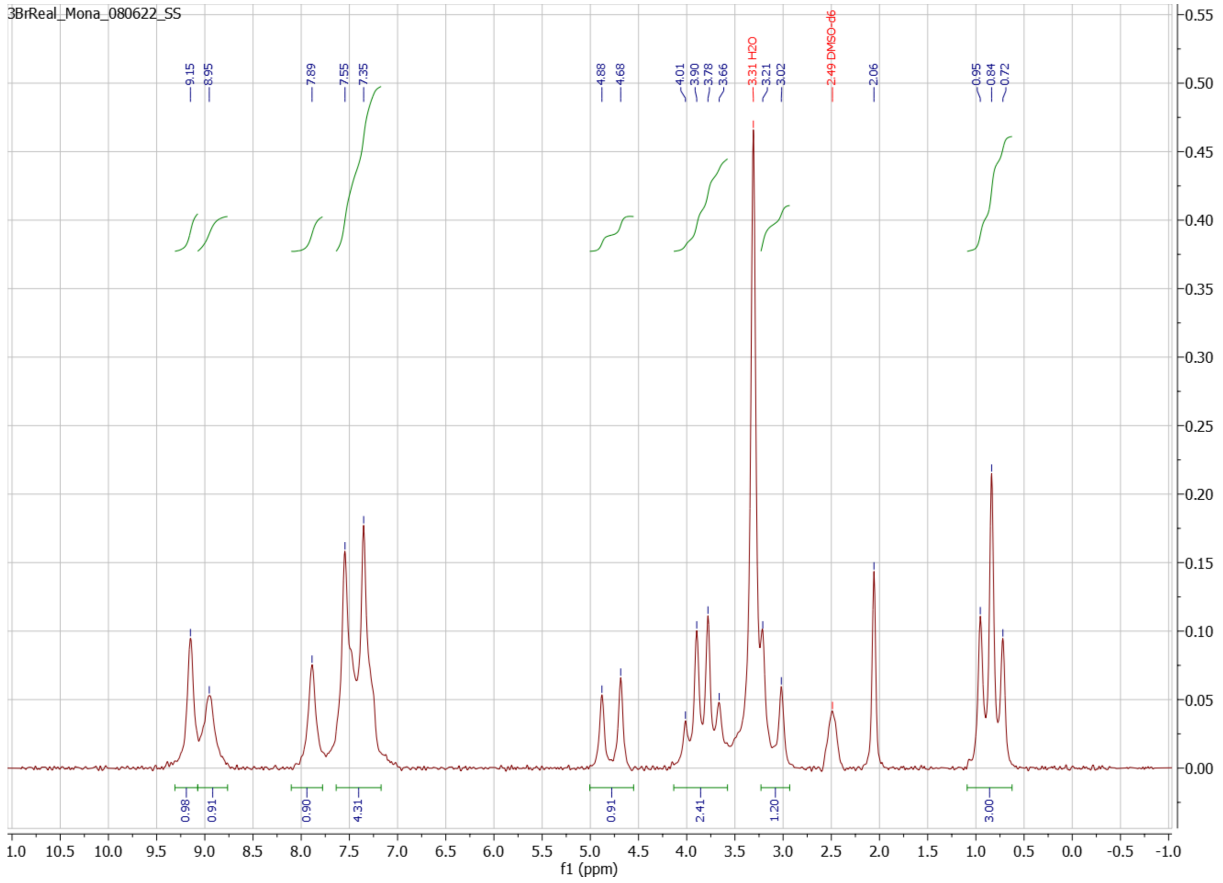

^{13}C NMR (15 MHz, DMSO-d_6) δ 177.16, 166.55, 157.32, 138.40, 129.49, 118.58, 115.68, 114.74, 60.52, 54.22, 49.54, 13.48.

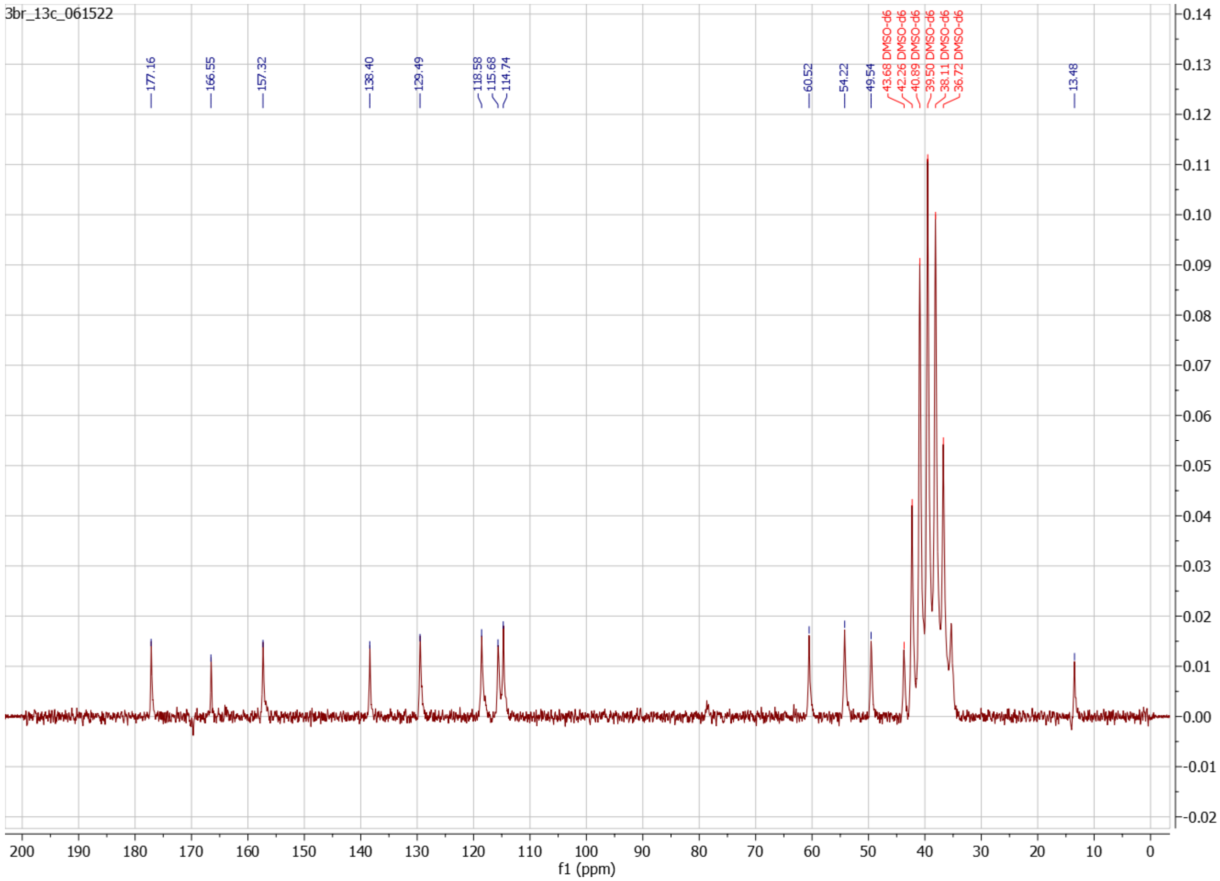

^{19}F NMR (60 MHz, DMSO-d_6) δ -80.22.

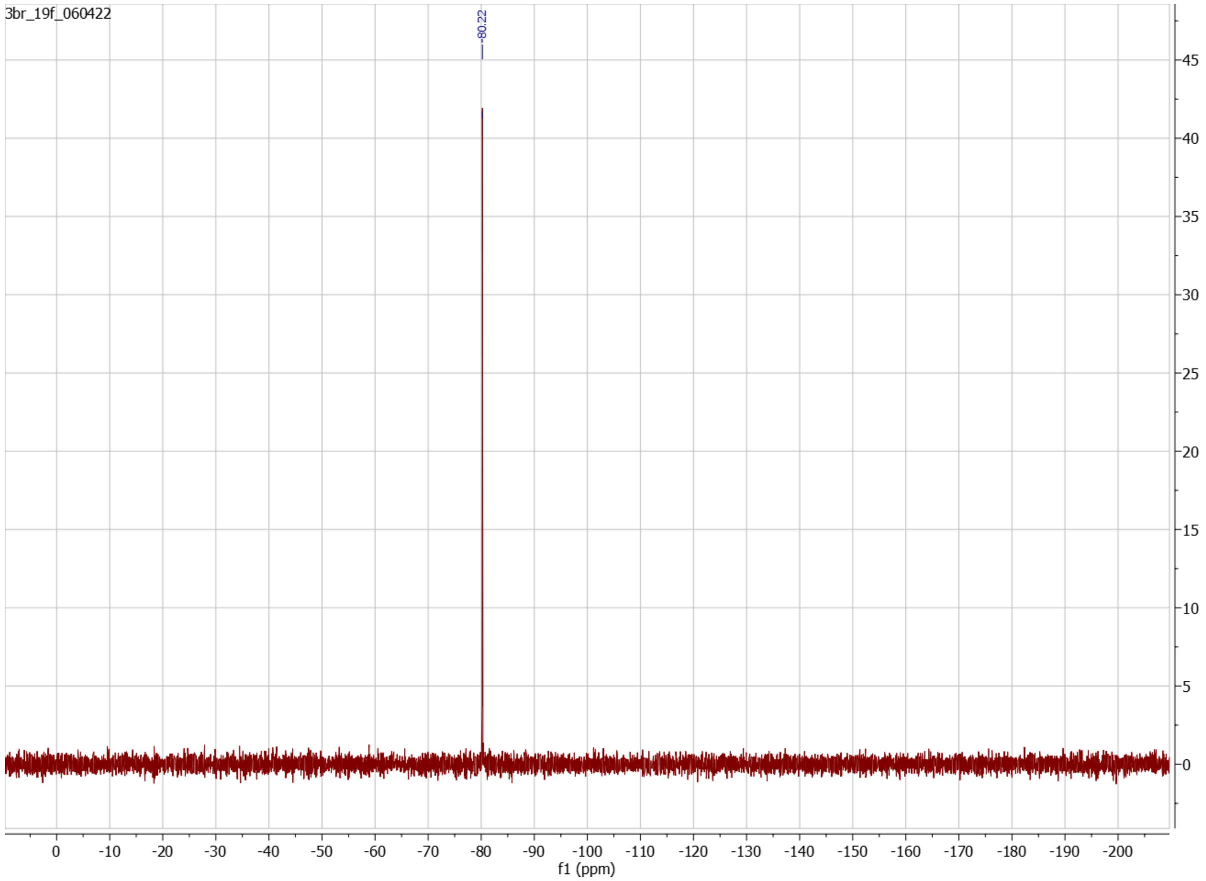

FT-IR (neat, ATR): 3190.24, 2982.16, 1731.84, 1556.62, 1477.68, 1434.4, 1376.79, 1342.69, 1304.35, 1243.27, 1190.29, 1097.46, 1023.56, 787.38, 715.92, 693.66, 584.41, 574.49, 559.83

COSY 2D NMR (60 MHz, DMSO-d_6)

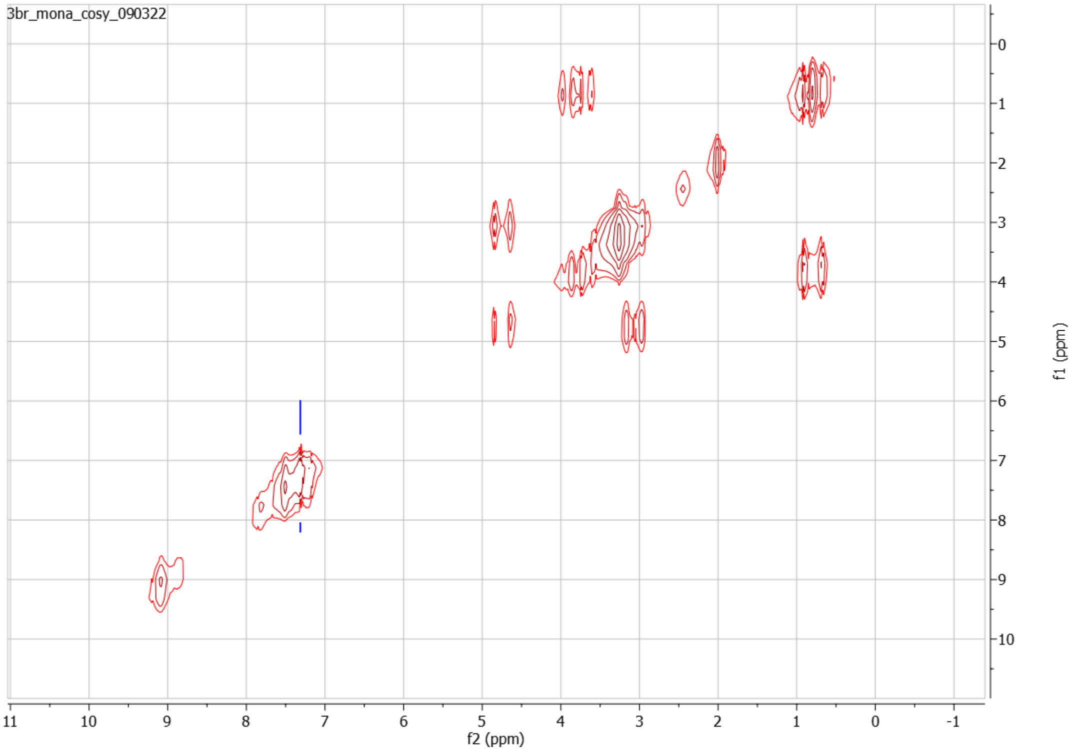

ethyl (4*S*,5*R*)-6-(3-chlorophenyl)-4-hydroxy-2-thioxo-4-(trifluoromethyl)hexahydropyrim idine-5-carboxylate (Compound 3f - 3-chloro substitution):

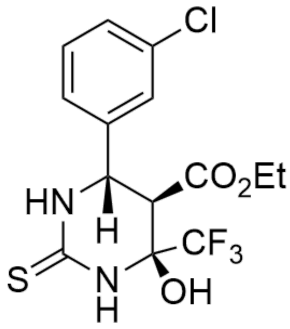

Following the general procedure yielded a white solid (230.43 mg, 60.20% qNMR isolated yield).

MW: 382.78 g/mol

Experimentalists: RC, PS

TLC R_f = 0.725 (50% EtOAc/Hex), UV active

LCMS *m/z:* Calcd for $C_{14}H_{15}ClF_3N_2O_3S^+$ 383.84 $[M+H]^+$, Found 383.77 $[M+H]^+$

^{1}**H NMR** (60 MHz, DMSO-d_6) δ 9.14 (s, 1H), 8.96 (bs, 1H), 7.89 (bs, 1H), 7.36 (s, 4H), 4.80 (d, J = 11.8 Hz, 1H), 3.84 (q, J = 7.1 Hz, 2H), 3.12 (d, J = 11.9 Hz, 1H), 0.84 (t, J = 7.1 Hz, 3H).

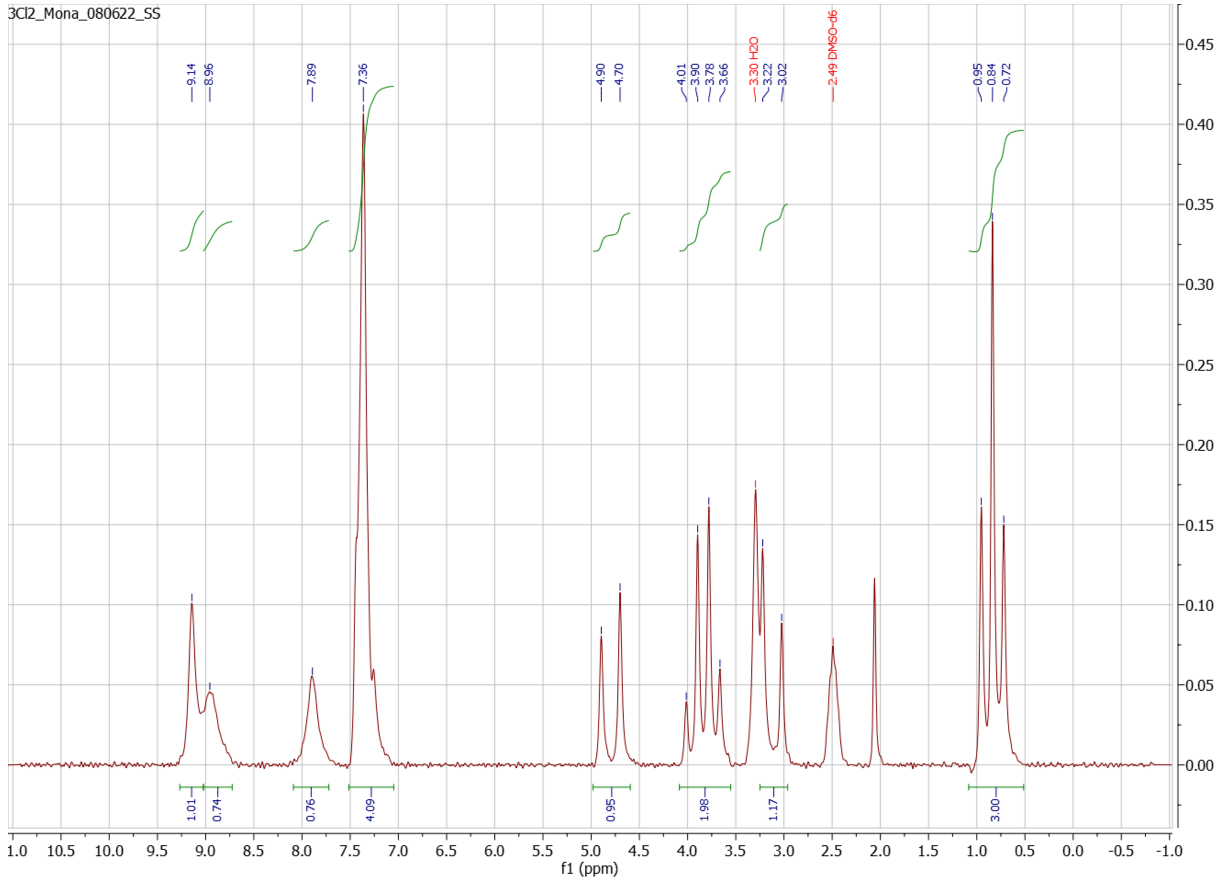

^{13}C NMR (15 MHz, DMSO-d_6) δ 177.14, 166.37, 135.85, 133.15, 130.12, 128.32, 60.51, 53.59, 49.02, 13.40.

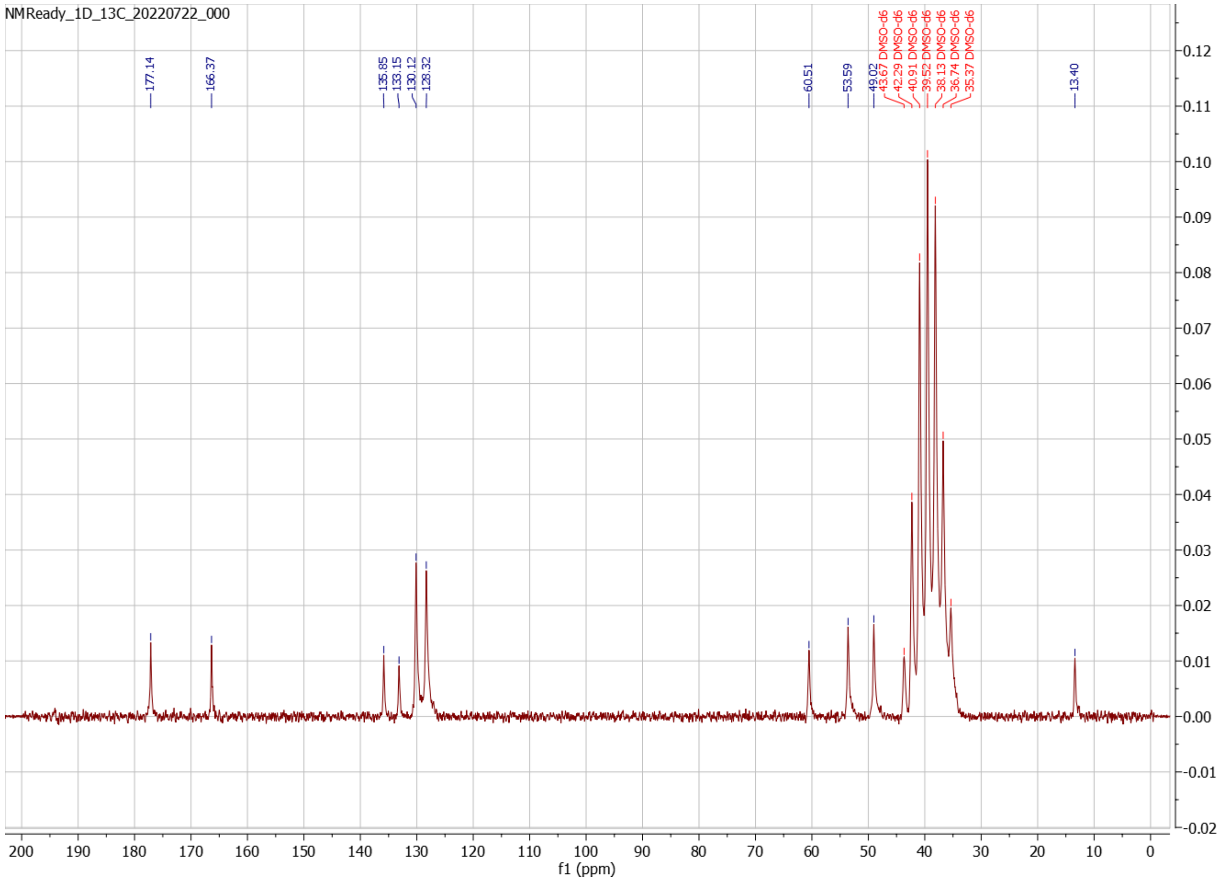

^{19}**F NMR** (60 MHz, DMSO-d_6) δ -80.22.

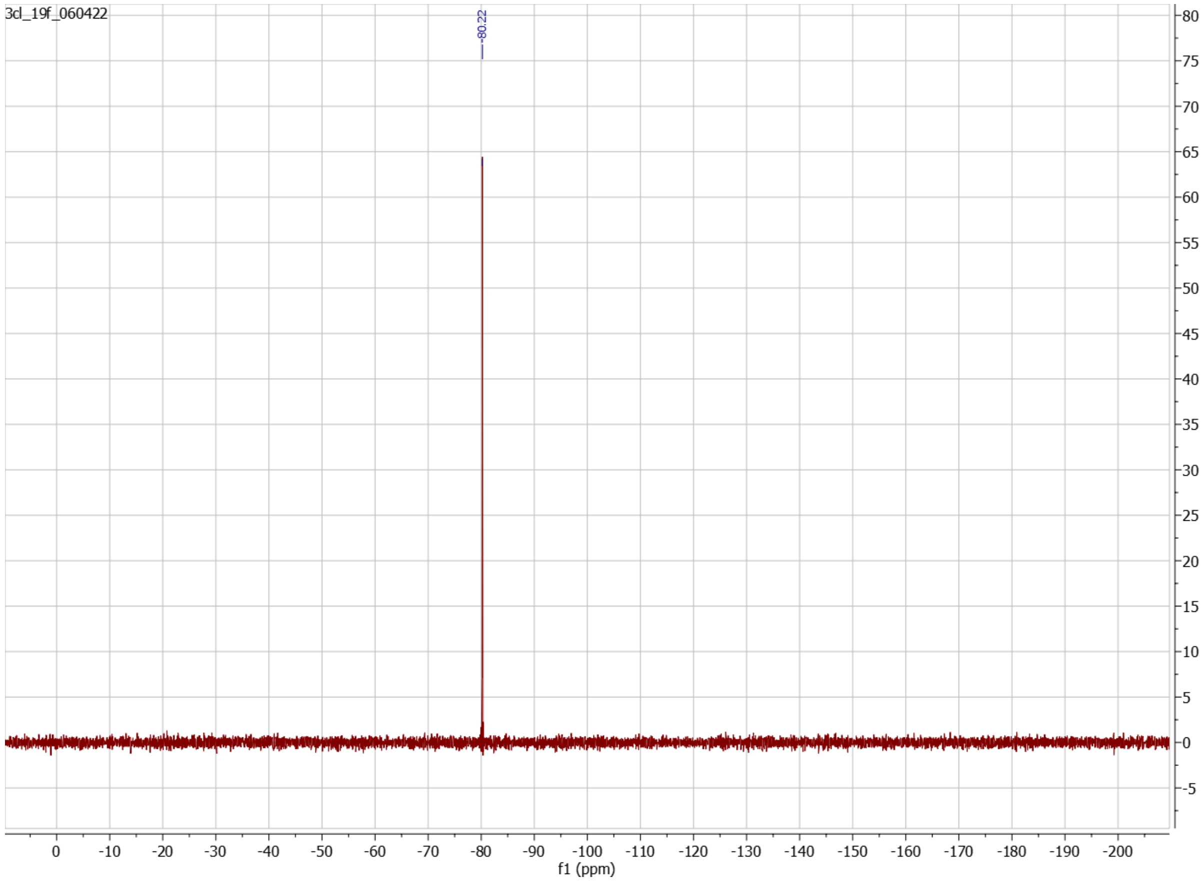

FT-IR (neat, ATR): 3183.02, 1732.46, 1557.51, 1505.81, 1376.63, 1339.53, 1242.58, 1191.79, 1099.25, 1021.38, 693.69, 620.76, 608.46, 591.14

COSY 2D NMR (60 MHz, DMSO-d_6)

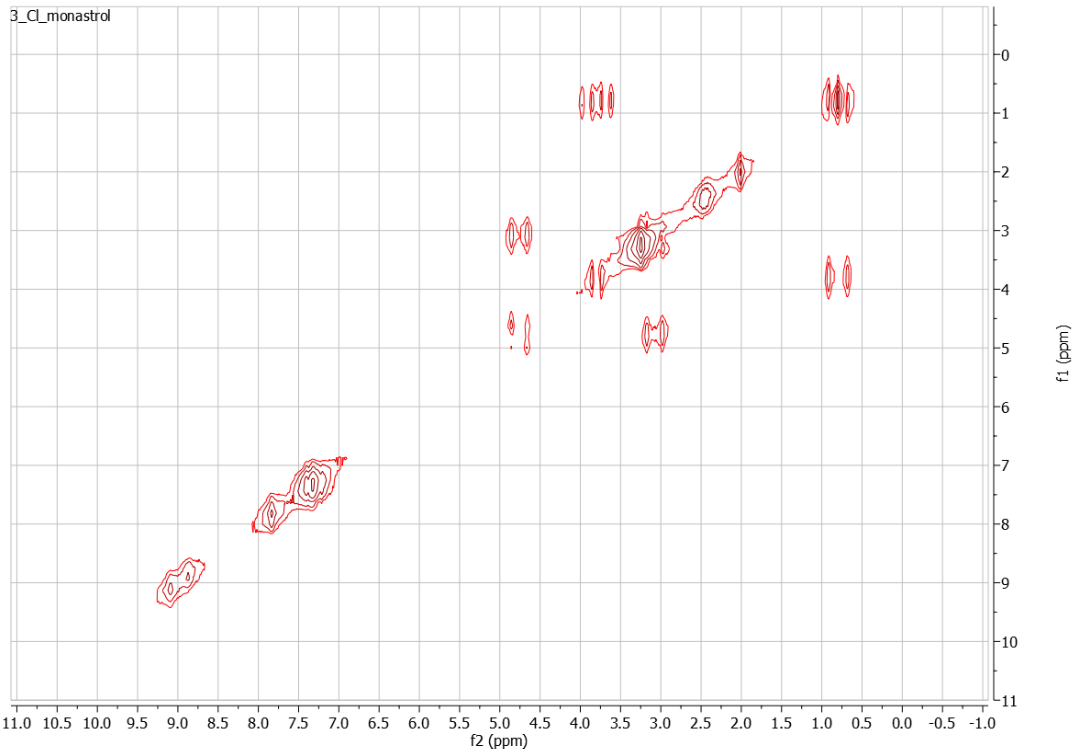

ethyl (4*S*,5*R*)-6-(3-fluorophenyl)-4-hydroxy-2-thioxo-4-(trifluoromethyl)hexahydropyrim idine-5-carboxylate (Compound 3g - 3-fluoro substitution):

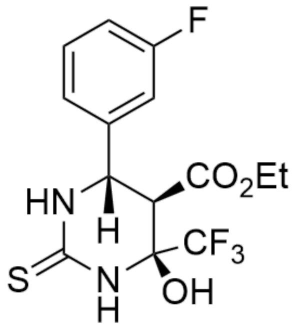

Following the general procedure yielded a white solid (288.34 mg, 78.71% qNMR isolated yield).

MW: 366.33 g/mol

Experimentalists: RC, SS

TLC R_f = 0.7125 (50% EtOAc/Hex), UV active

LCMS *m/z:* Calcd for $C_{14}H_{13}F_4N_2O_3S^-$ 365.06 $[M-H]^-$, Found 365.14 $[M-H]^-$

^{1}H NMR (60 MHz, DMSO-d_6) δ 9.14 (s, 1H), 8.94 (bs, 1H), 7.88 (bs, 1H), 7.22 (d, J = 8.7 Hz, 4H), 4.82 (d, J = 11.8 Hz, 1H), 3.83 (q, J = 7.1 Hz, 2H), 3.12 (d, J = 11.8 Hz, 1H), 0.84 (t, J = 7.1 Hz, 3H).

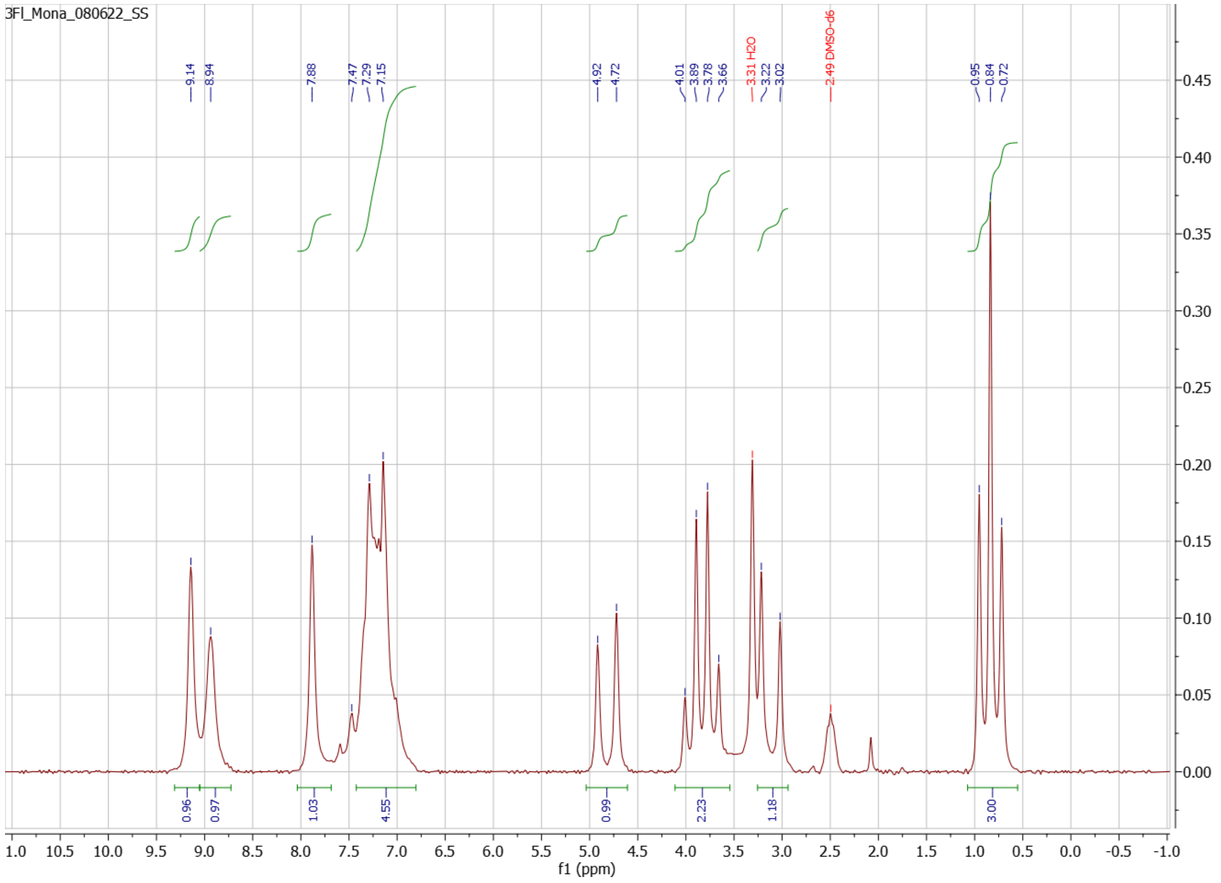

^{13}C NMR (15 MHz, DMSO-d_6) δ 177.15, 166.46, 139.56, 130.32, 124.43, 60.55, 53.89, 48.94, 13.43.

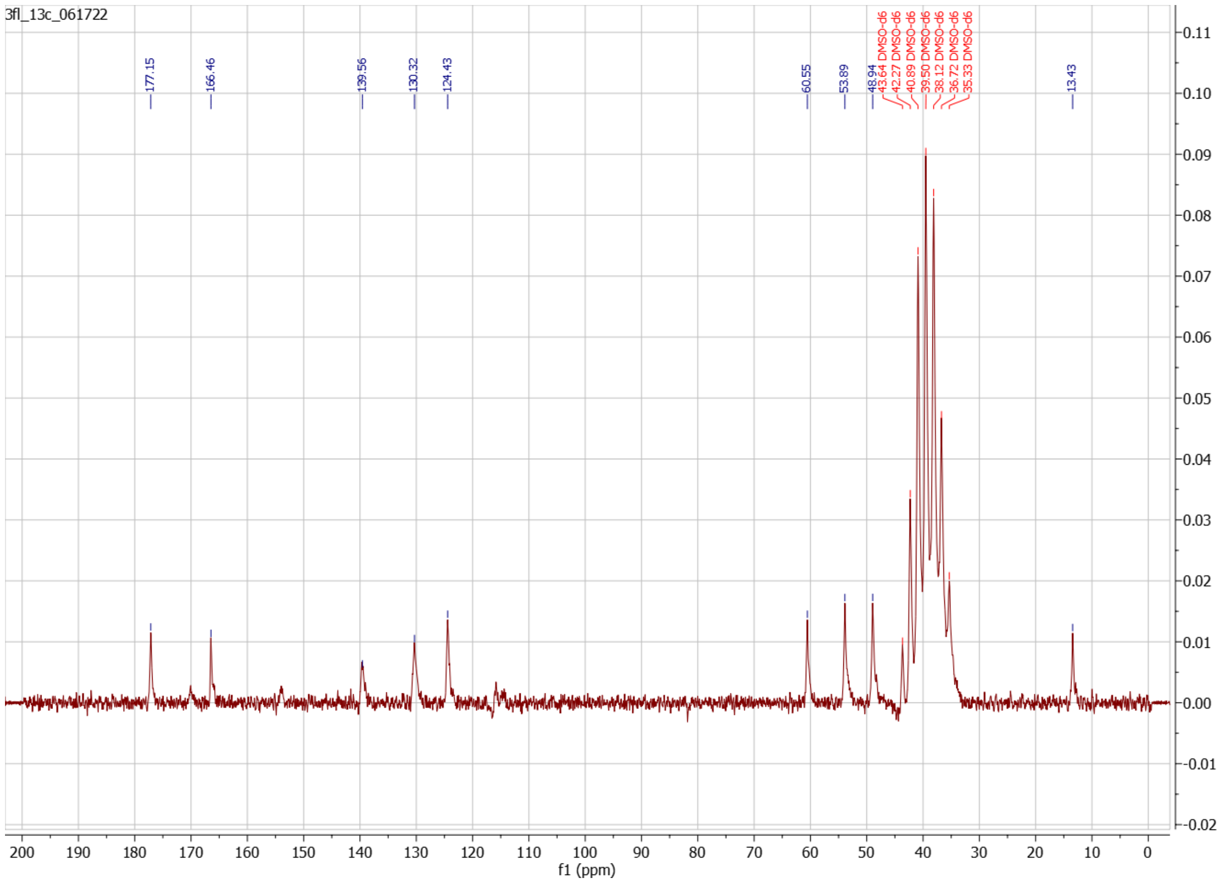

^{19}F NMR (60 MHz, DMSO-d_6) δ -80.25, -113.28 J = 5.1 Hz.

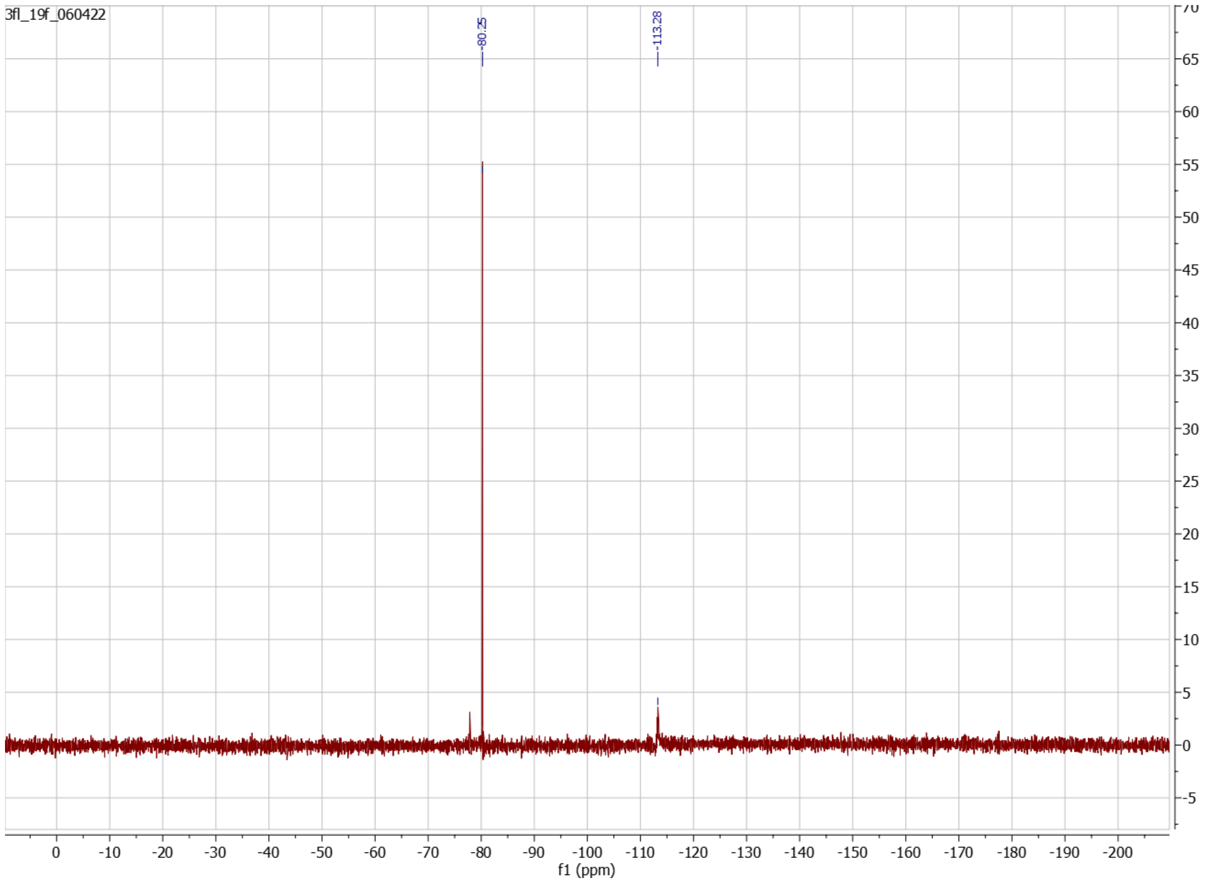

FT-IR (neat, ATR): 3402.28, 3197.52, 1734.95, 1616.82, 1593.85, 1564.22, 1488.23, 1452.92, 1398.11, 1376.86, 1346.03, 1260.09, 1196.57, 1143.14, 1095.25, 1031.45, 949.24, 793.71, 695.29, 611.18, 595, 582.68, 570.49, 564.67, 559.76

COSY 2D NMR (60 MHz, DMSO-d_6)

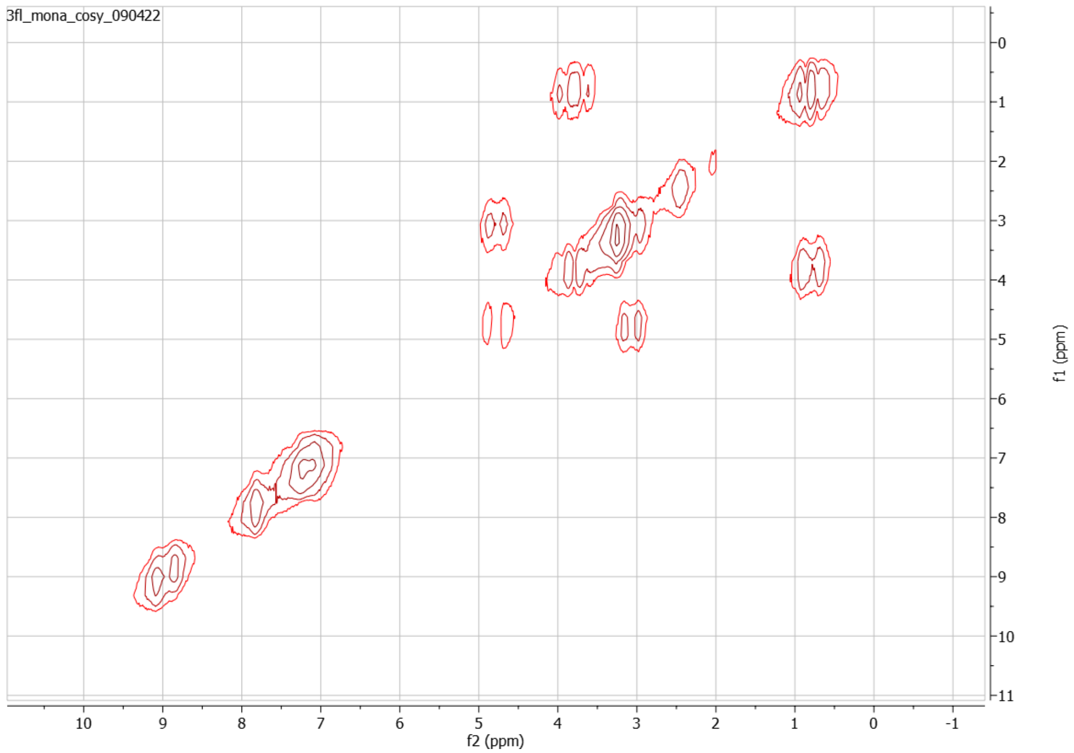

ethyl (4*S*,5*R*)-6-(3-cyanophenyl)-4-hydroxy-2-thioxo-4-(trifluoromethyl)hexahydropyrimidine-5-carboxylate (Compound 3h - 3-cyano substitution):

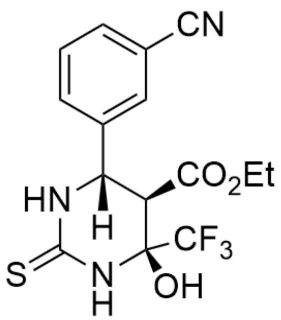

Following the general procedure yielded a white solid (145.20 mg, 38.89% qNMR isolated yield).

MW: 373.35 g/mol

Experimentalists: RC, PS

TLC R_f = 0.5875 (50% EtOAc/Hex), UV active

LCMS *m/z:* Calcd for $C_{15}H_{13}F_3N_3O_3S^-$ 372.06 [M-H]$^-$, Found 372.11 [M-H]$^-$

^{1}**H NMR** (60 MHz, DMSO-d_6) δ 9.15 (s, 1H), 9.00 (bs, 1H), 8.13 – 7.33 (m, 5H), 4.86 (d, J = 11.9 Hz, 1H), 3.82 (q, J = 7.0 Hz, 2H), 3.15 (d, J = 11.9 Hz, 1H), 0.82 (t, J = 7.0 Hz, 3H).

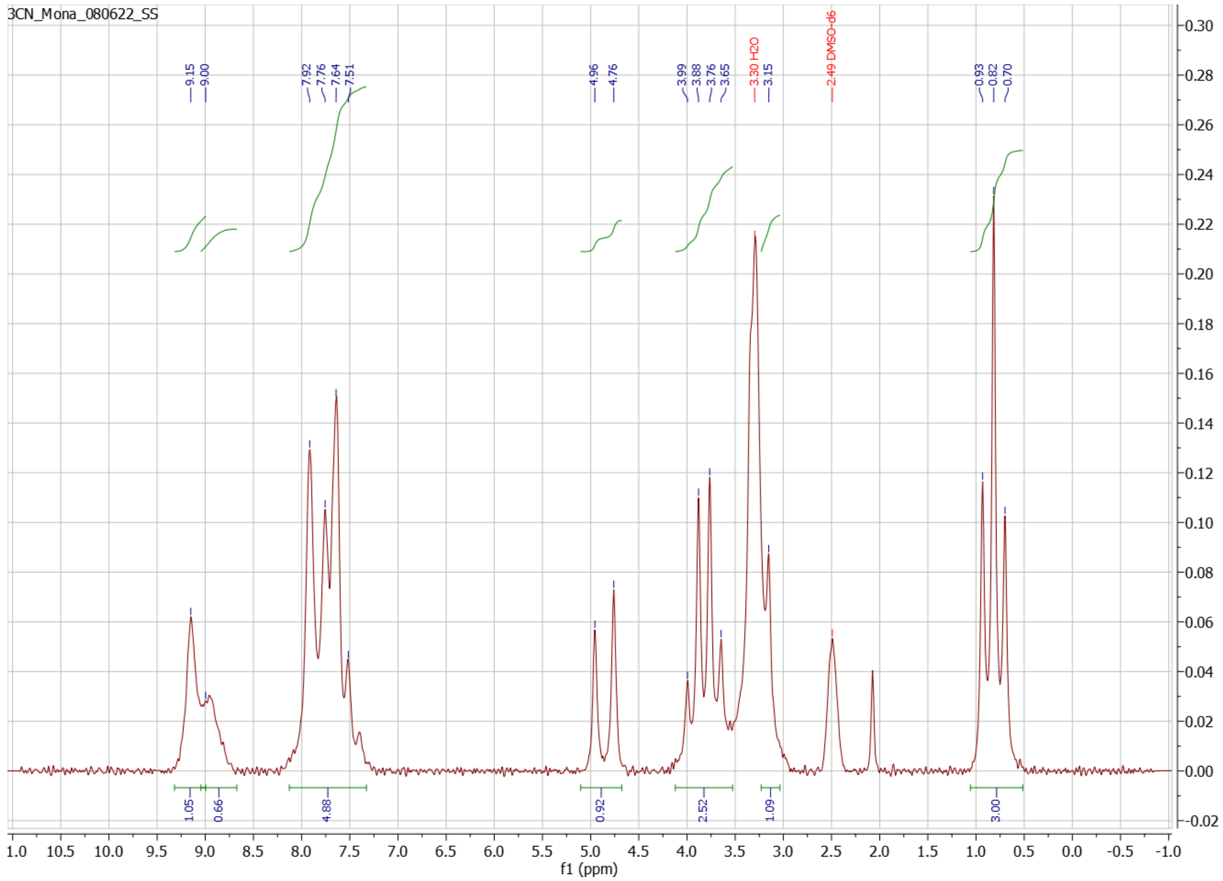

^{13}C NMR (15 MHz, DMSO-d_6) δ 177.22, 166.49, 139.55, 131.61, 130.96, 130.60, 127.49, 121.49, 60.66, 53.84, 48.95, 13.47.

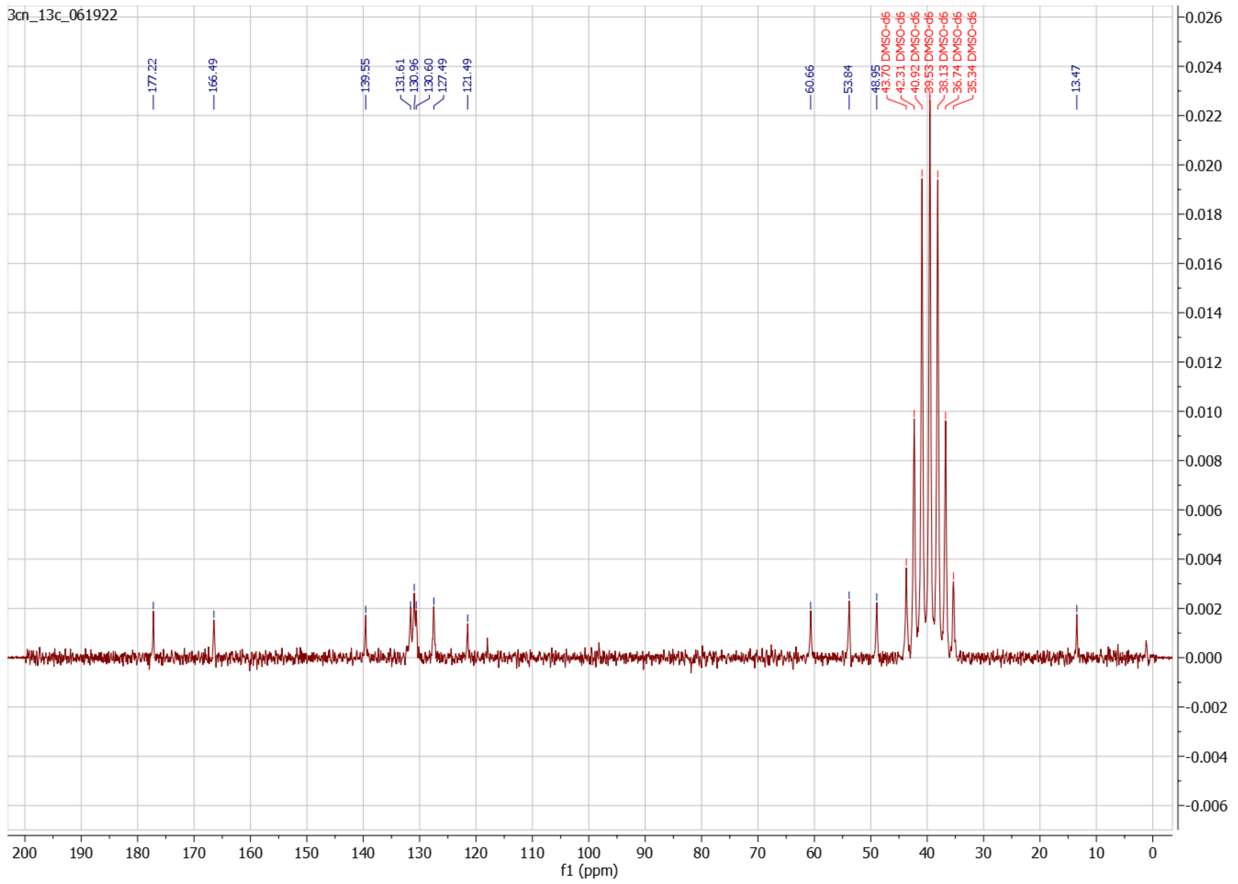

^{19}F NMR (60 MHz, DMSO-d_6) δ -80.11.

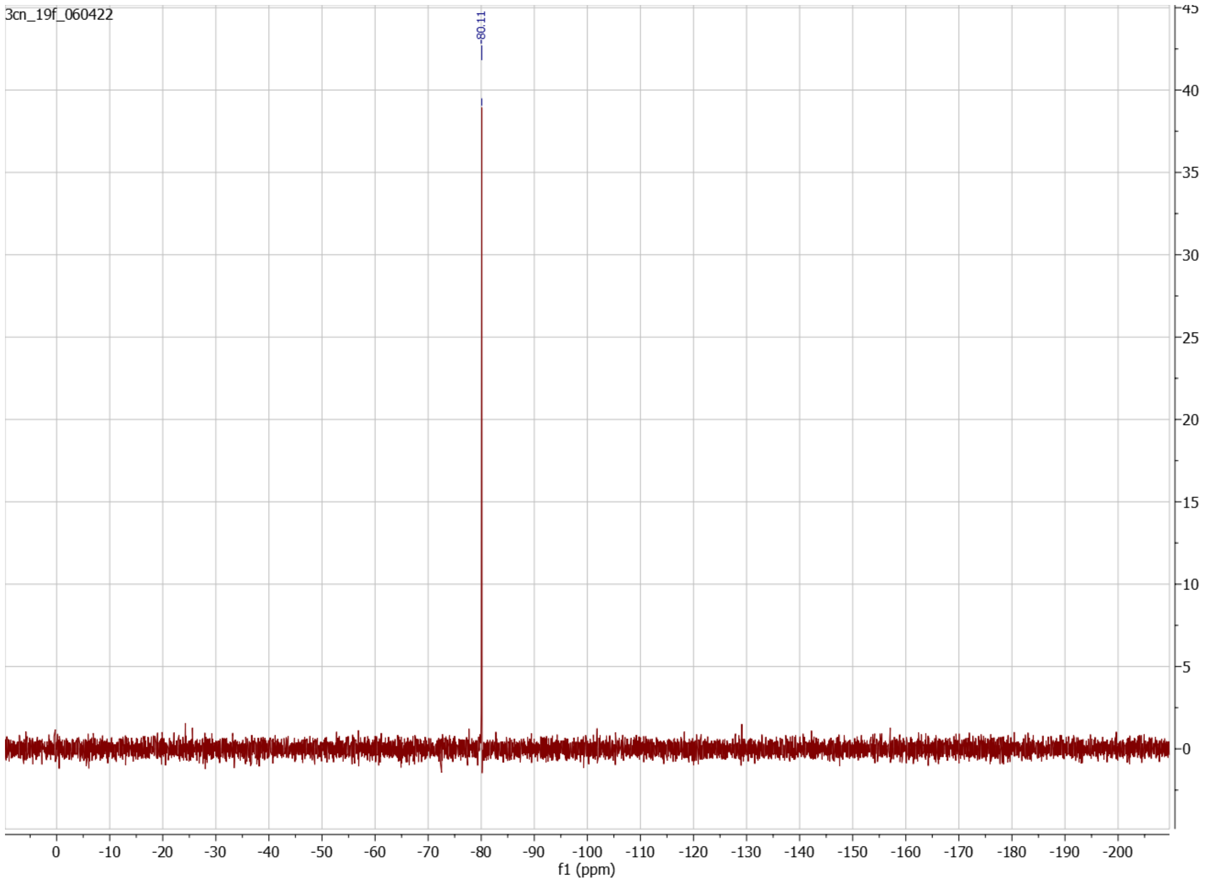

FT-IR (neat, ATR): 3238.15, 2982.03, 2356.83, 2233.63, 1731.57, 1563.2, 1483.83, 1376.15, 1345.64, 1248.87, 1200.3, 1093.45, 1032.78, 806.87, 693.41, 604.66, 584.3

COSY 2D NMR (60 MHz, DMSO-d_6)

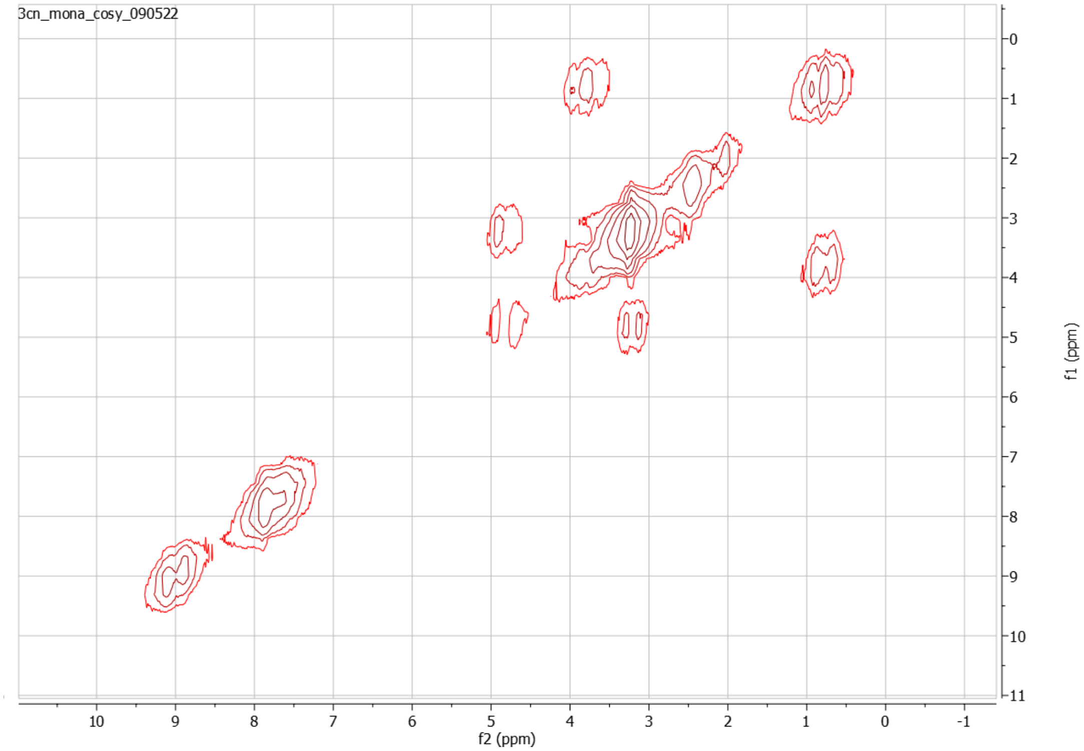

ethyl (4*S*,5*R*)-4-hydroxy-6-(3-nitrophenyl)-2-thioxo-4-(trifluoromethyl)hexahydropyrimidine-5-carboxylate (Compound 3i - 3-nitro substitution):

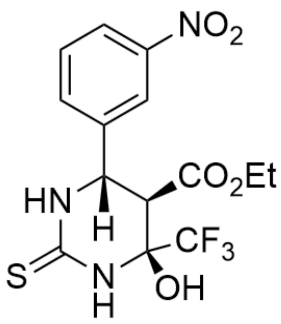

Following the general procedure yielded a white solid (155.52 mg, 39.54% qNMR isolated yield).

MW: 393.34 g/mol

Experimentalists: RC, SS

TLC R_f = 0.625 (50% EtOAc/Hex), UV active

LCMS *m/z:* Calcd for $C_{14}H_{13}F_3N_3O_5S^-$ 392.05 [M-H]$^-$, Found 392.02 [M-H]$^-$

^{1}H NMR (60 MHz, DMSO-d_6) δ 9.23 (s, 1H), 9.02 (s, 1H), 8.46 – 7.41 (m, 5H), 4.97 (d, J = 11.8 Hz, 1H), 3.82 (q, J = 7.0 Hz, 2H), 3.26 (d, J = 11.7, 1H), 0.82 (t, J = 7.0 Hz, 3H).

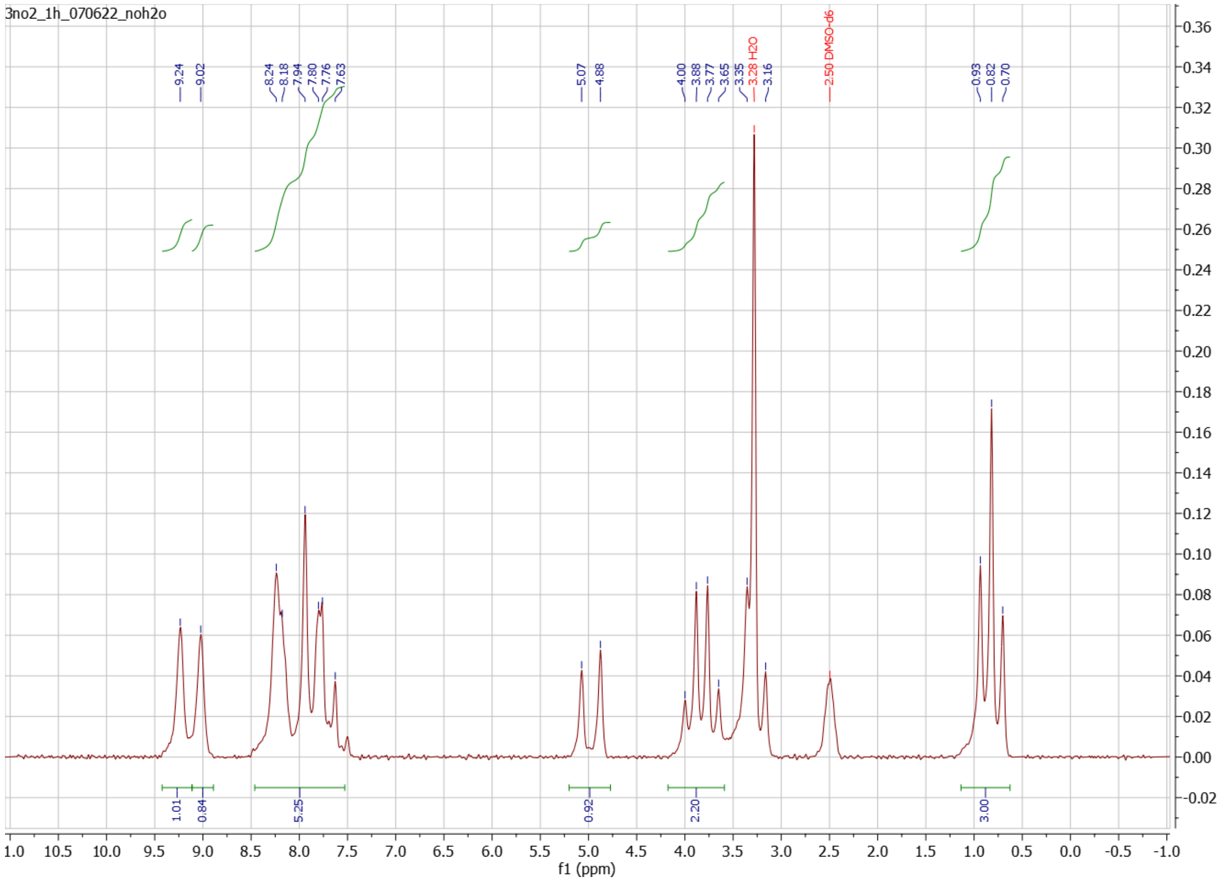

^{13}C NMR (15 MHz, DMSO-d_6) δ 177.33, 166.44, 147.62, 139.15, 135.21, 130.05, 123.26, 60.67, 53.70, 48.69, 13.38.

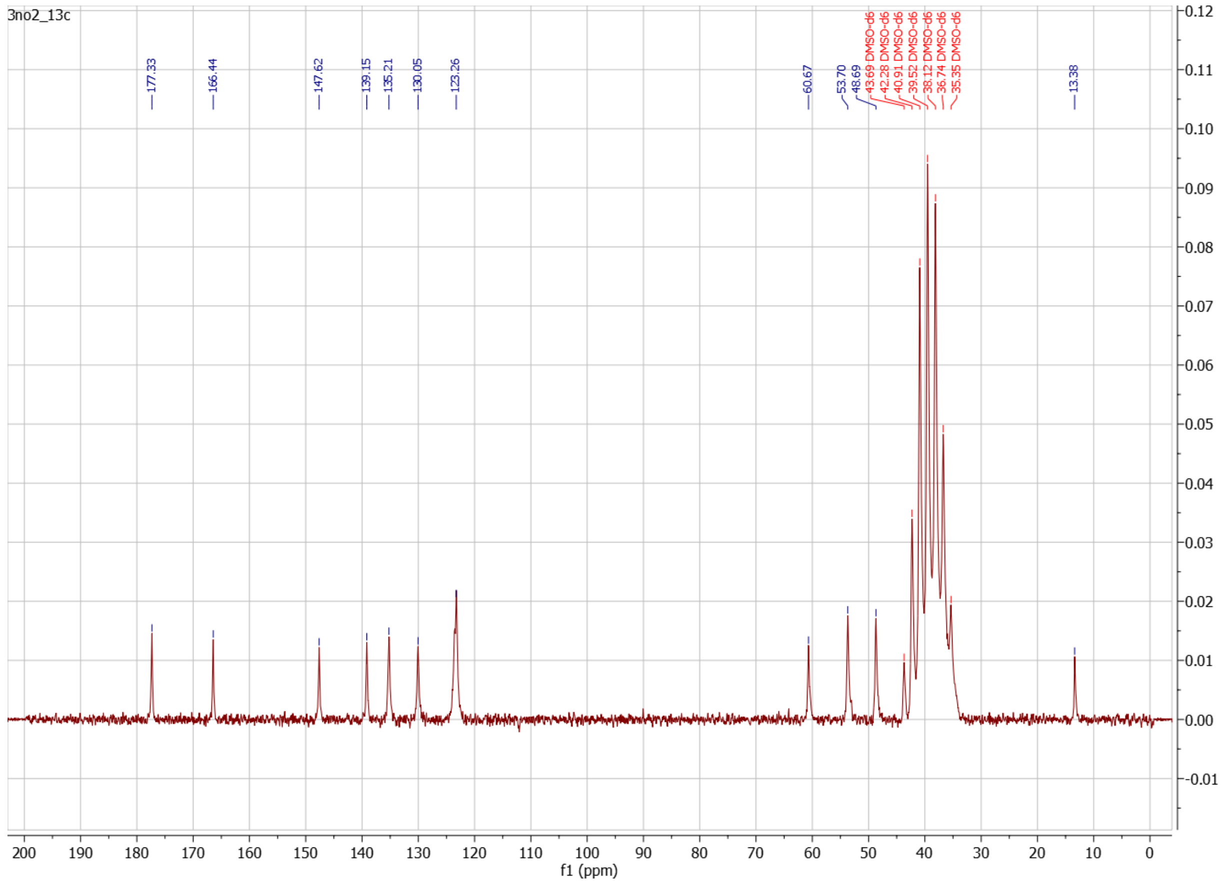

^{19}F NMR (60 MHz, DMSO-d_6) δ -80.09.

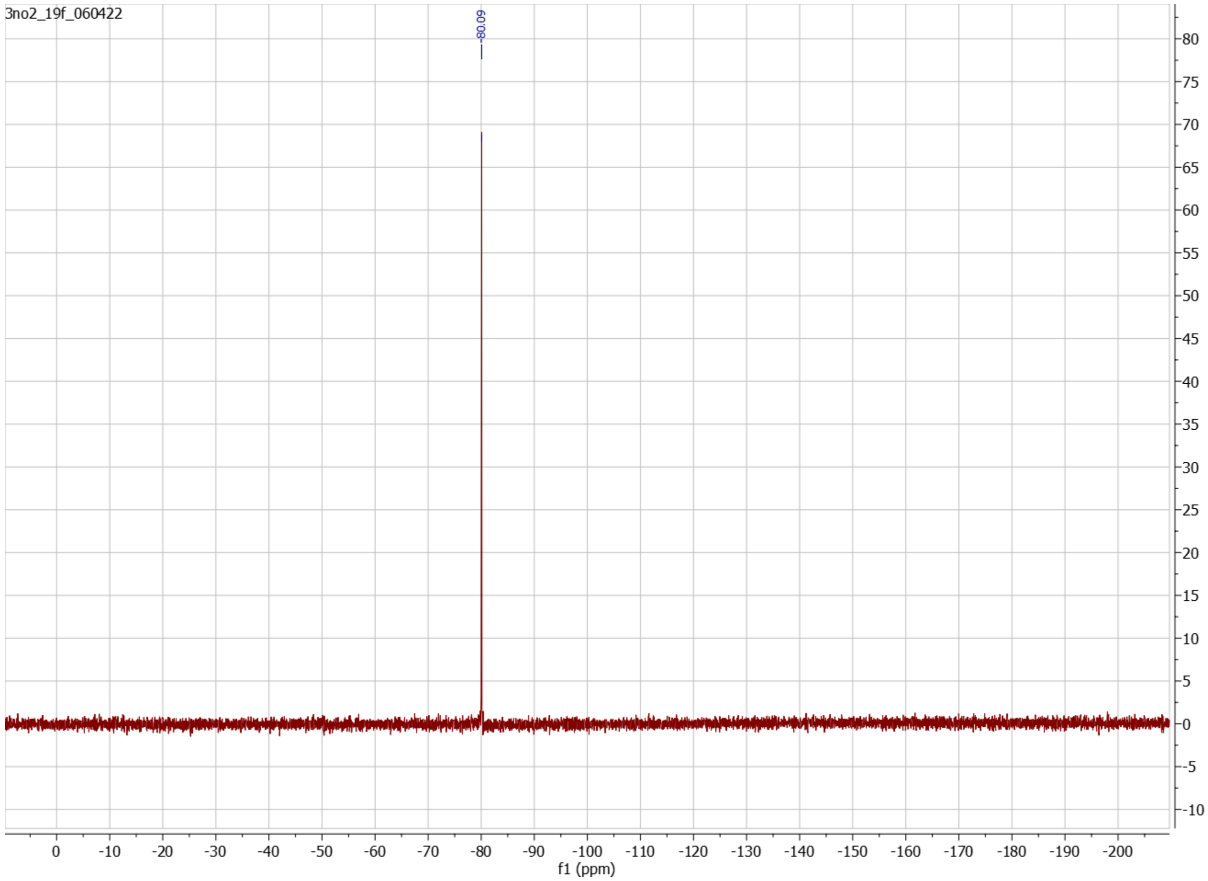

FT-IR (neat, ATR): 3370.64, 3209.35, 2987.11, 1732.08, 1567.65, 1537.09, 1518.2, 1381.14, 1353.56, 1264.03, 1206.96, 1141.05, 1099.55, 1083.06, 1034.1, 815.74, 731.48, 689.43, 620.93

COSY 2D NMR (60 MHz, DMSO-d_6)

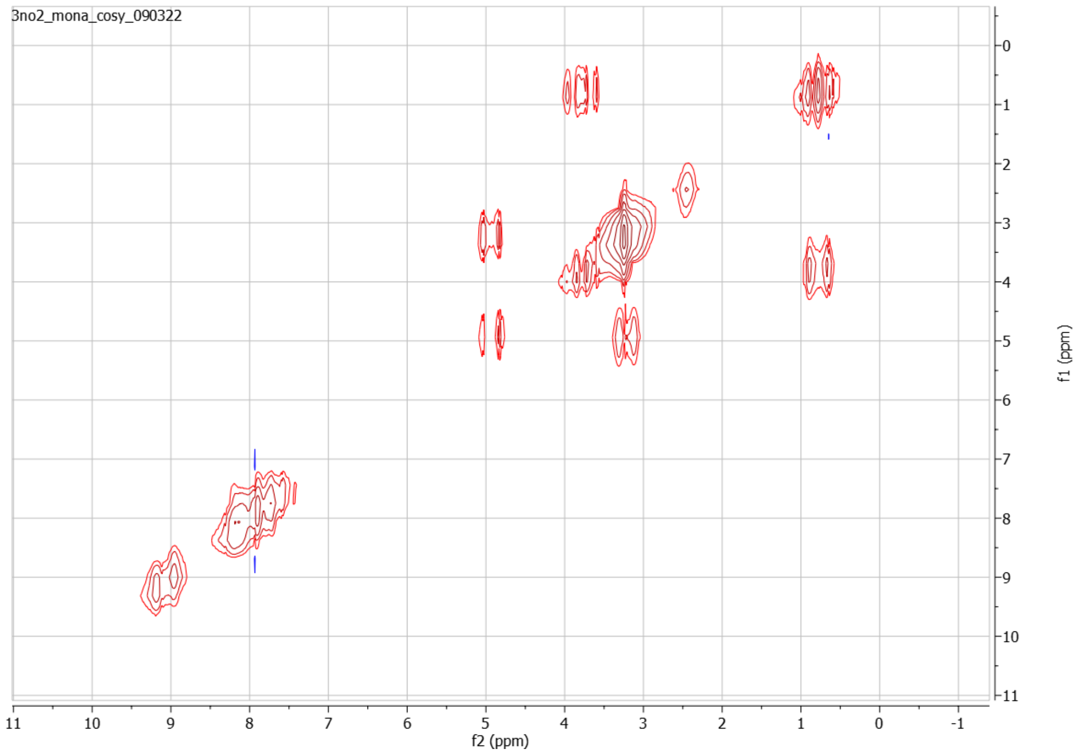

ethyl(4*S*,5*R*)-4-hydroxy-6-(4-(pyrrolidin-1-yl)phenyl)-2-thioxo-4-(trifluoromethyl)hexahydropyrimidine-5-carboxylate (Compound 3j - 4-pyrrolidine substitution):

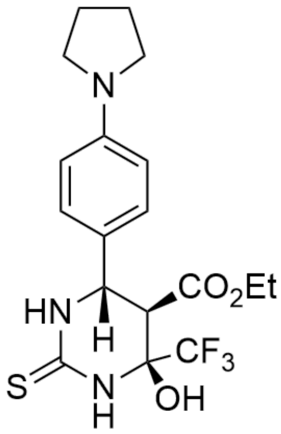

Following the general procedure yielded an orange solid (240.66 mg, 57.65% qNMR isolated yield).

MW: 417.45 g/mol

Experimentalists: RC, AF

TLC R_f = 0.675 (50% EtOAc/Hex), UV active

LCMS *m/z:* Calcd for $C_{18}H_{23}F_3N_3O_3S^+$ 418.14 $[M+H]^+$, Found 418.21 $[M+H]^+$

^{1}H NMR (60 MHz, DMSO-*d*$_6$) δ 8.81 (s, 1H), 8.74 (s, 1H), 7.76 (s, 1H), 7.07 (d, J = 8.3 Hz, 2H), 6.46 (d, J = 8.6 Hz, 2H), 4.66 (d, J = 11.7 Hz, 1H), 3.81 (q, J = 7.2 Hz, 2H), 3.19 (s, 4H), 2.95 (d, J = 12.3 Hz, 1H), 1.93 (s, 4H), 0.88 (t, J = 7.0 Hz, 3H).

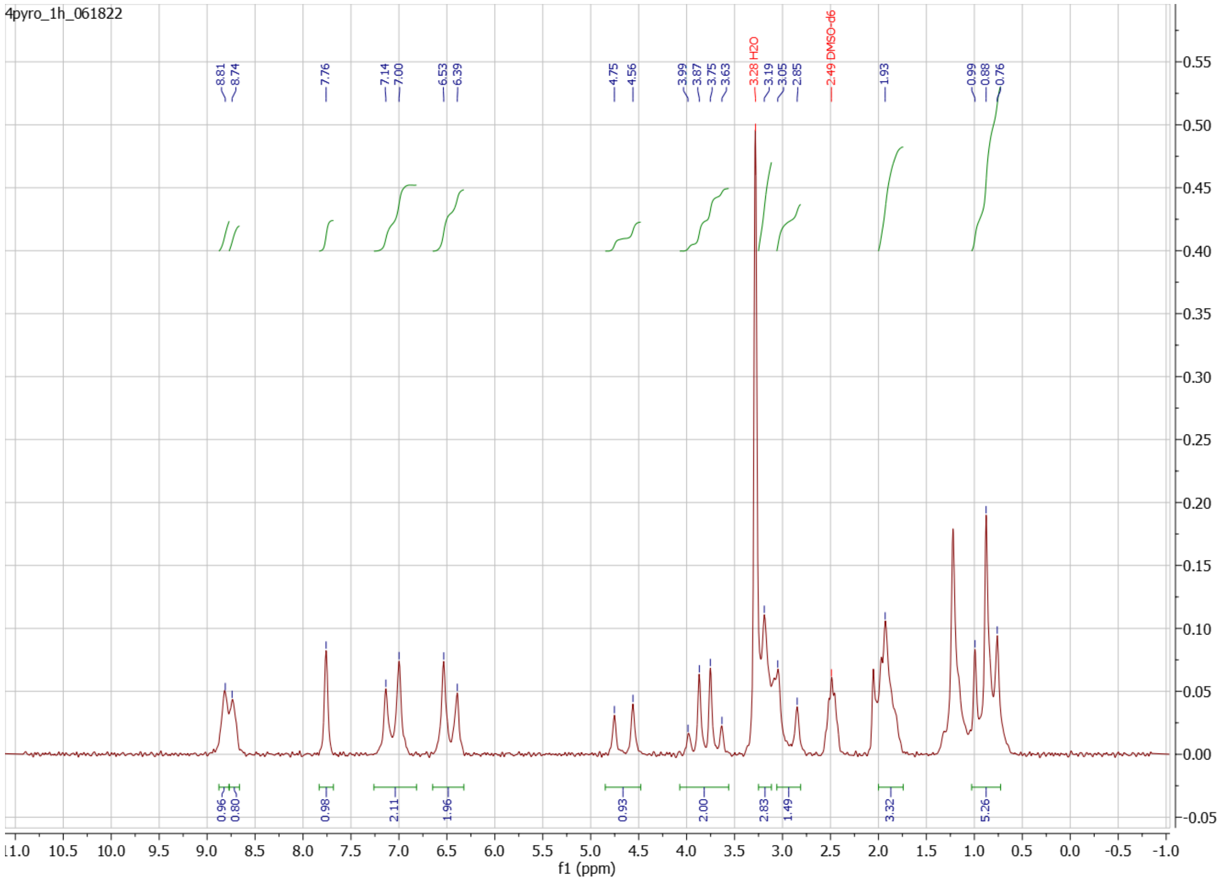

^{13}C NMR (15 MHz, DMSO-d_6) δ 176.85, 166.55, 147.71, 128.63, 122.65, 111.20, 60.29, 53.73, 49.35, 47.23, 24.84, 13.46.

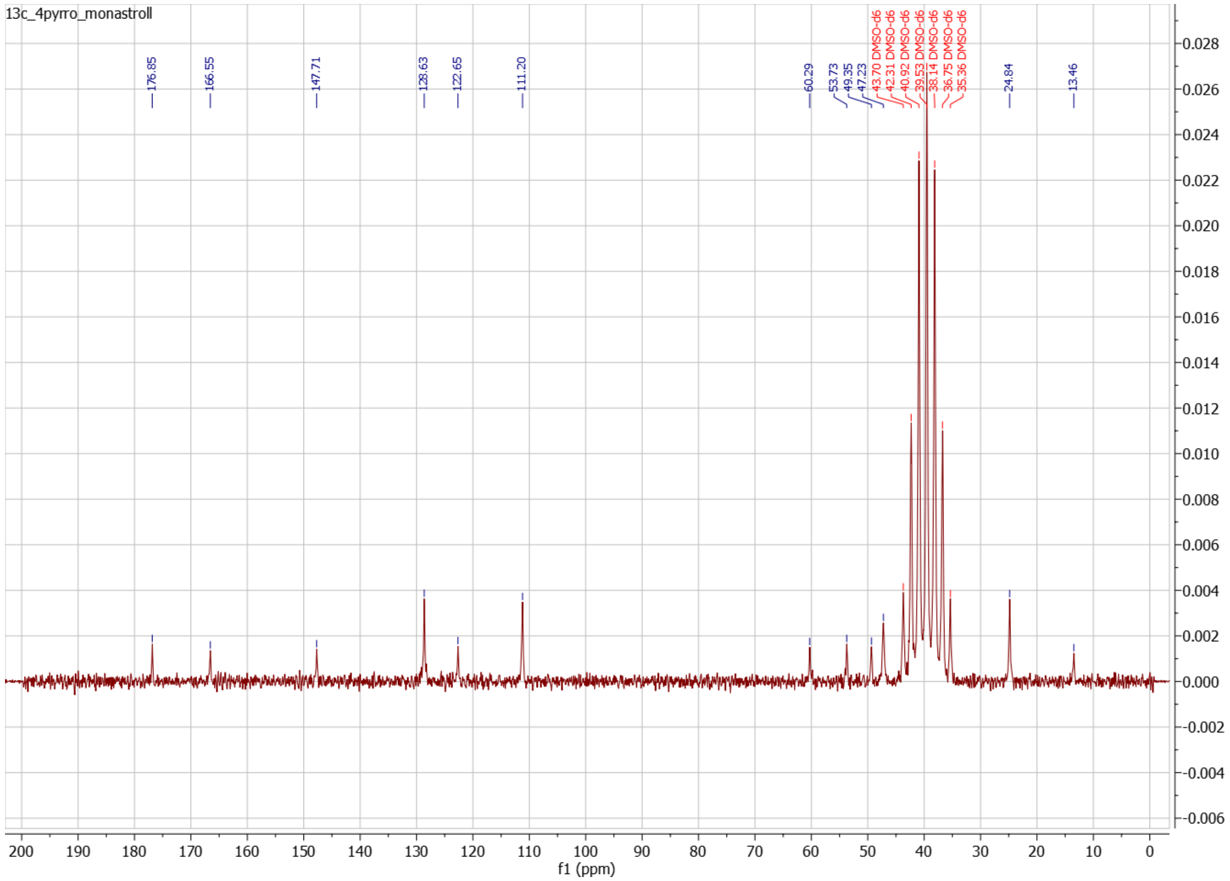

^{19}F NMR (60 MHz, DMSO-d_6) δ -80.46.

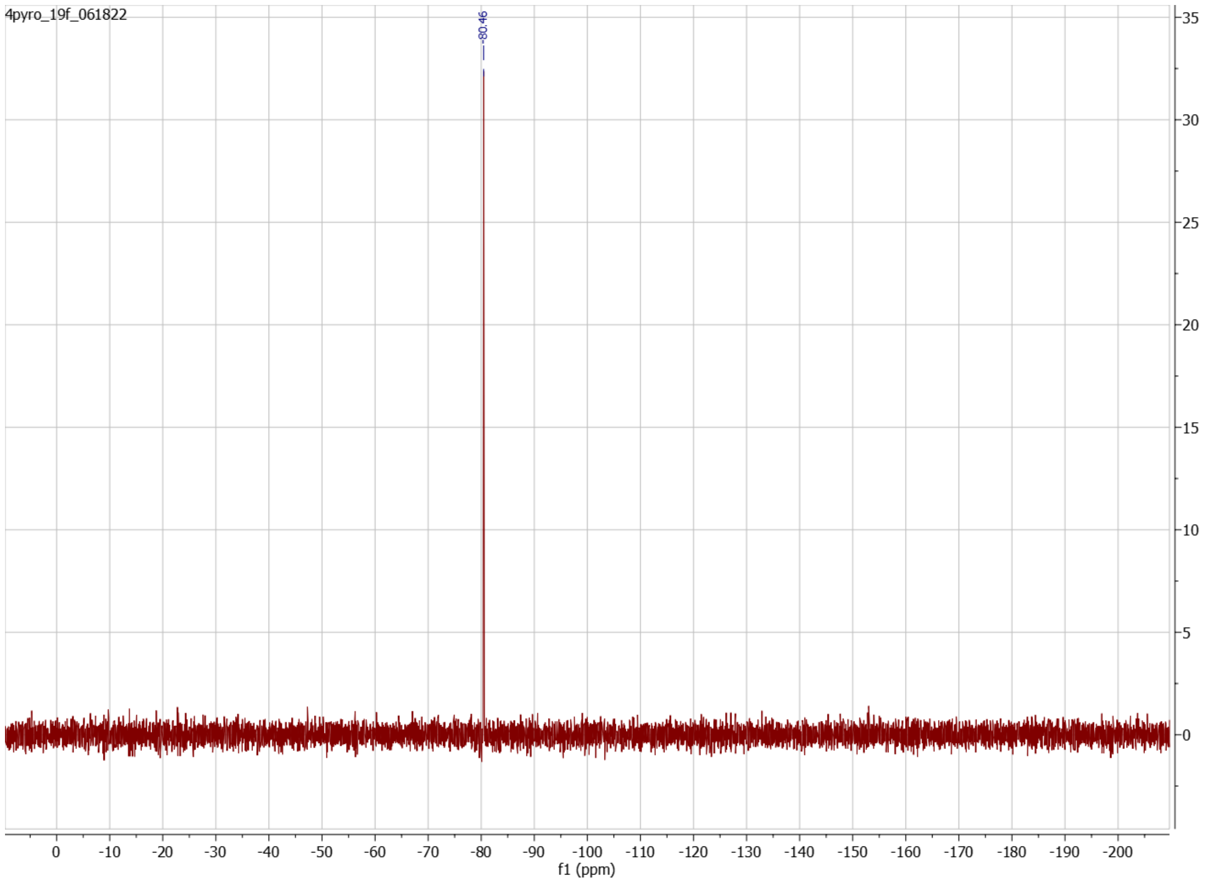

FT-IR (neat, ATR): 3182.39, 2968.47, 2159.1, 1735.62, 1613.75, 1524.04, 1486.26, 1377.38, 1343.28, 1241.75, 1184.28, 1023.46, 811.28, 627.8, 616.68, 606.24, 583.12, 562.55

COSY 2D NMR (60 MHz, DMSO-d_6)

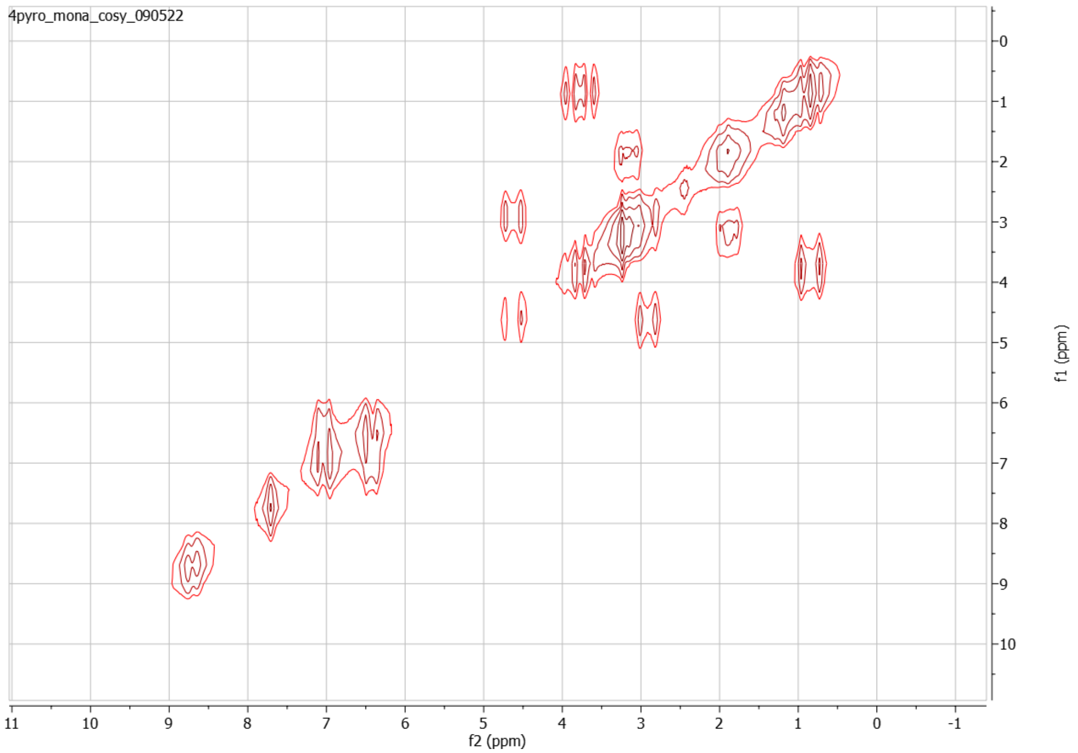

ethyl (4*S*,5*R*)-6-(4-(dimethylamino)phenyl)-4-hydroxy-2-thioxo-4-(trifluoromethyl)hexahydropyrimidine-5-carboxylate (Compound 3k - 4-dimethylamino substitution):

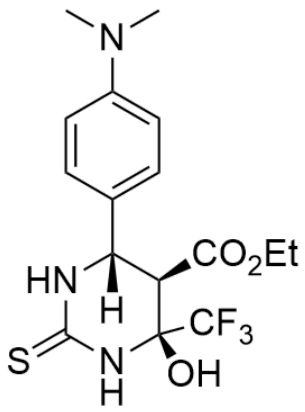

Following the general procedure yielded a light yellow solid (250.81 mg, 64.08% qNMR isolated yield).

MW: 391.41 g/mol

Experimentalists: RC

TLC R_f = 0.625 (50% EtOAc/Hex), UV active

LCMS *m/z:* Calcd for $C_{16}H_{21}F_3N_3O_3S^+$ 392.13 $[M+H]^+$, Found 392.23 $[M+H]^+$

^{1}**H NMR** (60 MHz, DMSO-d_6) δ 8.87 (s, 1H), 8.78 (s, 1H), 7.77 (s, 1H), 7.10 (d, J = 8.7 Hz, 2H), 6.65 (d, J = 8.7 Hz, 2H), 4.67 (d, J = 11.7 Hz, 1H), 3.81 (q, J = 7.0 Hz, 2H), 2.96 (d, J = 11.7 Hz, 1H), 2.87 (s, 6H), 0.85 (t, J = 7.0 Hz, 3H).

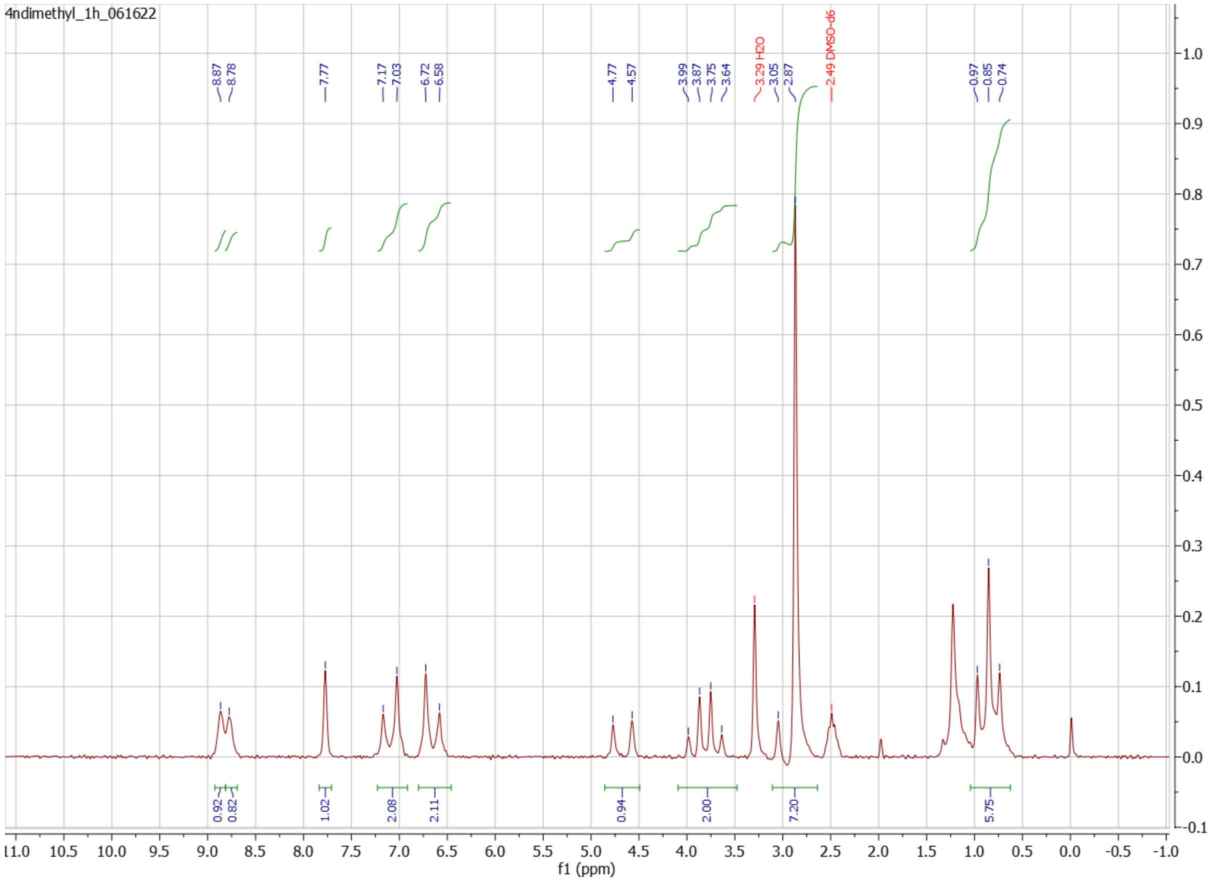

^{13}C NMR (15 MHz, DMSO-d_6) δ 177.23, 166.27, 147.61, 144.40, 129.88, 123.37, 60.78, 53.76, 48.87, 13.43.

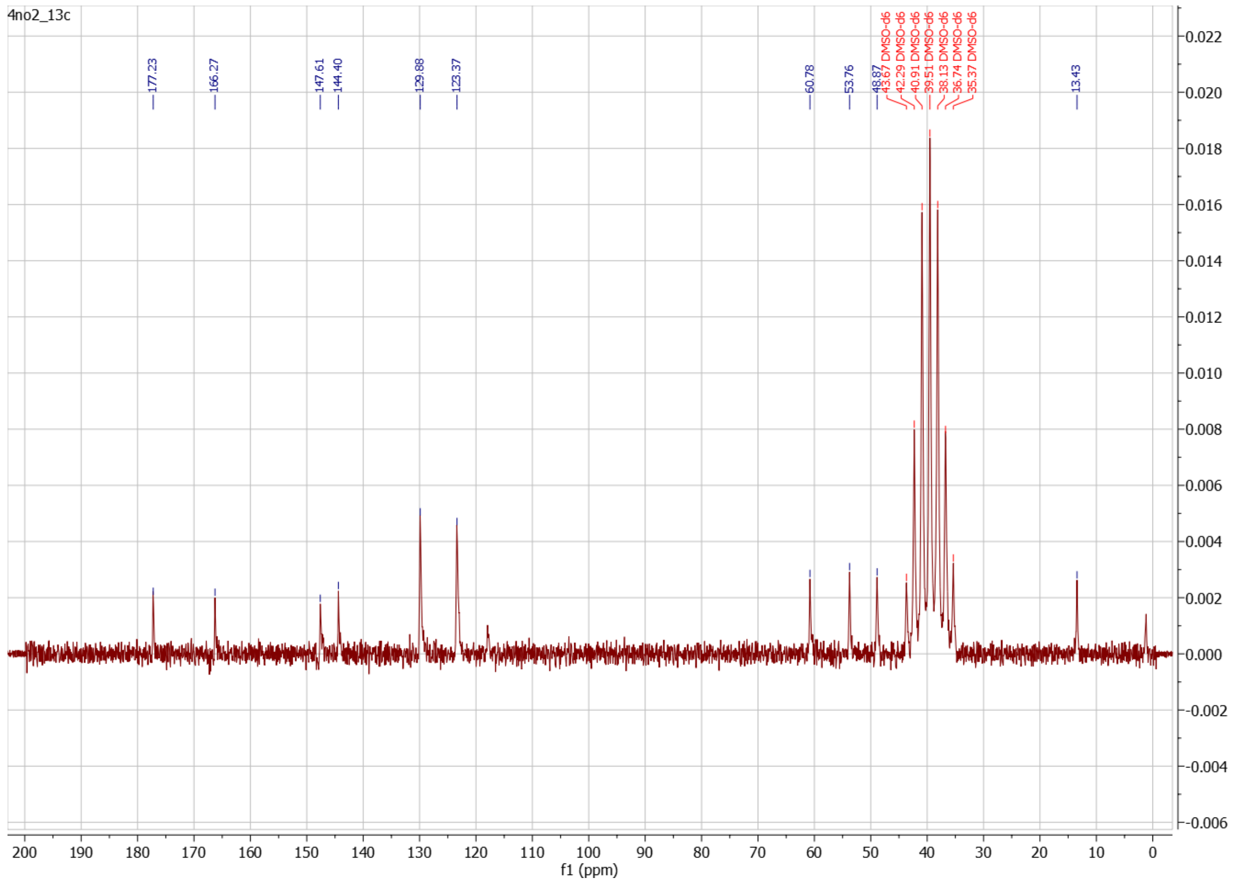

^{19}**F NMR** (60 MHz, DMSO-d_6) δ -80.46.

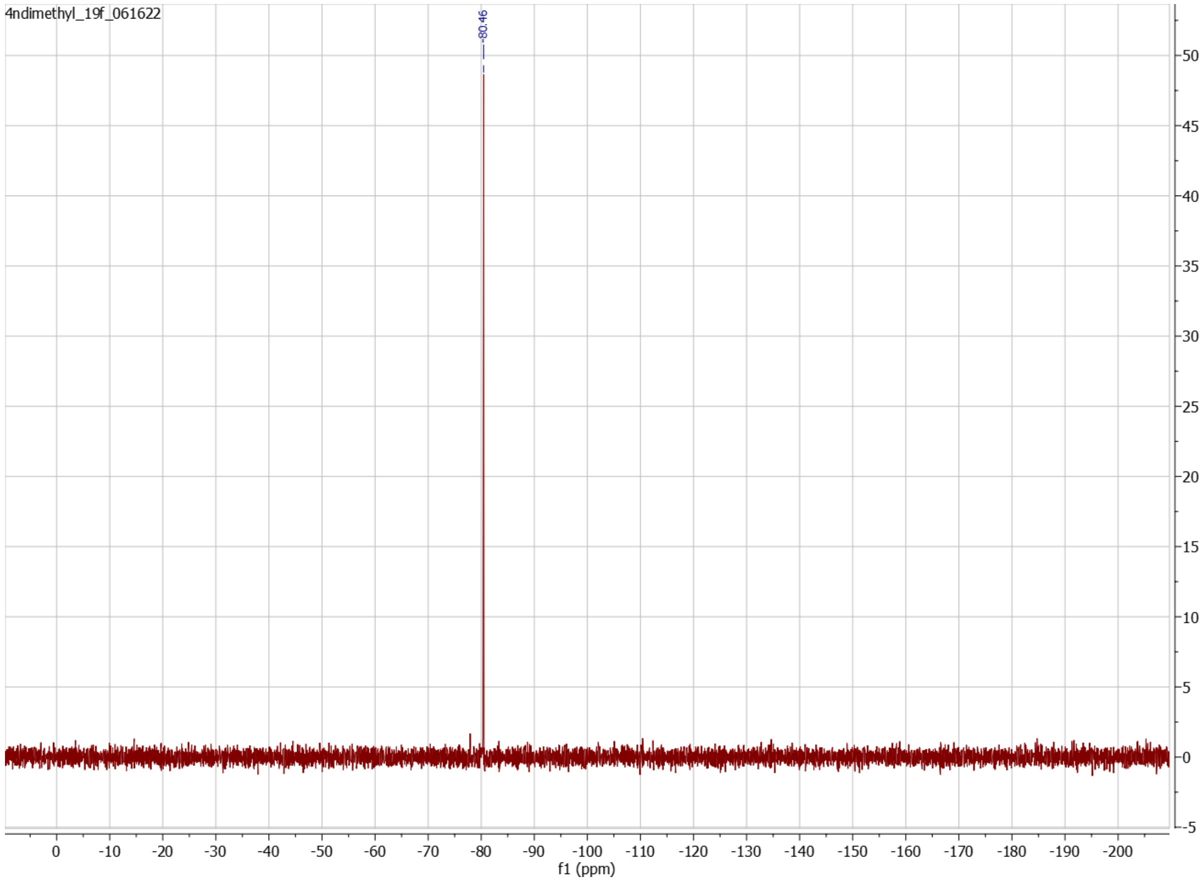

FT-IR (neat, ATR): 3182.39, 2982.91, 2357.18, 1737.16, 1613.75, 1548.25, 1524.78, 1343.7, 1189, 1025.2, 814.39, 701.51, 636.18, 608.21, 584.44, 570.38

COSY 2D NMR (60 MHz, DMSO-d_6)

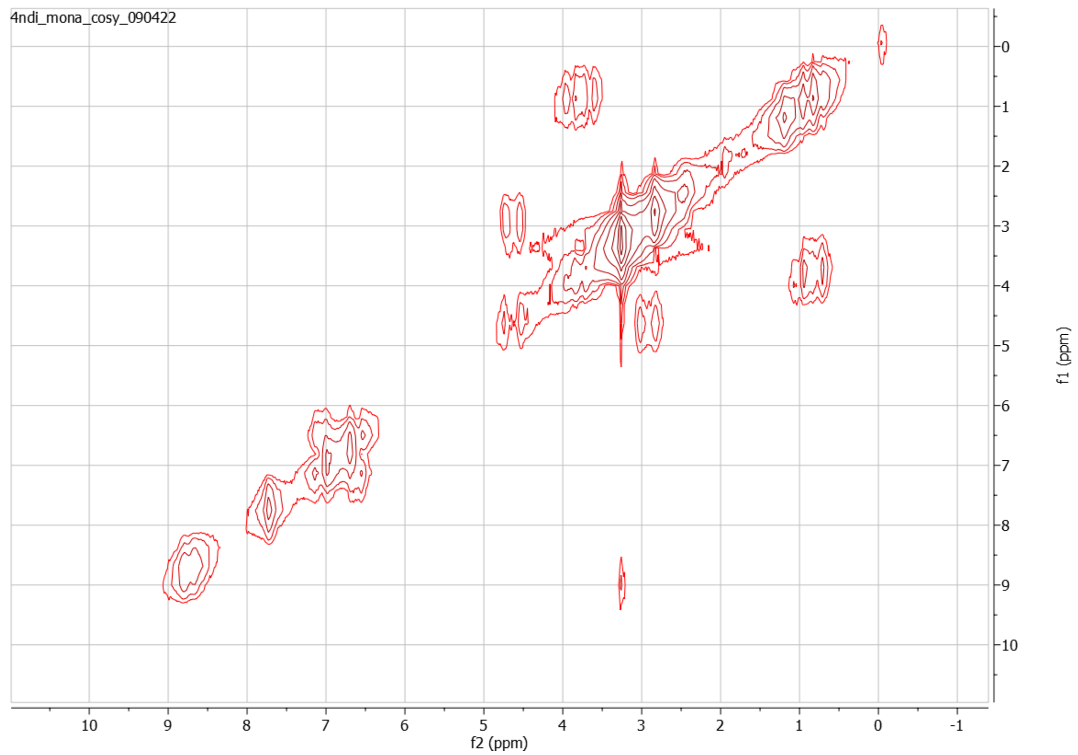

ethyl (4*S*,5*R*)-4-hydroxy-6-(4-methoxyphenyl)-2-thioxo-4-(trifluoromethyl)hexahydropyrimidine-5-carboxylate (Compound 3l - 4-methoxy substitution):

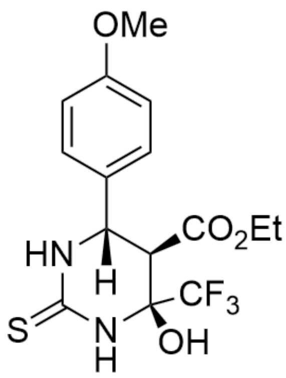

Following the general procedure yielded a white solid (305.17 mg, 80.93% qNMR isolated yield).

MW: 378.37 g/mol

Experimentalists: RC

TLC R_f = 0.65 (50% EtOAc/Hex), UV active

LCMS *m/z:* Calcd for $C_{15}H_{16}F_3N_2O_4S^-$ 377.08 [M-H]$^-$, Found 377.08 [M-H]$^-$

^{1}**H NMR** (60 MHz, DMSO-d_6) δ 8.99 (s, 1H), 8.84 (s, 1H), 7.82 (s, 1H), 7.24 (d, J = 8.8 Hz, 2H), 6.88 (d, J = 8.7 Hz, 2H), 4.73 (d, J = 11.9 Hz, 1H), 3.80 (q, J = 7.2 Hz, 2H), 3.73 (s, 3H), 2.99 (d, J = 11.9 Hz, 1H), 0.84 (t, J = 7.1 Hz, 3H).

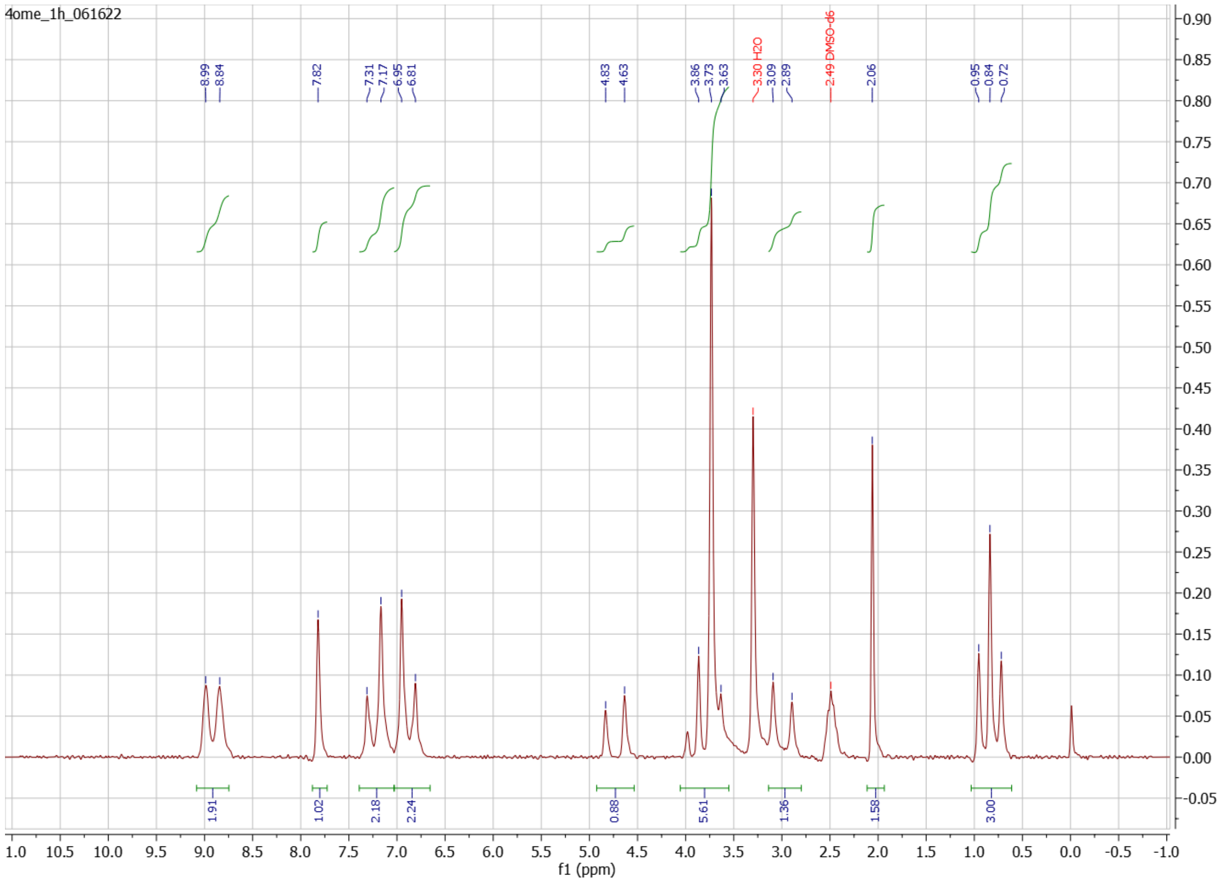

^{13}C NMR (60 MHz, DMSO-d_6) δ 176.93, 166.43, 159.28, 129.25, 128.59, 113.64, 60.32, 55.15, 53.58, 49.27, 13.42.

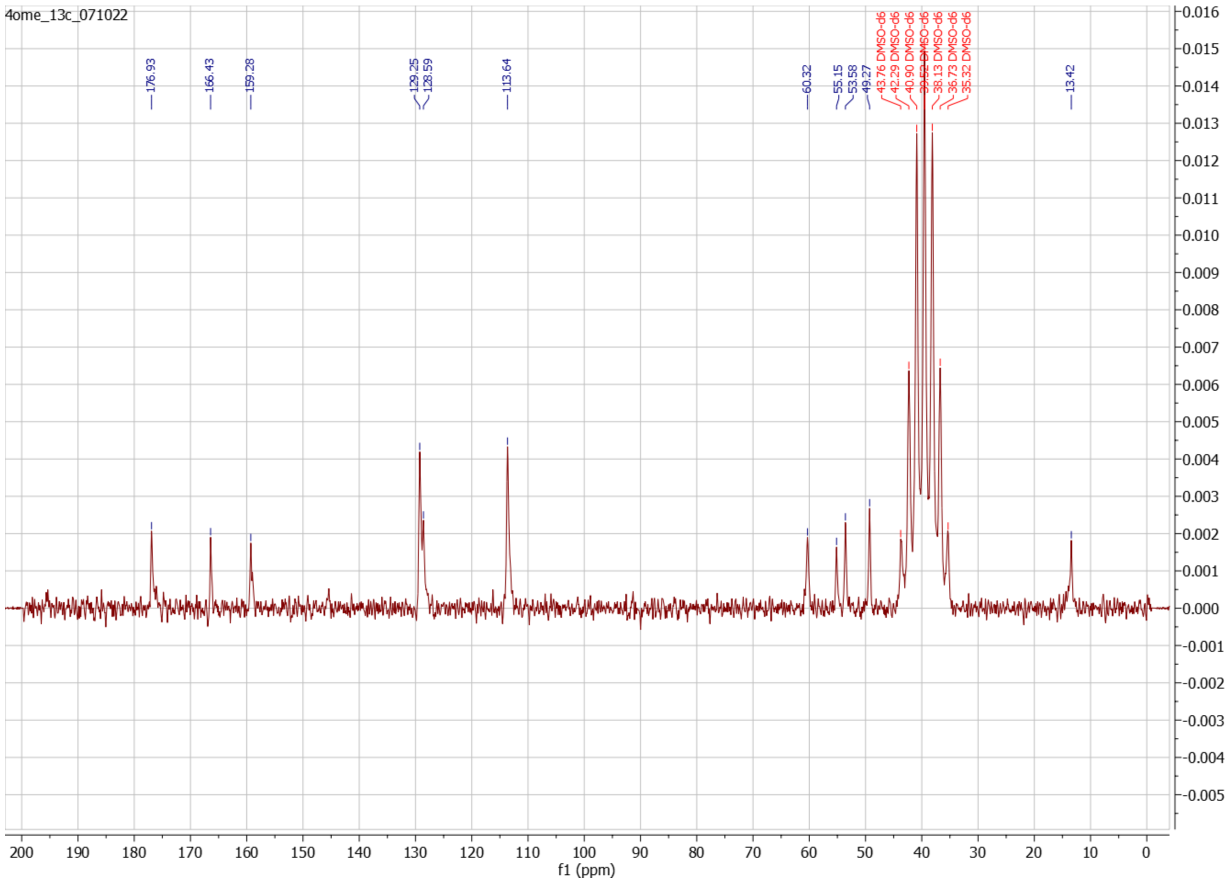

^{19}F NMR (60 MHz, DMSO-d_6) δ -80.41.

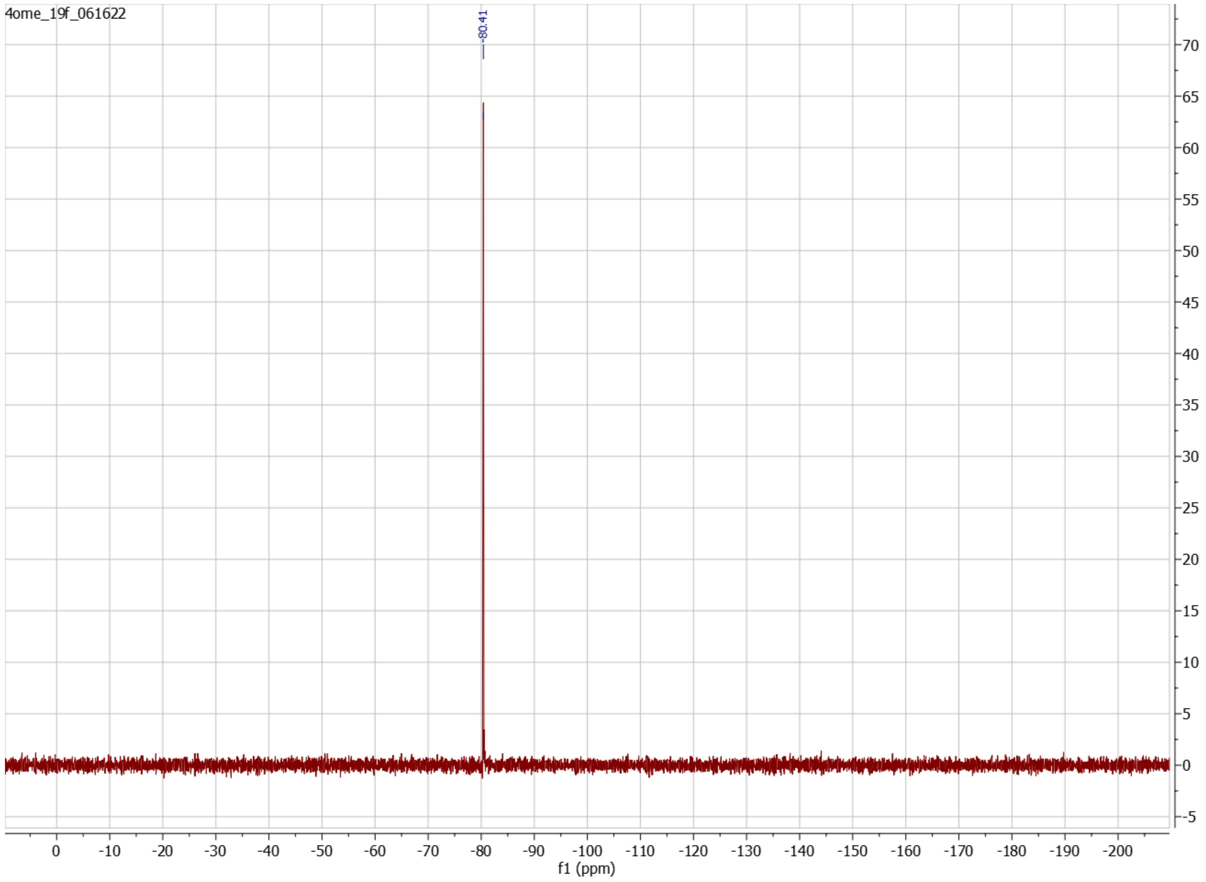

FT-IR (neat, ATR): 3178.52, 2979.06, 1726.99, 1611.97, 1552.61, 1514.06, 1377.01, 1343.64, 1306.8, 1252.1, 1191.55, 1027.46, 829.25, 623.45, 565.43, 556

COSY 2D NMR (60 MHz, DMSO-d_6)

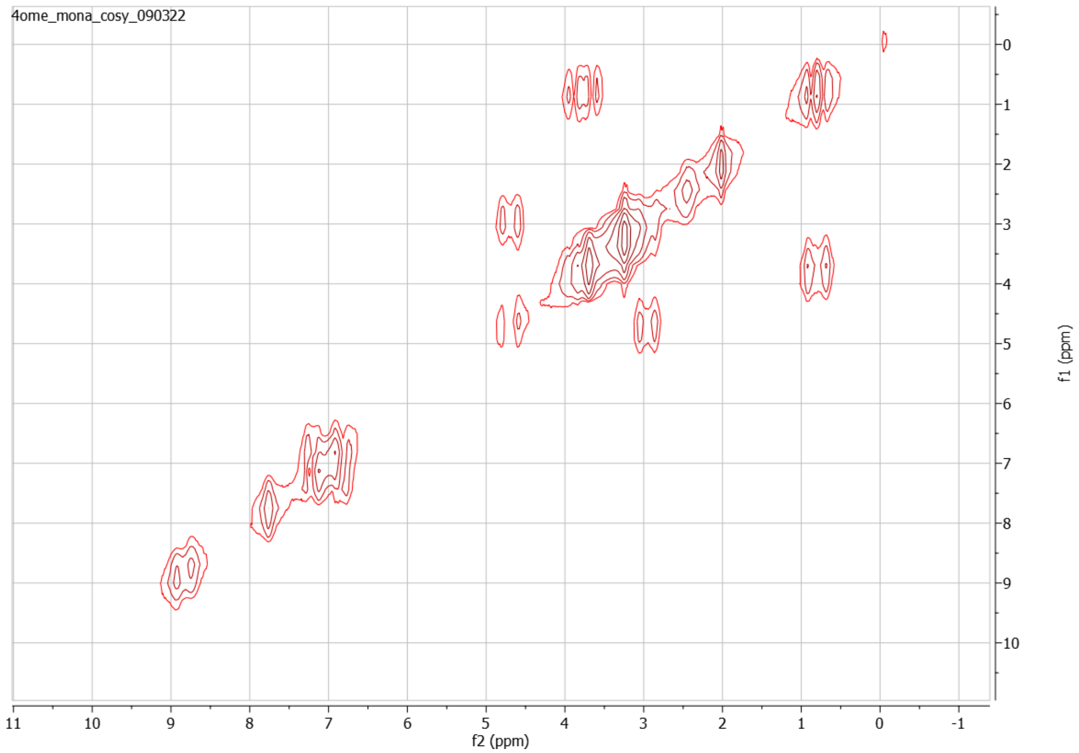

ethyl (4*S*,5*R*)-4-hydroxy-6-(4-hydroxyphenyl)-2-thioxo-4-(trifluoromethyl)hexahydropyrimidine-5-carboxylate (Compound 3m - 4-hydroxy substitution):

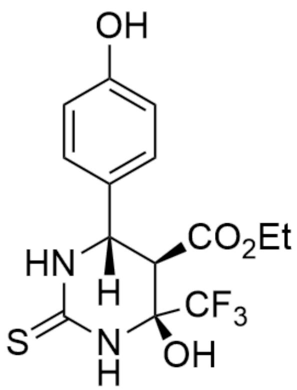

Following the general procedure yielded a white solid (235.36 mg, 64.60% qNMR isolated yield).

MW: 364.34 g/mol

Experimentalists: RC, SS

TLC R_f = 0.4125 (50% EtOAc/Hex), UV active

LCMS *m/z:* Calcd for $C_{14}H_{14}F_3N_2O_4S^-$ 363.07 $[M-H]^-$, Found 363.17 $[M-H]^-$

^{1}H NMR (60 MHz, DMSO-*d*$_6$) δ 9.44 (s, 1H), 8.92 (s, 1H), 8.78 (s, 1H), 7.78 (s, 1H), 7.10 (d, *J* = 8.6 Hz, 2H), 6.69 (d, *J* = 8.5 Hz, 2H), 4.68 (d, J = 11.9 Hz, 1H), 3.81 (q, J = 7.0 Hz, 2H), 2.95 (d, J = 11.8 Hz, 1H), 0.84 (t, J = 7.1 Hz, 3H).

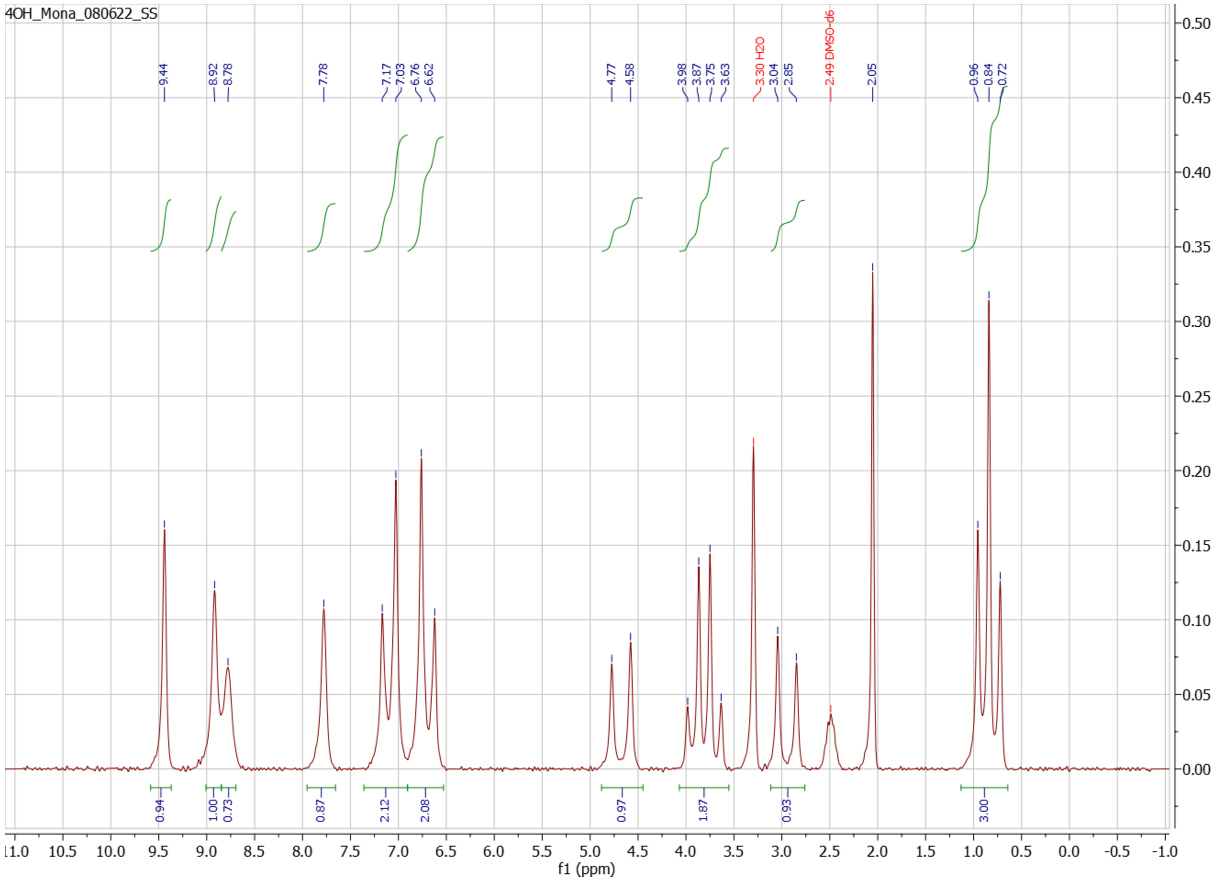

^{13}C NMR (15 MHz, DMSO-d_6) δ 176.96, 166.56, 157.52, 129.24, 126.87, 115.12, 60.39, 53.73, 49.37, 13.51.

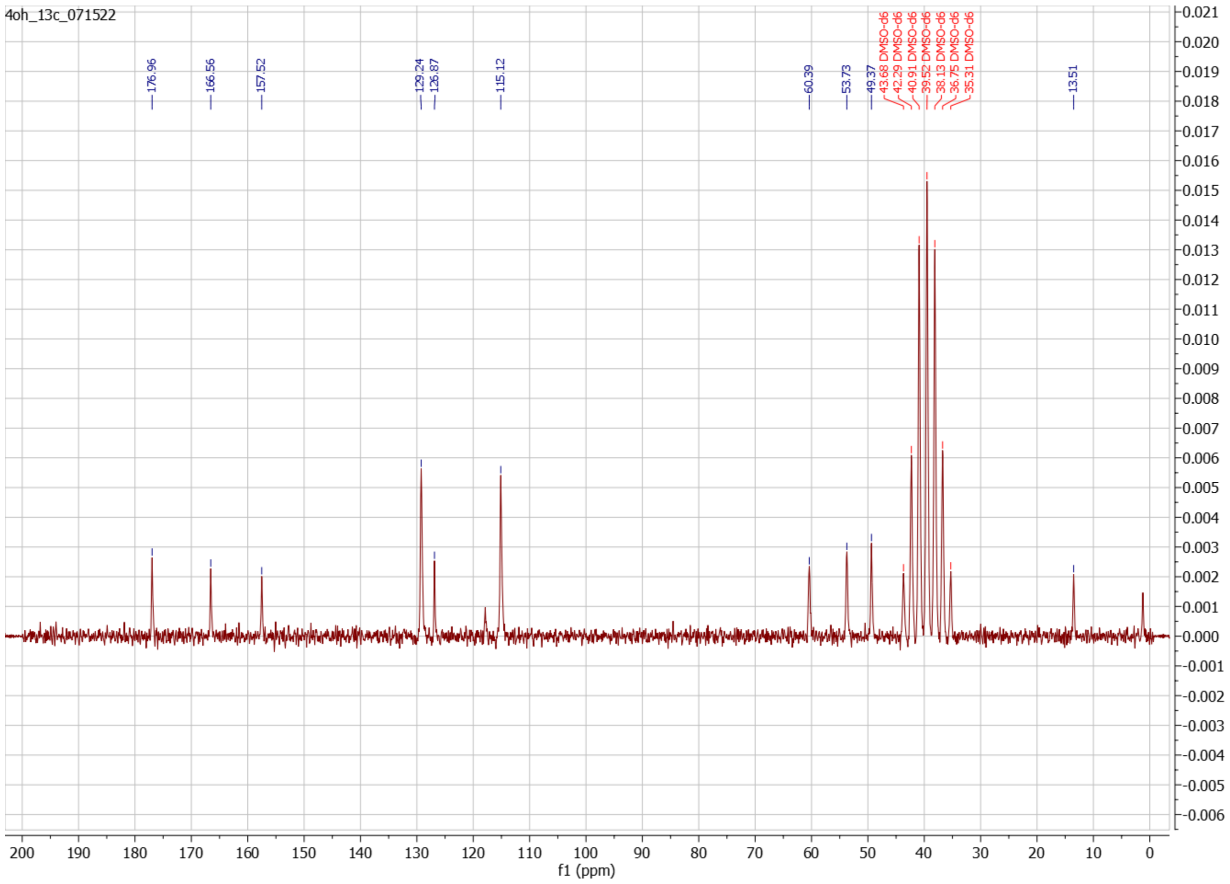

^{19}F NMR (60 MHz, DMSO-d_6) δ -80.46.

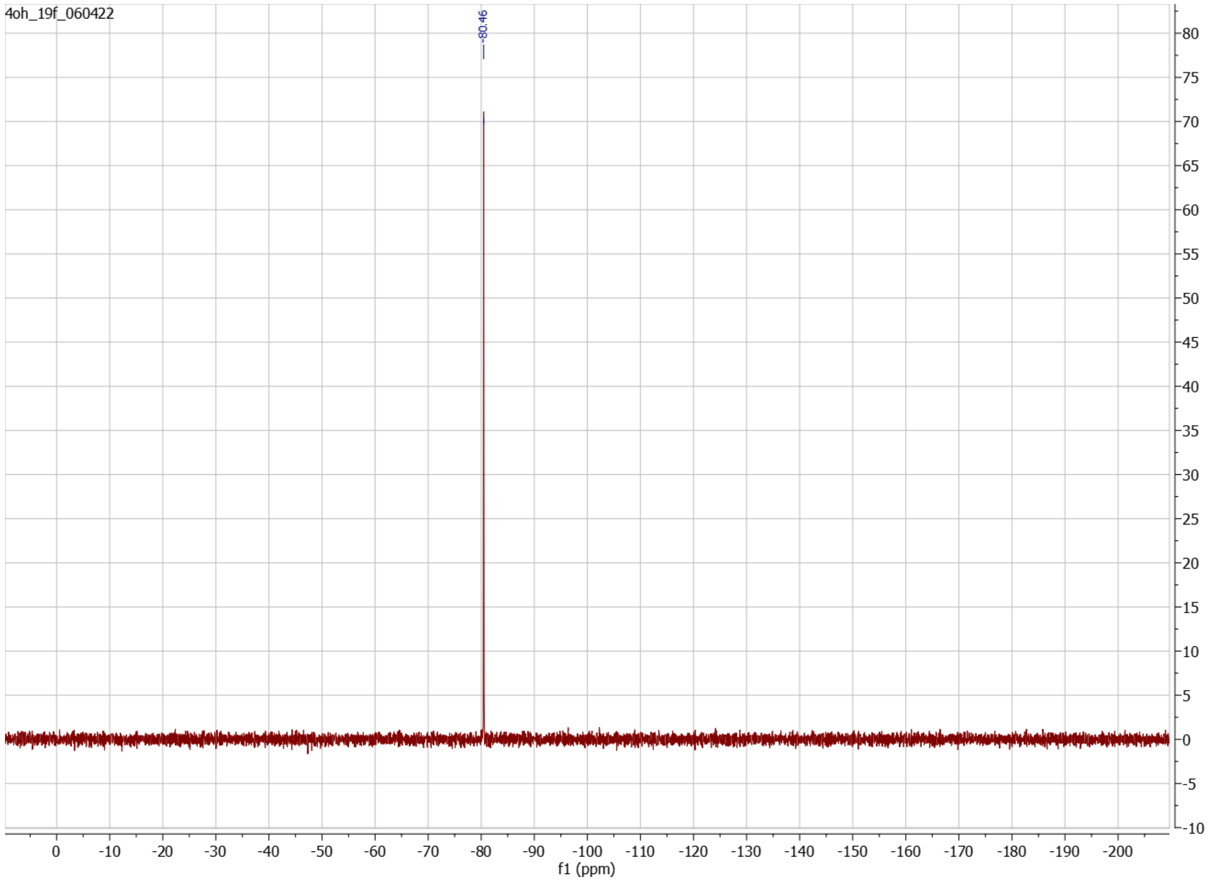

FT-IR (neat, ATR): 3310.46, 2361.17, 2337.23, 1726.26, 1614.59, 1553.94, 1515.79, 1448.49, 1377.59, 1344.08, 1308.84, 1245.18, 1194.45, 1109.85, 1021.82, 832.42, 708.77, 668.45, 640.79, 615.69, 610.85, 604.09, 586.44, 577.36, 567.88, 560.81, 552.18

COSY 2D NMR (60 MHz, DMSO-d_6)

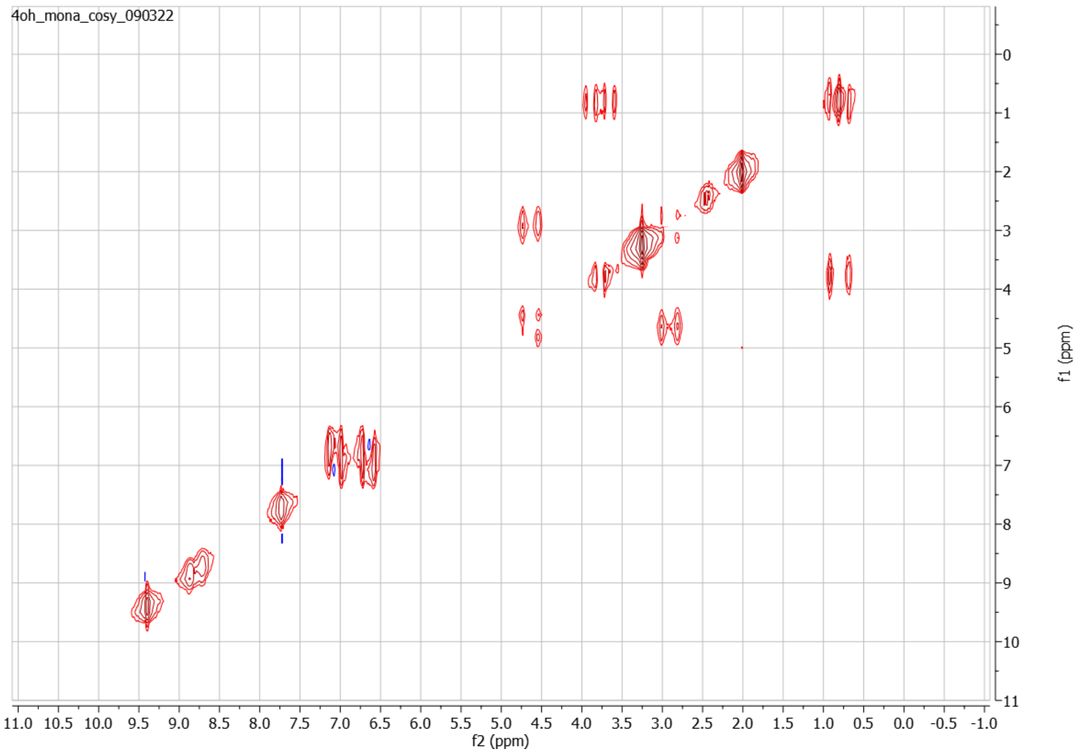

ethyl (4*S*,5*R*)-4-hydroxy-2-thioxo-6-(p-tolyl)-4-(trifluoromethyl)hexahydropyrimidine-5-carboxylate (Compound 3n - 4-methyl substitution):

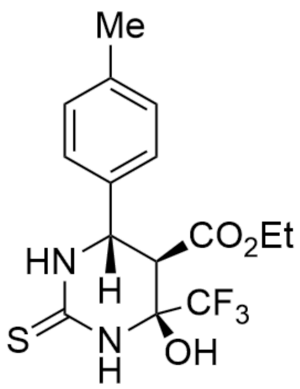

Following the general procedure yielded a white solid (161.39 mg, 44.54% qNMR isolated yield).

MW: 362.37 g/mol

Experimentalists: RC

TLC R_f = 0.75 (50% EtOAc/Hex), UV active

LCMS *m/z:* Calcd for $C_{15}H_{18}F_3N_2O_3S^+$ 363.10 $[M+H]^+$, Found 363.17 $[M+H]^+$

^{1}**H NMR** (60 MHz, DMSO-d_6) δ 9.00 (s, 1H), 8.87 (s, 1H), 7.83 (s, 1H), 7.16 (s, 4H), 4.74 (d, J = 11.9 Hz, 1H), 3.80 (q, J = 7.1 Hz, 2H), 2.98 (d, J = 11.9 Hz, 1H), 2.28 (s, 3H), 0.83 (t, J = 7.1 Hz, 3H).

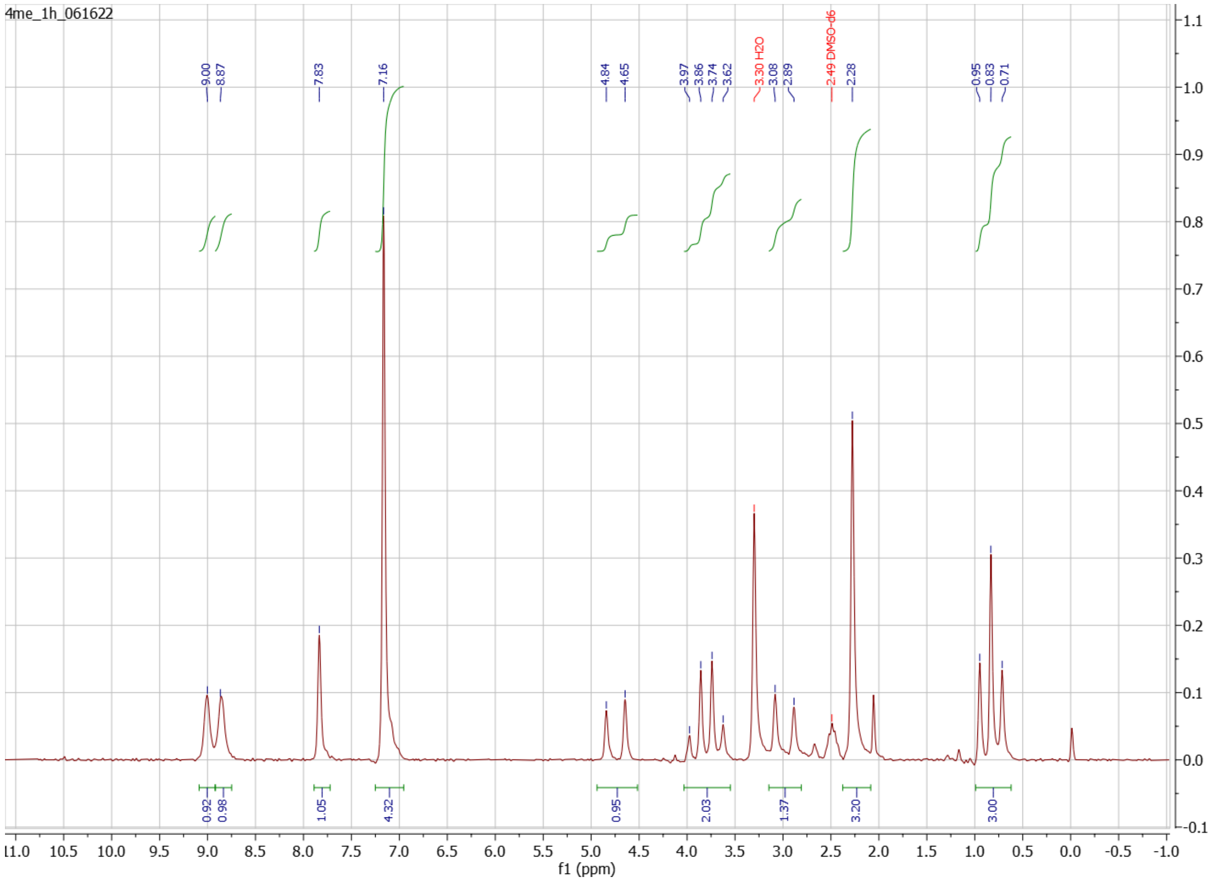

^{13}C NMR (15 MHz, DMSO-d_6) δ 177.08, 166.48, 137.80, 133.92, 128.88, 127.95, 60.39, 53.92, 49.32, 20.70, 13.40.

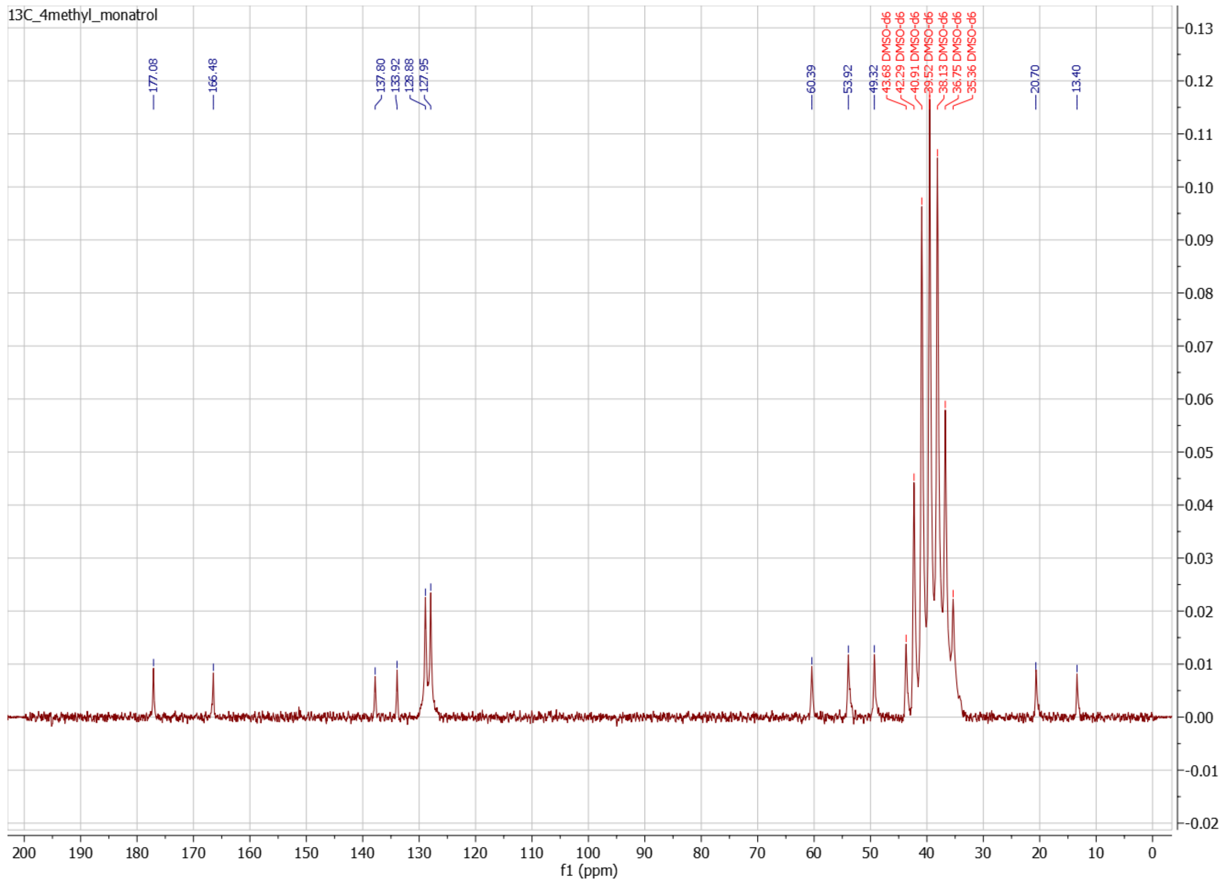

^{19}**F NMR** (60 MHz, DMSO-d_6) δ -80.41.

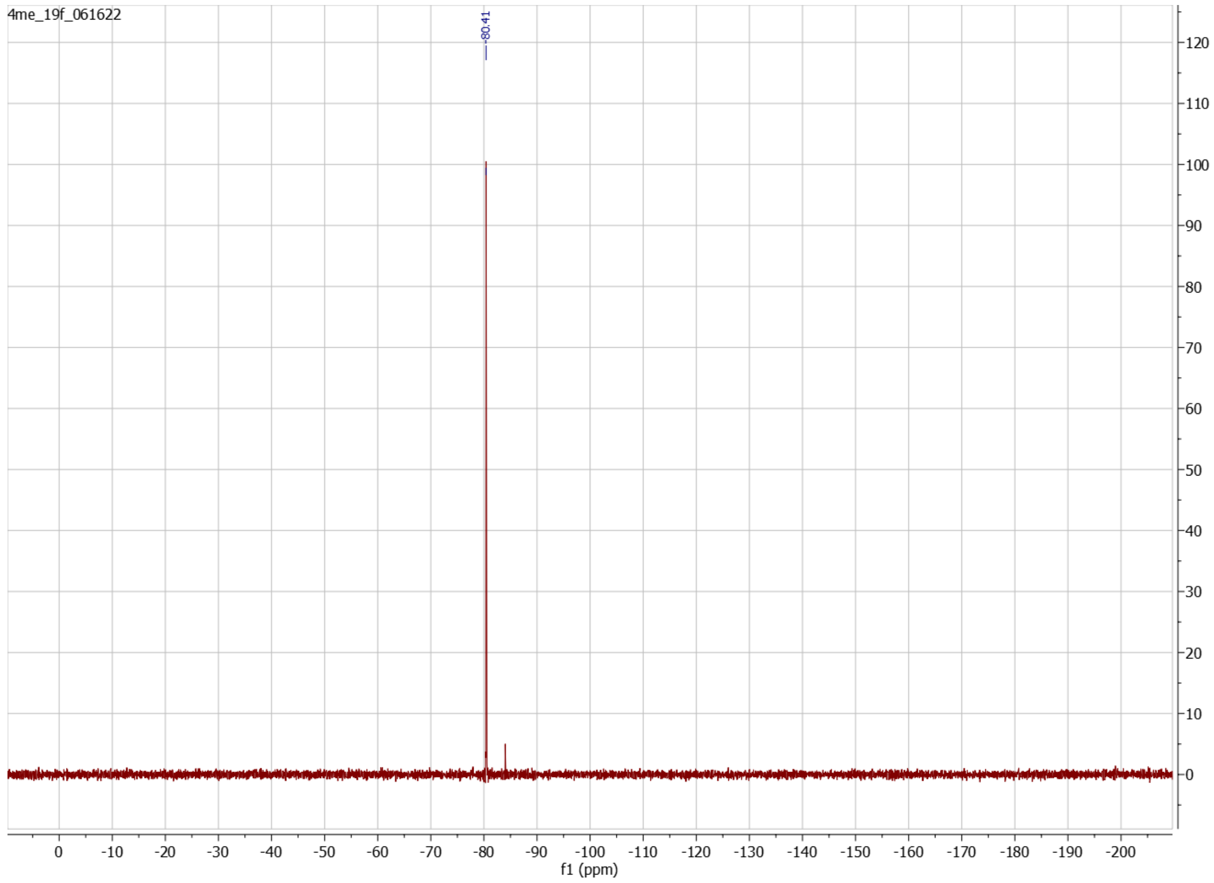

FT-IR (neat, ATR): 3189.74, 1735.74, 1552.51, 1499.63, 1377.34, 1342.6, 1243.81, 1192.02, 1022.51, 850.85, 812.89, 707.25, 620.96, 559.08

COSY 2D NMR (60 MHz, DMSO-d_6)

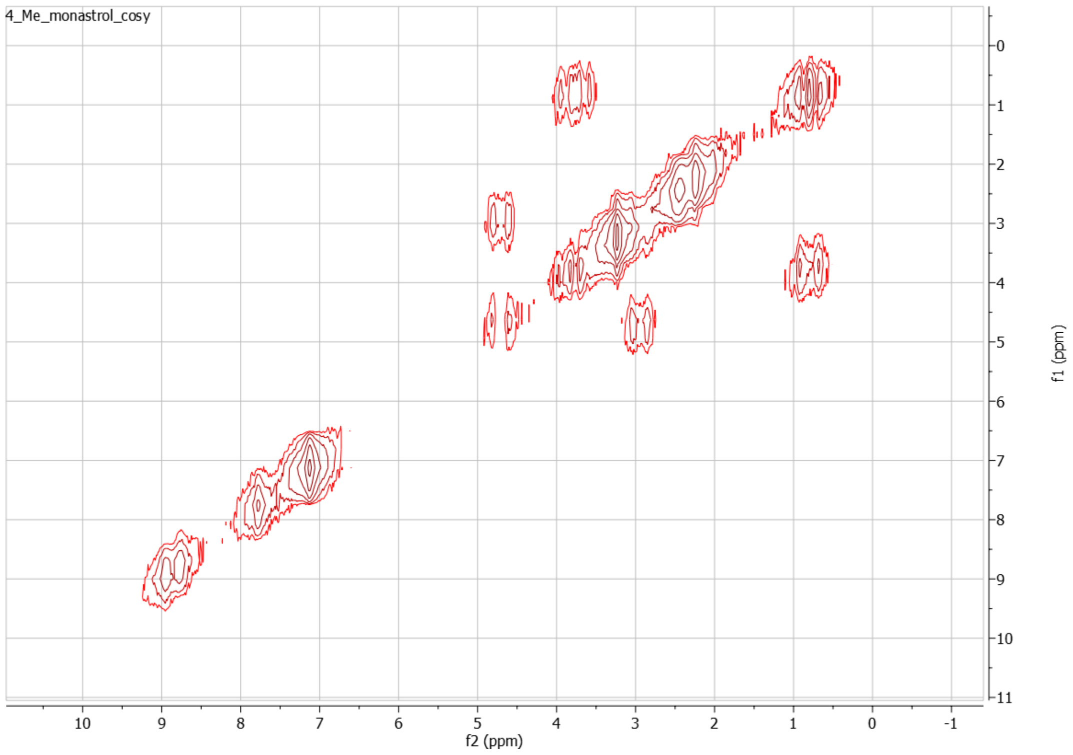

ethyl (4*S*,5*R*)-6-(4-bromophenyl)-4-hydroxy-2-thioxo-4-(trifluoromethyl)hexahydropyrimidine-5-carboxylate (Compound 3o - 4-bromo substitution):

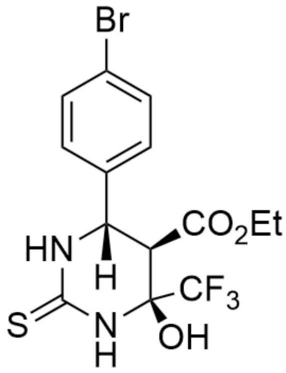

Following the general procedure yielded a white solid (386.39 mg, 90.44% qNMR isolated yield).

MW: 427.24 g/mol

Experimentalists: RC

TLC R_f = 0.75 (50% EtOAc/Hex), UV active

LCMS *m/z:* Calcd for $C_{14}H_{15}BrF_3N_2O_3S^+$ 426.99 $[M+H]^+$, Found 427.08 $[M+H]^+$

^{1}H NMR (60 MHz, DMSO-d_6) δ 9.11 (s, 1H), 8.94 (s, 1H), 7.88 (s, 1H), 7.42 (q, J = 8.4 Hz, 4H), 4.78 (d, J = 11.9 Hz, 1H), 3.83 (q, J = 7.1 Hz, 2H), 3.05 (d, J = 11.8 Hz, 1H), 0.84 (t, J = 7.1 Hz, 3H).

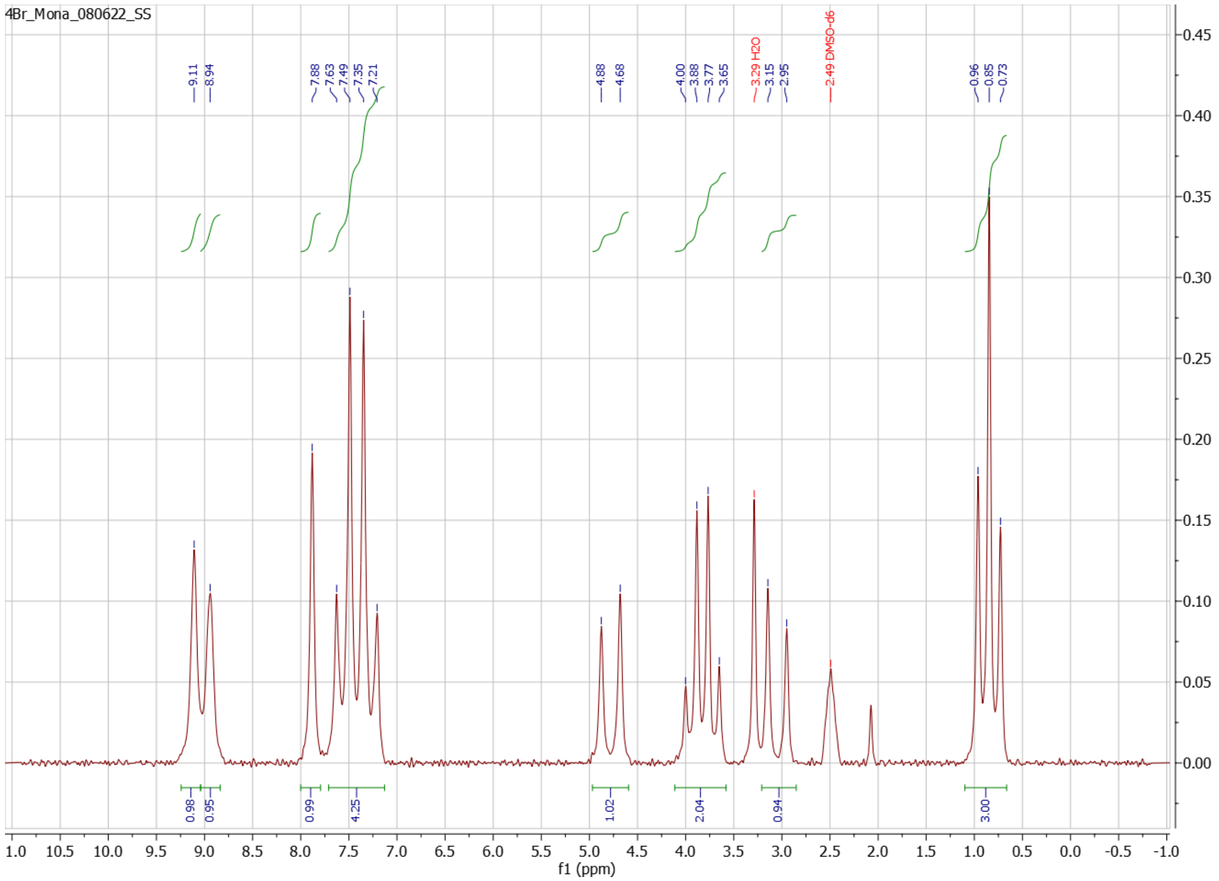

^{13}C NMR (15 MHz, DMSO-d_6) δ 177.12, 166.33, 136.27, 131.24, 130.40, 121.71, 60.53, 53.66, 48.96, 13.40.

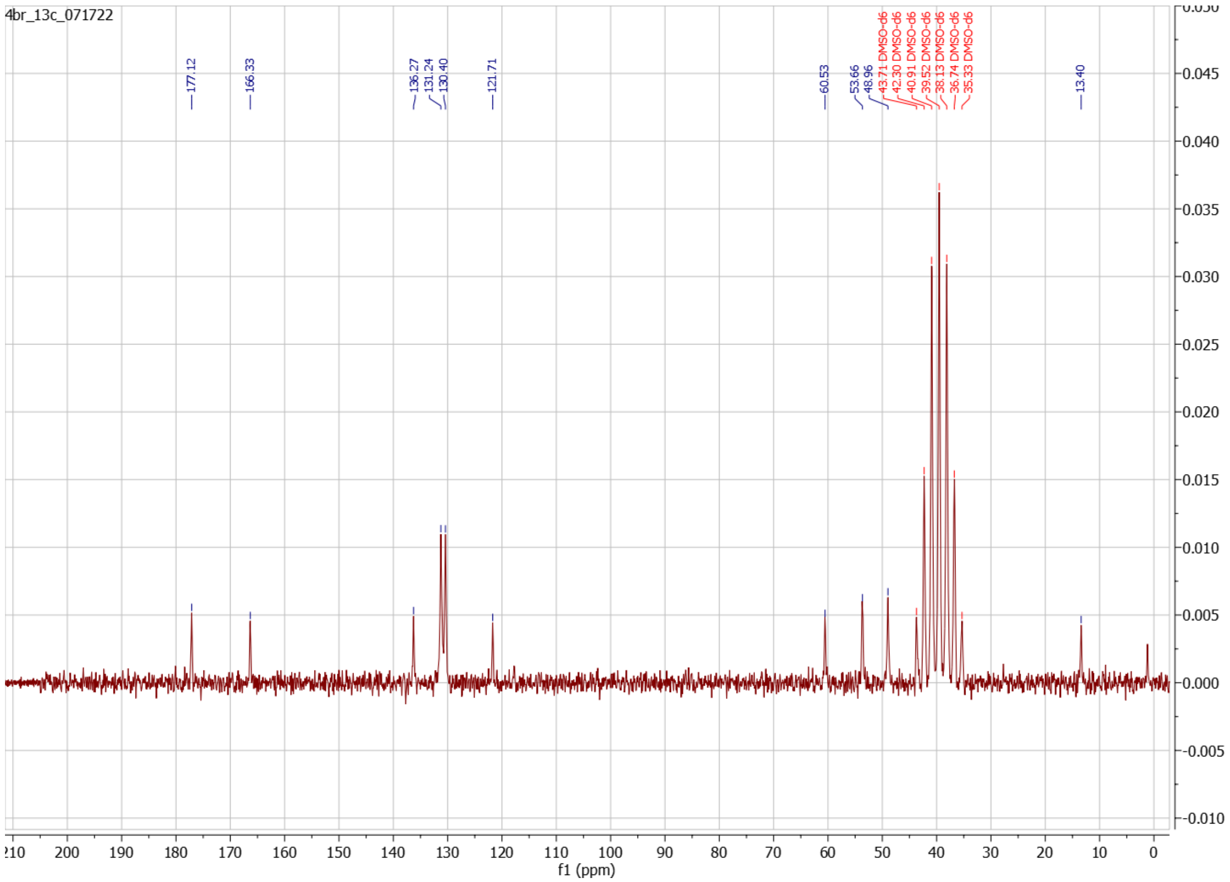

^{19}F NMR (60 MHz, DMSO-d_6) δ -80.30.

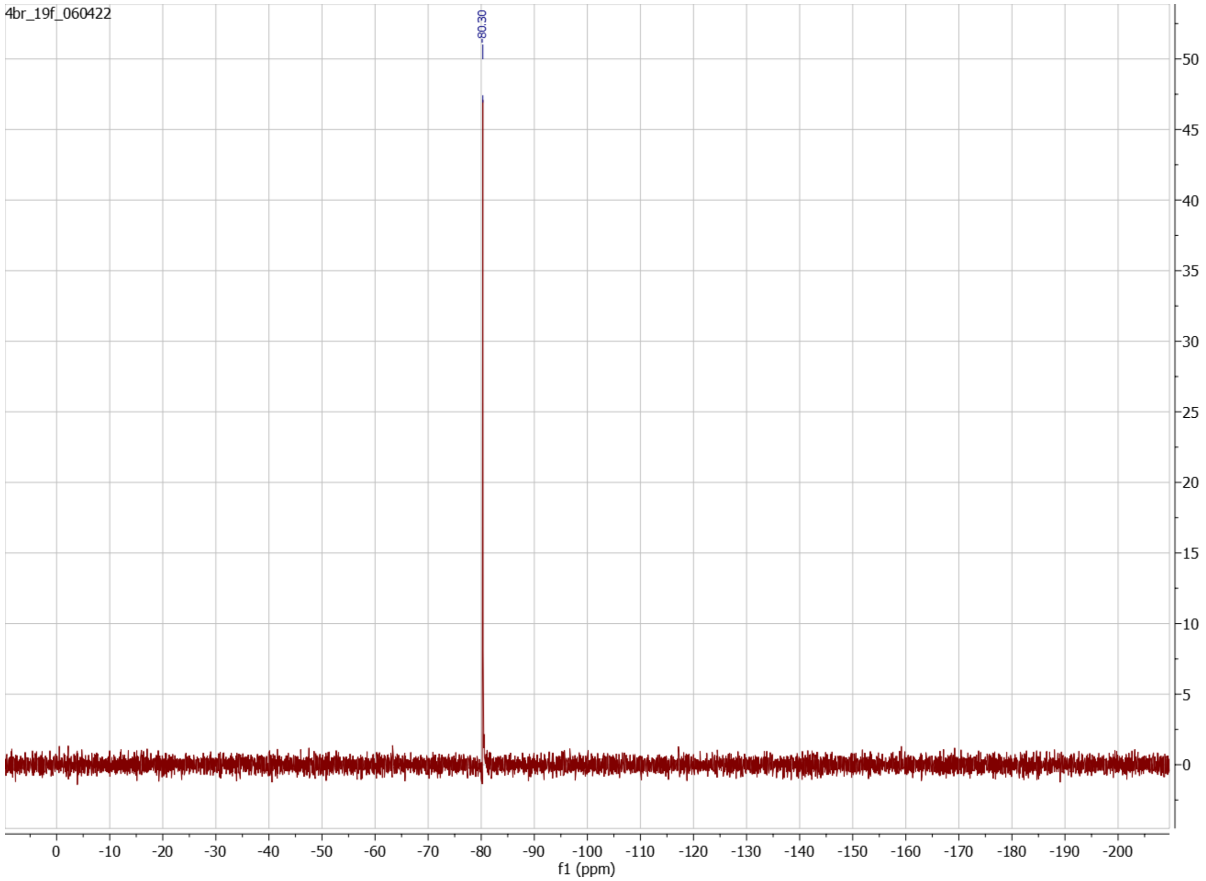

FT-IR (neat, ATR): 3182.54, 2979.56, 2359.19, 1732.61, 1683.05, 1652, 1635.04, 1557.79, 1506.17, 1488.89, 1456.35, 1396.2, 1375.54, 1340.03, 1307.93, 1243.29, 1193.3, 1011.55, 829.44, 712.43, 667.68, 640.3, 625.02, 611.95, 599.15, 586.28, 574, 563.57, 554.19

COSY 2D NMR (60 MHz, DMSO-d_6)

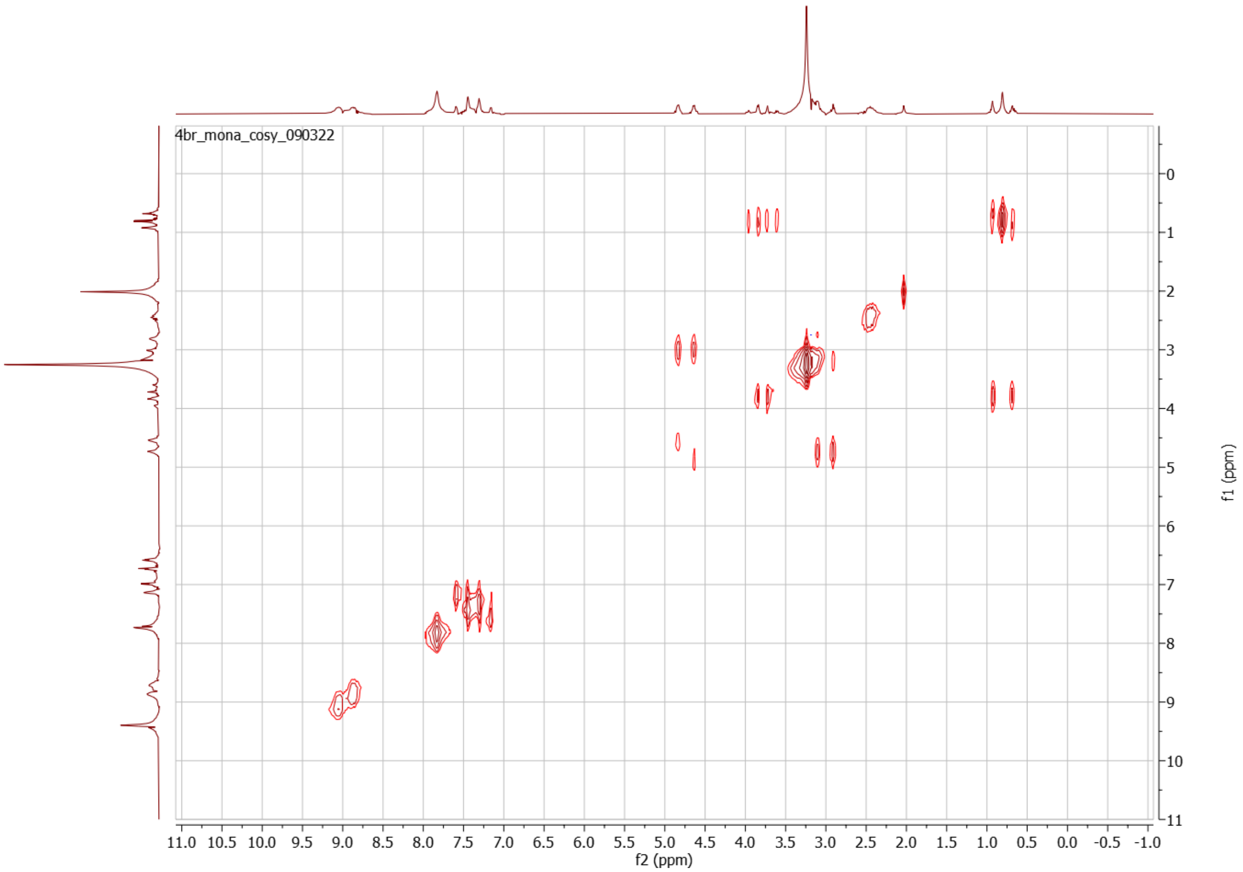

ethyl (4*S*,5*R*)-6-(4-chlorophenyl)-4-hydroxy-2-thioxo-4-(trifluoromethyl)hexahydropyrimidine-5-carboxylate (Compound 3p - 4-chloro substitution):

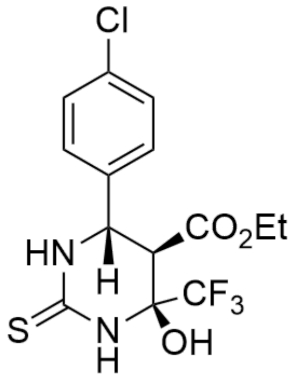

Following the general procedure yielded a white solid (297.84 mg, 77.81% qNMR isolated yield).

MW: 382.78 g/mol

Experimentalists: RC

TLC R_f = 0.75 (50% EtOAc/Hex), UV active

LCMS *m/z:* Calcd for $C_{14}H_{13}ClF_3N_2O_3S^-$ 381.03 [M-H]$^-$, Found 381.12 [M-H]$^-$

^{1}**H NMR** (60 MHz, DMSO-d_6) δ 9.11 (s, 1H), 8.95 (s, 1H), 7.89 (s, 1H), 7.38 (s, 4H), 4.80 (d, J = 11.8 Hz, 1H), 3.82 (q, J = 7.1 Hz, 2H), 3.06 (d, J = 11.8 Hz, 1H), 0.84 (t, J = 7.0 Hz, 3H).

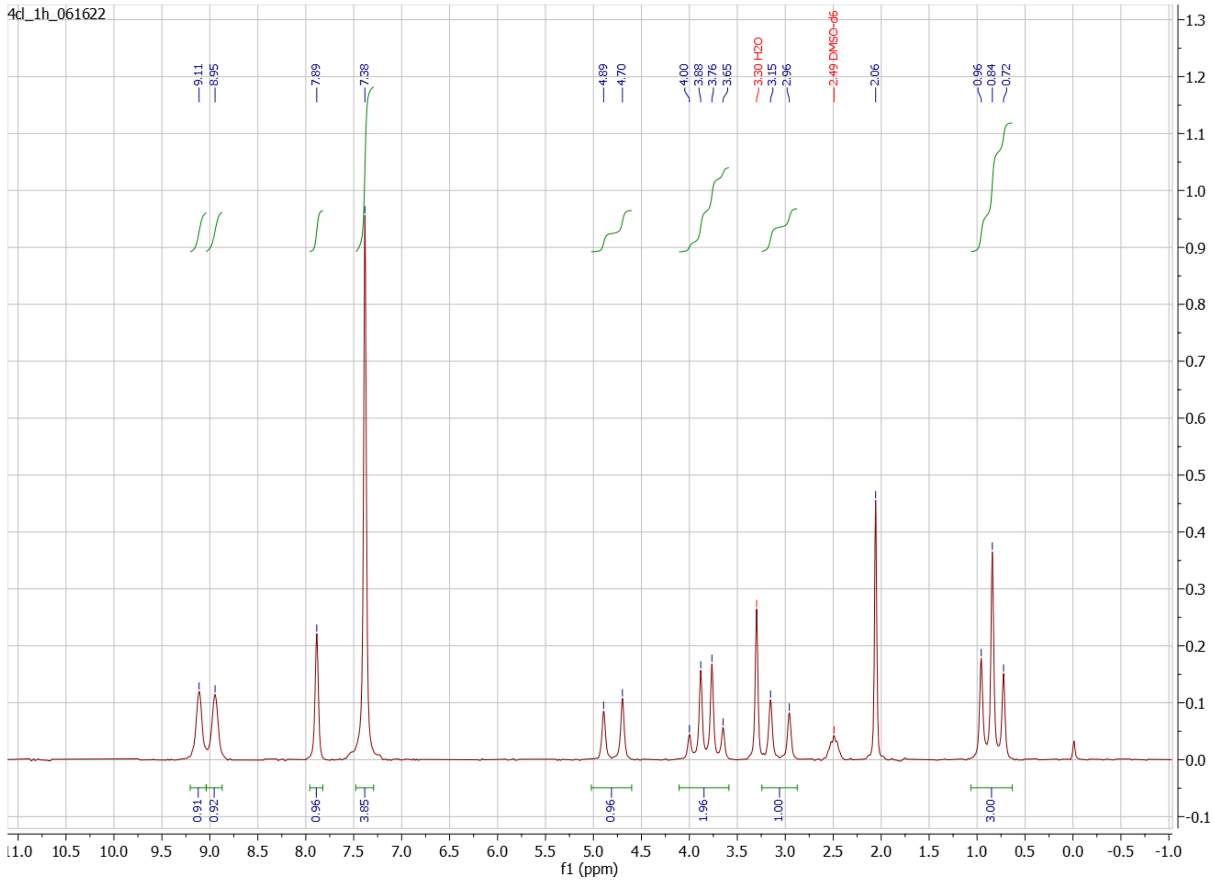

^{13}C NMR (15 MHz, DMSO-d_6) δ 177.64, 166.87, 136.35, 133.65, 130.63, 128.82, 61.01, 54.10, 49.53, 13.90.

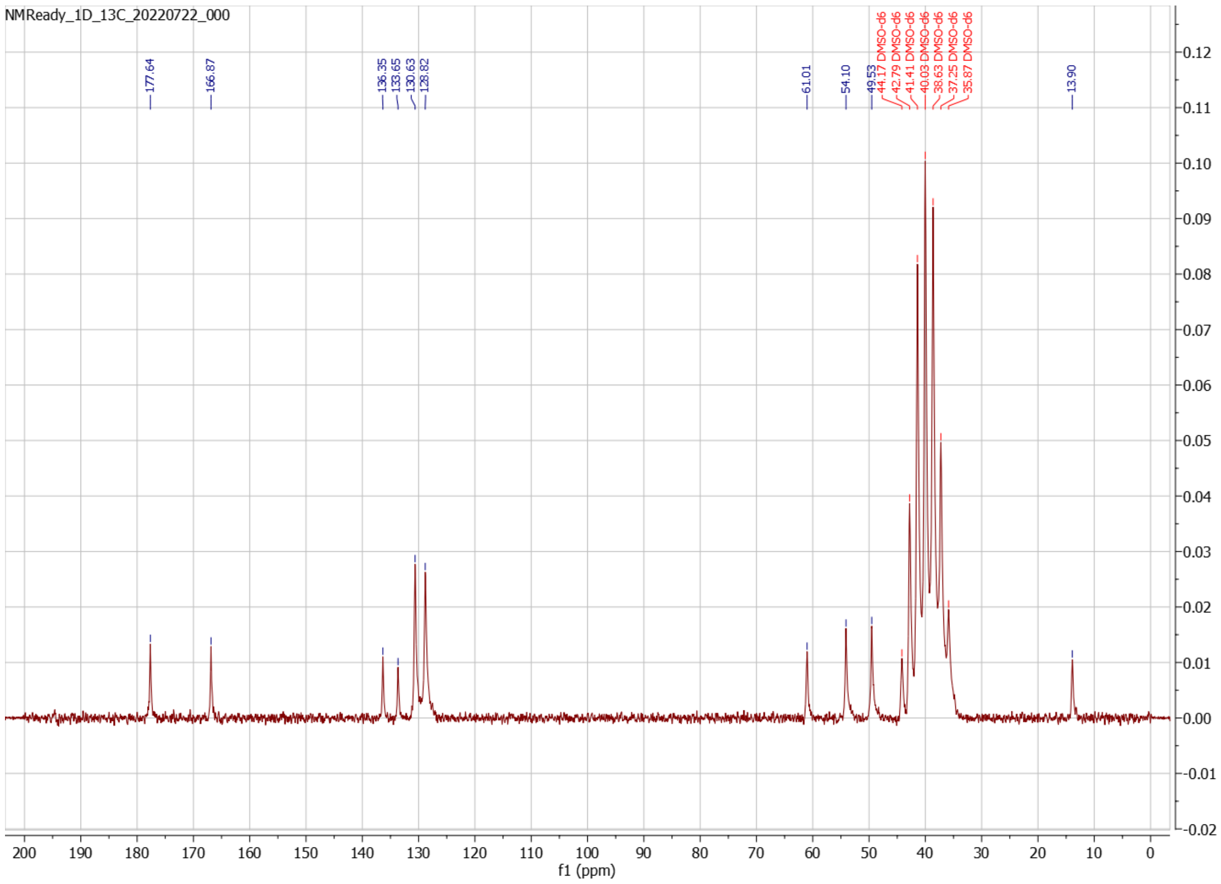

^{19}F NMR (60 MHz, DMSO-d_6) δ -80.30.

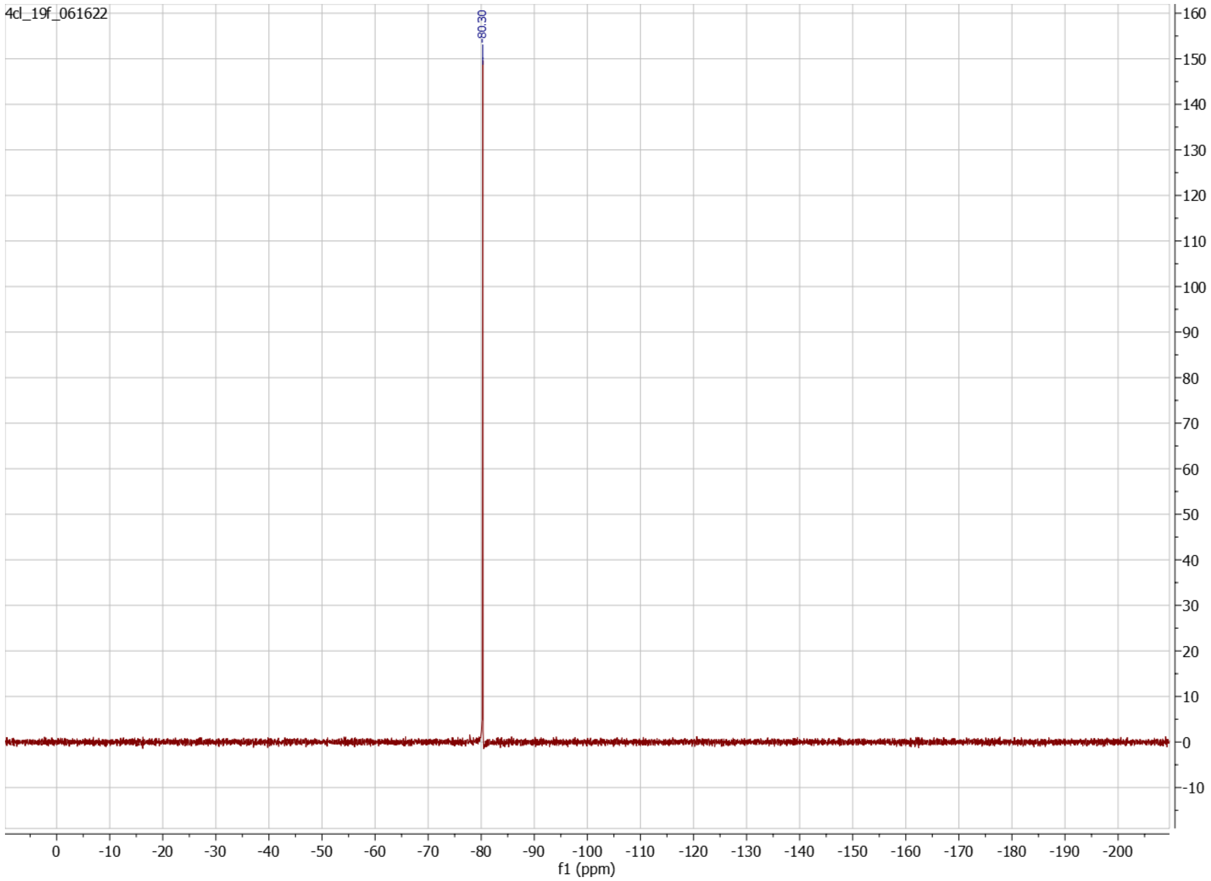

FT-IR (neat, ATR): 3188.82, 2983.34, 1735.96, 1567.36, 1492.05, 1377.88, 1347.47, 1279.76, 1254.44, 1194.24, 1139.1, 1090.32, 1032.46, 1014.48, 831.78, 573.38, 555.54

COSY 2D NMR (60 MHz, DMSO-d_6)

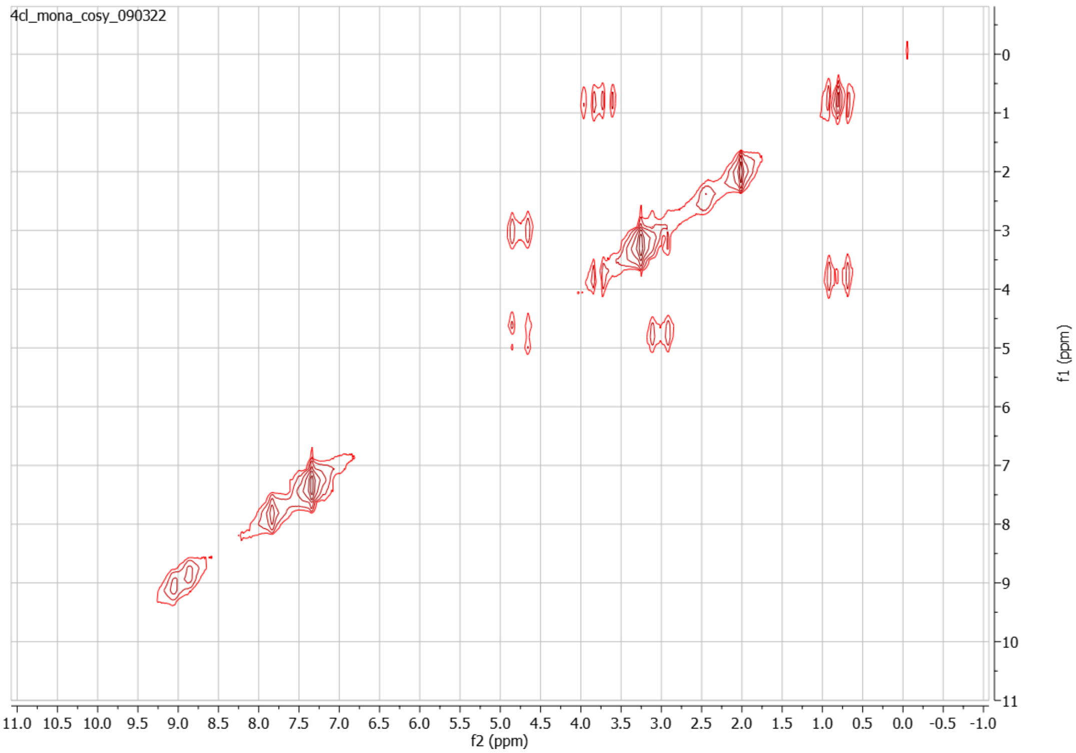

ethyl (4*S*,5*R*)-6-(4-fluorophenyl)-4-hydroxy-2-thioxo-4-(trifluoromethyl)hexahydropyrimidine-5-carboxylate (Compound 3q - 4-fluoro substitution):

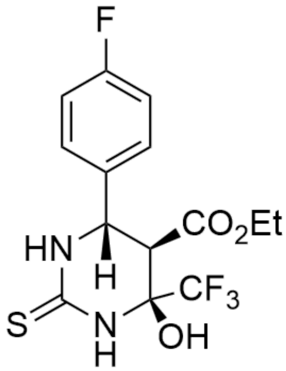

Following the general procedure yielded a white solid (218.11 mg, 59.54% qNMR isolated yield).

MW: 366.33 g/mol

Experimentalists: RC

TLC R_f = 0.7375 (50% EtOAc/Hex), UV active

LCMS *m/z:* Calcd for $C_{14}H_{15}F_4N_2O_3S^+$ 367.07 [M+H]$^+$, Found 367.05 [M+H]

^{1}**H NMR** (60 MHz, DMSO-*d*$_6$) δ 9.09 (s, 1H), 8.90 (s, 1H), 7.86 (s, 1H), 7.55 – 6.95 (m, 5H), 4.79 (d, J = 11.9 Hz, 1H), 3.81 (q, J = 7.1 Hz, 2H), 3.05 (d, J = 11.9 Hz, 1H), 0.82 (t, J = 7.0 Hz, 3H).

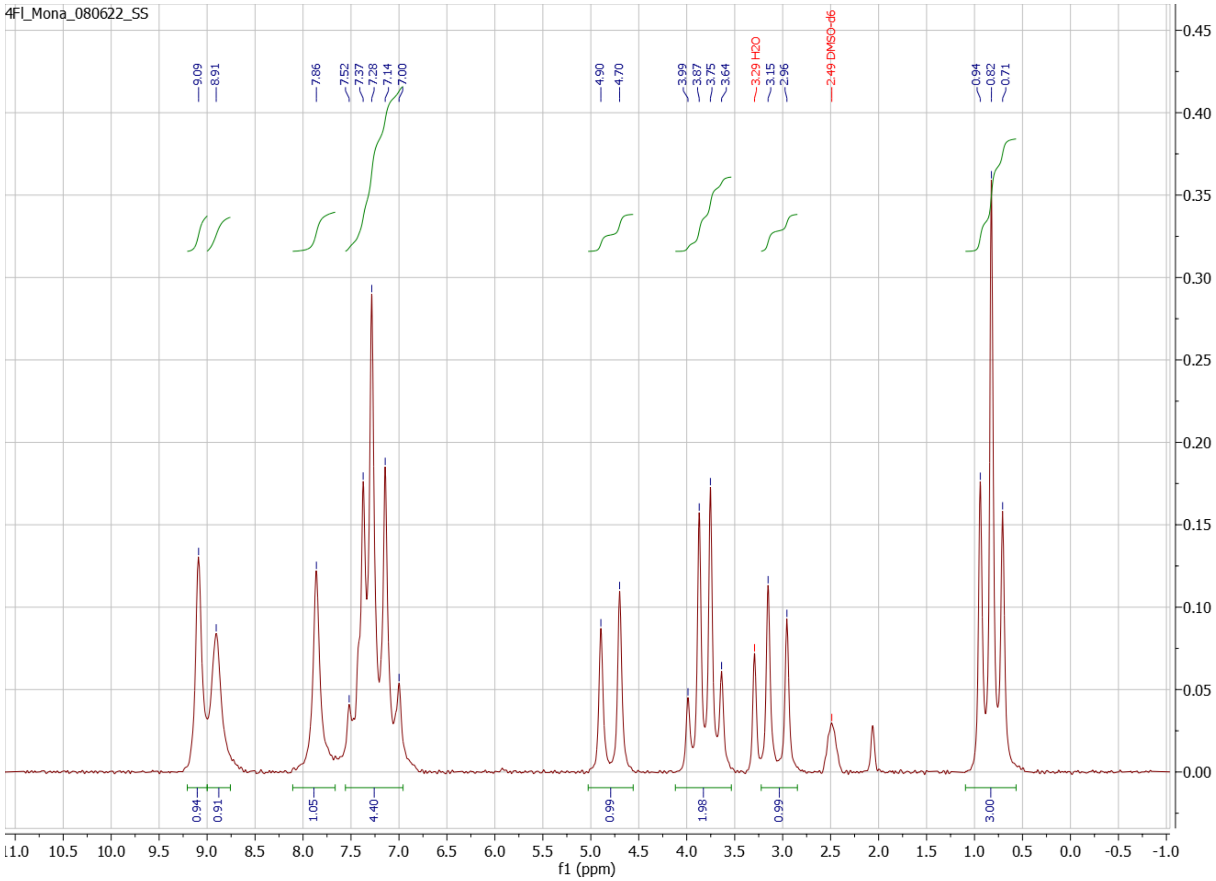

^{13}C NMR (15 MHz, DMSO-d_6) δ 177.19, 166.51, 133.08, 130.40, 115.82, 114.49, 60.49, 53.58, 49.21, 13.43.

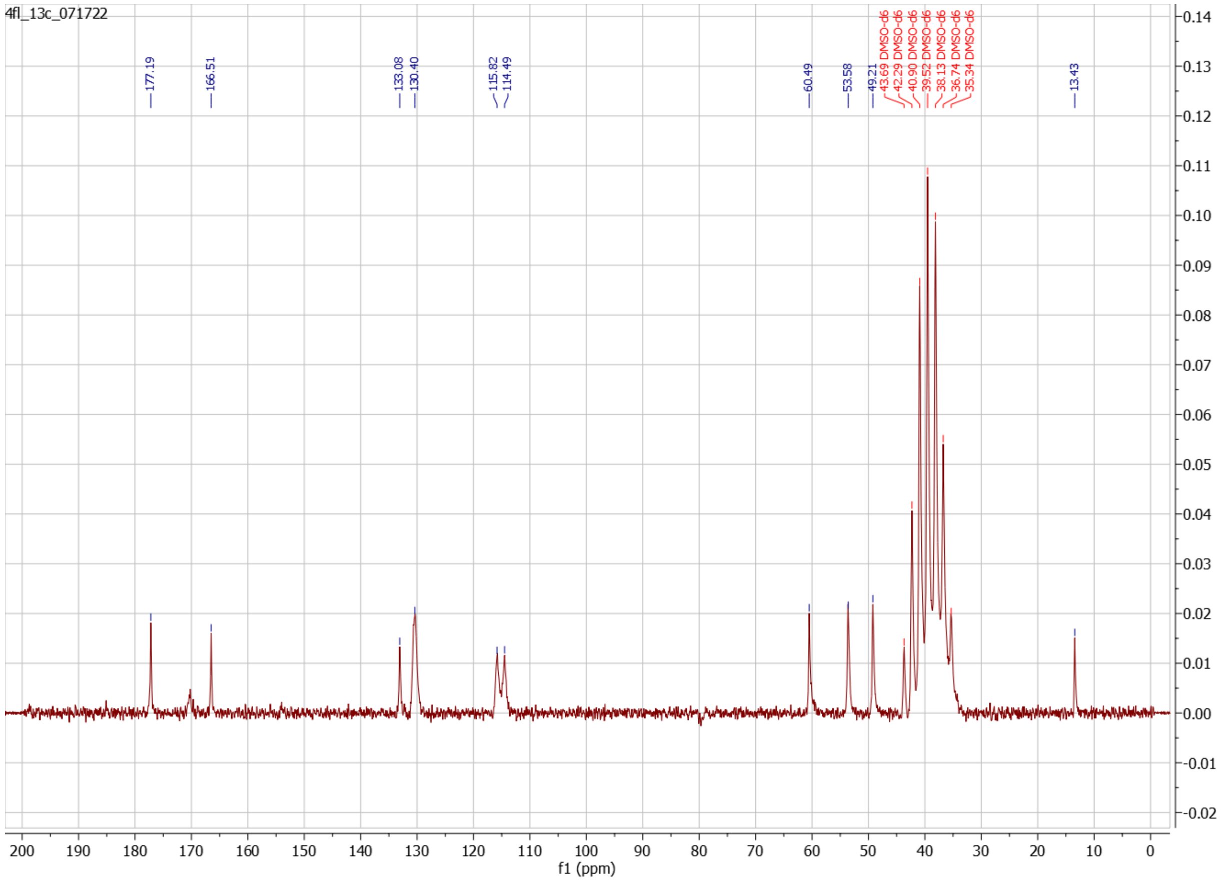

^{19}F NMR (60 MHz, DMSO-d_6) δ -80.33, -113.64 J = 5.2 Hz.

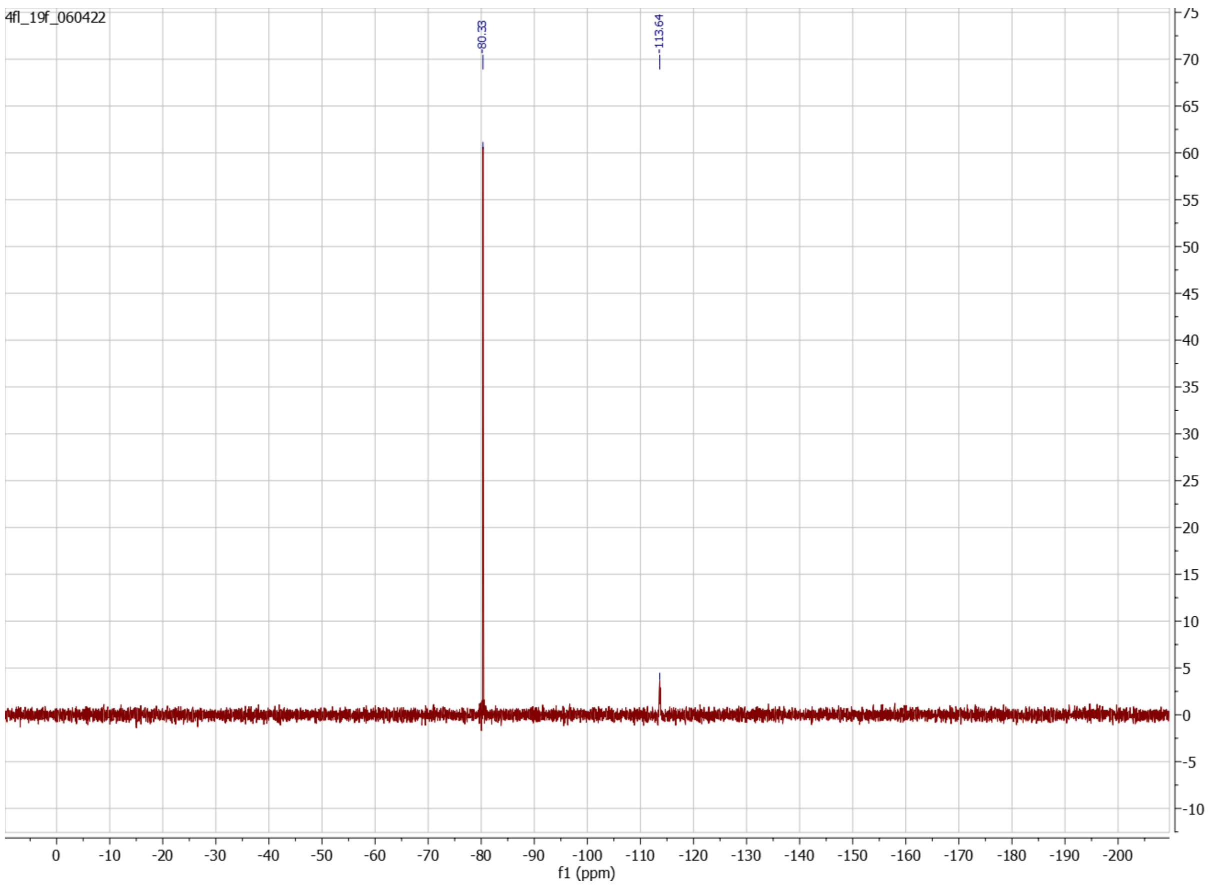

FT-IR (neat, ATR): 3190.06, 2982, 1735.86, 1606.74, 1556.07, 1510.58, 1377.49, 1343.22, 1308.39, 1232.95, 1192.23, 1161.17, 1099.76, 1022.39, 833.94, 706.67, 608.3, 554.71

COSY 2D NMR (60 MHz, DMSO-d_6)

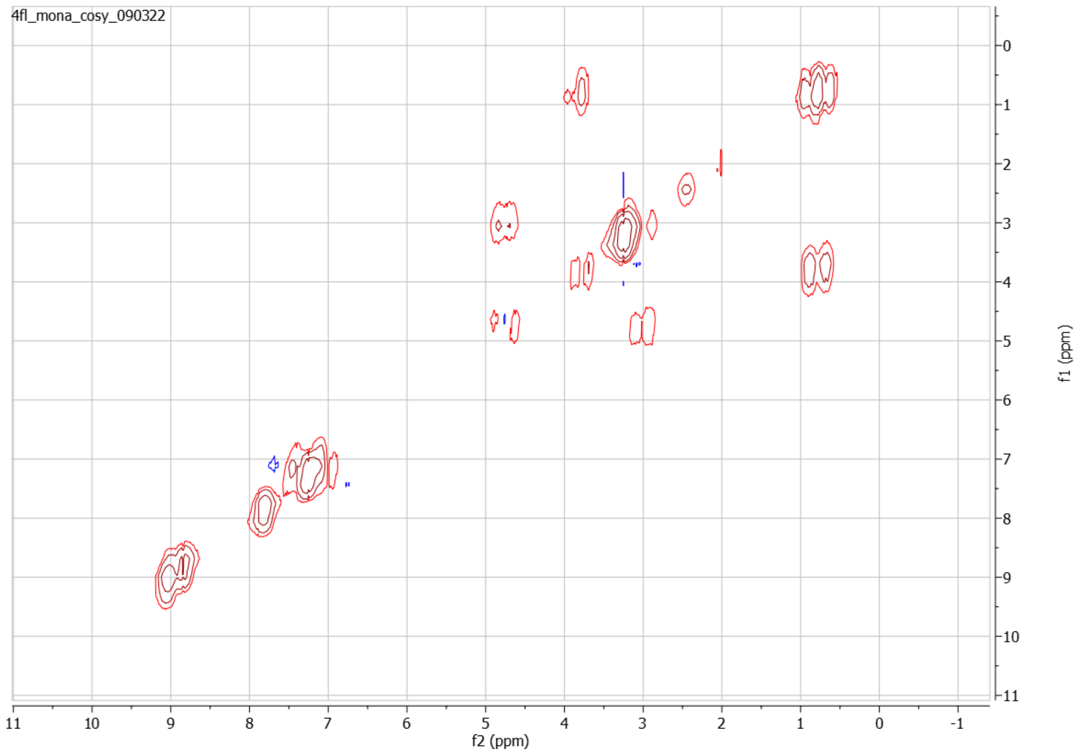

ethyl (4*S*,5*R*)-6-(4-cyanophenyl)-4-hydroxy-2-thioxo-4-(trifluoromethyl)hexahydropyrimidine-5-carboxylate (Compound 3r - 4-cyano substitution):

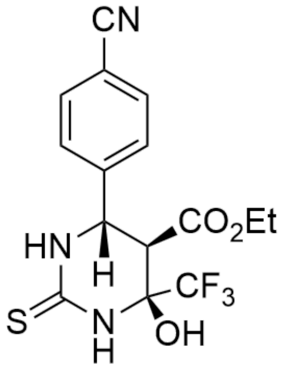

Following the general procedure yielded a white solid (231.59 mg, 62.03% qNMR isolated yield).

MW: 373.35 g/mol

Experimentalists: RC, SS

TLC R_f = 0.575 (50% EtOAc/Hex), UV active

LCMS *m/z:* Calcd for $C_{15}H_{15}F_3N_3O_3S^+$ 374.08 $[M+H]^+$, Found 374.03 $[M+H]^+$

^{1}H NMR (60 MHz, DMSO-d_6) δ 9.19 (s, 1H), 9.01 (s, 1H), 7.92 (s, 1H), 7.69 (app. m., 5H), 4.87 (d, J = 11.7 Hz, 1H), 3.81 (q, J = 7.1 Hz, 2H), 3.13 (d, J = 11.8 Hz, 1H), 0.82 (t, J = 7.0 Hz, 3H).

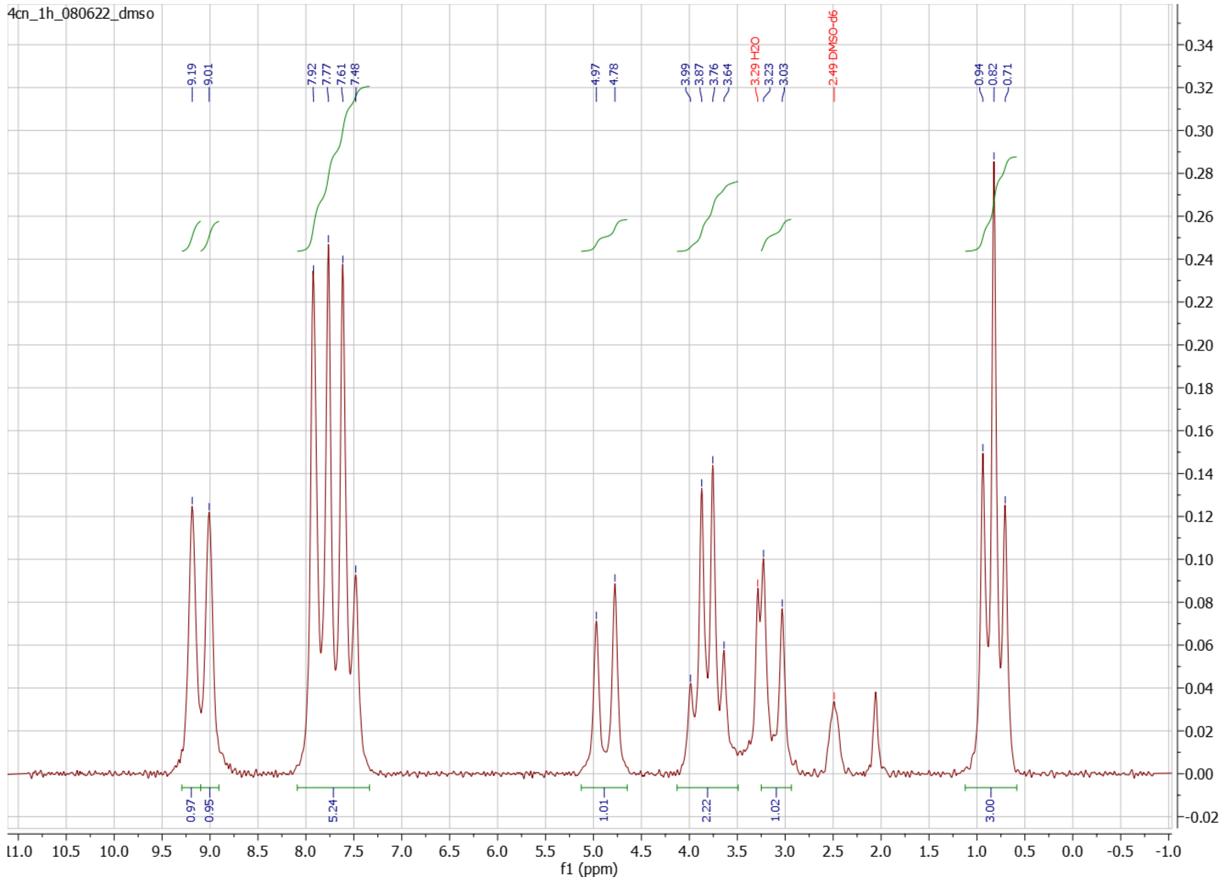

^{13}C NMR (15 MHz, DMSO-d_6) δ 177.24, 166.29, 142.39, 132.27, 129.49, 118.39, 111.35, 60.63, 53.98, 48.73, 13.38.

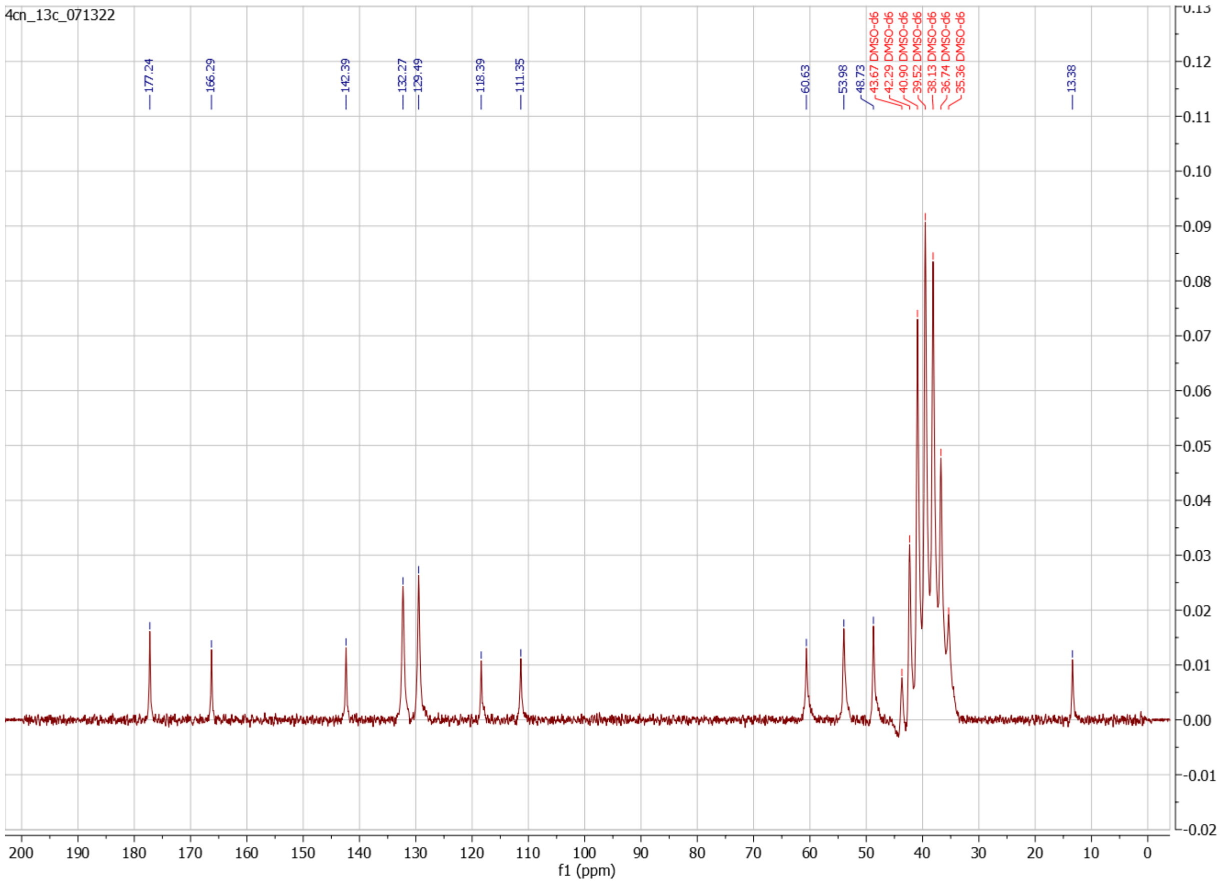

^{19}F NMR (60 MHz, DMSO-d_6) δ -80.19.

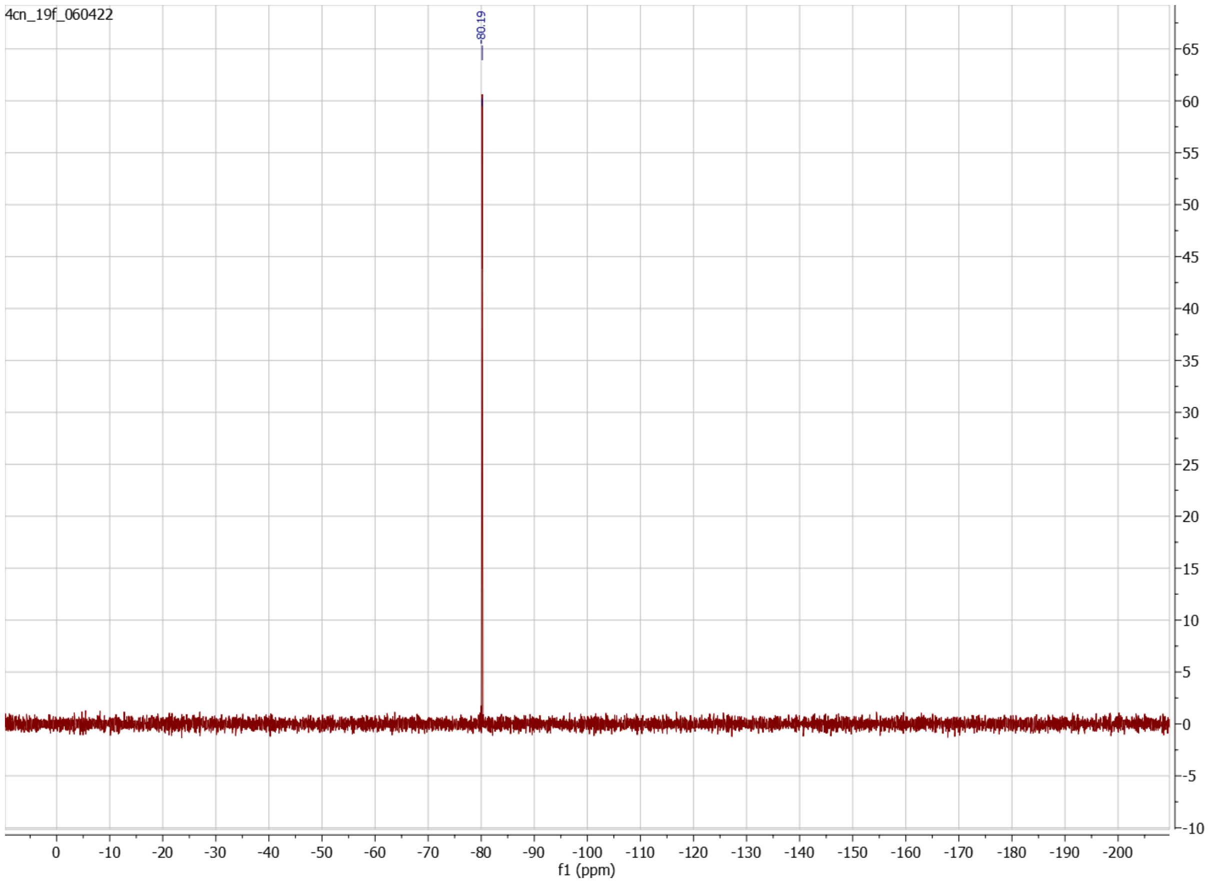

FT-IR (neat, ATR): 3332.29, 2972.45, 2883.36, 1651.36, 1379.43, 1086.98, 1045.17, 879.21, 666.35

COSY 2D NMR (60 MHz, DMSO-d_6)

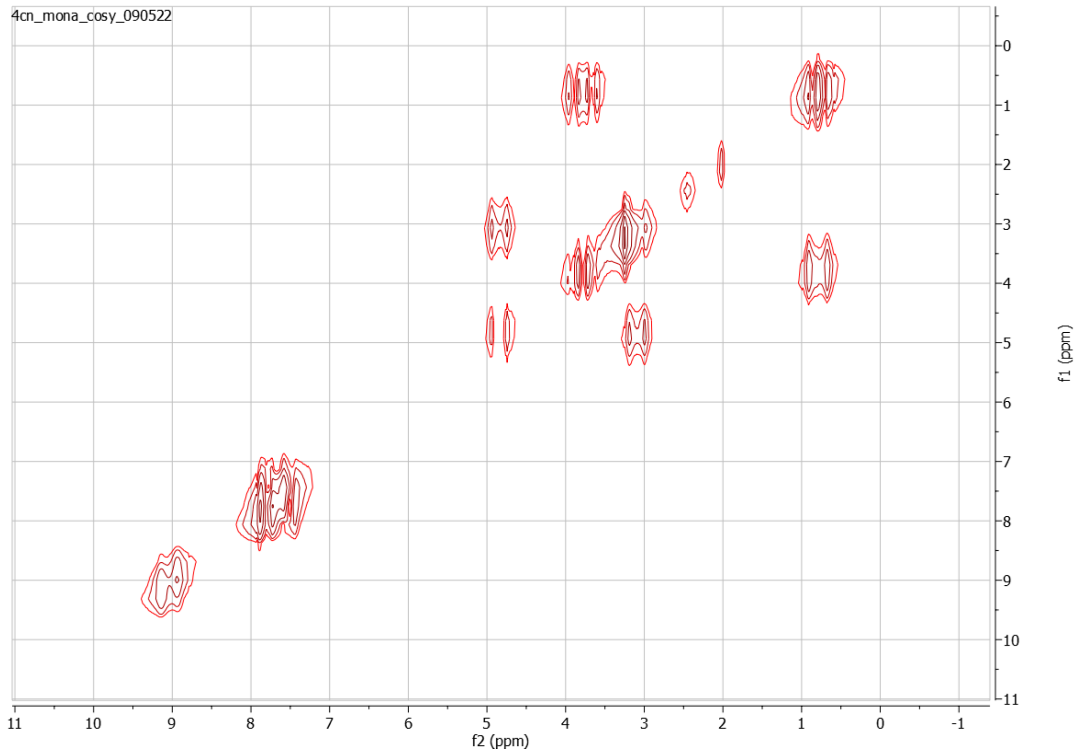

ethyl (4*S*,5*R*)-4-hydroxy-6-(4-nitrophenyl)-2-thioxo-4-(trifluoromethyl)hexahydropyrimidine-5-carboxylate (Compound 3s - 4-nitro substitution):

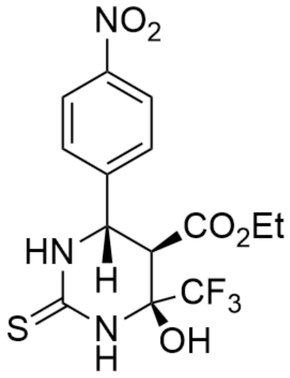

Following the general procedure yielded a white solid (201.70 mg, 51.28% qNMR isolated yield).

Molar Mass: 393.34 g/mol

Experimentalists: RC

TLC R_f = 0.625 (50% EtOAc/Hex), UV active

LCMS *m/z:* Calcd for $C_{14}H_{13}F_3N_3O_5S^-$ 392.05 $[M-H]^-$, Found 392.13 $[M-H]^-$

^{1}H NMR (60 MHz, DMSO-d_6) δ 9.24 (s, 1H), 9.06 (s, 1H), 8.21 (d, J = 8.6 Hz, 2H), 7.97 (s, 1H), 7.65 (d, J = 8.8 Hz, 2H), 4.94 (d, J = 11.8 Hz, 1H), 3.82 (q, J = 7.1 Hz, 2H), 3.15 (d, J = 11.9 Hz, 1H), 0.83 (t, J = 7.0 Hz, 3H).

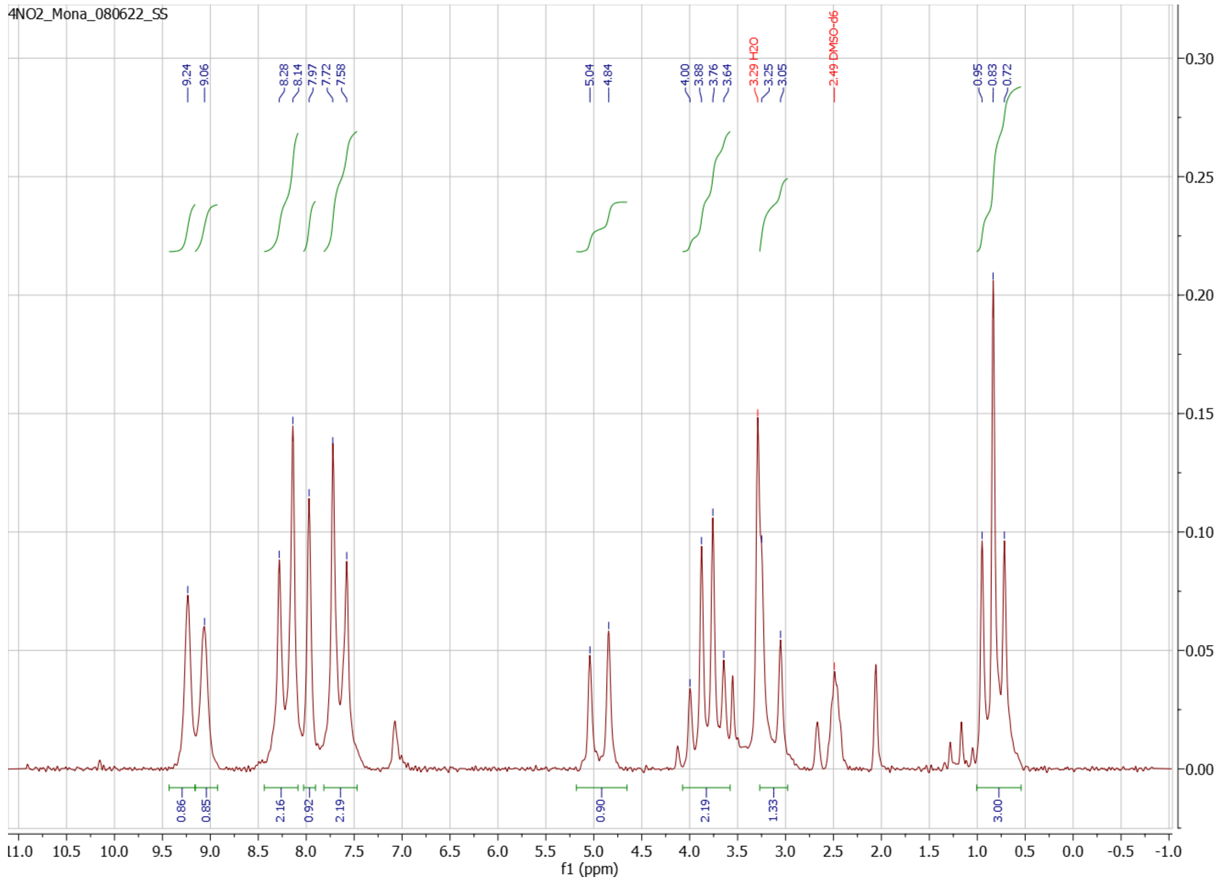

^{13}C NMR (15 MHz, DMSO-d_6) δ 177.67, 166.72, 148.05, 144.84, 130.33, 123.81, 118.39, 61.23, 54.20, 49.32, 13.88.

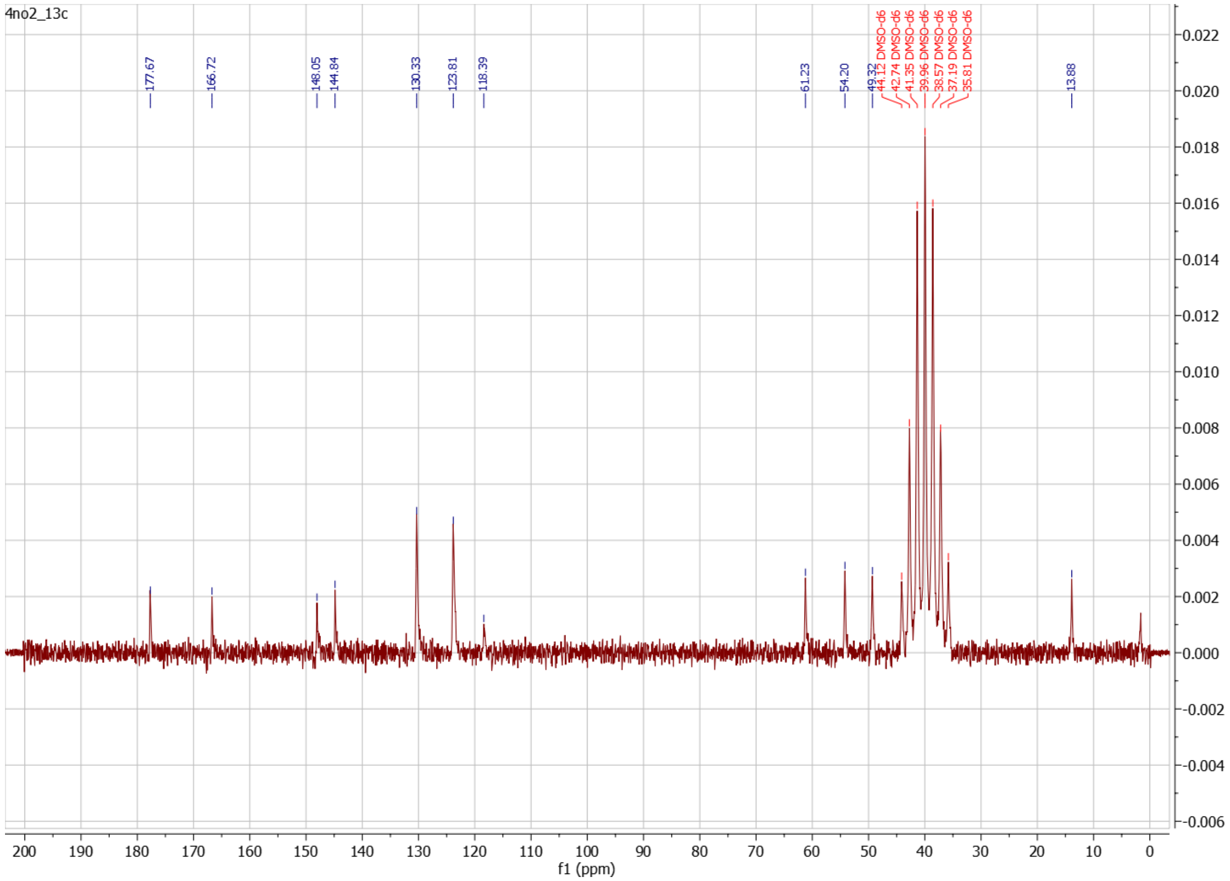

^{19}F NMR (60 MHz, DMSO-d_6) δ -80.14.

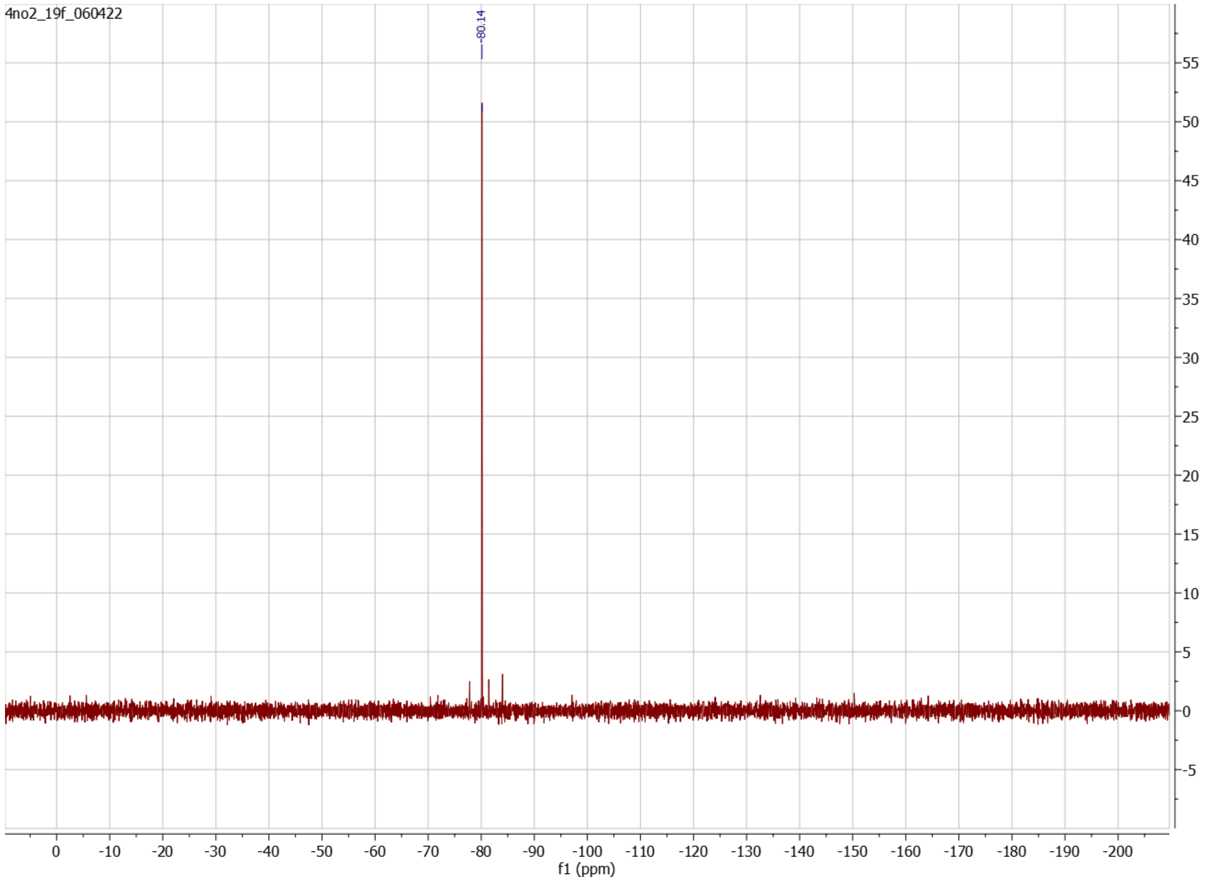

FT-IR (neat, ATR): 3199.34, 2986.64, 2357.88, 1731.82, 1682.58, 1651.75, 1569.01, 1520.27, 1472.57, 1455.9, 1397.99, 1378.68, 1349.35, 1287.66, 1265.65, 1199.69, 1141.45, 1094.39, 1031.94, 848.81, 755, 733.44, 695.98, 667.4, 649.21, 621.15, 606.69, 593.84, 586.59, 578.69, 567.67, 555.33

COSY 2D NMR (60 MHz, DMSO-d_6)

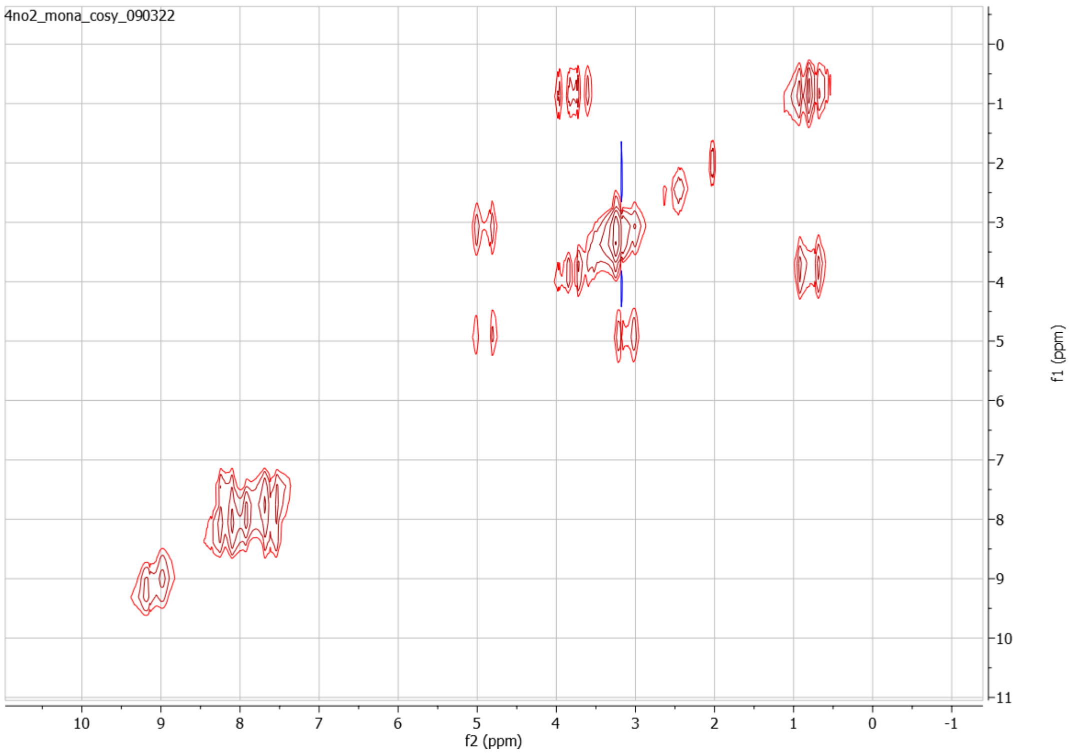

ethyl 4-(3-hydroxyphenyl)-2-thioxo-6-(trifluoromethyl)-1,2,3,4-tetrahydropyrimidine-5-carboxylate (Compound 4):

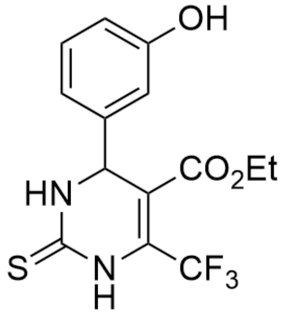

Following the dehydration procedure yielded a slightly yellow solid (213.76 mg, 30.86% qNMR isolated yield).

MW: 346.32 g/mol

Experimentalists: RC, PS, SS

TLC R_f = 0.73 (50% EtOAc/Hex), UV active

LCMS *m/z:* Calcd for $C_{13}H_{14}F_3N_2O_3S^+$ 347.06 $[M+H]^+$, Found 347.13 $[M+H]^+$

^{1}H NMR (60 MHz, DMSO-d_6) δ 10.82 (s, 1H), 9.80 (d, J = 3.4 Hz, 1H), 9.58 (s, 1H), 7.46 – 6.91 (m, 1H), 6.68 (m, 3H), 5.15 (s, 1H), 4.10 (q, J = 7.0 Hz, 2H), 1.11 (t, J = 7.0 Hz, 3H).

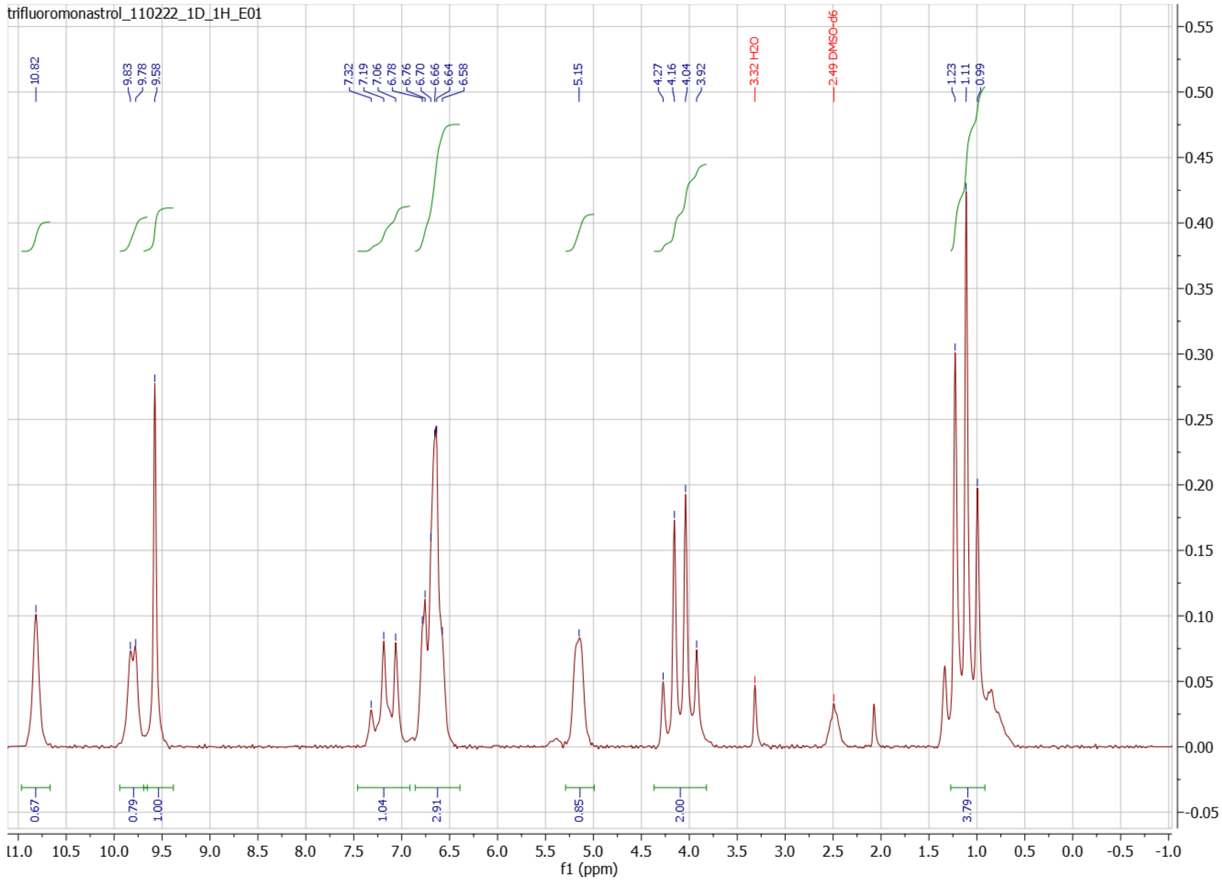

^{13}C NMR (15 MHz, DMSO-d_6) δ 174.94, 163.81, 158.21, 142.51, 130.31, 118.21 – 112.96 (m), 110.13, 61.82, 55.42, 13.91.

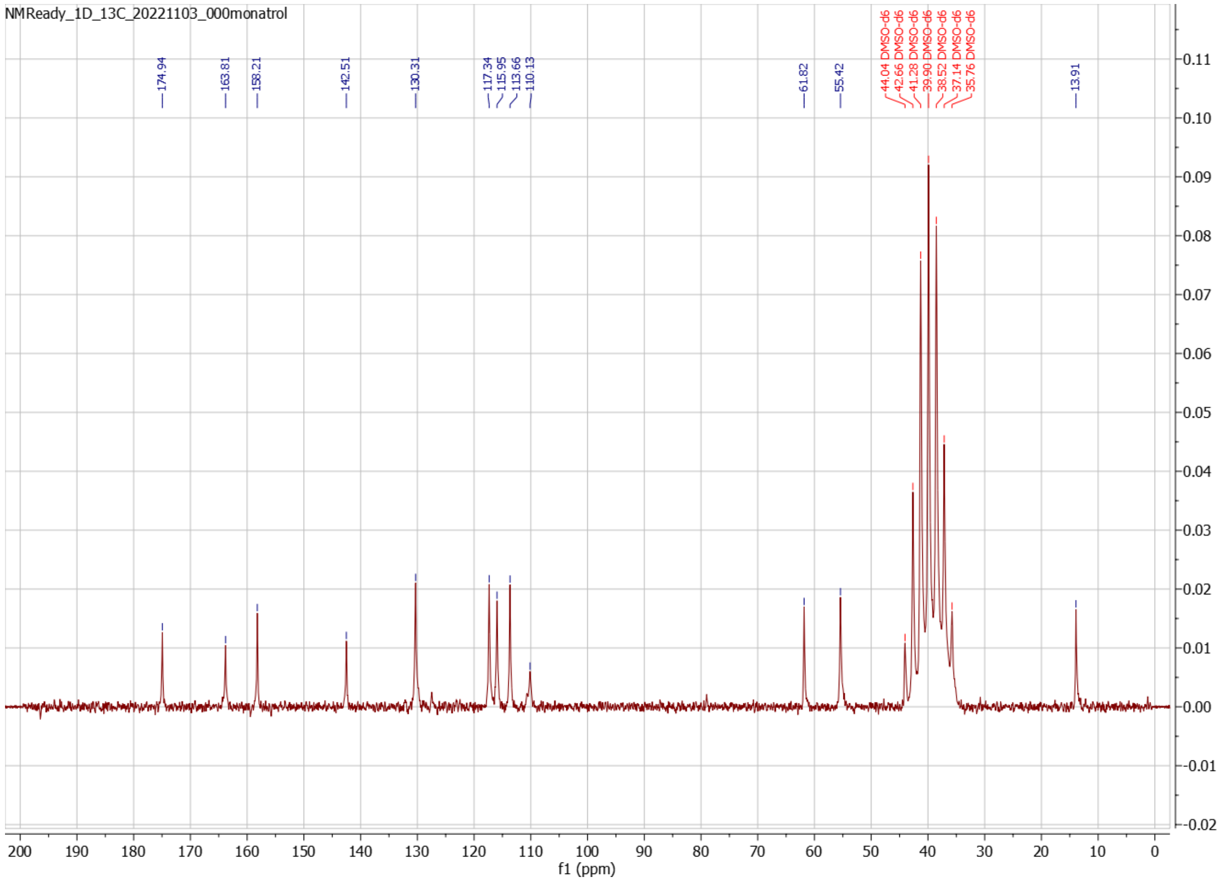

^{19}F NMR (60 MHz, DMSO-d_6) δ -62.62.

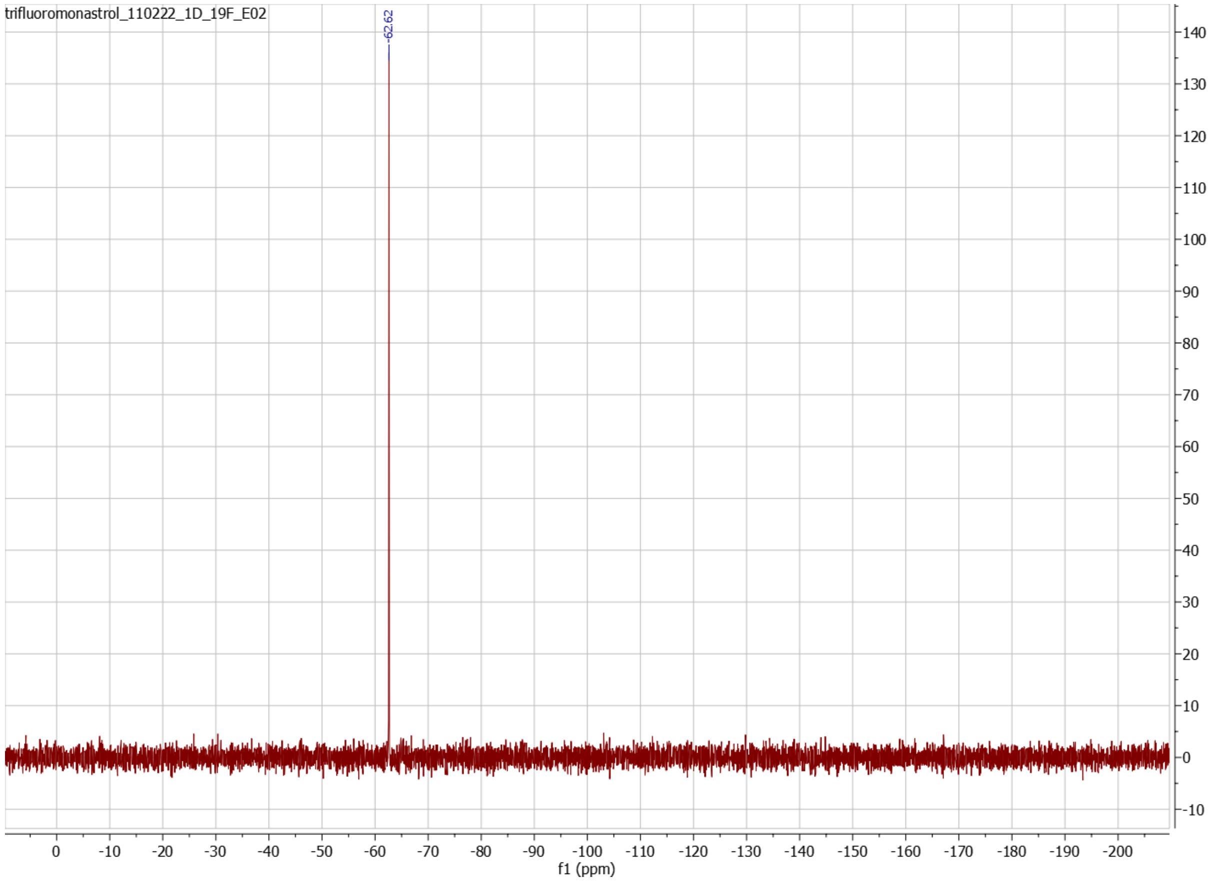

FT-IR (neat, ATR): 3280.10, 2984.61, 1705.52, 1557.90, 1458.09, 1371.47, 1310.47, 1193.47, 1153.69, 1104.45, 1051.18, 1025.86, 999.10, 859.88, 783.78, 697.78, 616.95.

COSY 2D NMR (60 MHz, DMSO-d_6)

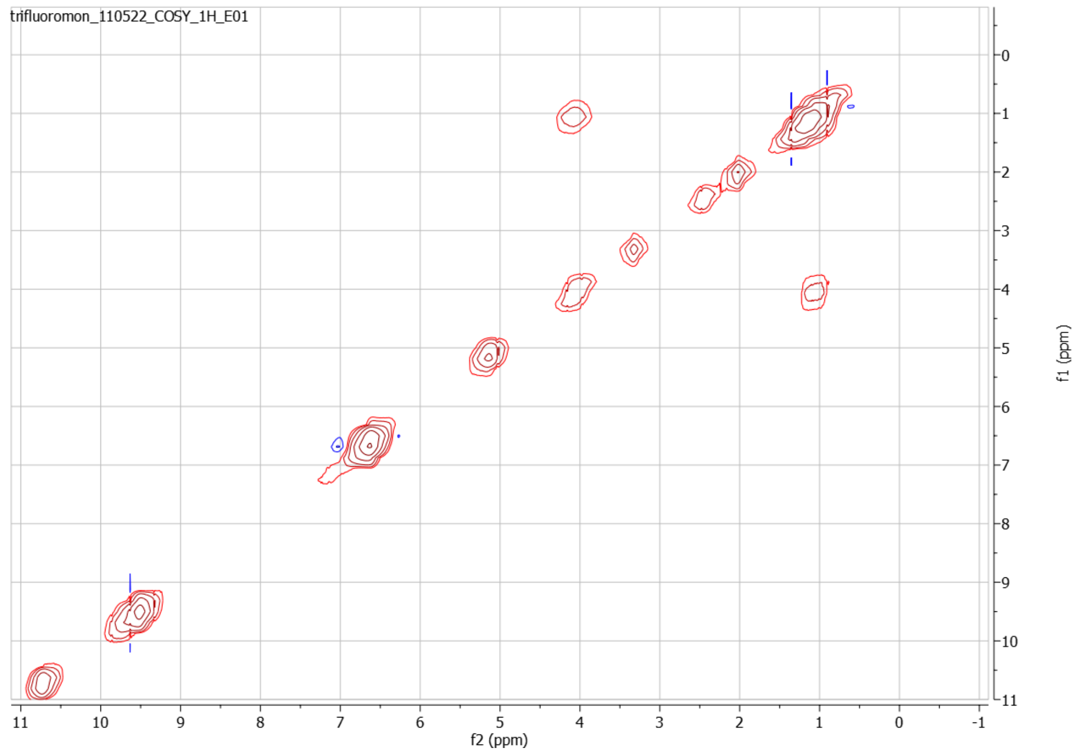

4. *In Silico* Modeling

Monastrol

C	3.455205	1.249263	-0.939237
C	4.868806	0.793438	-0.611913
O	5.087619	0.730302	0.823379
C	4.704169	-0.399253	1.453842
O	4.199456	-1.351748	0.866990
C	4.949586	-0.411182	2.902932
C	5.598114	0.554777	3.611799
C	6.132990	1.868718	3.105665
N	5.837713	0.343037	4.966723
C	5.668415	-0.853417	5.613032
S	6.209799	-1.060407	7.211374
N	5.088629	-1.821661	4.898446
C	4.406410	-1.649703	3.601376
C	2.886933	-1.625916	3.786985
C	2.116907	-2.673540	3.272638
C	0.727336	-2.681106	3.448376
O	-0.064887	-3.681122	2.960549
C	0.102163	-1.639977	4.145052
C	0.875766	-0.597596	4.656901
C	2.261222	-0.581378	4.481594
H	3.350079	1.356160	-2.024181
H	3.241447	2.217749	-0.476475
H	2.718610	0.519921	-0.591682
H	5.610220	1.513961	-0.963656
H	5.084947	-0.185508	-1.045017
H	6.904840	1.704806	2.349695
H	6.564318	2.444305	3.928021
H	5.342139	2.456864	2.636901
H	6.313288	1.064093	5.492565
H	4.955316	-2.705653	5.372954
H	4.653761	-2.525947	2.996626
H	2.595525	-3.484483	2.727825
H	0.484921	-4.335323	2.504654
H	-0.975674	-1.659369	4.274829
H	0.390175	0.210961	5.196415
H	2.846738	0.240933	4.882026

Monastrol (Hydrated)

O	2.406740	1.261832	0.560636
C	3.157423	0.412411	-0.199649
C	3.221215	0.504715	-1.593685

C	4.020000	-0.397705	-2.300319
C	4.745454	-1.384122	-1.633011
C	4.670582	-1.482590	-0.234380
C	5.495444	-2.566250	0.470198
H	6.521677	-2.460839	0.099421
N	5.562520	-2.380469	1.915867
C	4.963014	-3.141181	2.849141
N	4.187111	-4.155371	2.430601
C	3.899668	-4.533772	1.036209
O	3.934431	-5.944542	0.944234
C	2.519665	-4.013393	0.622362
C	5.041085	-4.012755	0.122714
H	4.660516	-4.016895	-0.898721
C	6.222329	-4.984954	0.102790
O	6.894158	-4.999779	1.259961
C	8.056504	-5.876081	1.352662
C	9.303535	-5.189569	0.820344
O	6.519418	-5.645584	-0.875679
S	5.182651	-2.821632	4.522389
C	3.882855	-0.578108	0.478766
H	1.958558	1.898987	-0.015168
H	2.658734	1.275559	-2.114585
H	4.079004	-0.323038	-3.382612
H	5.372762	-2.069449	-2.196999
H	6.164072	-1.647282	2.265226
H	3.736347	-4.704317	3.151081
H	3.062446	-6.293884	1.181391
H	2.465694	-2.924894	0.680866
H	1.762882	-4.429118	1.296963
H	2.293903	-4.327966	-0.400515
H	7.839783	-6.798911	0.810816
H	8.140457	-6.092175	2.419203
H	9.211012	-4.984280	-0.249716
H	10.169316	-5.842737	0.971927
H	9.485983	-4.248772	1.348586
H	3.821811	-0.619900	1.561106

Trifluoromonastrol

C	3.082661	-0.428765	1.812469
C	4.503075	-0.241172	1.305642
O	4.848221	-1.262812	0.324511
C	4.546360	-1.020503	-0.953733
O	3.930394	-0.038218	-1.342405
C	4.994392	-2.081808	-1.893069
C	6.006341	-2.962493	-1.712380

N	6.357430	-3.854435	-2.716380
C	5.906520	-3.760514	-4.009799
S	6.559238	-4.749265	-5.222059
N	4.967979	-2.834211	-4.226895
C	4.207742	-2.105722	-3.193321
C	2.802487	-2.686591	-3.031329
C	2.606155	-3.904577	-2.366674
C	1.320463	-4.443830	-2.258478
O	1.074143	-5.624973	-1.621450
C	0.223784	-3.766243	-2.811173
C	0.425978	-2.554378	-3.467533
C	1.709294	-2.009300	-3.581282
C	6.890930	-3.090934	-0.471441
F	7.925744	-3.930610	-0.724580
F	7.424887	-1.915240	-0.108483
F	6.223353	-3.604561	0.572858
H	2.882014	0.302061	2.602889
H	2.946524	-1.431515	2.228667
H	2.356817	-0.275664	1.009275
H	5.237323	-0.383077	2.100634
H	4.644422	0.741333	0.850962
H	7.120881	-4.496901	-2.553142
H	4.636737	-2.749225	-5.179677
H	4.104773	-1.075012	-3.541323
H	3.444712	-4.437870	-1.924979
H	1.905170	-5.992182	-1.285140
H	-0.767697	-4.198093	-2.714946
H	-0.424507	-2.028165	-3.891787
H	1.859259	-1.062537	-4.092449

Aryl Substituent: 3-NO2 Intermediate 12 Conformer 1

H	1.335895	-0.442883	0.066450
C	2.430925	-0.468166	0.088644
C	2.976469	0.949813	0.472821
O	3.136710	1.732553	-0.712381
C	2.129247	1.668573	1.510131
C	0.770888	1.907085	1.284554
C	0.042202	2.623604	2.233027
C	0.616327	3.113379	3.406469
C	1.970928	2.871916	3.623992
C	2.719160	2.158673	2.683956
N	-1.381986	2.867041	1.986135
O	-1.877065	2.429147	0.944790
O	-2.019550	3.499113	2.831343
C	2.817466	-1.580477	1.061456

O	3.128255	-2.698777	0.691564
O	2.751830	-1.177937	2.329306
C	3.062334	-2.159767	3.368560
C	1.830018	-2.967105	3.738287
C	2.851550	-0.860387	-1.316678
N	1.961583	-1.001514	-2.210718
C	2.113501	-1.361402	-3.545247
N	2.304182	-0.348203	-4.387586
S	1.884096	-2.966599	-4.017284
C	4.346723	-1.061840	-1.617404
F	5.113097	-0.802520	-0.539181
F	4.597653	-2.322171	-2.008119
F	4.744229	-0.238830	-2.608628
H	3.986127	0.837240	0.869452
H	2.264195	2.018792	-1.022721
H	0.264094	1.551134	0.394427
H	0.014843	3.663067	4.119280
H	2.444873	3.241198	4.527589
H	3.774985	1.978463	2.864181
H	3.415507	-1.552903	4.203654
H	3.874170	-2.795258	3.010058
H	1.016161	-2.311657	4.062256
H	2.077900	-3.642354	4.563820
H	1.486618	-3.569660	2.892845
H	2.428137	0.600501	-4.057739
H	2.294922	-0.515682	-5.384562

Aryl Substituent: 3-NO2 Intermediate 12 Conformer 2

H	1.312186	-0.339329	0.170288
C	2.406830	-0.381362	0.183267
C	2.978814	1.043707	0.493713
O	3.130881	1.768857	-0.728753
C	2.158806	1.822195	1.509608
C	0.800382	2.069924	1.294854
C	0.097749	2.840794	2.220105
C	0.698188	3.376447	3.359864
C	2.052710	3.125533	3.566734
C	2.775020	2.358110	2.649413
N	-1.326654	3.093689	1.984775
O	-1.844831	2.616513	0.972266
O	-1.941556	3.772591	2.810219
C	2.786308	-1.452965	1.203705
O	3.079143	-2.591190	0.883440
O	2.737011	-0.991499	2.452176
C	3.045124	-1.928144	3.532870

C	1.806606	-2.702424	3.949774
C	2.806598	-0.849303	-1.204910
N	1.904448	-1.029803	-2.079489
C	2.037119	-1.464434	-3.393825
N	2.249156	-0.503876	-4.291347
S	1.762771	-3.086075	-3.775813
C	4.296391	-1.077710	-1.513287
F	5.076579	-0.796890	-0.450419
F	4.528900	-2.350986	-1.871080
F	4.693579	-0.287056	-2.531050
H	3.993126	0.934415	0.879341
H	2.257778	2.055270	-1.037026
H	0.273738	1.680387	0.430745
H	0.116629	3.967882	4.055566
H	2.546790	3.529918	4.444107
H	3.831129	2.171201	2.820877
H	3.413065	-1.287948	4.336123
H	3.845986	-2.589549	3.197242
H	1.003096	-2.022704	4.248556
H	2.053202	-3.341135	4.804267
H	1.449347	-3.339851	3.136266
H	2.400773	0.457731	-4.014935
H	2.226885	-0.725031	-5.277623

Aryl Substituent: 3-NO2 Intermediate 12 Conformer 3

H	1.152486	-0.290131	0.200747
C	2.242149	-0.356838	0.292703
C	2.826361	1.062270	0.610657
O	3.102707	1.749237	-0.611463
C	1.943307	1.887900	1.532686
C	0.620542	2.176954	1.186607
C	-0.139054	2.987119	2.029674
C	0.369181	3.521966	3.213769
C	1.688282	3.229043	3.552282
C	2.466751	2.422240	2.718576
N	-1.524739	3.284125	1.655798
O	-1.961900	2.804708	0.606773
O	-2.189778	4.000557	2.407319
C	2.520047	-1.412573	1.361520
O	2.709382	-2.584682	1.089893
O	2.502648	-0.899409	2.590705
C	2.735504	-1.814126	3.708696
C	4.222183	-1.976349	3.974987
C	2.726999	-0.865111	-1.053542
N	1.894321	-0.998023	-2.002008

C	2.114862	-1.447767	-3.299724
N	2.396131	-0.494436	-4.185974
S	1.852099	-3.068753	-3.690630
C	4.220846	-1.189610	-1.225785
F	4.928307	-0.870476	-0.122045
F	4.409036	-2.496425	-1.470222
F	4.740760	-0.498739	-2.259133
H	3.800473	0.937375	1.085173
H	2.267627	2.047459	-1.002623
H	0.164498	1.790054	0.282046
H	-0.254529	4.144508	3.842589
H	2.111122	3.631071	4.467160
H	3.494340	2.202571	2.992777
H	2.255456	-2.767284	3.479636
H	2.222407	-1.338988	4.546122
H	4.724045	-2.449524	3.126451
H	4.362445	-2.612117	4.855316
H	4.692002	-1.008025	4.171687
H	2.537243	0.467203	-3.904157
H	2.448248	-0.723070	-5.169243

Aryl Substituent: 3-NO2 Intermediate 12 Conformer 4

H	1.326906	-0.250966	0.004561
C	2.419435	-0.313839	0.056093
C	3.011248	1.117499	0.296123
O	3.220456	1.759315	-0.963776
C	2.170321	1.973780	1.228786
C	2.752437	2.535850	2.366077
C	1.982733	3.362612	3.184076
C	0.647620	3.657078	2.907798
C	0.074336	3.096216	1.768395
C	0.827648	2.262194	0.938852
N	2.603089	3.944300	4.377972
O	3.783218	3.674031	4.613093
O	1.915981	4.677430	5.092880
C	2.743028	-1.327412	1.152185
O	3.023432	-2.488164	0.911737
O	2.660665	-0.790001	2.368102
C	2.913975	-1.664148	3.513391
C	1.649585	-2.396462	3.928524
C	2.853300	-0.873584	-1.287230
N	1.976254	-1.084376	-2.180477
C	2.142048	-1.598481	-3.462132
N	2.402773	-0.696173	-4.406201
S	1.845201	-3.233782	-3.758375

C	4.346363	-1.154757	-1.529126
F	5.098151	-0.819078	-0.461718
F	4.559569	-2.454322	-1.791989
F	4.795397	-0.444528	-2.583944
H	4.009004	1.013724	0.724152
H	2.363819	2.033178	-1.325047
H	3.786533	2.338147	2.620626
H	0.083209	4.303520	3.567802
H	-0.962389	3.307140	1.527685
H	0.353138	1.836126	0.058247
H	3.261266	-0.981607	4.290541
H	3.717005	-2.354536	3.248606
H	0.845460	-1.690088	4.155145
H	1.854896	-2.985112	4.828660
H	1.313686	-3.077652	3.141852
H	2.569287	0.276080	-4.180274
H	2.409416	-0.974253	-5.378170

Aryl Substituent: 3-NO2 Intermediate 12 Conformer 5

H	1.359233	-0.369236	-0.094692
C	2.452886	-0.403538	-0.040968
C	3.003463	1.033043	0.262716
O	3.210551	1.730343	-0.967539
C	2.128735	1.828645	1.217621
C	2.681806	2.360887	2.383388
C	1.880362	3.134241	3.222825
C	0.541053	3.403519	2.940987
C	-0.003351	2.872485	1.773509
C	0.782143	2.092014	0.921918
N	2.470581	3.685355	4.446158
O	3.654582	3.437352	4.686141
O	1.755501	4.371922	5.179645
C	2.801049	-1.451137	1.014624
O	3.116771	-2.592503	0.729706
O	2.696354	-0.965870	2.250832
C	2.968784	-1.878107	3.361524
C	1.722647	-2.659043	3.741696
C	2.906992	-0.891295	-1.405511
N	2.039952	-1.074617	-2.314323
C	2.222800	-1.521042	-3.618721
N	2.436148	-0.563784	-4.519523
S	1.998787	-3.152122	-3.992958
C	4.406443	-1.133280	-1.648922
F	5.147941	-0.793782	-0.575484
F	4.651269	-2.424188	-1.927158

F	4.839853	-0.399762	-2.693822
H	3.998203	0.938154	0.699497
H	2.351860	2.002025	-1.325515
H	3.717771	2.180988	2.643421
H	-0.048486	4.008425	3.618165
H	-1.042538	3.064873	1.527934
H	0.329980	1.688219	0.019298
H	3.295407	-1.218376	4.166833
H	3.790575	-2.536080	3.072983
H	0.899484	-1.983331	3.992193
H	1.940166	-3.277069	4.618993
H	1.407514	-3.317314	2.927409
H	2.561393	0.402974	-4.247907
H	2.454235	-0.795785	-5.503270

Aryl Substituent: 3-NO2 Intermediate 13 Conformer 0

C	4.339338	1.826563	1.892785
C	5.242807	1.064232	0.938417
O	5.344572	-0.341347	1.319940
C	4.418146	-1.187955	0.843449
O	3.460805	-0.855716	0.169290
C	4.755294	-2.587550	1.256962
C	3.857663	-3.579503	1.492701
C	2.392701	-3.601472	1.515165
C	1.580590	-2.481481	1.759938
C	0.200878	-2.644478	1.794658
C	-0.422984	-3.878095	1.591617
C	0.382580	-4.990483	1.362282
C	1.770783	-4.856217	1.339940
N	-0.633254	-1.466578	2.061202
O	-0.072454	-0.386570	2.257979
O	-1.856933	-1.614329	2.076013
C	6.193530	-2.921922	1.410778
N	6.568308	-3.692268	2.357565
C	7.829943	-4.211483	2.607799
N	8.691940	-3.389309	3.204287
S	8.129651	-5.843650	2.288846
C	7.195747	-2.384773	0.367628
F	6.574967	-1.871907	-0.710303
F	7.981115	-1.425383	0.897123
F	8.000094	-3.374652	-0.061887
H	4.349272	2.888320	1.625495
H	4.689699	1.727789	2.924509
H	3.308550	1.466996	1.830810
H	6.272945	1.421371	0.983515

H	4.883531	1.117201	-0.091322
H	4.302917	-4.550161	1.701274
H	1.995995	-1.500548	1.931445
H	-1.502441	-3.954439	1.624249
H	-0.071282	-5.964209	1.211844
H	2.390110	-5.733611	1.178114
H	8.460042	-2.422920	3.395354
H	9.578837	-3.745465	3.534540

Aryl Substituent: 3-NO2 Intermediate 13 Conformer 1

C	4.682392	1.994073	1.821136
C	5.566601	1.183149	0.889204
O	5.526718	-0.235931	1.228398
C	4.569745	-0.989843	0.663941
O	3.685509	-0.560794	-0.053808
C	4.774991	-2.425118	1.039606
C	3.794534	-3.357155	1.157234
C	2.334651	-3.261843	1.063672
C	1.651108	-4.439571	0.715962
C	0.262871	-4.423997	0.624132
C	-0.489382	-3.281532	0.897427
C	0.188276	-2.122812	1.275864
C	1.579588	-2.107200	1.357013
N	-0.431586	-5.657679	0.243286
O	0.246701	-6.660905	0.012450
O	-1.661894	-5.632239	0.171100
C	6.169863	-2.871766	1.282780
N	6.415438	-3.698999	2.223568
C	7.612182	-4.323263	2.542578
N	8.489339	-3.590997	3.227590
S	7.808581	-5.963275	2.185584
C	7.283270	-2.380311	0.334280
F	6.783322	-1.786648	-0.764715
F	8.097336	-1.502341	0.953764
F	8.040543	-3.415389	-0.074153
H	4.797831	3.058081	1.589862
H	4.967588	1.835865	2.865477
H	3.629452	1.727498	1.695272
H	6.619756	1.447470	0.998259
H	5.268704	1.296866	-0.155120
H	4.150212	-4.367712	1.347412
H	2.192649	-5.355458	0.512207
H	-1.569352	-3.309425	0.825559
H	-0.374649	-1.226252	1.513148
H	2.076198	-1.197330	1.666307

H	8.319709	-2.615581	3.437390
H	9.318304	-4.027736	3.607836

Aryl Substituent: 3-NO2 Intermediate 13 Conformer 2

C	4.399467	1.805157	1.910380
C	5.276269	1.047491	0.927882
O	5.368923	-0.365027	1.285490
C	4.424604	-1.193745	0.813042
O	3.458454	-0.840117	0.162571
C	4.754216	-2.603448	1.196747
C	3.850711	-3.590352	1.431492
C	2.386301	-3.600602	1.478276
C	1.586572	-2.477512	1.748364
C	0.206438	-2.631174	1.804397
C	-0.429887	-3.857876	1.598475
C	0.363594	-4.973521	1.343934
C	1.752084	-4.848927	1.300134
N	-0.614522	-1.450297	2.097448
O	-0.042663	-0.376569	2.296403
O	-1.838858	-1.589340	2.130343
C	6.191233	-2.955013	1.319071
N	6.574427	-3.746694	2.244501
C	7.833259	-4.285225	2.463539
N	8.715544	-3.484657	3.059874
S	8.107895	-5.914846	2.109908
C	7.181084	-2.410048	0.268140
F	6.546950	-1.874269	-0.790814
F	7.984255	-1.466814	0.799414
F	7.968590	-3.400325	-0.190789
H	4.417368	2.870789	1.659395
H	4.767488	1.686183	2.933791
H	3.363529	1.458679	1.861797
H	6.311179	1.392033	0.959461
H	4.898868	1.120651	-0.094126
H	4.290410	-4.568247	1.616494
H	2.011467	-1.501173	1.922637
H	-1.509194	-3.926609	1.648209
H	-0.099761	-5.942341	1.190875
H	2.362216	-5.729041	1.119053
H	8.498528	-2.519635	3.273923
H	9.602563	-3.858342	3.369772

Aryl Substituent: 3-NO2 Intermediate 13 Conformer 3

C	4.933040	1.998032	1.873243
C	5.764950	1.170142	0.908643
O	5.688781	-0.250516	1.234453

C	4.694048	-0.967837	0.687586
O	3.805882	-0.503174	-0.002588
C	4.863481	-2.412648	1.043570
C	3.859174	-3.317761	1.170586
C	2.400902	-3.181689	1.108506
C	1.678150	-4.338055	0.768266
C	0.289191	-4.284248	0.706565
C	-0.425647	-3.123666	1.002799
C	0.291235	-1.986234	1.373467
C	1.683863	-2.008914	1.424851
N	-0.446603	-5.496192	0.333577
O	0.199081	-6.515703	0.081587
O	-1.677089	-5.437353	0.289318
C	6.249430	-2.901972	1.254393
N	6.490217	-3.740797	2.186019
C	7.675028	-4.401492	2.476067
N	8.587476	-3.698712	3.145582
S	7.815237	-6.044443	2.106745
C	7.355620	-2.440300	0.282789
F	6.850128	-1.819574	-0.798412
F	8.215054	-1.596840	0.889032
F	8.065796	-3.497539	-0.152795
H	5.076424	3.060454	1.650418
H	5.241258	1.818487	2.907543
H	3.869006	1.766735	1.773654
H	6.828603	1.399854	0.990957
H	5.442221	1.304902	-0.125750
H	4.189815	-4.340020	1.342611
H	2.189827	-5.267052	0.547490
H	-1.507265	-3.121707	0.954004
H	-0.241681	-1.076165	1.628000
H	2.211561	-1.114960	1.728781
H	8.450808	-2.720009	3.363967
H	9.411196	-4.161436	3.505974

Aryl Substituent: 3-NO2 Intermediate 13 Conformer 4

C	6.277822	1.367530	-0.503787
C	5.478880	1.133154	0.767818
O	5.470873	-0.282966	1.124281
C	4.518258	-1.061556	0.586456
O	3.618903	-0.661932	-0.129104
C	4.738316	-2.484853	1.001530
C	3.760924	-3.410287	1.177484
C	2.299291	-3.308005	1.122896
C	1.597510	-4.484947	0.811819

C	0.207266	-4.458951	0.759119
C	-0.527983	-3.306622	1.037506
C	0.169146	-2.148367	1.380214
C	1.562385	-2.143212	1.421609
N	-0.507371	-5.691840	0.414974
O	0.156107	-6.703771	0.179018
O	-1.739033	-5.657212	0.376726
C	6.139165	-2.924383	1.221269
N	6.411722	-3.716673	2.184179
C	7.617712	-4.329715	2.490948
N	8.511980	-3.573717	3.126108
S	7.805544	-5.980668	2.184388
C	7.222265	-2.470827	0.220479
F	6.684421	-1.922695	-0.884954
F	8.052273	-1.564763	0.776862
F	7.970385	-3.518702	-0.169532
H	6.339265	2.443615	-0.695728
H	5.799199	0.891337	-1.363891
H	7.292877	0.974188	-0.401382
H	4.445467	1.470476	0.669885
H	5.939920	1.618109	1.629766
H	4.118272	-4.415941	1.389455
H	2.125907	-5.407967	0.605807
H	-1.609747	-3.326625	0.996889
H	-0.379685	-1.244092	1.621311
H	2.075175	-1.233742	1.705552
H	8.344781	-2.592639	3.309940
H	9.351907	-3.996439	3.498091

Aryl Substituent: 3-NO2 Intermediate 14 Conformer 1

C	1.983671	-0.319815	1.166553
C	2.445043	0.095316	-0.219578
O	3.133424	1.386684	-0.173743
C	4.436797	1.377720	0.098122
O	5.119620	0.386597	0.275446
C	4.985636	2.807795	0.160451
C	4.342926	3.577960	1.354811
O	4.505761	2.852756	2.558548
C	4.841630	5.022227	1.446856
C	5.345012	5.529979	2.651848
C	5.781598	6.854727	2.749877
C	5.717812	7.702034	1.647385
C	5.203112	7.186694	0.456958
C	4.764856	5.868805	0.336385
N	5.116012	8.063390	-0.714377

O	5.510086	9.226577	-0.601960
O	4.653248	7.599169	-1.759431
C	6.497787	2.727547	0.293289
O	7.090981	2.687241	1.348782
C	7.296498	2.740439	-1.030845
F	6.798827	1.832827	-1.891609
F	7.171741	3.961191	-1.593757
F	8.590425	2.487325	-0.832105
H	1.416302	-1.253256	1.091491
H	1.334972	0.443267	1.606738
H	2.835696	-0.488235	1.831035
H	1.604490	0.271611	-0.892364
H	3.120614	-0.638071	-0.663509
H	4.718874	3.301534	-0.779079
H	3.265510	3.599183	1.162688
H	5.458763	2.704156	2.680905
H	5.385669	4.883759	3.521470
H	6.171473	7.226266	3.692057
H	6.049950	8.731049	1.698807
H	4.363283	5.533651	-0.612446

Aryl Substituent: 3-NO2 Intermediate 14 Conformer 2

C	2.010501	-0.379364	1.207269
C	2.513055	-0.027427	-0.182005
O	3.151670	1.290062	-0.186405
C	4.441483	1.347513	0.139195
O	5.151239	0.395672	0.404217
C	4.934366	2.799173	0.139969
C	4.225232	3.604123	1.273089
O	4.382647	2.953192	2.519171
C	4.660026	5.070578	1.296201
C	4.557137	5.869290	0.145268
C	4.933375	7.213936	0.166271
C	5.417808	7.791745	1.338572
C	5.503538	6.989361	2.475129
C	5.131591	5.644505	2.476486
N	5.999700	7.580112	3.721449
O	6.334766	8.767165	3.710986
O	6.059924	6.864513	4.724283
C	6.443582	2.784129	0.319718
O	7.005915	2.830428	1.391696
C	7.281014	2.745095	-0.979961
F	6.834396	1.776120	-1.800985
F	7.139776	3.927800	-1.615701
F	8.574741	2.541457	-0.730383

H	1.479435	-1.335944	1.165156
H	1.318243	0.383049	1.576557
H	2.840029	-0.477923	1.912955
H	1.694939	0.077086	-0.896151
H	3.232511	-0.758552	-0.555082
H	4.678116	3.234063	-0.830974
H	3.154116	3.568555	1.049897
H	5.336883	2.846504	2.671907
H	4.169855	5.447628	-0.778085
H	4.846689	7.813842	-0.733747
H	5.717027	8.831379	1.378655
H	5.199975	5.064033	3.386484

Aryl Substituent: 3-NO2 Intermediate 14 Conformer 3

C	1.834363	-0.199898	0.906316
C	2.516860	0.136910	-0.407956
O	3.247311	1.401525	-0.305755
C	4.493974	1.354125	0.159796
O	5.097661	0.346016	0.474770
C	5.089112	2.763656	0.253800
C	4.320761	3.600085	1.323997
O	4.292888	2.918333	2.562851
C	4.857896	5.029280	1.429575
C	5.255664	5.558284	2.664268
C	5.728696	6.870266	2.767250
C	5.806844	7.682777	1.639877
C	5.395276	7.146933	0.418666
C	4.923579	5.841348	0.292874
N	5.456889	7.988066	-0.780230
O	5.877778	9.141110	-0.661517
O	5.085463	7.505316	-1.852925
C	6.564035	2.631401	0.596748
O	7.005448	2.605809	1.724604
C	7.535362	2.569186	-0.604951
F	7.117555	1.656867	-1.502756
F	7.544241	3.776042	-1.210158
F	8.777230	2.268320	-0.224823
H	1.245600	-1.114490	0.780918
H	1.159829	0.604777	1.213485
H	2.568502	-0.370165	1.698741
H	1.795691	0.316190	-1.206724
H	3.217989	-0.640577	-0.716600
H	4.974551	3.235407	-0.726902
H	3.280780	3.649300	0.986038
H	5.213919	2.734305	2.813710

H	5.183892	4.940787	3.552538
H	6.035242	7.259309	3.732845
H	6.169439	8.701273	1.695305
H	4.606282	5.487826	-0.681073

Aryl Substituent: 3-NO2 Intermediate 14 Conformer 4

C	2.136406	-0.401539	1.438052
C	2.443505	-0.007144	0.003928
O	3.077838	1.311300	-0.048094
C	4.400841	1.361287	0.093891
O	5.138931	0.402970	0.223752
C	4.892389	2.813254	0.074541
C	4.348918	3.577957	1.321044
O	4.670911	2.882764	2.510372
C	4.790369	5.042647	1.337792
C	4.550286	5.880508	0.236231
C	4.931619	7.223775	0.256834
C	5.558384	7.761221	1.379789
C	5.780088	6.920115	2.468998
C	5.405952	5.575945	2.469944
N	6.427774	7.467621	3.664587
O	6.762042	8.654883	3.654406
O	6.609918	6.717222	4.626375
C	6.412046	2.795403	0.042984
O	7.117136	2.807180	1.027866
C	7.061012	2.801335	-1.360906
F	6.505693	1.856166	-2.142437
F	6.831632	4.002672	-1.933128
F	8.377070	2.596969	-1.300052
H	1.606719	-1.359802	1.441210
H	1.499653	0.345786	1.920662
H	3.055127	-0.514974	2.020288
H	1.535126	0.116502	-0.587517
H	3.103553	-0.725154	-0.486058
H	4.505312	3.279367	-0.836609
H	3.257149	3.550655	1.245188
H	5.636732	2.773131	2.530017
H	4.051803	5.490651	-0.646735
H	4.737619	7.854345	-0.604667
H	5.864193	8.798987	1.418151
H	5.584054	4.964597	3.344100

Aryl Substituent: 3-NO2 Intermediate 14 Conformer 5

C	2.506994	-0.479412	-1.661578
C	2.361320	0.132135	-0.279076
O	3.066246	1.412611	-0.197294

C	4.360043	1.381050	0.117207
O	5.014528	0.378870	0.333168
C	4.934366	2.800960	0.183948
C	4.285362	3.591955	1.361742
O	4.420218	2.876685	2.574759
C	4.804665	5.029365	1.445661
C	5.313452	5.538515	2.647600
C	5.770047	6.857118	2.735805
C	5.721257	7.696559	1.626492
C	5.200879	7.180186	0.439232
C	4.742717	5.868546	0.328597
N	5.129631	8.049061	-0.740261
O	5.537671	9.207556	-0.633895
O	4.665669	7.580801	-1.782548
C	6.442346	2.693459	0.344239
O	7.016107	2.652519	1.410267
C	7.262838	2.677284	-0.966520
F	6.756947	1.774429	-1.828119
F	7.174620	3.896077	-1.540686
F	8.546709	2.395855	-0.745606
H	1.911065	-1.396513	-1.712485
H	3.549186	-0.735818	-1.870798
H	2.147296	0.208268	-2.432618
H	2.749803	-0.524838	0.501276
H	1.325031	0.390319	-0.056206
H	4.694010	3.294520	-0.762648
H	3.211177	3.627395	1.153664
H	5.368651	2.709658	2.708128
H	5.342252	4.899185	3.522715
H	6.164243	7.230030	3.675591
H	6.069198	8.720687	1.670090
H	4.337156	5.532822	-0.618445

Aryl Substituent: 3-NO2 Intermediate 15 Conformer 0

C	2.237677	1.322962	0.072595
C	2.931799	0.042041	0.487884
O	3.496750	-0.558533	-0.718817
C	4.236742	-1.656750	-0.550841
O	4.499041	-2.160923	0.526119
C	4.704486	-2.218908	-1.865977
C	3.867189	-2.729400	-2.805049
C	2.414992	-2.881922	-2.774370
C	1.735151	-2.995985	-4.005579
C	0.347771	-3.126432	-4.055063
C	-0.394235	-3.173327	-2.876363

C	0.289544	-3.090020	-1.661369
C	1.669886	-2.951971	-1.584914
N	-0.474082	-3.155937	-0.409685
O	-1.690500	-3.334264	-0.481649
O	0.138526	-3.024150	0.652510
C	6.154947	-2.256008	-2.177175
O	6.624825	-2.740341	-3.191116
C	7.150291	-1.637937	-1.157362
F	6.691967	-0.461571	-0.675361
F	7.338492	-2.472679	-0.118835
F	8.334707	-1.415539	-1.736818
H	1.794632	1.793668	0.955538
H	1.439313	1.122305	-0.648090
H	2.947084	2.025980	-0.373922
H	2.237171	-0.678489	0.931170
H	3.744755	0.219731	1.196338
H	4.348472	-3.074538	-3.718302
H	2.306310	-2.969765	-4.928793
H	-0.156826	-3.199308	-5.012465
H	-1.471607	-3.280230	-2.887621
H	2.140727	-2.948760	-0.611615

Aryl Substituent: 3-NO2 Intermediate 15 Conformer 1

C	4.029366	1.259524	1.073395
C	2.972464	0.290581	0.570013
O	3.419308	-0.368581	-0.656907
C	4.147179	-1.481214	-0.525412
O	4.457153	-1.998465	0.532550
C	4.552047	-2.036748	-1.863754
C	3.677951	-2.517588	-2.784406
C	2.225572	-2.655141	-2.713265
C	1.499997	-2.761307	-1.507401
C	0.113160	-2.894792	-1.516954
C	-0.586748	-2.925573	-2.723188
C	0.139972	-2.841157	-3.911258
C	1.525404	-2.723215	-3.930088
N	-0.579497	-2.893971	-5.189019
O	-1.806598	-3.001211	-5.158543
O	0.077559	-2.827764	-6.229461
C	5.990379	-2.086224	-2.226904
O	6.419289	-2.572537	-3.257936
C	7.029995	-1.478026	-1.244880
F	6.599967	-0.299913	-0.739383
F	7.256270	-2.317772	-0.218367
F	8.190717	-1.259673	-1.871784

H	3.647964	1.780862	1.957369
H	4.269260	2.005095	0.309566
H	4.945126	0.731586	1.352432
H	2.057339	0.805738	0.273756
H	2.736372	-0.475017	1.311641
H	4.127105	-2.850582	-3.718394
H	2.020176	-2.785437	-0.556877
H	-0.426845	-2.983722	-0.580388
H	-1.664442	-3.026427	-2.749808
H	2.050812	-2.673809	-4.876124

Aryl Substituent: 3-NO2 Intermediate 15 Conformer 2

C	2.832585	1.852340	-2.267043
C	3.028400	0.949776	-1.066168
O	3.647436	-0.284541	-1.545137
C	3.994288	-1.179970	-0.617969
O	3.867068	-1.023401	0.582959
C	4.555146	-2.432001	-1.236038
C	3.829741	-3.275238	-2.014715
C	2.422375	-3.208445	-2.399468
C	2.018501	-3.906149	-3.557523
C	0.694942	-3.874137	-3.995096
C	-0.265135	-3.167647	-3.273053
C	0.137261	-2.504685	-2.111476
C	1.450790	-2.513766	-1.658987
N	-0.864014	-1.771405	-1.327999
O	-2.037575	-1.818088	-1.698482
O	-0.480959	-1.140924	-0.339695
C	5.967017	-2.810729	-0.980985
O	6.485758	-3.833352	-1.391575
C	6.854985	-1.850486	-0.142999
F	6.654147	-0.561797	-0.496349
F	6.575032	-1.975104	1.167306
F	8.149201	-2.133646	-0.324376
H	2.362410	2.785457	-1.941907
H	2.184727	1.381399	-3.012381
H	3.791153	2.094106	-2.735639
H	2.079433	0.688405	-0.587256
H	3.684668	1.394232	-0.313753
H	4.378055	-4.126621	-2.413865
H	2.758002	-4.467308	-4.120908
H	0.407678	-4.406149	-4.895638
H	-1.300457	-3.133077	-3.588129
H	1.683339	-2.024947	-0.723056

Aryl Substituent: 3-NO2 Intermediate 15 Conformer 3

C	3.648326	1.159770	0.944260
C	2.694952	0.119233	0.381165
O	3.270409	-0.525455	-0.799607
C	4.069060	-1.576832	-0.595836
O	4.350805	-2.045897	0.492063
C	4.590401	-2.134559	-1.892547
C	3.809294	-2.722946	-2.834057
C	2.372000	-2.981206	-2.829020
C	1.733358	-3.177612	-4.072031
C	0.361529	-3.413613	-4.150300
C	-0.403486	-3.488380	-2.987906
C	0.241852	-3.324720	-1.760130
C	1.605042	-3.076876	-1.655207
N	-0.545644	-3.425302	-0.525413
O	-1.750097	-3.663966	-0.624476
O	0.035572	-3.261957	0.549244
C	6.044665	-2.069850	-2.181496
O	6.566786	-2.541233	-3.175837
C	6.974939	-1.354585	-1.162993
F	6.421257	-0.205423	-0.713939
F	7.211307	-2.148734	-0.103052
F	8.146892	-1.053974	-1.732032
H	3.171146	1.664676	1.790427
H	3.894554	1.911056	0.188086
H	4.573313	0.695468	1.296030
H	1.773657	0.571017	0.010180
H	2.452799	-0.651514	1.115768
H	4.332148	-3.050267	-3.730911
H	2.324160	-3.132513	-4.982111
H	-0.112056	-3.548715	-5.116764
H	-1.468811	-3.678730	-3.020944
H	2.048771	-3.008763	-0.671615

Aryl Substituent: 3-NO2 Intermediate 15 Conformer 4

C	2.114220	1.175003	0.195581
C	2.827327	-0.107559	0.571879
O	3.430865	-0.643859	-0.646355
C	4.188697	-1.734123	-0.509523
O	4.436203	-2.280980	0.550004
C	4.698527	-2.227343	-1.836590
C	3.894127	-2.708058	-2.819125
C	2.444567	-2.885894	-2.832664
C	1.797324	-2.952598	-4.084751
C	0.414171	-3.103797	-4.175579
C	-0.355562	-3.219732	-3.019621

C	0.296604	-3.183106	-1.785090
C	1.671898	-3.025015	-1.667134
N	-0.496103	-3.323477	-0.557844
O	-1.706549	-3.521185	-0.669291
O	0.087493	-3.231929	0.524715
C	6.156631	-2.225274	-2.111885
O	6.660953	-2.659998	-3.131758
C	7.114091	-1.629229	-1.043926
F	6.619014	-0.484347	-0.524092
F	7.296706	-2.503022	-0.036945
F	8.306249	-1.357368	-1.585070
H	1.644043	1.599432	1.087899
H	1.334628	0.988140	-0.548965
H	2.817065	1.910372	-0.207068
H	2.139543	-0.860739	0.969155
H	3.621037	0.056870	1.304970
H	4.404127	-3.002402	-3.734578
H	2.390597	-2.873214	-4.990848
H	-0.065383	-3.139235	-5.147852
H	-1.430213	-3.344341	-3.063312
H	2.118202	-3.059792	-0.682937

Aryl Substituent: 3-NO2 Intermediate 16 Conformer 1

C	4.491477	2.200246	-0.739388
C	3.361529	1.957689	0.246248
O	3.865456	1.364021	1.487233
C	3.988119	0.038698	1.530857
O	3.703514	-0.733029	0.636622
C	4.571403	-0.392468	2.884412
C	4.233304	-1.881319	3.189837
N	5.070826	-2.312323	4.310820
C	5.374795	-3.598302	4.633009
N	4.954329	-4.570617	3.813070
S	6.292562	-3.935320	6.034946
C	2.738849	-2.075000	3.447919
C	1.914528	-2.633133	2.460611
C	0.546913	-2.814998	2.686850
C	-0.019936	-2.440627	3.902007
C	0.814169	-1.886334	4.874291
C	2.179459	-1.698088	4.671664
N	0.234976	-1.489420	6.161731
O	-0.974724	-1.655279	6.330600
O	0.984122	-1.007949	7.014775
C	6.081493	-0.123604	2.947958
O	6.598783	0.483634	3.854816

C	6.978854	-0.643523	1.791122
F	6.722623	-1.939404	1.519975
F	6.727795	0.072621	0.674243
F	8.269813	-0.518691	2.095371
H	4.091518	2.714810	-1.619078
H	4.940597	1.258978	-1.066370
H	5.268221	2.829514	-0.295222
H	2.594908	1.297110	-0.162689
H	2.901665	2.890931	0.574045
H	4.145567	0.250131	3.659914
H	4.513379	-2.458596	2.306182
H	5.305776	-1.637993	5.027938
H	4.346876	-4.414642	3.021564
H	5.194486	-5.525613	4.032407
H	2.343398	-2.921215	1.506107
H	-0.075856	-3.252439	1.913618
H	-1.075702	-2.573312	4.101356
H	2.781633	-1.274148	5.466232

Aryl Substituent: 3-NO2 Intermediate 16 Conformer 2

C	5.494895	0.469562	-1.609727
C	4.671497	1.265593	-0.611881
O	4.960676	0.824623	0.755043
C	4.268222	-0.211813	1.239852
O	3.375542	-0.797433	0.665996
C	4.787339	-0.550837	2.641767
C	4.338325	-1.968680	3.101154
N	5.223471	-2.375297	4.200029
C	5.467705	-3.652007	4.606345
N	4.981766	-4.657180	3.867022
S	6.399570	-3.935723	6.010019
C	2.861301	-2.020642	3.487350
C	2.407949	-1.398208	4.660325
C	1.062517	-1.456081	5.028701
C	0.144996	-2.141011	4.233845
C	0.611405	-2.756866	3.073190
C	1.950511	-2.708248	2.684874
N	-0.338189	-3.484390	2.226500
O	-1.521133	-3.515754	2.572702
O	0.089532	-4.031315	1.207245
C	6.307507	-0.376947	2.692835
O	6.872318	0.363315	3.463154
C	7.189481	-1.201481	1.712103
F	6.441793	-1.931839	0.853828
F	7.971968	-0.383219	0.993545

F	7.973010	-2.043596	2.402256
H	5.337387	0.876972	-2.613393
H	5.198402	-0.583017	-1.615203
H	6.560329	0.535416	-1.371126
H	3.598907	1.164728	-0.785735
H	4.943587	2.321837	-0.608355
H	4.395023	0.204801	3.331718
H	4.507326	-2.646289	2.260893
H	5.488395	-1.674127	4.880655
H	4.375796	-4.528387	3.069758
H	5.186110	-5.605218	4.145402
H	3.107901	-0.866619	5.298647
H	0.728493	-0.968282	5.938403
H	-0.902622	-2.201481	4.500136
H	2.261395	-3.191358	1.767127

Aryl Substituent: 3-NO2 Intermediate 16 Conformer 3

C	4.334535	2.164916	-0.620694
C	3.262738	1.945308	0.432979
O	3.827343	1.329806	1.636802
C	3.922267	0.001600	1.663336
O	3.563275	-0.755614	0.783532
C	4.579077	-0.453761	2.974894
C	4.278140	-1.953807	3.259576
N	5.195238	-2.407473	4.307431
C	5.557378	-3.695859	4.550888
N	5.133217	-4.643809	3.705086
S	6.550506	-4.065535	5.892550
C	2.808246	-2.173852	3.615379
C	2.315437	-1.823005	4.881856
C	0.972765	-2.022058	5.207941
C	0.097099	-2.577080	4.275802
C	0.601946	-2.921557	3.022978
C	1.939582	-2.730102	2.675158
N	-0.302960	-3.509825	2.031114
O	-1.484324	-3.671364	2.344715
O	0.159018	-3.814897	0.929195
C	6.085919	-0.159638	2.966709
O	6.634314	0.467219	3.841156
C	6.937161	-0.673372	1.772302
F	6.698655	-1.977367	1.524733
F	6.615036	0.024863	0.661921
F	8.238215	-0.516216	2.011370
H	3.894724	2.699213	-1.469072
H	4.734599	1.214507	-0.982978

H	5.155836	2.767413	-0.221788
H	2.454379	1.308085	0.069815
H	2.849639	2.888065	0.794162
H	4.184458	0.164080	3.786220
H	4.503678	-2.501111	2.342587
H	5.433919	-1.763204	5.050565
H	4.494083	-4.472573	2.942243
H	5.422879	-5.597413	3.862333
H	2.983683	-1.398307	5.625428
H	0.607556	-1.746484	6.191693
H	-0.947464	-2.742657	4.507403
H	2.283905	-3.003434	1.685781

Aryl Substituent: 3-NO2 Intermediate 16 Conformer 4

C	4.538168	2.255235	-0.497094
C	3.406832	2.056101	0.496697
O	3.879929	1.370130	1.701923
C	3.907551	0.038647	1.682535
O	3.555899	-0.667412	0.758412
C	4.475886	-0.495873	3.005487
C	4.087182	-1.987180	3.220769
N	4.924748	-2.520174	4.297444
C	5.211654	-3.832371	4.512524
N	4.790626	-4.729012	3.610966
S	6.108984	-4.296311	5.891727
C	2.591066	-2.145896	3.490841
C	1.736597	-2.631055	2.490878
C	0.367061	-2.781312	2.728391
C	-0.171612	-2.447779	3.968035
C	0.692428	-1.966189	4.952855
C	2.060224	-1.810594	4.739183
N	0.143238	-1.612974	6.265837
O	-1.068482	-1.751717	6.445455
O	0.917955	-1.193120	7.128440
C	5.994217	-0.278655	3.078033
O	6.529202	0.296151	3.995358
C	6.876524	-0.801176	1.910318
F	6.593743	-2.086993	1.618434
F	6.635899	-0.062893	0.805036
F	8.170820	-0.707498	2.211154
H	4.166866	2.838167	-1.346199
H	4.908292	1.298012	-0.872990
H	5.367599	2.802265	-0.039452
H	2.585341	1.475759	0.072769
H	3.025514	3.006257	0.873109

H	4.073633	0.113035	3.819856
H	4.333502	-2.515167	2.297824
H	5.153986	-1.913486	5.074444
H	4.213160	-4.498884	2.815168
H	5.028336	-5.700097	3.746660
H	2.143916	-2.886369	1.517788
H	-0.279426	-3.162069	1.944605
H	-1.228372	-2.556797	4.176422
H	2.685613	-1.442208	5.543559

Aryl Substituent: 3-NO2 Intermediate 16 Conformer 5

C	2.014558	1.440628	-0.281105
C	3.531794	1.390600	-0.234868
O	4.000834	0.964630	1.085791
C	4.097677	-0.344731	1.311282
O	3.819126	-1.224887	0.521467
C	4.651902	-0.595985	2.720583
C	4.320163	-2.037634	3.205517
N	5.125840	-2.305544	4.398569
C	5.427661	-3.532359	4.900703
N	5.035837	-4.611429	4.210063
S	6.309724	-3.667364	6.359177
C	2.820269	-2.217326	3.443075
C	2.034819	-2.925416	2.522426
C	0.663066	-3.096253	2.732170
C	0.053275	-2.560793	3.863363
C	0.849105	-1.858488	4.769873
C	2.217713	-1.678324	4.582837
N	0.224477	-1.290002	5.968757
O	-0.988281	-1.448769	6.122879
O	0.940358	-0.679461	6.765686
C	6.157388	-0.300124	2.781736
O	6.648411	0.420522	3.617180
C	7.083377	-0.949337	1.716695
F	6.873158	-2.279674	1.640308
F	6.818329	-0.416620	0.505056
F	8.366362	-0.736140	2.004761
H	1.699127	1.834985	-1.252536
H	1.622171	2.096922	0.501243
H	1.582920	0.443352	-0.159400
H	3.974658	2.379572	-0.360370
H	3.942025	0.708706	-0.982213
H	4.202091	0.133988	3.399293
H	4.633620	-2.722146	2.414431
H	5.338051	-1.537114	5.021782

H	4.438545	-4.570388	3.396706
H	5.267494	-5.525219	4.569493
H	2.497588	-3.340211	1.632585
H	0.070591	-3.650751	2.011964
H	-1.006538	-2.681123	4.048629
H	2.789215	-1.134192	5.325112

Aryl Substituent: 3-NO2 Intermediate 2 Conformer 0

H	1.110031	-0.407894	-0.404311
C	2.028918	-0.072868	0.116074
O	2.285817	1.114073	0.239678
C	2.887049	-1.165526	0.630072
C	2.490721	-2.490818	0.429918
C	3.307545	-3.507712	0.918098
C	4.499420	-3.244559	1.595614
C	4.883632	-1.915844	1.789377
C	4.083880	-0.880691	1.310211
N	2.900709	-4.901603	0.712690
O	1.843203	-5.115410	0.116622
O	3.635446	-5.791291	1.145600
H	1.570809	-2.728819	-0.092077
H	5.106016	-4.064580	1.959495
H	5.807397	-1.696783	2.314439
H	4.370406	0.156326	1.453500

Aryl Substituent: 3-NO2 Intermediate 2 Conformer 1

H	1.109971	-0.407892	-0.404212
C	2.028835	-0.072867	0.116213
O	2.285678	1.114076	0.239913
C	2.886993	-1.165526	0.630166
C	2.490738	-2.490822	0.429891
C	3.307598	-3.507714	0.918011
C	4.499432	-3.244556	1.595597
C	4.883564	-1.915838	1.789498
C	4.083780	-0.880687	1.310381
N	2.900851	-4.901610	0.712457
O	1.843239	-5.115417	0.116577
O	3.635769	-5.791301	1.145053
H	1.570857	-2.728828	-0.092157
H	5.106059	-4.064579	1.959424
H	5.807290	-1.696773	2.314625
H	4.370246	0.156334	1.453763

Aryl Substituent: 3-NO2 Intermediate 2 Conformer 2

H	2.408946	0.942227	0.316935
C	2.020777	-0.075241	0.114571
O	0.969696	-0.241002	-0.483903

C	2.870125	-1.177754	0.622478
C	2.482574	-2.509156	0.426646
C	3.306149	-3.513872	0.917484
C	4.499396	-3.240588	1.595276
C	4.873957	-1.911935	1.784163
C	4.062861	-0.883077	1.299661
N	2.910808	-4.913291	0.718029
O	1.855054	-5.139482	0.124213
O	3.654354	-5.794033	1.154216
H	1.562779	-2.741882	-0.095367
H	5.111143	-4.055733	1.961263
H	5.795903	-1.682419	2.307678
H	4.352877	0.154136	1.445357

Aryl Substituent: 3-NO2 Intermediate 2 Conformer 3

H	1.099961	-0.408464	-0.389256
C	2.031327	-0.074101	0.108785
O	2.300043	1.112260	0.211517
C	2.890876	-1.166526	0.620893
C	2.480829	-2.491245	0.446465
C	3.299356	-3.507600	0.933078
C	4.506147	-3.244486	1.583747
C	4.904072	-1.916326	1.751697
C	4.102617	-0.881781	1.274129
N	2.878214	-4.901071	0.754947
O	1.808256	-5.114903	0.181535
O	3.614036	-5.790435	1.186656
H	1.549457	-2.729732	-0.054561
H	5.113041	-4.064619	1.946929
H	5.839478	-1.697198	2.255701
H	4.399293	0.154927	1.397636

Aryl Substituent: 3-NO2 Intermediate 2 Conformer 4

H	1.118020	-0.408320	-0.420235
C	2.023976	-0.073484	0.122347
O	2.268829	1.113676	0.267091
C	2.881183	-1.165971	0.638322
C	2.500449	-2.491727	0.412759
C	3.316652	-3.508046	0.903329
C	4.492816	-3.243802	1.607356
C	4.861562	-1.914663	1.826424
C	4.062003	-0.880159	1.345503
N	2.926085	-4.902389	0.671901
O	1.881026	-5.117305	0.054592
O	3.661247	-5.791571	1.105151
H	1.593004	-2.731078	-0.130015

H	5.099141	-4.063748	1.971891
H	5.772872	-1.694680	2.372419
H	4.336136	0.157368	1.508064

Aryl Substituent: 3-NO2 Intermediate 3 Conformer 0

H	2.523674	-0.499188	-0.477923
C	1.668366	0.159543	-0.283761
O	1.370484	0.934059	-1.425213
N	0.547491	-0.681201	0.130656
C	0.217627	-1.898596	-0.371830
N	1.084052	-2.513105	-1.192412
S	-1.277718	-2.623115	0.041073
C	2.045254	1.094172	0.858388
C	2.244755	2.453551	0.623339
C	2.620698	3.270189	1.690579
C	2.811102	2.780289	2.982146
C	2.613636	1.418347	3.202675
C	2.233483	0.582057	2.150905
N	2.826481	4.699257	1.439534
O	2.651134	5.123229	0.294555
O	3.166650	5.414186	2.385458
H	1.406057	0.362733	-2.205423
H	-0.196683	-0.223332	0.642176
H	2.014315	-2.166192	-1.380976
H	0.870899	-3.451549	-1.495295
H	2.113651	2.870513	-0.365025
H	3.105239	3.448369	3.781660
H	2.756315	1.007633	4.196799
H	2.080039	-0.476853	2.335915

Aryl Substituent: 3-NO2 Intermediate 3 Conformer 1

H	2.515734	-0.474767	-0.504900
C	1.664841	0.190419	-0.312855
O	1.385915	0.978954	-1.449537
N	0.531164	-0.642727	0.082035
C	0.189910	-1.847560	-0.444080
N	1.062503	-2.466496	-1.254742
S	-1.326083	-2.550336	-0.071615
C	2.039710	1.110249	0.841872
C	2.261164	2.468546	0.620168
C	2.633865	3.271596	1.698700
C	2.799343	2.769297	2.988932
C	2.580017	1.408628	3.196121
C	2.203197	0.585645	2.132690
N	2.863328	4.699586	1.461687
O	2.710662	5.134511	0.317607

O	3.200026	5.402368	2.417847
H	1.417251	0.413010	-2.233860
H	-0.217749	-0.177621	0.580215
H	2.004155	-2.135739	-1.413716
H	0.840223	-3.397528	-1.573629
H	2.149030	2.894376	-0.366754
H	3.091403	3.427169	3.797614
H	2.702824	0.988686	4.189040
H	2.032367	-0.472367	2.307159

Aryl Substituent: 3-NO2 Intermediate 3 Conformer 2

H	2.563896	-0.645900	-0.222131
C	1.699841	0.015376	-0.082962
O	1.401776	0.698185	-1.281603
N	0.585112	-0.803957	0.385879
C	0.273617	-2.060597	-0.023599
N	1.156955	-2.728721	-0.781754
S	-1.220290	-2.765553	0.425734
C	2.059308	1.038380	0.986940
C	2.270058	2.373256	0.644833
C	2.630772	3.273713	1.647899
C	2.794834	2.891147	2.978838
C	2.586765	1.552626	3.306619
C	2.221919	0.633683	2.320329
N	2.847972	4.676548	1.283797
O	2.698846	5.004581	0.104107
O	3.171128	5.466562	2.174371
H	1.448411	0.069533	-2.015776
H	-0.170044	-0.316630	0.852103
H	2.088483	-2.390847	-0.980572
H	0.956508	-3.690519	-1.011534
H	2.158333	2.707513	-0.376796
H	3.077114	3.621488	3.726465
H	2.708941	1.224618	4.333678
H	2.060044	-0.405785	2.589133

Aryl Substituent: 3-NO2 Intermediate 3 Conformer 3

H	2.794252	-0.646559	-0.249196
C	1.865538	-0.120903	0.003219
O	1.327280	0.508115	-1.139370
N	0.947109	-1.095345	0.588879
C	0.776644	-2.385492	0.199769
N	1.667211	-2.925756	-0.647508
S	-0.553132	-3.291138	0.784313
C	2.196729	0.944244	1.040358
C	2.125811	2.297457	0.714304

C	2.467769	3.239541	1.685458
C	2.883750	2.881562	2.967281
C	2.955442	1.524887	3.278752
C	2.614437	0.564046	2.324644
N	2.388128	4.661455	1.339841
O	2.013039	4.967572	0.205357
O	2.700574	5.488769	2.199826
H	1.370934	-0.111181	-1.881808
H	0.179387	-0.718432	1.131017
H	2.529140	-2.467996	-0.910535
H	1.585658	-3.908884	-0.859527
H	1.814182	2.614699	-0.270682
H	3.142047	3.644515	3.690641
H	3.277927	1.215863	4.267618
H	2.671245	-0.489840	2.579949

Aryl Substituent: 3-NO2 Intermediate 3 Conformer 4

H	1.262260	-0.483609	-0.121469
C	2.279303	-0.497369	0.289130
O	2.495209	-1.676664	1.034510
N	3.210431	-0.364341	-0.828232
C	3.089513	-0.929592	-2.057392
N	1.924114	-1.505380	-2.390812
S	4.407123	-0.899029	-3.150261
C	2.425772	0.701810	1.218063
C	2.485983	1.988793	0.678552
C	2.577567	3.077542	1.543818
C	2.613874	2.932372	2.931688
C	2.551186	1.644615	3.456501
C	2.453585	0.536728	2.608534
N	2.639109	4.424745	0.970901
O	2.611676	4.540170	-0.256896
O	2.714752	5.383200	1.743500
H	2.129757	-2.424587	0.540999
H	4.148722	-0.059526	-0.599569
H	1.095456	-1.463210	-1.813982
H	1.814099	-1.850435	-3.332492
H	2.464021	2.151365	-0.391819
H	2.688301	3.804058	3.569390
H	2.577083	1.500714	4.531804
H	2.399405	-0.462372	3.023736

Aryl Substituent: 3-NO2 Intermediate 4 Conformer 0

H	1.237709	0.548660	0.627321
C	2.082811	0.001319	0.195207
N	2.433389	-1.149711	0.642929

C	1.763751	-1.706856	1.738064
N	1.619115	-3.027836	1.623304
S	1.263398	-0.839111	3.106447
C	2.784011	0.649895	-0.915635
C	2.328418	1.899978	-1.353006
C	2.993563	2.524100	-2.405214
C	4.095850	1.947656	-3.037064
C	4.542772	0.700439	-2.594757
C	3.894788	0.054257	-1.545516
N	2.516174	3.832569	-2.863989
O	1.541788	4.327817	-2.294263
O	3.112442	4.373700	-3.796975
H	1.943012	-3.505005	0.791461
H	1.163700	-3.561362	2.349851
H	1.476069	2.379907	-0.886801
H	4.584619	2.466057	-3.852498
H	5.397779	0.236643	-3.075120
H	4.235843	-0.916116	-1.200746

Aryl Substituent: 3-NO2 Intermediate 4 Conformer 1

H	2.404148	-0.988095	0.439787
C	2.015873	-0.005035	0.151616
N	0.998928	0.511605	0.741580
C	0.333819	-0.208103	1.742343
N	-0.131352	0.588219	2.706046
S	0.060631	-1.881506	1.718218
C	2.718094	0.683911	-0.933802
C	2.299529	1.943751	-1.389162
C	3.003192	2.541203	-2.426009
C	4.111218	1.937202	-3.030211
C	4.521240	0.687962	-2.570811
C	3.830871	0.064659	-1.529519
N	2.568146	3.859123	-2.904956
O	1.594730	4.383484	-2.361045
O	3.198291	4.376885	-3.828838
H	0.053226	1.583105	2.675771
H	-0.637179	0.206137	3.492252
H	1.447796	2.437186	-0.939034
H	4.629599	2.442503	-3.835422
H	5.378279	0.201797	-3.024297
H	4.152621	-0.909590	-1.172808

Aryl Substituent: 3-NO2 Intermediate 4 Conformer 2

H	1.227810	0.522715	0.610281
C	2.079569	-0.017625	0.182470
N	2.434563	-1.167743	0.629426

C	1.760570	-1.732062	1.718573
N	1.646422	-3.056582	1.609181
S	1.219796	-0.869100	3.074931
C	2.783752	0.638340	-0.921839
C	2.322883	1.886852	-1.358452
C	2.990312	2.518489	-2.404620
C	4.100043	1.950911	-3.031311
C	4.552310	0.705272	-2.589947
C	3.902270	0.051651	-1.546522
N	2.507334	3.825055	-2.862467
O	1.525853	4.312037	-2.297748
O	3.105658	4.372782	-3.790277
H	1.991417	-3.530483	0.783964
H	1.188235	-3.594810	2.330497
H	1.464541	2.359333	-0.895681
H	4.591242	2.473902	-3.842331
H	5.413117	0.248354	-3.066495
H	4.248104	-0.917489	-1.203134

Aryl Substituent: 3-NO2 Intermediate 4 Conformer 3

H	2.359974	-1.033775	0.369599
C	1.991229	-0.035685	0.108544
N	0.987674	0.486425	0.715865
C	0.372680	-0.202445	1.768011
N	-0.947099	-0.011576	1.790419
S	1.193345	-1.110885	2.941863
C	2.700091	0.662932	-0.966397
C	2.314736	1.947531	-1.379930
C	3.030767	2.558457	-2.400315
C	4.117455	1.942434	-3.030534
C	4.492258	0.666973	-2.615168
C	3.788931	0.029825	-1.590980
N	2.629725	3.901705	-2.836045
O	1.671991	4.434342	-2.272581
O	3.271263	4.431630	-3.745080
H	-1.391726	0.549540	1.074795
H	-1.517774	-0.442040	2.503864
H	1.475871	2.447646	-0.913261
H	4.646385	2.456039	-3.823512
H	5.331482	0.170685	-3.090504
H	4.083342	-0.965056	-1.269351

Aryl Substituent: 3-NO2 Intermediate 4 Conformer 4

H	1.275812	0.570142	0.657106
C	2.098969	0.008903	0.201008
N	2.440109	-1.149860	0.635886

C	1.722739	-1.743459	1.680452
N	2.512741	-2.421154	2.514427
S	0.035105	-1.695443	1.841455
C	2.786465	0.650935	-0.922086
C	2.362336	1.923837	-1.323996
C	3.007972	2.537799	-2.394173
C	4.061226	1.929445	-3.077534
C	4.478543	0.660647	-2.668912
C	3.850501	0.024928	-1.601255
N	2.563893	3.870232	-2.815388
O	1.635219	4.395155	-2.197790
O	3.140822	4.400861	-3.766405
H	3.514688	-2.430333	2.371279
H	2.124404	-2.910706	3.307797
H	1.547437	2.427522	-0.817643
H	4.538031	2.440197	-3.904792
H	5.296831	0.173071	-3.188114
H	4.174054	-0.958918	-1.278523

Aryl Substituent: 3-NO2 Intermediate 6 Conformer 1

C	3.878133	-2.874864	-5.011950
C	5.280658	-2.309903	-4.871805
O	5.332919	-1.323375	-3.788576
C	5.071966	-0.050027	-4.077336
O	4.832599	0.386210	-5.189443
C	5.205759	0.798176	-2.813759
C	4.673966	2.267959	-2.921302
N	5.344891	2.924125	-4.038413
C	5.665986	4.235904	-4.124209
N	5.398858	5.035496	-3.080980
S	6.416169	4.844215	-5.540976
C	3.149952	2.402275	-2.959059
C	2.436567	2.285122	-4.155525
C	1.050233	2.412248	-4.125030
C	0.339873	2.654213	-2.947923
C	1.058537	2.775970	-1.762403
C	2.451716	2.654905	-1.768928
N	0.308635	2.287319	-5.385210
O	0.947411	2.087907	-6.420493
O	-0.919449	2.387345	-5.348528
C	4.659468	0.114893	-1.556336
O	5.176393	0.255271	-0.472964
C	3.371559	-0.761993	-1.611980
F	2.712102	-0.642286	-2.781968
F	3.688816	-2.054496	-1.435144

F	2.540425	-0.393299	-0.625561
H	3.888727	-3.673686	-5.760316
H	3.172529	-2.106316	-5.339194
H	3.532206	-3.292595	-4.062559
H	5.627425	-1.829766	-5.788139
H	5.998190	-3.071389	-4.563487
H	6.283419	0.880505	-2.627556
H	5.024608	2.736564	-1.996832
H	5.461616	2.379096	-4.884078
H	4.892245	4.738993	-2.259334
H	5.651637	6.009862	-3.143911
H	2.938791	2.095840	-5.094769
H	-0.738341	2.748609	-2.971061
H	0.536571	2.973859	-0.832132
H	2.998035	2.765912	-0.835732

Aryl Substituent: 3-NO2 Intermediate 6 Conformer 2

C	4.497302	-2.186151	0.081773
C	3.383073	-1.712640	-0.835857
O	3.911606	-0.866459	-1.908029
C	4.048787	0.434743	-1.653316
O	3.751569	0.990196	-0.613604
C	4.645273	1.159985	-2.864837
C	4.099189	2.632334	-2.939000
N	4.669121	3.351292	-4.076161
C	5.702040	4.227708	-4.096366
N	6.328573	4.523415	-2.945054
S	6.202739	4.918836	-5.580088
C	2.577012	2.649021	-3.037360
C	1.919292	1.981567	-4.073393
C	0.528224	2.050607	-4.141802
C	-0.234892	2.756119	-3.212100
C	0.430642	3.415222	-2.180688
C	1.823945	3.362530	-2.094026
N	-0.154360	1.349281	-5.234072
O	0.533618	0.730392	-6.048959
O	-1.384216	1.411929	-5.285559
C	6.174238	1.127277	-2.872922
O	6.817036	0.996539	-3.886865
C	6.933543	1.285263	-1.526037
F	6.442146	2.327046	-0.813757
F	6.776481	0.170855	-0.783614
F	8.234221	1.485208	-1.726961
H	4.079740	-2.873987	0.824219
H	4.957355	-1.347353	0.610346

H	5.269406	-2.716182	-0.483617
H	2.620774	-1.147517	-0.296433
H	2.914671	-2.542489	-1.366908
H	4.356201	0.629056	-3.774447
H	4.368364	3.139159	-2.012950
H	4.266342	3.161345	-4.985005
H	6.000546	4.237365	-2.033967
H	7.054946	5.223599	-2.968505
H	2.455136	1.414411	-4.825903
H	-1.313654	2.781790	-3.299617
H	-0.137052	3.970672	-1.441602
H	2.330645	3.879016	-1.284477

Aryl Substituent: 3-NO2 Intermediate 6 Conformer 3

C	3.514265	-2.397329	-0.308112
C	2.700387	-1.242354	-0.865240
O	3.431538	-0.553406	-1.932746
C	4.286520	0.399126	-1.569183
O	4.509552	0.761597	-0.430012
C	4.955657	1.033117	-2.793963
C	4.510307	2.521315	-2.917062
N	5.312325	3.130599	-3.982788
C	5.452483	4.466385	-4.213825
N	5.011253	5.319379	-3.279583
S	6.181751	5.010972	-5.658665
C	3.005037	2.643732	-3.132669
C	2.211623	3.222822	-2.141848
C	0.835623	3.325184	-2.353044
C	0.222805	2.869629	-3.518361
C	1.024521	2.291742	-4.502805
C	2.402249	2.180292	-4.313313
N	0.010078	3.945373	-1.312009
O	0.566743	4.333476	-0.282524
O	-1.200421	4.052893	-1.517318
C	6.480200	0.882576	-2.755032
O	7.077612	0.270833	-3.609824
C	7.323093	1.460419	-1.580694
F	6.786052	2.565147	-1.028952
F	7.438366	0.521976	-0.623908
F	8.550410	1.771233	-2.016711
H	2.911193	-2.932323	0.432837
H	4.422544	-2.039185	0.184132
H	3.788922	-3.100149	-1.100097
H	2.447796	-0.511519	-0.094700
H	1.789000	-1.586188	-1.356015

H	4.650246	0.488196	-3.690226
H	4.767850	3.002376	-1.971357
H	5.536370	2.555128	-4.785389
H	4.665815	5.028813	-2.375963
H	5.186836	6.304918	-3.407842
H	2.639830	3.590829	-1.216563
H	-0.847933	2.968150	-3.645014
H	0.572052	1.927723	-5.419172
H	3.009950	1.732304	-5.094441

Aryl Substituent: 3-NO2 Intermediate 6 Conformer 4

C	4.657554	-2.259270	0.162307
C	3.495819	-1.908404	-0.751089
O	3.937142	-1.057934	-1.859420
C	3.977689	0.256796	-1.649423
O	3.661071	0.823944	-0.622620
C	4.511965	0.973114	-2.897867
C	4.105848	2.475796	-2.900330
N	4.905370	3.153733	-3.923122
C	5.152760	4.489197	-3.989857
N	4.716768	5.264388	-2.988540
S	6.020159	5.133619	-5.315529
C	2.600562	2.652565	-3.100312
C	2.034023	2.497781	-4.368188
C	0.658853	2.664710	-4.516028
C	-0.178225	2.984117	-3.445642
C	0.395964	3.138691	-2.186802
C	1.773556	2.973646	-2.014599
N	0.072080	2.502340	-5.850174
O	0.823741	2.228056	-6.788533
O	-1.146180	2.646616	-5.970673
C	6.031346	0.790532	-3.028610
O	6.556057	0.379839	-4.035683
C	6.929017	1.132773	-1.807553
F	6.620259	2.341143	-1.294997
F	6.735554	0.209908	-0.840254
F	8.217311	1.129993	-2.146200
H	4.309302	-2.959963	0.928073
H	5.050710	-1.370566	0.662452
H	5.464916	-2.736665	-0.400700
H	2.696737	-1.389506	-0.218631
H	3.089699	-2.790472	-1.248076
H	4.100365	0.476338	-3.780949
H	4.377590	2.879762	-1.923257
H	5.143501	2.644202	-4.764695

H	4.157903	4.926558	-2.218223
H	4.928807	6.250531	-3.011440
H	2.637948	2.262001	-5.236173
H	-1.241861	3.108895	-3.604451
H	-0.229221	3.391444	-1.336786
H	2.208843	3.088552	-1.026907

Aryl Substituent: 3-NO2 Intermediate 6 Conformer 5

C	5.569561	-0.620552	1.472330
C	4.716119	-1.246810	0.382959
O	4.990778	-0.611448	-0.908250
C	4.306114	0.493173	-1.223769
O	3.430172	0.998714	-0.555757
C	4.809073	1.028202	-2.569689
C	4.380969	2.505477	-2.805217
N	5.252571	3.052760	-3.853019
C	5.517492	4.370553	-4.072876
N	5.089790	5.263706	-3.171393
S	6.399640	4.842435	-5.457876
C	2.898434	2.641432	-3.146930
C	2.023524	3.243863	-2.242742
C	0.679211	3.377724	-2.591065
C	0.173007	2.928709	-3.810080
C	1.054728	2.326900	-4.706372
C	2.404709	2.186550	-4.379207
N	-0.232042	4.016397	-1.637350
O	0.229997	4.411926	-0.564430
O	-1.418792	4.128861	-1.952190
C	6.324143	0.835817	-2.674655
O	6.860765	0.198820	-3.550173
C	7.238217	1.500354	-1.605454
F	6.518470	2.105269	-0.633214
F	8.025186	0.578994	-1.031187
F	8.017613	2.426384	-2.183852
H	5.425282	-1.173747	2.405805
H	5.288783	0.422436	1.643374
H	6.629207	-0.661934	1.204021
H	3.648405	-1.157271	0.589801
H	4.969901	-2.294606	0.217371
H	4.389655	0.390024	-3.355543
H	4.580861	3.048617	-1.878735
H	5.473724	2.457250	-4.641406
H	4.536628	5.026775	-2.360572
H	5.319323	6.236634	-3.308073
H	2.365713	3.597441	-1.278153

H	-0.877261	3.050892	-4.042766
H	0.689070	1.968822	-5.663013
H	3.076060	1.721962	-5.096053

Aryl Substituent: 3-NO2 Intermediate 7 Conformer 0

H	1.060127	0.203062	0.129330
C	2.143288	0.036254	0.176523
N	2.818683	0.845457	-0.840865
C	2.987093	0.487990	-2.124777
S	3.632339	1.553065	-3.284746
N	2.629468	-0.780342	-2.468438
C	2.003784	-1.755840	-1.602305
O	0.610255	-1.829157	-1.784039
C	2.414019	-1.453169	-0.131813
H	3.482992	-1.653052	-0.033018
C	1.651552	-2.330173	0.860109
O	0.510159	-2.113368	1.217944
O	2.414531	-3.341064	1.284919
C	1.815976	-4.287617	2.224338
C	1.981422	-3.809219	3.656912
C	2.515222	-3.150950	-2.049672
F	2.364812	-3.291084	-3.383048
F	3.818546	-3.314065	-1.764187
F	1.824436	-4.134821	-1.451278
C	2.631811	0.435576	1.560417
C	1.706608	0.705798	2.569616
C	2.175417	1.053697	3.836779
C	3.534801	1.146638	4.133274
C	4.450369	0.877106	3.117521
C	4.004171	0.523774	1.842347
N	1.198617	1.338746	4.892280
O	0.000236	1.235200	4.620406
O	1.621268	1.667759	6.002625
H	3.034945	1.807808	-0.615403
H	2.678773	-1.002594	-3.455180
H	0.260955	-0.936758	-1.923703
H	2.359178	-5.216369	2.042867
H	0.765585	-4.421938	1.958803
H	3.036876	-3.646940	3.894857
H	1.587737	-4.570390	4.338374
H	1.431169	-2.879717	3.826923
H	0.641375	0.649013	2.383700
H	3.857674	1.423317	5.128967
H	5.514136	0.942895	3.320447
H	4.729593	0.323473	1.059193

Aryl Substituent: 3-NO2 Intermediate 7 Conformer 1

H	1.065905	0.216154	0.125393
C	2.148511	0.046445	0.175614
N	2.828209	0.847386	-0.845284
C	2.996527	0.481731	-2.127084
S	3.643353	1.539195	-3.293158
N	2.638392	-0.788346	-2.462845
C	2.009160	-1.758009	-1.591925
O	0.617047	-1.833921	-1.779249
C	2.416032	-1.445804	-0.122451
H	3.484284	-1.647205	-0.019639
C	1.648628	-2.313619	0.873548
O	0.505473	-2.092698	1.223311
O	2.409005	-3.320389	1.312186
C	1.805345	-4.256892	2.258648
C	1.962158	-3.762474	3.686746
C	2.521671	-3.156151	-2.028705
F	2.371432	-3.306692	-3.360983
F	3.825110	-3.315494	-1.741861
F	1.832099	-4.136115	-1.422628
C	2.634980	0.453251	1.558111
C	1.708285	0.733269	2.563213
C	2.175312	1.088842	3.828906
C	3.534277	1.180046	4.127784
C	4.451358	0.900703	3.116035
C	4.006975	0.539556	1.842435
N	1.196963	1.384140	4.880154
O	-0.001084	1.282711	4.606066
O	1.618048	1.719141	5.989284
H	3.043841	1.811375	-0.626283
H	2.687128	-1.015976	-3.448306
H	0.261104	-0.939960	-1.889757
H	2.350073	-5.187235	2.090528
H	0.756602	-4.394568	1.988432
H	3.016058	-3.596163	3.928760
H	1.565775	-4.516755	4.374287
H	1.409645	-2.831991	3.843434
H	0.643286	0.678388	2.375384
H	3.855627	1.462956	5.122219
H	5.514854	0.965082	3.320797
H	4.733452	0.331886	1.062184

Aryl Substituent: 3-NO2 Intermediate 7 Conformer 2

H	1.091951	0.261471	0.067756
C	2.172079	0.075534	0.114791

N	2.857751	0.842887	-0.927279
C	3.008765	0.447775	-2.202693
S	3.663671	1.469546	-3.395717
N	2.624879	-0.822060	-2.509159
C	1.990250	-1.763992	-1.612237
O	0.595541	-1.823612	-1.785916
C	2.414947	-1.426791	-0.153746
H	3.481428	-1.639758	-0.056032
C	1.646367	-2.263013	0.867808
O	0.507097	-2.024240	1.218619
O	2.401616	-3.264401	1.327538
C	1.799067	-4.168041	2.305791
C	1.989000	-3.640976	3.718145
C	2.478950	-3.178443	-2.022895
F	2.313076	-3.356504	-3.349904
F	3.782949	-3.349559	-1.745358
F	1.782183	-4.134820	-1.388055
C	2.671186	0.502484	1.486818
C	4.041635	0.596101	1.743915
C	4.461802	0.973682	3.017888
C	3.566581	1.259502	4.049500
C	2.203078	1.162696	3.782548
C	1.758160	0.790297	2.510943
N	5.901074	1.074696	3.280094
O	6.680739	0.824804	2.358108
O	6.264045	1.404814	4.411032
H	3.091295	1.807574	-0.730701
H	2.663476	-1.071830	-3.489694
H	0.252223	-0.927270	-1.915427
H	2.325507	-5.111301	2.151752
H	0.743775	-4.294723	2.056732
H	3.049930	-3.490697	3.939043
H	1.588451	-4.368996	4.431121
H	1.459059	-2.694952	3.859496
H	4.777185	0.389854	0.975641
H	3.934925	1.551070	5.024913
H	1.484247	1.383637	4.564578
H	0.693260	0.720556	2.312242

Aryl Substituent: 3-NO2 Intermediate 7 Conformer 3

H	1.065091	0.129313	0.148068
C	2.150771	-0.025857	0.162047
N	2.791645	0.833531	-0.836701
C	2.939463	0.530360	-2.136892
S	3.553374	1.647611	-3.264253

N	2.586085	-0.726407	-2.525645
C	1.985421	-1.740250	-1.687295
O	0.586875	-1.808938	-1.834124
C	2.429815	-1.497515	-0.215883
H	3.502984	-1.689243	-0.152682
C	1.702907	-2.424707	0.756667
O	0.570513	-2.235293	1.155828
O	2.486395	-3.446048	1.114570
C	1.922666	-4.439035	2.027028
C	2.139543	-4.029589	3.474029
C	2.490144	-3.113147	-2.204936
F	2.310083	-3.197234	-3.539429
F	3.799880	-3.285014	-1.955800
F	1.814409	-4.123152	-1.633353
C	2.669818	0.320510	1.549001
C	1.767404	0.534805	2.591758
C	2.263936	0.835243	3.860452
C	3.629172	0.934493	4.126570
C	4.521784	0.720751	3.077690
C	4.047676	0.415523	1.800152
N	1.310851	1.062248	4.951155
O	0.107323	0.954281	4.705018
O	1.757544	1.349651	6.063631
H	3.003685	1.787909	-0.575926
H	2.623632	-0.909613	-3.520848
H	0.240885	-0.917768	-1.988620
H	2.459444	-5.356775	1.781467
H	0.863446	-4.562356	1.793527
H	3.202784	-3.876275	3.681286
H	1.771185	-4.823353	4.132068
H	1.595306	-3.110452	3.708290
H	0.698629	0.471242	2.430190
H	3.973797	1.173628	5.124681
H	5.589467	0.792762	3.256480
H	4.755468	0.258862	0.991329

Aryl Substituent: 3-NO2 Intermediate 7 Conformer 4

H	1.078112	0.157097	0.117917
C	2.163196	-0.003153	0.121825
N	2.796816	0.838600	-0.895904
C	2.924990	0.517234	-2.193920
S	3.529738	1.615725	-3.344399
N	2.557955	-0.741878	-2.561228
C	1.968931	-1.744115	-1.700821
O	0.568804	-1.815592	-1.829742

C	2.432336	-1.481339	-0.238864
H	3.505988	-1.673862	-0.185818
C	1.715915	-2.392927	0.755684
O	0.587755	-2.196586	1.163837
O	2.502788	-3.408861	1.121281
C	1.950417	-4.384762	2.058580
C	2.191366	-3.951796	3.494983
C	2.466990	-3.124232	-2.205658
F	2.268226	-3.227548	-3.536141
F	3.780420	-3.291640	-1.972721
F	1.800337	-4.126165	-1.610105
C	2.696820	0.358724	1.499429
C	4.072293	0.473773	1.718077
C	4.525062	0.789507	2.997694
C	3.657876	0.992640	4.071909
C	2.289057	0.875071	3.843124
C	1.811513	0.563822	2.566916
N	5.969261	0.914086	3.219549
O	6.723937	0.739690	2.260144
O	6.361105	1.187794	4.355904
H	3.017907	1.795104	-0.650848
H	2.584797	-0.939807	-3.553892
H	0.219299	-0.925455	-1.982262
H	2.480458	-5.308329	1.820287
H	0.887371	-4.508953	1.843656
H	3.258614	-3.802200	3.683548
H	1.827872	-4.731796	4.171932
H	1.657252	-3.024771	3.721368
H	4.786970	0.330936	0.916429
H	4.051022	1.238294	5.050285
H	1.591335	1.032212	4.658977
H	0.742697	0.477359	2.398285

Aryl Substituent: 3-OH Intermediate 10 Conformer 0

N	0.948297	-1.003179	0.671819
C	0.516991	-0.075553	1.524275
N	1.484709	0.454525	2.370551
C	2.281624	1.419499	2.154668
C	3.230747	1.876655	3.229133
C	4.678326	1.624534	2.832617
O	5.516735	2.206217	3.693456
C	6.948048	2.020967	3.465495
C	7.484779	3.058723	2.493989
O	5.009652	0.958260	1.868543
C	2.312692	2.178407	0.817060

F	3.183737	3.202934	0.855135
F	2.675634	1.359949	-0.190386
F	1.099173	2.679250	0.519759
S	-1.103566	0.351402	1.734468
H	1.929654	-1.237688	0.592284
H	0.280995	-1.526958	0.121547
H	3.014862	1.323989	4.147558
H	3.093358	2.942744	3.440049
H	7.113136	1.003541	3.105307
H	7.388660	2.130923	4.457848
H	7.042786	2.933398	1.501676
H	8.569808	2.941662	2.404298
H	7.276164	4.071799	2.850851

Aryl Substituent: 3-OH Intermediate 10 Conformer 1

N	0.856126	-0.851568	0.589589
C	0.455124	0.049221	1.484639
N	1.445683	0.529760	2.334127
C	2.257440	1.487133	2.141118
C	3.230839	1.887172	3.216749
C	4.667574	1.626601	2.787555
O	5.529160	2.168358	3.651549
C	6.953565	1.968736	3.393455
C	7.490401	3.029751	2.447488
O	4.972067	0.986175	1.797492
C	2.282581	2.294087	0.831763
F	3.171812	3.301723	0.894283
F	2.616670	1.507446	-0.210241
F	1.073751	2.825801	0.571739
S	-1.153929	0.494950	1.737813
H	1.831749	-1.099924	0.485318
H	0.171041	-1.343328	0.031718
H	3.019964	1.302844	4.116613
H	3.114974	2.946381	3.470780
H	7.096403	0.961435	2.997187
H	7.411951	2.039078	4.381335
H	7.029507	2.944333	1.459540
H	8.571672	2.898662	2.334737
H	7.304574	4.033574	2.841156

Aryl Substituent: 3-OH Intermediate 10 Conformer 2

N	0.980653	-0.680738	0.380079
C	0.526646	0.085181	1.369994
N	1.476054	0.483864	2.304509
C	2.286442	1.459997	2.238324
C	3.213219	1.759773	3.385338

C	4.671870	1.608371	2.979168
O	5.481810	2.125123	3.906347
C	6.920794	2.013326	3.680647
C	7.434868	3.190970	2.869811
O	5.034063	1.064863	1.951383
C	2.361832	2.383207	1.009750
F	3.164211	3.438691	1.238426
F	2.845389	1.717068	-0.058826
F	1.144647	2.852465	0.680346
S	-1.102641	0.438224	1.641026
H	1.968339	-0.864803	0.257803
H	0.327510	-1.106638	-0.263813
H	3.001597	1.057463	4.196785
H	3.042607	2.772001	3.767080
H	7.122210	1.060985	3.186333
H	7.345386	1.998463	4.685933
H	7.007772	3.191219	1.862884
H	8.524026	3.121138	2.781146
H	7.189096	4.138189	3.359229

Aryl Substituent: 3-OH Intermediate 10 Conformer 3

N	0.843322	-0.844050	0.596963
C	0.450533	0.058257	1.494129
N	1.449720	0.540859	2.332089
C	2.257420	1.500055	2.130710
C	3.244427	1.897288	3.194891
C	4.675267	1.627330	2.751908
O	5.547531	2.139256	3.623563
C	6.967886	1.918456	3.361580
C	7.429152	0.592981	3.943779
O	4.966883	1.012805	1.741788
C	2.264973	2.309379	0.822773
F	3.152039	3.319097	0.876060
F	2.587906	1.525232	-0.224853
F	1.051642	2.838580	0.578265
S	-1.156236	0.502647	1.764165
H	1.818125	-1.091248	0.482818
H	0.153128	-1.337075	0.046557
H	3.039793	1.316804	4.098578
H	3.137832	2.957910	3.447273
H	7.459306	2.764951	3.844245
H	7.133007	1.968401	2.283598
H	7.223952	0.545717	5.017472
H	8.509048	0.489931	3.794551
H	6.932542	-0.246964	3.449897

Aryl Substituent: 3-OH Intermediate 10 Conformer 4

N	0.956025	-1.158225	0.784558
C	0.556932	-0.100022	1.485589
N	1.553288	0.505368	2.242592
C	2.214135	1.557308	1.990438
C	3.215612	2.090993	2.980031
C	4.642831	1.760106	2.555676
O	5.518751	2.535342	3.200698
C	6.937991	2.300137	2.945323
C	7.410751	3.086335	1.734164
O	4.930213	0.883569	1.762355
C	2.022140	2.407028	0.727227
F	3.187249	2.972218	0.352488
F	1.553018	1.699320	-0.311076
F	1.151157	3.402955	0.988320
S	-1.049762	0.401655	1.635128
H	1.930211	-1.430879	0.746643
H	0.275854	-1.719033	0.290241
H	3.034567	1.621099	3.951214
H	3.110320	3.173030	3.103969
H	7.094614	1.227027	2.819471
H	7.429071	2.634679	3.860768
H	6.916488	2.738164	0.822924
H	8.490440	2.949340	1.613715
H	7.211301	4.154683	1.860846

Aryl Substituent: 3-OH Intermediate 10 Conformer 5

N	-0.358629	0.976000	0.658844
C	0.839540	1.411068	0.273775
N	1.695913	1.799832	1.298392
C	2.461789	1.072916	2.004370
C	3.338772	1.691551	3.058794
C	4.806955	1.621065	2.660673
O	5.588880	2.048981	3.655199
C	7.029082	2.081088	3.411739
C	7.441561	3.390711	2.761201
O	5.197207	1.235654	1.573676
C	2.505966	-0.456894	1.861957
F	3.489514	-0.995815	2.603040
F	1.337128	-0.987973	2.288058
F	2.685123	-0.835258	0.586056
S	1.298981	1.657970	-1.332304
H	-0.593869	0.858315	1.636159
H	-1.075080	0.804444	-0.033793
H	3.203983	1.192802	4.024373

H	3.056957	2.740786	3.181689
H	7.295537	1.219900	2.795936
H	7.467020	1.967019	4.404871
H	6.998996	3.490824	1.766161
H	8.531339	3.414563	2.656898
H	7.135436	4.244151	3.373635

Aryl Substituent: 3-OH Intermediate 10 Conformer 6

N	1.143390	-1.037919	0.536417
C	0.683912	-0.090119	1.349303
N	1.640814	0.469687	2.188456
C	2.247534	1.576858	2.072779
C	3.210565	2.039578	3.133347
C	4.657146	1.843724	2.691248
O	5.480544	2.606087	3.415694
C	6.912445	2.483812	3.151960
C	7.337363	3.394871	2.012542
O	5.001995	1.067285	1.820286
C	2.022675	2.559021	0.915115
F	3.155748	3.236714	0.640168
F	1.619612	1.952543	-0.211428
F	1.084867	3.460712	1.268633
S	-0.947223	0.312881	1.529238
H	2.130153	-1.258267	0.486224
H	0.497659	-1.572252	-0.028404
H	3.051235	1.442127	4.035867
H	3.041377	3.089745	3.389212
H	7.137990	1.436718	2.940158
H	7.378932	2.772689	4.095350
H	6.869312	3.091685	1.071964
H	8.424002	3.336683	1.890521
H	7.069852	4.434536	2.224052

Aryl Substituent: 3-OH Intermediate 11 Conformer 0

N	1.204938	-0.444484	0.284384
C	2.392861	0.024438	-0.099187
N	2.879938	-0.273823	-1.355918
C	2.338664	-0.828467	-2.509400
C	0.833904	-0.749538	-2.734182
F	0.344932	0.439770	-2.342997
F	0.506889	-0.929366	-4.020190
F	0.172894	-1.712860	-2.029992
C	3.087184	-1.349289	-3.511989
C	4.547431	-1.538253	-3.569113
O	5.213508	-1.011975	-2.517542
C	6.667887	-1.148168	-2.498158

C	7.333161	-0.020256	-3.268444
O	5.093856	-2.118726	-4.493718
S	3.365191	0.977718	0.922796
H	0.681152	-1.136176	-0.233554
H	0.871213	-0.196261	1.204352
H	3.878808	-0.103053	-1.436885
H	2.579278	-1.680214	-4.407147
H	6.923834	-2.127168	-2.906637
H	6.920364	-1.118202	-1.437029
H	7.073834	-0.063734	-4.329704
H	8.420164	-0.113898	-3.175030
H	7.036115	0.953985	-2.868714

Aryl Substituent: 3-OH Intermediate 11 Conformer 1

N	1.199690	0.615793	-0.117024
C	2.381162	-0.003611	-0.115001
N	2.843810	-0.622783	-1.258488
C	2.288149	-1.014296	-2.466218
C	0.778355	-1.076027	-2.635046
F	0.196784	-1.675561	-1.581235
F	0.236980	0.170372	-2.743391
F	0.425125	-1.743078	-3.740193
C	3.057970	-1.442009	-3.498890
C	4.523489	-1.486528	-3.446686
O	5.041230	-1.870225	-4.620523
C	6.492269	-1.983409	-4.712855
C	6.965809	-3.348446	-4.240184
O	5.203126	-1.214093	-2.455176
S	3.374229	-0.048811	1.267577
H	0.675695	0.815463	-0.957318
H	0.895525	1.055416	0.739490
H	3.846642	-0.829338	-1.231649
H	2.583901	-1.794188	-4.403474
H	6.943204	-1.175096	-4.133834
H	6.700420	-1.830358	-5.773233
H	6.756216	-3.490351	-3.176449
H	8.047525	-3.426136	-4.391515
H	6.480295	-4.147700	-4.808183

Aryl Substituent: 3-OH Intermediate 11 Conformer 2

N	1.287765	0.710503	-0.185902
C	2.413332	0.000417	-0.100861
N	2.872035	-0.703852	-1.196088
C	2.335314	-1.057108	-2.428627
C	0.821604	-1.124142	-2.586667
F	0.241521	-1.658633	-1.498423

F	0.283778	0.115985	-2.767424
F	0.458206	-1.853005	-3.649756
C	3.085482	-1.453057	-3.485445
C	4.553001	-1.495974	-3.609555
O	5.212383	-1.234467	-2.459432
C	6.672833	-1.247611	-2.498016
C	7.220334	0.097740	-2.943593
O	5.110205	-1.760685	-4.663183
S	3.340048	-0.061864	1.326134
H	0.808419	0.898473	-1.055318
H	0.977731	1.206665	0.636868
H	3.846181	-0.978869	-1.101648
H	2.572888	-1.787229	-4.376748
H	6.989214	-2.055705	-3.159816
H	6.960038	-1.482659	-1.472076
H	6.924429	0.320127	-3.972327
H	8.314113	0.073255	-2.897328
H	6.865844	0.899895	-2.289469

Aryl Substituent: 3-OH Intermediate 11 Conformer 3

N	1.154773	0.760379	-0.209700
C	2.330126	0.134514	-0.127705
N	2.815318	-0.576723	-1.206516
C	2.284897	-1.060467	-2.391913
C	0.778950	-1.127911	-2.590564
F	0.167667	-1.633370	-1.504821
F	0.250868	0.108367	-2.816456
F	0.446715	-1.883811	-3.643868
C	3.075074	-1.572431	-3.369445
C	4.538681	-1.617964	-3.280531
O	5.080184	-2.097777	-4.407384
C	6.532453	-2.220193	-4.459023
C	6.992210	-3.542507	-3.866385
O	5.197814	-1.268388	-2.299380
S	3.287696	0.191754	1.279204
H	0.656205	0.900264	-1.077302
H	0.832783	1.267836	0.601605
H	3.815904	-0.785246	-1.139882
H	2.619493	-1.993914	-4.253651
H	6.973471	-1.367756	-3.938597
H	6.763425	-2.154657	-5.523694
H	6.760525	-3.596555	-2.799150
H	8.076574	-3.633595	-3.988466
H	6.516194	-4.385048	-4.376975

Aryl Substituent: 3-OH Intermediate 11 Conformer 4

N	1.339119	0.820346	-0.298644
C	2.493544	0.158371	-0.213414
N	2.927834	-0.612000	-1.273484
C	2.345388	-1.075587	-2.447425
C	0.830623	-1.226327	-2.512164
F	0.338558	-1.697188	-1.353460
F	0.219599	-0.032360	-2.758880
F	0.449163	-2.055996	-3.491833
C	3.056808	-1.509521	-3.515816
C	4.516004	-1.492019	-3.719897
O	5.221459	-1.123511	-2.627971
C	6.676714	-1.072258	-2.747327
C	7.133360	0.263789	-3.308252
O	5.028798	-1.800780	-4.783967
S	3.485579	0.239083	1.168014
H	0.813955	0.922700	-1.155934
H	1.045735	1.366308	0.498295
H	3.916888	-0.838433	-1.211475
H	2.514767	-1.930539	-4.351248
H	6.997327	-1.907708	-3.372085
H	7.028678	-1.224530	-1.725903
H	6.772554	0.404052	-4.330760
H	8.227956	0.291503	-3.322437
H	6.775992	1.090905	-2.687632

Aryl Substituent: 3-OH Intermediate 11 Conformer 5

N	1.223489	0.593969	-0.113527
C	2.397325	-0.039866	-0.112684
N	2.856977	-0.652385	-1.260963
C	2.300450	-1.022642	-2.475106
C	0.790325	-1.070172	-2.646816
F	0.201786	-1.674434	-1.599647
F	0.259569	0.181538	-2.745138
F	0.433628	-1.724448	-3.758611
C	3.068506	-1.442904	-3.512083
C	4.533785	-1.496003	-3.459207
O	5.050122	-1.874662	-4.635304
C	6.501470	-1.977521	-4.733094
C	7.124273	-0.632916	-5.072234
O	5.213832	-1.242968	-2.462897
S	3.383624	-0.112135	1.273383
H	0.707769	0.811728	-0.954502
H	0.920995	1.028451	0.746229
H	3.857182	-0.870349	-1.233357
H	2.593229	-1.780323	-4.421655

H	6.659685	-2.706554	-5.529770
H	6.887114	-2.377167	-3.793058
H	6.700891	-0.229809	-5.997153
H	8.202542	-0.760552	-5.213765
H	6.966032	0.087824	-4.265341

Aryl Substituent: 3-OH Intermediate 11 Conformer 6

N	1.219163	0.646820	-0.127852
C	2.348260	-0.058883	-0.056188
N	2.827318	-0.715494	-1.172135
C	2.314042	-1.021339	-2.426851
C	0.803637	-1.083159	-2.615660
F	0.205458	-1.666146	-1.562767
F	0.266322	0.163181	-2.750854
F	0.460965	-1.764224	-3.716444
C	3.084766	-1.376777	-3.483468
C	4.554296	-1.423178	-3.578710
O	5.191402	-1.184039	-2.411030
C	6.651628	-1.223975	-2.414036
C	7.159927	-2.641613	-2.213958
O	5.132125	-1.663326	-4.627066
S	3.255795	-0.173530	1.380066
H	0.751107	0.868216	-0.995520
H	0.894308	1.106438	0.710301
H	3.802047	-0.987522	-1.073583
H	2.589836	-1.677411	-4.396433
H	6.934194	-0.571194	-1.586604
H	7.002067	-0.794177	-3.353881
H	6.771248	-3.068395	-1.284479
H	8.253257	-2.626308	-2.155314
H	6.868834	-3.283866	-3.049533

Aryl Substituent: 3-OH Intermediate 12 Conformer 0

O	-0.026371	2.344591	-1.372967
C	0.064196	1.109918	-0.793744
C	-1.017982	0.223336	-0.764279
C	-0.889861	-1.034671	-0.157646
C	-2.067343	-2.012533	-0.068426
C	-2.959793	-2.127105	-1.352092
C	-2.127513	-2.143362	-2.633503
O	-1.227713	-3.135723	-2.584251
C	-0.299640	-3.268185	-3.708893
C	0.914905	-2.375609	-3.523122
O	-2.260347	-1.367159	-3.559039
H	-3.422395	-3.119437	-1.272544
C	-4.102725	-1.137877	-1.446124

C	-5.145858	-1.375501	-2.556616
F	-5.006262	-2.595636	-3.108092
F	-6.393856	-1.294185	-2.052101
F	-5.040089	-0.460550	-3.533182
N	-4.222910	-0.197299	-0.604464
C	-5.186499	0.799136	-0.518790
N	-6.152157	0.573527	0.368758
S	-4.982211	2.245948	-1.366176
O	-1.645219	-3.317725	0.300931
C	0.335666	-1.385840	0.424438
C	1.412178	-0.494371	0.388270
C	1.289730	0.753569	-0.218073
H	-0.918669	2.471982	-1.727737
H	-1.956585	0.535998	-1.209266
H	-2.731410	-1.686361	0.738843
H	-0.842682	-3.034785	-4.626359
H	-0.032387	-4.325915	-3.702689
H	0.632735	-1.319408	-3.532493
H	1.617630	-2.552609	-4.344041
H	1.421401	-2.595989	-2.579224
H	-6.221101	-0.305496	0.865779
H	-6.826026	1.299315	0.570705
H	-1.085977	-3.651631	-0.419335
H	0.441994	-2.350208	0.906907
H	2.358517	-0.777208	0.841506
H	2.118632	1.454058	-0.250872

Aryl Substituent: 3-OH Intermediate 12 Conformer 1

O	-0.602554	-6.753750	0.866250
C	-0.186882	-5.473695	0.640421
C	-1.082262	-4.418177	0.440636
C	-0.604174	-3.118990	0.214706
C	-1.576513	-1.964732	0.028194
C	-2.443601	-2.151235	-1.259027
C	-1.596882	-1.953706	-2.523846
O	-2.108855	-2.645792	-3.545907
C	-1.431108	-2.537755	-4.837355
C	-0.288720	-3.534266	-4.938716
O	-0.607049	-1.250293	-2.580942
H	-2.844814	-3.166911	-1.285211
C	-3.669965	-1.250883	-1.261129
C	-3.464229	0.244754	-1.546304
F	-2.375185	0.721965	-0.916086
F	-3.279791	0.417850	-2.873765
F	-4.512762	0.991007	-1.171509

N	-4.809329	-1.773508	-1.069178
C	-6.068348	-1.187477	-1.062689
N	-6.548776	-0.910646	0.146795
S	-6.942026	-1.044103	-2.502021
O	-2.417283	-1.761261	1.161480
C	0.774864	-2.886790	0.184129
C	1.665061	-3.947898	0.380385
C	1.195259	-5.238798	0.609926
H	-1.570685	-6.788128	0.858610
H	-2.150896	-4.625893	0.462571
H	-1.006941	-1.042285	-0.080583
H	-1.086461	-1.509255	-4.962080
H	-2.217637	-2.748750	-5.563591
H	0.493912	-3.310837	-4.208546
H	0.149832	-3.478153	-5.940427
H	-0.646574	-4.555287	-4.775992
H	-5.988887	-1.040590	0.980137
H	-7.502550	-0.591620	0.247751
H	-2.853998	-2.597016	1.382861
H	1.149581	-1.883511	0.005591
H	2.735876	-3.765799	0.356349
H	1.876852	-6.069588	0.765013

Aryl Substituent: 3-OH Intermediate 12 Conformer 2

O	-1.550989	-6.961402	1.365212
C	-0.899413	-5.915357	0.775760
C	-1.522076	-4.688591	0.528781
C	-0.809047	-3.646739	-0.080496
C	-1.494626	-2.311881	-0.321771
C	-2.590377	-2.365784	-1.434746
C	-2.080114	-2.129495	-2.855441
O	-0.888842	-2.685098	-3.066354
C	-0.305981	-2.549778	-4.400463
C	0.457653	-1.242873	-4.529968
O	-2.728569	-1.521557	-3.689700
H	-3.050222	-3.359814	-1.428809
C	-3.726080	-1.393138	-1.167876
C	-3.415608	0.112619	-1.131704
F	-2.112411	0.360221	-1.374184
F	-4.134608	0.764724	-2.069294
F	-3.723589	0.649392	0.060122
N	-4.902775	-1.840346	-1.006727
C	-6.075003	-1.141424	-0.749210
N	-6.744371	-0.737768	-1.828032
S	-6.648908	-1.000837	0.833798

O	-2.205501	-1.867955	0.836295
C	0.533034	-3.834892	-0.425607
C	1.150223	-5.064728	-0.174149
C	0.444075	-6.107447	0.422071
H	-2.466734	-6.706805	1.552103
H	-2.557585	-4.539862	0.826273
H	-0.737623	-1.572931	-0.605956
H	-1.107721	-2.628407	-5.137101
H	0.353745	-3.414610	-4.485925
H	-0.213973	-0.384534	-4.440976
H	0.938530	-1.202883	-5.512862
H	1.234999	-1.167058	-3.763726
H	-6.364496	-0.847252	-2.759646
H	-7.666080	-0.335545	-1.725373
H	-1.560782	-1.727102	1.545270
H	1.089981	-3.028279	-0.892324
H	2.192980	-5.210800	-0.442385
H	0.913306	-7.065696	0.623623

Aryl Substituent: 3-OH Intermediate 12 Conformer 3

O	2.638217	-4.763573	0.379341
C	1.282222	-4.627217	0.448706
C	0.625989	-3.456396	0.050120
C	-0.766878	-3.363824	0.137036
C	-1.456575	-2.063718	-0.235692
C	-2.587977	-2.240783	-1.308375
C	-2.107180	-2.150920	-2.754024
O	-0.918547	-2.730312	-2.919955
C	-0.348786	-2.731055	-4.266297
C	0.422747	-1.448992	-4.529104
O	-2.763481	-1.626528	-3.636985
H	-3.043152	-3.230596	-1.196406
C	-3.716291	-1.249197	-1.092436
C	-3.424453	0.251921	-1.254576
F	-2.124921	0.481906	-1.529074
F	-4.152354	0.765116	-2.268258
F	-3.739941	0.935763	-0.142377
N	-4.872259	-1.680447	-0.792082
C	-6.027089	-0.957635	-0.517500
N	-6.758096	-0.620319	-1.578433
S	-6.498541	-0.708148	1.085187
O	-2.005064	-1.405791	0.912863
C	-1.503194	-4.450628	0.630251
C	-0.841470	-5.614393	1.030751
C	0.546380	-5.712274	0.942295

H	3.028695	-3.949669	0.027996
H	1.205629	-2.618637	-0.330972
H	-0.714360	-1.366649	-0.627231
H	-1.158556	-2.873837	-4.984307
H	0.303598	-3.605624	-4.274312
H	-0.242116	-0.580842	-4.516780
H	0.893587	-1.507430	-5.515914
H	1.208364	-1.305773	-3.781135
H	-6.439728	-0.799668	-2.522260
H	-7.673673	-0.212079	-1.447107
H	-2.496459	-2.056021	1.437168
H	-2.586567	-4.399586	0.706025
H	-1.412846	-6.455524	1.413072
H	1.069428	-6.613422	1.247482

Aryl Substituent: 3-OH Intermediate 12 Conformer 4

O	-1.348345	-6.617797	1.608402
C	-0.722686	-5.569588	0.999082
C	-1.416736	-4.442220	0.546566
C	-0.726946	-3.389721	-0.072788
C	-1.464371	-2.132285	-0.495711
C	-2.662019	-2.400291	-1.475611
C	-2.269758	-2.463959	-2.949001
O	-1.107955	-3.091369	-3.129098
C	-0.625509	-3.239041	-4.501252
C	0.156119	-2.011080	-4.936237
O	-2.967947	-2.008422	-3.837894
H	-3.117914	-3.366765	-1.236192
C	-3.765476	-1.375654	-1.287695
C	-3.483629	0.092521	-1.650185
F	-2.196609	0.279892	-2.006034
F	-4.255727	0.495457	-2.673470
F	-3.740476	0.900266	-0.602164
N	-4.887119	-1.750606	-0.826172
C	-6.025259	-0.994177	-0.572774
N	-6.106654	-0.491973	0.658191
S	-7.260688	-0.907692	-1.721165
O	-1.943949	-1.393342	0.634600
C	0.659673	-3.475100	-0.235806
C	1.346444	-4.607101	0.214005
C	0.666501	-5.654785	0.831147
H	-2.299249	-6.441615	1.663929
H	-2.496145	-4.401798	0.682819
H	-0.759275	-1.461924	-0.989221
H	-1.482356	-3.427826	-5.150701

H	0.005485	-4.128375	-4.462751
H	-0.487098	-1.127304	-4.968420
H	0.561078	-2.178367	-5.939703
H	0.991066	-1.818787	-4.255850
H	-5.339589	-0.568364	1.313733
H	-6.956150	-0.033495	0.958119
H	-2.355089	-2.012835	1.255998
H	1.198398	-2.662579	-0.714115
H	2.422992	-4.672779	0.083208
H	1.189164	-6.538419	1.184408

Aryl Substituent: 3-OH Intermediate 12 Conformer 5

O	-1.342334	-6.795958	1.292469
C	-0.693984	-5.690381	0.824482
C	-1.370250	-4.517471	0.472653
C	-0.658068	-3.406420	-0.002178
C	-1.382519	-2.110230	-0.317363
C	-2.528181	-2.270386	-1.377747
C	-2.071999	-2.124940	-2.826901
O	-0.882151	-2.688325	-3.033152
C	-0.334061	-2.634081	-4.387418
C	0.419475	-1.334916	-4.616381
O	-2.746606	-1.573910	-3.679401
H	-2.966447	-3.270333	-1.291364
C	-3.668067	-1.304566	-1.111399
C	-3.401910	0.205801	-1.225123
F	-2.110568	0.466384	-1.509847
F	-4.152916	0.742059	-2.209696
F	-3.711783	0.845289	-0.085205
N	-4.812622	-1.764301	-0.809364
C	-5.973972	-1.069669	-0.492465
N	-6.725430	-0.705380	-1.529893
S	-6.425611	-0.885960	1.124740
O	-1.924359	-1.500388	0.860552
C	0.733492	-3.479415	-0.123258
C	1.402878	-4.656958	0.224769
C	0.700465	-5.762809	0.698665
H	-2.294396	-6.621676	1.333205
H	-2.454029	-4.486688	0.572474
H	-0.658774	-1.387215	-0.696066
H	-1.153573	-2.760531	-5.097429
H	0.327661	-3.500214	-4.436910
H	-0.254242	-0.475147	-4.564063
H	0.875969	-1.353933	-5.611457
H	1.214803	-1.209017	-3.875625

H	-6.417196	-0.842875	-2.484005
H	-7.644349	-0.314917	-1.370796
H	-2.401403	-2.173523	1.368626
H	1.289839	-2.622336	-0.490845
H	2.483442	-4.712829	0.126535
H	1.209675	-6.681937	0.971982

Aryl Substituent: 3-OH Intermediate 12 Conformer 6

O	-1.438111	-6.774598	1.465276
C	-0.777286	-5.723406	0.899370
C	-1.428272	-4.544642	0.520674
C	-0.703928	-3.491728	-0.056802
C	-1.397252	-2.187260	-0.405350
C	-2.604726	-2.360291	-1.393039
C	-2.224439	-2.313118	-2.870265
O	-1.073251	-2.940937	-3.108165
C	-0.601466	-2.985300	-4.491117
C	0.194267	-1.737194	-4.833155
O	-2.921277	-1.778242	-3.715056
H	-3.073760	-3.335800	-1.226120
C	-3.692163	-1.336830	-1.122673
C	-3.377415	0.153597	-1.333556
F	-2.093753	0.348500	-1.694541
F	-4.157402	0.664856	-2.308980
F	-3.606546	0.865127	-0.217358
N	-4.835511	-1.733700	-0.738032
C	-5.952119	-0.976965	-0.403091
N	-6.740450	-0.636466	-1.421104
S	-6.314201	-0.691459	1.221870
O	-1.852230	-1.494226	0.763228
C	0.674324	-3.628048	-0.252272
C	1.318194	-4.811167	0.123926
C	0.603609	-5.859730	0.699022
H	-2.379240	-6.560492	1.547971
H	-2.502239	-4.463051	0.679908
H	-0.669724	-1.514643	-0.861718
H	-1.465131	-3.110397	-5.146978
H	0.017387	-3.883189	-4.528170
H	-0.436837	-0.845129	-4.790813
H	0.590742	-1.829867	-5.849593
H	1.036037	-1.610287	-4.145822
H	-6.485769	-0.835610	-2.380138
H	-7.634593	-0.201051	-1.239176
H	-2.321337	-2.121251	1.334045
H	1.239915	-2.815849	-0.699134

H	2.388215	-4.916602	-0.032075
H	1.092839	-6.782754	0.994804

Aryl Substituent: 3-OH Intermediate 13 Conformer 0

O	2.029409	0.831763	0.042879
C	0.705797	0.504158	0.042679
C	0.264949	-0.819011	0.028264
C	-1.116547	-1.104832	0.023728
C	-1.717577	-2.434699	-0.029773
C	-1.249379	-3.712989	0.059145
C	0.159460	-4.132265	0.327020
O	0.424929	-5.268783	-0.342069
C	1.732058	-5.887373	-0.146812
C	1.717661	-6.828550	1.046841
O	0.963017	-3.552772	1.036369
C	-2.237846	-4.798718	-0.154900
C	-2.181909	-6.043848	0.755412
F	-1.359507	-5.866082	1.806149
F	-3.404851	-6.322474	1.244077
F	-1.762302	-7.130316	0.074119
N	-3.159503	-4.665659	-1.029707
C	-4.238424	-5.492243	-1.295962
N	-3.981198	-6.570021	-2.036707
S	-5.788710	-5.018561	-0.813087
C	-2.032230	-0.028643	0.028381
C	-1.579206	1.289132	0.054151
C	-0.213970	1.564971	0.060480
H	2.565426	0.024856	0.034555
H	0.994666	-1.617981	0.030668
H	-2.791993	-2.388182	-0.195363
H	2.475360	-5.096107	-0.033639
H	1.911399	-6.423488	-1.080321
H	1.552328	-6.281197	1.978830
H	2.684120	-7.338685	1.113328
H	0.933434	-7.582358	0.936480
H	-3.040036	-6.804097	-2.325817
H	-4.745219	-7.135507	-2.381303
H	-3.098023	-0.235154	0.010814
H	-2.293013	2.107321	0.062897
H	0.156430	2.585394	0.076192

Aryl Substituent: 3-OH Intermediate 13 Conformer 1

O	-1.233006	2.158554	1.706165
C	-0.765068	1.110621	0.970347
C	-1.328963	-0.164754	1.034573
C	-0.802268	-1.226209	0.268259

C	-1.488236	-2.511067	0.395770
C	-1.108960	-3.791606	0.130661
C	0.260681	-4.223952	-0.292937
O	0.163208	-5.260544	-1.144882
C	1.400126	-5.878360	-1.611252
C	1.861093	-6.966501	-0.654997
O	1.310832	-3.728272	0.070657
C	-2.138496	-4.846143	0.282325
C	-1.734885	-6.195101	0.913166
F	-0.522171	-6.136837	1.494714
F	-2.619480	-6.555220	1.861436
F	-1.709843	-7.178287	-0.010557
N	-3.350558	-4.608252	-0.046159
C	-4.471944	-5.403537	0.123054
N	-4.633213	-6.383992	-0.765336
S	-5.606739	-4.999569	1.310489
C	0.279382	-0.971557	-0.597865
C	0.820639	0.310604	-0.668900
C	0.316298	1.351595	0.112191
H	-1.979548	1.866947	2.250464
H	-2.178839	-0.342993	1.689244
H	-2.503446	-2.420371	0.777717
H	2.152463	-5.096893	-1.732855
H	1.138699	-6.285999	-2.589296
H	2.137938	-6.546958	0.315970
H	2.738362	-7.467374	-1.077187
H	1.073751	-7.710420	-0.506143
H	-3.938398	-6.577199	-1.475326
H	-5.496434	-6.910248	-0.780210
H	0.683893	-1.757058	-1.221264
H	1.647379	0.506477	-1.345449
H	0.738752	2.350377	0.060112

Aryl Substituent: 3-OH Intermediate 13 Conformer 2

O	2.013144	0.724949	0.594007
C	0.723296	0.398344	0.295685
C	0.300675	-0.924363	0.165042
C	-1.046670	-1.210099	-0.141288
C	-1.637346	-2.538898	-0.270595
C	-1.169925	-3.820879	-0.243141
C	0.249238	-4.261620	-0.105776
O	0.289932	-5.432132	0.556368
C	1.581458	-6.095172	0.692726
C	2.312637	-5.618026	1.936296
O	1.236290	-3.683141	-0.526513

C	-2.186453	-4.899662	-0.328789
C	-1.890359	-6.131310	-1.211404
F	-0.800701	-5.956035	-1.981127
F	-1.701374	-7.236373	-0.461828
F	-2.928683	-6.375104	-2.033869
N	-3.312350	-4.771033	0.260813
C	-4.428089	-5.589582	0.205121
N	-4.392256	-6.681591	0.968424
S	-5.782733	-5.091043	-0.676844
C	-1.946685	-0.133460	-0.311226
C	-1.509806	1.184007	-0.187128
C	-0.178663	1.459427	0.116778
H	2.539979	-0.082366	0.690032
H	1.018875	-1.724561	0.286074
H	-2.716216	-2.488860	-0.402695
H	2.162192	-5.913576	-0.214042
H	1.326152	-7.154505	0.754888
H	2.570592	-4.558423	1.857121
H	3.238686	-6.190763	2.051850
H	1.700415	-5.769047	2.830451
H	-3.567665	-6.932497	1.498665
H	-5.225655	-7.243149	1.079994
H	-2.988343	-0.339504	-0.537853
H	-2.210502	2.002165	-0.323789
H	0.178415	2.479683	0.218073

Aryl Substituent: 3-OH Intermediate 13 Conformer 3

O	-1.377978	2.021672	1.845914
C	-0.869887	1.008291	1.088858
C	-1.403076	-0.281747	1.103024
C	-0.837820	-1.305502	0.313073
C	-1.495481	-2.608780	0.386445
C	-1.084087	-3.869100	0.075325
C	0.309639	-4.238672	-0.334123
O	0.271063	-5.198544	-1.273672
C	1.541471	-5.744438	-1.741575
C	2.007624	-6.885688	-0.852158
O	1.331970	-3.751386	0.111503
C	-2.089033	-4.954753	0.172448
C	-1.636117	-6.343548	0.671024
F	-0.456665	-6.271059	1.321271
F	-2.538978	-6.847135	1.535510
F	-1.503058	-7.223297	-0.336286
N	-3.316090	-4.714333	-0.087553
C	-4.415418	-5.552059	-0.024900

N	-5.205355	-5.367341	1.031973
S	-4.791926	-6.582000	-1.315298
C	0.252987	-0.997598	-0.524057
C	0.765762	0.297890	-0.542801
C	0.222062	1.301310	0.260780
H	-2.133450	1.698250	2.358998
H	-2.260422	-0.500493	1.735276
H	-2.516745	-2.554603	0.758776
H	2.272069	-4.934304	-1.781227
H	1.322989	-6.086299	-2.754642
H	2.238187	-6.530998	0.155998
H	2.914831	-7.325534	-1.279102
H	1.241897	-7.663167	-0.786050
H	-4.954678	-4.726028	1.773611
H	-6.083166	-5.863916	1.095036
H	0.686805	-1.751549	-1.166499
H	1.600190	0.534504	-1.196448
H	0.621284	2.310854	0.248048

Aryl Substituent: 3-OH Intermediate 13 Conformer 4

O	1.880155	0.688725	0.704665
C	0.622832	0.332536	0.316005
C	0.217242	-0.999148	0.231938
C	-1.097595	-1.314939	-0.170024
C	-1.666267	-2.656775	-0.261899
C	-1.166105	-3.923832	-0.187636
C	0.274955	-4.298676	-0.061340
O	0.388611	-5.408829	0.687286
C	1.710733	-6.009458	0.822401
C	2.465014	-5.413725	1.998824
O	1.218197	-3.712535	-0.563166
C	-2.148546	-5.036508	-0.235277
C	-1.775451	-6.323635	-1.003449
F	-0.731092	-6.120504	-1.831754
F	-1.462394	-7.335663	-0.177731
F	-2.814861	-6.725639	-1.763241
N	-3.304718	-4.894197	0.287102
C	-4.376828	-5.768619	0.318191
N	-5.354492	-5.493661	-0.544044
S	-4.480839	-6.964408	1.512761
C	-1.985841	-0.259531	-0.475676
C	-1.564796	1.067048	-0.400429
C	-0.264588	1.372324	-0.005154
H	2.399764	-0.105941	0.897539
H	0.926432	-1.780842	0.469824

H	-2.744169	-2.632615	-0.407751
H	2.250836	-5.873435	-0.116898
H	1.501508	-7.070235	0.971380
H	2.678283	-4.354183	1.832814
H	3.416489	-5.941783	2.120865
H	1.890937	-5.520266	2.924160
H	-5.257667	-4.762128	-1.236632
H	-6.214954	-6.022591	-0.511806
H	-3.005147	-0.489212	-0.771035
H	-2.255010	1.869245	-0.643791
H	0.078569	2.400034	0.063681

Aryl Substituent: 3-OH Intermediate 13 Conformer 5

O	-1.509419	2.197154	-1.223741
C	-0.907442	1.086362	-0.711628
C	-1.492159	-0.180032	-0.768350
C	-0.824445	-1.307376	-0.244112
C	-1.541155	-2.577902	-0.339049
C	-1.133835	-3.876588	-0.291431
C	0.285777	-4.345389	-0.230241
O	0.352723	-5.485117	0.481335
C	1.638946	-6.168803	0.551779
C	2.471263	-5.652325	1.713369
O	1.243146	-3.804823	-0.752202
C	-2.188288	-4.917220	-0.316554
C	-1.956575	-6.195339	-1.149919
F	-0.896472	-6.081250	-1.970911
F	-1.758005	-7.265974	-0.354214
F	-3.032506	-6.456144	-1.916265
N	-3.294611	-4.720373	0.292436
C	-4.439759	-5.499062	0.297223
N	-4.421071	-6.563863	1.098677
S	-5.799772	-4.982723	-0.566046
C	0.426860	-1.130856	0.378798
C	0.990100	0.141699	0.449759
C	0.341196	1.250017	-0.096808
H	-2.359587	1.953891	-1.619407
H	-2.470164	-0.299114	-1.228954
H	-2.614157	-2.456345	-0.475962
H	2.152220	-6.040740	-0.403550
H	1.370610	-7.218659	0.682376
H	2.745096	-4.604144	1.564908
H	3.391472	-6.241186	1.787434
H	1.925125	-5.747138	2.656707
H	-3.591664	-6.824855	1.616475

H	-5.270274	-7.090644	1.252790
H	0.948139	-1.970649	0.817151
H	1.949818	0.276203	0.940081
H	0.780874	2.241407	-0.046457

Aryl Substituent: 3-OH Intermediate 13 Conformer 6

O	-1.669865	2.364405	0.039910
C	-0.981676	1.211125	-0.194914
C	-1.584130	-0.047340	-0.146451
C	-0.829711	-1.217289	-0.376031
C	-1.566850	-2.478871	-0.320715
C	-1.174377	-3.766107	-0.114951
C	0.213461	-4.206029	0.236173
O	0.465966	-5.399149	-0.331729
C	1.745884	-6.036097	-0.039070
C	1.658969	-6.879708	1.222515
O	1.002529	-3.593314	0.930743
C	-2.211373	-4.816976	-0.237095
C	-2.211589	-5.982413	0.774074
F	-1.397706	-5.743200	1.819360
F	-3.449125	-6.180834	1.264285
F	-1.817741	-7.133930	0.191497
N	-3.124337	-4.714094	-1.125646
C	-4.239346	-5.511453	-1.325749
N	-4.029374	-6.662538	-1.963750
S	-5.767475	-4.924700	-0.899971
C	0.536024	-1.096826	-0.699912
C	1.121228	0.166075	-0.767274
C	0.379742	1.320021	-0.508814
H	-2.594655	2.156954	0.240613
H	-2.644957	-0.125782	0.079595
H	-2.638151	-2.360003	-0.472443
H	2.508382	-5.259163	0.041519
H	1.943149	-6.648576	-0.920580
H	1.477537	-6.256011	2.102031
H	2.605573	-7.410971	1.365424
H	0.855387	-7.616747	1.141051
H	-3.098919	-6.965230	-2.222434
H	-4.817289	-7.222502	-2.260224
H	1.131556	-1.973775	-0.914599
H	2.171746	0.256781	-1.027612
H	0.835002	2.304469	-0.557106

Aryl Substituent: 3-OH Intermediate 14 Conformer 0

O	-0.706664	-1.696124	-2.584384
C	-0.446328	-1.047904	-1.412410

C	0.474992	-1.529131	-0.472906
C	0.690438	-0.812851	0.704851
C	0.000588	0.375775	0.953821
C	-0.923353	0.855450	0.016799
C	-1.630304	2.176320	0.255513
O	-1.212077	3.189031	-0.672167
C	-3.189137	2.080089	0.199302
C	-3.866659	1.716330	1.514439
O	-3.154506	0.850634	2.231884
C	-3.702581	0.429537	3.521406
C	-3.315347	1.407681	4.617257
O	-4.949761	2.177039	1.834680
C	-3.783756	3.393945	-0.302827
C	-3.618914	4.676844	0.559295
F	-2.900744	4.458587	1.681937
F	-3.003386	5.635637	-0.150763
F	-4.826978	5.140616	0.922281
O	-4.391327	3.503738	-1.342612
C	-1.145032	0.139520	-1.165892
H	-0.164883	-2.497458	-2.638059
H	1.012197	-2.454345	-0.666421
H	1.404567	-1.187145	1.432980
H	0.175225	0.927663	1.872527
H	-1.341858	2.559174	1.235353
H	-1.210769	2.810116	-1.564314
H	-3.492295	1.327622	-0.537241
H	-3.263334	-0.555980	3.683083
H	-4.785422	0.334971	3.421911
H	-2.228091	1.511760	4.681226
H	-3.682800	1.034786	5.578899
H	-3.756793	2.392248	4.439379
H	-1.857208	0.479225	-1.914126

Aryl Substituent: 3-OH Intermediate 14 Conformer 1

O	1.493399	-1.227838	1.724880
C	0.651667	-0.795945	0.741710
C	0.508107	-1.468116	-0.478002
C	-0.374262	-0.967544	-1.437396
C	-1.113694	0.190141	-1.191868
C	-0.966473	0.865814	0.030500
C	-1.709663	2.162612	0.290867
O	-1.284411	3.212106	-0.591698
C	-3.263332	2.032104	0.177246
C	-3.979543	1.641401	1.463632
O	-3.274937	0.783027	2.197477

C	-3.863729	0.331764	3.458529
C	-3.561364	1.311034	4.579956
O	-5.083006	2.075398	1.749250
C	-3.867942	3.336987	-0.336928
C	-3.766828	4.614718	0.542242
F	-3.066011	4.408413	1.677869
F	-3.171864	5.603381	-0.143724
F	-4.998505	5.030984	0.884210
O	-4.437999	3.442087	-1.398214
C	-0.082953	0.370688	0.991720
H	1.946637	-2.032529	1.432195
H	1.079875	-2.372769	-0.669702
H	-0.486031	-1.490509	-2.382932
H	-1.798899	0.558186	-1.951674
H	-1.463674	2.521695	1.291068
H	-1.248387	2.861169	-1.494520
H	-3.521154	1.278888	-0.575231
H	-3.398265	-0.639712	3.631233
H	-4.937261	0.200988	3.310208
H	-2.482729	1.452756	4.695806
H	-3.959206	0.914659	5.519890
H	-4.029196	2.281218	4.391258
H	0.045232	0.878345	1.943141

Aryl Substituent: 3-OH Intermediate 14 Conformer 2

O	-1.122291	-2.015376	-2.187007
C	-0.797389	-1.234091	-1.116931
C	0.135266	-1.630592	-0.149193
C	0.421171	-0.778450	0.917658
C	-0.211764	0.461570	1.028647
C	-1.151047	0.854246	0.066541
C	-1.796070	2.223205	0.161407
O	-1.387416	3.098249	-0.896950
C	-3.363101	2.187378	0.171674
C	-3.950229	1.877136	1.541850
O	-3.687245	0.622468	1.906370
C	-4.148629	0.184864	3.222950
C	-3.096445	0.473525	4.280000
O	-4.578119	2.687781	2.201138
C	-3.929214	3.505856	-0.346506
C	-3.574209	4.856450	0.336839
F	-2.685370	4.740438	1.341208
F	-3.063052	5.702012	-0.575525
F	-4.693197	5.414923	0.832397
O	-4.682904	3.565510	-1.291779

C	-1.441490	0.003469	-1.006322
H	-0.611381	-2.837743	-2.154321
H	0.628188	-2.595746	-0.235633
H	1.145516	-1.086320	1.666433
H	0.019950	1.121622	1.859514
H	-1.453589	2.709181	1.076334
H	-1.430481	2.611556	-1.734210
H	-3.713470	1.423734	-0.529054
H	-4.316496	-0.886351	3.100189
H	-5.096153	0.680495	3.441391
H	-2.142428	0.008936	4.013223
H	-3.425239	0.063051	5.240302
H	-2.945898	1.550279	4.400778
H	-2.166575	0.273034	-1.770312

Aryl Substituent: 3-OH Intermediate 14 Conformer 3

O	1.722486	-0.959772	1.732448
C	0.798091	-0.639386	0.781343
C	0.584124	-1.424428	-0.357987
C	-0.380084	-1.033463	-1.289130
C	-1.131732	0.126086	-1.094220
C	-0.914613	0.914274	0.047499
C	-1.672851	2.212741	0.247716
O	-1.338617	3.191138	-0.748588
C	-3.226385	2.041100	0.256907
C	-3.839691	1.725301	1.615170
O	-3.060725	0.940130	2.355639
C	-3.543044	0.572411	3.686865
C	-3.166824	1.632247	4.708201
O	-4.933086	2.151580	1.947528
C	-3.899170	3.295087	-0.297114
C	-3.768028	4.631334	0.486295
F	-3.000072	4.511722	1.590792
F	-3.227616	5.577274	-0.298026
F	-4.984255	5.055903	0.869871
O	-4.544696	3.317454	-1.319354
C	0.051110	0.529164	0.979718
H	2.177754	-1.776461	1.478945
H	1.165446	-2.330605	-0.509637
H	-0.546354	-1.643722	-2.172288
H	-1.880486	0.407440	-1.830710
H	-1.367953	2.662263	1.193630
H	-1.358041	2.765361	-1.619231
H	-3.518439	1.232891	-0.422794
H	-3.051353	-0.380141	3.889921

H	-4.622799	0.421759	3.633673
H	-2.084343	1.790042	4.726475
H	-3.483674	1.302435	5.703004
H	-3.660720	2.582830	4.487982
H	0.234338	1.125514	1.868657

Aryl Substituent: 3-OH Intermediate 14 Conformer 4

O	1.347397	-1.317612	1.968821
C	0.585917	-0.850321	0.937457
C	0.518226	-1.495955	-0.302885
C	-0.282646	-0.959493	-1.312939
C	-1.016718	0.207521	-1.097298
C	-0.947201	0.855454	0.146610
C	-1.685564	2.159609	0.382136
O	-1.173837	3.223319	-0.435168
C	-3.227201	2.062872	0.143107
C	-4.049635	1.632912	1.351163
O	-3.414558	0.741713	2.108775
C	-4.106776	0.254080	3.301893
C	-3.873232	1.184793	4.479563
O	-5.169677	2.068042	1.560736
C	-3.769084	3.399142	-0.359804
C	-3.718758	4.636101	0.580109
F	-3.128845	4.363803	1.764145
F	-3.039985	5.635435	-0.005121
F	-4.966110	5.071191	0.827899
O	-4.250130	3.560814	-1.457390
C	-0.144142	0.324887	1.158386
H	1.806888	-2.124320	1.691756
H	1.085263	-2.408143	-0.471197
H	-0.334851	-1.461622	-2.274798
H	-1.637998	0.603719	-1.896764
H	-1.513307	2.487486	1.408149
H	-1.070098	2.894527	-1.341148
H	-3.437895	1.346985	-0.659034
H	-3.672539	-0.732315	3.471478
H	-5.167761	0.150555	3.067272
H	-2.803908	1.299457	4.680536
H	-4.348181	0.763024	5.371394
H	-4.307657	2.170892	4.293089
H	-0.076398	0.811748	2.126774

Aryl Substituent: 3-OH Intermediate 14 Conformer 5

O	1.569604	-1.088857	1.962758
C	0.725722	-0.716833	0.957160
C	0.581916	-1.459154	-0.221134

C	-0.302350	-1.016645	-1.206957
C	-1.043400	0.152328	-1.028545
C	-0.896074	0.897974	0.152279
C	-1.640119	2.206489	0.340071
O	-1.212244	3.207884	-0.595534
C	-3.193173	2.070858	0.228882
C	-3.912639	1.724976	1.526396
O	-3.206273	0.900360	2.296157
C	-3.795098	0.498157	3.573656
C	-3.480490	1.515818	4.656720
O	-5.019921	2.163369	1.790114
C	-3.797828	3.356970	-0.330061
C	-3.694362	4.664839	0.503714
F	-3.019295	4.490644	1.660256
F	-3.068033	5.617612	-0.206250
F	-4.925205	5.112945	0.804079
O	-4.367733	3.426347	-1.394284
C	-0.010378	0.461133	1.139506
H	2.023700	-1.908659	1.717413
H	1.154853	-2.372631	-0.360386
H	-0.414329	-1.594016	-2.120262
H	-1.729950	0.473606	-1.808154
H	-1.397121	2.618018	1.320642
H	-1.169788	2.808665	-1.477787
H	-3.450401	1.292169	-0.497660
H	-3.336142	-0.469818	3.780351
H	-4.869920	0.370080	3.433206
H	-2.400090	1.651765	4.762880
H	-3.878287	1.159088	5.612399
H	-3.940089	2.482599	4.433009
H	0.118038	1.024119	2.059268

Aryl Substituent: 3-OH Intermediate 14 Conformer 6

O	-1.054746	-1.941377	-2.458007
C	-0.695328	-1.229267	-1.351107
C	0.290714	-1.669543	-0.458365
C	0.605980	-0.888999	0.654287
C	-0.048490	0.323153	0.884381
C	-1.037034	0.762093	-0.005528
C	-1.706963	2.106261	0.208747
O	-1.337353	3.061124	-0.797839
C	-3.267467	2.036644	0.262898
C	-3.858356	1.760144	1.639597
O	-3.110632	0.922714	2.354684
C	-3.574143	0.583456	3.700346

C	-3.093520	1.611036	4.710641
O	-4.910122	2.257240	2.006116
C	-3.873577	3.331469	-0.273725
C	-3.634633	4.656538	0.503061
F	-2.843248	4.488077	1.584363
F	-3.058621	5.566097	-0.298632
F	-4.809528	5.157381	0.921668
O	-4.545006	3.394745	-1.277492
C	-1.358222	-0.017856	-1.123148
H	-0.532690	-2.756276	-2.501836
H	0.800005	-2.613317	-0.637220
H	1.370262	-1.231788	1.346110
H	0.203380	0.925242	1.752278
H	-1.346738	2.534409	1.145116
H	-1.401117	2.635821	-1.666533
H	-3.631977	1.249509	-0.406441
H	-3.141962	-0.401079	3.885459
H	-4.662788	0.506964	3.681455
H	-2.002809	1.695177	4.692964
H	-3.398604	1.299091	5.714846
H	-3.529118	2.593730	4.509233
H	-2.122251	0.289589	-1.833075

Aryl Substituent: 3-OH Intermediate 15 Conformer 0

O	1.256855	-0.804183	-2.273231
C	0.395956	-0.511823	-1.259248
C	0.292684	-1.297802	-0.103514
C	-0.603066	-0.932722	0.905260
C	-1.413709	0.190484	0.769919
C	-1.325941	0.978814	-0.397028
C	-2.120829	2.178606	-0.622385
C	-3.311779	2.560070	-0.085978
C	-4.129080	1.722131	0.860823
O	-4.619622	0.635335	0.255798
C	-5.509754	-0.225189	1.034186
C	-4.738405	-1.239302	1.861785
O	-4.334404	2.028217	2.021879
C	-3.815487	3.893938	-0.483388
C	-5.204435	4.354743	0.039238
F	-6.113917	3.356287	-0.018760
F	-5.114301	4.764712	1.318597
F	-5.665347	5.374417	-0.694225
O	-3.212195	4.673133	-1.202766
C	-0.397230	0.630097	-1.396044
H	1.754199	-1.607065	-2.057129

H	0.920171	-2.178278	0.008796
H	-0.658989	-1.532388	1.808792
H	-2.067259	0.479953	1.583693
H	-1.695802	2.886492	-1.332322
H	-6.123615	-0.712346	0.274754
H	-6.141407	0.410111	1.658601
H	-4.066426	-1.829696	1.232314
H	-5.449482	-1.921001	2.340203
H	-4.154390	-0.750840	2.646320
H	-0.297991	1.237093	-2.290939

Aryl Substituent: 3-OH Intermediate 15 Conformer 1

O	-0.826522	-1.746373	2.104056
C	-0.656963	-1.009285	0.971558
C	0.270159	-1.357539	-0.022945
C	0.417303	-0.545590	-1.148725
C	-0.351431	0.606354	-1.295054
C	-1.310588	0.944435	-0.316070
C	-2.096525	2.149532	-0.539422
C	-3.298796	2.530346	-0.027075
C	-4.141214	1.688916	0.893868
O	-4.605217	0.596760	0.277428
C	-5.509228	-0.269870	1.033009
C	-4.755990	-1.258925	1.906639
O	-4.385126	1.996343	2.047112
C	-3.786194	3.870787	-0.421912
C	-5.191595	4.328430	0.057877
F	-6.098833	3.330703	-0.035698
F	-5.144363	4.732270	1.341344
F	-5.628031	5.352119	-0.685436
O	-3.157747	4.658039	-1.110604
C	-1.443883	0.134444	0.826770
H	-0.218249	-2.500594	2.097249
H	0.879585	-2.249470	0.097891
H	1.145300	-0.813163	-1.908595
H	-0.229145	1.241063	-2.167374
H	-1.652079	2.864044	-1.230454
H	-6.083452	-0.778291	0.256706
H	-6.176905	0.362601	1.622000
H	-4.046958	-1.846742	1.316774
H	-5.475823	-1.946145	2.363700
H	-4.214069	-0.750563	2.708325
H	-2.116310	0.400053	1.633270

Aryl Substituent: 3-OH Intermediate 15 Conformer 2

O	1.181831	-0.793219	-2.391880

C	0.354309	-0.508040	-1.348431
C	0.288192	-1.302705	-0.195883
C	-0.575105	-0.945895	0.843609
C	-1.390051	0.177893	0.742612
C	-1.339149	0.975293	-0.420308
C	-2.141939	2.176019	-0.610477
C	-3.320042	2.546127	-0.038927
C	-4.110929	1.691656	0.915465
O	-4.610195	0.610549	0.307499
C	-5.477429	-0.265138	1.094704
C	-4.681457	-1.285906	1.890196
O	-4.290742	1.981296	2.084952
C	-3.838551	3.883019	-0.405969
C	-5.217663	4.329692	0.153736
F	-6.123547	3.327641	0.103293
F	-5.100296	4.723475	1.436083
F	-5.699903	5.356562	-0.555566
O	-3.255064	4.675357	-1.127354
C	-0.442404	0.635002	-1.450939
H	1.683070	-1.599653	-2.199186
H	0.919349	-2.183523	-0.110301
H	-0.602327	-1.552471	1.743838
H	-2.018303	0.460830	1.578372
H	-1.736911	2.893711	-1.322253
H	-6.106404	-0.745275	0.343212
H	-6.097548	0.358254	1.742271
H	-4.020544	-1.863241	1.237331
H	-5.377341	-1.978500	2.375280
H	-4.082415	-0.804601	2.667771
H	-0.371792	1.248891	-2.343850

Aryl Substituent: 3-OH Intermediate 15 Conformer 3

O	1.604871	-0.512820	-2.278275
C	0.645624	-0.368154	-1.322309
C	0.470034	-1.289109	-0.280855
C	-0.526710	-1.072999	0.674847
C	-1.365573	0.034472	0.596366
C	-1.204158	0.959900	-0.457372
C	-2.021592	2.155016	-0.619710
C	-3.265432	2.440912	-0.146810
C	-4.107091	1.476196	0.642468
O	-4.476784	0.427070	-0.099497
C	-5.307994	-0.588879	0.542259
C	-6.780400	-0.230382	0.426490
O	-4.409714	1.649411	1.809809

C	-3.790683	3.792404	-0.437978
C	-5.234565	4.144829	0.015881
F	-6.093595	3.129071	-0.228956
F	-5.264001	4.408073	1.335856
F	-5.679365	5.225528	-0.635403
O	-3.167119	4.665484	-1.019157
C	-0.176602	0.758744	-1.398762
H	2.111420	-1.321749	-2.111576
H	1.118956	-2.158552	-0.213473
H	-0.640296	-1.778043	1.492831
H	-2.100110	0.202678	1.374680
H	-1.566147	2.948104	-1.210801
H	-4.995618	-0.689575	1.583532
H	-5.070082	-1.505901	0.000527
H	-7.001427	0.696695	0.961972
H	-7.383130	-1.033919	0.862216
H	-7.070666	-0.110982	-0.621613
H	-0.021750	1.470477	-2.204088

Aryl Substituent: 3-OH Intermediate 15 Conformer 4

O	-0.763814	-1.887711	1.881489
C	-0.531048	-1.043906	0.838139
C	0.513194	-1.248784	-0.077105
C	0.719693	-0.332369	-1.109208
C	-0.103453	0.783439	-1.238901
C	-1.176526	0.979126	-0.342733
C	-2.007359	2.157586	-0.548173
C	-3.270935	2.433473	-0.123439
C	-4.126388	1.469545	0.652071
O	-4.463302	0.408331	-0.088685
C	-5.306211	-0.607726	0.537929
C	-6.777106	-0.266156	0.365717
O	-4.464929	1.651824	1.808062
C	-3.804360	3.772155	-0.455409
C	-5.263827	4.114808	-0.046030
F	-6.104859	3.086679	-0.302357
F	-5.333037	4.393054	1.269296
F	-5.701857	5.182678	-0.722309
O	-3.176014	4.641959	-1.036489
C	-1.373784	0.060952	0.706177
H	-0.109296	-2.601987	1.876002
H	1.164528	-2.112125	0.033678
H	1.536036	-0.489221	-1.807733
H	0.065305	1.499824	-2.037024
H	-1.542975	2.947739	-1.136096

H	-5.029720	-0.690864	1.590852
H	-5.040367	-1.529508	0.017704
H	-7.026259	0.665726	0.880109
H	-7.386672	-1.069950	0.791302
H	-7.031446	-0.163961	-0.693464
H	-2.140512	0.210968	1.457029

Aryl Substituent: 3-OH Intermediate 15 Conformer 5

O	-0.613730	-1.873952	1.914222
C	-0.385183	-1.011064	0.885594
C	0.699907	-1.157565	0.007389
C	0.898472	-0.223830	-1.010602
C	0.026966	0.851628	-1.162924
C	-1.085897	0.987671	-0.305065
C	-1.965516	2.125589	-0.534521
C	-3.256189	2.336890	-0.156810
C	-4.091698	1.328431	0.583163
O	-4.349363	0.255973	-0.173024
C	-5.164904	-0.802407	0.418681
C	-6.643374	-0.529744	0.195903
O	-4.480689	1.488102	1.726487
C	-3.841537	3.649742	-0.503492
C	-5.332388	3.916418	-0.155089
F	-6.109245	2.848352	-0.447392
F	-5.470010	4.186067	1.156732
F	-5.795231	4.963330	-0.847447
O	-3.233915	4.554046	-1.053065
C	-1.275596	0.052673	0.730329
H	0.072622	-2.557612	1.925114
H	1.387973	-1.989494	0.135834
H	1.745996	-0.335248	-1.680090
H	0.188795	1.581746	-1.949981
H	-1.519046	2.941399	-1.100881
H	-4.922131	-0.879096	1.480372
H	-4.837644	-1.707386	-0.096026
H	-6.954201	0.386423	0.704836
H	-7.228533	-1.363915	0.596574
H	-6.864945	-0.433978	-0.871213
H	-2.075266	0.158612	1.453792

Aryl Substituent: 3-OH Intermediate 15 Conformer 6

O	1.811263	-0.375289	-2.182982
C	0.818869	-0.279765	-1.255139
C	0.657777	-1.210727	-0.220311
C	-0.375042	-1.045014	0.706822
C	-1.262880	0.021630	0.606223

C	-1.115616	0.956708	-0.441156
C	-1.983374	2.112637	-0.624968
C	-3.251664	2.340088	-0.186673
C	-4.068734	1.336381	0.579854
O	-4.369294	0.272333	-0.172161
C	-5.171588	-0.780996	0.445587
C	-6.654755	-0.490444	0.284538
O	-4.410579	1.493522	1.738548
C	-3.831325	3.666094	-0.492351
C	-5.304511	3.947818	-0.084726
F	-6.105589	2.893335	-0.360862
F	-5.390113	4.203332	1.234109
F	-5.778467	5.008920	-0.747364
O	-3.232587	4.568914	-1.053629
C	-0.053608	0.807073	-1.353387
H	2.350461	-1.160072	-2.003725
H	1.345412	-2.048340	-0.135554
H	-0.478061	-1.757173	1.520033
H	-2.026415	0.153105	1.363391
H	-1.549619	2.927302	-1.202927
H	-4.886736	-0.867704	1.495982
H	-4.875608	-1.686078	-0.087537
H	-6.933513	0.427327	0.809066
H	-7.233038	-1.319057	0.706189
H	-6.918975	-0.387569	-0.772132
H	0.090010	1.527907	-2.152658

Aryl Substituent: 3-OH Intermediate 16 Conformer 0

O	0.777387	-1.234679	1.908394
C	0.183204	-0.751604	0.780287
C	0.113902	-1.484341	-0.410420
C	-0.508744	-0.920338	-1.524003
C	-1.062985	0.359493	-1.463513
C	-0.990497	1.094947	-0.269407
C	-1.564339	2.504330	-0.166288
N	-1.118234	3.308672	-1.304229
C	-1.044931	4.655848	-1.367621
N	-1.361734	5.365988	-0.269251
S	-0.585929	5.430555	-2.825629
C	-3.129292	2.566782	-0.019084
C	-3.879712	1.642600	-0.973651
O	-3.882909	2.121638	-2.217815
C	-4.585410	1.338696	-3.237649
C	-6.061095	1.697681	-3.266096
O	-4.392390	0.598110	-0.614469

C	-3.574456	2.271379	1.405751
C	-5.090209	2.410856	1.735070
F	-5.800542	2.887287	0.693675
F	-5.602422	1.224437	2.094497
F	-5.239425	3.261821	2.764330
O	-2.833307	2.001791	2.321764
C	-0.364564	0.535947	0.847504
H	1.123214	-2.123277	1.737286
H	0.544129	-2.481472	-0.460890
H	-0.562189	-1.486037	-2.449605
H	-1.543243	0.769275	-2.346831
H	-1.160499	2.945980	0.749008
H	-0.888968	2.828636	-2.163181
H	-1.467742	4.946929	0.644222
H	-1.221122	6.364968	-0.295679
H	-3.444617	3.595831	-0.236989
H	-4.426361	0.278719	-3.030979
H	-4.082637	1.609513	-4.167048
H	-6.549591	1.428553	-2.325430
H	-6.549149	1.146912	-4.076772
H	-6.198491	2.768151	-3.444914
H	-0.299400	1.084824	1.780854

Aryl Substituent: 3-OH Intermediate 16 Conformer 1

O	-0.757490	-1.618952	-2.770668
C	-0.672561	-1.014906	-1.551601
C	-0.115623	-1.645571	-0.432435
C	-0.062228	-0.960505	0.781190
C	-0.554594	0.341645	0.891271
C	-1.116055	0.971875	-0.227249
C	-1.639524	2.398439	-0.099874
N	-1.111816	3.226610	-1.184304
C	-0.985112	4.571245	-1.191524
N	-1.326109	5.251668	-0.081124
S	-0.431821	5.381607	-2.596400
C	-3.206247	2.519300	-0.018383
C	-3.949653	1.661982	-1.038273
O	-3.866007	2.175552	-2.265507
C	-4.551745	1.456498	-3.342498
C	-6.003711	1.892423	-3.437489
O	-4.529650	0.633620	-0.738799
C	-3.725484	2.190057	1.373637
C	-5.247562	2.379966	1.643129
F	-5.890118	2.931595	0.594909
F	-5.823893	1.201577	1.924872

F	-5.408710	3.189925	2.702986
O	-3.037039	1.855066	2.308903
C	-1.172207	0.289116	-1.448622
H	-0.376642	-2.508178	-2.718547
H	0.270866	-2.658065	-0.517310
H	0.374280	-1.446346	1.649149
H	-0.509496	0.863836	1.840733
H	-1.261773	2.788393	0.849243
H	-0.856834	2.770114	-2.048714
H	-1.477687	4.803060	0.811916
H	-1.143493	6.244059	-0.062760
H	-3.470778	3.567623	-0.210265
H	-4.458451	0.384758	-3.157441
H	-3.987017	1.724145	-4.236567
H	-6.554263	1.626760	-2.530686
H	-6.475494	1.386930	-4.286392
H	-6.077072	2.972479	-3.594988
H	-1.594685	0.740701	-2.341317

Aryl Substituent: 3-OH Intermediate 16 Conformer 2

O	0.576071	-1.333493	2.061141
C	0.044305	-0.825494	0.913096
C	0.026225	-1.538339	-0.291557
C	-0.531828	-0.948398	-1.425742
C	-1.072893	0.337427	-1.372192
C	-1.053435	1.052059	-0.163364
C	-1.615015	2.466746	-0.065727
N	-1.097485	3.284517	-1.163030
C	-1.010033	4.631622	-1.200915
N	-1.395280	5.328072	-0.115115
S	-0.450977	5.424284	-2.613488
C	-3.184932	2.547119	-0.003158
C	-3.894056	1.649638	-1.013054
O	-3.821742	2.147818	-2.247197
C	-4.478849	1.391949	-3.316622
C	-5.943724	1.779457	-3.423329
O	-4.441480	0.607552	-0.700191
C	-3.709795	2.233583	1.390413
C	-5.239712	2.383851	1.639513
F	-5.886326	2.892890	0.572415
F	-5.783588	1.194886	1.940827
F	-5.437089	3.211360	2.679381
O	-3.021965	1.940857	2.340262
C	-0.491796	0.467219	0.974304
H	0.924774	-2.221208	1.891467

H	0.446293	-2.540037	-0.336810
H	-0.544798	-1.498428	-2.362190
H	-1.501400	0.768292	-2.271957
H	-1.257036	2.887051	0.878455
H	-0.807833	2.814150	-2.008893
H	-1.553667	4.896342	0.784924
H	-1.242020	6.325611	-0.116554
H	-3.475627	3.583501	-0.220351
H	-4.351326	0.326522	-3.116424
H	-3.919177	1.665454	-4.212102
H	-6.489348	1.508610	-2.515076
H	-6.395393	1.247616	-4.267061
H	-6.051184	2.854373	-3.595830
H	-0.467659	1.000069	1.918829

Aryl Substituent: 3-OH Intermediate 16 Conformer 3

O	0.647601	-1.308177	1.910640
C	0.038839	-0.800128	0.801473
C	-0.054952	-1.510385	-0.401171
C	-0.690732	-0.921517	-1.494201
C	-1.234426	0.360961	-1.401445
C	-1.138022	1.073545	-0.195172
C	-1.701252	2.484194	-0.056936
N	-1.268702	3.306383	-1.187394
C	-1.194236	4.654245	-1.229863
N	-1.498752	5.346995	-0.116479
S	-0.751528	5.452035	-2.680308
C	-3.263237	2.553247	0.116788
C	-4.035139	1.648013	-0.838884
O	-4.055451	2.144682	-2.075523
C	-4.780944	1.381682	-3.094515
C	-6.254339	1.751232	-3.092107
O	-4.549698	0.601917	-0.486221
C	-3.686937	2.238166	1.543902
C	-5.195914	2.383250	1.900025
F	-5.919100	2.884064	0.879279
F	-5.711408	1.193421	2.244759
F	-5.322634	3.215078	2.947455
O	-2.933128	1.947387	2.442932
C	-0.498657	0.489637	0.901201
H	0.986158	-2.194928	1.716848
H	0.366733	-2.509555	-0.476623
H	-0.763249	-1.469640	-2.429030
H	-1.725283	0.790288	-2.269530
H	-1.280301	2.906938	0.859634

H	-1.049174	2.839845	-2.056198
H	-1.584597	4.913212	0.792362
H	-1.351481	6.345378	-0.128030
H	-3.576344	3.586893	-0.081245
H	-4.625955	0.317718	-2.906196
H	-4.292369	1.662819	-4.028392
H	-6.728537	1.471119	-2.147346
H	-6.759907	1.216210	-3.902595
H	-6.387166	2.825193	-3.252466
H	-0.414781	1.020581	1.843374

Aryl Substituent: 3-OH Intermediate 16 Conformer 4

O	0.342748	-1.354214	2.125352
C	-0.066358	-0.783637	0.956093
C	0.065116	-1.421955	-0.282581
C	-0.368182	-0.768693	-1.437352
C	-0.931897	0.506398	-1.370978
C	-1.073458	1.140924	-0.127229
C	-1.690723	2.539910	-0.021813
N	-1.314574	3.412874	-1.136871
C	-0.112664	4.006635	-1.300045
N	0.833849	3.783154	-0.372062
S	0.171074	5.041539	-2.637550
C	-3.247702	2.555986	0.081675
C	-3.925556	1.658679	-0.953616
O	-3.836632	2.195717	-2.174313
C	-4.425185	1.446935	-3.290499
C	-5.899867	1.777302	-3.437529
O	-4.448786	0.595221	-0.685416
C	-3.742753	2.213533	1.478969
C	-5.279524	2.259735	1.728569
F	-5.952850	2.776974	0.682120
F	-5.749453	1.026516	1.971346
F	-5.529176	3.023541	2.803917
O	-3.035998	1.981921	2.430998
C	-0.632524	0.495829	1.032548
H	0.724952	-2.225537	1.943430
H	0.507421	-2.413571	-0.338370
H	-0.257788	-1.259506	-2.400171
H	-1.239297	1.008520	-2.282256
H	-1.337181	3.006901	0.902975
H	-1.976683	3.542953	-1.890867
H	0.762527	3.044402	0.314401
H	1.744014	4.195429	-0.511450
H	-3.575910	3.587793	-0.105101

H	-4.257702	0.382917	-3.114733
H	-3.845621	1.771636	-4.155365
H	-6.466104	1.452093	-2.560356
H	-6.296939	1.256497	-4.314922
H	-6.046770	2.852049	-3.578704
H	-0.730943	0.968899	2.003758

Aryl Substituent: 3-OH Intermediate 16 Conformer 5

O	-0.959547	-1.571723	-2.843702
C	-0.806435	-0.974736	-1.627886
C	-0.170535	-1.604853	-0.551473
C	-0.046620	-0.926594	0.660810
C	-0.546424	0.368540	0.811710
C	-1.188134	0.997766	-0.263366
C	-1.718692	2.417401	-0.093339
N	-1.278579	3.255437	-1.209023
C	-1.175632	4.602098	-1.221933
N	-1.450074	5.275285	-0.088879
S	-0.735405	5.423475	-2.659827
C	-3.277089	2.519010	0.099408
C	-4.080174	1.652295	-0.866343
O	-4.097396	2.171249	-2.093854
C	-4.854251	1.447309	-3.118660
C	-6.316665	1.857511	-3.093774
O	-4.619291	0.613698	-0.528293
C	-3.693452	2.181165	1.523252
C	-5.194350	2.355492	1.899521
F	-5.917503	2.893145	0.897726
F	-5.734157	1.171751	2.227906
F	-5.288639	3.169713	2.964223
O	-2.938790	1.848948	2.406902
C	-1.316466	0.321395	-1.482864
H	-0.557040	-2.452729	-2.824606
H	0.222727	-2.611532	-0.668559
H	0.451338	-1.412128	1.495200
H	-0.445132	0.885737	1.759528
H	-1.278838	2.809106	0.828063
H	-1.079310	2.805211	-2.091178
H	-1.529225	4.822333	0.811262
H	-1.280372	6.270108	-0.079714
H	-3.568031	3.563503	-0.073099
H	-4.727598	0.376152	-2.950924
H	-4.367678	1.731601	-4.052592
H	-6.788949	1.573868	-2.149120
H	-6.845054	1.351150	-3.908112

H	-6.420991	2.937403	-3.234037
H	-1.803246	0.772438	-2.342454

Aryl Substituent: 3-OH Intermediate 16 Conformer 6

O	-0.471650	-1.409666	-2.781436
C	-0.531768	-0.864412	-1.532851
C	-0.088058	-1.544437	-0.391193
C	-0.170541	-0.918985	0.852338
C	-0.694658	0.370646	0.971907
C	-1.147042	1.046878	-0.170408
C	-1.719794	2.462563	-0.044192
N	-1.322772	3.339462	-1.149145
C	-0.103378	3.896210	-1.312067
N	0.843507	3.621138	-0.397861
S	0.202472	4.950515	-2.629467
C	-3.275037	2.528326	0.069547
C	-3.990338	1.679846	-0.981492
O	-3.889044	2.240333	-2.190790
C	-4.513330	1.540114	-3.318991
C	-5.975037	1.930306	-3.448017
O	-4.550766	0.630444	-0.733820
C	-3.773148	2.168877	1.461477
C	-5.305615	2.263941	1.723143
F	-5.966626	2.830826	0.694650
F	-5.818143	1.042553	1.938736
F	-5.520978	3.008937	2.818845
O	-3.069050	1.887726	2.401941
C	-1.054797	0.429097	-1.422713
H	-0.083685	-2.295814	-2.728975
H	0.322372	-2.546943	-0.483274
H	0.183162	-1.441342	1.736709
H	-0.760243	0.845961	1.944567
H	-1.345294	2.903386	0.885223
H	-1.988346	3.509971	-1.891857
H	0.756712	2.860622	0.262844
H	1.767382	3.999926	-0.542448
H	-3.568184	3.575178	-0.090857
H	-4.386538	0.466667	-3.168184
H	-3.927215	1.861338	-4.180732
H	-6.547735	1.607982	-2.573964
H	-6.397613	1.444475	-4.333512
H	-6.081130	3.012750	-3.564789
H	-1.363855	0.941904	-2.327789

Aryl Substituent: 3-OH Intermediate 17 Conformer 0

H	1.088593	0.023227	0.072408

O	2.053986	-0.029165	0.087962
H	2.324421	0.858938	-0.182370

Aryl Substituent: 3-OH Intermediate 1 Conformer 0

N	1.192197	0.096666	-0.050839
C	2.534267	0.063096	-0.047970
N	3.176314	1.239079	-0.132486
S	3.393383	-1.421105	0.059662
H	0.674750	0.963242	-0.113756
H	0.675230	-0.766096	0.012784
H	2.682879	2.119532	-0.196154
H	4.183981	1.253586	-0.134241

Aryl Substituent: 3-OH Intermediate 2 Conformer 0

O	2.269238	0.031071	0.011599
C	0.907004	-0.003726	0.005095
C	0.179530	-1.201582	0.001221
C	-1.219741	-1.174205	-0.004874
C	-1.904695	0.037177	-0.006567
C	-1.172540	1.236901	-0.002312
C	-1.861559	2.542791	-0.002744
H	-1.187269	3.423647	0.000491
O	-3.076140	2.689387	-0.006559
C	0.227651	1.219375	0.003231
H	2.618520	-0.872584	0.015632
H	0.709081	-2.151325	0.002617
H	-1.767923	-2.111873	-0.008078
H	-2.988943	0.074555	-0.010606
H	0.794789	2.146393	0.006853

Aryl Substituent: 3-OH Intermediate 2 Conformer 1

O	2.269237	0.031072	0.011404
C	0.907002	-0.003726	0.005010
C	0.179531	-1.201582	0.001246
C	-1.219739	-1.174205	-0.004747
C	-1.904693	0.037178	-0.006441
C	-1.172539	1.236902	-0.002286
C	-1.861559	2.542791	-0.002719
H	-1.187272	3.423650	0.000441
O	-3.076141	2.689384	-0.006453
C	0.227651	1.219375	0.003152
H	2.618517	-0.872585	0.015424
H	0.709079	-2.151326	0.002645
H	-1.767925	-2.111872	-0.007869
H	-2.988942	0.074551	-0.010402
H	0.794793	2.146392	0.006694

Aryl Substituent: 3-OH Intermediate 2 Conformer 2

O	2.280138	0.002368	0.011396
C	0.917456	-0.008046	0.005201
C	0.174220	-1.200828	0.001213
C	-1.220924	-1.158928	-0.004790
C	-1.889251	0.065875	-0.006215
C	-1.143803	1.254924	-0.001829
C	-1.856108	2.547968	-0.002237
H	-2.962123	2.466356	-0.004874
O	-1.313319	3.644608	0.000728
C	0.257905	1.221488	0.003666
H	2.613626	-0.907196	0.014907
H	0.694036	-2.155993	0.002362
H	-1.782051	-2.088653	-0.008141
H	-2.975036	0.106126	-0.010382
H	0.825335	2.146531	0.007296

Aryl Substituent: 3-OH Intermediate 2 Conformer 3

O	2.268286	0.028579	0.082965
C	0.906701	-0.004658	0.037057
C	0.179015	-1.201691	-0.005900
C	-1.219591	-1.173113	-0.051310
C	-1.903549	0.038800	-0.053868
C	-1.171251	1.237656	-0.010570
C	-1.859819	2.543972	-0.010248
H	-1.185906	3.424360	0.025840
O	-3.073833	2.691075	-0.045886
C	0.228304	1.218976	0.034410
H	2.616817	-0.875351	0.084558
H	0.708200	-2.151636	-0.003943
H	-1.767520	-2.110335	-0.084448
H	-2.987199	0.077623	-0.088050
H	0.795346	2.145442	0.068192

Aryl Substituent: 3-OH Intermediate 2 Conformer 4

O	2.268829	0.028732	-0.060395
C	0.906841	-0.004614	-0.027222
C	0.178889	-1.201724	0.008313
C	-1.220065	-1.173285	0.041678
C	-1.904047	0.038600	0.040838
C	-1.171473	1.237557	0.005761
C	-1.860025	2.543881	0.004913
H	-1.185937	3.424372	-0.024534
O	-3.074241	2.690875	0.033704
C	0.228399	1.218994	-0.028466
H	2.617614	-0.875076	-0.052558
H	0.708147	-2.151638	0.009245

H	-1.768171	-2.110598	0.068657
H	-2.987924	0.077370	0.067104
H	0.795662	2.145553	-0.055241

Aryl Substituent: 3-OH Intermediate 2 Conformer 5

O	2.278928	0.003052	0.076029
C	0.916806	-0.007629	0.034018
C	0.175105	-1.200376	-0.017311
C	-1.219513	-1.159110	-0.058289
C	-1.888776	0.065124	-0.048199
C	-1.144919	1.254108	0.003082
C	-1.858337	2.546691	0.014171
H	-2.963825	2.465003	-0.019025
O	-1.316523	3.643007	0.056389
C	0.256279	1.221362	0.043995
H	2.613089	-0.906208	0.068025
H	0.696092	-2.154869	-0.025131
H	-1.779102	-2.088907	-0.097958
H	-2.974130	0.105090	-0.079144
H	0.822028	2.146561	0.083847

Aryl Substituent: 3-OH Intermediate 2 Conformer 6

O	2.279534	0.003015	-0.053216
C	0.917051	-0.007675	-0.023656
C	0.174885	-1.200463	0.019526
C	-1.220025	-1.159168	0.048841
C	-1.889076	0.065146	0.036161
C	-1.144764	1.254177	-0.006651
C	-1.857961	2.546880	-0.018900
H	-2.963610	2.465328	0.008669
O	-1.315765	3.643218	-0.055623
C	0.256718	1.221392	-0.036677
H	2.613755	-0.906130	-0.037202
H	0.695649	-2.155006	0.028770
H	-1.779967	-2.089010	0.081785
H	-2.974649	0.105147	0.059104
H	0.822825	2.146648	-0.069329

Aryl Substituent: 3-OH Intermediate 3 Conformer 0

O	2.194007	0.044368	0.692703
C	0.895731	-0.003640	0.275159
C	0.291373	-1.182671	-0.174926
C	-1.044418	-1.154937	-0.580788
C	-1.777185	0.032174	-0.548564
C	-1.162358	1.214026	-0.105668
C	-1.962371	2.510099	-0.041380
N	-2.638682	2.728209	-1.318384

C	-3.592643	3.666992	-1.533828
N	-3.792974	4.561398	-0.549729
S	-4.503466	3.679681	-2.982938
H	-2.724969	2.430579	0.743626
O	-1.199639	3.642614	0.341959
C	0.167510	1.193229	0.314066
H	2.588001	-0.837982	0.624517
H	0.860353	-2.108700	-0.201440
H	-1.516740	-2.071396	-0.923131
H	-2.818571	0.035024	-0.856834
H	-2.447684	2.095015	-2.082741
H	-3.067933	4.715980	0.142610
H	-4.462074	5.299559	-0.710088
H	-0.546903	3.819969	-0.354631
H	0.649635	2.093409	0.678429

Aryl Substituent: 3-OH Intermediate 3 Conformer 1

O	2.247160	0.201321	0.172307
C	0.899369	0.049390	0.023367
C	0.301253	-1.194404	-0.207273
C	-1.086339	-1.271335	-0.343437
C	-1.878264	-0.125526	-0.258040
C	-1.271558	1.120902	-0.036008
C	-2.129261	2.375229	0.085393
N	-3.051111	2.445139	-1.046916
C	-4.087272	3.313622	-1.143450
N	-4.154223	4.281715	-0.212566
S	-5.257004	3.152764	-2.382999
H	-2.719497	2.326762	1.009233
O	-1.384557	3.574346	0.221230
C	0.112569	1.205757	0.113542
H	2.680232	-0.661610	0.093890
H	0.915808	-2.088790	-0.274637
H	-1.552194	-2.237525	-0.515583
H	-2.957205	-0.203811	-0.354710
H	-2.972014	1.753944	-1.780151
H	-3.321547	4.532616	0.309589
H	-4.886518	4.970694	-0.298113
H	-0.894115	3.722132	-0.603698
H	0.593889	2.157970	0.306129

Aryl Substituent: 3-OH Intermediate 3 Conformer 2

O	2.193438	0.074418	0.694565
C	0.897085	0.005550	0.274029
C	0.313419	-1.182433	-0.179834
C	-1.021707	-1.175945	-0.588797

C	-1.774114	-0.001189	-0.555945
C	-1.180029	1.189733	-0.109117
C	-2.001534	2.472214	-0.044179
N	-2.676267	2.683099	-1.323136
C	-3.644509	3.606821	-1.539881
N	-3.863363	4.494996	-0.554022
S	-4.549418	3.608837	-2.992705
H	-2.765630	2.377542	0.737694
O	-1.259177	3.616043	0.345567
C	0.149001	1.190089	0.313683
H	2.602450	-0.800970	0.625190
H	0.897688	-2.098891	-0.207033
H	-1.478045	-2.099347	-0.934166
H	-2.814653	-0.014909	-0.866788
H	-2.472203	2.055048	-2.088372
H	-3.143550	4.659563	0.141485
H	-4.542962	5.223216	-0.715778
H	-0.606701	3.806345	-0.347839
H	0.615581	2.097170	0.681178

Aryl Substituent: 3-OH Intermediate 3 Conformer 3

O	2.236678	-0.100488	0.219985
C	0.879900	-0.096264	0.076618
C	0.137181	-1.271047	-0.103089
C	-1.246582	-1.189066	-0.253037
C	-1.900114	0.046018	-0.221640
C	-1.156537	1.215352	-0.028858
C	-1.841450	2.576907	-0.012848
N	-1.410250	3.327509	1.165393
C	-1.662887	4.642633	1.374573
N	-2.513074	5.238216	0.519154
S	-0.907116	5.491375	2.654628
H	-1.551960	3.143173	-0.906992
O	-3.255770	2.511470	-0.095813
C	0.234249	1.144787	0.112443
H	2.563944	-1.011056	0.173497
H	0.642946	-2.233036	-0.128355
H	-1.821600	-2.099469	-0.397155
H	-2.974611	0.102095	-0.352539
H	-0.817511	2.868475	1.843308
H	-3.158178	4.670865	-0.020290
H	-2.757549	6.202171	0.690079
H	-3.587722	2.084920	0.710759
H	0.834612	2.040661	0.243076

Aryl Substituent: 3-OH Intermediate 3 Conformer 4

O	2.109046	-0.127655	0.613455
C	0.798377	-0.115292	0.234678
C	0.146321	-1.252000	-0.261609
C	-1.193132	-1.161879	-0.637941
C	-1.892252	0.043004	-0.522895
C	-1.240783	1.172503	-0.015976
C	-1.971643	2.505529	0.095973
N	-1.769287	3.064311	1.431386
C	-2.108031	4.324661	1.798092
N	-2.833887	5.035614	0.917183
S	-1.604759	4.968982	3.301631
H	-1.562502	3.210778	-0.638393
O	-3.349090	2.435932	-0.234079
C	0.106727	1.095189	0.355427
H	2.473966	-1.015774	0.485091
H	0.687672	-2.190256	-0.354059
H	-1.697277	-2.042088	-1.027030
H	-2.929652	0.106615	-0.830651
H	-1.280164	2.509713	2.120368
H	-3.357005	4.553053	0.194423
H	-3.140130	5.957302	1.190794
H	-3.792227	1.878473	0.425751
H	0.639313	1.963684	0.732381

Aryl Substituent: 3-OH Intermediate 3 Conformer 5

O	2.072398	0.153390	0.619601
C	0.767956	0.028569	0.239824
C	0.232319	-1.176665	-0.233916
C	-1.108665	-1.226343	-0.612662
C	-1.923268	-0.094121	-0.521681
C	-1.387619	1.103680	-0.036306
C	-2.247011	2.359465	0.051421
N	-2.103244	2.958425	1.377064
C	-2.559390	4.187860	1.719947
N	-3.341679	4.813769	0.822482
S	-2.127713	4.901790	3.214435
H	-1.907219	3.088950	-0.694483
O	-3.610221	2.148676	-0.277828
C	-0.040082	1.166935	0.336810
H	2.525095	-0.695274	0.505106
H	0.863661	-2.058787	-0.307252
H	-1.522833	-2.159300	-0.984647
H	-2.961104	-0.138738	-0.831178
H	-1.568916	2.463302	2.077815
H	-3.815577	4.272074	0.107746

H	-3.734227	5.707204	1.079252
H	-3.997744	1.562498	0.392114
H	0.403083	2.090739	0.697636

Aryl Substituent: 3-OH Intermediate 3 Conformer 6

O	2.196593	-0.137601	0.648229
C	0.891908	-0.077705	0.252815
C	0.186401	-1.200932	-0.192751
C	-1.149229	-1.062086	-0.575808
C	-1.782627	0.180282	-0.524900
C	-1.066849	1.305391	-0.085847
C	-1.757374	2.661521	0.001281
N	-2.441302	2.941779	-1.259546
C	-3.316318	3.959030	-1.451433
N	-3.424741	4.858548	-0.457320
S	-4.247163	4.060358	-2.884108
H	-2.507057	2.639213	0.802269
O	-0.896861	3.725962	0.373239
C	0.263833	1.173895	0.310664
H	2.516618	-1.048336	0.567911
H	0.677410	-2.170196	-0.233006
H	-1.700054	-1.934929	-0.914818
H	-2.825230	0.269646	-0.815569
H	-2.313273	2.304188	-2.033352
H	-2.675707	4.947591	0.220938
H	-4.030013	5.652857	-0.602518
H	-0.245090	3.852159	-0.335285
H	0.823725	2.029367	0.671413

Aryl Substituent: 3-OH Intermediate 4 Conformer 0

O	2.232495	0.084688	0.047152
C	0.871411	0.027020	0.024592
C	0.163759	-1.181750	-0.004195
C	-1.235944	-1.172683	-0.025413
C	-1.940095	0.026314	-0.017884
C	-1.229646	1.242866	0.009560
C	-1.909973	2.534912	0.012904
H	-1.271193	3.425576	0.029087
N	-3.193276	2.644191	0.002024
C	-3.781002	3.911027	-0.044350
N	-4.951572	3.944385	0.596484
S	-3.158238	5.258252	-0.872471
C	0.173834	1.239304	0.032157
H	2.597145	-0.812842	0.045821
H	0.707272	-2.123439	-0.008830
H	-1.770801	-2.117671	-0.046457

H	-3.024237	0.041294	-0.029935
H	-5.291897	3.117448	1.070836
H	-5.494501	4.794754	0.638184
H	0.729458	2.172356	0.054732

Aryl Substituent: 3-OH Intermediate 4 Conformer 1

O	2.231713	0.085999	0.050824
C	0.870652	0.027726	0.026423
C	0.163573	-1.181377	-0.005226
C	-1.236133	-1.173041	-0.028269
C	-1.940903	0.025556	-0.019785
C	-1.231066	1.242474	0.010602
C	-1.912010	2.534015	0.015343
H	-1.273880	3.425070	0.034469
N	-3.195443	2.642590	0.002024
C	-3.783238	3.909845	-0.042380
N	-4.961192	3.937157	0.585162
S	-3.152660	5.264455	-0.852410
C	0.172465	1.239659	0.035046
H	2.596783	-0.811358	0.048914
H	0.707569	-2.122785	-0.010654
H	-1.770417	-2.118294	-0.051495
H	-3.025015	0.040101	-0.033229
H	-5.305039	3.106565	1.050547
H	-5.504304	4.787279	0.629563
H	0.727545	2.172966	0.059932

Aryl Substituent: 3-OH Intermediate 4 Conformer 2

O	2.237670	0.076599	0.005829
C	0.876092	0.022234	0.008547
C	0.165750	-1.184542	0.047581
C	-1.234008	-1.172277	0.047635
C	-1.935242	0.028049	0.009885
C	-1.221939	1.242431	-0.026874
C	-1.899714	2.535745	-0.057802
H	-1.258118	3.424417	-0.075273
N	-3.182534	2.647805	-0.068647
C	-3.769522	3.914670	-0.045113
N	-4.912563	3.953806	-0.733461
S	-3.176820	5.255821	0.814348
C	0.181673	1.235703	-0.029460
H	2.599526	-0.821411	0.040705
H	0.706225	-2.127508	0.075580
H	-1.772048	-2.115244	0.076643
H	-3.019444	0.044225	0.006952
H	-5.236698	3.129484	-1.223103

H	-5.451761	4.805594	-0.790603
H	0.740075	2.166898	-0.058470

Aryl Substituent: 3-OH Intermediate 4 Conformer 3

O	2.209483	0.093471	0.299609
C	0.858382	0.031096	0.134770
C	0.167613	-1.178829	-0.012436
C	-1.222572	-1.175011	-0.176325
C	-1.933810	0.019794	-0.195235
C	-1.240173	1.237458	-0.049098
C	-1.927988	2.525380	-0.066961
H	-1.302137	3.417625	0.050863
N	-3.204009	2.629190	-0.208380
C	-3.794795	3.894093	-0.266040
N	-5.028579	3.894791	0.243822
S	-3.098221	5.278388	-0.964242
C	0.153701	1.239090	0.116951
H	2.580328	-0.801529	0.292815
H	0.716165	-2.117467	0.002789
H	-1.745330	-2.120417	-0.287887
H	-3.011114	0.029418	-0.317843
H	-5.410857	3.046951	0.643329
H	-5.580095	4.740515	0.260125
H	0.696607	2.172894	0.231574

Aryl Substituent: 3-OH Intermediate 4 Conformer 4

O	2.256862	-0.033494	0.051418
C	0.894415	-0.019205	0.029882
C	0.130622	-1.199231	0.012365
C	-1.262765	-1.129696	-0.010786
C	-1.907554	0.106660	-0.016515
C	-1.143930	1.288181	0.003145
C	-1.846770	2.567649	0.006684
H	-2.941853	2.523601	-0.005269
N	-1.231935	3.699659	0.020281
C	-1.965900	4.887665	0.074647
N	-1.338279	5.891323	-0.542444
S	-3.445936	5.101336	0.882920
C	0.259921	1.222567	0.024494
H	2.573796	-0.948908	0.059229
H	0.633587	-2.163249	0.015106
H	-1.842504	-2.047653	-0.024797
H	-2.991849	0.164698	-0.033596
H	-0.453875	5.724868	-1.005867
H	-1.746869	6.814072	-0.576804
H	0.849516	2.132857	0.035508

Aryl Substituent: 3-OH Intermediate 4 Conformer 5

O	2.256019	-0.027311	-0.033302
C	0.893406	-0.018448	-0.011579
C	0.134033	-1.201276	-0.024228
C	-1.259489	-1.136885	0.000709
C	-1.908648	0.096794	0.039011
C	-1.149266	1.280908	0.050527
C	-1.856357	2.557787	0.084814
H	-2.951274	2.508847	0.097157
N	-1.246091	3.691957	0.104945
C	-1.983921	4.878050	0.088781
N	-1.374069	5.857479	0.760139
S	-3.448675	5.117224	-0.739658
C	0.254752	1.220532	0.026705
H	2.575975	-0.941471	-0.054508
H	0.639001	-2.163810	-0.052595
H	-1.836713	-2.056491	-0.009109
H	-2.993185	0.149725	0.058827
H	-0.496167	5.676951	1.230414
H	-1.787768	6.776411	0.822704
H	0.842139	2.132227	0.040645

Aryl Substituent: 3-OH Intermediate 4 Conformer 6

O	2.223715	0.024486	-0.332770
C	0.875361	0.003515	-0.136471
C	0.143559	-1.186271	-0.028389
C	-1.240818	-1.140656	0.173993
C	-1.906075	0.076573	0.269826
C	-1.171902	1.274171	0.159196
C	-1.813084	2.583161	0.243745
H	-1.159270	3.457180	0.142478
N	-3.079215	2.725115	0.432516
C	-3.637664	4.005888	0.454956
N	-4.694162	4.077383	1.267936
S	-3.119904	5.326331	-0.481157
C	0.216972	1.233982	-0.041770
H	2.561009	-0.882736	-0.376262
H	0.656524	-2.142170	-0.101339
H	-1.794529	-2.071154	0.257821
H	-2.977586	0.119257	0.430351
H	-4.979662	3.263556	1.797886
H	-5.207451	4.940410	1.374203
H	0.790986	2.152178	-0.126551

Aryl Substituent: 3-OH Intermediate 5 Conformer 0

O	1.269762	0.274755	-0.162930

C	2.575194	-0.025113	-0.083189
C	3.453875	1.112394	0.411920
F	2.815463	1.782122	1.403736
F	4.636014	0.693536	0.882582
F	3.690271	2.003451	-0.573192
C	3.133824	-1.191476	-0.458446
C	2.469982	-2.386563	-1.003414
O	1.139895	-2.258917	-1.156065
C	0.422869	-3.405127	-1.694271
C	0.445966	-3.405612	-3.214948
O	3.094266	-3.402430	-1.283253
H	1.080865	1.095645	0.321096
H	4.206394	-1.290536	-0.354870
H	-0.592606	-3.287249	-1.310617
H	0.860702	-4.318146	-1.285383
H	0.037775	-2.470587	-3.610877
H	-0.166345	-4.233586	-3.587650
H	1.464834	-3.534561	-3.590228

Aryl Substituent: 3-OH Intermediate 5 Conformer 1

O	1.255965	0.305187	-0.242715
C	2.562968	0.029441	-0.170452
C	3.454110	1.144985	0.373573
F	2.706800	2.231112	0.683634
F	4.105696	0.764257	1.486402
F	4.378295	1.527037	-0.525304
C	3.144104	-1.130483	-0.536121
C	2.510056	-2.334991	-1.089340
O	1.173585	-2.242969	-1.220352
C	0.483408	-3.396448	-1.777160
C	0.479524	-3.355032	-3.297468
O	3.157775	-3.328949	-1.394917
H	1.089865	1.206423	0.081345
H	4.218688	-1.208832	-0.422763
H	-0.528258	-3.321984	-1.373050
H	0.957357	-4.305744	-1.401641
H	0.031930	-2.424893	-3.660995
H	-0.109102	-4.194141	-3.683227
H	1.495833	-3.437175	-3.692549

Aryl Substituent: 3-OH Intermediate 5 Conformer 2

O	1.293133	0.148510	0.059594
C	2.609879	-0.108853	0.055650
C	3.473897	1.015443	0.603832
F	2.860164	1.582195	1.672102
F	4.688418	0.601783	0.988907

F	3.637963	1.989101	-0.315728
C	3.188233	-1.219882	-0.438749
C	2.540183	-2.390188	-1.051529
O	1.201823	-2.295381	-1.145397
C	0.499964	-3.421364	-1.742873
C	0.466628	-3.309794	-3.259231
O	3.183454	-3.360102	-1.433080
H	1.100917	0.922569	0.614265
H	4.267085	-1.287895	-0.389706
H	-0.503667	-3.365243	-1.316431
H	0.979432	-4.347467	-1.418776
H	0.011309	-2.364697	-3.570847
H	-0.129086	-4.131314	-3.671082
H	1.474869	-3.372221	-3.677621

Aryl Substituent: 3-OH Intermediate 5 Conformer 3

O	1.417395	0.540426	-0.805084
C	2.635113	0.130754	-0.418022
C	3.548650	1.243520	0.070178
F	2.830606	2.135330	0.797380
F	4.556319	0.796775	0.831324
F	4.080331	1.922191	-0.967628
C	3.096060	-1.132234	-0.493185
C	2.394911	-2.331737	-0.978116
O	1.133162	-2.107300	-1.386609
C	0.384125	-3.253302	-1.879138
C	0.651875	-3.492751	-3.356895
O	2.931109	-3.432697	-0.993833
H	1.255590	1.450213	-0.505648
H	4.109805	-1.316839	-0.162699
H	-0.659358	-2.985895	-1.700298
H	0.641791	-4.127404	-1.277430
H	0.421105	-2.598829	-3.944498
H	0.018580	-4.312011	-3.713434
H	1.696932	-3.767011	-3.524965

Aryl Substituent: 3-OH Intermediate 5 Conformer 4

O	1.250930	0.265850	-0.141755
C	2.567209	0.028332	-0.157267
C	3.457270	1.152225	0.371225
F	2.698600	2.199419	0.773663
F	4.194843	0.752167	1.421826
F	4.305595	1.599618	-0.571289
C	3.157867	-1.100360	-0.597570
C	2.525888	-2.305562	-1.151675
O	1.183187	-2.241279	-1.222071

C	0.494233	-3.403308	-1.761850
C	0.237927	-4.444723	-0.683552
O	3.181358	-3.273275	-1.518884
H	1.078376	1.148563	0.226845
H	4.238965	-1.151728	-0.549269
H	1.086349	-3.813082	-2.582850
H	-0.440250	-2.999111	-2.156473
H	1.178406	-4.851848	-0.302438
H	-0.348791	-5.267885	-1.104931
H	-0.324963	-4.012311	0.149414

Aryl Substituent: 3-OH Intermediate 5 Conformer 5

O	1.302530	0.141352	0.146293
C	2.622167	-0.089549	0.067905
C	3.489870	1.030960	0.616457
F	2.946575	1.506312	1.764429
F	4.745346	0.643208	0.876014
F	3.543341	2.065328	-0.248788
C	3.194199	-1.172421	-0.491935
C	2.536349	-2.337204	-1.105273
O	1.193790	-2.262666	-1.133722
C	0.481156	-3.391300	-1.712771
C	0.243791	-4.482041	-0.679932
O	3.176652	-3.281504	-1.550659
H	1.123218	0.896485	0.730427
H	4.275104	-1.221495	-0.499465
H	1.048334	-3.764220	-2.568157
H	-0.459479	-2.960757	-2.062263
H	1.190282	-4.912846	-0.341951
H	-0.358796	-5.280478	-1.125700
H	-0.295428	-4.087063	0.186591

Aryl Substituent: 3-OH Intermediate 5 Conformer 6

O	1.394979	0.375876	-0.744695
C	2.660753	0.069206	-0.420932
C	3.488613	1.249227	0.060179
F	2.740603	2.022656	0.886055
F	4.594889	0.880869	0.719026
F	3.860684	2.029589	-0.976206
C	3.227736	-1.145466	-0.547558
C	2.612576	-2.393923	-1.025670
O	1.330359	-2.266214	-1.411177
C	0.658178	-3.469007	-1.878947
C	0.068146	-4.256952	-0.719718
O	3.234922	-3.448044	-1.064747
H	1.164824	1.259649	-0.413978

H	4.266634	-1.246192	-0.262302
H	1.368556	-4.068568	-2.451946
H	-0.121340	-3.095688	-2.546214
H	0.856231	-4.631896	-0.060904
H	-0.491806	-5.113517	-1.109716
H	-0.617036	-3.635506	-0.134951

Aryl Substituent: 3-OH Intermediate 6 Conformer 0

S	0.337289	1.497801	-1.435599
C	2.012157	1.753887	-1.179677
N	2.469349	2.841108	-0.536227
N	2.918509	0.849364	-1.613906
C	4.350003	0.878674	-1.308899
C	4.959889	-0.539031	-1.511148
C	4.480848	-1.505172	-0.430653
C	5.004011	-2.969582	-0.484601
F	3.974679	-3.823126	-0.611769
F	5.849621	-3.170934	-1.516261
F	5.653768	-3.256577	0.652966
O	3.767641	-1.203706	0.496514
C	4.718024	-1.099449	-2.917166
O	3.535027	-1.742216	-2.948119
C	3.144662	-2.408122	-4.196025
C	3.722252	-3.811571	-4.248168
O	5.464504	-0.957348	-3.858452
C	5.121243	1.941647	-2.091554
C	4.730585	2.319915	-3.380470
C	5.471978	3.277279	-4.082295
O	5.132109	3.683918	-5.338767
C	6.606531	3.858526	-3.499361
C	6.986343	3.479547	-2.213589
C	6.249062	2.527899	-1.502573
H	1.804647	3.549311	-0.263009
H	3.447775	3.097212	-0.528303
H	2.527251	0.022729	-2.047015
H	4.466718	1.088887	-0.238961
H	6.046243	-0.448140	-1.401369
H	3.478091	-1.790484	-5.031582
H	2.054933	-2.415439	-4.157075
H	4.813876	-3.788005	-4.306445
H	3.340499	-4.322907	-5.137518
H	3.427188	-4.384091	-3.364350
H	3.846166	1.884249	-3.837881
H	4.334045	3.216752	-5.627361
H	7.167087	4.601770	-4.057925

H	7.859965	3.936278	-1.757353
H	6.548431	2.244147	-0.497054

Aryl Substituent: 3-OH Intermediate 6 Conformer 1

S	0.462906	0.569220	-1.749564
C	2.030165	1.080899	-1.283419
N	2.231559	2.266064	-0.684842
N	3.101181	0.279624	-1.487361
C	4.487058	0.611942	-1.160824
C	5.304858	-0.711660	-1.010609
C	4.763259	-1.485038	0.203438
C	4.280233	-2.950997	0.047987
F	3.111850	-2.972014	-0.621706
F	5.178972	-3.680214	-0.644513
F	4.099687	-3.520184	1.240483
O	4.694161	-0.968309	1.292620
C	5.366438	-1.496150	-2.318761
O	6.617200	-1.804396	-2.643670
C	6.831943	-2.565456	-3.877047
C	6.988940	-1.633140	-5.065694
O	4.377391	-1.784544	-2.975486
C	5.153744	1.583700	-2.133296
C	4.789114	1.621301	-3.483946
C	5.438516	2.501049	-4.358439
O	5.119198	2.579238	-5.681867
C	6.454731	3.342892	-3.887165
C	6.809612	3.300728	-2.540289
C	6.164453	2.429074	-1.658369
H	1.438460	2.873360	-0.543851
H	3.149717	2.662001	-0.538501
H	2.944343	-0.547906	-2.052285
H	4.504167	1.055920	-0.159928
H	6.323925	-0.429122	-0.731117
H	5.996894	-3.256290	-4.004956
H	7.745567	-3.127872	-3.679456
H	6.068457	-1.073779	-5.254046
H	7.218847	-2.226505	-5.956603
H	7.807384	-0.926186	-4.901622
H	3.997170	0.979422	-3.859958
H	4.390936	1.971546	-5.878629
H	6.944438	4.020935	-4.579272
H	7.590909	3.959728	-2.172481
H	6.443636	2.409041	-0.608174

Aryl Substituent: 3-OH Intermediate 6 Conformer 2

S	0.375164	0.936834	-1.405698

C	2.010701	1.310613	-1.055725
N	2.350333	2.448025	-0.428162
N	2.996523	0.445079	-1.385780
C	4.426671	0.659348	-1.165628
C	5.149577	-0.726455	-1.155929
C	4.656271	-1.524945	0.062447
C	4.090562	-2.958518	-0.113715
F	2.868483	-2.894659	-0.675151
F	4.888690	-3.694155	-0.915500
F	3.988020	-3.576159	1.063879
O	4.692281	-1.054100	1.173863
C	5.032391	-1.435427	-2.502492
O	6.223508	-1.768527	-2.986471
C	6.261182	-2.466197	-4.275373
C	6.119019	-3.965741	-4.079780
O	3.964280	-1.638095	-3.060763
C	5.078914	1.637035	-2.141610
C	4.633167	1.745865	-3.463973
C	5.266821	2.631378	-4.343961
O	4.870064	2.777522	-5.640033
C	6.348260	3.408185	-3.906596
C	6.784821	3.294603	-2.588298
C	6.156386	2.415962	-1.701015
H	1.620082	3.101697	-0.189331
H	3.305297	2.765853	-0.338314
H	2.731804	-0.340450	-1.970541
H	4.561405	1.042552	-0.148619
H	6.208381	-0.535517	-0.957694
H	7.236039	-2.199453	-4.685474
H	5.472294	-2.058814	-4.909937
H	6.900228	-4.348680	-3.416742
H	6.216248	-4.462332	-5.050637
H	5.141853	-4.218442	-3.660110
H	3.791683	1.154292	-3.814089
H	4.116454	2.195229	-5.817633
H	6.824674	4.091716	-4.602593
H	7.617829	3.902378	-2.246759
H	6.500151	2.339199	-0.672766

Aryl Substituent: 3-OH Intermediate 6 Conformer 3

S	0.486685	0.531411	-1.889486
C	2.039964	1.038497	-1.375166
N	2.228862	2.225530	-0.779324
N	3.113774	0.231112	-1.534295
C	4.486350	0.561746	-1.159121

C	5.298485	-0.759262	-0.956778
C	4.721090	-1.499340	0.258125
C	4.115742	-2.916870	0.091213
F	2.968632	-2.838275	-0.610095
F	4.967967	-3.718482	-0.581847
F	3.852943	-3.466765	1.277357
O	4.712462	-0.986899	1.350924
C	5.410179	-1.588250	-2.234672
O	6.669372	-1.939960	-2.471644
C	6.946448	-2.742103	-3.665255
C	7.225670	-1.846277	-4.858328
O	4.453046	-1.875768	-2.936206
C	5.190361	1.524170	-2.115120
C	4.892377	1.536409	-3.483069
C	5.571277	2.413273	-4.337201
O	5.315536	2.471602	-5.675379
C	6.553261	3.274801	-3.829964
C	6.844036	3.256364	-2.467867
C	6.166751	2.389115	-1.605343
H	1.435312	2.836825	-0.664210
H	3.143716	2.614041	-0.593637
H	2.969703	-0.601207	-2.093460
H	4.466933	1.020168	-0.164762
H	6.308428	-0.468132	-0.654501
H	6.100015	-3.407360	-3.841034
H	7.822481	-3.330073	-3.386778
H	6.344631	-1.256232	-5.127215
H	7.497674	-2.469965	-5.716057
H	8.056986	-1.166563	-4.649228
H	4.127319	0.878656	-3.887394
H	4.594849	1.863905	-5.897372
H	7.066508	3.949944	-4.507835
H	7.597468	3.931212	-2.072303
H	6.393708	2.390301	-0.542774

Aryl Substituent: 3-OH Intermediate 6 Conformer 4

S	0.467949	0.518858	-1.718145
C	2.026989	1.055091	-1.251628
N	2.201730	2.210673	-0.590620
N	3.119007	0.303546	-1.522091
C	4.499395	0.659750	-1.198414
C	5.358850	-0.644271	-1.132802
C	4.862878	-1.496864	0.047165
C	4.423597	-2.967586	-0.177730
F	3.244961	-2.990776	-0.829328

F	5.332497	-3.629999	-0.922064
F	4.281418	-3.603961	0.985505
O	4.794453	-1.039172	1.162475
C	5.420673	-1.356217	-2.481866
O	6.674279	-1.607158	-2.843202
C	6.890618	-2.296335	-4.117953
C	7.006074	-1.298294	-5.256798
O	4.429164	-1.641168	-3.136293
C	5.120577	1.702877	-2.126004
C	4.730282	1.804209	-3.466129
C	5.338106	2.749642	-4.301027
O	4.992083	2.891207	-5.612513
C	6.338351	3.593811	-3.800780
C	6.718934	3.487905	-2.464452
C	6.115128	2.550394	-1.621704
H	1.392156	2.779185	-0.393286
H	3.109498	2.623587	-0.427849
H	2.976981	-0.496822	-2.128583
H	4.520016	1.048543	-0.174991
H	6.373884	-0.345440	-0.855404
H	6.071673	-3.001064	-4.271552
H	7.820991	-2.844326	-3.962345
H	6.069275	-0.753130	-5.401332
H	7.236048	-1.837049	-6.181753
H	7.809337	-0.580463	-5.066932
H	3.949797	1.161239	-3.863810
H	4.278175	2.273797	-5.830334
H	6.795640	4.323123	-4.462264
H	7.487643	4.148446	-2.073620
H	6.413993	2.480711	-0.579047

Aryl Substituent: 3-OH Intermediate 6 Conformer 5

S	0.594173	-0.109185	-1.576059
C	2.108817	0.532066	-1.096402
N	2.196185	1.511124	-0.178761
N	3.254471	0.039335	-1.616414
C	4.603585	0.486822	-1.285319
C	5.646480	-0.668886	-1.460257
C	5.733794	-1.594071	-0.243294
C	4.486989	-2.436750	0.138456
F	3.849247	-2.894873	-0.959192
F	4.829194	-3.479999	0.897164
F	3.624860	-1.667315	0.834226
O	6.712736	-1.670596	0.460597
C	5.511637	-1.491398	-2.748797

O	6.418091	-2.467268	-2.752045
C	6.449941	-3.369857	-3.907127
C	7.322876	-2.802391	-5.012190
O	4.703376	-1.283096	-3.635214
C	5.040501	1.754911	-2.023991
C	4.705358	1.968947	-3.365908
C	5.128700	3.135307	-4.012691
O	4.826669	3.394251	-5.317498
C	5.890050	4.090481	-3.324392
C	6.218906	3.869970	-1.989172
C	5.796514	2.709684	-1.332325
H	1.337866	1.895892	0.186206
H	3.036162	2.055992	-0.040216
H	3.154005	-0.588780	-2.403480
H	4.615847	0.721487	-0.215720
H	6.635918	-0.203705	-1.502096
H	5.423763	-3.537601	-4.238856
H	6.858737	-4.295115	-3.499111
H	6.900932	-1.876483	-5.412665
H	7.386045	-3.531719	-5.826350
H	8.335010	-2.604866	-4.647206
H	4.120167	1.234374	-3.910613
H	4.294022	2.669684	-5.677758
H	6.205863	4.990344	-3.843186
H	6.801600	4.612521	-1.451480
H	6.052513	2.550859	-0.287966

Aryl Substituent: 3-OH Intermediate 6 Conformer 6

S	0.371021	1.387636	-1.492670
C	2.035667	1.657598	-1.192389
N	2.460354	2.701712	-0.460456
N	2.967476	0.807003	-1.678676
C	4.392854	0.840346	-1.346266
C	5.035337	-0.544538	-1.649059
C	4.553338	-1.602978	-0.660072
C	5.098511	-3.051076	-0.823940
F	4.084538	-3.902938	-1.048750
F	5.971590	-3.154022	-1.847124
F	5.724376	-3.425615	0.301425
O	3.819548	-1.388349	0.275123
C	4.834249	-0.992778	-3.101087
O	3.661082	-1.645257	-3.213149
C	3.310490	-2.211662	-4.520679
C	3.913867	-3.596566	-4.675130
O	5.600438	-0.766171	-4.009045

C	5.153422	1.977768	-2.028089
C	4.784862	2.438683	-3.296571
C	5.517157	3.462739	-3.907444
O	5.199513	3.949305	-5.141214
C	6.619144	4.030389	-3.252982
C	6.976485	3.569591	-1.987795
C	6.248846	2.549174	-1.367798
H	1.776099	3.375374	-0.150361
H	3.431599	2.982604	-0.429363
H	2.600822	0.008620	-2.180766
H	4.486734	0.967381	-0.260978
H	6.117302	-0.443192	-1.508294
H	3.651666	-1.521926	-5.294437
H	2.220456	-2.239340	-4.508337
H	5.005913	-3.550704	-4.704508
H	3.561854	-4.037941	-5.612918
H	3.608749	-4.245243	-3.849292
H	3.925036	2.013850	-3.807813
H	4.426938	3.479170	-5.488328
H	7.172497	4.826558	-3.741360
H	7.824796	4.014964	-1.476011
H	6.531272	2.200632	-0.377796

Aryl Substituent: 3-OH Intermediate 7 Conformer 0

O	3.787179	-2.500060	2.485560
C	3.053665	-1.704741	1.932315
O	2.211685	-0.873125	2.555396
C	2.198985	-0.900889	4.015668
C	1.232246	-1.954422	4.530115
C	2.951858	-1.537239	0.415870
H	2.037990	-0.985933	0.187047
C	4.137006	-0.738029	-0.178001
O	5.332309	-1.356256	0.224912
C	4.143799	0.731893	0.313282
F	2.968189	1.346039	0.097785
F	4.423915	0.804257	1.624385
F	5.101228	1.422466	-0.350035
N	3.977019	-0.687985	-1.617112
C	3.485232	-1.710893	-2.368706
N	2.944151	-2.747308	-1.712420
C	2.902281	-2.928557	-0.256108
H	3.790025	-3.483382	0.066053
C	1.656572	-3.709955	0.135846
C	1.777273	-4.796019	1.009516
C	0.638606	-5.509814	1.403130

O	0.703654	-6.574682	2.253447
C	-0.622989	-5.144119	0.916867
C	-0.734210	-4.063785	0.042673
C	0.395270	-3.341537	-0.350954
S	3.535989	-1.641232	-4.072098
H	1.886537	0.106489	4.295817
H	3.216943	-1.077399	4.368638
H	0.222860	-1.778302	4.146637
H	1.198611	-1.909750	5.623616
H	1.553118	-2.957642	4.236465
H	6.071042	-0.959919	-0.264303
H	4.450998	0.047976	-2.125940
H	2.600285	-3.506438	-2.285942
H	2.753958	-5.085856	1.389018
H	1.623481	-6.723499	2.518264
H	-1.495400	-5.710840	1.227600
H	-1.712791	-3.782414	-0.335982
H	0.295432	-2.505100	-1.036320

Aryl Substituent: 3-OH Intermediate 7 Conformer 1

O	4.055120	-2.926930	2.295121
C	3.211341	-2.127838	1.938790
O	2.385088	-1.477134	2.761641
C	2.564159	-1.714272	4.190119
C	1.541837	-0.867821	4.920804
C	2.936292	-1.749742	0.483293
H	1.959318	-1.266206	0.427627
C	3.978910	-0.752923	-0.078806
O	5.258653	-1.301183	0.109810
C	3.911663	0.619073	0.639745
F	2.670666	1.134176	0.638177
F	4.327528	0.521412	1.912665
F	4.726585	1.497122	0.008233
N	3.664317	-0.502644	-1.470272
C	3.204317	-1.452432	-2.330561
N	2.828058	-2.625637	-1.802013
C	2.938626	-3.023089	-0.392934
H	3.895389	-3.531843	-0.234357
C	1.804431	-3.970825	-0.029536
C	2.096604	-5.156861	0.652310
C	1.064115	-6.030286	1.015930
O	1.298562	-7.198014	1.681097
C	-0.263396	-5.722056	0.692845
C	-0.546122	-4.539710	0.009852
C	0.476758	-3.659836	-0.353073

S	3.088056	-1.130024	-4.001170
H	3.590215	-1.443530	4.454827
H	2.429399	-2.782781	4.379643
H	1.689549	0.195193	4.707968
H	1.650334	-1.022109	5.998775
H	0.523675	-1.146637	4.633221
H	5.904435	-0.765698	-0.378410
H	4.020101	0.344455	-1.895602
H	2.508985	-3.326243	-2.458369
H	3.125019	-5.402601	0.905156
H	2.248264	-7.291528	1.848009
H	-1.052300	-6.411474	0.977657
H	-1.575751	-4.302524	-0.242595
H	0.243600	-2.745301	-0.890217

Aryl Substituent: 3-OH Intermediate 7 Conformer 2

O	3.996518	-2.502630	2.413175
C	3.238059	-1.689097	1.923560
O	2.455666	-0.853504	2.614966
C	2.545999	-0.899763	4.072202
C	1.601005	-1.944883	4.641199
C	3.031613	-1.499533	0.420401
H	2.111361	-0.933757	0.264122
C	4.182297	-0.707229	-0.246010
O	5.394184	-1.348927	0.059459
C	4.247868	0.754211	0.265214
F	3.070357	1.389521	0.142069
F	4.621259	0.801820	1.553920
F	5.167745	1.439686	-0.454166
N	3.918450	-0.632279	-1.668571
C	3.370105	-1.643252	-2.397340
N	2.863944	-2.682255	-1.717601
C	2.916474	-2.881028	-0.263809
H	3.815560	-3.451853	-0.008351
C	1.688701	-3.650403	0.201881
C	1.852092	-4.743382	1.059831
C	0.732470	-5.445747	1.522631
O	0.839332	-6.515526	2.362565
C	-0.553163	-5.062688	1.120155
C	-0.707265	-3.975707	0.260769
C	0.403147	-3.264054	-0.200490
S	3.309901	-1.555465	-4.099394
H	2.269800	0.108400	4.385583
H	3.583524	-1.095790	4.349802
H	0.570845	-1.750639	4.328116

H	1.641338	-1.910570	5.734857
H	1.886252	-2.949697	4.317806
H	6.101480	-0.954449	-0.475630
H	4.367384	0.103763	-2.199777
H	2.480429	-3.433893	-2.275728
H	2.847429	-5.047288	1.374326
H	1.772554	-6.676255	2.566659
H	-1.410470	-5.621359	1.483131
H	-1.704696	-3.680629	-0.052653
H	0.269752	-2.421874	-0.872975

Aryl Substituent: 3-OH Intermediate 7 Conformer 3

O	4.023327	-2.698604	2.368347
C	3.238306	-1.874294	1.943169
O	2.445463	-1.106100	2.698096
C	2.550371	-1.253505	4.147451
C	1.630414	-2.354858	4.647401
C	3.004197	-1.592935	0.458463
H	2.075193	-1.029511	0.354009
C	4.133116	-0.746018	-0.176546
O	5.358925	-1.384888	0.073287
C	4.183397	0.683745	0.419809
F	2.994126	1.305654	0.350685
F	4.573595	0.660133	1.704259
F	5.081467	1.426899	-0.269132
N	3.847711	-0.590986	-1.588488
C	3.292897	-1.559135	-2.368413
N	2.804005	-2.641171	-1.745290
C	2.891798	-2.930642	-0.308600
H	3.803577	-3.503561	-0.108989
C	1.689416	-3.749734	0.136026
C	0.391556	-3.343437	-0.203230
C	-0.709774	-4.090246	0.227281
O	-1.993308	-3.741290	-0.076108
C	-0.519495	-5.244935	0.999883
C	0.773185	-5.641663	1.332692
C	1.881147	-4.903345	0.903505
S	3.203008	-1.367659	-4.060623
H	2.257758	-0.275867	4.534144
H	3.593856	-1.448314	4.402565
H	0.593614	-2.157286	4.359333
H	1.682344	-2.399840	5.740195
H	1.929282	-3.327928	4.248023
H	6.050745	-0.951988	-0.452016
H	4.282166	0.179453	-2.081295

H	2.420016	-3.362346	-2.341986
H	0.232092	-2.453630	-0.808400
H	-1.992605	-2.934752	-0.612607
H	-1.385971	-5.813552	1.323515
H	0.920819	-6.539037	1.926839
H	2.886065	-5.219330	1.165145

Aryl Substituent: 3-OH Intermediate 7 Conformer 4

O	3.998238	-2.478634	2.436173
C	3.244274	-1.663759	1.942407
O	2.468620	-0.817802	2.629340
C	2.556758	-0.858464	4.086632
C	1.593963	-1.885916	4.658105
C	3.035251	-1.483635	0.438525
H	2.122976	-0.905407	0.280649
C	4.193972	-0.714152	-0.239812
O	5.397778	-1.371942	0.063066
C	4.285565	0.749643	0.260633
F	3.118741	1.404303	0.137674
F	4.664435	0.799722	1.547815
F	5.214177	1.414327	-0.467051
N	3.922673	-0.645045	-1.661455
C	3.355380	-1.653194	-2.379509
N	2.834505	-2.678052	-1.688850
C	2.897129	-2.868627	-0.234123
H	3.791017	-3.449153	0.017404
C	1.668720	-3.624622	0.249787
C	0.385712	-3.211255	-0.134371
C	-0.739203	-3.899396	0.331543
O	-2.009806	-3.541577	-0.013316
C	-0.587480	-5.001988	1.184558
C	0.690732	-5.406082	1.561665
C	1.822005	-4.726640	1.097611
S	3.289449	-1.578976	-4.081837
H	2.296533	0.155412	4.395411
H	3.590484	-1.070143	4.366787
H	0.567008	-1.673610	4.346196
H	1.636470	-1.851479	5.751680
H	1.860470	-2.895912	4.334690
H	6.107184	-0.994201	-0.481132
H	4.380463	0.079389	-2.201040
H	2.444929	-3.431898	-2.239773
H	0.256286	-2.361502	-0.801050
H	-1.982751	-2.771140	-0.599895
H	-1.471811	-5.525578	1.534821

H	0.808452	-6.263200	2.218614
H	2.815402	-5.048014	1.394328

Aryl Substituent: 3-OH Intermediate 7 Conformer 5

O	3.911731	-2.598716	2.455933
C	3.130245	-1.800090	1.978119
O	2.288958	-1.033308	2.680084
C	2.346236	-1.130356	4.136218
C	3.405999	-0.201875	4.705986
C	2.947302	-1.565439	0.478073
H	2.012760	-1.023721	0.321660
C	4.083791	-0.717196	-0.141666
O	5.310231	-1.329999	0.163909
C	4.094300	0.728733	0.415902
F	2.898708	1.329427	0.295157
F	4.447371	0.746708	1.711564
F	5.001684	1.466331	-0.266605
N	3.840667	-0.604229	-1.565368
C	3.333425	-1.605371	-2.335903
N	2.853243	-2.683333	-1.699306
C	2.887830	-2.927353	-0.252044
H	3.800374	-3.477644	0.000836
C	1.677753	-3.749980	0.166926
C	1.861614	-4.864365	0.992457
C	0.757804	-5.618142	1.410239
O	0.884806	-6.711359	2.216238
C	-0.532465	-5.263419	0.996382
C	-0.706874	-4.154222	0.169694
C	0.387653	-3.392418	-0.247459
S	3.291718	-1.460320	-4.034754
H	2.530801	-2.171960	4.406601
H	1.343666	-0.843775	4.457930
H	4.407724	-0.506541	4.391290
H	3.362661	-0.237167	5.799435
H	3.233297	0.829627	4.385876
H	6.012653	-0.898759	-0.348659
H	4.269117	0.165611	-2.064319
H	2.497414	-3.425286	-2.287831
H	2.860587	-5.145634	1.316261
H	1.818649	-6.846733	2.435397
H	-1.377453	-5.860840	1.324785
H	-1.707653	-3.881156	-0.152763
H	0.238476	-2.533730	-0.895275

Aryl Substituent: 3-OH Intermediate 7 Conformer 6

O	4.086467	-2.583464	2.377159

C	3.322044	-1.751471	1.929349
O	2.556167	-0.936802	2.662723
C	2.671168	-1.032450	4.115666
C	3.804783	-0.166768	4.639856
C	3.073218	-1.523948	0.437897
H	2.158496	-0.939774	0.322841
C	4.214503	-0.737627	-0.250177
O	5.425115	-1.403505	0.003115
C	4.319434	0.712417	0.286875
F	3.148145	1.367217	0.218624
F	4.738158	0.727650	1.563197
F	5.223964	1.399751	-0.449569
N	3.906092	-0.630101	-1.661695
C	3.310292	-1.613023	-2.391410
N	2.807127	-2.656485	-1.716343
C	2.910946	-2.888743	-0.270605
H	3.810029	-3.478276	-0.061970
C	1.689851	-3.650090	0.224840
C	1.869267	-4.768489	1.045997
C	0.757688	-5.465677	1.535155
O	0.880396	-6.559902	2.340454
C	-0.536543	-5.050480	1.197227
C	-0.706984	-3.937767	0.374440
C	0.395716	-3.231949	-0.113525
S	3.190335	-1.485884	-4.087821
H	2.806357	-2.082486	4.382833
H	1.701195	-0.687509	4.477648
H	4.774235	-0.527766	4.286291
H	3.798427	-0.205870	5.733111
H	3.678476	0.874030	4.328446
H	6.119321	-1.014525	-0.552462
H	4.348969	0.109396	-2.193079
H	2.387845	-3.387565	-2.276000
H	2.871188	-5.096727	1.311292
H	1.818249	-6.740577	2.502347
H	-1.387870	-5.604750	1.580399
H	-1.711183	-3.617720	0.111256
H	0.249419	-2.369392	-0.756882

Aryl Substituent: 3-OH Intermediate 8 Conformer 0

O	5.147886	0.501958	1.314851
C	4.371766	-0.425271	1.288103
C	2.879208	-0.296092	1.094420
C	2.144357	-0.736708	2.350751
O	0.834686	-0.855346	2.130191

C	-0.007704	-1.223628	3.267202
C	-0.425606	0.006553	4.054700
O	2.702937	-0.934030	3.415659
C	4.961129	-1.856269	1.427553
F	4.007167	-2.807008	1.422048
F	5.794067	-2.096973	0.397222
F	5.663496	-1.965928	2.566167
H	2.548366	-0.897588	0.240627
H	2.644217	0.752108	0.888172
H	0.539797	-1.936836	3.886289
H	-0.863824	-1.721609	2.809099
H	0.440841	0.494068	4.510311
H	-1.111450	-0.292840	4.854053
H	-0.940335	0.725939	3.410782

Aryl Substituent: 3-OH Intermediate 8 Conformer 1

O	5.175917	-1.105317	0.688327
C	4.359736	-0.391784	1.224593
C	2.870164	-0.448958	0.980174
C	2.144919	-0.898287	2.238873
O	0.825869	-0.742477	2.119632
C	-0.006506	-1.184026	3.237429
C	-0.338948	-2.661674	3.117608
O	2.717874	-1.353656	3.213068
C	4.889208	0.686097	2.210758
F	3.895382	1.408919	2.761969
F	5.595840	0.113239	3.197902
F	5.702489	1.532475	1.550899
H	2.676342	-1.165361	0.176686
H	2.494939	0.531093	0.665331
H	-0.899235	-0.561036	3.161961
H	0.516827	-0.958670	4.168586
H	-0.830513	-2.875032	2.163701
H	-1.020086	-2.944277	3.927037
H	0.562885	-3.275068	3.197666

Aryl Substituent: 3-OH Intermediate 8 Conformer 2

O	5.153258	-1.112774	0.679519
C	4.332887	-0.408695	1.221399
C	2.850238	-0.427190	0.931899
C	2.082509	-0.907520	2.153496
O	0.778377	-0.654799	2.039215
C	-0.089090	-1.095714	3.130426
C	-0.162711	-0.043497	4.223950
O	2.613423	-1.472429	3.093621
C	4.849824	0.620038	2.265382

F	3.846413	1.289237	2.865479
F	5.576552	0.006095	3.211615
F	5.638755	1.520264	1.648284
H	2.668266	-1.113651	0.099871
H	2.501822	0.569208	0.639470
H	0.285644	-2.049121	3.507813
H	-1.057076	-1.249155	2.650587
H	0.812287	0.098808	4.698323
H	-0.873653	-0.369488	4.990285
H	-0.505527	0.914282	3.821165

Aryl Substituent: 3-OH Intermediate 8 Conformer 3

O	5.096423	0.536317	1.305607
C	4.332192	-0.400755	1.289217
C	2.850664	-0.303129	1.010749
C	2.055036	-0.693387	2.246375
O	0.763797	-0.861352	1.959041
C	-0.130506	-1.207732	3.062782
C	-0.174587	-2.711812	3.272861
O	2.553860	-0.807152	3.352286
C	4.928673	-1.814688	1.535339
F	3.983638	-2.774416	1.553144
F	5.799049	-2.106959	0.550176
F	5.591428	-1.849339	2.701793
H	2.573715	-0.950281	0.171398
H	2.614589	0.730171	0.740427
H	-1.099029	-0.814088	2.750028
H	0.204564	-0.681434	3.958541
H	-0.475044	-3.225263	2.354608
H	-0.906435	-2.945037	4.053255
H	0.799873	-3.092865	3.590971

Aryl Substituent: 3-OH Intermediate 8 Conformer 4

O	5.153842	-0.339177	1.003675
C	4.132034	0.227073	1.316850
C	2.741001	-0.246663	0.966605
C	1.979332	-0.628805	2.225961
O	0.686041	-0.827372	1.968599
C	-0.163050	-1.251632	3.080367
C	-0.136874	-2.762302	3.239837
O	2.509428	-0.747634	3.316686
C	4.265046	1.558801	2.106893
F	3.068152	2.092272	2.417996
F	4.951325	1.363396	3.244027
F	4.938622	2.454352	1.360697
H	2.824790	-1.121720	0.315750

H	2.194879	0.533904	0.425351
H	-1.155508	-0.890913	2.805147
H	0.171451	-0.740922	3.985361
H	-0.439138	-3.257763	2.312328
H	-0.837098	-3.053201	4.029811
H	0.861125	-3.110892	3.519959

Aryl Substituent: 3-OH Intermediate 8 Conformer 5

O	5.149681	-0.208307	0.940882
C	4.191842	-0.945575	0.899574
C	2.751421	-0.490872	0.901739
C	2.054294	-0.951132	2.172392
O	0.736296	-0.763433	2.097637
C	-0.062081	-1.134227	3.265464
C	-0.446547	-2.603256	3.216302
O	2.649978	-1.407644	3.132446
C	4.467213	-2.471620	0.798954
F	3.333558	-3.198788	0.794958
F	5.130738	-2.729324	-0.344117
F	5.230041	-2.880328	1.825099
H	2.223725	-0.886029	0.026580
H	2.729868	0.601543	0.852522
H	-0.936615	-0.484686	3.202122
H	0.507131	-0.892589	4.165096
H	-0.984726	-2.835511	2.292465
H	-1.101453	-2.832150	4.063483
H	0.437836	-3.242972	3.283502

Aryl Substituent: 3-OH Intermediate 8 Conformer 6

O	5.161041	-0.297889	0.825343
C	4.162023	0.241062	1.242914
C	2.751345	-0.224608	0.969946
C	2.075357	-0.656112	2.261503
O	0.763180	-0.822259	2.090612
C	-0.012484	-1.271957	3.245335
C	-0.450873	-0.092887	4.097406
O	2.681656	-0.843292	3.301755
C	4.346476	1.529157	2.093004
F	3.171569	2.048778	2.498236
F	5.093226	1.271103	3.178611
F	4.982802	2.461267	1.359586
H	2.792418	-1.073775	0.281700
H	2.170830	0.574801	0.496331
H	0.591428	-1.982043	3.813602
H	-0.864718	-1.791036	2.803652
H	0.411405	0.416109	4.537085

H	-1.088566	-0.453864	4.911045
H	-1.023615	0.625942	3.503832

Aryl Substituent: 3-OH Intermediate 9 Conformer 0

N	0.905888	0.337267	-0.030989
C	2.229903	0.506266	-0.165506
N	2.744671	0.596496	-1.424512
C	2.056621	0.276550	-2.682659
C	1.775908	-1.239504	-2.743911
C	1.158204	-1.862512	-3.987124
O	0.760803	-0.978566	-4.903752
C	0.167062	-1.500031	-6.130480
C	-1.327079	-1.719416	-5.961233
O	1.051654	-3.071053	-4.097488
O	0.830227	0.951159	-2.770804
C	3.021325	0.735833	-3.848402
F	3.949816	1.618950	-3.415261
F	3.688917	-0.307998	-4.377312
F	2.337105	1.337540	-4.828356
S	3.273204	0.611818	1.184373
H	0.286496	0.493382	-0.818293
H	0.519415	0.338219	0.901229
H	3.746594	0.726009	-1.457030
H	1.110060	-1.483415	-1.910730
H	2.709840	-1.774171	-2.558015
H	0.684190	-2.422233	-6.402444
H	0.380513	-0.729400	-6.873343
H	-1.526706	-2.494969	-5.216658
H	-1.756197	-2.040091	-6.916295
H	-1.824738	-0.794865	-5.653439
H	0.985305	1.909733	-2.747565

Aryl Substituent: 3-OH Intermediate 9 Conformer 1

N	1.217711	-0.559768	-0.056195
C	2.326687	0.177437	-0.197304
N	2.621466	0.665330	-1.440120
C	2.041712	0.215442	-2.710318
C	2.522928	-1.215937	-3.048770
C	1.957889	-1.846777	-4.313456
O	0.714257	-2.305816	-4.123459
C	0.061714	-2.953243	-5.255684
C	0.421123	-4.428690	-5.324992
O	2.580676	-1.949393	-5.354103
O	0.640016	0.228619	-2.677482
C	2.515217	1.244351	-3.769590
F	2.019207	2.464847	-3.483017

F	3.860736	1.337526	-3.793738
F	2.091454	0.907409	-4.995860
S	3.360842	0.529309	1.115293
H	0.536913	-0.627391	-0.804200
H	0.966880	-0.877141	0.868260
H	3.522024	1.125179	-1.495155
H	2.260985	-1.848521	-2.197266
H	3.609521	-1.191869	-3.143392
H	0.344079	-2.425671	-6.169069
H	-1.003508	-2.807672	-5.066695
H	1.488489	-4.562705	-5.520865
H	-0.140380	-4.900148	-6.138400
H	0.164956	-4.936925	-4.390396
H	0.342407	1.134519	-2.485126

Aryl Substituent: 3-OH Intermediate 9 Conformer 2

N	0.941985	0.772966	0.029719
C	2.255023	0.882499	-0.206077
N	2.700558	0.760334	-1.494798
C	1.963889	0.155104	-2.607738
C	1.766868	-1.366816	-2.477806
C	3.034653	-2.182746	-2.285403
O	2.844078	-3.432960	-2.725876
C	3.932819	-4.386404	-2.534044
C	3.854671	-5.033807	-1.161296
O	4.063478	-1.779298	-1.774529
O	0.668932	0.691668	-2.727822
C	2.741961	0.530467	-3.900410
F	4.055334	0.246319	-3.809578
F	2.245673	-0.125516	-4.961574
F	2.623181	1.853440	-4.137797
S	3.376649	1.190197	1.045300
H	0.276596	0.716780	-0.732612
H	0.608050	0.930907	0.968713
H	3.705898	0.649533	-1.553026
H	1.212785	-1.735415	-3.342146
H	1.137351	-1.539339	-1.597431
H	4.880745	-3.866184	-2.684216
H	3.788440	-5.115817	-3.332972
H	4.002935	-4.294193	-0.369462
H	4.639493	-5.792419	-1.074302
H	2.886372	-5.521738	-1.014527
H	0.741282	1.644339	-2.909391

Aryl Substituent: 3-OH Intermediate 9 Conformer 3

N	1.675242	-0.177525	0.396932

C	2.817919	0.216754	-0.179276
N	2.778686	0.605495	-1.489657
C	1.693401	0.345486	-2.442255
C	1.628354	-1.157837	-2.796110
C	0.496104	-1.565815	-3.727278
O	0.974838	-1.967648	-4.914700
C	0.005021	-2.388822	-5.918408
C	-0.514820	-1.198242	-6.708063
O	-0.680958	-1.561706	-3.422526
O	0.449363	0.746999	-1.937627
C	2.019289	1.208789	-3.689076
F	1.959410	2.517937	-3.377471
F	3.257316	0.946622	-4.155421
F	1.139970	0.981353	-4.678401
S	4.299729	0.257375	0.668666
H	0.784465	-0.047630	-0.069023
H	1.688224	-0.408093	1.379272
H	3.691496	0.786758	-1.889240
H	1.486742	-1.699098	-1.857006
H	2.589762	-1.449544	-3.220993
H	-0.802988	-2.926953	-5.418399
H	0.562785	-3.079957	-6.553016
H	-1.080703	-0.518428	-6.065375
H	-1.178393	-1.553231	-7.503418
H	0.310237	-0.645698	-7.167267
H	0.485511	1.699060	-1.742163

Aryl Substituent: 3-OH Intermediate 9 Conformer 4

N	1.370146	-0.779972	-0.118956
C	2.392254	0.079981	-0.216512
N	2.599360	0.701061	-1.416710
C	2.027530	0.308575	-2.709767
C	2.643056	-1.027450	-3.189560
C	2.123175	-1.591540	-4.504162
O	0.925442	-2.171746	-4.355809
C	0.325817	-2.775952	-5.540013
C	0.809128	-4.204868	-5.728690
O	2.745273	-1.548793	-5.549501
O	0.634020	0.171205	-2.643323
C	2.357828	1.472229	-3.680164
F	1.747465	2.602891	-3.272400
F	3.684900	1.709133	-3.726424
F	1.932300	1.201891	-4.922588
S	3.422432	0.424418	1.101205
H	0.676533	-0.851018	-0.854820

H	1.177652	-1.195615	0.780174
H	3.446960	1.254187	-1.449605
H	2.461736	-1.761188	-2.400398
H	3.719885	-0.884308	-3.290665
H	0.559484	-2.153216	-6.405951
H	-0.747285	-2.736972	-5.343774
H	1.884140	-4.230989	-5.927092
H	0.290338	-4.653432	-6.582383
H	0.597209	-4.808645	-4.841026
H	0.251123	1.019631	-2.360987

Aryl Substituent: 3-OH Intermediate 9 Conformer 5

N	1.384449	-0.287441	0.201240
C	2.455087	0.401648	-0.214579
N	2.586354	0.641040	-1.554232
C	1.857460	-0.034010	-2.634336
C	2.315931	-1.506840	-2.757361
C	1.641954	-2.344820	-3.833352
O	0.398310	-2.696396	-3.485359
C	-0.373198	-3.464123	-4.455369
C	-1.068867	-2.541026	-5.442854
O	2.193242	-2.681684	-4.865197
O	0.471954	-0.002956	-2.426428
C	2.176724	0.774079	-3.918902
F	1.697059	2.029136	-3.808516
F	3.506470	0.853393	-4.129329
F	1.610353	0.212438	-4.997713
S	3.637814	0.988836	0.868251
H	0.615331	-0.485373	-0.428678
H	1.252606	-0.414682	1.193569
H	3.465925	1.074301	-1.807709
H	2.138140	-1.975497	-1.786110
H	3.388704	-1.511188	-2.955247
H	-1.090847	-4.018069	-3.847485
H	0.295397	-4.166342	-4.957342
H	-1.714121	-1.828700	-4.920091
H	-1.688401	-3.135103	-6.122667
H	-0.340207	-1.984497	-6.038968
H	0.186677	0.925177	-2.372134

Aryl Substituent: 3-OH Intermediate 9 Conformer 6

N	1.118844	-0.402996	-0.036651
C	2.262640	0.273783	-0.200949
N	2.625262	0.629620	-1.469385
C	2.064666	0.097571	-2.717000
C	2.492778	-1.375920	-2.916608

C	1.958621	-2.085414	-4.152769
O	0.676780	-2.442058	-4.004437
C	0.046399	-3.149195	-5.113949
C	0.292208	-4.646104	-5.017732
O	2.633779	-2.327307	-5.136485
O	0.665167	0.171889	-2.733270
C	2.616070	1.011175	-3.842347
F	2.162241	2.270222	-3.681875
F	3.964432	1.049205	-3.825539
F	2.219966	0.582960	-5.049911
S	3.262365	0.705994	1.115980
H	0.463013	-0.510741	-0.802247
H	0.817358	-0.614502	0.902986
H	3.547805	1.042655	-1.531748
H	2.162173	-1.924378	-2.031132
H	3.582452	-1.406831	-2.958303
H	0.425640	-2.736461	-6.050927
H	-1.013579	-2.908638	-5.013707
H	1.355746	-4.876054	-5.126090
H	-0.253850	-5.154397	-5.819413
H	-0.060159	-5.038401	-4.058956
H	0.401084	1.102123	-2.628735

Aryl Substituent: 3-OMe Intermediate 12 Conformer 0

H	1.371023	0.233224	0.651401
C	2.463153	0.182371	0.548471
C	2.717433	-1.212354	-0.113216
O	1.590091	-1.538197	-0.917943
C	4.031609	-1.369858	-0.885814
C	4.043238	-2.250263	-1.980656
C	5.223879	-2.473324	-2.697323
O	5.321962	-3.309981	-3.772538
C	4.149247	-4.004640	-4.200318
C	6.407379	-1.816518	-2.324405
C	6.391853	-0.948173	-1.239275
C	5.214606	-0.720615	-0.515808
C	2.857031	1.350340	-0.354658
O	3.610223	2.250065	-0.035547
O	2.257474	1.235189	-1.546708
C	2.553911	2.251725	-2.558095
C	1.631938	3.449188	-2.408729
C	3.021457	0.356817	1.944240
N	3.822510	-0.491919	2.443392
C	4.350770	-0.542912	3.729192
N	5.407798	0.236333	3.951082

S	3.736021	-1.662014	4.832345
C	2.515901	1.568661	2.752028
F	1.524791	2.216335	2.115237
F	2.049460	1.181058	3.953222
F	3.518625	2.443467	2.966139
H	2.704739	-1.944123	0.701528
H	1.562567	-0.893607	-1.643564
H	3.122746	-2.746875	-2.257036
H	4.453396	-4.602721	-5.060444
H	3.359334	-3.306999	-4.502104
H	3.766975	-4.665441	-3.413521
H	7.314475	-1.999045	-2.892571
H	7.305873	-0.437109	-0.949431
H	5.239111	-0.041038	0.326550
H	2.396375	1.732302	-3.504260
H	3.605073	2.530819	-2.465453
H	0.583013	3.143319	-2.464121
H	1.828348	4.156121	-3.221486
H	1.803823	3.962020	-1.458424
H	5.741958	0.896567	3.260793
H	5.919800	0.148808	4.818806

Aryl Substituent: 3-OMe Intermediate 12 Conformer 1

H	1.335547	0.710333	-0.227932
C	2.427688	0.629176	-0.231248
C	2.839312	-0.569179	-1.151637
O	2.887058	-1.690005	-0.263448
C	1.874674	-0.808356	-2.300990
C	0.596342	-1.323145	-2.027198
C	-0.296856	-1.565690	-3.075451
O	-1.559102	-2.061360	-2.914647
C	-2.012228	-2.340855	-1.589049
C	0.089760	-1.301161	-4.401274
C	1.360420	-0.801220	-4.663758
C	2.259207	-0.554177	-3.618500
C	2.964533	1.988815	-0.670200
O	3.358819	2.825138	0.123676
O	2.919708	2.146980	-1.991966
C	3.371329	3.426765	-2.535366
C	2.243199	4.444140	-2.541754
C	2.819494	0.376896	1.213533
N	1.908354	0.230174	2.084623
C	2.032535	-0.030798	3.442848
N	2.054216	-1.323684	3.764296
S	1.973039	1.229697	4.566237

C	4.312457	0.290908	1.572634
F	5.095037	0.368332	0.477804
F	4.667232	1.288244	2.401856
F	4.590524	-0.872316	2.194385
H	3.835761	-0.371567	-1.560978
H	3.212467	-2.451709	-0.765752
H	0.325960	-1.544483	-1.001211
H	-3.033001	-2.710823	-1.693763
H	-2.013386	-1.436863	-0.968843
H	-1.393960	-3.108470	-1.109151
H	-0.616696	-1.499333	-5.201799
H	1.656494	-0.604862	-5.690570
H	3.249842	-0.162430	-3.828138
H	3.699783	3.180159	-3.546257
H	4.225207	3.769174	-1.947737
H	1.382611	4.069563	-3.103960
H	2.592490	5.365315	-3.019692
H	1.925362	4.684606	-1.523506
H	2.087623	-2.043165	3.053286
H	2.020644	-1.601667	4.735497

Aryl Substituent: 3-OMe Intermediate 12 Conformer 2

H	1.482878	0.405025	-0.082719
C	2.576626	0.434952	-0.037370
C	3.144751	-0.776928	-0.848536
O	3.238018	-1.828457	0.117292
C	2.273377	-1.180531	-2.026702
C	2.745070	-1.005574	-3.334598
C	1.955793	-1.402078	-4.422040
O	2.326612	-1.272951	-5.730212
C	3.600744	-0.696369	-6.019880
C	0.695837	-1.979183	-4.198058
C	0.238160	-2.156849	-2.894888
C	1.020438	-1.763134	-1.805193
C	2.999552	1.806250	-0.556843
O	3.293902	2.727357	0.184710
O	2.977280	1.869615	-1.887354
C	3.323235	3.146295	-2.509344
C	2.106230	4.049767	-2.611202
C	2.924566	0.327547	1.436455
N	1.991356	0.166551	2.281304
C	2.075071	0.024616	3.660008
N	2.205108	-1.230570	4.087561
S	1.841303	1.356721	4.672771
C	4.401543	0.402785	1.858571

F	5.223050	0.486606	0.793187
F	4.626998	1.476607	2.635807
F	4.755222	-0.690300	2.563825
H	4.143098	-0.516181	-1.216393
H	3.651778	-2.590301	-0.314824
H	3.719823	-0.557731	-3.488550
H	3.686947	-0.692314	-7.107259
H	4.415811	-1.291433	-5.591703
H	3.668419	0.332231	-5.646089
H	0.098900	-2.282804	-5.052848
H	-0.735360	-2.609573	-2.727785
H	0.666773	-1.920099	-0.790557
H	3.701112	2.863955	-3.493366
H	4.125298	3.607118	-1.929701
H	1.300313	3.558513	-3.164492
H	2.381907	4.965587	-3.144341
H	1.739029	4.326980	-1.619229
H	2.341153	-1.996934	3.440646
H	2.150581	-1.433482	5.076241

Aryl Substituent: 3-OMe Intermediate 12 Conformer 3

H	1.017024	0.754297	-0.710122
C	2.106217	0.750225	-0.599135
C	2.693368	-0.511333	-1.311350
O	2.767143	-1.508390	-0.290847
C	1.843768	-0.961408	-2.490151
C	0.687227	-1.721031	-2.248561
C	-0.108370	-2.139811	-3.319998
O	-1.247820	-2.881660	-3.189964
C	-1.667233	-3.251456	-1.875471
C	0.252579	-1.801771	-4.635771
C	1.401786	-1.052689	-4.865244
C	2.204019	-0.629922	-3.798162
C	2.616955	2.049155	-1.227097
O	3.126212	2.135473	-2.325971
O	2.403239	3.077507	-0.395995
C	2.871608	4.398233	-0.811526
C	4.325506	4.596669	-0.416370
C	2.352191	0.708100	0.897441
N	1.376202	0.516904	1.685820
C	1.383737	0.413066	3.070882
N	1.501411	-0.830277	3.534566
S	1.081647	1.766799	4.035339
C	3.793003	0.885170	1.404004
F	4.670997	0.924861	0.379548

F	3.920983	2.037026	2.090334
F	4.153478	-0.120610	2.220397
H	3.696580	-0.261889	-1.671916
H	3.191451	-2.290450	-0.673666
H	0.438820	-1.986795	-1.228065
H	-2.586092	-3.824921	-2.005033
H	-1.871641	-2.368671	-1.258400
H	-0.915848	-3.875061	-1.377011
H	-0.375776	-2.139223	-5.454632
H	1.679490	-0.798754	-5.884619
H	3.095968	-0.039774	-3.980199
H	2.210324	5.089283	-0.286528
H	2.721067	4.496612	-1.888119
H	4.460124	4.427487	0.655699
H	4.627729	5.623032	-0.648193
H	4.979136	3.913186	-0.965834
H	1.677569	-1.609591	2.913074
H	1.399921	-1.010298	4.523947

Aryl Substituent: 3-OMe Intermediate 12 Conformer 4

H	1.272574	0.752232	-0.394817
C	2.363644	0.659682	-0.416190
C	2.746847	-0.584999	-1.285841
O	2.822403	-1.660829	-0.345708
C	1.745188	-0.877163	-2.391031
C	0.482494	-1.391280	-2.051043
C	-0.447249	-1.678453	-3.055293
O	-1.698011	-2.177613	-2.829026
C	-2.096985	-2.416078	-1.478204
C	-0.113198	-1.458754	-4.403380
C	1.142205	-0.958923	-4.731310
C	2.077625	-0.667590	-3.730469
C	2.901373	1.994959	-0.924917
O	3.223264	2.897144	-0.171504
O	2.937789	2.057207	-2.254810
C	3.410791	3.301143	-2.859959
C	4.925490	3.305429	-2.977483
C	2.781760	0.470261	1.030686
N	1.889465	0.341370	1.923449
C	2.044978	0.127442	3.286858
N	2.075779	-1.154413	3.649209
S	2.010917	1.423984	4.369176
C	4.282981	0.423656	1.361298
F	5.038499	0.454867	0.244310
F	4.640548	1.470548	2.125841

F	4.594457	-0.699362	2.036879
H	3.729917	-0.409231	-1.735164
H	3.129326	-2.447261	-0.820734
H	0.252999	-1.576905	-1.008209
H	-3.117001	-2.798982	-1.530818
H	-2.084157	-1.491625	-0.889064
H	-1.452489	-3.161057	-0.997119
H	-0.847400	-1.690688	-5.169107
H	1.397736	-0.797342	-5.774943
H	3.055528	-0.275185	-3.991474
H	3.046275	4.137286	-2.260370
H	2.928099	3.316234	-3.838364
H	5.395647	3.283703	-1.990426
H	5.244348	4.218759	-3.490348
H	5.275128	2.445597	-3.556769
H	2.085946	-1.895962	2.960443
H	2.064667	-1.401407	4.629244

Aryl Substituent: 3-OMe Intermediate 13 Conformer 0

C	4.489964	-1.174983	3.040587
C	5.252091	-0.579492	1.868732
O	5.362764	-1.536620	0.774241
C	4.372608	-1.568334	-0.136482
O	3.353479	-0.904494	-0.057227
C	4.738307	-2.514160	-1.232093
C	3.890119	-3.288377	-1.970079
C	2.452762	-3.543688	-1.971985
C	1.530629	-3.074583	-1.008688
C	0.182700	-3.420043	-1.108874
O	-0.778341	-3.018753	-0.229529
C	-0.389391	-2.192463	0.870151
C	-0.268071	-4.228767	-2.169097
C	0.635789	-4.693629	-3.117887
C	1.987799	-4.362852	-3.022509
C	6.185970	-2.641795	-1.532712
N	6.684574	-3.781145	-1.825605
C	7.965576	-4.091477	-2.250631
N	8.897864	-4.159034	-1.300389
S	8.228674	-4.503323	-3.870135
C	7.053589	-1.365264	-1.568742
F	6.311737	-0.243324	-1.532372
F	7.916500	-1.331587	-0.532771
F	7.781913	-1.328927	-2.701072
H	4.489155	-0.461088	3.870831
H	4.961759	-2.100927	3.382826

H	3.452460	-1.385174	2.766823
H	6.288732	-0.359423	2.129991
H	4.772267	0.325639	1.490657
H	4.405734	-3.868900	-2.732393
H	1.872929	-2.439155	-0.207877
H	-1.304597	-1.985952	1.426095
H	0.049068	-1.249193	0.524568
H	0.324571	-2.709260	1.522093
H	-1.323625	-4.476755	-2.225079
H	0.286423	-5.320505	-3.932727
H	2.691071	-4.738001	-3.759811
H	8.693137	-3.925196	-0.337263
H	9.817310	-4.514997	-1.524563

Aryl Substituent: 3-OMe Intermediate 13 Conformer 1

C	4.567501	-1.184631	3.009050
C	5.304056	-0.573395	1.828953
O	5.400587	-1.519761	0.723903
C	4.392795	-1.550837	-0.167421
O	3.371909	-0.893149	-0.062463
C	4.742165	-2.487010	-1.276263
C	3.885492	-3.259872	-2.006079
C	2.449310	-3.521363	-1.991436
C	1.532361	-3.042349	-1.027904
C	0.185512	-3.396157	-1.112027
O	-0.770339	-2.987052	-0.230591
C	-0.376516	-2.143506	0.854192
C	-0.269637	-4.223013	-2.156257
C	0.628793	-4.696829	-3.105787
C	1.979648	-4.357253	-3.026771
C	6.185633	-2.608645	-1.600100
N	6.684148	-3.745334	-1.902866
C	7.959632	-4.049270	-2.349151
N	8.907203	-4.115597	-1.414154
S	8.198240	-4.456040	-3.973703
C	7.047051	-1.328327	-1.648256
F	6.301646	-0.209603	-1.592583
F	7.930490	-1.295065	-0.629698
F	7.752324	-1.284690	-2.794892
H	4.577404	-0.478468	3.845818
H	5.051596	-2.110554	3.333680
H	3.526535	-1.399253	2.752392
H	6.343836	-0.348900	2.073389
H	4.811686	0.332074	1.468226
H	4.393062	-3.835723	-2.777252

H	1.878310	-2.392281	-0.240553
H	-1.287953	-1.934699	1.415450
H	0.053282	-1.202551	0.491926
H	0.346299	-2.647116	1.506657
H	-1.324283	-4.477293	-2.199867
H	0.276032	-5.337185	-3.908586
H	2.678580	-4.739241	-3.764671
H	8.717717	-3.882777	-0.447676
H	9.824893	-4.465684	-1.654232

Aryl Substituent: 3-OMe Intermediate 13 Conformer 2

C	4.687341	-0.531100	2.998884
C	5.646618	-0.185631	1.872325
O	5.572360	-1.169019	0.797895
C	4.666135	-0.967012	-0.175661
O	3.850638	-0.063642	-0.177730
C	4.839235	-2.000288	-1.244137
C	3.841216	-2.526445	-2.007275
C	2.387621	-2.373733	-1.981550
C	1.712674	-2.693419	-3.182644
C	0.321139	-2.600134	-3.260349
O	-0.410215	-2.867671	-4.378449
C	0.283417	-3.270625	-5.562082
C	-0.412635	-2.219738	-2.124267
C	0.249468	-1.935235	-0.932181
C	1.640279	-2.002531	-0.849449
C	6.215858	-2.480045	-1.506229
N	6.417658	-3.703196	-1.818101
C	7.592179	-4.321972	-2.211724
N	8.443572	-4.638463	-1.236216
S	7.797713	-4.762323	-3.832055
C	7.379472	-1.466815	-1.471905
F	6.944204	-0.193497	-1.459735
F	8.156749	-1.655386	-0.385970
F	8.161391	-1.613830	-2.558186
H	4.824348	0.180403	3.819866
H	4.880327	-1.538218	3.380480
H	3.648648	-0.471280	2.662410
H	6.687024	-0.232184	2.198930
H	5.440844	0.801455	1.453064
H	4.190714	-3.190806	-2.795604
H	2.295249	-3.003719	-4.042347
H	-0.485544	-3.424921	-6.319862
H	0.832065	-4.206011	-5.402740
H	0.978693	-2.493401	-5.899597

H	-1.494727	-2.164652	-2.194370
H	-0.328171	-1.659383	-0.054852
H	2.130233	-1.784878	0.089705
H	8.272683	-4.373736	-0.274535
H	9.251331	-5.210917	-1.441360

Aryl Substituent: 3-OMe Intermediate 13 Conformer 3

C	4.734326	-0.636409	3.094387
C	5.686835	-0.257590	1.972810
O	5.594463	-1.199656	0.863296
C	4.681906	-0.954030	-0.094110
O	3.874755	-0.044016	-0.056429
C	4.836331	-1.948907	-1.201300
C	3.825928	-2.438414	-1.972249
C	2.374132	-2.273890	-1.923052
C	1.682007	-2.535248	-3.128286
C	0.290598	-2.426178	-3.184590
O	-0.456471	-2.637677	-4.304316
C	0.218639	-3.000332	-5.511507
C	-0.425825	-2.089179	-2.023949
C	0.253200	-1.862703	-0.828950
C	1.644268	-1.945819	-0.766588
C	6.206037	-2.429898	-1.495124
N	6.394036	-3.641687	-1.856099
C	7.559808	-4.254254	-2.284138
N	8.417045	-4.614987	-1.329353
S	7.747801	-4.632582	-3.922105
C	7.379067	-1.428991	-1.431981
F	6.955673	-0.153240	-1.367399
F	8.163237	-1.666126	-0.360651
F	8.150809	-1.541008	-2.529629
H	4.884672	0.043004	3.939844
H	4.921776	-1.658567	3.436742
H	3.693309	-0.555435	2.769704
H	6.729559	-0.325357	2.288114
H	5.486495	0.746204	1.592337
H	4.160523	-3.075962	-2.788678
H	2.251281	-2.812665	-4.007847
H	-0.561009	-3.116597	-6.265142
H	0.759310	-3.946685	-5.396006
H	0.917859	-2.217356	-5.826878
H	-1.508146	-2.021874	-2.078013
H	-0.311292	-1.620690	0.066738
H	2.147760	-1.774323	0.175068
H	8.256797	-4.386453	-0.356617

H	9.218498	-5.185025	-1.563854

Aryl Substituent: 3-OMe Intermediate 13 Conformer 4

C	4.447173	-1.389108	3.014984
C	5.235802	-0.749197	1.884810
O	5.351233	-1.654555	0.747430
C	4.375486	-1.628741	-0.178527
O	3.363935	-0.955453	-0.083543
C	4.744925	-2.526205	-1.313296
C	3.896299	-3.260312	-2.090726
C	2.457490	-3.506242	-2.111248
C	1.533097	-3.075461	-1.132318
C	0.183770	-3.408028	-1.254254
O	-0.779344	-3.040497	-0.362496
C	-0.391135	-2.266431	0.774780
C	-0.266363	-4.165939	-2.351651
C	0.639630	-4.593166	-3.315971
C	1.992997	-4.274889	-3.199503
C	6.193592	-2.650501	-1.608905
N	6.684537	-3.776870	-1.959735
C	7.965934	-4.076393	-2.390888
N	8.889479	-4.205650	-1.438512
S	8.238407	-4.401101	-4.028631
C	7.073341	-1.382515	-1.566519
F	6.341239	-0.256690	-1.481347
F	7.921711	-1.411540	-0.518507
F	7.817821	-1.293858	-2.685262
H	4.443086	-0.715953	3.878571
H	4.900776	-2.337132	3.319236
H	3.411302	-1.570787	2.715783
H	6.270983	-0.556529	2.172164
H	4.774254	0.179587	1.543146
H	4.412712	-3.809013	-2.875743
H	1.874472	-2.478699	-0.301966
H	-1.307571	-2.080665	1.335983
H	0.052880	-1.310565	0.473616
H	0.317961	-2.815198	1.405562
H	-1.323006	-4.405001	-2.423604
H	0.290962	-5.181079	-4.159637
H	2.697679	-4.620880	-3.949607
H	8.678147	-4.025294	-0.465328
H	9.806810	-4.559549	-1.674342

Aryl Substituent: 3-OMe Intermediate 14 Conformer 1

C	8.607048	-0.796321	3.994609
C	8.177387	-0.485145	2.571306

O	6.757516	-0.136666	2.522831
C	6.430286	1.138109	2.735766
O	7.214900	2.048783	2.923451
C	4.908867	1.311874	2.718144
C	4.273881	0.584565	3.945955
O	4.905930	1.002872	5.143071
C	2.750903	0.726864	3.975657
C	2.098023	1.163327	5.136958
C	0.707025	1.267741	5.151059
C	-0.057373	0.940930	4.027903
C	0.595201	0.495196	2.870103
O	-0.037763	0.138337	1.714680
C	-1.463480	0.224595	1.670762
C	1.994161	0.390716	2.850023
C	4.597428	2.798389	2.728321
O	4.456378	3.464476	3.730249
C	4.430144	3.463609	1.341837
F	5.477448	3.162685	0.549286
F	3.313718	2.979716	0.757772
F	4.325852	4.790037	1.436322
H	9.655120	-1.113191	3.993413
H	8.002846	-1.604990	4.416412
H	8.515448	0.085756	4.634557
H	8.255530	-1.359161	1.922829
H	8.749504	0.339453	2.142261
H	4.535088	0.858718	1.795546
H	4.525464	-0.475212	3.835263
H	4.784704	1.964718	5.209401
H	2.675178	1.408390	6.021104
H	0.203083	1.608464	6.051534
H	-1.136469	1.030951	4.064731
H	-1.751751	-0.101782	0.670661
H	-1.805919	1.253467	1.831336
H	-1.926359	-0.432261	2.416385
H	2.460750	0.030891	1.937401

Aryl Substituent: 3-OMe Intermediate 14 Conformer 2

C	3.484913	-2.026579	-0.522443
C	2.861781	-0.643548	-0.441265
O	3.108415	-0.037638	0.867348
C	4.251935	0.622634	1.033894
O	5.109933	0.776180	0.180594
C	4.387409	1.207105	2.433974
C	3.808133	0.323630	3.585584
O	4.605256	0.544586	4.758981

C	2.330248	0.543754	3.848173
C	1.444479	-0.543268	3.832309
C	0.097415	-0.342106	4.129415
C	-0.390108	0.928592	4.450476
C	0.495390	2.015178	4.470485
O	0.139726	3.297965	4.767811
C	-1.228973	3.565083	5.081832
C	1.850867	1.816843	4.166220
C	5.861366	1.512502	2.691137
O	6.292735	2.623714	2.894612
C	6.888105	0.345310	2.686931
F	6.306576	-0.856915	2.487849
F	7.791327	0.536825	1.709918
F	7.545826	0.302987	3.856513
H	3.229150	-2.480535	-1.485431
H	3.105657	-2.673075	0.274544
H	4.574522	-1.971550	-0.447440
H	1.773626	-0.683965	-0.509487
H	3.251159	0.025696	-1.210658
H	3.889383	2.183013	2.423726
H	3.968770	-0.725277	3.332491
H	4.318426	1.370832	5.177001
H	1.809672	-1.535858	3.585956
H	-0.590513	-1.182934	4.114239
H	-1.441339	1.059477	4.678001
H	-1.285118	4.635437	5.283926
H	-1.550014	3.008671	5.969950
H	-1.886453	3.317411	4.240535
H	2.506118	2.684322	4.185597

Aryl Substituent: 3-OMe Intermediate 14 Conformer 3

C	8.565616	-0.881651	3.984566
C	8.156245	-0.538414	2.562611
O	6.747801	-0.147078	2.508241
C	6.456958	1.133179	2.740054
O	7.266829	2.016702	2.949082
C	4.941729	1.353207	2.713350
C	4.275086	0.624193	3.922902
O	4.914558	0.997236	5.131213
C	2.757474	0.817381	3.950132
C	2.113455	1.221266	5.128224
C	0.726645	1.370364	5.142971
C	-0.042919	1.120757	4.003578
C	0.600174	0.708132	2.828454
O	-0.038366	0.426414	1.655527

C	-1.460242	0.561995	1.610603
C	1.995201	0.558721	2.807909
C	4.675190	2.848299	2.747759
O	4.544359	3.499843	3.760491
C	4.541847	3.542675	1.371986
F	5.589155	3.226983	0.585241
F	3.419091	3.100172	0.767318
F	4.472886	4.869505	1.489224
H	9.604330	-1.227981	3.986462
H	7.935693	-1.678755	4.390434
H	8.493914	-0.006297	4.636138
H	8.213416	-1.405459	1.902697
H	8.756052	0.274533	2.149416
H	4.562842	0.928104	1.779552
H	4.491700	-0.441119	3.792942
H	4.818361	1.960095	5.219938
H	2.694480	1.407074	6.024146
H	0.230042	1.685593	6.056711
H	-1.118702	1.244026	4.040965
H	-1.754016	0.292008	0.595409
H	-1.769553	1.593102	1.817554
H	-1.948349	-0.113022	2.323172
H	2.453116	0.227318	1.880426

Aryl Substituent: 3-OMe Intermediate 14 Conformer 4

C	8.782644	-0.379432	1.155507
C	8.160988	-0.600319	2.523778
O	6.744499	-0.233808	2.519886
C	6.440198	1.042920	2.754673
O	7.241716	1.934737	2.960108
C	4.922157	1.244919	2.743612
C	4.268790	0.506467	3.955035
O	4.901284	0.891735	5.163089
C	2.748299	0.675586	3.978754
C	2.096307	1.106772	5.142425
C	0.707348	1.236349	5.149728
C	-0.055862	0.940066	4.017312
C	0.595657	0.499284	2.857006
O	-0.036364	0.170977	1.692639
C	-1.459906	0.285048	1.640947
C	1.992583	0.369436	2.843932
C	4.639633	2.737089	2.781235
O	4.505960	3.386128	3.795196
C	4.492301	3.431999	1.407111
F	5.534681	3.121859	0.611580

F	3.366763	2.984843	0.811292
F	4.418193	4.758370	1.526128
H	9.818863	-0.732886	1.170658
H	8.785944	0.681755	0.891577
H	8.239971	-0.936214	0.385766
H	8.665088	-0.020511	3.299270
H	8.157284	-1.655354	2.801947
H	4.541034	0.817656	1.811617
H	4.501922	-0.555526	3.826645
H	4.797304	1.854316	5.246279
H	2.671825	1.328064	6.033844
H	0.204084	1.573031	6.052096
H	-1.133355	1.049283	4.048965
H	-1.747730	-0.021842	0.634575
H	-1.783757	1.317935	1.813865
H	-1.940135	-0.373120	2.374327
H	2.458492	0.014187	1.929195

Aryl Substituent: 3-OMe Intermediate 14 Conformer 5

C	2.211370	0.022295	-1.164542
C	3.710511	0.175776	-0.971329
O	4.009663	0.907447	0.259060
C	4.077125	0.202248	1.392532
O	3.895505	-0.995944	1.484850
C	4.435911	1.113520	2.570419
C	4.041767	0.464728	3.933432
O	4.760986	1.098614	4.991622
C	2.538282	0.473755	4.154083
C	1.842909	-0.734310	4.307670
C	0.468323	-0.716123	4.541292
C	-0.233874	0.489665	4.628990
C	0.460676	1.697992	4.476059
O	-0.113539	2.933847	4.539159
C	-1.521227	3.014795	4.773903
C	1.842819	1.682871	4.235832
C	5.914871	1.499500	2.563782
O	6.300410	2.643069	2.622620
C	6.973919	0.368632	2.452074
F	6.752031	-0.618566	3.341031
F	6.912731	-0.170439	1.212819
F	8.206073	0.843536	2.644544
H	2.020919	-0.461502	-2.128163
H	1.716513	0.998060	-1.165234
H	1.774564	-0.597705	-0.376892
H	4.158424	0.788813	-1.755011

H	4.216949	-0.790657	-0.930684
H	3.908382	2.061782	2.437184
H	4.388720	-0.568350	3.923916
H	4.384241	1.978472	5.142260
H	2.376364	-1.677744	4.241750
H	-0.071744	-1.651389	4.660118
H	-1.301743	0.478108	4.811849
H	-1.760877	4.078813	4.785192
H	-1.790687	2.569852	5.738687
H	-2.087474	2.521587	3.975392
H	2.345505	2.639552	4.114935

Aryl Substituent: 3-OMe Intermediate 15 Conformer 0

C	2.283665	1.213805	0.502459
C	3.009414	-0.107538	0.350300
O	3.575230	-0.141162	-0.994296
C	4.295832	-1.219700	-1.313194
O	4.526352	-2.146291	-0.556486
C	4.798162	-1.152935	-2.729247
C	3.988270	-1.205473	-3.822016
C	2.545314	-1.378919	-3.921148
C	1.924011	-0.966105	-5.121334
C	0.547473	-1.091743	-5.268829
C	-0.235816	-1.652532	-4.254527
C	0.378053	-2.095862	-3.071945
O	-0.281023	-2.680061	-2.033715
C	-1.694717	-2.867827	-2.145560
C	1.760896	-1.956093	-2.908373
C	6.249715	-1.032820	-2.983329
O	6.758786	-1.020705	-4.092242
C	7.209547	-0.911934	-1.767731
F	6.718230	-0.071906	-0.829301
F	7.396873	-2.115564	-1.193797
F	8.401262	-0.444237	-2.156231
H	1.843563	1.269994	1.502827
H	1.479427	1.307451	-0.233454
H	2.971975	2.055483	0.380690
H	2.336488	-0.963246	0.461049
H	3.826014	-0.217419	1.069118
H	4.503568	-1.100983	-4.775556
H	2.525958	-0.538360	-5.917225
H	0.068346	-0.761338	-6.185608
H	-1.305185	-1.752129	-4.397647
H	-2.003731	-3.351512	-1.218346
H	-1.944176	-3.513158	-2.995425

H	-2.216176	-1.909573	-2.249268
H	2.203402	-2.351608	-2.001615

Aryl Substituent: 3-OMe Intermediate 15 Conformer 1

C	4.375723	0.573588	1.541856
C	3.246987	0.054453	0.666342
O	3.659851	0.006826	-0.734444
C	4.270700	-1.105177	-1.156814
O	4.508188	-2.079077	-0.464470
C	4.645573	-1.010236	-2.610206
C	3.742946	-0.948465	-3.627064
C	2.287470	-1.008980	-3.601879
C	1.538096	-1.574906	-2.545676
C	0.151410	-1.592191	-2.622409
C	-0.524569	-1.051408	-3.723871
C	0.211531	-0.501232	-4.782652
O	-0.333987	0.039668	-5.906656
C	-1.759097	0.048937	-6.030844
C	1.610997	-0.498520	-4.722916
C	6.074604	-0.987054	-2.990717
O	6.483158	-0.964868	-4.140227
C	7.146143	-0.987679	-1.865003
F	6.808674	-0.149362	-0.858431
F	7.290138	-2.222873	-1.349600
F	8.331030	-0.595554	-2.346795
H	4.023773	0.652885	2.575609
H	4.700878	1.564425	1.210948
H	5.232765	-0.104747	1.519824
H	2.389901	0.730034	0.666601
H	2.924204	-0.943411	0.969787
H	4.181664	-0.838734	-4.617624
H	2.031966	-2.040980	-1.701700
H	-0.425780	-2.039221	-1.818469
H	-1.607405	-1.077225	-3.750856
H	-1.970391	0.518910	-6.991883
H	-2.223989	0.632567	-5.228123
H	-2.165197	-0.968968	-6.024275
H	2.166747	-0.079125	-5.556263

Aryl Substituent: 3-OMe Intermediate 15 Conformer 2

C	4.547122	0.499402	1.594330
C	3.379642	0.025347	0.744459
O	3.746723	-0.001584	-0.669564
C	4.321321	-1.116254	-1.133647
O	4.560697	-2.109015	-0.469311
C	4.652024	-0.997874	-2.596084

C	3.718813	-0.901850	-3.582156
C	2.264114	-0.940775	-3.511748
C	1.539404	-1.514027	-2.442339
C	0.150894	-1.508403	-2.475180
C	-0.550705	-0.937463	-3.544939
C	0.160313	-0.379832	-4.616853
O	-0.411761	0.189556	-5.713236
C	-1.839782	0.222335	-5.791910
C	1.560828	-0.399762	-4.601567
C	6.068527	-0.988653	-3.021424
O	6.440944	-0.945414	-4.182559
C	7.174717	-1.034927	-1.930831
F	6.884689	-0.214468	-0.895191
F	7.311350	-2.284314	-1.448643
F	8.351103	-0.653216	-2.440888
H	4.229219	0.564862	2.640020
H	4.885413	1.489277	1.273852
H	5.386682	-0.197977	1.531589
H	2.539305	0.720394	0.786220
H	3.042787	-0.971009	1.037428
H	4.127710	-0.779005	-4.583869
H	2.052034	-2.002994	-1.622862
H	-0.407770	-1.960771	-1.661205
H	-1.634121	-0.945958	-3.537827
H	-2.073937	0.711564	-6.737925
H	-2.270025	0.799278	-4.965392
H	-2.261190	-0.789338	-5.789092
H	2.096714	0.025967	-5.444655

Aryl Substituent: 3-OMe Intermediate 15 Conformer 3

C	2.721209	1.095634	1.002025
C	3.356954	-0.241956	0.687565
O	3.804311	-0.198954	-0.700545
C	4.356630	-1.310451	-1.190656
O	4.522772	-2.338196	-0.554009
C	4.747167	-1.141814	-2.630897
C	3.848605	-0.994755	-3.639147
C	2.389761	-1.020573	-3.610507
C	1.635698	-1.659317	-2.600874
C	0.249129	-1.639466	-2.663331
C	-0.423398	-0.989881	-3.705306
C	0.315732	-0.365513	-4.718927
O	-0.222884	0.284504	-5.784829
C	-1.640764	0.340833	-5.898462
C	1.715555	-0.399796	-4.673256

C	6.185231	-1.125973	-3.012872
O	6.581343	-1.010637	-4.156331
C	7.247150	-1.263075	-1.888738
F	6.923545	-0.489311	-0.824450
F	7.318917	-2.539601	-1.468799
F	8.449535	-0.889183	-2.325187
H	2.380667	1.096820	2.041164
H	1.857941	1.281403	0.356730
H	3.438252	1.911716	0.876374
H	2.650668	-1.070342	0.797434
H	4.224939	-0.447256	1.320782
H	4.295365	-0.836932	-4.620147
H	2.127900	-2.210239	-1.808537
H	-0.331187	-2.143749	-1.896279
H	-1.506717	-0.990492	-3.723437
H	-1.845534	0.900387	-6.811911
H	-2.092519	0.863073	-5.045898
H	-2.078091	-0.661828	-5.982751
H	2.268516	0.081222	-5.474292

Aryl Substituent: 3-OMe Intermediate 15 Conformer 4

C	2.913134	1.057462	1.128795
C	3.489248	-0.292084	0.751068
O	3.875833	-0.222587	-0.654155
C	4.423657	-1.322428	-1.178507
O	4.641724	-2.348917	-0.559598
C	4.749170	-1.139497	-2.635181
C	3.810438	-0.960275	-3.604529
C	2.355985	-0.956248	-3.517956
C	1.626300	-1.567382	-2.474136
C	0.238292	-1.514319	-2.487036
C	-0.456643	-0.858072	-3.511245
C	0.259620	-0.261647	-4.558579
O	-0.306035	0.390749	-5.611099
C	-1.732798	0.479747	-5.665344
C	1.658670	-0.329147	-4.564520
C	6.161093	-1.155403	-3.074572
O	6.524078	-1.066532	-4.236153
C	7.274393	-1.291330	-1.999298
F	7.017339	-0.520262	-0.919081
F	7.378348	-2.568639	-1.586275
F	8.457302	-0.917360	-2.500497
H	2.612443	1.039121	2.180861
H	2.032973	1.291956	0.522644
H	3.654080	1.851422	0.995756

H	2.760181	-1.099534	0.867462
H	4.376724	-0.543194	1.338587
H	4.213150	-0.796931	-4.602923
H	2.132098	-2.121958	-1.692903
H	-0.325432	-1.995918	-1.693576
H	-1.539577	-0.831487	-3.489425
H	-1.961231	1.034452	-6.575975
H	-2.130321	1.019910	-4.798584
H	-2.191300	-0.514277	-5.717883
H	2.198583	0.127687	-5.388555

Aryl Substituent: 3-OMe Intermediate 16 Conformer 1

C	8.734058	-0.393589	-0.523814
C	8.245492	-0.695210	-1.929564
O	6.841209	-1.114615	-1.915685
C	6.587655	-2.404332	-1.708396
O	7.418438	-3.272390	-1.519797
C	5.074105	-2.671705	-1.736661
C	4.440476	-2.265484	-3.100596
N	5.073386	-3.019020	-4.181791
C	5.139900	-2.633219	-5.477323
N	4.618975	-1.441911	-5.811509
S	5.900945	-3.634631	-6.641463
C	2.913603	-2.363064	-3.070256
C	2.232962	-3.500034	-3.530155
C	0.841647	-3.544230	-3.448819
C	0.110337	-2.478493	-2.916180
C	0.792598	-1.342293	-2.459721
O	0.192070	-0.239731	-1.929093
C	-1.234236	-0.229557	-1.826265
C	2.192811	-1.290008	-2.542975
C	4.855581	-4.133147	-1.364512
O	4.869269	-5.056081	-2.146427
C	4.589887	-4.405878	0.134980
F	5.510422	-3.782819	0.895776
F	3.377366	-3.908703	0.456106
F	4.608662	-5.709917	0.411022
H	9.759783	-0.014093	-0.575674
H	8.732740	-1.294706	0.095592
H	8.108606	0.367482	-0.048040
H	8.832943	-1.480903	-2.408070
H	8.243534	0.195190	-2.559736
H	4.618892	-2.032283	-0.973783
H	4.715260	-1.213090	-3.222978
H	5.347457	-3.974639	-3.991870

H	4.034455	-0.899554	-5.190979
H	4.665082	-1.150570	-6.776207
H	2.780760	-4.335189	-3.953000
H	0.310536	-4.421783	-3.806844
H	-0.970263	-2.539533	-2.867062
H	-1.493866	0.732489	-1.382958
H	-1.592250	-1.038483	-1.179257
H	-1.704842	-0.316682	-2.812216
H	2.698856	-0.394590	-2.190801

Aryl Substituent: 3-OMe Intermediate 16 Conformer 2

C	8.740854	-0.423383	-0.447977
C	8.273368	-0.730007	-1.859783
O	6.867474	-1.144351	-1.865601
C	6.606020	-2.432294	-1.658035
O	7.430663	-3.302913	-1.454591
C	5.092124	-2.694892	-1.704090
C	4.468032	-2.261771	-3.063816
N	5.102266	-3.000801	-4.154419
C	5.178252	-2.593803	-5.442808
N	4.666757	-1.393379	-5.759362
S	5.939900	-3.580008	-6.619379
C	2.940409	-2.350301	-3.044907
C	2.256673	-3.481120	-3.515784
C	0.864585	-3.517410	-3.446199
C	0.135204	-2.449577	-2.915015
C	0.820324	-1.319515	-2.447998
O	0.222059	-0.215578	-1.917841
C	-1.205210	-0.194235	-1.833202
C	2.221610	-1.275514	-2.518814
C	4.867955	-4.162589	-1.360093
O	4.887637	-5.071139	-2.158668
C	4.588048	-4.461996	0.131741
F	5.505150	-3.858666	0.911999
F	3.375465	-3.964107	0.451539
F	4.597166	-5.771096	0.383211
H	9.768031	-0.046144	-0.485597
H	8.728067	-1.321808	0.175197
H	8.109765	0.341018	0.014829
H	8.865182	-1.520246	-2.325199
H	8.284418	0.157270	-2.494222
H	4.633103	-2.068634	-0.932298
H	4.749768	-1.209265	-3.166852
H	5.370589	-3.960605	-3.978035
H	4.076952	-0.861161	-5.135014

H	4.716257	-1.088696	-6.719763
H	2.802604	-4.317339	-3.938903
H	0.331271	-4.390179	-3.812493
H	-0.946121	-2.504353	-2.875362
H	-1.462818	0.768619	-1.390459
H	-1.578114	-1.001972	-1.193134
H	-1.663836	-0.274900	-2.825344
H	2.729997	-0.384961	-2.157861

Aryl Substituent: 3-OMe Intermediate 16 Conformer 3

C	8.697557	-0.376400	-0.618155
C	8.181803	-0.650961	-2.019668
O	6.782153	-1.084882	-1.984777
C	6.545377	-2.381671	-1.804027
O	7.387966	-3.246178	-1.655894
C	5.033959	-2.662764	-1.804508
C	4.365262	-2.229719	-3.142870
N	4.976526	-2.954016	-4.256299
C	4.999896	-2.541920	-5.545138
N	4.457758	-1.348422	-5.837860
S	5.738350	-3.509430	-6.751197
C	2.840081	-2.336874	-3.079025
C	2.155449	-3.472278	-3.537293
C	0.766674	-3.525321	-3.425566
C	0.041434	-2.469968	-2.864469
C	0.727383	-1.335316	-2.410038
O	0.133049	-0.242204	-1.853700
C	-1.290890	-0.239297	-1.723070
C	2.125238	-1.274289	-2.523348
C	4.837000	-4.134455	-1.461956
O	4.840198	-5.038842	-2.265378
C	4.609577	-4.444694	0.036650
F	5.546798	-3.837216	0.789319
F	3.403852	-3.960033	0.400008
F	4.639052	-5.755076	0.279748
H	9.717698	0.015922	-0.683038
H	8.719513	-1.291612	-0.020149
H	8.074329	0.366228	-0.111343
H	8.766075	-1.419805	-2.528446
H	8.157267	0.253284	-2.629285
H	4.590681	-2.046042	-1.016184
H	4.630750	-1.173036	-3.248233
H	5.262830	-3.911123	-4.093668
H	3.866545	-0.839654	-5.195345
H	4.460585	-1.044853	-6.799979

H	2.698021	-4.299237	-3.982389
H	0.232350	-4.401604	-3.781888
H	-1.037446	-2.538025	-2.792362
H	-1.545908	0.716920	-1.264719
H	-1.633144	-1.056402	-1.077790
H	-1.780207	-0.318234	-2.700599
H	2.633757	-0.380201	-2.171370

Aryl Substituent: 3-OMe Intermediate 16 Conformer 4

C	8.658047	-0.318712	-0.699580
C	8.155184	-0.650083	-2.093569
O	6.758396	-1.091602	-2.052827
C	6.528333	-2.382098	-1.825361
O	7.375496	-3.234956	-1.639913
C	5.018924	-2.673872	-1.819631
C	4.338516	-2.254335	-3.156035
N	4.942353	-2.987451	-4.267917
C	4.948327	-2.590040	-5.561447
N	4.398583	-1.401720	-5.860980
S	5.674294	-3.569036	-6.765993
C	2.814210	-2.365468	-3.079611
C	2.131203	-3.512870	-3.509197
C	0.743211	-3.568600	-3.388116
C	0.017530	-2.503826	-2.845896
C	0.701818	-1.357107	-2.420045
O	0.106537	-0.253851	-1.885166
C	-1.316259	-0.254834	-1.742424
C	2.098818	-1.293773	-2.542389
C	4.836351	-4.145669	-1.466693
O	4.854117	-5.055734	-2.263592
C	4.603483	-4.447283	0.032599
F	5.526858	-3.821386	0.787131
F	3.388794	-3.975257	0.382492
F	4.648333	-5.754988	0.286732
H	9.677184	0.074968	-0.771653
H	8.678838	-1.209843	-0.066171
H	8.027776	0.440423	-0.227151
H	8.748709	-1.433759	-2.567830
H	8.130171	0.230032	-2.737530
H	4.575686	-2.055810	-1.032066
H	4.600156	-1.197950	-3.272715
H	5.236821	-3.940722	-4.097559
H	3.817369	-0.885594	-5.215262
H	4.385780	-1.110409	-6.826767
H	2.673648	-4.347265	-3.940470

H	0.210384	-4.454356	-3.722534
H	-1.060720	-2.573979	-2.766454
H	-1.571960	0.709543	-1.301900
H	-1.649121	-1.060130	-1.077742
H	-1.813542	-0.355977	-2.713882
H	2.606561	-0.390823	-2.212687

Aryl Substituent: 3-OMe Intermediate 16 Conformer 5

C	4.707772	-1.109624	2.406283
C	3.487787	-1.711384	1.729978
O	3.866517	-2.441032	0.518239
C	3.971266	-1.743024	-0.612858
O	3.757460	-0.553454	-0.735122
C	4.432091	-2.656277	-1.757156
C	3.956099	-2.100167	-3.131943
N	4.680822	-2.821343	-4.183956
C	4.822232	-2.413714	-5.470418
N	4.332461	-1.211316	-5.808151
S	5.642836	-3.401536	-6.601978
C	2.438582	-2.191019	-3.278655
C	1.810899	-3.430746	-3.484341
C	0.425990	-3.483352	-3.617857
C	-0.354209	-2.322677	-3.553024
C	0.273362	-1.087930	-3.348800
O	-0.375136	0.108191	-3.269816
C	-1.798494	0.115725	-3.404846
C	1.671089	-1.028819	-3.211261
C	5.950469	-2.871300	-1.721681
O	6.456102	-3.966511	-1.792529
C	6.877920	-1.633075	-1.581926
F	6.530694	-0.661002	-2.448509
F	6.771381	-1.134671	-0.330950
F	8.150953	-1.963187	-1.799251
H	4.399315	-0.640244	3.346084
H	5.172386	-0.346277	1.777043
H	5.448498	-1.881991	2.633491
H	2.753434	-0.951042	1.457768
H	3.012230	-2.470752	2.352305
H	4.010126	-3.651814	-1.597151
H	4.254147	-1.050197	-3.171424
H	4.938512	-3.786010	-4.020383
H	3.707888	-0.683646	-5.214235
H	4.428863	-0.905734	-6.764875
H	2.397927	-4.342280	-3.550103
H	-0.064060	-4.439531	-3.778466

H	-1.429773	-2.392609	-3.663026
H	-2.101183	1.159158	-3.309361
H	-2.276021	-0.476971	-2.616227
H	-2.107314	-0.266382	-4.384589
H	2.135478	-0.062434	-3.038827

Aryl Substituent: 3-OMe Intermediate 2 Conformer 0

H	2.464472	1.058553	-0.170040
C	2.033563	0.093195	0.165259
O	0.959028	-0.294255	-0.274598
C	2.853636	-0.638305	1.149794
C	2.410319	-1.876522	1.650654
C	3.195649	-2.556952	2.581130
O	2.873879	-3.757742	3.137215
C	1.648961	-4.378186	2.737579
C	4.418220	-1.997381	3.005720
C	4.847047	-0.772688	2.504349
C	4.066908	-0.082457	1.570947
H	1.464412	-2.269093	1.297304
H	1.597040	-5.317564	3.288995
H	1.641033	-4.584023	1.661125
H	0.785406	-3.754053	2.994665
H	5.013622	-2.543760	3.731151
H	5.791335	-0.356669	2.842650
H	4.393471	0.874903	1.174101

Aryl Substituent: 3-OMe Intermediate 2 Conformer 1

H	2.464724	1.058301	-0.170472
C	2.033172	0.093601	0.165881
O	0.957906	-0.293140	-0.272815
C	2.853325	-0.637981	1.150293
C	2.409200	-1.875398	1.652406
C	3.194584	-2.555907	2.582778
O	2.872089	-3.755983	3.139994
C	1.645977	-4.375247	2.742212
C	4.418026	-1.997218	3.006002
C	4.847663	-0.773316	2.503385
C	4.067469	-0.083013	1.570083
H	1.462638	-2.267312	1.300079
H	1.593336	-5.313966	3.294687
H	1.636746	-4.582274	1.665996
H	0.783490	-3.749705	2.999453
H	5.013468	-2.543612	3.731388
H	5.792612	-0.357962	2.840659
H	4.394675	0.873733	1.172289

Aryl Substituent: 3-OMe Intermediate 2 Conformer 2

H	1.093617	-0.433560	-0.099622
C	2.034110	0.088920	0.171263
O	2.311782	1.169165	-0.331719
C	2.874832	-0.622118	1.154930
C	2.423619	-1.858070	1.649455
C	3.201469	-2.551751	2.582426
O	2.866546	-3.753062	3.130063
C	1.635748	-4.359716	2.727136
C	4.422654	-2.001875	3.010642
C	4.860338	-0.774786	2.513028
C	4.092141	-0.075032	1.582630
H	1.476609	-2.254123	1.298620
H	1.573013	-5.299564	3.276586
H	1.627275	-4.563872	1.650310
H	0.778200	-3.727436	2.984603
H	5.013099	-2.554177	3.735644
H	5.806731	-0.367733	2.857068
H	4.415717	0.880992	1.184535

Aryl Substituent: 3-OMe Intermediate 2 Conformer 3

H	2.414976	1.053407	-0.125022
C	2.064479	0.031872	0.126882
O	1.059943	-0.428828	-0.399335
C	2.897051	-0.678594	1.116541
C	2.552016	-1.982847	1.516763
C	3.348073	-2.642173	2.453237
O	3.118012	-3.901437	2.918054
C	1.979538	-4.606371	2.415542
C	4.483344	-1.995648	2.983768
C	4.815654	-0.706252	2.581289
C	4.023610	-0.036601	1.642970
H	1.671295	-2.441215	1.083269
H	1.988396	-5.578280	2.910054
H	2.046884	-4.746774	1.330657
H	1.048880	-4.080310	2.657151
H	5.087901	-2.528486	3.711750
H	5.693646	-0.223788	3.000287
H	4.274002	0.970928	1.322144

Aryl Substituent: 3-OMe Intermediate 2 Conformer 4

H	2.468301	1.002411	-0.204280
C	1.997183	0.099831	0.235319
O	0.867049	-0.235328	-0.094406
C	2.836548	-0.619219	1.212904
C	2.346510	-1.780751	1.838269
C	3.150673	-2.449854	2.760816

O	2.789459	-3.579910	3.429575
C	1.491897	-4.125636	3.177073
C	4.438536	-1.955529	3.052799
C	4.913441	-0.806565	2.428794
C	4.114671	-0.128317	1.502452
H	1.351217	-2.124668	1.583580
H	1.413183	-5.011660	3.807952
H	1.379973	-4.413422	2.125491
H	0.702925	-3.413747	3.445519
H	5.046600	-2.492595	3.774720
H	5.907694	-0.440074	2.666181
H	4.475937	0.770231	1.009741

Aryl Substituent: 3-OMe Intermediate 3 Conformer 0

H	2.593270	-0.692502	0.153365
C	1.845445	0.033492	-0.186922
O	1.706802	-0.009739	-1.595889
N	0.585878	-0.274891	0.498409
C	0.064099	-1.506414	0.710062
N	0.816950	-2.579937	0.408891
S	-1.522765	-1.683828	1.330648
C	2.298911	1.424412	0.214770
C	1.788909	2.540977	-0.463392
C	2.178621	3.826347	-0.068190
O	1.746739	4.976420	-0.661763
C	0.831227	4.872486	-1.754161
C	3.074432	3.990204	1.002013
C	3.572758	2.873784	1.665615
C	3.189388	1.584676	1.277805
H	1.488385	-0.917153	-1.855194
H	-0.057829	0.493044	0.640897
H	1.800043	-2.516731	0.181833
H	0.463664	-3.491141	0.659669
H	1.112674	2.390379	-1.295391
H	0.621603	5.896272	-2.066636
H	-0.101870	4.386076	-1.447157
H	1.270654	4.318357	-2.591710
H	3.366956	4.995331	1.290450
H	4.268956	3.006973	2.488822
H	3.582100	0.715106	1.797155

Aryl Substituent: 3-OMe Intermediate 3 Conformer 1

H	2.677732	-0.718616	0.171014
C	1.882176	-0.004706	-0.073315
O	1.559482	-0.064365	-1.450813
N	0.727703	-0.319106	0.775774

C	0.248659	-1.552563	1.063035
N	0.964623	-2.622789	0.673462
S	-1.241021	-1.736831	1.888519
C	2.365583	1.395886	0.253738
C	1.763494	2.498571	-0.369450
C	2.181899	3.792837	-0.037707
O	1.665756	4.930860	-0.585441
C	0.623852	4.803960	-1.555340
C	3.199029	3.979458	0.913761
C	3.788082	2.876609	1.523487
C	3.376214	1.578798	1.199148
H	1.329715	-0.978468	-1.674182
H	0.103902	0.445505	1.000386
H	1.908614	-2.553319	0.318614
H	0.655072	-3.534820	0.973965
H	0.993403	2.330716	-1.111648
H	0.364400	5.821675	-1.849699
H	-0.258058	4.310458	-1.130723
H	0.963481	4.245189	-2.435136
H	3.511589	4.991209	1.154033
H	4.577453	3.027178	2.254474
H	3.839664	0.720176	1.676743

Aryl Substituent: 3-OMe Intermediate 3 Conformer 2

H	2.861990	-0.666275	0.361925
C	2.018968	-0.005754	0.127721
O	1.634375	-0.143780	-1.227196
N	0.924923	-0.339489	1.045855
C	0.551449	-1.576561	1.451231
N	1.331865	-2.621900	1.127577
S	-0.892293	-1.795871	2.348886
C	2.445052	1.430888	0.371809
C	1.806354	2.472055	-0.316242
C	2.173758	3.798390	-0.057475
O	1.619548	4.882518	-0.673529
C	0.591331	4.659914	-1.640773
C	3.175516	4.078063	0.886993
C	3.801207	3.034968	1.562398
C	3.441323	1.706366	1.310625
H	1.434400	-1.076210	-1.396651
H	0.251269	0.392112	1.234105
H	2.247692	-2.520169	0.712507
H	1.095017	-3.527082	1.504786
H	1.047349	2.232945	-1.050059
H	0.295130	5.648249	-1.994572

H	-0.274202	4.157315	-1.193862
H	0.959573	4.065596	-2.485073
H	3.447641	5.113233	1.069871
H	4.578616	3.257144	2.287943
H	3.933249	0.894534	1.838999

Aryl Substituent: 3-OMe Intermediate 3 Conformer 3

H	2.574947	-0.697175	0.093809
C	1.847818	0.039572	-0.267228
O	1.771183	0.018095	-1.681417
N	0.556234	-0.265243	0.356293
C	0.029983	-1.490896	0.587893
N	0.791003	-2.571629	0.344637
S	-1.575690	-1.650437	1.165542
C	2.293114	1.421610	0.173313
C	1.799966	2.551896	-0.494905
C	2.177490	3.828856	-0.062591
O	1.757677	4.990820	-0.641248
C	0.865231	4.909822	-1.754431
C	3.046638	3.970833	1.032562
C	3.529344	2.840995	1.684662
C	3.156289	1.559865	1.261431
H	1.554322	-0.882369	-1.964878
H	-0.088119	0.506515	0.472160
H	1.773426	-2.515121	0.114970
H	0.427381	-3.478059	0.596999
H	1.145664	2.418796	-1.347300
H	0.661354	5.940049	-2.048954
H	-0.073532	4.416399	-1.477097
H	1.322028	4.373583	-2.594287
H	3.330887	4.969694	1.349565
H	4.205106	2.957285	2.527261
H	3.536156	0.680046	1.772939

Aryl Substituent: 3-OMe Intermediate 3 Conformer 4

H	3.154285	-0.657197	0.189878
C	2.248297	-0.046216	0.102283
O	1.677259	-0.176945	-1.185390
N	1.323986	-0.471469	1.158759
C	1.102666	-1.737140	1.586050
N	1.890113	-2.720846	1.117283
S	-0.164646	-2.070212	2.690938
C	2.620030	1.408272	0.329633
C	1.844742	2.424023	-0.246363
C	2.168111	3.764039	0.000962
O	1.483065	4.826193	-0.513430

C	0.359414	4.564097	-1.356574
C	3.262108	4.082488	0.822668
C	4.023190	3.063950	1.387767
C	3.708571	1.722163	1.146148
H	1.511034	-1.115934	-1.354528
H	0.644370	0.210422	1.471037
H	2.722875	-2.548784	0.570998
H	1.780348	-3.644099	1.508928
H	1.014944	2.155682	-0.887367
H	-0.030092	5.540742	-1.646738
H	-0.417810	4.004748	-0.823088
H	0.654956	4.008051	-2.253847
H	3.497733	5.127719	0.998737
H	4.871312	3.316053	2.018084
H	4.306441	0.929898	1.587872

Aryl Substituent: 3-OMe Intermediate 4 Conformer 0

H	2.484573	0.109774	-0.998853
C	2.071774	-0.068954	0.000594
N	0.955910	-0.695731	0.150807
C	0.234194	-1.106804	-0.971958
N	-0.453564	-2.228223	-0.744461
S	0.146449	-0.270926	-2.449726
C	2.852593	0.432901	1.126171
C	2.411670	0.262326	2.455224
C	3.183639	0.762074	3.502916
O	2.860336	0.656737	4.821388
C	1.643911	-0.011665	5.166379
C	4.394465	1.430274	3.226547
C	4.824077	1.594004	1.914241
C	4.058768	1.096655	0.855637
H	-0.382805	-2.692026	0.152455
H	-1.030185	-2.636169	-1.465954
H	1.478875	-0.258563	2.630985
H	1.585852	0.018660	6.254886
H	1.654511	-1.054300	4.828680
H	0.774569	0.500578	4.738478
H	4.978206	1.808990	4.060176
H	5.758498	2.110186	1.715939
H	4.388682	1.221201	-0.171553

Aryl Substituent: 3-OMe Intermediate 4 Conformer 1

H	2.468620	0.116259	-0.996229
C	2.054700	-0.054849	0.004068
N	0.929507	-0.664535	0.156685
C	0.200816	-1.065979	-0.965784

N	-0.521051	-2.163523	-0.727601
S	0.144310	-0.247675	-2.454884
C	2.844279	0.436310	1.128021
C	2.401355	0.275753	2.457725
C	3.182040	0.764903	3.503935
O	2.857687	0.667923	4.822778
C	1.630251	0.021023	5.169794
C	4.403640	1.412372	3.225487
C	4.835182	1.566301	1.912620
C	4.061101	1.079606	0.855455
H	-0.466197	-2.618346	0.175055
H	-1.104697	-2.564770	-1.447204
H	1.460094	-0.229122	2.634975
H	1.573060	0.055075	6.258238
H	1.623116	-1.022507	4.834788
H	0.769565	0.546818	4.740848
H	4.994011	1.783212	4.057991
H	5.777918	2.066517	1.712809
H	4.392393	1.196633	-0.172170

Aryl Substituent: 3-OMe Intermediate 4 Conformer 2

H	1.215661	-0.507771	0.218267
C	2.157352	-0.007298	-0.036014
N	2.520996	0.125527	-1.264814
C	1.736858	-0.422031	-2.283072
N	1.811444	0.288708	-3.410821
S	0.840407	-1.862372	-2.174498
C	2.954144	0.491759	1.080765
C	2.451012	0.299570	2.382888
C	3.183346	0.753506	3.484143
O	2.791722	0.619143	4.781482
C	1.544390	-0.027534	5.048832
C	4.414971	1.399026	3.279166
C	4.905982	1.587447	1.987348
C	4.185019	1.139699	0.881750
H	2.360942	1.138393	-3.437567
H	1.301886	0.004611	-4.234884
H	1.498740	-0.202348	2.511339
H	1.434365	-0.024795	6.133771
H	1.546273	-1.061191	4.684410
H	0.708787	0.517794	4.595311
H	4.969947	1.745981	4.145588
H	5.858842	2.089523	1.848069
H	4.556514	1.284254	-0.126661

Aryl Substituent: 3-OMe Intermediate 4 Conformer 3

H	2.449828	0.194891	-1.003047
C	2.033918	0.017347	-0.004615
N	0.895974	-0.569470	0.141630
C	0.206293	-1.035494	-0.980010
N	-1.116948	-0.993498	-0.808640
S	0.921352	-1.664875	-2.388090
C	2.832704	0.482512	1.124201
C	2.399116	0.293512	2.453262
C	3.195014	0.745108	3.504785
O	2.880820	0.617580	4.823510
C	1.648070	-0.021834	5.164650
C	4.420820	1.386858	3.232311
C	4.841588	1.571680	1.919913
C	4.052284	1.122446	0.857316
H	-1.503550	-0.620768	0.049327
H	-1.741901	-1.312936	-1.534491
H	1.451028	-0.200105	2.625799
H	1.601050	-0.016390	6.254153
H	1.623683	-1.055978	4.802452
H	0.790660	0.526779	4.758322
H	5.022611	1.731717	4.067789
H	5.787379	2.067987	1.724733
H	4.376405	1.263631	-0.169559

Aryl Substituent: 3-OMe Intermediate 4 Conformer 4

H	2.422083	0.151095	-0.990097
C	1.997541	0.013876	0.011012
N	0.830706	-0.510538	0.166627
C	0.072281	-0.860309	-0.952910
N	-0.719540	-1.908623	-0.715035
S	0.063845	-0.034341	-2.438870
C	2.822847	0.448723	1.132740
C	2.370704	0.325681	2.463247
C	3.186052	0.759011	3.507595
O	2.856213	0.690270	4.826853
C	1.583781	0.138987	5.176638
C	4.451947	1.313226	3.226366
C	4.892452	1.430367	1.912572
C	4.083672	0.999144	0.857292
H	-0.691991	-2.368666	0.186165
H	-1.332360	-2.265742	-1.433652
H	1.395018	-0.107911	2.642681
H	1.529426	0.182424	6.264901
H	1.498216	-0.902712	4.846760
H	0.764852	0.725679	4.744951

H	5.070059	1.642463	4.056201
H	5.869581	1.858626	1.710776
H	4.422714	1.088270	-0.170612

Aryl Substituent: 3-OMe Intermediate 6 Conformer 0

C	5.232366	1.800891	-6.474811
C	4.117877	2.392502	-5.630115
O	4.586014	2.664524	-4.267663
C	4.512789	1.673562	-3.380336
O	4.105393	0.548764	-3.604572
C	5.012928	2.134348	-2.009205
C	4.291393	3.416648	-1.464753
N	4.952452	4.602311	-2.000303
C	5.978824	5.301280	-1.462743
N	6.413786	4.981679	-0.234179
S	6.706932	6.571598	-2.356323
C	2.778921	3.495305	-1.715911
C	1.991717	2.331982	-1.713968
C	0.607150	2.415345	-1.904528
O	-0.224345	1.334570	-1.934591
C	0.346820	0.031919	-1.795457
C	-0.005314	3.665761	-2.080183
C	0.775958	4.814567	-2.063956
C	2.162200	4.739583	-1.884129
C	5.008254	0.970805	-1.031857
O	4.366636	0.934067	-0.006697
C	5.955463	-0.228773	-1.324637
F	6.740860	-0.015765	-2.395948
F	6.748361	-0.432954	-0.258509
F	5.238405	-1.343350	-1.533292
H	4.874947	1.677707	-7.502364
H	5.535170	0.820340	-6.097174
H	6.103665	2.462189	-6.489813
H	3.253156	1.729327	-5.569299
H	3.804014	3.370625	-5.996696
H	6.069312	2.401723	-2.146880
H	4.426783	3.402953	-0.378806
H	4.743536	4.831931	-2.963577
H	5.966546	4.295491	0.356665
H	7.127719	5.558016	0.184978
H	2.445973	1.364857	-1.560083
H	-0.483799	-0.669332	-1.883278
H	0.824199	-0.092191	-0.816243
H	1.078574	-0.168596	-2.586853
H	-1.080138	3.710761	-2.226028

H	0.305189	5.784688	-2.195791
H	2.751314	5.649340	-1.873091

Aryl Substituent: 3-OMe Intermediate 6 Conformer 1

C	2.710483	-1.786713	-0.778983
C	3.051216	-0.593286	0.096303
O	3.369944	0.579741	-0.721109
C	4.620391	0.716760	-1.138955
O	5.538673	-0.050394	-0.899897
C	4.837047	1.951828	-2.011050
C	4.093322	3.246600	-1.556785
N	4.795649	4.373557	-2.196668
C	4.811230	5.662002	-1.761838
N	4.237606	5.938408	-0.581731
S	5.586533	6.869926	-2.690241
C	2.598264	3.247774	-1.856669
C	2.150307	3.217736	-3.186722
C	0.779059	3.262380	-3.460568
O	0.243280	3.236728	-4.713425
C	1.130688	3.156007	-5.831284
C	-0.144827	3.342719	-2.402977
C	0.308419	3.375277	-1.090117
C	1.679803	3.327659	-0.808513
C	6.351610	2.160229	-2.109912
O	6.969931	2.071220	-3.145056
C	7.157139	2.525519	-0.830007
F	6.386298	2.551153	0.278084
F	8.146478	1.642906	-0.636075
F	7.705295	3.744659	-0.979678
H	2.405010	-2.622363	-0.140887
H	3.575807	-2.102952	-1.367975
H	1.884673	-1.551135	-1.456705
H	3.899386	-0.795910	0.752675
H	2.197137	-0.268372	0.692042
H	4.515595	1.689472	-3.025771
H	4.235702	3.345025	-0.478276
H	5.077404	4.266162	-3.163390
H	3.633394	5.291292	-0.095383
H	4.251917	6.892400	-0.253224
H	2.868443	3.168379	-3.997461
H	0.494202	3.142887	-6.716793
H	1.729737	2.238684	-5.798444
H	1.797580	4.024880	-5.873886
H	-1.204467	3.379241	-2.636690
H	-0.408479	3.438107	-0.276443

H	2.025019	3.336309	0.221786

Aryl Substituent: 3-OMe Intermediate 6 Conformer 2

C	2.633915	-1.776437	-0.773493
C	3.086023	-0.628052	0.111397
O	3.432961	0.546126	-0.692966
C	4.665349	0.619610	-1.174185
O	5.546945	-0.206682	-1.003579
C	4.915236	1.863852	-2.024399
C	4.232161	3.178035	-1.533602
N	4.965036	4.288976	-2.167022
C	5.032073	5.568602	-1.713242
N	4.467943	5.848166	-0.528059
S	5.860136	6.758489	-2.618785
C	2.732323	3.242055	-1.803199
C	2.257003	3.253699	-3.124134
C	0.883116	3.354192	-3.368306
O	0.321441	3.369930	-4.609963
C	1.182111	3.268846	-5.746934
C	-0.015597	3.451107	-2.290659
C	0.464702	3.443151	-0.987121
C	1.838555	3.337573	-0.735094
C	6.435302	2.013097	-2.146292
O	7.032314	1.928277	-3.194022
C	7.274108	2.310663	-0.870553
F	6.521774	2.327699	0.250767
F	8.235234	1.389629	-0.720408
F	7.860173	3.515031	-0.992378
H	2.312850	-2.610138	-0.140497
H	3.449124	-2.125368	-1.413287
H	1.790266	-1.476736	-1.402112
H	3.951659	-0.895896	0.719903
H	2.282055	-0.270278	0.755993
H	4.567083	1.633825	-3.038181
H	4.398426	3.252884	-0.456316
H	5.243926	4.181617	-3.134247
H	3.807765	5.230630	-0.076610
H	4.507837	6.799714	-0.194284
H	2.956147	3.193730	-3.950674
H	0.528315	3.297990	-6.619329
H	1.740499	2.325567	-5.740624
H	1.885635	4.108244	-5.789972
H	-1.077506	3.531613	-2.501806
H	-0.232755	3.518448	-0.157804
H	2.204738	3.312920	0.287780

Aryl Substituent: 3-OMe Intermediate 6 Conformer 3

C	8.515418	1.357389	0.720392
C	8.018515	2.699551	0.212788
O	6.641710	2.594390	-0.277989
C	6.466654	2.220945	-1.542855
O	7.349176	1.947809	-2.334126
C	4.970854	2.172893	-1.894143
C	4.280317	3.552523	-1.681379
N	4.925749	4.562356	-2.518984
C	4.925480	5.895492	-2.284473
N	4.313426	6.343116	-1.176654
S	5.716454	6.965615	-3.364007
C	2.761309	3.462149	-1.847616
C	2.148254	3.755728	-3.074196
C	0.758851	3.633521	-3.203569
O	0.069953	3.895916	-4.349997
C	0.802074	4.321431	-5.501623
C	-0.014846	3.218290	-2.106905
C	0.602438	2.933408	-0.893925
C	1.989610	3.055390	-0.754864
C	4.848083	1.629163	-3.312409
O	4.887430	2.301494	-4.317264
C	4.646643	0.098857	-3.423958
F	5.548862	-0.551193	-2.663925
F	3.415925	-0.209076	-2.965215
F	4.756089	-0.322549	-4.684002
H	9.515520	1.484691	1.147519
H	8.580032	0.628169	-0.091921
H	7.855102	0.965727	1.499723
H	8.641639	3.088575	-0.594511
H	7.952839	3.438534	1.012497
H	4.498194	1.473435	-1.197420
H	4.493270	3.801755	-0.637059
H	5.271794	4.273671	-3.425127
H	3.715184	5.765531	-0.602637
H	4.309070	7.335796	-0.997306
H	2.753970	4.083228	-3.909659
H	0.061264	4.468053	-6.288458
H	1.524049	3.559915	-5.818634
H	1.326889	5.265274	-5.314171
H	-1.090511	3.133565	-2.227287
H	-0.000359	2.621343	-0.045874
H	2.463525	2.838229	0.198925

Aryl Substituent: 3-OMe Intermediate 6 Conformer 4

C	8.459351	0.919545	0.586555
C	8.000560	2.306804	0.173292
O	6.622479	2.274224	-0.324243
C	6.440188	1.993739	-1.611916
O	7.316799	1.752342	-2.419879
C	4.944102	2.011545	-1.965624
C	4.295331	3.396062	-1.669621
N	4.972129	4.434998	-2.444908
C	5.010737	5.750594	-2.128959
N	4.406149	6.146074	-0.996440
S	5.842325	6.860232	-3.134880
C	2.774621	3.362577	-1.840147
C	2.172682	3.743219	-3.048325
C	0.780351	3.672723	-3.183518
O	0.101687	4.020237	-4.313220
C	0.848785	4.487531	-5.438852
C	-0.007568	3.222248	-2.111011
C	0.598728	2.850909	-0.916081
C	1.988998	2.920679	-0.771235
C	4.807535	1.558284	-3.414216
O	4.870586	2.288192	-4.376909
C	4.558172	0.044822	-3.618277
F	5.437405	-0.678484	-2.898908
F	3.316884	-0.250602	-3.179003
F	4.656182	-0.302938	-4.901412
H	9.461272	0.989930	1.022502
H	8.506717	0.246963	-0.274344
H	7.786567	0.492830	1.336253
H	8.635929	2.733859	-0.604723
H	7.953140	2.989711	1.022600
H	4.449842	1.285815	-1.312134
H	4.514103	3.576557	-0.612417
H	5.315847	4.191329	-3.365030
H	3.764612	5.558663	-0.481917
H	4.418513	7.127991	-0.765073
H	2.789673	4.097511	-3.864345
H	0.114181	4.704244	-6.215259
H	1.545704	3.722316	-5.799941
H	1.404090	5.401046	-5.196978
H	-1.085275	3.179098	-2.235119
H	-0.014823	2.511743	-0.086357
H	2.454811	2.635435	0.168520

Aryl Substituent: 3-OMe Intermediate 7 Conformer 0

H	0.972633	0.129123	-0.139019

C	2.063271	0.018041	-0.127561
N	2.644838	0.862178	-1.176739
C	2.772613	0.522097	-2.469248
S	3.315277	1.628575	-3.646667
N	2.480401	-0.762767	-2.805912
C	1.894959	-1.738297	-1.917609
O	0.494500	-1.691039	-2.029118
C	2.389258	-1.459596	-0.472883
H	3.468746	-1.611712	-0.430894
C	1.708721	-2.378629	0.534076
O	0.492496	-2.526073	0.577876
O	2.576959	-2.970681	1.339019
C	2.053353	-3.873266	2.370296
C	1.688783	-3.106212	3.628872
C	2.384735	-3.126130	-2.409443
F	2.080651	-3.297682	-3.710513
F	3.716712	-3.268417	-2.272995
F	1.790239	-4.113565	-1.710050
C	2.580245	0.432267	1.239142
C	3.961769	0.545576	1.465612
C	4.428384	0.904647	2.734659
O	5.744631	1.044882	3.058907
C	6.723141	0.807670	2.043964
C	3.513880	1.147362	3.775494
C	2.149391	1.034373	3.539346
C	1.672834	0.680031	2.270540
H	2.841017	1.827650	-0.947104
H	2.481315	-0.973044	-3.795923
H	0.118055	-2.060804	-1.203002
H	2.875479	-4.569234	2.541645
H	1.199249	-4.410332	1.954164
H	2.545011	-2.536501	4.001017
H	1.383570	-3.818123	4.402684
H	0.858892	-2.418504	3.446712
H	4.654652	0.360778	0.652781
H	7.691136	0.970094	2.519413
H	6.605683	1.505308	1.206724
H	6.667693	-0.221396	1.670617
H	3.896976	1.426756	4.752252
H	1.447738	1.229562	4.345167
H	0.605111	0.602037	2.087702

Aryl Substituent: 3-OMe Intermediate 7 Conformer 1

H	1.046654	0.150390	-0.010149
C	2.131629	-0.007315	-0.014229

N	2.737327	0.837133	-1.049161
C	2.835893	0.523902	-2.350748
S	3.419047	1.632412	-3.506878
N	2.478088	-0.736457	-2.715795
C	1.862834	-1.708078	-1.843532
O	0.464483	-1.596941	-1.932612
C	2.389467	-1.487889	-0.399843
H	3.461944	-1.686680	-0.378352
C	1.684470	-2.403412	0.593282
O	0.463612	-2.493656	0.657157
O	2.537997	-3.061296	1.362242
C	1.990551	-3.973157	2.372588
C	1.683094	-3.233670	3.662477
C	2.283601	-3.103159	-2.377054
F	1.955195	-3.227635	-3.677521
F	3.609219	-3.308460	-2.262730
F	1.653933	-4.080610	-1.694148
C	2.686365	0.350997	1.354094
C	1.788138	0.634146	2.392820
C	2.275271	0.945383	3.669733
O	1.486952	1.237999	4.742375
C	0.068369	1.226358	4.567590
C	3.660026	0.976683	3.899508
C	4.542331	0.700589	2.859246
C	4.065583	0.385568	1.582819
H	2.986326	1.784241	-0.795485
H	2.461763	-0.923073	-3.710337
H	0.082689	-1.971039	-1.111144
H	2.782316	-4.711302	2.506078
H	1.105572	-4.456019	1.954630
H	2.570933	-2.716701	4.037596
H	1.358574	-3.956406	4.418086
H	0.881437	-2.504478	3.518043
H	0.722858	0.615831	2.193257
H	-0.353943	1.485054	5.539224
H	-0.288575	0.233916	4.268271
H	-0.247865	1.965905	3.823031
H	4.019937	1.224526	4.893438
H	5.612365	0.731338	3.043820
H	4.762043	0.176460	0.776214

Aryl Substituent: 3-OMe Intermediate 7 Conformer 2

H	0.963644	0.242459	-0.173662
C	2.048318	0.076438	-0.134503
N	2.699136	0.841712	-1.199817

C	2.816859	0.436703	-2.474461
S	3.411771	1.463403	-3.699078
N	2.455292	-0.842630	-2.755908
C	1.898488	-1.813453	-1.829206
O	0.517391	-1.988210	-1.998667
C	2.324757	-1.426862	-0.385459
H	3.398259	-1.602633	-0.294988
C	1.584569	-2.258081	0.659634
O	0.409172	-2.099453	0.930149
O	2.396991	-3.147731	1.234078
C	1.828090	-4.010131	2.268200
C	1.954185	-3.361723	3.636454
C	2.489841	-3.192390	-2.224075
F	2.259397	-3.440247	-3.529496
F	3.819594	-3.228534	-2.027653
F	1.933252	-4.184127	-1.509260
C	2.548507	0.532664	1.227867
C	3.927230	0.673954	1.455895
C	4.384671	1.073553	2.716709
O	5.698172	1.244301	3.039142
C	6.682494	1.001087	2.031525
C	3.464080	1.327775	3.748211
C	2.102096	1.185074	3.511125
C	1.634507	0.790878	2.251288
H	2.932267	1.808040	-1.012558
H	2.470737	-1.100844	-3.734856
H	0.042999	-1.215535	-1.657291
H	2.414109	-4.928056	2.198943
H	0.788412	-4.220505	2.011016
H	2.999627	-3.135451	3.865918
H	1.574695	-4.050556	4.398271
H	1.373386	-2.436560	3.688099
H	4.625543	0.482627	0.649344
H	7.646050	1.201005	2.501715
H	6.549000	1.670425	1.173851
H	6.652619	-0.039802	1.688942
H	3.839351	1.638459	4.718537
H	1.394881	1.389034	4.309987
H	0.569661	0.685025	2.069338

Aryl Substituent: 3-OMe Intermediate 7 Conformer 3

H	1.110547	0.241987	-0.041045
C	2.188808	0.043281	-0.037538
N	2.830581	0.848003	-1.082463
C	2.919825	0.511545	-2.378967

S	3.549868	1.578361	-3.549655
N	2.512284	-0.738593	-2.726256
C	1.860188	-1.673683	-1.840886
O	0.467043	-1.511950	-1.934662
C	2.392210	-1.451634	-0.399572
H	3.456565	-1.689516	-0.372360
C	1.652052	-2.324902	0.605963
O	0.428612	-2.372101	0.666214
O	2.479386	-2.998324	1.390025
C	1.896680	-3.871775	2.414478
C	1.602805	-3.097169	3.686814
C	2.229436	-3.091328	-2.352855
F	1.899094	-3.222568	-3.652281
F	3.546328	-3.344458	-2.232561
F	1.562524	-4.034295	-1.657105
C	2.750697	0.401894	1.327671
C	1.859348	0.742173	2.354960
C	2.351662	1.055073	3.629495
O	1.570041	1.400369	4.691389
C	0.153195	1.443146	4.507875
C	3.735027	1.031075	3.868049
C	4.610924	0.698670	2.838800
C	4.128794	0.381273	1.565011
H	3.117129	1.787799	-0.841774
H	2.493706	-0.940752	-3.717735
H	0.070369	-1.860753	-1.109058
H	2.664276	-4.630989	2.569122
H	1.000252	-4.335734	1.999455
H	2.502679	-2.597495	4.056625
H	1.254016	-3.794788	4.455116
H	0.822609	-2.349254	3.522398
H	0.795642	0.766798	2.147884
H	-0.263829	1.735014	5.472352
H	-0.241968	0.461022	4.222832
H	-0.128811	2.182049	3.749074
H	4.099332	1.281030	4.859851
H	5.680143	0.686854	3.030154
H	4.820433	0.127545	0.767068

Aryl Substituent: 3-OMe Intermediate 7 Conformer 4

H	1.056977	0.083390	-0.008300
C	2.142141	-0.068108	-0.044943
N	2.719906	0.827861	-1.052206
C	2.790566	0.576926	-2.369014
S	3.346019	1.740793	-3.483762

N	2.426778	-0.665576	-2.785670
C	1.834816	-1.679918	-1.946571
O	0.434381	-1.568475	-1.995580
C	2.396400	-1.527239	-0.507394
H	3.469746	-1.721502	-0.521943
C	1.719571	-2.492222	0.458001
O	0.501065	-2.592496	0.546588
O	2.594540	-3.179569	1.175516
C	2.076071	-4.137196	2.158344
C	1.796007	-3.455439	3.485735
C	2.244449	-3.046458	-2.556756
F	1.884649	-3.108661	-3.853279
F	3.572804	-3.253533	-2.484511
F	1.633021	-4.057431	-1.906885
C	2.727175	0.226777	1.325829
C	1.852098	0.460953	2.396095
C	2.367286	0.713034	3.675063
O	1.602910	0.955203	4.777230
C	0.180800	0.950922	4.633469
C	3.756768	0.734578	3.875495
C	4.615878	0.507498	2.804409
C	4.111110	0.251585	1.525560
H	2.974773	1.761894	-0.759173
H	2.391011	-0.805603	-3.787354
H	0.073789	-1.984123	-1.184570
H	2.874678	-4.875357	2.241417
H	1.183955	-4.607376	1.741120
H	2.689867	-2.948872	3.860773
H	1.494443	-4.212031	4.217326
H	0.986908	-2.726157	3.392476
H	0.782655	0.451547	2.219466
H	-0.219864	1.163451	5.625262
H	-0.183170	-0.026559	4.295864
H	-0.151437	1.724438	3.931615
H	4.138548	0.936532	4.871651
H	5.689720	0.530340	2.966664
H	4.789690	0.080179	0.695144

Aryl Substituent: 4-NO2 Intermediate 12 Conformer 0

H	1.236101	-0.515797	-0.357258
C	2.305261	-0.335888	-0.213970
C	2.494126	1.142486	0.249750
O	3.827300	1.384056	0.655505
C	1.977549	2.147119	-0.780294
C	0.667372	2.044880	-1.278139

C	0.176447	2.973026	-2.189393
C	1.011843	4.014520	-2.598925
C	2.312236	4.149285	-2.114327
C	2.786380	3.210400	-1.201661
N	0.505371	4.994488	-3.558138
O	-0.646120	4.857699	-3.980939
O	1.251424	5.915086	-3.902995
C	2.771688	-1.290167	0.895925
O	3.748545	-2.008712	0.809784
O	1.962894	-1.205367	1.953007
C	2.284743	-2.033208	3.115550
C	1.676636	-3.418579	2.979757
C	3.050385	-0.677778	-1.488491
N	4.143620	-0.101877	-1.772786
C	4.954878	-0.267327	-2.898994
N	6.009687	-1.051290	-2.695123
S	4.659702	0.617897	-4.300492
C	2.367133	-1.729926	-2.370746
F	1.824260	-2.697160	-1.604935
F	1.368957	-1.142804	-3.061228
F	3.202438	-2.309910	-3.242133
H	1.884453	1.251375	1.153309
H	4.401125	1.146428	-0.094115
H	0.012986	1.243729	-0.948313
H	-0.832095	2.899832	-2.576324
H	2.933870	4.971687	-2.445553
H	3.789647	3.308958	-0.804652
H	1.857346	-1.483078	3.955297
H	3.370594	-2.073060	3.220272
H	0.593759	-3.357208	2.836257
H	1.871328	-3.987634	3.894724
H	2.117046	-3.960800	2.138398
H	6.146755	-1.536138	-1.816780
H	6.708331	-1.151244	-3.418831

Aryl Substituent: 4-NO2 Intermediate 12 Conformer 1

H	1.183404	-0.362144	-0.186418
C	2.263167	-0.214395	-0.093630
C	2.522940	1.278395	0.280749
O	3.877103	1.487633	0.631931
C	2.013298	2.246324	-0.787209
C	0.692284	2.156932	-1.258031
C	0.209611	3.055285	-2.202997
C	1.064538	4.053702	-2.674510
C	2.376733	4.174941	-2.218809

C	2.842244	3.266585	-1.271575
N	0.566928	5.001644	-3.669895
O	-0.595582	4.877585	-4.065580
O	1.331048	5.883932	-4.070802
C	2.739095	-1.125083	1.048139
O	3.678316	-1.892087	0.960216
O	1.986057	-0.943952	2.133847
C	2.327144	-1.719042	3.327019
C	1.652901	-3.079937	3.300961
C	2.946557	-0.649329	-1.374500
N	4.048047	-0.129341	-1.726876
C	4.809182	-0.381271	-2.871961
N	5.844135	-1.190655	-2.665836
S	4.489353	0.440212	-4.306420
C	2.194155	-1.716725	-2.178471
F	1.633572	-2.617294	-1.347068
F	1.201867	-1.124934	-2.873564
F	2.979457	-2.377963	-3.038465
H	1.948222	1.460705	1.195300
H	4.416205	1.193597	-0.123446
H	0.023001	1.389528	-0.881729
H	-0.807226	2.991424	-2.569289
H	3.013918	4.963753	-2.598640
H	3.855055	3.355116	-0.897001
H	1.965650	-1.102832	4.151694
H	3.413991	-1.803544	3.383866
H	0.567804	-2.976649	3.206829
H	1.869108	-3.605642	4.236811
H	2.025906	-3.687994	2.472115
H	6.000167	-1.631340	-1.767602
H	6.511245	-1.352039	-3.407950

Aryl Substituent: 4-NO2 Intermediate 12 Conformer 2

H	1.144564	-0.323909	-0.227483
C	2.226930	-0.213802	-0.114732
C	2.529340	1.264681	0.284328
O	3.885018	1.425869	0.654174
C	2.062389	2.262096	-0.775835
C	0.736297	2.236359	-1.240300
C	0.291528	3.160221	-2.179182
C	1.189922	4.119829	-2.650699
C	2.508322	4.178216	-2.200669
C	2.935534	3.245104	-1.259372
N	0.732286	5.094457	-3.639373
O	-0.436489	5.026237	-4.030197

O	1.534629	5.942419	-4.039775
C	2.652854	-1.155461	1.021614
O	3.563290	-1.956709	0.936173
O	1.892770	-0.958724	2.099620
C	2.187474	-1.760859	3.287269
C	1.461407	-3.094036	3.235349
C	2.915638	-0.654754	-1.390706
N	4.040392	-0.169300	-1.718196
C	4.811465	-0.432782	-2.854054
N	5.812263	-1.282709	-2.642763
S	4.546846	0.421142	-4.280749
C	2.139692	-1.684779	-2.220815
F	1.536477	-2.577140	-1.411016
F	1.178603	-1.049713	-2.921726
F	2.914787	-2.360122	-3.079309
H	1.949302	1.452302	1.194348
H	4.423942	1.119490	-0.096417
H	0.033840	1.499640	-0.862622
H	-0.728976	3.145682	-2.540535
H	3.179604	4.938397	-2.580160
H	3.952885	3.285581	-0.888841
H	1.838899	-1.141073	4.114813
H	3.269404	-1.888035	3.357148
H	0.382596	-2.947251	3.127746
H	1.643783	-3.638668	4.167571
H	1.822012	-3.706449	2.404234
H	5.934212	-1.744594	-1.749937
H	6.485567	-1.458353	-3.376026

Aryl Substituent: 4-NO2 Intermediate 12 Conformer 3

H	1.187987	-0.131112	-0.026415
C	2.277276	-0.043137	0.017355
C	2.636727	1.449147	0.299596
O	4.013387	1.595926	0.589889
C	2.142184	2.388682	-0.800434
C	0.800814	2.354385	-1.217938
C	0.334590	3.230067	-2.192042
C	1.226904	4.149792	-2.747005
C	2.560727	4.215437	-2.346042
C	3.009435	3.330850	-1.368722
N	0.746513	5.074009	-3.772650
O	-0.435741	4.999637	-4.119314
O	1.544040	5.887947	-4.246523
C	2.746586	-0.918338	1.189261
O	3.644058	-1.734875	1.112614

O	2.040016	-0.644755	2.286805
C	2.382427	-1.375277	3.507377
C	1.649173	-2.704209	3.568490
C	2.880693	-0.585495	-1.262503
N	3.993995	-0.151363	-1.686480
C	4.690862	-0.510424	-2.843836
N	5.683376	-1.370066	-2.632243
S	4.361241	0.251445	-4.308595
C	2.035133	-1.650073	-1.972246
F	1.470587	-2.474541	-1.067724
F	1.041418	-1.041297	-2.650697
F	2.740237	-2.396571	-2.831840
H	2.109661	1.713230	1.222917
H	4.505207	1.231471	-0.167006
H	0.103238	1.648958	-0.777069
H	-0.697755	3.208488	-2.517668
H	3.227249	4.943895	-2.790612
H	4.039524	3.377433	-1.036042
H	2.072695	-0.703881	4.309711
H	3.465839	-1.505436	3.538330
H	0.567440	-2.556926	3.496743
H	1.867919	-3.191333	4.524412
H	1.972213	-3.369305	2.762825
H	5.850295	-1.769852	-1.716990
H	6.310030	-1.610135	-3.388288

Aryl Substituent: 4-NO2 Intermediate 12 Conformer 4

H	1.190311	-0.396364	-0.161795
C	2.271347	-0.251753	-0.079825
C	2.539643	1.237734	0.302132
O	3.896584	1.437868	0.647489
C	2.029059	2.215554	-0.756268
C	0.706948	2.132681	-1.225226
C	0.223547	3.041464	-2.159792
C	1.078753	4.043650	-2.622776
C	2.392162	4.158210	-2.168966
C	2.858405	3.239508	-1.232190
N	0.580041	5.002960	-3.606629
O	-0.583739	4.885029	-4.000482
O	1.344392	5.888479	-3.999915
C	2.759604	-1.166421	1.053406
O	3.695358	-1.935968	0.951765
O	2.017038	-0.989462	2.147082
C	2.389425	-1.744836	3.342981
C	3.453800	-1.008500	4.138164

C	2.940801	-0.679585	-1.370287
N	4.042645	-0.163329	-1.727207
C	4.794058	-0.410893	-2.879751
N	5.822853	-1.231862	-2.688815
S	4.471971	0.427789	-4.303720
C	2.175356	-1.736405	-2.175699
F	1.623862	-2.644190	-1.346126
F	1.175210	-1.135221	-2.851246
F	2.947320	-2.390458	-3.053195
H	1.970807	1.416239	1.221035
H	4.430172	1.147024	-0.112981
H	0.037517	1.362187	-0.855693
H	-0.794190	2.982788	-2.524434
H	3.029605	4.950083	-2.541932
H	3.871972	3.322896	-0.858522
H	2.723736	-2.736712	3.033205
H	1.454868	-1.832614	3.899191
H	4.381440	-0.920425	3.565747
H	3.667560	-1.566132	5.055889
H	3.111027	-0.007158	4.415205
H	5.982235	-1.681020	-1.795419
H	6.484895	-1.389833	-3.436198

Aryl Substituent: 4-NO2 Intermediate 13 Conformer 0

C	4.625465	-1.344838	2.062808
C	5.525721	-0.562285	1.122066
O	5.537673	-1.157153	-0.211253
C	4.596928	-0.760912	-1.082839
O	3.690031	0.006841	-0.823195
C	4.852302	-1.393785	-2.418325
C	3.895535	-1.720072	-3.323405
C	2.432306	-1.658806	-3.246696
C	1.737008	-1.472633	-4.459588
C	0.348623	-1.416784	-4.490422
C	-0.347280	-1.583611	-3.293392
C	0.305393	-1.807161	-2.078965
C	1.693506	-1.840860	-2.060331
N	-1.811187	-1.537368	-3.312445
O	-2.373462	-1.346015	-4.393240
O	-2.412362	-1.689990	-2.246489
C	6.265259	-1.660979	-2.787787
N	6.560995	-2.726457	-3.425667
C	7.785269	-3.107996	-3.956101
N	8.662990	-3.617411	-3.093428
S	8.012983	-3.042278	-5.628584

C	7.333728	-0.599915	-2.452823
F	6.781672	0.567738	-2.075426
F	8.143593	-1.022535	-1.461705
F	8.103760	-0.354605	-3.528796
H	4.700958	-0.919046	3.068732
H	4.928889	-2.394918	2.109733
H	3.580841	-1.289662	1.744401
H	6.570549	-0.602554	1.434441
H	5.212300	0.479828	1.032149
H	4.270256	-2.078471	-4.280119
H	2.294997	-1.361171	-5.384112
H	-0.187574	-1.258631	-5.417444
H	-0.268989	-1.954565	-1.173233
H	2.198691	-2.030615	-1.122450
H	8.472836	-3.655092	-2.100073
H	9.516798	-4.037237	-3.435996

Aryl Substituent: 4-NO2 Intermediate 13 Conformer 1

C	4.669848	-1.373997	2.043839
C	5.549578	-0.563272	1.107521
O	5.557055	-1.136796	-0.235158
C	4.602910	-0.739664	-1.091706
O	3.688792	0.012163	-0.811526
C	4.853446	-1.348572	-2.439045
C	3.893265	-1.668128	-3.342442
C	2.429781	-1.623980	-3.257212
C	1.726205	-1.435419	-4.465037
C	0.337151	-1.396653	-4.488930
C	-0.350907	-1.582780	-3.290210
C	0.310374	-1.808661	-2.080921
C	1.698913	-1.825274	-2.068996
N	-1.815304	-1.554760	-3.302037
O	-2.385066	-1.360307	-4.378357
O	-2.409485	-1.725015	-2.234834
C	6.266194	-1.597180	-2.822999
N	6.567256	-2.651335	-3.476692
C	7.791037	-3.013973	-4.021437
N	8.680330	-3.525664	-3.172102
S	8.005256	-2.924038	-5.694646
C	7.326143	-0.528829	-2.483373
F	6.764591	0.628015	-2.087625
F	8.148380	-0.954506	-1.503718
F	8.084959	-0.262654	-3.562288
H	4.750521	-0.965174	3.056366
H	4.987420	-2.420652	2.068706

H	3.621218	-1.327229	1.737431
H	6.597860	-0.592996	1.409298
H	5.219955	0.475321	1.037072
H	4.265025	-2.007708	-4.307139
H	2.278201	-1.309017	-5.391213
H	-0.205294	-1.237031	-5.412051
H	-0.257560	-1.970988	-1.173683
H	2.210701	-2.015924	-1.134998
H	8.496854	-3.580880	-2.178291
H	9.535000	-3.933641	-3.526670

Aryl Substituent: 4-NO2 Intermediate 13 Conformer 2

C	4.891327	-1.417091	2.051909
C	5.731045	-0.584775	1.098008
O	5.706366	-1.145307	-0.249863
C	4.722396	-0.753283	-1.074303
O	3.807029	-0.016365	-0.760630
C	4.941102	-1.346982	-2.433931
C	3.958445	-1.672959	-3.311019
C	2.497787	-1.649930	-3.180658
C	1.754627	-1.458974	-4.364189
C	0.365083	-1.439086	-4.344605
C	-0.282911	-1.646748	-3.127209
C	0.418581	-1.875607	-1.941393
C	1.806930	-1.873145	-1.972626
N	-1.747193	-1.638799	-3.093408
O	-2.352754	-1.441852	-4.149556
O	-2.305539	-1.827473	-2.010072
C	6.344847	-1.574601	-2.861412
N	6.639253	-2.618844	-3.533696
C	7.850979	-2.963380	-4.115462
N	8.767137	-3.476259	-3.295878
S	8.019382	-2.852590	-5.792717
C	7.401613	-0.496925	-2.541560
F	6.837760	0.650302	-2.121921
F	8.254422	-0.920763	-1.587518
F	8.128616	-0.213860	-3.637891
H	4.996966	-1.017165	3.065692
H	5.224106	-2.459305	2.056387
H	3.833329	-1.382348	1.777782
H	6.788204	-0.602409	1.368115
H	5.385098	0.449677	1.047817
H	4.305325	-1.999682	-4.289302
H	2.275796	-1.315394	-5.305600
H	-0.207900	-1.277501	-5.248733

H	-0.118459	-2.054822	-1.018657
H	2.349791	-2.065375	-1.056694
H	8.609022	-3.547215	-2.298679
H	9.615189	-3.873265	-3.677729

Aryl Substituent: 4-NO2 Intermediate 13 Conformer 3

C	4.427611	-1.478356	2.111936
C	5.404737	-0.691264	1.255584
O	5.475021	-1.236141	-0.097252
C	4.603672	-0.773564	-1.005467
O	3.708969	0.016371	-0.770883
C	4.913401	-1.362831	-2.350801
C	3.982830	-1.648901	-3.295491
C	2.517907	-1.591671	-3.249250
C	1.847127	-1.339682	-4.463797
C	0.459426	-1.284433	-4.519239
C	-0.259866	-1.518216	-3.347458
C	0.368646	-1.807391	-2.134231
C	1.756256	-1.840076	-2.089865
N	-1.723237	-1.473658	-3.393517
O	-2.264149	-1.222521	-4.472955
O	-2.345132	-1.686628	-2.350112
C	6.338870	-1.617322	-2.686592
N	6.646746	-2.619275	-3.412873
C	7.892876	-3.025159	-3.866755
N	8.143304	-2.745960	-5.144170
S	8.895600	-3.969499	-2.885454
C	7.402110	-0.606145	-2.208157
F	6.837701	0.571155	-1.871531
F	8.088846	-1.059146	-1.147230
F	8.287047	-0.361221	-3.194399
H	4.459428	-1.095550	3.137274
H	4.693621	-2.539376	2.131999
H	3.405022	-1.374471	1.738675
H	6.428070	-0.777650	1.624538
H	5.129280	0.363229	1.188845
H	4.380484	-1.968358	-4.256545
H	2.423289	-1.176292	-5.369201
H	-0.058646	-1.075967	-5.446619
H	-0.223257	-2.005623	-1.249748
H	2.243169	-2.082366	-1.154151
H	7.505559	-2.190676	-5.700290
H	8.984861	-3.098668	-5.578616

Aryl Substituent: 4-NO2 Intermediate 13 Conformer 4

C	6.200450	0.872665	1.085678

C	5.436841	-0.441040	1.053225
O	5.481069	-1.036431	-0.279814
C	4.541977	-0.665653	-1.164495
O	3.617697	0.086828	-0.922962
C	4.813287	-1.318686	-2.487976
C	3.862166	-1.697174	-3.378001
C	2.398349	-1.681175	-3.287191
C	1.683722	-1.533276	-4.493903
C	0.293980	-1.521222	-4.508291
C	-0.382202	-1.693042	-3.300630
C	0.291354	-1.879865	-2.091382
C	1.680020	-1.870474	-2.089431
N	-1.846813	-1.691833	-3.302438
O	-2.427515	-1.534192	-4.378977
O	-2.430593	-1.846502	-2.227099
C	6.231979	-1.547802	-2.861751
N	6.559576	-2.618413	-3.474500
C	7.791886	-2.971567	-4.005685
N	8.692454	-3.430309	-3.138284
S	8.004875	-2.937162	-5.681201
C	7.263617	-0.440495	-2.563340
F	6.668381	0.717544	-2.221783
F	8.088172	-0.800655	-1.558891
F	8.024495	-0.201686	-3.646388
H	6.222348	1.249091	2.113496
H	5.719340	1.624522	0.454180
H	7.229766	0.730969	0.745245
H	4.391414	-0.314031	1.340050
H	5.900600	-1.193995	1.692481
H	4.240265	-2.065653	-4.329524
H	2.226995	-1.416838	-5.426544
H	-0.258037	-1.392419	-5.430555
H	-0.267404	-2.032678	-1.176810
H	2.202826	-2.032172	-1.155726
H	8.508321	-3.456439	-2.143387
H	9.556340	-3.831340	-3.478197

Aryl Substituent: 4-NO2 Intermediate 14 Conformer 1

C	2.046345	0.462604	1.749533
C	2.590650	-0.076002	0.437945
O	3.101473	1.011996	-0.397739
C	4.363915	1.390600	-0.208383
O	5.149156	0.885200	0.571183
C	4.714719	2.574035	-1.117735
C	3.882107	3.828225	-0.704657

O	4.044062	4.100129	0.673553
C	4.169402	5.030341	-1.603720
C	4.015607	4.924348	-2.996430
C	4.255194	6.015292	-3.824550
C	4.650665	7.221674	-3.243024
C	4.800630	7.361003	-1.863865
C	4.554319	6.258094	-1.049768
N	4.909259	8.373743	-4.106084
O	4.771179	8.233514	-5.324267
O	5.254518	9.433975	-3.577707
C	6.209149	2.828498	-1.004785
O	6.716528	3.585940	-0.207105
C	7.102091	2.080004	-2.021810
F	6.791647	0.770184	-2.049633
F	6.869612	2.589290	-3.250456
F	8.397584	2.213865	-1.737508
H	1.616792	-0.363338	2.325853
H	1.261180	1.203313	1.571406
H	2.839524	0.921947	2.346027
H	1.807608	-0.528669	-0.172130
H	3.395363	-0.796759	0.593294
H	4.461907	2.288087	-2.143209
H	2.830321	3.552756	-0.834662
H	4.995891	4.209615	0.838044
H	3.695061	3.988919	-3.445215
H	4.138278	5.941165	-4.898359
H	5.101505	8.311888	-1.442228
H	4.651757	6.354724	0.025093

Aryl Substituent: 4-NO2 Intermediate 14 Conformer 2

C	1.714636	0.465980	1.468118
C	2.367303	-0.065752	0.204015
O	2.948003	1.025880	-0.580025
C	4.187377	1.406892	-0.277383
O	4.900644	0.902079	0.568933
C	4.615696	2.594088	-1.147903
C	3.748558	3.846149	-0.805420
O	3.793404	4.118249	0.581450
C	4.107346	5.048631	-1.677849
C	4.061510	4.943314	-3.078296
C	4.366277	6.034007	-3.885020
C	4.717418	7.239594	-3.274086
C	4.760956	7.378092	-1.887377
C	4.450875	6.275332	-1.095156
N	5.042106	8.392051	-4.113893

O	5.002443	8.251476	-5.339215
O	5.341459	9.453127	-3.559780
C	6.094271	2.847787	-0.902180
O	6.529152	3.609742	-0.066894
C	7.075159	2.091241	-1.828900
F	6.761318	0.783184	-1.883833
F	6.964338	2.598966	-3.074773
F	8.338554	2.218488	-1.422329
H	1.238957	-0.362728	2.002702
H	0.946920	1.207865	1.229193
H	2.455422	0.921796	2.130996
H	1.638671	-0.514088	-0.472972
H	3.155486	-0.788321	0.423054
H	4.455323	2.313211	-2.193247
H	2.712024	3.568731	-1.023747
H	4.727916	4.228149	0.825583
H	3.774738	4.008578	-3.550779
H	4.331957	5.960399	-4.964680
H	5.030075	8.328418	-1.443598
H	4.465208	6.371592	-0.015908

Aryl Substituent: 4-NO2 Intermediate 14 Conformer 3

C	2.140611	0.502358	1.875334
C	2.533498	-0.006300	0.499324
O	3.031291	1.088227	-0.335859
C	4.325446	1.388577	-0.246540
O	5.145471	0.807439	0.438601
C	4.662860	2.591720	-1.135132
C	3.955930	3.871164	-0.587542
O	4.266263	4.064829	0.778430
C	4.230174	5.098148	-1.456821
C	3.955858	5.066220	-2.834642
C	4.180508	6.183177	-3.631746
C	4.683231	7.340727	-3.034121
C	4.954644	7.406681	-1.668130
C	4.721855	6.278756	-0.884987
N	4.925987	8.519707	-3.864629
O	4.681486	8.444169	-5.071811
O	5.364591	9.537042	-3.321335
C	6.174339	2.750559	-1.160680
O	6.803365	3.441914	-0.390239
C	6.916094	1.990961	-2.285681
F	6.526107	0.702774	-2.322796
F	6.595618	2.557146	-3.468455
F	8.239122	2.040289	-2.128159

H	1.715125	-0.323101	2.455338
H	1.388748	1.293396	1.799033
H	3.009711	0.889110	2.414811
H	1.674265	-0.387558	-0.054378
H	3.305156	-0.776352	0.552017
H	4.293681	2.373096	-2.141514
H	2.880985	3.665405	-0.624281
H	5.234296	4.114519	0.852279
H	3.552557	4.169786	-3.295832
H	3.971143	6.165558	-4.693875
H	5.336987	8.321456	-1.232810
H	4.913798	6.318703	0.180728

Aryl Substituent: 4-NO2 Intermediate 14 Conformer 4

C	2.241524	0.548056	1.991645
C	2.465036	0.082929	0.563248
O	2.964940	1.179103	-0.268496
C	4.279798	1.388673	-0.281201
O	5.111912	0.724002	0.306563
C	4.624482	2.604905	-1.148818
C	4.051464	3.901882	-0.496249
O	4.480288	4.013522	0.846789
C	4.340244	5.146265	-1.335347
C	3.958401	5.193704	-2.686821
C	4.194889	6.328727	-3.454491
C	4.818674	7.424010	-2.853640
C	5.199149	7.411133	-1.512313
C	4.953063	6.266256	-0.758508
N	5.073668	8.621757	-3.653039
O	4.733759	8.615515	-4.839286
O	5.617522	9.584713	-3.105827
C	6.136153	2.668142	-1.293597
O	6.869797	3.282670	-0.551031
C	6.732714	1.913972	-2.505074
F	6.251784	0.657656	-2.562171
F	6.361123	2.553681	-3.634193
F	8.064209	1.867943	-2.453687
H	1.807351	-0.272254	2.572504
H	1.550560	1.395671	2.022463
H	3.184233	0.840888	2.462178
H	1.531507	-0.207202	0.078926
H	3.177091	-0.742446	0.507780
H	4.161874	2.453039	-2.128686
H	2.965360	3.768647	-0.453860
H	5.452267	3.997645	0.843635

H	3.460836	4.346573	-3.149215
H	3.902697	6.372306	-4.496110
H	5.674191	8.279457	-1.073114
H	5.229243	6.245861	0.289140

Aryl Substituent: 4-NO2 Intermediate 14 Conformer 5

C	2.810000	-1.428557	-0.331936
C	2.485388	-0.100426	0.329352
O	3.010093	1.016938	-0.458478
C	4.270028	1.385706	-0.236186
O	5.032540	0.869087	0.558402
C	4.648564	2.579622	-1.120756
C	3.820300	3.837532	-0.710966
O	3.965265	4.101669	0.670857
C	4.129492	5.041234	-1.600573
C	3.970578	4.947109	-2.993571
C	4.231467	6.037784	-3.815532
C	4.653536	7.231925	-3.227293
C	4.808989	7.359462	-1.847591
C	4.541059	6.257008	-1.039685
N	4.934741	8.383633	-4.083494
O	4.790960	8.254254	-5.302265
O	5.303653	9.432934	-3.549265
C	6.143143	2.816411	-0.976193
O	6.642634	3.566560	-0.166699
C	7.049047	2.058807	-1.975262
F	6.720984	0.753806	-2.016660
F	6.853571	2.576675	-3.206638
F	8.339181	2.173082	-1.659255
H	2.342836	-2.237293	0.239518
H	3.889193	-1.603490	-0.354692
H	2.422094	-1.460720	-1.354384
H	2.898294	-0.033895	1.337597
H	1.410902	0.085697	0.361649
H	4.413444	2.309851	-2.154847
H	2.768061	3.572063	-0.857211
H	4.915880	4.197676	0.850051
H	3.629432	4.021388	-3.447437
H	4.110550	5.972567	-4.889478
H	5.130506	8.301395	-1.421111
H	4.641995	6.345104	0.035635

Aryl Substituent: 4-NO2 Intermediate 15 Conformer 0

C	2.537993	1.466198	0.579610
C	3.248323	0.131108	0.490981
O	3.696402	-0.033277	-0.890006

C	4.361192	-1.154739	-1.172405
O	4.631223	-2.027319	-0.367053
C	4.749035	-1.223497	-2.624420
C	3.857626	-1.275709	-3.646158
C	2.397072	-1.312979	-3.605696
C	1.702066	-0.837456	-4.737140
C	0.312967	-0.831170	-4.772405
C	-0.380509	-1.336238	-3.672510
C	0.272745	-1.848922	-2.550025
C	1.661127	-1.833947	-2.520922
N	-1.846286	-1.346210	-3.702894
O	-2.409388	-0.900195	-4.704733
O	-2.444583	-1.798997	-2.724832
C	6.187878	-1.253844	-2.996055
O	6.594643	-1.395658	-4.134604
C	7.252329	-1.082857	-1.877452
F	6.886426	-0.129167	-0.993127
F	7.420076	-2.236935	-1.206743
F	8.429006	-0.729434	-2.405406
H	2.190253	1.619968	1.605744
H	1.670301	1.494850	-0.086210
H	3.211473	2.287352	0.316841
H	2.589329	-0.706000	0.739101
H	4.125954	0.083922	1.141411
H	4.293793	-1.287155	-4.643527
H	2.260004	-0.458808	-5.587662
H	-0.225939	-0.454361	-5.632316
H	-0.299297	-2.256695	-1.726441
H	2.167765	-2.271828	-1.668944

Aryl Substituent: 4-NO2 Intermediate 15 Conformer 1

C	4.075848	0.946521	1.288773
C	3.020989	0.233242	0.459693
O	3.490777	0.033199	-0.911268
C	4.200588	-1.069597	-1.161257
O	4.480806	-1.928779	-0.345062
C	4.631480	-1.136249	-2.601577
C	3.774654	-1.246734	-3.647754
C	2.317412	-1.359770	-3.647111
C	1.630066	-0.933785	-4.802631
C	0.243907	-0.999712	-4.874730
C	-0.452328	-1.527434	-3.787248
C	0.195659	-1.993298	-2.641567
C	1.580066	-1.906053	-2.575859
N	-1.914544	-1.611790	-3.855831

O	-2.472220	-1.208643	-4.878617
O	-2.515412	-2.080589	-2.886885
C	6.080503	-1.094650	-2.930233
O	6.527608	-1.215106	-4.056026
C	7.101862	-0.875119	-1.779831
F	6.669730	0.068514	-0.913373
F	7.294763	-2.017307	-1.096194
F	8.279074	-0.476629	-2.272669
H	3.678246	1.135029	2.291298
H	4.342863	1.906476	0.837124
H	4.978501	0.337250	1.384501
H	2.119321	0.836501	0.342997
H	2.758186	-0.738014	0.883248
H	4.239517	-1.247011	-4.632126
H	2.190652	-0.536504	-5.642880
H	-0.290307	-0.660527	-5.753061
H	-0.376859	-2.420517	-1.828223
H	2.085790	-2.306715	-1.705322

Aryl Substituent: 4-NO2 Intermediate 15 Conformer 2

C	2.458519	1.264835	0.707550
C	3.167092	-0.065232	0.553708
O	3.642770	-0.148334	-0.825511
C	4.321345	-1.247668	-1.157757
O	4.582927	-2.162551	-0.397901
C	4.736879	-1.230957	-2.603715
C	3.864412	-1.236487	-3.642985
C	2.403968	-1.294277	-3.630454
C	1.721307	-0.763218	-4.744521
C	0.333050	-0.773688	-4.803714
C	-0.371341	-1.351321	-3.747277
C	0.270309	-1.919271	-2.644918
C	1.657647	-1.887054	-2.590510
N	-1.836172	-1.379831	-3.804524
O	-2.388622	-0.884721	-4.789019
O	-2.444227	-1.896636	-2.864932
C	6.182317	-1.224137	-2.949719
O	6.610922	-1.289625	-4.087233
C	7.224650	-1.116481	-1.802768
F	6.837483	-0.217980	-0.870879
F	7.384763	-2.307575	-1.198249
F	8.409524	-0.728492	-2.286144
H	2.090272	1.358725	1.733754
H	1.604709	1.336460	0.027181
H	3.139406	2.097572	0.507895

H	2.500836	-0.913185	0.737214
H	4.031141	-0.154978	1.217628
H	4.317734	-1.187007	-4.631498
H	2.287894	-0.328501	-5.561905
H	-0.197205	-0.355240	-5.649620
H	-0.309580	-2.381932	-1.856561
H	2.156673	-2.366716	-1.756715

Aryl Substituent: 4-NO2 Intermediate 15 Conformer 3

C	2.053431	-1.057113	1.041999
C	3.386341	-0.370321	0.799927
O	3.730007	-0.340573	-0.623333
C	4.378428	-1.395786	-1.121479
O	4.668843	-2.402218	-0.501035
C	4.746970	-1.184743	-2.567199
C	3.852902	-1.107894	-3.584320
C	2.398864	-1.251979	-3.558148
C	1.660163	-0.618150	-4.578889
C	0.274063	-0.706708	-4.617356
C	-0.369810	-1.464052	-3.638478
C	0.330159	-2.135212	-2.634144
C	1.714426	-2.025202	-2.598009
N	-1.831445	-1.573934	-3.675839
O	-2.434834	-0.989577	-4.578056
O	-2.386127	-2.244131	-2.802424
C	6.181325	-1.051217	-2.937986
O	6.579365	-0.915997	-4.080324
C	7.251816	-1.086621	-1.812850
F	6.869544	-0.349288	-0.747156
F	7.454925	-2.350656	-1.398923
F	8.413666	-0.598943	-2.259513
H	1.796215	-0.967515	2.102593
H	2.102301	-2.120468	0.792420
H	1.258164	-0.588156	0.455487
H	4.199838	-0.861245	1.337754
H	3.351130	0.684471	1.076752
H	4.281185	-0.906747	-4.564746
H	2.180381	-0.043113	-5.338370
H	-0.299981	-0.211712	-5.390229
H	-0.202688	-2.735223	-1.907501
H	2.263832	-2.580877	-1.847426

Aryl Substituent: 4-NO2 Intermediate 15 Conformer 4

C	4.067893	0.978701	1.274970
C	3.012990	0.262534	0.448453
O	3.488352	0.044518	-0.917966

C	4.194969	-1.063872	-1.151885
O	4.467387	-1.915069	-0.324781
C	4.632454	-1.148318	-2.589360
C	3.780107	-1.267718	-3.638234
C	2.322527	-1.375548	-3.643960
C	1.643322	-0.961493	-4.808647
C	0.257396	-1.023064	-4.887874
C	-0.447000	-1.534374	-3.797798
C	0.192672	-1.988149	-2.642648
C	1.577047	-1.905402	-2.570079
N	-1.909130	-1.613937	-3.873646
O	-2.459440	-1.221283	-4.904476
O	-2.517274	-2.068441	-2.902442
C	6.083055	-1.115685	-2.911621
O	6.535365	-1.253893	-4.033290
C	7.099498	-0.881718	-1.759713
F	6.665209	0.074376	-0.908132
F	7.287495	-2.014676	-1.059543
F	8.279524	-0.491886	-2.252803
H	3.666353	1.180647	2.273300
H	4.342843	1.931962	0.814040
H	4.966391	0.365238	1.382128
H	2.115720	0.870019	0.320330
H	2.741920	-0.702326	0.881298
H	4.249700	-1.280736	-4.620274
H	2.210182	-0.576795	-5.650519
H	-0.270565	-0.692841	-5.773364
H	-0.386225	-2.402736	-1.827272
H	2.076263	-2.296736	-1.691591

Aryl Substituent: 4-NO2 Intermediate 16 Conformer 1

C	8.864254	2.898892	0.857469
C	8.115614	1.756334	0.194317
O	6.688382	2.070616	0.078596
C	6.291593	2.746559	-0.995348
O	6.997211	3.102061	-1.919663
C	4.782706	3.033459	-0.929064
C	4.425218	3.867512	0.337826
N	5.182886	5.114181	0.337684
C	5.587736	5.807229	1.431324
N	5.322882	5.297934	2.642991
S	6.432877	7.284400	1.247003
C	2.912313	4.045010	0.485845
C	2.148497	2.958058	0.939831
C	0.768007	3.064221	1.071154

C	0.160391	4.277645	0.744872
C	0.893104	5.376235	0.297823
C	2.274640	5.251766	0.169966
N	-1.292531	4.402268	0.881840
O	-1.925819	3.422635	1.282117
O	-1.815139	5.480296	0.589561
C	4.378862	3.701743	-2.238145
O	4.362721	4.896367	-2.426028
C	3.933669	2.743313	-3.369243
F	4.805392	1.727051	-3.505619
F	2.734184	2.221821	-3.035678
F	3.819407	3.378635	-4.535277
H	9.910420	2.607882	0.997206
H	8.436619	3.128575	1.837868
H	8.839023	3.799616	0.237731
H	8.133092	0.850133	0.801170
H	8.499475	1.536668	-0.803331
H	4.270257	2.069792	-0.842591
H	4.779613	3.254674	1.172093
H	5.294446	5.591514	-0.547981
H	4.744191	4.484186	2.794824
H	5.634838	5.804413	3.457682
H	2.635245	2.022555	1.201015
H	0.171433	2.232385	1.423664
H	0.390758	6.304504	0.056637
H	2.852935	6.102960	-0.171341

Aryl Substituent: 4-NO2 Intermediate 16 Conformer 2

C	2.851069	-0.848765	-2.745489
C	4.155784	-0.669869	-1.989512
O	4.510747	0.748252	-1.887244
C	3.992611	1.446612	-0.871688
O	3.256519	1.008151	-0.014314
C	4.474880	2.900439	-0.953447
C	4.222319	3.671790	0.372211
N	5.028381	4.898457	0.328183
C	5.479662	5.609349	1.397384
N	5.311718	5.093899	2.622068
S	6.261495	7.108985	1.153322
C	2.743752	3.965562	0.617251
C	2.082400	3.391932	1.710801
C	0.745636	3.685819	1.966985
C	0.080349	4.560385	1.108629
C	0.711596	5.146515	0.010172
C	2.048379	4.844548	-0.226939

N	-1.325972	4.875908	1.368519
O	-1.871919	4.347973	2.340219
O	-1.899297	5.654890	0.603285
C	5.943405	2.948010	-1.383666
O	6.328203	3.478574	-2.398439
C	7.009072	2.292162	-0.459611
F	6.441245	1.477570	0.458293
F	7.876968	1.575961	-1.182058
F	7.686671	3.247142	0.200435
H	2.651040	-1.918646	-2.864224
H	2.910980	-0.396751	-3.739849
H	2.015166	-0.401809	-2.200292
H	4.999402	-1.109875	-2.522903
H	4.105492	-1.083373	-0.980508
H	3.927480	3.377483	-1.774287
H	4.596591	3.044975	1.185101
H	5.056664	5.411412	-0.544200
H	4.823710	4.230214	2.809587
H	5.685070	5.596016	3.413555
H	2.608965	2.702383	2.362905
H	0.226557	3.249378	2.810910
H	0.165402	5.822201	-0.635678
H	2.547306	5.306041	-1.074068

Aryl Substituent: 4-NO2 Intermediate 16 Conformer 3

C	1.909994	-0.526564	-2.028364
C	3.425565	-0.435863	-2.063990
O	3.869021	0.958968	-2.002068
C	4.007754	1.506282	-0.795369
O	3.782840	0.957533	0.264698
C	4.533675	2.942943	-0.927194
C	4.292604	3.750456	0.380607
N	5.099872	4.970896	0.315247
C	5.506563	5.719788	1.375464
N	5.257950	5.261444	2.609147
S	6.334472	7.192304	1.112333
C	2.806587	4.031868	0.599016
C	2.097210	3.332624	1.584885
C	0.743722	3.583652	1.798361
C	0.112386	4.543276	1.008581
C	0.792152	5.255431	0.018708
C	2.143530	4.993319	-0.179037
N	-1.310630	4.815654	1.225647
O	-1.898406	4.179595	2.103470
O	-1.854464	5.668344	0.519993

C	6.011808	2.949479	-1.344072
O	6.420345	3.572463	-2.294258
C	7.017934	2.103853	-0.515263
F	6.754194	0.791766	-0.692369
F	8.271437	2.342458	-0.897494
F	6.909352	2.373917	0.801986
H	1.611783	-1.572997	-2.149980
H	1.465173	0.055245	-2.840981
H	1.515639	-0.166194	-1.074330
H	3.831870	-0.794459	-3.010667
H	3.887843	-0.980103	-1.238171
H	4.012571	3.421133	-1.761355
H	4.659853	3.136297	1.204845
H	5.203641	5.431542	-0.579963
H	4.726686	4.424878	2.803698
H	5.585262	5.797174	3.398950
H	2.601808	2.582063	2.184177
H	0.187523	3.051813	2.559935
H	0.270745	5.995120	-0.575497
H	2.681035	5.550100	-0.940522

Aryl Substituent: 4-NO2 Intermediate 16 Conformer 4

C	2.909070	-0.826182	-2.721258
C	4.173480	-0.629173	-1.903792
O	4.513153	0.792548	-1.800249
C	3.939507	1.498994	-0.820643
O	3.163947	1.065142	0.003560
C	4.414204	2.955440	-0.897510
C	4.103874	3.741651	0.406545
N	4.885535	4.984313	0.364799
C	5.293237	5.721777	1.433332
N	5.106584	5.222196	2.661676
S	6.047205	7.235112	1.184215
C	2.613372	4.010184	0.601153
C	1.933651	3.453184	1.692018
C	0.584921	3.726622	1.903985
C	-0.073617	4.563686	1.003938
C	0.575871	5.132079	-0.093214
C	1.924390	4.851145	-0.285804
N	-1.492360	4.857549	1.217071
O	-2.054167	4.345960	2.188499
O	-2.059850	5.603024	0.414964
C	5.898338	3.010773	-1.268363
O	6.320434	3.533019	-2.272607
C	6.929846	2.375998	-0.292040

F	6.333183	1.552568	0.599240
F	7.845722	1.675641	-0.968842
F	7.555625	3.345197	0.398557
H	2.724821	-1.898799	-2.840874
H	3.014095	-0.381542	-3.715218
H	2.043238	-0.382721	-2.221885
H	5.045368	-1.068592	-2.390122
H	4.076562	-1.031867	-0.893802
H	3.896835	3.419481	-1.744925
H	4.464963	3.136777	1.241893
H	4.923581	5.484718	-0.514507
H	4.633644	4.350640	2.851099
H	5.445625	5.747103	3.453794
H	2.455714	2.792819	2.377218
H	0.051427	3.302972	2.745433
H	0.034281	5.778347	-0.772208
H	2.436989	5.299216	-1.131923

Aryl Substituent: 4-NO2 Intermediate 16 Conformer 5

C	8.416973	0.437955	-0.567415
C	8.135991	1.738303	0.164105
O	6.714016	2.088983	0.077473
C	6.314617	2.771958	-0.990698
O	7.018781	3.132946	-1.914294
C	4.807088	3.064241	-0.918990
C	4.442861	3.882513	0.355610
N	5.188671	5.136476	0.369634
C	5.559561	5.835013	1.471434
N	5.263165	5.328584	2.676647
S	6.398604	7.318613	1.305947
C	2.927886	4.045207	0.501544
C	2.175777	2.955421	0.967583
C	0.793650	3.047053	1.093563
C	0.172817	4.248905	0.749985
C	0.893967	5.350509	0.291253
C	2.277122	5.240637	0.168920
N	-1.281891	4.357801	0.880522
O	-1.905104	3.375008	1.288845
O	-1.816163	5.426318	0.574649
C	4.406055	3.745348	-2.222707
O	4.395842	4.941418	-2.401341
C	3.955359	2.797575	-3.360713
F	4.817542	1.773150	-3.500058
F	2.750078	2.286074	-3.032703
F	3.850437	3.439589	-4.523738

H	9.468147	0.167531	-0.423523
H	8.233506	0.541196	-1.640450
H	7.796199	-0.373069	-0.175506
H	8.717716	2.569801	-0.237671
H	8.314521	1.650346	1.236510
H	4.293267	2.100223	-0.843330
H	4.800836	3.264246	1.184473
H	5.316909	5.612956	-0.514154
H	4.712408	4.493331	2.814884
H	5.566391	5.827477	3.499223
H	2.672581	2.028871	1.241187
H	0.205394	2.213041	1.454820
H	0.380968	6.269384	0.036859
H	2.846853	6.093766	-0.181883

Aryl Substituent: 4-NO2 Intermediate 2 Conformer 0

H	2.460397	0.334814	0.895316
C	2.090980	0.032980	-0.104322
O	1.108803	0.562179	-0.598953
C	2.876185	-1.039438	-0.763731
C	2.509110	-1.509200	-2.035650
C	3.248592	-2.513335	-2.647248
C	4.352937	-3.032889	-1.965297
C	4.740388	-2.586044	-0.702229
C	3.988802	-1.578652	-0.102533
N	5.139816	-4.095472	-2.607419
O	4.789408	-4.482656	-3.723413
O	6.111020	-4.547242	-1.998545
H	1.645083	-1.079728	-2.531576
H	2.986782	-2.892643	-3.626845
H	5.603264	-3.017656	-0.211453
H	4.266434	-1.210018	0.880899

Aryl Substituent: 4-NO2 Intermediate 2 Conformer 1

H	2.460367	0.334849	0.895315
C	2.090986	0.033039	-0.104352
O	1.108834	0.562312	-0.598933
C	2.876197	-1.039404	-0.763741
C	2.509184	-1.509160	-2.035693
C	3.248655	-2.513312	-2.647272
C	4.352955	-3.032901	-1.965304
C	4.740367	-2.586089	-0.702231
C	3.988781	-1.578699	-0.102546
N	5.139797	-4.095492	-2.607391
O	4.789183	-4.482948	-3.723226
O	6.111215	-4.547027	-1.998692

H	1.645212	-1.079729	-2.531739
H	2.986727	-2.892509	-3.626875
H	5.603190	-3.017655	-0.211336
H	4.266450	-1.210177	0.880917

Aryl Substituent: 4-NO2 Intermediate 2 Conformer 2

H	2.476795	0.352647	0.885222
C	2.086871	0.026428	-0.098784
O	1.082619	0.529125	-0.576602
C	2.873634	-1.043655	-0.760370
C	2.481226	-1.542306	-2.013524
C	3.222408	-2.543858	-2.627132
C	4.354044	-3.031300	-1.966424
C	4.766739	-2.555059	-0.722177
C	4.013153	-1.550885	-0.119834
N	5.142935	-4.090900	-2.610995
O	4.770981	-4.502927	-3.710967
O	6.137179	-4.515402	-2.020020
H	1.596435	-1.136765	-2.492680
H	2.942447	-2.946193	-3.592451
H	5.650385	-2.962956	-0.248300
H	4.309350	-1.159694	0.849338

Aryl Substituent: 4-NO2 Intermediate 2 Conformer 3

H	1.251075	0.416169	-0.704841
C	2.089593	0.026293	-0.095329
O	2.314134	0.465211	1.021141
C	2.888237	-1.044224	-0.741751
C	2.540802	-1.473530	-2.030565
C	3.276036	-2.476767	-2.656637
C	4.353918	-3.031273	-1.966724
C	4.722152	-2.622320	-0.681447
C	3.979138	-1.620581	-0.070324
N	5.136430	-4.090691	-2.619323
O	4.803223	-4.444975	-3.751327
O	6.087381	-4.572834	-2.001870
H	1.695483	-1.022644	-2.542564
H	3.028039	-2.825372	-3.651016
H	5.567527	-3.083463	-0.186752
H	4.231933	-1.274598	0.926329

Aryl Substituent: 4-NO2 Intermediate 2 Conformer 4

H	2.405739	0.274415	0.929705
C	2.090163	0.032088	-0.103965
O	1.160848	0.620476	-0.632756
C	2.876582	-1.039279	-0.763904
C	2.573059	-1.437161	-2.076478

C	3.312955	-2.440787	-2.688218
C	4.353203	-3.032670	-1.965558
C	4.676552	-2.657778	-0.661674
C	3.925674	-1.649951	-0.062104
N	5.139683	-4.095460	-2.607353
O	4.847919	-4.416775	-3.760482
O	6.052164	-4.613140	-1.961252
H	1.757424	-0.952667	-2.602674
H	3.100176	-2.766258	-3.698583
H	5.491217	-3.144107	-0.140597
H	4.153941	-1.336345	0.952593

Aryl Substituent: 4-NO2 Intermediate 3 Conformer 0

H	1.130500	0.134500	0.041411
C	2.058865	0.657031	-0.220776
O	1.815221	2.033218	-0.413019
N	3.027290	0.403704	0.843761
C	2.771553	0.352807	2.176886
N	1.494198	0.312347	2.586543
S	4.063762	0.339248	3.299585
C	2.573012	0.057305	-1.522947
C	2.562835	0.801927	-2.708458
C	3.004941	0.233999	-3.901383
C	3.448080	-1.087902	-3.890134
C	3.463407	-1.854529	-2.723212
C	3.024158	-1.271397	-1.539686
N	3.908556	-1.692503	-5.141050
O	3.890949	-1.003355	-6.164160
O	4.293119	-2.864519	-5.115927
H	1.243541	2.348097	0.301588
H	4.001738	0.550550	0.611174
H	0.709700	0.215844	1.956806
H	1.312196	0.194898	3.571911
H	2.208133	1.824827	-2.696252
H	3.006436	0.800282	-4.824257
H	3.810953	-2.879531	-2.749032
H	3.032859	-1.852849	-0.623373

Aryl Substituent: 4-NO2 Intermediate 3 Conformer 1

H	1.177455	0.198870	0.050932
C	2.217460	0.512740	-0.105210
O	2.309806	1.920529	-0.140904
N	3.023712	-0.073713	0.962862
C	2.676388	-0.177070	2.272078
N	1.403790	0.070660	2.620933
S	3.848375	-0.611918	3.441034

C	2.671147	-0.046224	-1.447404
C	2.855222	0.801178	-2.546997
C	3.237423	0.281665	-3.781614
C	3.425877	-1.094851	-3.899621
C	3.243035	-1.962852	-2.821601
C	2.864727	-1.428238	-1.594579
N	3.825300	-1.647757	-5.194703
O	3.977165	-0.869840	-6.140119
O	3.991619	-2.867033	-5.282525
H	1.778352	2.283026	0.582086
H	4.018486	-0.147085	0.788731
H	0.662196	0.212956	1.949247
H	1.131635	-0.094889	3.578376
H	2.698807	1.866574	-2.434571
H	3.386936	0.926553	-4.638424
H	3.395073	-3.027334	-2.947512
H	2.721113	-2.089547	-0.746297

Aryl Substituent: 4-NO2 Intermediate 3 Conformer 2

H	1.122514	0.156818	0.031467
C	2.048028	0.681935	-0.235641
O	1.796408	2.054540	-0.443134
N	3.017064	0.445651	0.832090
C	2.760518	0.401557	2.165430
N	1.483534	0.352406	2.574582
S	4.052158	0.405460	3.288963
C	2.566393	0.071418	-1.531124
C	2.553308	0.803547	-2.724415
C	2.999731	0.225644	-3.910905
C	3.450220	-1.093590	-3.885371
C	3.468536	-1.847899	-2.710529
C	3.024826	-1.254889	-1.533567
N	3.915589	-1.708564	-5.129394
O	3.895925	-1.030009	-6.159526
O	4.306403	-2.878183	-5.091725
H	1.226453	2.374746	0.270485
H	3.990478	0.599315	0.599596
H	0.699086	0.253424	1.945236
H	1.301118	0.244280	3.560898
H	2.192955	1.824539	-2.723152
H	2.999094	0.782351	-4.839580
H	3.821823	-2.871153	-2.725269
H	3.035937	-1.826642	-0.611215

Aryl Substituent: 4-NO2 Intermediate 3 Conformer 3

H	1.155922	-0.155746	0.078982

C	2.180717	0.214823	-0.051576
O	2.212153	1.623464	0.022682
N	3.014048	-0.418165	0.968269
C	2.674717	-0.639242	2.264733
N	1.395484	-0.466428	2.633419
S	3.865230	-1.125178	3.394289
C	2.655751	-0.218496	-1.432250
C	2.815490	0.719969	-2.459019
C	3.218325	0.313793	-3.729263
C	3.451775	-1.041933	-3.956251
C	3.294137	-1.999263	-2.952184
C	2.894991	-1.577319	-1.688532
N	3.872629	-1.475891	-5.289294
O	4.001239	-0.620195	-6.168648
O	4.079639	-2.678124	-5.473451
H	1.661117	1.906944	0.765964
H	4.010270	-0.438805	0.788459
H	0.646851	-0.289263	1.977998
H	1.133083	-0.718283	3.574607
H	2.623805	1.767326	-2.262743
H	3.349424	1.030022	-4.530639
H	3.481057	-3.044767	-3.162134
H	2.770448	-2.308643	-0.896520

Aryl Substituent: 4-NO2 Intermediate 3 Conformer 4

H	0.993349	-0.105468	-0.034907
C	2.077117	0.064607	-0.044160
O	2.366439	1.424701	0.192587
N	2.675239	-0.818126	0.957143
C	2.181212	-1.084897	2.194381
N	0.919863	-0.718929	2.474408
S	3.157385	-1.868628	3.361443
C	2.594081	-0.321777	-1.423330
C	3.105027	0.645216	-2.296944
C	3.548208	0.283242	-3.567132
C	3.464613	-1.054847	-3.949972
C	2.954060	-2.038295	-3.100320
C	2.521257	-1.661892	-1.833711
N	3.922999	-1.440866	-5.285312
O	4.372624	-0.562066	-6.025264
O	3.838324	-2.627737	-5.611047
H	1.809235	1.736676	0.919821
H	3.663717	-1.011424	0.853117
H	0.276184	-0.375080	1.775328
H	0.529998	-0.993460	3.363585

H	3.154442	1.679952	-1.981984
H	3.949551	1.020102	-4.251485
H	2.900283	-3.068141	-3.429856
H	2.125493	-2.414562	-1.159390

Aryl Substituent: 4-NO2 Intermediate 4 Conformer 0

H	2.462926	-0.928082	-0.227218
C	2.045096	0.071564	-0.065020
N	0.983583	0.247233	0.634991
C	0.306761	-0.856706	1.168229
N	-0.177864	-0.610769	2.386104
S	0.042858	-2.313841	0.343801
C	2.761338	1.191349	-0.684076
C	2.311652	2.519299	-0.551811
C	3.008307	3.556861	-1.154877
C	4.158855	3.251551	-1.888764
C	4.630844	1.947899	-2.036919
C	3.922407	0.916905	-1.427242
N	4.899671	4.346551	-2.526057
O	4.475708	5.495082	-2.382701
O	5.910261	4.065272	-3.173185
H	-0.002894	0.280143	2.833675
H	-0.696063	-1.318364	2.886965
H	1.417734	2.723523	0.026903
H	2.679356	4.584393	-1.065555
H	5.526390	1.754263	-2.613313
H	4.269073	-0.107127	-1.527930

Aryl Substituent: 4-NO2 Intermediate 4 Conformer 1

H	2.463323	-0.921245	-0.208521
C	2.036909	0.077306	-0.062621
N	0.964046	0.253347	0.619478
C	0.284243	-0.847472	1.153269
N	-0.206532	-0.599271	2.368178
S	0.023426	-2.305863	0.329222
C	2.755658	1.194907	-0.682681
C	2.297267	2.521669	-0.570009
C	2.996753	3.557133	-1.173634
C	4.159011	3.251223	-1.888446
C	4.639683	1.948728	-2.016955
C	3.928465	0.919770	-1.406865
N	4.902838	4.343757	-2.526009
O	4.470964	5.491386	-2.399686
O	5.923393	4.061619	-3.156998
H	-0.033452	0.291891	2.815906
H	-0.728608	-1.306303	2.865754

H	1.394481	2.727837	-0.005926
H	2.659822	4.583160	-1.097855
H	5.544163	1.752937	-2.578468
H	4.282949	-0.102918	-1.492831

Aryl Substituent: 4-NO2 Intermediate 4 Conformer 2

H	2.400232	-0.935400	-0.330134
C	2.017509	0.068677	-0.116609
N	0.981490	0.247237	0.619408
C	0.344717	-0.848453	1.212436
N	-0.983937	-0.738537	1.188720
S	1.155891	-2.133339	1.963661
C	2.746919	1.190564	-0.716561
C	2.354512	2.525720	-0.501722
C	3.068341	3.566339	-1.079144
C	4.175946	3.256494	-1.874582
C	4.588906	1.945252	-2.107717
C	3.864479	0.911428	-1.521859
N	4.931729	4.353868	-2.489382
O	4.555870	5.508406	-2.276573
O	5.905719	4.068502	-3.188791
H	-1.422578	0.056154	0.740846
H	-1.566357	-1.453878	1.599890
H	1.488810	2.733719	0.117236
H	2.782097	4.598994	-0.925085
H	5.451795	1.746876	-2.730472
H	4.166509	-0.118123	-1.688567

Aryl Substituent: 4-NO2 Intermediate 4 Conformer 3

H	2.461249	-0.901622	-0.162130
C	1.985874	0.084145	-0.112585
N	0.843109	0.248128	0.448109
C	0.153312	-0.852902	0.968612
N	-0.461037	-0.569070	2.117616
S	0.025342	-2.356181	0.196100
C	2.722553	1.199567	-0.715647
C	2.206320	2.509659	-0.719621
C	2.925050	3.542936	-1.304142
C	4.164742	3.251515	-1.881834
C	4.703990	1.965686	-1.892503
C	3.972550	0.938935	-1.303078
N	4.928609	4.341872	-2.499504
O	4.443592	5.474855	-2.478552
O	6.018087	4.072361	-3.009016
H	-0.366244	0.348764	2.533806
H	-1.007340	-1.271058	2.595913

H	1.243485	2.704510	-0.260561
H	2.545088	4.556565	-1.317396
H	5.667738	1.781545	-2.349661
H	4.370931	-0.071110	-1.298926

Aryl Substituent: 4-NO2 Intermediate 4 Conformer 4

H	1.127297	0.359787	0.460596
C	1.939657	0.069932	-0.214907
N	2.182344	-1.162326	-0.479423
C	1.437216	-2.172559	0.138568
N	1.148758	-3.173184	-0.694331
S	1.021054	-2.184374	1.781740
C	2.728116	1.159522	-0.799547
C	2.388620	2.483976	-0.474167
C	3.112148	3.548938	-1.002717
C	4.175585	3.265104	-1.858807
C	4.537462	1.958536	-2.201924
C	3.806827	0.906251	-1.668334
N	4.943708	4.380759	-2.423632
O	4.617329	5.527913	-2.112187
O	5.878298	4.116481	-3.182721
H	1.424858	-3.123640	-1.666950
H	0.623693	-3.973955	-0.373170
H	1.556042	2.679096	0.194982
H	2.863489	4.574728	-0.762470
H	5.370285	1.784212	-2.871315
H	4.060416	-0.118096	-1.917883

Aryl Substituent: 4-NO2 Intermediate 6 Conformer 1

C	8.821401	1.135863	-3.063485
C	8.148994	0.557399	-1.831156
O	6.729957	0.295192	-2.090302
C	6.407529	-0.878993	-2.624224
O	7.177907	-1.781528	-2.889883
C	4.892551	-0.963560	-2.872624
C	4.420954	0.154230	-3.850544
N	5.148309	0.049803	-5.110540
C	5.454087	1.073287	-5.946852
N	5.109564	2.317457	-5.584403
S	6.278788	0.764756	-7.414765
C	2.897563	0.167316	-3.996604
C	2.128472	0.707085	-2.953378
C	0.740010	0.725449	-3.028307
C	0.129033	0.198390	-4.167189
C	0.865910	-0.338936	-5.221348
C	2.256782	-0.351746	-5.129165

N	-1.332489	0.216879	-4.260354
O	-1.969919	0.693207	-3.318151
O	-1.857774	-0.244630	-5.276130
C	4.578321	-2.374808	-3.353761
O	4.546542	-2.717388	-4.513212
C	4.249742	-3.401575	-2.242811
F	5.149326	-3.330711	-1.244138
F	3.036739	-3.100016	-1.732792
F	4.222036	-4.646959	-2.716475
H	9.858067	1.389815	-2.819506
H	8.312483	2.045905	-3.394580
H	8.829786	0.413408	-3.884476
H	8.133854	1.267285	-1.003113
H	8.615269	-0.374323	-1.506521
H	4.394735	-0.790152	-1.913100
H	4.723508	1.084357	-3.360311
H	5.320107	-0.878863	-5.474723
H	4.536209	2.528733	-4.780154
H	5.344507	3.081241	-6.200088
H	2.617353	1.124160	-2.077490
H	0.139345	1.143367	-2.230426
H	0.360251	-0.736084	-6.092452
H	2.837791	-0.762312	-5.947269

Aryl Substituent: 4-NO2 Intermediate 6 Conformer 2

C	8.838418	1.126926	-3.152987
C	8.149259	0.658260	-1.883829
O	6.729561	0.390173	-2.132651
C	6.399045	-0.818748	-2.576711
O	7.162567	-1.745720	-2.767634
C	4.884444	-0.909601	-2.824958
C	4.423839	0.141763	-3.878531
N	5.152715	-0.054918	-5.126668
C	5.462404	0.905589	-6.033338
N	5.138060	2.176068	-5.754184
S	6.264656	0.486361	-7.486512
C	2.901017	0.156909	-4.030184
C	2.131429	0.775395	-3.032131
C	0.743327	0.797606	-3.116035
C	0.133893	0.194511	-4.217396
C	0.871845	-0.421960	-5.226872
C	2.261050	-0.437372	-5.125624
N	-1.327038	0.216129	-4.318971
O	-1.965305	0.764250	-3.417212
O	-1.850982	-0.314867	-5.300932

C	4.561181	-2.348547	-3.208426
O	4.531989	-2.770869	-4.341385
C	4.219604	-3.293043	-2.030365
F	5.119753	-3.166130	-1.037895
F	3.010510	-2.942132	-1.542978
F	4.175940	-4.567340	-2.418200
H	9.876001	1.388175	-2.920839
H	8.343461	2.012843	-3.561591
H	8.845064	0.340143	-3.912515
H	8.135424	1.433688	-1.116817
H	8.602744	-0.248410	-1.479782
H	4.384550	-0.667690	-1.881605
H	4.733199	1.100547	-3.452517
H	5.307659	-1.008111	-5.430270
H	4.600847	2.455195	-4.945545
H	5.385323	2.894009	-6.418213
H	2.618716	1.250914	-2.185428
H	0.141774	1.275755	-2.353402
H	0.367543	-0.876921	-6.070015
H	2.843315	-0.909230	-5.909052

Aryl Substituent: 4-NO2 Intermediate 6 Conformer 3

C	8.772880	1.055434	-3.095012
C	8.059020	0.621700	-1.827062
O	6.636664	0.380357	-2.088199
C	6.286033	-0.826469	-2.522461
O	7.032152	-1.771247	-2.693832
C	4.773535	-0.887226	-2.791492
C	4.356735	0.151128	-3.876067
N	5.102389	-0.091154	-5.106005
C	5.472337	0.842949	-6.017585
N	5.186032	2.128824	-5.766739
S	6.304837	0.372537	-7.437479
C	2.837535	0.199945	-4.054230
C	2.066173	0.847593	-3.076139
C	0.680660	0.901665	-3.183839
C	0.075487	0.300585	-4.288703
C	0.815432	-0.344256	-5.278760
C	2.202077	-0.391127	-5.153910
N	-1.382681	0.354662	-4.414948
O	-2.022768	0.928565	-3.530709
O	-1.902621	-0.176472	-5.398962
C	4.422231	-2.325839	-3.150182
O	4.388875	-2.768132	-4.275323
C	4.054034	-3.241373	-1.957464

F	4.944352	-3.107759	-0.957174
F	2.844509	-2.863360	-1.491684
F	3.995932	-4.522456	-2.320450
H	9.812614	1.299778	-2.854255
H	8.300829	1.944208	-3.524075
H	8.772977	0.256322	-3.841583
H	8.053163	1.409207	-1.072363
H	8.487949	-0.288056	-1.403569
H	4.264193	-0.612749	-1.862170
H	4.682313	1.111676	-3.466737
H	5.234818	-1.054141	-5.388367
H	4.609995	2.435914	-4.995785
H	5.466895	2.824402	-6.441184
H	2.550232	1.321327	-2.226611
H	0.078149	1.402544	-2.436730
H	0.314662	-0.796797	-6.125296
H	2.785769	-0.885407	-5.922264

Aryl Substituent: 4-NO2 Intermediate 6 Conformer 4

C	2.510726	-2.011623	0.871352
C	4.012697	-1.851374	0.713404
O	4.398416	-1.896528	-0.699218
C	4.352657	-0.755516	-1.385190
O	4.003655	0.323218	-0.949073
C	4.842790	-0.985721	-2.822064
C	4.384058	0.166175	-3.761886
N	5.146047	0.063917	-5.008346
C	5.354007	1.067198	-5.902842
N	4.921382	2.295413	-5.588705
S	6.172919	0.748599	-7.369172
C	2.872196	0.138753	-3.984468
C	2.054369	1.094169	-3.365840
C	0.675594	1.084133	-3.564740
C	0.129466	0.102309	-4.389864
C	0.918136	-0.862366	-5.019580
C	2.293011	-0.835746	-4.811780
N	-1.319576	0.083130	-4.605356
O	-2.003637	0.941603	-4.043430
O	-1.787716	-0.791217	-5.338219
C	6.365085	-1.185832	-2.857387
O	6.887670	-2.133468	-3.392956
C	7.267001	-0.130412	-2.159454
F	7.080176	-0.193987	-0.823863
F	8.553919	-0.353588	-2.421159
F	6.954893	1.118457	-2.562590

H	2.265570	-2.048756	1.937780
H	2.166776	-2.939941	0.405948
H	1.975009	-1.169158	0.425362
H	4.559075	-2.684598	1.157381
H	4.371148	-0.911379	1.137329
H	4.434403	-1.937149	-3.173756
H	4.650405	1.103244	-3.269237
H	5.379483	-0.857052	-5.356950
H	4.375359	2.505812	-4.765678
H	5.093522	3.049443	-6.236593
H	2.494165	1.845837	-2.718610
H	0.035479	1.819812	-3.094482
H	0.460861	-1.609902	-5.655332
H	2.914005	-1.576309	-5.306236

Aryl Substituent: 4-NO2 Intermediate 6 Conformer 5

C	2.799113	-2.547464	1.063186
C	4.023139	-1.674203	0.851694
O	4.444930	-1.699773	-0.551364
C	3.869225	-0.832910	-1.391110
O	3.031817	-0.008962	-1.093365
C	4.434009	-1.041414	-2.801874
C	4.129059	0.160944	-3.738892
N	5.002196	0.031023	-4.912928
C	5.421197	1.038269	-5.726274
N	5.117701	2.297927	-5.385985
S	6.339242	0.679941	-7.121988
C	2.654349	0.254440	-4.124930
C	1.884499	1.348724	-3.709915
C	0.549945	1.463667	-4.090299
C	-0.003061	0.463128	-4.888711
C	0.737758	-0.640212	-5.316077
C	2.070752	-0.734958	-4.930783
N	-1.405946	0.573937	-5.293668
O	-2.046505	1.557166	-4.914046
O	-1.882332	-0.321363	-5.995654
C	5.929589	-1.359656	-2.733458
O	6.414176	-2.392010	-3.132875
C	6.890716	-0.293037	-2.134884
F	6.214752	0.687403	-1.494708
F	7.735331	-0.861177	-1.266469
F	7.607425	0.274813	-3.119298
H	2.551861	-2.563791	2.129666
H	2.991509	-3.574120	0.738130
H	1.936319	-2.156503	0.516784

H	4.892461	-2.053429	1.390402
H	3.841400	-0.635838	1.135072
H	3.971903	-1.950581	-3.202543
H	4.412228	1.070290	-3.203291
H	5.135188	-0.893779	-5.302870
H	4.500782	2.542637	-4.625033
H	5.446191	3.048859	-5.974415
H	2.323917	2.112832	-3.076551
H	-0.052441	2.307105	-3.777410
H	0.276967	-1.398934	-5.935854
H	2.656038	-1.585189	-5.268113

Aryl Substituent: 4-NO2 Intermediate 7 Conformer 0

H	1.016337	0.296209	0.000331
C	2.096482	0.107745	0.036909
N	2.780651	0.921638	-0.969660
C	2.934823	0.583094	-2.260787
S	3.588711	1.658513	-3.406301
N	2.555573	-0.673032	-2.623331
C	1.922981	-1.657077	-1.770927
O	0.530406	-1.720092	-1.955426
C	2.339413	-1.382404	-0.296594
H	3.405106	-1.600498	-0.201688
C	1.562646	-2.261637	0.682245
O	0.420446	-2.037473	1.032881
O	2.313623	-3.282541	1.104067
C	1.701792	-4.228059	2.036133
C	1.878957	-3.764276	3.472063
C	2.423945	-3.048994	-2.240388
F	2.263838	-3.170692	-3.574377
F	3.728450	-3.221423	-1.965951
F	1.732968	-4.037282	-1.649985
C	2.601981	0.469254	1.425200
C	1.687692	0.696790	2.462748
C	2.134328	1.008081	3.744695
C	3.508079	1.092744	3.968905
C	4.441889	0.874952	2.954870
C	3.978830	0.561108	1.680927
N	3.987454	1.425052	5.313292
O	3.151422	1.610232	6.200517
O	5.204650	1.503495	5.494651
H	3.013093	1.876913	-0.730486
H	2.597203	-0.879262	-3.613814
H	0.176027	-0.821245	-2.023136
H	2.229101	-5.164077	1.845215

H	0.648768	-4.342626	1.772087
H	2.937890	-3.625895	3.709684
H	1.470475	-4.522563	4.148037
H	1.349007	-2.824479	3.650230
H	0.622287	0.628838	2.269252
H	1.437618	1.187832	4.553630
H	5.501369	0.950068	3.164337
H	4.694690	0.395071	0.881945

Aryl Substituent: 4-NO2 Intermediate 7 Conformer 1

H	1.024771	0.312010	-0.004677
C	2.103870	0.118553	0.036181
N	2.795146	0.922909	-0.972965
C	2.949877	0.576492	-2.262053
S	3.609872	1.643147	-3.412285
N	2.566541	-0.680050	-2.618037
C	1.928770	-1.657646	-1.761473
O	0.537546	-1.720996	-1.952540
C	2.340676	-1.374856	-0.287463
H	3.404941	-1.597209	-0.186747
C	1.555867	-2.244038	0.693927
O	0.411640	-2.014528	1.034518
O	2.302305	-3.261957	1.130413
C	1.682968	-4.197922	2.067236
C	1.851459	-3.721070	3.499929
C	2.429526	-3.053311	-2.220208
F	2.271601	-3.184455	-3.553541
F	3.733483	-3.224209	-1.942294
F	1.737307	-4.037122	-1.623855
C	2.606820	0.485554	1.424030
C	1.690768	0.721112	2.458232
C	2.135434	1.036721	3.739823
C	3.508913	1.117541	3.967026
C	4.444424	0.891879	2.956328
C	3.983371	0.573865	1.682680
N	3.986265	1.454118	5.311073
O	3.148811	1.646892	6.195327
O	5.203322	1.528285	5.495043
H	3.030640	1.878735	-0.738987
H	2.607774	-0.891458	-3.607444
H	0.178839	-0.822322	-1.996202
H	2.210271	-5.136313	1.888377
H	0.631549	-4.313494	1.797321
H	2.909009	-3.581913	3.743106
H	1.437582	-4.472472	4.180282

H	1.321544	-2.779006	3.665865
H	0.625526	0.655960	2.262722
H	1.437402	1.222575	4.546230
H	5.503665	0.964247	3.168023
H	4.700606	0.401553	0.886277

Aryl Substituent: 4-NO2 Intermediate 7 Conformer 2

H	1.012769	0.209623	0.024781
C	2.096589	0.041115	0.029230
N	2.742022	0.909652	-0.957550
C	2.881296	0.626891	-2.263447
S	3.500369	1.758095	-3.373890
N	2.514621	-0.619320	-2.671769
C	1.908711	-1.643384	-1.849077
O	0.511070	-1.705228	-1.999584
C	2.356960	-1.428436	-0.374212
H	3.428045	-1.632552	-0.314687
C	1.619455	-2.363737	0.582819
O	0.484697	-2.173876	0.975109
O	2.396148	-3.392142	0.934449
C	1.823497	-4.391440	1.834520
C	2.040205	-3.999121	3.286287
C	2.406602	-3.010375	-2.389217
F	2.220330	-3.074313	-3.723849
F	3.716849	-3.189197	-2.148431
F	1.730625	-4.026729	-1.829277
C	2.628753	0.352434	1.419577
C	1.735604	0.516364	2.487176
C	2.207069	0.780224	3.770826
C	3.584004	0.882023	3.966659
C	4.497040	0.727078	2.922670
C	4.009378	0.460262	1.647165
N	4.089395	1.164196	5.312880
O	3.271756	1.294395	6.226651
O	5.309017	1.257461	5.469159
H	2.965687	1.857263	-0.682408
H	2.545353	-0.785252	-3.670195
H	0.161478	-0.806895	-2.092779
H	2.354652	-5.310040	1.580081
H	0.764089	-4.505252	1.597112
H	3.104488	-3.860077	3.498237
H	1.660954	-4.795494	3.934937
H	1.506234	-3.075991	3.528402
H	0.667269	0.435926	2.315864
H	1.526769	0.910771	4.602844

H	5.559698	0.813661	3.110685
H	4.708854	0.343117	0.825353

Aryl Substituent: 4-NO2 Intermediate 7 Conformer 3

H	1.023753	0.214096	0.072038
C	2.110012	0.061445	0.082943
N	2.744032	0.900977	-0.935521
C	2.889478	0.570123	-2.229612
S	3.491547	1.668319	-3.381974
N	2.544766	-0.696611	-2.590059
C	1.950605	-1.697054	-1.729651
O	0.555543	-1.787189	-1.883400
C	2.393033	-1.418484	-0.263716
H	3.467091	-1.602747	-0.196215
C	1.670530	-2.326461	0.730477
O	0.525785	-2.150393	1.100035
O	2.476628	-3.304419	1.152444
C	1.923524	-4.274386	2.096049
C	1.212924	-5.401721	1.365944
C	2.471301	-3.075823	-2.215303
F	2.284406	-3.197932	-3.545145
F	3.784358	-3.222121	-1.969131
F	1.813475	-4.080855	-1.612776
C	2.635662	0.433055	1.461064
C	1.738682	0.623556	2.520836
C	2.204218	0.943328	3.793830
C	3.579069	1.073541	3.986836
C	4.495959	0.893246	2.950200
C	4.014254	0.570840	1.685392
N	4.078035	1.414254	5.321871
O	3.257123	1.566792	6.229217
O	5.295868	1.531865	5.475811
H	2.950343	1.862464	-0.697161
H	2.576770	-0.899118	-3.581750
H	0.185522	-0.895872	-1.966500
H	1.255992	-3.747873	2.780970
H	2.796595	-4.633862	2.642929
H	0.334563	-5.032165	0.829883
H	0.882345	-6.148190	2.095475
H	1.884227	-5.888009	0.652568
H	0.671969	0.520279	2.351681
H	1.520853	1.094476	4.619839
H	5.556882	1.003467	3.135594
H	4.716676	0.434262	0.869087

Aryl Substituent: 4-NO2 Intermediate 7 Conformer 4

H	1.024546	0.267360	0.021608
C	2.101661	0.060555	0.032135
N	2.782723	0.904208	-0.952298
C	2.920925	0.613572	-2.256550
S	3.588177	1.719272	-3.364853
N	2.512230	-0.619358	-2.665061
C	1.865431	-1.619738	-1.844283
O	0.467613	-1.632576	-2.003815
C	2.312325	-1.418216	-0.367268
H	3.375292	-1.659029	-0.301651
C	1.537830	-2.323255	0.589897
O	0.402470	-2.100365	0.963122
O	2.287932	-3.359013	0.975857
C	1.678223	-4.333073	1.879404
C	0.909478	-5.389782	1.103970
C	2.319045	-3.004177	-2.378708
F	2.137176	-3.066261	-3.713457
F	3.620844	-3.227826	-2.130077
F	1.605078	-3.995480	-1.819055
C	2.637101	0.355710	1.424641
C	1.744653	0.561498	2.485306
C	2.218299	0.813611	3.770502
C	3.596844	0.860644	3.974758
C	4.509630	0.662698	2.937735
C	4.019660	0.408613	1.660608
N	4.104690	1.130795	5.322605
O	3.287224	1.301219	6.229881
O	5.326078	1.174463	5.486571
H	3.039699	1.843251	-0.676729
H	2.547150	-0.789953	-3.662641
H	0.150957	-0.722886	-2.104179
H	1.037536	-3.797923	2.583020
H	2.527660	-4.761411	2.413754
H	0.057123	-4.950473	0.578657
H	0.532118	-6.143045	1.803188
H	1.556433	-5.886442	0.375431
H	0.675055	0.523278	2.307255
H	1.538629	0.976028	4.597401
H	5.573699	0.707487	3.132198
H	4.719162	0.259222	0.844018

Aryl Substituent: 4-OH Intermediate 12 Conformer 1

H	1.343694	0.321980	-0.075195
C	2.435620	0.384911	-0.130912
C	3.006034	-1.050418	-0.390694

O	3.195017	-1.587626	0.924094
C	2.097136	-1.927451	-1.227441
C	2.476222	-2.343418	-2.508230
C	1.653142	-3.173590	-3.270642
C	0.427602	-3.603918	-2.748539
O	-0.422658	-4.415727	-3.441161
C	0.036920	-3.203326	-1.462139
C	0.869180	-2.375906	-0.714600
C	2.762063	1.404338	-1.218379
O	3.083549	2.552985	-0.968397
O	2.625966	0.895864	-2.442442
C	2.873841	1.786005	-3.574910
C	1.623923	2.570829	-3.935407
C	2.880199	0.924025	1.216212
N	2.010280	1.125655	2.118254
C	2.185186	1.583673	3.416682
N	2.389036	0.629948	4.324564
S	1.965351	3.218332	3.785951
C	4.375510	1.192968	1.454585
F	5.127179	0.818234	0.400428
F	4.601474	2.500117	1.675060
F	4.817773	0.517196	2.534195
H	3.972089	-0.954855	-0.898823
H	3.601402	-2.461667	0.828205
H	3.426308	-2.014267	-2.920549
H	1.960391	-3.491589	-4.264010
H	-0.041009	-4.618840	-4.308144
H	-0.912273	-3.550647	-1.065223
H	0.566587	-2.083384	0.287469
H	3.172655	1.110300	-4.377899
H	3.708091	2.443191	-3.322078
H	0.789134	1.897781	-4.152624
H	1.822615	3.172683	-4.828358
H	1.335513	3.244767	-3.124054
H	2.507152	-0.338199	4.054303
H	2.398409	0.864645	5.307653

Aryl Substituent: 4-OH Intermediate 12 Conformer 2

H	1.383456	0.439897	0.033504
C	2.476597	0.470645	-0.024741
C	2.998995	-0.968722	-0.353509
O	3.177534	-1.571987	0.933848
C	2.056495	-1.775552	-1.223070
C	2.411134	-2.139062	-2.526650
C	1.555385	-2.904103	-3.320575

C	0.321360	-3.321178	-2.807778
O	-0.560417	-4.070211	-3.531188
C	-0.045088	-2.973268	-1.499136
C	0.819544	-2.210331	-0.720373
C	2.832072	1.528679	-1.065162
O	3.184778	2.655519	-0.763012
O	2.684099	1.080279	-2.311061
C	2.956458	2.013800	-3.402231
C	1.725543	2.841669	-3.730023
C	2.942444	0.934618	1.343113
N	2.081827	1.130016	2.255478
C	2.276766	1.528828	3.570882
N	2.457125	0.533289	4.437898
S	2.104955	3.152217	4.007065
C	4.446973	1.139474	1.587000
F	5.181133	0.796453	0.510050
F	4.718195	2.424498	1.875803
F	4.870217	0.392259	2.626492
H	3.965080	-0.881567	-0.863069
H	3.557627	-2.452070	0.794837
H	3.367452	-1.819397	-2.931921
H	1.843467	-3.181631	-4.331686
H	-0.193722	-4.239406	-4.411773
H	-1.001326	-3.310149	-1.110219
H	0.535264	-1.958651	0.298012
H	3.242773	1.367634	-4.233573
H	3.804424	2.639961	-3.118227
H	0.875988	2.197477	-3.975666
H	1.938774	3.476175	-4.596596
H	1.451237	3.486801	-2.890803
H	2.553773	-0.425156	4.127367
H	2.484307	0.728151	5.429293

Aryl Substituent: 4-OH Intermediate 12 Conformer 3

H	1.257089	0.231257	-0.182623
C	2.347159	0.301808	-0.262535
C	2.929241	-1.136954	-0.470785
O	3.176182	-1.606909	0.860065
C	2.000400	-2.064849	-1.227608
C	2.325094	-2.527137	-2.507454
C	1.481928	-3.402042	-3.194398
C	0.291376	-3.831218	-2.595958
O	-0.575258	-4.686364	-3.211823
C	-0.044851	-3.383398	-1.309720
C	0.806575	-2.511587	-0.638223

C	2.638213	1.280839	-1.396719
O	2.843505	2.466070	-1.200945
O	2.604770	0.696967	-2.593907
C	2.844748	1.541062	-3.762871
C	4.332187	1.667971	-4.045201
C	2.810474	0.903766	1.051095
N	1.962913	1.108604	1.973062
C	2.165220	1.615700	3.249513
N	2.419638	0.697768	4.181214
S	1.916764	3.256139	3.570523
C	4.302923	1.229930	1.227168
F	5.029204	0.828926	0.163486
F	4.493388	2.552555	1.376713
F	4.803823	0.620332	2.319368
H	3.873514	-1.054232	-1.020152
H	3.592240	-2.478878	0.790141
H	3.247222	-2.199242	-2.979449
H	1.747257	-3.755761	-4.187817
H	-0.231349	-4.920231	-4.086756
H	-0.967168	-3.729675	-0.853020
H	0.546988	-2.182415	0.364486
H	2.379057	2.512925	-3.589059
H	2.322062	1.025723	-4.570221
H	4.844775	2.182621	-3.227767
H	4.476287	2.249481	-4.961738
H	4.788400	0.683622	-4.187206
H	2.550652	-0.276083	3.938648
H	2.453957	0.965108	5.155375

Aryl Substituent: 4-OH Intermediate 12 Conformer 4

H	1.179632	0.258625	-0.092248
C	2.262048	0.356197	-0.227467
C	2.862140	-1.062571	-0.511084
O	3.201366	-1.562548	0.787725
C	1.907744	-1.990362	-1.236002
C	2.151220	-2.394125	-2.553184
C	1.284235	-3.267056	-3.212320
C	0.152555	-3.754183	-2.547663
O	-0.732675	-4.611360	-3.133626
C	-0.101496	-3.365504	-1.223933
C	0.772238	-2.494119	-0.581021
C	2.470943	1.375697	-1.344160
O	2.656633	2.559498	-1.122019
O	2.391408	0.826821	-2.555701
C	2.551758	1.710635	-3.708996

C	4.019550	1.878364	-4.063481
C	2.783316	0.927972	1.077804
N	1.983751	1.081567	2.051322
C	2.246018	1.550340	3.331493
N	2.568790	0.607556	4.216105
S	1.982870	3.173110	3.722130
C	4.275373	1.285475	1.182311
F	4.951446	0.930307	0.070553
F	4.442825	2.608163	1.357551
F	4.849152	0.660348	2.228928
H	3.768759	-0.942886	-1.114436
H	3.629766	-2.423030	0.668081
H	3.027394	-2.020626	-3.075973
H	1.485805	-3.574476	-4.235719
H	-0.446949	-4.800657	-4.039641
H	-0.978289	-3.756471	-0.716427
H	0.577636	-2.210615	0.449932
H	2.075947	2.666229	-3.481031
H	1.998149	1.208074	-4.503693
H	4.563780	2.377898	-3.257152
H	4.104698	2.491352	-4.966769
H	4.487059	0.908864	-4.260370
H	2.713593	-0.353065	3.932332
H	2.657710	0.844063	5.194745

Aryl Substituent: 4-OH Intermediate 12 Conformer 5

H	1.382189	0.501299	-0.002822
C	2.476380	0.487019	-0.034928
C	2.951790	-0.976960	-0.324275
O	3.083806	-1.559576	0.978072
C	1.998222	-1.768055	-1.196293
C	2.362965	-2.167394	-2.486519
C	1.496774	-2.920230	-3.280883
C	0.241784	-3.288594	-2.781792
O	-0.650723	-4.024493	-3.505540
C	-0.135729	-2.903418	-1.486797
C	0.739443	-2.153212	-0.707517
C	2.894352	1.510218	-1.087134
O	3.286197	2.627712	-0.798896
O	2.750462	1.044592	-2.327108
C	3.075992	1.946474	-3.430152
C	1.881205	2.810456	-3.796175
C	2.928920	0.958178	1.334799
N	2.056320	1.196696	2.225273
C	2.236244	1.607323	3.538866

N	2.356984	0.617849	4.423317
S	2.119908	3.242340	3.949392
C	4.434141	1.115714	1.608873
F	5.179788	0.717731	0.559166
F	4.744912	2.397802	1.869975
F	4.806186	0.382633	2.677532
H	3.930061	-0.934576	-0.815966
H	3.440066	-2.453127	0.863758
H	3.335738	-1.886129	-2.880928
H	1.793027	-3.225979	-4.281531
H	-0.273018	-4.226526	-4.374422
H	-1.108586	-3.202442	-1.108302
H	0.446339	-1.873139	0.300869
H	3.353900	1.274822	-4.243967
H	3.940464	2.547697	-3.142168
H	1.014180	2.191752	-4.046257
H	2.132547	3.420775	-4.669888
H	1.614027	3.480514	-2.974323
H	2.419468	-0.348173	4.128014
H	2.367577	0.825327	5.412478

Aryl Substituent: 4-OH Intermediate 13 Conformer 0

C	4.841572	-2.563891	-1.059841
C	5.695034	-1.448159	-0.480120
O	5.618969	-0.247801	-1.302685
C	4.642408	0.639928	-1.032955
O	3.764239	0.452160	-0.207436
C	4.821144	1.853468	-1.877761
C	3.826386	2.658917	-2.367223
C	2.377318	2.637078	-2.343178
C	1.733559	3.780884	-2.876895
C	0.352246	3.885018	-2.939058
C	-0.439551	2.823472	-2.475496
O	-1.791609	2.851826	-2.508485
C	0.172162	1.668972	-1.954098
C	1.552098	1.579876	-1.885127
C	6.207865	2.216718	-2.241381
N	6.466106	2.710593	-3.393487
C	7.652601	3.225980	-3.881363
N	8.557179	2.333980	-4.287097
S	7.823520	4.899534	-4.076062
C	7.321953	2.074564	-1.181224
F	6.832061	1.860000	0.053565
F	8.158834	1.060202	-1.482705
F	8.059840	3.200787	-1.127290

H	4.981642	-3.472485	-0.464933
H	5.131773	-2.780444	-2.092367
H	3.781236	-2.297878	-1.037565
H	6.755843	-1.705407	-0.488095
H	5.390680	-1.192672	0.537056
H	4.214056	3.514712	-2.917070
H	2.339827	4.602227	-3.249149
H	-0.117500	4.775062	-3.349022
H	-2.096611	3.694023	-2.879932
H	-0.455700	0.854808	-1.605975
H	1.995657	0.686809	-1.468615
H	8.409393	1.339495	-4.172386
H	9.384770	2.644643	-4.777546

Aryl Substituent: 4-OH Intermediate 13 Conformer 1

C	4.900485	-2.507174	-0.976549
C	5.740126	-1.377033	-0.404710
O	5.655582	-0.185644	-1.239149
C	4.670279	0.695647	-0.980713
O	3.790504	0.507832	-0.157075
C	4.840683	1.904271	-1.834500
C	3.837628	2.691538	-2.336187
C	2.389090	2.643656	-2.323599
C	1.728198	3.778545	-2.855054
C	0.345713	3.857239	-2.927945
C	-0.429601	2.777104	-2.479634
O	-1.781527	2.779533	-2.524277
C	0.199772	1.630375	-1.961906
C	1.580466	1.567296	-1.881028
C	6.224533	2.285259	-2.189068
N	6.484031	2.781816	-3.339835
C	7.666980	3.310771	-3.821204
N	8.585342	2.428973	-4.218413
S	7.818064	4.985573	-4.020519
C	7.333740	2.158451	-1.121892
F	6.838739	1.944758	0.111039
F	8.181528	1.150420	-1.414297
F	8.061051	3.291523	-1.068706
H	5.047504	-3.408136	-0.371806
H	5.197313	-2.731236	-2.005603
H	3.837305	-2.252124	-0.961483
H	6.803459	-1.623935	-0.405729
H	5.429220	-1.114715	0.608767
H	4.215329	3.552077	-2.885606
H	2.322155	4.613709	-3.216286

H	-0.137446	4.741331	-3.335213
H	-2.099780	3.618336	-2.892340
H	-0.415501	0.801469	-1.626294
H	2.035963	0.678642	-1.467850
H	8.450456	1.433134	-4.099640
H	9.412717	2.748918	-4.703298

Aryl Substituent: 4-OH Intermediate 13 Conformer 2

C	4.675843	-2.615645	-1.052846
C	5.612756	-1.555180	-0.498859
O	5.584736	-0.349148	-1.315898
C	4.674966	0.595050	-1.013577
O	3.807825	0.457182	-0.166973
C	4.904244	1.808114	-1.849593
C	3.930582	2.648505	-2.321999
C	2.481493	2.650935	-2.302711
C	1.857476	3.828886	-2.782121
C	0.478127	3.958418	-2.840548
C	-0.331519	2.888241	-2.430899
O	-1.682687	2.939810	-2.464197
C	0.260514	1.698853	-1.967833
C	1.638545	1.584759	-1.901155
C	6.302508	2.142027	-2.199534
N	6.569760	2.720628	-3.308383
C	7.775072	3.165456	-3.814114
N	7.967244	4.482949	-3.746109
S	8.819894	2.108132	-4.629846
C	7.417897	1.868778	-1.165751
F	6.918069	1.701962	0.075684
F	8.142738	0.780323	-1.473366
F	8.265231	2.917803	-1.112897
H	4.780920	-3.533397	-0.464850
H	4.918904	-2.844774	-2.094792
H	3.634409	-2.287437	-0.995326
H	6.655224	-1.876289	-0.540399
H	5.356693	-1.285869	0.528077
H	4.336445	3.510608	-2.848219
H	2.477907	4.656960	-3.113858
H	0.023542	4.874690	-3.207487
H	-1.973749	3.804968	-2.791565
H	-0.381062	0.877725	-1.663985
H	2.064850	0.662499	-1.532719
H	7.314588	5.087111	-3.263574
H	8.774515	4.895669	-4.191878

Aryl Substituent: 4-OH Intermediate 13 Conformer 3

C	5.151288	-2.523805	-1.020671
C	5.946689	-1.370501	-0.431935
O	5.822217	-0.172909	-1.252355
C	4.799899	0.664735	-0.992001
O	3.922000	0.430528	-0.178180
C	4.928760	1.889281	-1.829894
C	3.900931	2.644102	-2.331543
C	2.454897	2.546052	-2.330374
C	1.759966	3.655669	-2.871993
C	0.376288	3.686250	-2.957264
C	-0.365298	2.582215	-2.510059
O	-1.716066	2.537659	-2.566886
C	0.298436	1.460690	-1.980402
C	1.679715	1.445028	-1.888357
C	6.299924	2.324618	-2.172045
N	6.548521	2.839003	-3.317184
C	7.712741	3.417573	-3.786924
N	8.666864	2.575857	-4.186781
S	7.799695	5.098862	-3.970151
C	7.405628	2.232077	-1.097975
F	6.911757	1.984382	0.128902
F	8.296510	1.263265	-1.395381
F	8.084822	3.393626	-1.027335
H	5.327769	-3.425946	-0.425704
H	5.460345	-2.724802	-2.050869
H	4.079680	-2.306983	-1.007659
H	7.018423	-1.577866	-0.431173
H	5.622466	-1.132154	0.583333
H	4.251372	3.521534	-2.872226
H	2.327563	4.509339	-3.232269
H	-0.133157	4.551361	-3.373192
H	-2.059551	3.362506	-2.943596
H	-0.290833	0.613056	-1.644927
H	2.162986	0.575376	-1.466470
H	8.570212	1.574463	-4.078216
H	9.483140	2.932359	-4.664941

Aryl Substituent: 4-OH Intermediate 13 Conformer 4

C	6.395926	-1.017240	1.018090
C	5.634628	-1.288332	-0.269703
O	5.598874	-0.104416	-1.119740
C	4.609606	0.787258	-0.916839
O	3.706162	0.629535	-0.113192
C	4.796254	1.961281	-1.816489
C	3.800464	2.714249	-2.380054

C	2.352400	2.646185	-2.405602
C	1.689472	3.760989	-2.974868
C	0.308020	3.818047	-3.082840
C	-0.462663	2.734765	-2.634442
O	-1.813203	2.716364	-2.713150
C	0.169779	1.606043	-2.082338
C	1.548890	1.565011	-1.966185
C	6.185295	2.344646	-2.148044
N	6.473821	2.781349	-3.315934
C	7.662676	3.307696	-3.786695
N	8.610432	2.425236	-4.105116
S	7.787826	4.972335	-4.070099
C	7.257974	2.297270	-1.038845
F	6.718416	2.148979	0.185409
F	8.127646	1.284739	-1.239005
F	7.969940	3.440575	-1.026029
H	6.479623	-1.947832	1.588868
H	5.875914	-0.279941	1.635697
H	7.403072	-0.649890	0.802778
H	4.608508	-1.606866	-0.076451
H	6.137315	-2.038076	-0.883310
H	4.183335	3.556927	-2.953044
H	2.280070	4.598165	-3.336949
H	-0.177293	4.687350	-3.518423
H	-2.133697	3.542936	-3.106017
H	-0.441381	0.772746	-1.750118
H	2.007629	0.688455	-1.531095
H	8.489076	1.434067	-3.941176
H	9.446295	2.736195	-4.581348

Aryl Substituent: 4-OH Intermediate 14 Conformer 1

C	1.966033	1.157620	-0.621423
C	2.396412	-0.172618	-0.027566
O	3.068406	-0.996481	-1.032013
C	4.379719	-0.816351	-1.196114
O	5.075255	-0.055414	-0.550090
C	4.905111	-1.702518	-2.328612
C	4.330164	-1.217480	-3.699221
O	4.579263	0.167614	-3.870443
C	4.822733	-2.074005	-4.865095
C	4.687523	-3.470151	-4.841573
C	5.113555	-4.255110	-5.912361
C	5.687278	-3.648314	-7.037190
O	6.128236	-4.362502	-8.113885
C	5.819478	-2.255706	-7.079898

C	5.387109	-1.483041	-6.002748
C	6.422576	-1.650508	-2.311220
O	7.090382	-0.829426	-2.900609
C	7.127111	-2.757097	-1.491529
F	6.594807	-2.833615	-0.255803
F	6.932272	-3.944566	-2.102845
F	8.438726	-2.538740	-1.384745
H	1.414020	1.727358	0.133268
H	1.312100	1.005872	-1.485320
H	2.832682	1.747931	-0.931916
H	1.540860	-0.776750	0.278426
H	3.072203	-0.042402	0.819644
H	4.563701	-2.723423	-2.135155
H	3.242007	-1.311306	-3.619774
H	5.542216	0.291515	-3.827847
H	4.236279	-3.965771	-3.985852
H	5.000473	-5.336082	-5.877202
H	5.985623	-5.307395	-7.955294
H	6.258648	-1.791268	-7.957772
H	5.482240	-0.403471	-6.051225

Aryl Substituent: 4-OH Intermediate 14 Conformer 2

C	1.890302	1.041201	-0.576243
C	2.441737	-0.233334	0.039275
O	3.121912	-1.044906	-0.969715
C	4.408417	-0.781966	-1.203305
O	5.078719	0.046348	-0.616248
C	4.941419	-1.670472	-2.330564
C	4.267394	-1.278713	-3.685021
O	4.398453	0.115460	-3.909179
C	4.769830	-2.130819	-4.850079
C	4.795742	-3.530905	-4.767094
C	5.226369	-4.310246	-5.840439
C	5.641995	-3.694132	-7.027762
O	6.079170	-4.401674	-8.110294
C	5.612787	-2.298452	-7.129381
C	5.177558	-1.531950	-6.049306
C	6.450331	-1.510677	-2.391537
O	7.027362	-0.665302	-3.039339
C	7.270930	-2.535925	-1.574412
F	6.800463	-2.613143	-0.313877
F	7.137680	-3.752752	-2.143398
F	8.565944	-2.219391	-1.534419
H	1.337224	1.599476	0.186201
H	1.207122	0.812952	-1.399725

H	2.696886	1.677726	-0.950813
H	1.646701	-0.883690	0.407468
H	3.146181	-0.025801	0.846802
H	4.683230	-2.704362	-2.083997
H	3.194589	-1.451740	-3.549122
H	5.349772	0.312642	-3.930980
H	4.470725	-4.036669	-3.861682
H	5.240232	-5.394420	-5.758092
H	6.052602	-5.348953	-7.909594
H	5.930228	-1.827249	-8.054846
H	5.146795	-0.451560	-6.141982

Aryl Substituent: 4-OH Intermediate 14 Conformer 3

C	3.191810	1.881337	0.055124
C	2.582239	0.489996	0.071610
O	3.026224	-0.285861	-1.086424
C	4.179075	-0.942816	-0.979824
O	4.890778	-0.962633	0.010733
C	4.532520	-1.710548	-2.246888
C	4.147001	-1.001961	-3.586916
O	5.140147	-1.349176	-4.567877
C	2.740012	-1.299942	-4.056372
C	1.848705	-0.256406	-4.333507
C	0.564085	-0.511223	-4.813436
C	0.153172	-1.832377	-5.030347
O	-1.087367	-2.149714	-5.497822
C	1.034055	-2.889754	-4.762187
C	2.310489	-2.618657	-4.278036
C	6.026634	-2.023093	-2.224191
O	6.480026	-3.144314	-2.207026
C	7.045002	-0.848886	-2.211871
F	6.445839	0.359151	-2.280657
F	7.771421	-0.888382	-1.081116
F	7.889949	-0.956301	-3.249679
H	2.792947	2.459405	0.895137
H	2.943030	2.405478	-0.872474
H	4.279680	1.834704	0.156231
H	1.496819	0.521047	-0.034707
H	2.844728	-0.060106	0.977105
H	4.034232	-2.684140	-2.181636
H	4.255386	0.075316	-3.451816
H	4.931763	-2.228097	-4.919270
H	2.158995	0.772307	-4.170344
H	-0.118418	0.309353	-5.021311
H	-1.598670	-1.338693	-5.636789

H	0.705109	-3.910187	-4.932738
H	2.974682	-3.455727	-4.073177

Aryl Substituent: 4-OH Intermediate 14 Conformer 4

C	2.387410	-0.919186	1.321192
C	2.294112	-0.245754	-0.037116
O	2.986688	-1.030892	-1.059039
C	4.294517	-0.820111	-1.213370
O	4.965685	-0.039816	-0.564622
C	4.849826	-1.690946	-2.343637
C	4.266896	-1.231768	-3.719707
O	4.478901	0.158262	-3.901024
C	4.788110	-2.084374	-4.875994
C	4.670819	-3.482107	-4.847923
C	5.124827	-4.266388	-5.907513
C	5.709447	-3.657155	-7.025421
O	6.178305	-4.370506	-8.090833
C	5.823335	-2.263128	-7.073072
C	5.362889	-1.491146	-6.007033
C	6.364953	-1.592364	-2.319616
O	7.010120	-0.753597	-2.909471
C	7.099488	-2.673944	-1.492884
F	6.564483	-2.763485	-0.259263
F	6.943490	-3.868322	-2.101785
F	8.403299	-2.415132	-1.381250
H	1.796763	-0.349487	2.046140
H	3.422194	-0.953470	1.673091
H	1.991575	-1.938319	1.279511
H	2.722311	0.758348	-0.024764
H	1.265025	-0.198559	-0.396697
H	4.539605	-2.721555	-2.147474
H	3.181376	-1.355196	-3.644465
H	5.437626	0.309462	-3.851317
H	4.210839	-3.979360	-3.997663
H	5.025170	-5.348542	-5.869071
H	6.047155	-5.316522	-7.929012
H	6.270612	-1.797039	-7.945955
H	5.443651	-0.410602	-6.059846

Aryl Substituent: 4-OH Intermediate 14 Conformer 5

C	4.589803	-0.442840	1.947364
C	4.659280	0.454037	0.723471
O	5.139499	-0.294715	-0.438770
C	4.242939	-0.979914	-1.153966
O	3.047732	-1.006815	-0.949117
C	4.903961	-1.751955	-2.304432

C	4.281746	-1.285293	-3.656364
O	4.464522	0.111763	-3.821032
C	4.784022	-2.110124	-4.840056
C	4.684830	-3.509476	-4.834568
C	5.120619	-4.269158	-5.919422
C	5.667681	-3.632618	-7.041252
O	6.116486	-4.320805	-8.131718
C	5.762944	-2.236666	-7.066576
C	5.321342	-1.489772	-5.974976
C	6.412609	-1.592483	-2.288511
O	7.026345	-0.746763	-2.904387
C	7.198534	-2.640285	-1.462755
F	6.636255	-2.827518	-0.253512
F	7.160603	-3.817875	-2.123489
F	8.473551	-2.283485	-1.295598
H	4.319062	0.159196	2.820765
H	5.558715	-0.913691	2.138004
H	3.834722	-1.223578	1.820241
H	5.395765	1.249880	0.843089
H	3.689171	0.888291	0.475094
H	4.649538	-2.803290	-2.135229
H	3.200987	-1.428971	-3.548836
H	5.422084	0.276835	-3.803183
H	4.253259	-4.027126	-3.981472
H	5.035672	-5.353058	-5.898224
H	5.998653	-5.271004	-7.985015
H	6.180540	-1.749466	-7.942645
H	5.386530	-0.407458	-6.010920

Aryl Substituent: 4-OH Intermediate 15 Conformer 0

C	3.849016	0.578712	-1.688112
C	2.824047	0.204355	-0.629900
O	3.275823	-0.950087	0.141730
C	4.046573	-0.711244	1.211505
O	4.391081	0.396574	1.584561
C	4.446316	-1.984765	1.899136
C	3.565658	-2.835016	2.509962
C	2.140269	-2.742931	2.724436
C	1.462970	-3.923577	3.110703
C	0.092858	-3.939845	3.323205
C	-0.642067	-2.751967	3.181462
O	-1.977565	-2.692395	3.380326
C	0.013309	-1.554887	2.836046
C	1.378059	-1.554182	2.609055
C	5.865576	-2.369884	1.938623

O	6.307221	-3.348255	2.525789
C	6.899876	-1.491319	1.180941
F	6.437700	-1.103569	-0.030519
F	7.193700	-0.383420	1.888482
F	8.036562	-2.171329	0.984805
H	3.463871	1.411242	-2.285979
H	4.044180	-0.265033	-2.356732
H	4.790790	0.890129	-1.228580
H	1.884133	-0.125598	-1.076011
H	2.630344	1.031106	0.056545
H	4.027199	-3.736357	2.911145
H	2.029752	-4.842851	3.229505
H	-0.412026	-4.859395	3.606258
H	-2.318048	-3.568255	3.620041
H	-0.566306	-0.640444	2.762419
H	1.861128	-0.610514	2.385151

Aryl Substituent: 4-OH Intermediate 15 Conformer 1

C	4.089972	0.709453	-1.573947
C	3.021750	0.312766	-0.567938
O	3.433924	-0.868633	0.184613
C	4.167292	-0.669504	1.288324
O	4.509147	0.423130	1.705900
C	4.528559	-1.966878	1.952227
C	3.617450	-2.825599	2.503673
C	2.185875	-2.724594	2.667353
C	1.482646	-3.909068	2.990439
C	0.105351	-3.916523	3.150528
C	-0.611675	-2.716615	3.018649
O	-1.953202	-2.648272	3.168267
C	0.068292	-1.516729	2.736447
C	1.440622	-1.524336	2.561103
C	5.941554	-2.368347	2.033013
O	6.350998	-3.369503	2.604839
C	7.012504	-1.477206	1.344014
F	6.601484	-1.047053	0.128430
F	7.288687	-0.395060	2.096967
F	8.149807	-2.162446	1.170540
H	3.735210	1.563108	-2.160705
H	4.304270	-0.115598	-2.259825
H	5.015609	0.998096	-1.068984
H	2.097121	0.005406	-1.059730
H	2.808437	1.120242	0.135477
H	4.054816	-3.743387	2.894491
H	2.035402	-4.837858	3.101238

H	-0.419328	-4.838619	3.385162
H	-2.311842	-3.527735	3.364202
H	-0.498831	-0.593953	2.669986
H	1.941398	-0.579685	2.385449

Aryl Substituent: 4-OH Intermediate 15 Conformer 2

C	2.430612	0.181066	-1.571102
C	3.192524	0.539723	-0.311161
O	3.605340	-0.706991	0.322382
C	4.295051	-0.596518	1.464404
O	4.607835	0.464643	1.975651
C	4.642646	-1.940351	2.036386
C	3.715322	-2.843347	2.479492
C	2.277684	-2.757588	2.590202
C	1.566058	-3.965256	2.781735
C	0.182955	-3.986901	2.878458
C	-0.532714	-2.780660	2.815121
O	-1.879965	-2.726146	2.908115
C	0.154030	-1.560740	2.666590
C	1.532825	-1.552898	2.552840
C	6.053861	-2.341152	2.142586
O	6.447427	-3.382608	2.649969
C	7.143601	-1.393452	1.569156
F	6.775517	-0.868699	0.378058
F	7.382500	-0.373958	2.416875
F	8.291991	-2.057454	1.385887
H	2.109516	1.101812	-2.066750
H	1.542509	-0.413840	-1.337578
H	3.060835	-0.383694	-2.264603
H	2.575032	1.100171	0.397388
H	4.088818	1.129239	-0.523927
H	4.140920	-3.789008	2.812651
H	2.117211	-4.899815	2.838771
H	-0.347473	-4.925838	3.011174
H	-2.243353	-3.619764	3.007684
H	-0.413550	-0.635957	2.653262
H	2.037336	-0.596419	2.482088

Aryl Substituent: 4-OH Intermediate 15 Conformer 3

C	2.182939	1.487858	0.412503
C	3.443157	0.852997	-0.151445
O	3.702054	-0.462107	0.430819
C	4.385732	-0.503224	1.582505
O	4.764821	0.477501	2.198112
C	4.666015	-1.917702	2.007933
C	3.708205	-2.822324	2.374602

C	2.279915	-2.685245	2.540784
C	1.509095	-3.870103	2.593877
C	0.129963	-3.833170	2.734498
C	-0.519209	-2.594286	2.858990
O	-1.858223	-2.484134	3.007662
C	0.229557	-1.402392	2.851250
C	1.603233	-1.450691	2.691076
C	6.057806	-2.397461	2.028408
O	6.406913	-3.506425	2.409127
C	7.184935	-1.449517	1.532302
F	6.822900	-0.780770	0.413841
F	7.494096	-0.541654	2.478511
F	8.292442	-2.147447	1.250554
H	1.980577	2.418235	-0.128448
H	2.299346	1.729340	1.472547
H	1.321083	0.825550	0.289308
H	4.320668	1.481256	0.015226
H	3.342877	0.646956	-1.218527
H	4.096925	-3.818093	2.583658
H	2.008889	-4.830815	2.506341
H	-0.448430	-4.752790	2.759216
H	-2.267518	-3.363310	3.001466
H	-0.286001	-0.456744	2.984020
H	2.161736	-0.523287	2.731085

Aryl Substituent: 4-OH Intermediate 15 Conformer 4

C	4.199390	0.859314	-1.449349
C	3.109619	0.423744	-0.483309
O	3.506795	-0.784740	0.233660
C	4.220096	-0.625935	1.356992
O	4.555517	0.450897	1.818467
C	4.567144	-1.946463	1.981774
C	3.644464	-2.822822	2.484020
C	2.210218	-2.724257	2.624171
C	1.498162	-3.918062	2.888021
C	0.118078	-3.927102	3.022203
C	-0.593199	-2.720499	2.924320
O	-1.937075	-2.653588	3.051936
C	0.095052	-1.512792	2.701794
C	1.470331	-1.518216	2.551164
C	5.977948	-2.352663	2.076919
O	6.374695	-3.373493	2.622248
C	7.062963	-1.441081	1.438388
F	6.682130	-0.985397	0.222343
F	7.314639	-0.375305	2.222551

F	8.207332	-2.117313	1.279501
H	3.855895	1.733561	-2.011995
H	4.431104	0.060680	-2.160397
H	5.112681	1.130285	-0.913079
H	2.196781	0.134037	-1.006801
H	2.878940	1.203851	0.245114
H	4.072694	-3.755125	2.849796
H	2.046239	-4.852364	2.972700
H	-0.413244	-4.855917	3.210907
H	-2.301698	-3.539221	3.204440
H	-0.468292	-0.586187	2.661391
H	1.977002	-0.569225	2.421711

Aryl Substituent: 4-OH Intermediate 16 Conformer 0

C	4.146412	3.498553	4.414283
C	5.286125	2.538802	4.710408
O	5.291163	1.424731	3.763080
C	4.516567	0.374874	4.039048
O	3.839616	0.248846	5.042057
C	4.642519	-0.668768	2.927223
C	4.081335	-2.089936	3.286094
N	4.642204	-2.541120	4.561805
C	5.848744	-3.114984	4.770244
N	6.629627	-3.383446	3.709226
S	6.364680	-3.482222	6.363157
C	2.567107	-2.279120	3.278557
C	1.630820	-1.251372	3.441688
C	0.260061	-1.518349	3.423010
C	-0.196840	-2.828454	3.248496
O	-1.519975	-3.150834	3.221349
C	0.728118	-3.870885	3.088601
C	2.088634	-3.589724	3.101651
C	4.092846	-0.142995	1.608372
O	3.433054	0.860014	1.472314
C	4.419417	-0.995401	0.348467
F	5.635920	-1.573919	0.452494
F	3.507052	-1.981360	0.222540
F	4.393755	-0.247365	-0.754893
H	4.217901	4.358907	5.087688
H	3.176297	3.019262	4.571761
H	4.200311	3.861379	3.383694
H	5.226031	2.131655	5.721458
H	6.259873	3.008777	4.564400
H	5.720030	-0.798020	2.763669
H	4.462500	-2.764472	2.514808

H	4.140202	-2.265713	5.395301
H	6.340853	-3.252769	2.750207
H	7.492158	-3.882955	3.864828
H	1.949942	-0.228243	3.591694
H	-0.453175	-0.707267	3.547415
H	-2.052018	-2.348443	3.329818
H	0.367024	-4.884963	2.947962
H	2.796109	-4.404701	2.969024

Aryl Substituent: 4-OH Intermediate 16 Conformer 1

C	2.095241	1.999768	0.435642
C	2.596335	0.605865	0.100321
O	3.076802	-0.080370	1.302057
C	4.331531	0.134983	1.671810
O	5.129233	0.854264	1.092337
C	4.723493	-0.594225	2.955152
C	4.174815	-2.048199	3.110308
N	5.034337	-2.705517	4.113547
C	5.249005	-4.041548	4.238043
N	4.754561	-4.854800	3.293661
S	6.165303	-4.644620	5.550257
C	2.698395	-2.125991	3.466363
C	1.792935	-2.743076	2.596292
C	0.442279	-2.861773	2.925446
C	-0.021026	-2.354820	4.145077
O	-1.325964	-2.438393	4.529036
C	0.874669	-1.731721	5.027037
C	2.219009	-1.623822	4.685956
C	6.251802	-0.538711	3.042009
O	6.850178	0.053053	3.910182
C	7.103048	-1.290302	1.978877
F	6.346061	-1.865941	1.020067
F	7.960668	-0.446580	1.388890
F	7.815025	-2.260683	2.579885
H	1.673445	2.454620	-0.466616
H	2.909811	2.635986	0.792441
H	1.312855	1.960157	1.199262
H	3.406254	0.626878	-0.631085
H	1.793725	-0.038578	-0.260775
H	4.365208	0.014734	3.793663
H	4.336673	-2.559788	2.158776
H	5.280746	-2.174189	4.939613
H	4.099771	-4.552722	2.586770
H	4.922530	-5.846218	3.375822
H	2.138070	-3.130158	1.640409

H	-0.249240	-3.344902	2.239681
H	-1.837737	-2.893671	3.843831
H	0.504532	-1.343311	5.970801
H	2.898626	-1.145381	5.387154

Aryl Substituent: 4-OH Intermediate 16 Conformer 2

C	5.118961	1.525089	-0.907086
C	4.241376	2.014199	0.232411
O	4.552472	1.287776	1.464637
C	3.920999	0.127281	1.687154
O	3.067765	-0.358019	0.977029
C	4.456324	-0.490472	2.983379
C	4.085429	-1.997853	3.111324
N	5.004707	-2.583874	4.102809
C	5.318968	-3.900452	4.219589
N	4.860129	-4.745143	3.284765
S	6.311818	-4.435859	5.505649
C	2.620181	-2.228121	3.454635
C	1.762815	-2.847336	2.538081
C	0.425922	-3.092273	2.853627
C	-0.073106	-2.710520	4.104510
O	-1.367254	-2.919805	4.476020
C	0.773835	-2.086372	5.032687
C	2.105450	-1.852507	4.705425
C	5.962073	-0.247382	3.092687
O	6.486372	0.324629	4.020383
C	6.891194	-0.776586	1.962983
F	6.190042	-1.331925	0.948764
F	7.628323	0.225730	1.461468
F	7.720973	-1.709711	2.454559
H	4.940928	2.144901	-1.791597
H	4.890726	0.486589	-1.162748
H	6.176770	1.598294	-0.638415
H	3.178490	1.890165	0.018332
H	4.443555	3.056164	0.484346
H	4.020130	0.068783	3.818694
H	4.301012	-2.464037	2.146372
H	5.233092	-2.034696	4.921971
H	4.156070	-4.490676	2.606703
H	5.095234	-5.723141	3.363569
H	2.135048	-3.133845	1.558109
H	-0.227571	-3.576897	2.132714
H	-1.845213	-3.358148	3.756231
H	0.376715	-1.796076	6.000453
H	2.748649	-1.376275	5.441475

Aryl Substituent: 4-OH Intermediate 16 Conformer 3

C	4.050154	2.386791	-0.221873
C	2.970583	1.931576	0.745084
O	3.554471	1.229636	1.889830
C	3.783490	-0.077249	1.757957
O	3.529456	-0.745907	0.775684
C	4.447040	-0.630328	3.025730
C	4.123990	-2.146424	3.197609
N	5.017940	-2.676886	4.234609
C	5.305737	-3.986428	4.436839
N	4.795918	-4.882623	3.577907
S	6.325724	-4.457441	5.728639
C	2.647530	-2.378395	3.494609
C	1.800536	-2.900900	2.509359
C	0.446161	-3.118179	2.766027
C	-0.080117	-2.808040	4.025330
O	-1.391406	-2.997458	4.341756
C	0.756806	-2.282916	5.021951
C	2.105084	-2.072997	4.753222
C	5.956166	-0.360927	3.028695
O	6.534604	0.108188	3.980791
C	6.777466	-0.698864	1.754492
F	6.478772	-1.928164	1.290017
F	6.484115	0.195984	0.785638
F	8.086491	-0.642531	2.000353
H	3.587599	2.971176	-1.023882
H	4.564266	1.533060	-0.670754
H	4.785977	3.018376	0.284559
H	2.246705	1.268179	0.268366
H	2.448363	2.776087	1.197005
H	4.066441	-0.075408	3.887222
H	4.374782	-2.633405	2.252313
H	5.313464	-2.062287	4.982013
H	4.060480	-4.656831	2.922729
H	4.994479	-5.859912	3.731071
H	2.197616	-3.133677	1.525125
H	-0.199179	-3.528481	1.993585
H	-1.860963	-3.362765	3.576992
H	0.337891	-2.049877	5.996005
H	2.741869	-1.675751	5.539596

Aryl Substituent: 4-OH Intermediate 16 Conformer 4

C	2.224811	2.109607	0.423993
C	2.466265	0.628337	0.191950
O	2.951073	-0.019465	1.412756

C	4.257091	-0.001182	1.641412
O	5.096008	0.519685	0.924334
C	4.647445	-0.682673	2.951126
C	4.057023	-2.114473	3.167165
N	4.886820	-2.743098	4.211201
C	5.051086	-4.077099	4.408627
N	4.507999	-4.919855	3.517114
S	5.967958	-4.642656	5.736559
C	2.573903	-2.137353	3.501943
C	1.658630	-2.713125	2.613988
C	0.297990	-2.771323	2.917574
C	-0.164887	-2.243969	4.128576
O	-1.479340	-2.267780	4.487505
C	0.741359	-1.663873	5.029121
C	2.095297	-1.616491	4.713923
C	6.177411	-0.669566	3.018024
O	6.797804	-0.026065	3.832927
C	7.006752	-1.526421	2.018758
F	6.240264	-2.171567	1.115032
F	7.877694	-0.751972	1.357018
F	7.705445	-2.452193	2.701865
H	1.791831	2.547684	-0.481276
H	3.160093	2.631334	0.644810
H	1.525505	2.265981	1.250670
H	3.192292	0.451468	-0.603598
H	1.540887	0.096011	-0.032486
H	4.314424	-0.035596	3.770898
H	4.219534	-2.676716	2.244772
H	5.175768	-2.172390	4.995857
H	3.826327	-4.633075	2.828814
H	4.626126	-5.911298	3.662892
H	2.005817	-3.116891	1.665924
H	-0.401467	-3.223714	2.218995
H	-1.998904	-2.694905	3.790132
H	0.371303	-1.261018	5.966855
H	2.783464	-1.171805	5.428650

Aryl Substituent: 4-OH Intermediate 2 Conformer 0

H	2.541978	0.899822	-0.145686
C	2.108556	-0.112963	-0.009126
O	1.167895	-0.482837	-0.704998
C	2.749322	-0.914514	1.037094
C	2.306741	-2.219121	1.326380
C	2.920045	-2.968639	2.318583
C	3.995004	-2.418242	3.041552

O	4.632164	-3.103801	4.021119
C	4.447488	-1.117767	2.763697
C	3.824639	-0.377532	1.767999
H	1.476482	-2.630685	0.760371
H	2.580065	-3.976230	2.544544
H	4.232722	-3.981218	4.123045
H	5.277861	-0.714133	3.334133
H	4.171038	0.629859	1.549291

Aryl Substituent: 4-OH Intermediate 2 Conformer 1

H	2.542195	0.899925	-0.145300
C	2.108673	-0.112856	-0.009130
O	1.168235	-0.482582	-0.705366
C	2.749247	-0.914666	1.036989
C	2.306961	-2.219433	1.326036
C	2.920313	-2.968833	2.318267
C	3.994948	-2.418193	3.041536
O	4.632042	-3.103667	4.021143
C	4.447111	-1.117565	2.763950
C	3.824247	-0.377436	1.768210
H	1.476964	-2.631217	0.759784
H	2.580883	-3.976593	2.544304
H	4.232758	-3.981145	4.123170
H	5.277260	-0.713923	3.334724
H	4.170263	0.630085	1.549484

Aryl Substituent: 4-OH Intermediate 2 Conformer 2

H	2.557267	0.892990	-0.159682
C	2.100388	-0.105739	0.001529
O	1.131871	-0.458303	-0.664470
C	2.746609	-0.911560	1.041205
C	2.276606	-2.199871	1.358509
C	2.894528	-2.952803	2.345082
C	4.001094	-2.422393	3.034563
O	4.641845	-3.112087	4.008947
C	4.482118	-1.138789	2.727393
C	3.854548	-0.394881	1.737394
H	1.421710	-2.596114	0.818900
H	2.535057	-3.948231	2.592863
H	4.217663	-3.974602	4.136115
H	5.337177	-0.750975	3.271753
H	4.221820	0.599856	1.496098

Aryl Substituent: 4-OH Intermediate 2 Conformer 3

H	1.299465	-0.634238	-0.540904
C	2.110275	-0.101993	-0.000969
O	2.396580	1.053955	-0.297258

C	2.764325	-0.890045	1.047106
C	2.343265	-2.208059	1.291818
C	2.947877	-2.975353	2.280634
C	3.990869	-2.422648	3.040863
O	4.622079	-3.115610	4.018995
C	4.423136	-1.102912	2.806286
C	3.812485	-0.348939	1.818308
H	1.534898	-2.631831	0.700591
H	2.622804	-3.994856	2.471096
H	4.242386	-4.004727	4.091166
H	5.231418	-0.700267	3.408675
H	4.134037	0.670122	1.625493

Aryl Substituent: 4-OH Intermediate 3 Conformer 0

H	1.210255	0.272936	-0.284288
C	1.887673	-0.434877	0.209589
O	1.217177	-1.645757	0.505423
N	3.028327	-0.646653	-0.690871
C	2.978373	-0.807237	-2.033957
N	1.803719	-0.608399	-2.659244
S	4.378460	-1.267680	-2.908373
C	2.380303	0.181780	1.503013
C	2.697295	-0.612105	2.610726
C	3.179531	-0.035088	3.786242
C	3.352471	1.352471	3.862048
O	3.815519	1.975259	4.983686
C	3.039590	2.156937	2.757118
C	2.557861	1.569393	1.590819
H	0.852094	-2.003455	-0.317127
H	3.906380	-0.907058	-0.260302
H	1.000290	-0.198018	-2.202738
H	1.794615	-0.631052	-3.667877
H	2.555364	-1.686473	2.560308
H	3.418508	-0.657810	4.645111
H	3.984765	1.316104	5.673104
H	3.172375	3.232025	2.829474
H	2.314055	2.199760	0.739114

Aryl Substituent: 4-OH Intermediate 3 Conformer 1

H	1.158821	0.292313	-0.276567
C	1.973357	-0.323318	0.124975
O	1.538457	-1.655176	0.332556
N	3.087393	-0.255560	-0.829928
C	3.000776	-0.314202	-2.179349
N	1.779859	-0.279260	-2.743405
S	4.415244	-0.444447	-3.138162

C	2.417668	0.256210	1.451356
C	3.031126	-0.547759	2.419301
C	3.470432	-0.001122	3.625185
C	3.299335	1.366685	3.874109
O	3.703982	1.958128	5.034465
C	2.686462	2.180660	2.911402
C	2.251763	1.622834	1.711967
H	1.199864	-2.002145	-0.505931
H	4.017980	-0.384071	-0.453488
H	0.941196	-0.058240	-2.224125
H	1.723310	-0.225760	-3.749293
H	3.156346	-1.609997	2.235858
H	3.941892	-0.632636	4.374552
H	4.102985	1.292838	5.614644
H	2.553298	3.238169	3.117890
H	1.774956	2.260055	0.971188

Aryl Substituent: 4-OH Intermediate 3 Conformer 2

H	1.204907	0.253892	-0.278543
C	1.877060	-0.453909	0.222719
O	1.197157	-1.656396	0.531581
N	3.015578	-0.684480	-0.676299
C	2.960982	-0.868434	-2.016185
N	1.783529	-0.679827	-2.640172
S	4.356754	-1.346348	-2.887713
C	2.375726	0.172516	1.508992
C	2.690595	-0.612624	2.623550
C	3.178332	-0.027264	3.792602
C	3.358761	1.360051	3.854962
O	3.827584	1.990870	4.969734
C	3.048038	2.155791	2.743133
C	2.560943	1.559878	1.583292
H	0.825920	-2.017953	-0.286520
H	3.894185	-0.939652	-0.243754
H	0.984242	-0.255951	-2.188774
H	1.771532	-0.717477	-3.648378
H	2.542951	-1.686666	2.583357
H	3.415836	-0.643238	4.656724
H	3.994759	1.337271	5.664929
H	3.186812	3.230767	2.805066
H	2.319017	2.183482	0.726098

Aryl Substituent: 4-OH Intermediate 3 Conformer 3

H	1.166610	0.656815	-0.277338
C	1.955559	-0.021817	0.069782
O	1.471145	-1.350032	0.157010

N	3.076282	0.089255	-0.873382
C	2.993406	0.147211	-2.222916
N	1.775772	0.267738	-2.783684
S	4.406063	0.050590	-3.187877
C	2.413920	0.421132	1.443056
C	3.001226	-0.485784	2.333157
C	3.454763	-0.064926	3.583370
C	3.324469	1.279144	3.956016
O	3.745157	1.750199	5.164767
C	2.737450	2.195444	3.072362
C	2.288394	1.762616	1.827448
H	1.118677	-1.605597	-0.708233
H	4.000314	-0.102755	-0.507901
H	0.945375	0.484237	-2.248992
H	1.727531	0.416302	-3.780556
H	3.094727	-1.530211	2.053483
H	3.905915	-0.776026	4.271321
H	4.126548	1.024158	5.680361
H	2.635850	3.233221	3.374641
H	1.832147	2.478486	1.148105

Aryl Substituent: 4-OH Intermediate 3 Conformer 4

H	0.884097	0.585922	-0.104182
C	1.836453	0.076409	0.087868
O	1.666189	-1.329705	0.065359
N	2.788194	0.527636	-0.936179
C	2.539461	0.674529	-2.258370
N	1.269571	0.548833	-2.687004
S	3.815877	0.994348	-3.355632
C	2.343114	0.483966	1.455120
C	3.212824	-0.342807	2.176037
C	3.701949	0.051416	3.421479
C	3.322693	1.287671	3.960177
O	3.763414	1.726012	5.174121
C	2.452922	2.123537	3.246076
C	1.971237	1.718256	2.004059
H	1.270794	-1.578214	-0.783211
H	3.767485	0.527663	-0.680955
H	0.478961	0.519683	-2.057301
H	1.073907	0.764245	-3.653249
H	3.500524	-1.306661	1.768574
H	4.373412	-0.598272	3.977702
H	4.345808	1.059026	5.567267
H	2.161107	3.076787	3.676001
H	1.294505	2.370418	1.457442

Aryl Substituent: 4-OH Intermediate 4 Conformer 0

H	2.462799	0.069203	0.878244
C	2.084910	-0.028687	-0.146398
N	1.019263	0.606774	-0.515341
C	0.321864	1.376670	0.414137
N	-0.420466	2.317646	-0.177137
S	0.315019	1.165329	2.105804
C	2.838441	-0.887265	-1.034971
C	2.453920	-1.108376	-2.375718
C	3.195356	-1.942371	-3.195856
C	4.346282	-2.577137	-2.689415
O	5.107867	-3.401463	-3.445328
C	4.745458	-2.367043	-1.359003
C	3.996011	-1.529623	-0.546459
H	-0.391201	2.420636	-1.183602
H	-0.982228	2.953240	0.370289
H	1.567285	-0.613930	-2.759008
H	2.898304	-2.111308	-4.227748
H	4.736278	-3.469736	-4.338328
H	5.635977	-2.864839	-0.989339
H	4.302861	-1.365722	0.483178

Aryl Substituent: 4-OH Intermediate 4 Conformer 1

H	2.460641	0.085620	0.867178
C	2.073325	-0.031184	-0.151917
N	0.993106	0.581661	-0.516680
C	0.292250	1.350265	0.410799
N	-0.467512	2.275794	-0.182766
S	0.300626	1.153195	2.104250
C	2.831357	-0.887912	-1.038491
C	2.435230	-1.132536	-2.371725
C	3.181584	-1.963901	-3.190022
C	4.349244	-2.572208	-2.689311
O	5.116343	-3.392492	-3.443888
C	4.759974	-2.338553	-1.366324
C	4.005450	-1.504042	-0.555569
H	-0.449892	2.368565	-1.190482
H	-1.036900	2.906054	0.362917
H	1.535707	-0.658279	-2.750469
H	2.876056	-2.151497	-4.216215
H	4.735725	-3.478927	-4.331479
H	5.663228	-2.816372	-1.001217
H	4.320761	-1.321750	0.468411

Aryl Substituent: 4-OH Intermediate 4 Conformer 2

H	2.453133	0.038707	0.891891

C	2.039928	-0.110456	-0.112695
N	0.912808	0.432822	-0.444744
C	0.251438	1.254466	0.466035
N	-1.055392	1.340514	0.199665
S	0.959960	2.126014	1.748640
C	2.812796	-0.936134	-1.015735
C	2.399024	-1.196429	-2.340546
C	3.167237	-1.986579	-3.179125
C	4.373798	-2.539269	-2.706989
O	5.162656	-3.318611	-3.481845
C	4.800322	-2.292782	-1.391305
C	4.023589	-1.499874	-0.560160
H	-1.446018	0.813939	-0.571457
H	-1.664514	1.908081	0.770550
H	1.466680	-0.769451	-2.695486
H	2.848349	-2.186823	-4.198868
H	4.769751	-3.417155	-4.362775
H	5.732777	-2.728826	-1.047933
H	4.351177	-1.306753	0.457983

Aryl Substituent: 4-OH Intermediate 4 Conformer 3

H	2.446222	0.070183	0.874658
C	2.047084	-0.071066	-0.136911
N	0.937787	0.499926	-0.487684
C	0.255558	1.292716	0.440537
N	-0.838026	1.832833	-0.103828
S	0.672026	1.590963	2.067805
C	2.814174	-0.910482	-1.029569
C	2.406756	-1.171830	-2.356705
C	3.166722	-1.981733	-3.183333
C	4.359530	-2.552739	-2.697569
O	5.140128	-3.350955	-3.460539
C	4.780976	-2.303933	-1.380414
C	4.012658	-1.491203	-0.561268
H	-1.060786	1.635880	-1.071623
H	-1.445608	2.432927	0.434860
H	1.486572	-0.728022	-2.722308
H	2.852976	-2.182710	-4.204504
H	4.751855	-3.450132	-4.343503
H	5.703121	-2.753763	-1.027173
H	4.335774	-1.296456	0.457970

Aryl Substituent: 4-OH Intermediate 4 Conformer 4

H	2.441686	0.003986	0.915163
C	1.982580	-0.225126	-0.053934
N	0.788042	0.191562	-0.327875

C	0.065238	0.889795	0.637309
N	-0.890129	1.647932	0.091156
S	0.270785	0.791689	2.327213
C	2.780119	-0.997899	-0.982268
C	2.303921	-1.370166	-2.258414
C	3.093350	-2.114797	-3.118807
C	4.386362	-2.505006	-2.719456
O	5.200485	-3.231520	-3.519118
C	4.877504	-2.142704	-1.454202
C	4.078651	-1.396841	-0.600694
H	-0.984527	1.687986	-0.915800
H	-1.497528	2.210781	0.668630
H	1.307659	-1.063455	-2.560253
H	2.725916	-2.400082	-4.101197
H	4.758841	-3.416444	-4.362219
H	5.876650	-2.453149	-1.166349
H	4.456495	-1.114541	0.378515

Aryl Substituent: 4-OH Intermediate 6 Conformer 1

C	2.240307	-1.774695	-0.803882
C	2.680062	-0.435554	-0.238544
O	3.179561	0.440772	-1.300808
C	4.455148	0.326138	-1.642285
O	5.259647	-0.441676	-1.139406
C	4.868589	1.251950	-2.784696
C	4.215504	2.670352	-2.788241
N	5.089646	3.523074	-3.616727
C	5.202592	4.875175	-3.539282
N	4.579892	5.508075	-2.534394
S	6.156459	5.722742	-4.677929
C	2.768765	2.690487	-3.257080
C	1.749230	3.107019	-2.394010
C	0.422710	3.170881	-2.821941
C	0.100303	2.811180	-4.135734
O	-1.173493	2.849950	-4.618454
C	1.111575	2.391149	-5.012541
C	2.429805	2.335907	-4.571803
C	6.398791	1.317284	-2.762575
O	7.099610	0.901690	-3.655765
C	7.112648	1.964508	-1.541274
F	6.247148	2.336589	-0.573077
F	7.995193	1.107316	-1.010462
F	7.780108	3.061473	-1.941248
H	1.804670	-2.376742	0.000223
H	3.088767	-2.323059	-1.222306

H	1.483551	-1.640488	-1.582370
H	3.463538	-0.545928	0.513269
H	1.843307	0.122357	0.183943
H	4.616784	0.738244	-3.719935
H	4.262732	3.051973	-1.765671
H	5.433240	3.135169	-4.486888
H	3.896909	5.064339	-1.937587
H	4.666866	6.511282	-2.470800
H	1.985641	3.376772	-1.367533
H	-0.358989	3.498721	-2.141033
H	-1.774784	3.155417	-3.923109
H	0.850148	2.116629	-6.029810
H	3.200516	2.017027	-5.269436

Aryl Substituent: 4-OH Intermediate 6 Conformer 2

C	5.163982	-1.327466	0.796440
C	4.409426	-1.727132	-0.460038
O	4.741396	-0.824545	-1.563715
C	4.031398	0.304780	-1.687439
O	3.093406	0.626849	-0.991439
C	4.599873	1.127040	-2.848843
C	4.124781	2.610409	-2.802412
N	5.064387	3.389231	-3.627947
C	5.281283	4.728005	-3.542889
N	4.693522	5.401780	-2.543979
S	6.314102	5.503599	-4.664505
C	2.674051	2.790173	-3.227724
C	1.706712	3.201727	-2.303920
C	0.380195	3.399345	-2.689234
C	0.004301	3.180829	-4.019787
O	-1.272455	3.355900	-4.462528
C	0.962471	2.765476	-4.956570
C	2.281661	2.575844	-4.558326
C	6.123345	1.002533	-2.879951
O	6.746657	0.600909	-3.835359
C	6.937210	1.431538	-1.625635
F	6.134063	1.805419	-0.603871
F	7.705852	0.416383	-1.202461
F	7.733052	2.468067	-1.929997
H	4.977124	-2.067378	1.581149
H	4.834579	-0.349956	1.159685
H	6.240088	-1.288128	0.604327
H	3.327886	-1.711067	-0.315401
H	4.714381	-2.709402	-0.823578
H	4.259860	0.659785	-3.779917

H	4.235542	2.949076	-1.769147
H	5.389398	2.977102	-4.493571
H	3.976069	5.005252	-1.953892
H	4.861616	6.393612	-2.467136
H	1.984185	3.360139	-1.265200
H	-0.360247	3.722202	-1.961617
H	-1.834419	3.642917	-3.727415
H	0.659889	2.600333	-5.986011
H	3.012080	2.262917	-5.300345

Aryl Substituent: 4-OH Intermediate 6 Conformer 3

C	4.201719	-2.240694	-0.151918
C	3.128973	-1.622871	-1.032329
O	3.730272	-0.743504	-2.036842
C	3.896552	0.539251	-1.713079
O	3.567075	1.054875	-0.663201
C	4.597225	1.289339	-2.852900
C	4.162905	2.787746	-2.864322
N	5.079464	3.513215	-3.752691
C	5.283566	4.853884	-3.754732
N	4.661445	5.587612	-2.819598
S	6.341371	5.564346	-4.898455
C	2.696520	2.946025	-3.246206
C	1.743650	3.276462	-2.274717
C	0.396543	3.423573	-2.608748
C	-0.014032	3.237076	-3.933724
O	-1.311410	3.367966	-4.327459
C	0.929861	2.903697	-4.917415
C	2.269009	2.761216	-4.570945
C	6.120563	1.137405	-2.773201
O	6.796955	0.837707	-3.729032
C	6.827616	1.374574	-1.410392
F	6.406622	2.515832	-0.831123
F	6.541893	0.351636	-0.575466
F	8.150638	1.437469	-1.563847
H	3.728927	-2.917418	0.567321
H	4.745048	-1.472769	0.405178
H	4.913333	-2.817762	-0.749809
H	2.408191	-1.042841	-0.453756
H	2.603239	-2.375515	-1.621786
H	4.321703	0.820126	-3.800706
H	4.305836	3.164402	-1.849138
H	5.461684	3.029846	-4.555181
H	3.917268	5.224766	-2.240222
H	4.804749	6.586266	-2.818656

H	2.051613	3.413640	-1.241608
H	-0.332595	3.684994	-1.845979
H	-1.860292	3.602635	-3.564330
H	0.599333	2.764079	-5.942038
H	2.988770	2.512317	-5.346548

Aryl Substituent: 4-OH Intermediate 6 Conformer 4

C	5.312128	-0.975154	1.124050
C	4.553228	-1.473634	-0.093886
O	4.862397	-0.647167	-1.262035
C	4.134236	0.459996	-1.459019
O	3.198228	0.817914	-0.778019
C	4.680247	1.204699	-2.682112
C	4.165651	2.673629	-2.750866
N	5.083731	3.410097	-3.637459
C	5.254295	4.757690	-3.666275
N	4.634849	5.493219	-2.731846
S	6.271674	5.470453	-4.842544
C	2.710554	2.777986	-3.186943
C	1.729237	3.225520	-2.295119
C	0.397843	3.352412	-2.693132
C	0.031216	3.024493	-4.003758
O	-1.249775	3.125631	-4.457043
C	1.003586	2.572200	-4.908319
C	2.327434	2.454395	-4.498075
C	6.206665	1.116985	-2.705837
O	6.840269	0.662077	-3.630189
C	7.009095	1.657990	-1.488118
F	6.196603	2.088164	-0.496590
F	7.801415	0.695939	-0.991230
F	7.780006	2.687484	-1.870554
H	5.142550	-1.660721	1.960421
H	4.971217	0.020619	1.420910
H	6.385997	-0.934417	0.920237
H	3.472810	-1.462669	0.059439
H	4.869891	-2.474728	-0.389715
H	4.353277	0.657651	-3.573399
H	4.266502	3.095277	-1.747390
H	5.434144	2.936162	-4.460425
H	3.916454	5.124630	-2.124870
H	4.760230	6.494234	-2.746867
H	2.000756	3.468173	-1.271263
H	-0.353799	3.704804	-1.991122
H	-1.822193	3.444941	-3.743657
H	0.707886	2.322334	-5.922561

H	3.068968	2.111592	-5.215518

Aryl Substituent: 4-OH Intermediate 6 Conformer 5

C	2.211806	-1.639524	-0.725792
C	2.762302	-0.341563	-0.161468
O	3.211964	0.545875	-1.236741
C	4.445945	0.381852	-1.692164
O	5.252538	-0.436262	-1.281153
C	4.812907	1.324052	-2.837766
C	4.094035	2.709739	-2.849199
N	4.931778	3.607500	-3.667374
C	4.942076	4.964849	-3.610204
N	4.214658	5.561936	-2.653553
S	5.902092	5.864641	-4.702377
C	2.652341	2.653960	-3.333743
C	1.599437	2.987387	-2.474423
C	0.276290	2.979967	-2.917290
C	-0.008937	2.629903	-4.242129
O	-1.277235	2.601985	-4.739573
C	1.035805	2.290854	-5.114980
C	2.350076	2.307218	-4.659818
C	6.338250	1.461315	-2.819745
O	7.053482	1.122974	-3.733781
C	7.022609	2.092890	-1.573941
F	6.133318	2.414626	-0.608708
F	7.919781	1.244058	-1.054285
F	7.664657	3.216653	-1.935715
H	1.818288	-2.247839	0.095124
H	2.993688	-2.211608	-1.232805
H	1.398480	-1.444857	-1.430978
H	3.600701	-0.512367	0.516155
H	1.992300	0.239303	0.347967
H	4.585509	0.792388	-3.769018
H	4.112089	3.094446	-1.826337
H	5.354490	3.229869	-4.506295
H	3.500021	5.080607	-2.126138
H	4.213804	6.570271	-2.613665
H	1.807204	3.246347	-1.439067
H	-0.531277	3.243951	-2.238979
H	-1.903143	2.861551	-4.047197
H	0.802733	2.023289	-6.140972
H	3.146538	2.051763	-5.354673

Aryl Substituent: 4-OH Intermediate 7 Conformer 0

H	1.091686	0.190232	0.131352
C	2.178641	0.045538	0.118066

N	2.768733	0.933508	-0.891926
C	2.861751	0.668408	-2.204182
S	3.430015	1.821806	-3.324164
N	2.510166	-0.580409	-2.612857
C	1.912206	-1.589681	-1.771837
O	0.511595	-1.489763	-1.844425
C	2.448926	-1.416920	-0.326022
H	3.523399	-1.606362	-0.320853
C	1.762150	-2.371384	0.641756
O	0.542715	-2.477310	0.712111
O	2.628464	-3.041720	1.385399
C	2.098562	-3.977514	2.382590
C	1.809638	-3.265874	3.692434
C	2.340628	-2.959846	-2.360092
F	2.000031	-3.040291	-3.661005
F	3.669379	-3.156362	-2.266727
F	1.727493	-3.967824	-1.707050
C	2.727286	0.363455	1.494325
C	1.849470	0.653902	2.544982
C	2.328554	0.930059	3.825803
C	3.707348	0.923200	4.067133
O	4.239244	1.188191	5.292997
C	4.599205	0.641637	3.020889
C	4.108174	0.363476	1.749670
H	3.012461	1.872367	-0.604644
H	2.493393	-0.732572	-3.613239
H	0.142361	-1.896011	-1.032422
H	2.893587	-4.716773	2.487936
H	1.208291	-4.453459	1.967989
H	2.703238	-2.757770	4.065943
H	1.494409	-4.004512	4.436536
H	1.008360	-2.531462	3.575230
H	0.777750	0.668215	2.364486
H	1.637718	1.155991	4.633914
H	3.525867	1.362621	5.925148
H	5.666440	0.645516	3.219924
H	4.809662	0.151697	0.946833

Aryl Substituent: 4-OH Intermediate 7 Conformer 1

H	1.098809	0.213379	0.129064
C	2.185233	0.064122	0.121045
N	2.782469	0.937897	-0.897048
C	2.877690	0.657755	-2.206015
S	3.452390	1.796961	-3.337238
N	2.523287	-0.594483	-2.601607

C	1.919531	-1.592329	-1.750963
O	0.519582	-1.489977	-1.830257
C	2.451529	-1.404203	-0.305318
H	3.525426	-1.596772	-0.294263
C	1.758128	-2.345786	0.670503
O	0.538080	-2.445902	0.738108
O	2.619394	-3.012071	1.423680
C	2.082273	-3.936799	2.427557
C	1.792680	-3.212135	3.730047
C	2.346762	-2.970403	-2.321510
F	2.010042	-3.065502	-3.622436
F	3.674736	-3.168742	-2.221664
F	1.729351	-3.969180	-1.658478
C	2.730474	0.395728	1.495474
C	1.850416	0.698908	2.540537
C	2.326394	0.987260	3.819889
C	3.704417	0.979825	4.065156
O	4.233679	1.256270	5.289669
C	4.598665	0.685636	3.024391
C	4.110719	0.395438	1.754695
H	3.028884	1.879042	-0.619982
H	2.506070	-0.757049	-3.600338
H	0.145814	-1.883795	-1.014287
H	2.873755	-4.678518	2.541860
H	1.191202	-4.412269	2.014188
H	2.687897	-2.705947	4.102232
H	1.470679	-3.942300	4.479623
H	0.995994	-2.474307	3.603579
H	0.779239	0.713607	2.356760
H	1.633714	1.223145	4.623545
H	3.519103	1.438203	5.918359
H	5.665335	0.689222	3.226435
H	4.814057	0.173972	0.956109

Aryl Substituent: 4-OH Intermediate 7 Conformer 2

H	1.060299	0.202204	0.157005
C	2.144693	0.035738	0.125129
N	2.746425	0.910300	-0.888862
C	2.831693	0.635509	-2.198186
S	3.414377	1.768684	-3.330819
N	2.440521	-0.606035	-2.600774
C	1.879963	-1.641174	-1.758383
O	0.478904	-1.721332	-1.858047
C	2.389441	-1.429369	-0.304428
H	3.462883	-1.628394	-0.293457

C	1.702529	-2.367276	0.684617
O	0.565194	-2.216082	1.086459
O	2.526168	-3.352221	1.059085
C	2.013674	-4.326551	2.019087
C	2.247819	-3.853695	3.444302
C	2.370293	-2.999440	-2.323439
F	2.111154	-3.074231	-3.645120
F	3.695492	-3.154817	-2.156420
F	1.746804	-4.026051	-1.720972
C	2.719083	0.346792	1.494755
C	1.866115	0.581192	2.579450
C	2.376385	0.842199	3.851066
C	3.761826	0.876329	4.050274
O	4.322544	1.129306	5.266275
C	4.628502	0.649458	2.971153
C	4.106277	0.385198	1.708767
H	3.014591	1.843962	-0.606609
H	2.423469	-0.762032	-3.600907
H	0.109002	-0.826385	-1.858527
H	2.573913	-5.236530	1.797719
H	0.954230	-4.492337	1.814584
H	3.310979	-3.668512	3.624008
H	1.909757	-4.627491	4.141372
H	1.689430	-2.936396	3.650663
H	0.789913	0.557692	2.432971
H	1.704215	1.024460	4.685674
H	3.623644	1.265232	5.923450
H	5.700943	0.683238	3.136648
H	4.789155	0.215658	0.880237

Aryl Substituent: 4-OH Intermediate 7 Conformer 3

H	1.120582	0.268162	0.121962
C	2.202157	0.086993	0.115562
N	2.825468	0.936341	-0.906865
C	2.925822	0.641771	-2.212386
S	3.537079	1.757078	-3.348103
N	2.546847	-0.605736	-2.600401
C	1.900361	-1.574702	-1.748034
O	0.505838	-1.423835	-1.841658
C	2.425736	-1.391696	-0.299175
H	3.492768	-1.618555	-0.276436
C	1.692533	-2.303079	0.675294
O	0.469106	-2.356783	0.735035
O	2.522926	-2.993851	1.440955
C	1.941084	-3.917426	2.420546

C	1.691349	-5.275367	1.787953
C	2.284350	-2.972098	-2.302380
F	1.956193	-3.068044	-3.605243
F	3.603120	-3.216047	-2.187831
F	1.625664	-3.943169	-1.636040
C	2.756992	0.407506	1.489158
C	1.883475	0.671982	2.550170
C	2.368926	0.943640	3.829499
C	3.749657	0.959390	4.058873
O	4.287632	1.222042	5.282682
C	4.636967	0.702531	3.002673
C	4.139754	0.426962	1.733173
H	3.090002	1.874856	-0.637661
H	2.532437	-0.774960	-3.598064
H	0.110416	-1.793969	-1.024915
H	1.025982	-3.471954	2.814025
H	2.693457	-3.966432	3.208598
H	0.946692	-5.208071	0.990192
H	1.315181	-5.962034	2.553112
H	2.616307	-5.690045	1.376768
H	0.810094	0.669258	2.379191
H	1.681265	1.149451	4.645707
H	3.577014	1.379743	5.922287
H	5.705692	0.722258	3.192525
H	4.837372	0.232988	0.922549

Aryl Substituent: 4-OH Intermediate 7 Conformer 4

H	1.043570	0.263748	0.212168
C	2.123740	0.087562	0.167270
N	2.715004	0.957851	-0.859610
C	2.882342	0.657570	-2.155220
S	3.514140	1.778306	-3.275021
N	2.550501	-0.603250	-2.547663
C	1.850765	-1.560562	-1.715476
O	0.455201	-1.397605	-1.738236
C	2.337196	-1.383968	-0.256996
H	3.402475	-1.621600	-0.237662
C	1.615225	-2.304547	0.727164
O	0.492233	-2.110226	1.149053
O	2.403311	-3.325760	1.081243
C	1.853148	-4.308727	2.010893
C	1.066086	-5.379415	1.273588
C	2.198348	-2.962958	-2.276288
F	1.894500	-3.006426	-3.595455
F	3.502758	-3.256094	-2.143621

F	1.485111	-3.921697	-1.663386
C	2.730205	0.395985	1.524565
C	1.902012	0.654985	2.622696
C	2.440198	0.920844	3.881918
C	3.829310	0.934295	4.055110
O	4.417440	1.189925	5.257655
C	4.671866	0.681337	2.962600
C	4.121694	0.413720	1.712977
H	2.943839	1.905334	-0.589277
H	2.618790	-0.790620	-3.540184
H	0.160381	-1.344939	-2.661423
H	1.237403	-3.784551	2.744503
H	2.733344	-4.723355	2.505034
H	0.180704	-4.955823	0.791978
H	0.738103	-6.140179	1.989418
H	1.684904	-5.863729	0.512900
H	0.822987	0.646603	2.496695
H	1.786237	1.121351	4.726809
H	3.733464	1.345606	5.925791
H	5.747524	0.698590	3.108435
H	4.785758	0.225297	0.873266

Aryl Substituent: 4-OMe Intermediate 12 Conformer 0

H	0.706894	-0.204793	0.439273
C	1.780991	-0.089195	0.271779
C	2.075721	-0.440837	-1.222924
O	3.407748	-0.096317	-1.566081
C	1.710356	-1.884330	-1.565991
C	0.443297	-2.399163	-1.267034
C	0.082430	-3.703939	-1.613543
C	1.004805	-4.523013	-2.278910
O	0.761675	-5.810757	-2.663237
C	-0.518866	-6.371969	-2.370265
C	2.274639	-4.016316	-2.592130
C	2.616991	-2.714474	-2.240198
C	2.116702	1.385049	0.534560
O	3.044859	1.767461	1.220800
O	1.255170	2.178380	-0.107734
C	1.454742	3.621899	0.008498
C	0.769773	4.167586	1.249954
C	2.544092	-0.943756	1.261993
N	3.725823	-1.327719	1.010380
C	4.586982	-2.101000	1.790328
N	5.471427	-1.388289	2.483936
S	4.571467	-3.781592	1.680150

C	1.775302	-1.277201	2.545743
F	1.122078	-0.183319	2.987860
F	0.855617	-2.225869	2.277991
F	2.561849	-1.718447	3.536251
H	1.442802	0.231706	-1.812012
H	3.991182	-0.578182	-0.954335
H	-0.295765	-1.781970	-0.762352
H	-0.908779	-4.064070	-1.364329
H	-0.495394	-7.390793	-2.759400
H	-1.322676	-5.812356	-2.862888
H	-0.705663	-6.398637	-1.290290
H	2.980207	-4.656765	-3.113218
H	3.598040	-2.332468	-2.501546
H	1.013859	4.022347	-0.905835
H	2.527716	3.823047	0.014160
H	-0.296803	3.923681	1.246332
H	0.873855	5.257465	1.268730
H	1.223986	3.763902	2.159090
H	5.451519	-0.375882	2.480037
H	6.207347	-1.858102	2.993410

Aryl Substituent: 4-OMe Intermediate 12 Conformer 1

H	0.853426	-0.088787	0.147463
C	1.943667	-0.040671	0.083530
C	2.363326	-0.485846	-1.355136
O	3.739113	-0.224258	-1.578998
C	1.958752	-1.926983	-1.663055
C	0.649346	-2.373804	-1.449363
C	0.258769	-3.676991	-1.768869
C	1.193746	-4.564140	-2.319180
O	0.924278	-5.856957	-2.666981
C	-0.400573	-6.349274	-2.459712
C	2.506850	-4.126393	-2.546446
C	2.878341	-2.825218	-2.223087
C	2.336947	1.425506	0.309465
O	3.207309	1.794503	1.074280
O	1.597408	2.227570	-0.461037
C	1.869645	3.662503	-0.394776
C	1.091783	4.312595	0.736734
C	2.554943	-0.882364	1.184087
N	3.734241	-1.334490	1.073678
C	4.468773	-2.109713	1.972152
N	5.312062	-1.407773	2.725222
S	4.377516	-3.791264	1.931023
C	1.641801	-1.116342	2.392925

F	1.010339	0.029286	2.721013
F	0.701174	-2.026149	2.068085
F	2.296295	-1.553528	3.476782
H	1.828276	0.183957	-2.037050
H	4.233825	-0.705736	-0.893275
H	-0.100282	-1.704394	-1.035164
H	-0.764846	-3.983230	-1.587832
H	-0.391363	-7.385871	-2.799343
H	-1.133953	-5.781064	-3.043864
H	-0.677837	-6.315590	-1.399571
H	3.222550	-4.819275	-2.979192
H	3.893784	-2.496813	-2.417060
H	1.552279	4.034936	-1.370124
H	2.946193	3.804928	-0.282863
H	0.018816	4.127182	0.629770
H	1.258544	5.394556	0.712531
H	1.422958	3.936191	1.708548
H	5.343887	-0.396748	2.678273
H	5.963292	-1.887149	3.331816

Aryl Substituent: 4-OMe Intermediate 12 Conformer 2

H	0.869919	-0.137526	0.275610
C	1.950936	-0.057193	0.133713
C	2.281224	-0.510988	-1.325519
O	3.630626	-0.217125	-1.647869
C	1.894727	-1.966128	-1.586573
C	0.610430	-2.437639	-1.289749
C	0.232231	-3.754706	-1.563830
C	1.154403	-4.630900	-2.152152
O	0.895204	-5.935434	-2.462050
C	-0.404013	-6.452812	-2.170694
C	2.441853	-4.168583	-2.462299
C	2.801236	-2.853764	-2.183263
C	2.314409	1.423034	0.313069
O	3.224057	1.827980	1.011254
O	1.500467	2.192160	-0.414597
C	1.734514	3.635180	-0.385607
C	1.020265	4.279385	0.790324
C	2.665078	-0.863202	1.198506
N	3.846031	-1.283698	1.008611
C	4.667158	-2.020537	1.863648
N	5.539539	-1.279728	2.542737
S	4.625337	-3.704325	1.856242
C	1.850428	-1.102866	2.474806
F	1.204922	0.027352	2.827717

F	0.921325	-2.050331	2.236430
F	2.597081	-1.495556	3.515444
H	1.681715	0.134880	-1.976136
H	4.183804	-0.671198	-0.988506
H	-0.128599	-1.776711	-0.843968
H	-0.771996	-4.080129	-1.318858
H	-0.390691	-7.494735	-2.493479
H	-1.182450	-5.910968	-2.720624
H	-0.620700	-6.406649	-1.097001
H	3.147375	-4.853464	-2.923516
H	3.796066	-2.506786	-2.441260
H	1.337855	3.984180	-1.340373
H	2.811665	3.809602	-0.352676
H	-0.051840	4.062909	0.762125
H	1.153915	5.365092	0.741218
H	1.429968	3.926039	1.740604
H	5.536878	-0.269302	2.476140
H	6.249245	-1.728012	3.106000

Aryl Substituent: 4-OMe Intermediate 12 Conformer 3

H	0.827177	-0.181363	0.432805
C	1.910150	-0.090740	0.315322
C	2.262730	-0.422420	-1.171765
O	3.616216	-0.103749	-1.448849
C	1.880908	-1.851100	-1.557442
C	0.595200	-2.348300	-1.312929
C	0.222860	-3.638256	-1.700280
C	1.152360	-4.460111	-2.352141
O	0.899218	-5.734427	-2.772921
C	-0.400425	-6.277706	-2.535530
C	2.441180	-3.970923	-2.610957
C	2.794761	-2.683645	-2.218885
C	2.271121	1.369023	0.620904
O	3.176634	1.714906	1.355069
O	1.459603	2.195552	-0.044199
C	1.690955	3.631166	0.105868
C	0.961969	4.175214	1.322700
C	2.606795	-0.984717	1.319538
N	3.792331	-1.385452	1.116775
C	4.600062	-2.194884	1.916342
N	5.459357	-1.518422	2.674507
S	4.563625	-3.871410	1.754998
C	1.768444	-1.333548	2.554554
F	1.128430	-0.232901	3.000015
F	0.834040	-2.243631	2.214090

F	2.492768	-1.829365	3.566248
H	1.672516	0.275764	-1.775178
H	4.160551	-0.611349	-0.822081
H	-0.149510	-1.728477	-0.820040
H	-0.782529	-3.985093	-1.492351
H	-0.382138	-7.288182	-2.946091
H	-1.176904	-5.692568	-3.042093
H	-0.624087	-6.323954	-1.463272
H	3.152236	-4.613263	-3.122155
H	3.790773	-2.314121	-2.437933
H	1.304791	4.057727	-0.821329
H	2.767280	3.803388	0.165789
H	-0.109064	3.959279	1.264994
H	1.092568	5.261602	1.364436
H	1.362501	3.745905	2.245243
H	5.456071	-0.506161	2.701149
H	6.160173	-2.016289	3.206374

Aryl Substituent: 4-OMe Intermediate 12 Conformer 4

H	0.952206	-0.023922	0.295901
C	2.043676	-0.005015	0.239108
C	2.459695	-0.408189	-1.212983
O	3.843669	-0.177427	-1.418707
C	2.017059	-1.824310	-1.578537
C	0.696883	-2.246575	-1.381720
C	0.273617	-3.525089	-1.754771
C	1.185723	-4.411771	-2.342897
O	0.883768	-5.681806	-2.743875
C	-0.452531	-6.149622	-2.554347
C	2.509260	-3.998032	-2.553873
C	2.913446	-2.721279	-2.177065
C	2.481677	1.438931	0.517267
O	3.357047	1.753082	1.300745
O	1.770317	2.289962	-0.226668
C	2.107912	3.709048	-0.133913
C	3.228036	4.062831	-1.097262
C	2.623055	-0.904380	1.310992
N	3.787480	-1.390618	1.189217
C	4.491342	-2.222554	2.060700
N	5.357192	-1.578316	2.839385
S	4.339795	-3.897441	1.961504
C	1.697082	-1.152175	2.507246
F	1.099726	0.000097	2.873359
F	0.730500	-2.020534	2.146972
F	2.332500	-1.647458	3.577499

H	1.948892	0.302493	-1.871623
H	4.319803	-0.703987	-0.753395
H	-0.035444	-1.576455	-0.938683
H	-0.757159	-3.813195	-1.584892
H	-0.469776	-7.170440	-2.938561
H	-1.172647	-5.538673	-3.111209
H	-0.726910	-6.155108	-1.492938
H	3.207042	-4.690082	-3.016212
H	3.936708	-2.410489	-2.358538
H	2.373459	3.933411	0.900851
H	1.177369	4.218375	-0.389796
H	4.151451	3.538638	-0.835544
H	3.418359	5.140053	-1.049112
H	2.954006	3.806974	-2.125114
H	5.427187	-0.568210	2.826513
H	5.990629	-2.102138	3.427776

Aryl Substituent: 4-OMe Intermediate 13 Conformer 0

C	4.869444	2.592015	-0.929426
C	5.779468	1.411018	-0.635508
O	5.643089	0.975416	0.748231
C	4.704840	0.049776	1.027067
O	3.902331	-0.369916	0.210009
C	4.820555	-0.369373	2.451578
C	3.793386	-0.761531	3.269569
C	2.352250	-0.845914	3.142071
C	1.671126	-1.461076	4.218358
C	0.288332	-1.595202	4.245398
C	-0.468330	-1.089725	3.175931
O	-1.815329	-1.156669	3.088628
C	-2.543675	-1.793774	4.146825
C	0.185299	-0.456084	2.098641
C	1.562214	-0.340265	2.077861
C	6.173262	-0.348389	3.048589
N	6.333955	-0.016012	4.274111
C	7.476804	-0.053247	5.050827
N	8.340046	0.948092	4.875530
S	7.638046	-1.239309	6.248929
C	7.374780	-0.818581	2.199922
F	6.993505	-1.439204	1.068532
F	8.171251	0.216965	1.862919
F	8.126295	-1.691342	2.899577
H	5.050107	2.942423	-1.951005
H	5.069300	3.419105	-0.241513
H	3.817386	2.306238	-0.844634

H	6.833414	1.681310	-0.723796
H	5.565788	0.563131	-1.289658
H	4.137484	-1.073873	4.253934
H	2.247802	-1.844120	5.056073
H	-0.186538	-2.079265	5.090044
H	-3.593395	-1.735117	3.858930
H	-2.392579	-1.271773	5.097489
H	-2.247859	-2.842972	4.250836
H	-0.418019	-0.064684	1.285444
H	2.032387	0.140754	1.232009
H	8.200230	1.649739	4.159928
H	9.124551	1.051435	5.504752

Aryl Substituent: 4-OMe Intermediate 13 Conformer 1

C	4.942655	2.503575	-0.929436
C	5.826787	1.301208	-0.643168
O	5.685980	0.863878	0.739483
C	4.728016	-0.041269	1.018812
O	3.913082	-0.439756	0.203515
C	4.839810	-0.467285	2.441653
C	3.807678	-0.837768	3.263457
C	2.364385	-0.888539	3.143449
C	1.674885	-1.484607	4.225194
C	0.289508	-1.586194	4.260161
C	-0.460960	-1.066765	3.193103
O	-1.809599	-1.102578	3.113166
C	-2.546896	-1.718425	4.177714
C	0.201376	-0.452038	2.110054
C	1.580448	-0.368173	2.081706
C	6.195420	-0.478634	3.032719
N	6.368523	-0.152875	4.258439
C	7.513594	-0.216627	5.030475
N	8.395786	0.768072	4.855586
S	7.655480	-1.410811	6.222958
C	7.382694	-0.972285	2.177403
F	6.982976	-1.581975	1.046422
F	8.199505	0.046814	1.838945
F	8.118796	-1.862518	2.871397
H	5.127204	2.853477	-1.950482
H	5.163821	3.323427	-0.239376
H	3.884713	2.241424	-0.841428
H	6.886249	1.547874	-0.734740
H	5.591600	0.460765	-1.299571
H	4.149160	-1.159571	4.245674
H	2.246912	-1.878396	5.061106

H	-0.191957	-2.055933	5.109162
H	-3.596522	-1.636401	3.895240
H	-2.378425	-1.196528	5.125508
H	-2.275108	-2.773852	4.284275
H	-0.397016	-0.049729	1.298539
H	2.057227	0.099014	1.231832
H	8.264187	1.478071	4.146682
H	9.182126	0.855432	5.484875

Aryl Substituent: 4-OMe Intermediate 13 Conformer 2

C	5.149172	2.530528	-0.897540
C	6.000339	1.304192	-0.613015
O	5.833052	0.856947	0.763492
C	4.847816	-0.024310	1.023433
O	4.031804	-0.392935	0.195338
C	4.931316	-0.466361	2.443413
C	3.879706	-0.817111	3.248873
C	2.437153	-0.828718	3.110117
C	1.718325	-1.421172	4.174591
C	0.330424	-1.487454	4.190059
C	-0.392113	-0.934413	3.120645
O	-1.740075	-0.934389	3.022548
C	-2.507122	-1.544433	4.069211
C	0.300109	-0.322184	2.055006
C	1.681138	-0.273608	2.045753
C	6.278865	-0.520259	3.050765
N	6.445007	-0.211613	4.281764
C	7.576478	-0.314629	5.069116
N	8.486974	0.648075	4.918844
S	7.669250	-1.526173	6.249142
C	7.463066	-1.037557	2.205173
F	7.060687	-1.627852	1.064888
F	8.309566	-0.037250	1.884195
F	8.168121	-1.951845	2.899999
H	5.354698	2.885179	-1.912960
H	5.382421	3.338182	-0.197163
H	4.084081	2.294066	-0.824572
H	7.066383	1.526255	-0.690003
H	5.752939	0.475939	-1.280342
H	4.200147	-1.157422	4.231951
H	2.268894	-1.840509	5.012446
H	-0.174278	-1.955914	5.026190
H	-3.550481	-1.430933	3.774670
H	-2.337229	-1.039848	5.026081
H	-2.264798	-2.608055	4.164917

H	-0.276922	0.106732	1.241661
H	2.181195	0.192900	1.208936
H	8.384777	1.369902	4.217062
H	9.266318	0.706548	5.560119

Aryl Substituent: 4-OMe Intermediate 13 Conformer 3

C	6.590465	0.167308	-1.493754
C	5.714417	1.119279	-0.695219
O	5.624108	0.706402	0.700219
C	4.667765	-0.182361	1.033289
O	3.833107	-0.608163	0.253177
C	4.794848	-0.543673	2.474350
C	3.765071	-0.849977	3.323753
C	2.319417	-0.863110	3.215111
C	1.620620	-1.445282	4.297726
C	0.233058	-1.513892	4.337329
C	-0.508659	-0.970020	3.276509
O	-1.858414	-0.972435	3.201812
C	-2.605922	-1.577872	4.265010
C	0.163957	-0.362660	2.195710
C	1.544733	-0.313732	2.161783
C	6.157298	-0.564648	3.048131
N	6.357310	-0.177187	4.251564
C	7.511781	-0.235939	5.010526
N	8.422874	0.706989	4.767326
S	7.632608	-1.365315	6.266483
C	7.312952	-1.146209	2.206135
F	6.869129	-1.809699	1.122208
F	8.153684	-0.176604	1.788210
F	8.035654	-2.015397	2.938604
H	6.706276	0.553634	-2.511655
H	6.140341	-0.827413	-1.552054
H	7.581489	0.080021	-1.040069
H	4.703984	1.181971	-1.103516
H	6.148437	2.119616	-0.646911
H	4.107692	-1.144166	4.314239
H	2.186528	-1.856939	5.129211
H	-0.256411	-1.976168	5.185868
H	-3.654443	-1.469474	3.987301
H	-2.421755	-1.066584	5.215693
H	-2.358734	-2.640132	4.363625
H	-0.427636	0.063475	1.391417
H	2.028187	0.153343	1.315457
H	8.302579	1.380995	4.022159
H	9.221005	0.803723	5.380243

Aryl Substituent: 4-OMe Intermediate 13 Conformer 4

C	4.860104	2.441428	-0.934325
C	5.738018	1.233520	-0.652414
O	5.634336	0.824030	0.742084
C	4.675450	-0.064608	1.067605
O	3.832051	-0.469399	0.284974
C	4.825182	-0.464250	2.494474
C	3.814398	-0.811713	3.352154
C	2.367881	-0.851874	3.273993
C	1.704371	-1.425611	4.383725
C	0.319718	-1.514145	4.459586
C	-0.456378	-1.003815	3.406524
O	-1.806993	-1.028811	3.365558
C	-2.518918	-1.619383	4.461313
C	0.180071	-0.411319	2.295929
C	1.558469	-0.340294	2.227248
C	6.197103	-0.475386	3.046296
N	6.408627	-0.125198	4.259216
C	7.575779	-0.182272	4.997974
N	8.461018	0.789777	4.774237
S	7.743684	-1.351255	6.211754
C	7.354541	-0.998857	2.167950
F	6.916630	-1.627264	1.061607
F	8.171863	0.004093	1.785299
F	8.100994	-1.882535	2.859268
H	5.016845	2.769316	-1.967275
H	5.112844	3.271476	-0.267692
H	3.802308	2.194600	-0.808410
H	6.797086	1.464914	-0.780858
H	5.472427	0.383936	-1.284998
H	4.181296	-1.119156	4.329802
H	2.296565	-1.812301	5.208849
H	-0.141265	-1.967061	5.328827
H	-3.575495	-1.530303	4.208475
H	-2.316993	-1.084182	5.395043
H	-2.255108	-2.675852	4.577126
H	-0.437772	-0.015662	1.495802
H	2.014740	0.110028	1.357236
H	8.313369	1.486068	4.054971
H	9.266055	0.885017	5.378274

Aryl Substituent: 4-OMe Intermediate 14 Conformer 1

C	8.675194	-2.207717	-2.714140
C	8.069023	-1.340251	-1.624487
O	6.633452	-1.591572	-1.498135

C	6.253767	-2.596452	-0.708357
O	6.997696	-3.297063	-0.047916
C	4.732045	-2.761455	-0.741938
C	4.273452	-3.232801	-2.158932
O	5.006286	-4.382440	-2.550370
C	2.758981	-3.420019	-2.245513
C	1.876557	-2.422169	-1.814111
C	0.491145	-2.574915	-1.918061
C	-0.036359	-3.751474	-2.467389
O	-1.368380	-4.009368	-2.613575
C	-2.300359	-3.018299	-2.176521
C	0.837359	-4.755588	-2.911578
C	2.213967	-4.586379	-2.802514
C	4.337410	-3.760037	0.332376
O	4.281305	-4.959095	0.169946
C	3.961461	-3.160334	1.707700
F	4.906715	-2.290472	2.114582
F	2.799588	-2.484279	1.583683
F	3.812333	-4.104056	2.638343
H	9.733858	-1.953216	-2.829699
H	8.173542	-2.038470	-3.671562
H	8.602578	-3.268400	-2.458175
H	8.128770	-0.278818	-1.870065
H	8.536234	-1.516936	-0.653958
H	4.290952	-1.784318	-0.524921
H	4.578652	-2.443263	-2.853669
H	4.830991	-5.071340	-1.887601
H	2.256357	-1.494519	-1.393895
H	-0.157227	-1.778781	-1.571630
H	-3.290268	-3.427413	-2.382748
H	-2.171915	-2.079242	-2.727298
H	-2.202162	-2.824876	-1.101971
H	0.419410	-5.661253	-3.341145
H	2.877089	-5.366517	-3.160655

Aryl Substituent: 4-OMe Intermediate 14 Conformer 2

C	8.575619	-1.932771	-2.753143
C	8.002443	-1.229234	-1.535029
O	6.581933	-1.540058	-1.376441
C	6.267560	-2.646973	-0.702504
O	7.060682	-3.399199	-0.168238
C	4.750918	-2.857258	-0.692509
C	4.244717	-3.180838	-2.135296
O	4.989944	-4.255378	-2.683717
C	2.733391	-3.402612	-2.180997

C	1.848025	-2.464070	-1.637023
C	0.463361	-2.643333	-1.697407
C	-0.060122	-3.786759	-2.316494
O	-1.391041	-4.066454	-2.429113
C	-2.326031	-3.134807	-1.881495
C	0.816230	-4.730334	-2.873518
C	2.192284	-4.535115	-2.806770
C	4.432846	-3.982658	0.276202
O	4.399196	-5.156922	-0.019708
C	4.110415	-3.554513	1.727297
F	5.060617	-2.719177	2.191106
F	2.934434	-2.891812	1.734883
F	4.018482	-4.603002	2.546782
H	9.621631	-1.635842	-2.881833
H	8.025173	-1.657293	-3.657622
H	8.539122	-3.019097	-2.632775
H	8.022807	-0.143933	-1.645577
H	8.521502	-1.512139	-0.617420
H	4.290979	-1.926343	-0.348394
H	4.497980	-2.308400	-2.746680
H	4.867272	-5.017953	-2.094001
H	2.226154	-1.563091	-1.160756
H	-0.187670	-1.892996	-1.264594
H	-3.314259	-3.551157	-2.081184
H	-2.239654	-2.153535	-2.362406
H	-2.189398	-3.021868	-0.799716
H	0.400736	-5.609653	-3.356799
H	2.856421	-5.266920	-3.254210

Aryl Substituent: 4-OMe Intermediate 14 Conformer 3

C	8.738012	-2.281937	-2.620816
C	8.094736	-1.367072	-1.592928
O	6.653354	-1.604582	-1.514778
C	6.234849	-2.575869	-0.703220
O	6.946499	-3.254487	0.013632
C	4.714579	-2.733994	-0.790791
C	4.307608	-3.242984	-2.210167
O	5.051293	-4.404597	-2.541672
C	2.797239	-3.430367	-2.349800
C	1.900978	-2.422821	-1.972901
C	0.520242	-2.576126	-2.126586
C	0.011795	-3.763117	-2.671355
O	-1.314097	-4.022558	-2.863093
C	-2.260100	-3.020332	-2.485921
C	0.899856	-4.777325	-3.060853

C	2.271619	-4.607415	-2.903031
C	4.274245	-3.699960	0.295854
O	4.213294	-4.902519	0.163946
C	3.857212	-3.060110	1.640938
F	4.801084	-2.196120	2.062174
F	2.712431	-2.368403	1.457058
F	3.657180	-3.978262	2.587728
H	9.802044	-2.037430	-2.704290
H	8.275962	-2.150105	-3.603696
H	8.648560	-3.330729	-2.324461
H	8.172122	-0.317009	-1.879073
H	8.521117	-1.506376	-0.597812
H	4.271352	-1.748890	-0.617062
H	4.641543	-2.473736	-2.914401
H	4.846212	-5.075627	-1.869318
H	2.266834	-1.487891	-1.556715
H	-0.139507	-1.772491	-1.821348
H	-3.242038	-3.431903	-2.723077
H	-2.106999	-2.093812	-3.051432
H	-2.204819	-2.803545	-1.412757
H	0.496803	-5.691266	-3.487077
H	2.946308	-5.395534	-3.219898

Aryl Substituent: 4-OMe Intermediate 14 Conformer 4

C	8.524458	-0.396952	-0.516902
C	8.070740	-1.332648	-1.624111
O	6.636801	-1.605747	-1.523589
C	6.257178	-2.606841	-0.728906
O	7.002085	-3.305584	-0.067574
C	4.735684	-2.775661	-0.763386
C	4.272470	-3.247606	-2.178601
O	5.000731	-4.399976	-2.570828
C	2.757189	-3.430220	-2.259896
C	1.881256	-2.422186	-1.838964
C	0.494837	-2.569482	-1.935937
C	-0.040310	-3.750866	-2.467271
O	-1.374101	-4.004270	-2.604947
C	-2.299575	-3.002284	-2.179206
C	0.826875	-4.765081	-2.901114
C	2.204765	-4.601169	-2.799413
C	4.344535	-3.773611	0.313081
O	4.283887	-4.972450	0.150296
C	3.979555	-3.173708	1.691471
F	4.931568	-2.309744	2.094666
F	2.820727	-2.491158	1.575351

F	3.830558	-4.118721	2.621148
H	9.582202	-0.154398	-0.662902
H	8.412379	-0.865611	0.464707
H	7.951829	0.535119	-0.534169
H	8.605432	-2.283996	-1.598603
H	8.181819	-0.878182	-2.609826
H	4.293573	-1.799108	-0.545251
H	4.577365	-2.459749	-2.875412
H	4.828558	-5.086505	-1.904859
H	2.267983	-1.491110	-1.432852
H	-0.148417	-1.765486	-1.598226
H	-3.292239	-3.409175	-2.376408
H	-2.168223	-2.071839	-2.743748
H	-2.197020	-2.794048	-1.107830
H	0.403008	-5.674306	-3.317124
H	2.862438	-5.389140	-3.150564

Aryl Substituent: 4-OMe Intermediate 14 Conformer 5

C	8.449910	-0.329535	-0.389919
C	7.971602	-1.135192	-1.585597
O	6.558144	-1.486895	-1.447340
C	6.266050	-2.600261	-0.774196
O	7.075817	-3.343494	-0.252323
C	4.753289	-2.836010	-0.752083
C	4.230405	-3.151303	-2.190189
O	4.974932	-4.216162	-2.758516
C	2.719991	-3.382412	-2.215480
C	1.837352	-2.443868	-1.667261
C	0.453063	-2.631013	-1.705962
C	-0.072964	-3.782916	-2.307014
O	-1.403848	-4.070718	-2.397902
C	-2.335732	-3.140281	-1.843148
C	0.800567	-4.726503	-2.868495
C	2.176377	-4.523205	-2.823460
C	4.465731	-3.975248	0.210609
O	4.434406	-5.146190	-0.098112
C	4.170992	-3.566840	1.673367
F	5.114020	-2.715803	2.122804
F	2.981865	-2.929523	1.715306
F	4.120883	-4.624727	2.484294
H	9.485791	-0.017247	-0.558270
H	8.415916	-0.926521	0.525623
H	7.837877	0.567005	-0.253449
H	8.544897	-2.055339	-1.713050
H	8.007413	-0.552707	-2.507509

H	4.283029	-1.916404	-0.391388
H	4.468464	-2.271994	-2.797883
H	4.869259	-4.983609	-2.171873
H	2.217844	-1.536387	-1.205183
H	-0.195840	-1.880174	-1.270832
H	-3.324423	-3.564449	-2.023087
H	-2.263609	-2.162652	-2.333764
H	-2.182386	-3.017075	-0.764726
H	0.383278	-5.612342	-3.338102
H	2.837438	-5.255407	-3.274792

Aryl Substituent: 4-OMe Intermediate 15 Conformer 0

C	2.886861	-0.143967	1.578264
C	3.492483	-0.651963	0.285290
O	3.877035	0.513070	-0.502543
C	4.447449	0.272145	-1.689395
O	4.678119	-0.842305	-2.125156
C	4.772436	1.546060	-2.414938
C	3.827497	2.430096	-2.859419
C	2.384481	2.372042	-2.841704
C	1.680885	3.569996	-3.096770
C	0.292534	3.625590	-3.088463
C	-0.439539	2.450388	-2.848876
O	-1.787559	2.377969	-2.826591
C	-2.547950	3.571837	-3.059525
C	0.241300	1.233618	-2.628831
C	1.621725	1.196584	-2.622047
C	6.176353	1.895656	-2.678796
O	6.545091	2.883836	-3.299061
C	7.291600	0.958074	-2.139333
F	7.032441	0.544961	-0.877682
F	7.412119	-0.134777	-2.918099
F	8.471565	1.590948	-2.127958
H	2.586348	-0.996202	2.195480
H	2.002011	0.469270	1.382786
H	3.610314	0.452230	2.142450
H	2.781276	-1.244887	-0.297938
H	4.385074	-1.260110	0.456971
H	4.240359	3.329557	-3.314185
H	2.242603	4.479506	-3.291689
H	-0.207049	4.568141	-3.275546
H	-3.593735	3.272276	-2.991924
H	-2.344114	3.978547	-4.055502
H	-2.331244	4.329311	-2.299235
H	-0.343084	0.331705	-2.477825

H	2.110318	0.237798	-2.494210

Aryl Substituent: 4-OMe Intermediate 15 Conformer 1

C	4.562415	-0.763556	1.467474
C	3.391134	-0.408035	0.566218
O	3.748383	0.679286	-0.340674
C	4.331292	0.347808	-1.500872
O	4.576029	-0.792567	-1.853557
C	4.655541	1.565405	-2.318183
C	3.712861	2.398017	-2.856480
C	2.270417	2.327924	-2.870878
C	1.567494	3.488742	-3.263304
C	0.179316	3.533360	-3.299614
C	-0.552906	2.382328	-2.963091
O	-1.900575	2.301851	-2.970505
C	-2.659937	3.460026	-3.344730
C	0.128069	1.199666	-2.602497
C	1.507923	1.174451	-2.554617
C	6.061663	1.911456	-2.575814
O	6.434725	2.858746	-3.254525
C	7.175646	1.022262	-1.956521
F	6.898845	0.684283	-0.675861
F	7.322678	-0.114056	-2.665367
F	8.348559	1.668399	-1.962455
H	4.251099	-1.536688	2.177541
H	4.896360	0.110877	2.033981
H	5.403137	-1.148128	0.884089
H	2.548297	-0.012382	1.135736
H	3.060561	-1.264306	-0.025243
H	4.129262	3.260224	-3.375595
H	2.129532	4.378841	-3.532655
H	-0.319837	4.448116	-3.594663
H	-3.705761	3.161251	-3.274386
H	-2.431020	3.763846	-4.371497
H	-2.467267	4.293026	-2.660715
H	-0.455450	0.313075	-2.376072
H	1.997113	0.238050	-2.314666

Aryl Substituent: 4-OMe Intermediate 15 Conformer 2

C	2.861060	-0.139423	1.561528
C	3.474688	-0.660068	0.277406
O	3.864321	0.497051	-0.519485
C	4.438378	0.244043	-1.702165
O	4.669966	-0.874845	-2.125795
C	4.766075	1.510569	-2.439295
C	3.823081	2.394193	-2.888851

C	2.379938	2.340276	-2.868367
C	1.679124	3.539312	-3.125901
C	0.290944	3.598582	-3.115068
C	-0.443736	2.425979	-2.870749
O	-1.791927	2.357229	-2.845779
C	-2.549529	3.552650	-3.079843
C	0.234292	1.208013	-2.648675
C	1.614661	1.167335	-2.644175
C	6.170511	1.853453	-2.709654
O	6.540493	2.834090	-3.341030
C	7.284741	0.919283	-2.162128
F	7.026707	0.519668	-0.895998
F	7.402554	-0.181554	-2.929975
F	8.465822	1.550285	-2.158878
H	2.557303	-0.985617	2.185443
H	1.977042	0.471206	1.354473
H	3.580749	0.462838	2.124077
H	2.767078	-1.258595	-0.304431
H	4.366125	-1.266561	0.460520
H	4.237863	3.289003	-3.350984
H	2.242867	4.446767	-3.324528
H	-0.206527	4.541887	-3.303950
H	-3.595980	3.256139	-3.009177
H	-2.346824	3.956463	-4.077235
H	-2.329014	4.311342	-2.321859
H	-0.352209	0.308036	-2.494349
H	2.101262	0.207671	-2.514922

Aryl Substituent: 4-OMe Intermediate 15 Conformer 3

C	2.354323	-1.688673	-0.319979
C	3.690140	-1.077740	0.069406
O	3.942395	0.179938	-0.630963
C	4.513072	0.108872	-1.841204
O	4.792621	-0.928797	-2.415294
C	4.805671	1.475893	-2.394591
C	3.852103	2.390765	-2.747069
C	2.410996	2.303439	-2.781072
C	1.683967	3.510353	-2.878166
C	0.294850	3.532578	-2.903330
C	-0.411953	2.318702	-2.861539
O	-1.758003	2.214305	-2.889457
C	-2.542690	3.412621	-2.966802
C	0.294581	1.098210	-2.804276
C	1.675192	1.092213	-2.761566
C	6.206469	1.892510	-2.571836

O	6.560679	2.956943	-3.060158
C	7.337732	0.931153	-2.112723
F	7.055929	0.356315	-0.921211
F	7.522323	-0.051538	-3.015660
F	8.492721	1.596046	-1.982197
H	2.171017	-2.569665	0.304044
H	2.352021	-2.006831	-1.366115
H	1.536897	-0.979568	-0.160065
H	4.519777	-1.757511	-0.135688
H	3.706809	-0.793636	1.123059
H	4.256224	3.350803	-3.065319
H	2.226534	4.450904	-2.919877
H	-0.225079	4.480726	-2.964325
H	-3.581788	3.083717	-2.975348
H	-2.323264	3.966267	-3.885572
H	-2.365891	4.054271	-2.097386
H	-0.269759	0.171212	-2.807232
H	2.189482	0.138801	-2.761487

Aryl Substituent: 4-OMe Intermediate 15 Conformer 4

C	4.364586	-0.730964	1.465295
C	3.245157	-0.380425	0.498433
O	3.651332	0.703925	-0.390987
C	4.298184	0.367658	-1.515473
O	4.563428	-0.774222	-1.848112
C	4.666531	1.580422	-2.320741
C	3.755021	2.418993	-2.901999
C	2.314200	2.357810	-2.980433
C	1.635320	3.525145	-3.395029
C	0.250223	3.577930	-3.490833
C	-0.502137	2.428763	-3.194736
O	-1.848628	2.356068	-3.261071
C	-2.584894	3.521445	-3.658617
C	0.156248	1.239630	-2.813775
C	1.532552	1.206446	-2.705896
C	6.085381	1.915711	-2.514863
O	6.496002	2.852136	-3.187073
C	7.161903	1.032662	-1.824408
F	6.818151	0.726351	-0.551740
F	7.335708	-0.121045	-2.497472
F	8.338065	1.671506	-1.786375
H	4.015700	-1.503851	2.157957
H	4.663400	0.145300	2.048358
H	5.238086	-1.113865	0.931067
H	2.371128	0.015690	1.018439

H	2.949670	-1.239633	-0.107201
H	4.200041	3.278116	-3.402179
H	2.213375	4.413836	-3.633435
H	-0.230878	4.497471	-3.800653
H	-3.634331	3.227939	-3.636465
H	-2.309623	3.832228	-4.671805
H	-2.417943	4.347815	-2.959937
H	-0.441330	0.354700	-2.619858
H	2.006472	0.265809	-2.452080

Aryl Substituent: 4-OMe Intermediate 16 Conformer 1

C	8.839675	-0.492934	-3.127410
C	8.154524	-0.459251	-1.772538
O	6.746332	-0.844405	-1.892915
C	6.455090	-2.142008	-1.836921
O	7.251325	-3.044228	-1.659145
C	4.946718	-2.365502	-2.026139
C	4.455210	-1.820933	-3.402027
N	5.195151	-2.479462	-4.479078
C	5.413206	-1.968565	-5.712735
N	4.955770	-0.733338	-5.975898
S	6.279853	-2.869891	-6.884550
C	2.936192	-1.891421	-3.543643
C	2.150675	-0.869928	-2.998366
C	0.756141	-0.901623	-3.073911
C	0.125249	-1.976938	-3.714958
O	-1.223093	-2.110949	-3.853509
C	-2.065512	-1.094387	-3.304754
C	0.904191	-3.004282	-4.273117
C	2.290696	-2.960170	-4.185646
C	4.663645	-3.847011	-1.813922
O	4.726813	-4.693992	-2.675313
C	4.257480	-4.248847	-0.376017
F	5.123845	-3.732352	0.516944
F	3.035233	-3.741926	-0.112677
F	4.216912	-5.572918	-0.225694
H	9.867382	-0.131501	-3.018449
H	8.321697	0.152789	-3.842697
H	8.874744	-1.510528	-3.526448
H	8.113763	0.550509	-1.361757
H	8.632578	-1.127297	-1.054075
H	4.432325	-1.784941	-1.254027
H	4.752385	-0.767545	-3.393098
H	5.430309	-3.456510	-4.359075
H	4.299005	-0.251340	-5.378092

H	5.104095	-0.354082	-6.898818
H	2.627853	-0.025467	-2.505560
H	0.181555	-0.090243	-2.643363
H	-3.087887	-1.402340	-3.526925
H	-1.868463	-0.120770	-3.767949
H	-1.935832	-1.013424	-2.219477
H	0.403432	-3.827505	-4.773505
H	2.873539	-3.761343	-4.629349

Aryl Substituent: 4-OMe Intermediate 16 Conformer 2

C	8.824283	-0.583456	-3.070099
C	8.083636	-0.424383	-1.754011
O	6.669214	-0.774990	-1.906011
C	6.334499	-2.056155	-1.771121
O	7.094391	-2.966638	-1.500360
C	4.827463	-2.248314	-2.002786
C	4.409382	-1.813846	-3.440493
N	5.155631	-2.598798	-4.425192
C	5.415809	-2.221008	-5.698037
N	4.993315	-1.009755	-6.096143
S	6.289821	-3.254295	-6.749518
C	2.894800	-1.831849	-3.634931
C	2.144058	-0.714089	-3.255295
C	0.753061	-0.690442	-3.384075
C	0.091240	-1.808280	-3.910210
O	-1.256389	-1.896952	-4.086598
C	-2.064650	-0.779933	-3.706886
C	0.835807	-2.933607	-4.302296
C	2.218633	-2.943024	-4.164162
C	4.486831	-3.694728	-1.668624
O	4.545940	-4.618196	-2.447923
C	4.025357	-3.951039	-0.214124
F	4.872371	-3.369650	0.657179
F	2.806860	-3.397344	-0.043878
F	3.949470	-5.253920	0.057912
H	9.857752	-0.245333	-2.942320
H	8.359190	0.020114	-3.855111
H	8.843024	-1.629420	-3.388647
H	8.058936	0.615083	-1.424014
H	8.508284	-1.048339	-0.965686
H	4.303833	-1.583455	-1.308666
H	4.752495	-0.776920	-3.516671
H	5.360839	-3.564105	-4.201103
H	4.331035	-0.458612	-5.568122
H	5.174204	-0.727532	-7.047596

H	2.646637	0.162574	-2.852253
H	0.206221	0.195180	-3.083383
H	-3.092784	-1.066625	-3.930862
H	-1.801293	0.115217	-4.281771
H	-1.969603	-0.566240	-2.636147
H	0.311183	-3.789523	-4.715826
H	2.775216	-3.819978	-4.479840

Aryl Substituent: 4-OMe Intermediate 16 Conformer 3

C	2.874232	-1.651808	2.231832
C	4.142290	-1.103280	1.601470
O	4.477502	-1.834034	0.377645
C	3.910039	-1.427859	-0.765491
O	3.140197	-0.498534	-0.876748
C	4.392795	-2.318115	-1.916106
C	4.101532	-1.677483	-3.306070
N	5.001677	-2.323293	-4.277644
C	5.413886	-1.796617	-5.460701
N	5.114967	-0.517333	-5.725653
S	6.325186	-2.748540	-6.551841
C	2.637140	-1.764854	-3.711183
C	1.861514	-0.610350	-3.839657
C	0.523472	-0.670760	-4.241431
C	-0.057079	-1.914874	-4.518969
O	-1.347008	-2.096130	-4.916897
C	-2.177889	-0.942512	-5.068795
C	0.712925	-3.084789	-4.390914
C	2.040901	-3.005798	-3.994063
C	5.868936	-2.669378	-1.729614
O	6.284788	-3.801398	-1.637735
C	6.911562	-1.517445	-1.668272
F	6.321139	-0.301245	-1.674315
F	7.648679	-1.620198	-0.553658
F	7.732549	-1.585959	-2.728444
H	2.690727	-1.130773	3.177201
H	2.973051	-2.720995	2.441018
H	2.010418	-1.494511	1.580017
H	5.013118	-1.254778	2.240765
H	4.051151	-0.044044	1.353632
H	3.864512	-3.274332	-1.831289
H	4.389389	-0.626100	-3.235929
H	5.113344	-3.327813	-4.219358
H	4.494320	0.040559	-5.157151
H	5.435214	-0.116441	-6.594277
H	2.296389	0.359562	-3.612800

H	-0.046100	0.246406	-4.332260
H	-3.151416	-1.316412	-5.388079
H	-1.779427	-0.263126	-5.830994
H	-2.286556	-0.402728	-4.121129
H	0.250616	-4.042230	-4.610651
H	2.618119	-3.923560	-3.911261

Aryl Substituent: 4-OMe Intermediate 16 Conformer 4

C	8.906545	-0.980582	-3.357912
C	8.221107	-0.710673	-2.029926
O	6.785289	-0.986240	-2.121385
C	6.382328	-2.235440	-1.901082
O	7.096569	-3.170888	-1.593495
C	4.861153	-2.353911	-2.083982
C	4.423983	-1.955303	-3.526263
N	5.102674	-2.814770	-4.497549
C	5.355837	-2.497041	-5.788175
N	5.001116	-1.276038	-6.220456
S	6.136896	-3.619339	-6.821416
C	2.905452	-1.905963	-3.679965
C	2.219261	-0.741340	-3.319391
C	0.827844	-0.655091	-3.411640
C	0.099156	-1.756784	-3.880760
O	-1.255687	-1.786352	-4.017569
C	-1.999417	-0.620421	-3.654137
C	0.778437	-2.928860	-4.253445
C	2.162711	-3.000088	-4.152041
C	4.454375	-3.765680	-1.682055
O	4.445514	-4.721187	-2.424089
C	4.014492	-3.939700	-0.208866
F	4.903170	-3.358467	0.619896
F	2.823884	-3.327988	-0.039370
F	3.889914	-5.225445	0.120905
H	9.962189	-0.700876	-3.279838
H	8.452049	-0.390086	-4.158924
H	8.851543	-2.040464	-3.621752
H	8.266432	0.344036	-1.754998
H	8.636094	-1.317011	-1.222940
H	4.394600	-1.635023	-1.403085
H	4.815842	-0.941109	-3.652122
H	5.256530	-3.782270	-4.243558
H	4.398828	-0.658689	-5.694087
H	5.180383	-1.036835	-7.183902
H	2.773540	0.123381	-2.960738
H	0.332269	0.265463	-3.127355

H	-3.046103	-0.865137	-3.838575
H	-1.712403	0.241685	-4.267028
H	-1.862544	-0.375520	-2.594663
H	0.202740	-3.772070	-4.622781
H	2.667606	-3.913182	-4.451686

Aryl Substituent: 4-OMe Intermediate 16 Conformer 5

C	3.175054	-1.683547	2.284907
C	4.414243	-1.103131	1.626246
O	4.729546	-1.815861	0.386545
C	4.123061	-1.409956	-0.736632
O	3.337417	-0.491171	-0.818666
C	4.583691	-2.290014	-1.904616
C	4.223079	-1.668658	-3.285866
N	5.065080	-2.340899	-4.291204
C	5.384468	-1.856160	-5.519033
N	5.007016	-0.605488	-5.822417
S	6.282435	-2.814816	-6.614967
C	2.738404	-1.751383	-3.611917
C	1.964594	-0.592972	-3.714570
C	0.607288	-0.648688	-4.046593
C	0.005248	-1.892053	-4.277918
O	-1.304488	-2.069263	-4.606393
C	-2.134883	-0.911870	-4.730347
C	0.773450	-3.065750	-4.174708
C	2.120713	-2.991435	-3.848244
C	6.074781	-2.600248	-1.773042
O	6.524403	-3.719995	-1.690478
C	7.087123	-1.419850	-1.768085
F	6.465165	-0.221746	-1.697229
F	7.917657	-1.525031	-0.722446
F	7.817034	-1.442020	-2.895494
H	3.007706	-1.177109	3.241139
H	3.300571	-2.752881	2.478528
H	2.290535	-1.536174	1.659034
H	5.304999	-1.243390	2.240149
H	4.295471	-0.043678	1.391555
H	4.084389	-3.259302	-1.794853
H	4.521777	-0.617460	-3.253029
H	5.227854	-3.334664	-4.188502
H	4.346579	-0.078596	-5.268856
H	5.242513	-0.240158	-6.732932
H	2.416659	0.376442	-3.521065
H	0.039762	0.271317	-4.119613
H	-3.125585	-1.283031	-4.995335

H	-1.771354	-0.244521	-5.520109
H	-2.191880	-0.360083	-3.785089
H	0.294181	-4.022508	-4.358068
H	2.695344	-3.912296	-3.784185

Aryl Substituent: 4-OMe Intermediate 2 Conformer 0

H	2.564402	0.813660	-0.573389
C	2.120009	-0.031232	-0.006818
O	1.073911	-0.548478	-0.386281
C	2.880205	-0.436701	1.178824
C	2.442314	-1.491335	1.997588
C	3.165286	-1.873589	3.120943
C	4.355239	-1.191340	3.441131
O	5.135057	-1.480801	4.509306
C	4.742045	-2.546656	5.383024
C	4.803947	-0.132615	2.627570
C	4.071558	0.236667	1.510770
H	1.523324	-2.009492	1.739822
H	2.808642	-2.689412	3.737920
H	5.510078	-2.592939	6.155331
H	4.701315	-3.501010	4.847245
H	3.769628	-2.341329	5.843161
H	5.723844	0.377440	2.895338
H	4.419196	1.054162	0.883515

Aryl Substituent: 4-OMe Intermediate 2 Conformer 1

H	2.566405	0.811035	-0.575688
C	2.121072	-0.032590	-0.007982
O	1.074847	-0.549752	-0.387217
C	2.880220	-0.436679	1.178862
C	2.441006	-1.489697	1.998990
C	3.162989	-1.870685	3.123410
C	4.353217	-1.188797	3.443299
O	5.132193	-1.477156	4.512415
C	4.737374	-2.540851	5.387943
C	4.803227	-0.131700	2.628351
C	4.071843	0.236370	1.510491
H	1.521845	-2.007617	1.741427
H	2.805362	-2.685309	3.741401
H	5.504484	-2.586158	6.161227
H	4.696547	-3.496309	4.854130
H	3.764621	-2.333808	5.846591
H	5.723333	0.378101	2.895876
H	4.420516	1.052601	0.882172

Aryl Substituent: 4-OMe Intermediate 2 Conformer 2

H	2.414312	0.949690	-0.439230

C	2.049167	0.029883	0.063622
O	1.021656	-0.519616	-0.321242
C	2.885054	-0.429234	1.176786
C	2.550543	-1.580171	1.910081
C	3.345012	-2.012929	2.964695
C	4.502826	-1.284662	3.300965
O	5.346028	-1.616461	4.306843
C	5.057340	-2.778213	5.094941
C	4.847809	-0.129216	2.572630
C	4.045225	0.289681	1.523809
H	1.655268	-2.132452	1.640118
H	3.068591	-2.902831	3.517178
H	5.860434	-2.843389	5.829315
H	5.050668	-3.682504	4.477121
H	4.095768	-2.676277	5.609106
H	5.744888	0.414119	2.852135
H	4.311812	1.181881	0.962126

Aryl Substituent: 4-OMe Intermediate 2 Conformer 3

H	2.574901	0.803023	-0.583685
C	2.113830	-0.020142	0.001118
O	1.047138	-0.510798	-0.355743
C	2.878483	-0.432428	1.181607
C	2.420457	-1.461934	2.021035
C	3.147181	-1.850194	3.139852
C	4.361236	-1.199583	3.434441
O	5.146996	-1.497402	4.495945
C	4.733480	-2.537192	5.391522
C	4.830318	-0.166444	2.599729
C	4.093917	0.209259	1.487720
H	1.482937	-1.955686	1.782867
H	2.775804	-2.646536	3.773387
H	5.509203	-2.593741	6.155373
H	4.658150	-3.498235	4.871644
H	3.773030	-2.295836	5.859219
H	5.768674	0.318970	2.848672
H	4.456766	1.006799	0.843596

Aryl Substituent: 4-OMe Intermediate 2 Conformer 4

H	1.222023	-0.645514	-0.188648
C	2.125233	-0.026929	-0.005221
O	2.409091	0.895262	-0.763467
C	2.898536	-0.412392	1.178113
C	2.470448	-1.489156	1.968457
C	3.182185	-1.880625	3.100002
C	4.348540	-1.180412	3.451289

O	5.119130	-1.469611	4.526006
C	4.735081	-2.557401	5.376367
C	4.788833	-0.094974	2.662552
C	4.071579	0.281276	1.542360
H	1.567219	-2.029177	1.693667
H	2.832097	-2.715321	3.694786
H	5.490694	-2.599531	6.161002
H	4.723800	-3.503450	4.824591
H	3.750456	-2.380237	5.822127
H	5.693625	0.427194	2.957910
H	4.402432	1.114697	0.930006

Aryl Substituent: 4-OMe Intermediate 3 Conformer 0

H	2.688702	-1.018862	-0.226867
C	2.106356	-0.103227	-0.074266
O	0.753909	-0.532357	-0.232507
N	2.335220	0.398661	1.285511
C	2.171853	-0.334950	2.416261
N	1.497332	-1.490587	2.301312
S	2.816096	0.203427	3.909601
C	2.515436	0.942585	-1.087629
C	2.056076	2.262124	-0.998628
C	2.395901	3.214133	-1.962623
C	3.210715	2.843806	-3.042463
O	3.604429	3.687636	-4.036052
C	3.163142	5.047200	-3.987338
C	3.675797	1.521570	-3.141527
C	3.328988	0.586403	-2.173692
H	0.176717	0.243210	-0.146050
H	2.908827	1.224887	1.385176
H	0.929534	-1.653659	1.475097
H	1.296992	-2.011043	3.142086
H	1.425286	2.566127	-0.166566
H	2.026533	4.227668	-1.862525
H	3.587942	5.529872	-4.868236
H	3.525848	5.550734	-3.083994
H	2.069852	5.112129	-4.026416
H	4.308347	1.250827	-3.981355
H	3.695173	-0.433312	-2.261310

Aryl Substituent: 4-OMe Intermediate 3 Conformer 1

H	1.527322	-0.809919	-0.450275
C	1.885847	0.098003	0.051021
O	0.755151	0.549617	0.780710
N	2.980470	-0.280310	0.944962
C	2.927299	-1.294015	1.842665

N	1.728035	-1.875940	2.025609
S	4.332261	-1.811787	2.672164
C	2.391504	1.096156	-0.979679
C	1.698117	2.279409	-1.236644
C	2.146268	3.189580	-2.199983
C	3.307588	2.909076	-2.930116
O	3.835428	3.721030	-3.889407
C	3.169604	4.953231	-4.173132
C	4.004985	1.712389	-2.689773
C	3.548749	0.819352	-1.728725
H	1.025851	1.314455	1.314109
H	3.864049	0.203344	0.861844
H	0.885038	-1.373329	1.769446
H	1.654604	-2.595466	2.729298
H	0.789871	2.499990	-0.685744
H	1.584879	4.100703	-2.369395
H	3.757524	5.437487	-4.953852
H	3.134062	5.599713	-3.288571
H	2.150339	4.780796	-4.537771
H	4.898825	1.501582	-3.268948
H	4.097129	-0.104849	-1.565214

Aryl Substituent: 4-OMe Intermediate 3 Conformer 2

H	2.689715	-1.012783	-0.216263
C	2.102016	-0.099358	-0.071009
O	0.753065	-0.534475	-0.241812
N	2.315288	0.405937	1.289905
C	2.143040	-0.325771	2.420629
N	1.474406	-1.484359	2.301407
S	2.769645	0.218515	3.919185
C	2.516951	0.946206	-1.082300
C	2.051249	2.263991	-1.000663
C	2.397012	3.215510	-1.963030
C	3.224385	2.846565	-3.033744
O	3.624846	3.690025	-4.024947
C	3.178061	5.048038	-3.982995
C	3.695861	1.526079	-3.125387
C	3.342996	0.591362	-2.159305
H	0.171580	0.238459	-0.160746
H	2.884224	1.234841	1.393752
H	0.915887	-1.651586	1.469717
H	1.267326	-2.003589	3.141267
H	1.410608	2.567159	-0.175877
H	2.022316	4.227636	-1.868653
H	3.610257	5.530828	-4.860216

H	3.529538	5.554212	-3.076696
H	2.084999	5.108770	-4.033547
H	4.338159	1.256138	-3.958036
H	3.714372	-0.426953	-2.241235

Aryl Substituent: 4-OMe Intermediate 3 Conformer 3

H	1.433538	-0.822900	-0.501416
C	1.987613	-0.012698	-0.010415
O	1.061478	0.505627	0.931891
N	3.152669	-0.597366	0.654342
C	3.111089	-1.676288	1.473339
N	1.890388	-2.116473	1.829241
S	4.549555	-2.441444	1.997342
C	2.447147	1.003537	-1.044788
C	1.897234	2.284989	-1.090631
C	2.300411	3.214616	-2.055220
C	3.267628	2.854928	-3.001848
O	3.734131	3.676798	-3.984122
C	3.209389	5.004263	-4.061094
C	3.816998	1.561223	-2.973674
C	3.407952	0.649927	-2.008438
H	1.514564	1.180501	1.462807
H	4.069135	-0.230815	0.437033
H	1.096062	-1.488878	1.763571
H	1.836990	-2.881081	2.485712
H	1.136733	2.569161	-0.370846
H	1.854945	4.202296	-2.057669
H	3.715157	5.476686	-4.904060
H	3.419604	5.569094	-3.145545
H	2.128371	4.995333	-4.242070
H	4.559233	1.290848	-3.718551
H	3.837887	-0.348484	-2.010491

Aryl Substituent: 4-OMe Intermediate 3 Conformer 4

H	1.771535	-0.960720	-0.629920
C	2.107177	-0.064609	-0.092596
O	0.982994	0.298491	0.693740
N	3.245517	-0.440345	0.746772
C	3.264059	-1.499022	1.592015
N	2.097630	-2.144602	1.776686
S	4.714253	-1.993660	2.354852
C	2.536434	1.007133	-1.083098
C	1.777963	2.164535	-1.262901
C	2.154689	3.143892	-2.188238
C	3.309403	2.960243	-2.958880
O	3.770971	3.845426	-3.887006

C	3.037446	5.055139	-4.090801
C	4.071482	1.790167	-2.796829
C	3.685662	0.827661	-1.872563
H	1.237462	1.047525	1.256892
H	4.104781	0.085320	0.662909
H	1.226082	-1.665983	1.575817
H	2.077504	-2.896531	2.449565
H	0.874742	2.310064	-0.679719
H	1.544442	4.032565	-2.297164
H	3.579552	5.607548	-4.859367
H	2.993912	5.653717	-3.173629
H	2.018959	4.849554	-4.439891
H	4.959569	1.654878	-3.406621
H	4.284181	-0.073886	-1.769924

Aryl Substituent: 4-OMe Intermediate 4 Conformer 0

H	2.479472	-1.065784	-0.103983
C	2.069753	-0.055371	0.012880
N	1.046002	0.152138	0.777429
C	0.432656	-0.926632	1.412833
N	-0.259212	-0.539873	2.488719
S	0.462035	-2.554282	0.906977
C	2.733836	1.001917	-0.719623
C	2.301911	2.342587	-0.666241
C	2.953239	3.335701	-1.384394
C	4.067184	2.999920	-2.179612
O	4.772193	3.888179	-2.915632
C	4.377092	5.266444	-2.903398
C	4.512094	1.664808	-2.241581
C	3.853115	0.684083	-1.520512
H	-0.256271	0.434479	2.762855
H	-0.762174	-1.211825	3.049764
H	1.445489	2.596052	-0.049633
H	2.600083	4.358041	-1.325156
H	5.081423	5.780276	-3.557816
H	4.440749	5.685387	-1.893689
H	3.359872	5.387656	-3.290292
H	5.372052	1.428959	-2.860091
H	4.199405	-0.344859	-1.571804

Aryl Substituent: 4-OMe Intermediate 4 Conformer 1

H	2.475814	-1.056066	-0.085261
C	2.060528	-0.046646	0.019405
N	1.025626	0.161655	0.768413
C	0.405737	-0.914512	1.400216
N	-0.284341	-0.528890	2.477851

S	0.424894	-2.539758	0.885730
C	2.730338	1.008491	-0.710983
C	2.290812	2.347367	-0.673654
C	2.947926	3.338265	-1.389326
C	4.075476	3.002294	-2.165305
O	4.787163	3.888741	-2.896971
C	4.385446	5.265381	-2.900352
C	4.527849	1.668920	-2.211051
C	3.862936	0.690224	-1.492888
H	-0.276589	0.443969	2.757143
H	-0.792724	-1.199994	3.034955
H	1.424083	2.600390	-0.071484
H	2.589562	4.359501	-1.343844
H	5.097433	5.777847	-3.547476
H	4.431729	5.691373	-1.892646
H	3.373734	5.378987	-3.303571
H	5.398188	1.433660	-2.815096
H	4.214180	-0.337599	-1.531205

Aryl Substituent: 4-OMe Intermediate 4 Conformer 2

H	2.467932	-1.070557	-0.131894
C	2.023752	-0.070474	-0.062421
N	0.937837	0.118211	0.616557
C	0.362812	-0.948638	1.304569
N	-0.936361	-0.743455	1.541989
S	1.162013	-2.347184	1.863127
C	2.708404	0.993838	-0.765033
C	2.247462	2.325945	-0.749254
C	2.926114	3.327806	-1.428354
C	4.095827	3.009823	-2.147591
O	4.829489	3.907646	-2.842504
C	4.407163	5.278080	-2.866377
C	4.568134	1.682799	-2.173793
C	3.881294	0.692996	-1.492468
H	-1.381730	0.099074	1.200917
H	-1.486816	-1.427581	2.040147
H	1.344731	2.563852	-0.195811
H	2.550770	4.343541	-1.401145
H	5.142532	5.801586	-3.477446
H	4.396483	5.704172	-1.857744
H	3.415038	5.375710	-3.319337
H	5.470356	1.461449	-2.734768
H	4.247864	-0.329939	-1.515064

Aryl Substituent: 4-OMe Intermediate 4 Conformer 3

H	2.461671	-1.060099	-0.099964

C	2.032024	-0.054221	-0.020994
N	0.964614	0.147327	0.685872
C	0.364498	-0.933243	1.339558
N	-0.684019	-0.532142	2.063449
S	0.822211	-2.575690	1.282242
C	2.711831	1.003518	-0.734801
C	2.258108	2.338657	-0.716927
C	2.929172	3.333487	-1.413012
C	4.084994	3.006090	-2.150763
O	4.810826	3.896665	-2.861932
C	4.394652	5.269193	-2.886558
C	4.551357	1.676689	-2.177755
C	3.872249	0.693917	-1.479411
H	-0.932080	0.449318	2.077691
H	-1.230395	-1.191653	2.598284
H	1.368100	2.583642	-0.146395
H	2.559956	4.351400	-1.383710
H	5.122323	5.785471	-3.512747
H	4.402935	5.700700	-1.880259
H	3.395621	5.368872	-3.323487
H	5.443101	1.448614	-2.752591
H	4.233651	-0.330810	-1.502690

Aryl Substituent: 4-OMe Intermediate 4 Conformer 4

H	2.372038	-1.079546	-0.197879
C	2.038358	-0.052416	-0.007831
N	1.036552	0.175815	0.779413
C	0.347422	-0.895764	1.344097
N	-0.293993	-0.541040	2.461368
S	0.236377	-2.475436	0.712355
C	2.774284	1.001844	-0.673175
C	2.440923	2.363371	-0.525333
C	3.158712	3.353352	-1.181538
C	4.240976	2.993186	-2.009327
O	5.003624	3.876442	-2.691978
C	4.705715	5.275263	-2.587409
C	4.588547	1.636995	-2.164282
C	3.863947	0.659645	-1.504072
H	-0.209753	0.405693	2.809313
H	-0.843506	-1.211869	2.978293
H	1.608226	2.634900	0.115648
H	2.881929	4.392307	-1.050038
H	5.437392	5.779597	-3.218769
H	4.809175	5.622700	-1.554147
H	3.695439	5.491146	-2.950398

H	5.426444	1.383060	-2.805520
H	4.134670	-0.385845	-1.626693

Aryl Substituent: 4-OMe Intermediate 6 Conformer 1

C	8.837254	2.585817	1.885316
C	8.141463	1.592580	0.971225
O	6.706158	1.875531	0.893425
C	6.301700	2.762602	-0.013009
O	7.013316	3.338398	-0.813868
C	4.782735	2.975974	0.081749
C	4.375909	3.546074	1.475049
N	5.075743	4.810629	1.707224
C	5.362561	5.341094	2.918309
N	5.022525	4.639635	4.012671
S	6.170641	6.847414	3.036733
C	2.861366	3.620554	1.654204
C	2.167525	2.485606	2.087846
C	0.778448	2.491737	2.237562
C	0.060331	3.661296	1.952486
O	-1.291212	3.785735	2.065470
C	-2.043002	2.649687	2.500004
C	0.747613	4.809718	1.524842
C	2.129310	4.786372	1.377284
C	4.361421	3.850698	-1.091267
O	4.355760	5.060389	-1.086583
C	3.907346	3.088990	-2.359580
F	4.818848	2.156260	-2.696964
F	2.744219	2.458364	-2.094132
F	3.725902	3.912285	-3.392142
H	9.891504	2.306629	1.981807
H	8.386864	2.580258	2.882302
H	8.787176	3.599315	1.477571
H	8.184998	0.576452	1.365674
H	8.549598	1.610092	-0.040825
H	4.307354	1.994517	-0.009600
H	4.771232	2.814494	2.186954
H	5.221157	5.421106	0.913160
H	4.388688	3.852989	3.984648
H	5.216970	5.041791	4.917300
H	2.713971	1.573754	2.319226
H	0.276358	1.594653	2.579648
H	-3.085396	2.970032	2.514547
H	-1.743215	2.335618	3.506342
H	-1.928805	1.808302	1.806949
H	0.179991	5.711260	1.315487

H	2.640973	5.686899	1.052357

Aryl Substituent: 4-OMe Intermediate 6 Conformer 2

C	8.848025	2.482705	1.981588
C	8.184562	1.584652	0.952497
O	6.760287	1.903752	0.825366
C	6.419425	2.875348	-0.018135
O	7.182229	3.504785	-0.726281
C	4.901332	3.110746	0.016686
C	4.427427	3.557198	1.433624
N	5.133684	4.779320	1.820681
C	5.349207	5.198220	3.089257
N	4.933602	4.413336	4.095463
S	6.157708	6.679532	3.387398
C	2.906092	3.647061	1.534794
C	2.169635	2.488882	1.806064
C	0.774573	2.510287	1.877100
C	0.093576	3.719223	1.678045
O	-1.260203	3.861255	1.727332
C	-2.054809	2.701311	1.986721
C	0.823236	4.890337	1.414063
C	2.210779	4.851545	1.342519
C	4.556125	4.100216	-1.088054
O	4.570624	5.304082	-0.969354
C	4.154188	3.471652	-2.443752
F	5.061441	2.549746	-2.819629
F	2.964457	2.851590	-2.299497
F	4.046855	4.391994	-3.402379
H	9.891091	2.174583	2.107321
H	8.346223	2.402951	2.950453
H	8.836412	3.527731	1.659557
H	8.189259	0.538377	1.261160
H	8.645690	1.677367	-0.032330
H	4.415934	2.152127	-0.191513
H	4.769650	2.753144	2.093216
H	5.332860	5.456195	1.095076
H	4.335186	3.610738	3.960130
H	5.094080	4.718645	5.043345
H	2.686766	1.545839	1.970206
H	0.238932	1.593696	2.093411
H	-3.091168	3.040835	1.974096
H	-1.823241	2.271497	2.967968
H	-1.910805	1.939268	1.212206
H	0.283595	5.821314	1.269556
H	2.755078	5.769319	1.143226

Aryl Substituent: 4-OMe Intermediate 6 Conformer 3

C	1.980777	-0.005613	-1.759414
C	3.482120	0.142451	-1.936782
O	3.932690	1.463546	-1.494162
C	4.215230	1.612762	-0.199794
O	4.126460	0.747174	0.648444
C	4.708236	3.038687	0.076986
C	4.343025	3.466720	1.532955
N	5.072832	4.704830	1.831024
C	5.301547	5.210202	3.067783
N	4.882391	4.498085	4.125434
S	6.139035	6.691971	3.254984
C	2.835593	3.594480	1.716685
C	2.121400	2.619643	2.419393
C	0.736923	2.714515	2.593662
C	0.047688	3.806677	2.051928
O	-1.295127	4.004763	2.158255
C	-2.069260	3.034717	2.868904
C	0.756408	4.793967	1.343079
C	2.130369	4.685847	1.180125
C	6.208310	3.182847	-0.202532
O	6.667867	4.109014	-0.828975
C	7.178194	2.100735	0.344223
F	6.943723	1.854786	1.648733
F	6.998153	0.950127	-0.338091
F	8.448122	2.482362	0.204269
H	1.665866	-0.972963	-2.164152
H	1.445032	0.783252	-2.295567
H	1.703640	0.030648	-0.702259
H	3.772075	0.108084	-2.987903
H	4.036427	-0.615050	-1.379447
H	4.227615	3.716726	-0.632786
H	4.716025	2.683225	2.197157
H	5.296092	5.334361	1.071158
H	4.239652	3.722453	4.043424
H	5.036191	4.875609	5.048414
H	2.645775	1.762987	2.834156
H	0.217172	1.942352	3.148068
H	-3.100120	3.388376	2.828348
H	-1.749429	2.959571	3.914427
H	-2.002188	2.048558	2.395537
H	0.208879	5.636865	0.932581
H	2.659988	5.465253	0.638358

Aryl Substituent: 4-OMe Intermediate 6 Conformer 4

C	8.898967	3.078244	1.839091
C	8.258905	1.993867	0.990310
O	6.807976	2.182876	0.915278
C	6.340029	2.991694	-0.032703
O	7.006051	3.563398	-0.874836
C	4.812229	3.118828	0.072681
C	4.385510	3.720210	1.446372
N	5.011567	5.031867	1.619633
C	5.286230	5.623749	2.804800
N	5.002266	4.947202	3.930264
S	6.008516	7.176688	2.853051
C	2.870810	3.712430	1.639627
C	2.248892	2.552971	2.115444
C	0.863377	2.482574	2.280437
C	0.075120	3.598752	1.968146
O	-1.280355	3.647199	2.092741
C	-1.959581	2.482038	2.567753
C	0.689640	4.771504	1.498035
C	2.068904	4.824153	1.335507
C	4.327211	3.918243	-1.129524
O	4.249902	5.124782	-1.173286
C	3.902630	3.079810	-2.358920
F	4.855934	2.178331	-2.663669
F	2.773566	2.406305	-2.054567
F	3.673258	3.846686	-3.424951
H	9.969487	2.870962	1.937861
H	8.458624	3.100783	2.840317
H	8.781722	4.062186	1.376383
H	8.369271	1.006163	1.440053
H	8.655807	1.979513	-0.026189
H	4.395662	2.107785	0.026748
H	4.829838	3.043070	2.182656
H	5.111057	5.617412	0.800283
H	4.418948	4.122058	3.942700
H	5.191516	5.391558	4.815996
H	2.850172	1.682277	2.368391
H	0.417967	1.568899	2.655429
H	-3.019065	2.740316	2.583812
H	-1.633113	2.216131	3.579653
H	-1.801655	1.628835	1.898152
H	0.068085	5.631424	1.267792
H	2.523523	5.742530	0.977350

Aryl Substituent: 4-OMe Intermediate 6 Conformer 5

C	2.128681	0.111568	-2.135907

C	3.640012	0.260870	-2.167120
O	4.050086	1.558101	-1.627012
C	4.218759	1.648093	-0.306960
O	4.049294	0.747692	0.490886
C	4.700341	3.054762	0.070230
C	4.292686	3.401514	1.534920
N	5.035118	4.605128	1.930195
C	5.238709	5.027105	3.202634
N	4.803107	4.246022	4.202693
S	6.066351	6.495720	3.503655
C	2.783111	3.549139	1.678687
C	2.030814	2.558536	2.316100
C	0.644105	2.674040	2.457439
C	-0.008396	3.803713	1.948077
O	-1.349648	4.024838	2.027955
C	-2.161619	3.040647	2.673720
C	0.738675	4.806744	1.303704
C	2.114424	4.677646	1.173116
C	6.208134	3.208288	-0.159944
O	6.686497	4.147029	-0.752394
C	7.161360	2.111419	0.388080
F	6.890756	1.831252	1.678756
F	6.998408	0.978307	-0.327857
F	8.435113	2.493563	0.293411
H	1.853476	-0.834483	-2.613267
H	1.644825	0.926870	-2.681778
H	1.754704	0.099439	-1.108433
H	4.025299	0.271089	-3.187746
H	4.139775	-0.523765	-1.595741
H	4.242787	3.772664	-0.615493
H	4.628869	2.573000	2.162637
H	5.256183	5.292211	1.221085
H	4.178179	3.465361	4.057792
H	4.942963	4.558216	5.151838
H	2.526795	1.673105	2.704464
H	0.094143	1.888303	2.961421
H	-3.183868	3.417121	2.621957
H	-1.871809	2.912496	3.722870
H	-2.100978	2.075144	2.158709
H	0.219392	5.678293	0.916994
H	2.673987	5.469202	0.681226

Aryl Substituent: 4-OMe Intermediate 7 Conformer 0

H	1.054260	0.199023	-0.001797
C	2.141470	0.057694	0.022821

N	2.765337	0.961974	-0.951994
C	2.907703	0.715976	-2.263532
S	3.508545	1.888736	-3.345665
N	2.580750	-0.529519	-2.702061
C	1.954187	-1.552034	-1.898774
O	0.557227	-1.455876	-2.024879
C	2.433638	-1.397429	-0.431002
H	3.507855	-1.583732	-0.386969
C	1.713196	-2.367248	0.496417
O	0.492517	-2.479240	0.516234
O	2.552210	-3.042625	1.266411
C	1.987132	-3.993116	2.229962
C	1.641272	-3.298358	3.535077
C	2.410885	-2.912418	-2.488502
F	2.121024	-2.976264	-3.802592
F	3.735880	-3.104894	-2.346678
F	1.777440	-3.931730	-1.873497
C	2.638466	0.358298	1.422330
C	1.726202	0.631788	2.444192
C	2.152615	0.894607	3.749786
C	3.522802	0.890393	4.040170
O	4.053413	1.136606	5.269798
C	3.159611	1.421007	6.349267
C	4.450741	0.623297	3.016914
C	4.011034	0.359599	1.727122
H	2.992572	1.898109	-0.643350
H	2.595598	-0.665450	-3.704784
H	0.157812	-1.873570	-1.233266
H	2.781081	-4.729772	2.358889
H	1.117009	-4.468151	1.773686
H	2.515450	-2.787599	3.948714
H	1.303145	-4.047302	4.258529
H	0.839476	-2.568715	3.394345
H	0.661371	0.644570	2.226012
H	1.417310	1.103751	4.517307
H	3.790723	1.585302	7.223380
H	2.484384	0.578767	6.538804
H	2.569605	2.322774	6.149788
H	5.509506	0.629177	3.257160
H	4.742545	0.160595	0.948226

Aryl Substituent: 4-OMe Intermediate 7 Conformer 1

H	1.073428	0.232564	-0.033206
C	2.158467	0.077782	0.004501
N	2.803501	0.956374	-0.979895

C	2.955379	0.685470	-2.285467
S	3.582121	1.830990	-3.381962
N	2.615979	-0.563105	-2.705301
C	1.968868	-1.563423	-1.890267
O	0.574563	-1.452138	-2.031852
C	2.436065	-1.388726	-0.420656
H	3.507271	-1.588019	-0.362517
C	1.694356	-2.333185	0.516177
O	0.472142	-2.429071	0.526319
O	2.517122	-3.006846	1.304922
C	1.930352	-3.934639	2.277682
C	1.576622	-3.215030	3.567083
C	2.414025	-2.939698	-2.451174
F	2.135797	-3.023545	-3.766631
F	3.735056	-3.146104	-2.293062
F	1.761959	-3.939799	-1.824248
C	2.646381	0.396969	1.403071
C	1.728386	0.698753	2.411801
C	2.146186	0.979269	3.716420
C	3.513607	0.964288	4.019343
O	4.036097	1.225587	5.249295
C	3.136258	1.539678	6.315427
C	4.447358	0.668737	3.009272
C	4.016071	0.387613	1.720257
H	3.038599	1.895231	-0.685623
H	2.640849	-0.717517	-3.705185
H	0.162292	-1.850790	-1.237015
H	2.714827	-4.677295	2.428316
H	1.060631	-4.408061	1.818907
H	2.451601	-2.708049	3.983664
H	1.220978	-3.948747	4.297749
H	0.785038	-2.478682	3.405193
H	0.665748	0.720077	2.183733
H	1.406601	1.210185	4.473553
H	3.761577	1.711530	7.192263
H	2.448888	0.709318	6.513544
H	2.559347	2.445000	6.094839
H	5.503960	0.666501	3.258971
H	4.752047	0.166655	0.951559

Aryl Substituent: 4-OMe Intermediate 7 Conformer 2

H	1.072319	0.196993	0.022941
C	2.157832	0.043319	0.045077
N	2.791763	0.956189	-0.915070
C	2.933263	0.729532	-2.230192

S	3.550220	1.912036	-3.292439
N	2.591256	-0.504477	-2.689069
C	1.952722	-1.532646	-1.902458
O	0.557013	-1.417885	-2.026553
C	2.433758	-1.407404	-0.432382
H	3.505793	-1.606517	-0.391395
C	1.702334	-2.384425	0.478962
O	0.480669	-2.485619	0.495014
O	2.533585	-3.079280	1.239973
C	1.957636	-4.038020	2.188786
C	1.607281	-3.358040	3.500489
C	2.393339	-2.888425	-2.514678
F	2.104575	-2.926090	-3.830050
F	3.715567	-3.099869	-2.374419
F	1.746324	-3.910160	-1.918229
C	2.658144	0.316077	1.449058
C	1.748975	0.584994	2.475010
C	2.178224	0.822873	3.784409
C	3.548218	0.797652	4.074860
O	4.081567	1.018595	5.308063
C	3.191074	1.294809	6.392409
C	4.473065	0.535077	3.047661
C	4.030496	0.296255	1.753969
H	3.030414	1.884385	-0.591302
H	2.608096	-0.625123	-3.693724
H	0.152884	-1.845239	-1.242476
H	2.747170	-4.780107	2.313517
H	1.087938	-4.503139	1.721507
H	2.481972	-2.858899	3.926996
H	1.259142	-4.114465	4.211334
H	0.811261	-2.621347	3.364118
H	0.684380	0.613906	2.256977
H	1.445272	1.029351	4.554948
H	3.824101	1.437138	7.268993
H	2.506398	0.457158	6.567839
H	2.611310	2.206446	6.208353
H	5.531800	0.524575	3.287924
H	4.759851	0.100417	0.972247

Aryl Substituent: 4-OMe Intermediate 7 Conformer 3

H	1.080604	0.276789	-0.043338
C	2.161768	0.098050	-0.000955
N	2.829385	0.952295	-0.990833
C	2.994544	0.661418	-2.290607
S	3.656331	1.781781	-3.392289

N	2.639568	-0.586540	-2.699673
C	1.954187	-1.558741	-1.882273
O	0.565481	-1.410157	-2.042829
C	2.408187	-1.378823	-0.409087
H	3.473340	-1.603813	-0.335434
C	1.630785	-2.294979	0.525812
O	0.406097	-2.352424	0.525286
O	2.424859	-2.985660	1.329229
C	1.799010	-3.914827	2.275687
C	1.594069	-5.273558	1.628829
C	2.367187	-2.953954	-2.420821
F	2.101945	-3.047024	-3.738125
F	3.679476	-3.195674	-2.243873
F	1.679255	-3.928080	-1.789448
C	2.653203	0.414875	1.397237
C	1.736766	0.677275	2.418255
C	2.159346	0.945990	3.723809
C	3.529281	0.959211	4.015315
O	4.055256	1.212198	5.245584
C	3.156522	1.485028	6.323973
C	4.461153	0.702418	2.993029
C	4.025176	0.432218	1.703642
H	3.079094	1.890518	-0.706883
H	2.672730	-0.752581	-3.697492
H	0.131910	-1.783105	-1.246969
H	0.860788	-3.477132	2.620384
H	2.508631	-3.959394	3.102713
H	0.893171	-5.210623	0.791972
H	1.182373	-5.964385	2.371619
H	2.543021	-5.680555	1.267668
H	0.671901	0.676522	2.199762
H	1.420897	1.146543	4.490649
H	3.784299	1.656219	7.199176
H	2.491004	0.634789	6.511976
H	2.556098	2.379892	6.124375
H	5.519610	0.720844	3.233950
H	4.758991	0.240057	0.925201

Aryl Substituent: 4-OMe Intermediate 7 Conformer 4

H	1.050603	0.123148	-0.036000
C	2.140913	0.006018	-0.026941
N	2.733138	0.947375	-0.986429
C	2.863923	0.737143	-2.305498
S	3.426157	1.949040	-3.364964
N	2.557192	-0.503594	-2.770911

C	1.963606	-1.559068	-1.985289
O	0.563330	-1.489026	-2.090019
C	2.459613	-1.430465	-0.520412
H	3.538094	-1.593732	-0.495126
C	1.773497	-2.439410	0.391171
O	0.556032	-2.579233	0.424564
O	2.638123	-3.115697	1.131719
C	2.107694	-4.102043	2.078910
C	1.770174	-3.448241	3.407122
C	2.440370	-2.894413	-2.615149
F	2.137764	-2.929830	-3.927442
F	3.770320	-3.064255	-2.491812
F	1.833548	-3.941658	-2.020748
C	2.647664	0.284086	1.373654
C	1.741600	0.518214	2.410777
C	2.176995	0.759660	3.717444
C	3.550046	0.773657	3.993760
O	4.089056	1.001452	5.223227
C	3.201642	1.242399	6.318478
C	4.471733	0.546670	2.955209
C	4.023144	0.303787	1.664362
H	2.942330	1.880942	-0.657908
H	2.566208	-0.615850	-3.776620
H	0.183888	-1.937382	-1.305462
H	2.918186	-4.825467	2.175917
H	1.239798	-4.582678	1.624353
H	2.643375	-2.939197	3.824830
H	1.449313	-4.221420	4.112788
H	0.958252	-2.724597	3.296901
H	0.674449	0.516469	2.203792
H	1.446267	0.938363	4.496957
H	3.838900	1.397143	7.189879
H	2.544370	0.383090	6.493990
H	2.592747	2.137414	6.147429
H	5.532722	0.566527	3.184660
H	4.749924	0.135760	0.873837

No Aryl Substituent (Benzaldehyde) Intermediate 12 Conformer 0

H	1.253003	0.635142	0.071169
C	2.310998	0.362083	0.028944
C	2.424728	-1.171440	0.309269
O	3.742597	-1.632025	0.067134
C	1.880273	-1.551234	1.686489
C	0.568219	-1.206892	2.047539
C	0.050962	-1.580089	3.288792

C	0.837875	-2.306344	4.188421
C	2.140609	-2.659562	3.832455
C	2.657350	-2.286459	2.588018
C	2.794797	0.668648	-1.395269
O	3.804663	1.293806	-1.656700
O	1.959215	0.142480	-2.293797
C	2.294156	0.315387	-3.706584
C	1.750365	1.631276	-4.236259
C	3.094734	1.223372	0.997662
N	4.195095	0.818980	1.480345
C	5.058490	1.464011	2.367760
N	6.090049	2.061072	1.776070
S	4.853302	1.311381	4.032760
C	2.449526	2.576456	1.320730
F	1.930750	3.121109	0.201573
F	1.439209	2.386895	2.192859
F	3.306872	3.458439	1.851386
H	1.802286	-1.655136	-0.451054
H	4.335627	-1.106313	0.631692
H	-0.063349	-0.655435	1.355233
H	-0.967524	-1.306843	3.551188
H	0.435667	-2.597273	5.154946
H	2.758721	-3.229348	4.521162
H	3.664522	-2.577667	2.309817
H	1.826885	-0.541696	-4.194214
H	3.378438	0.249878	-3.814917
H	0.668833	1.696016	-4.084007
H	1.951667	1.697711	-5.310524
H	2.230405	2.481627	-3.743952
H	6.173835	2.091533	0.767486
H	6.828149	2.463454	2.337379

No Aryl Substituent (Benzaldehyde) Intermediate 12 Conformer 1

H	1.154378	0.401477	0.058995
C	2.231070	0.217106	0.015340
C	2.475622	-1.289747	0.352232
O	3.824311	-1.648561	0.108523
C	1.981429	-1.658783	1.751348
C	0.653710	-1.398415	2.125244
C	0.185496	-1.765595	3.387609
C	1.037964	-2.401356	4.295935
C	2.357287	-2.670513	3.927820
C	2.824913	-2.303747	2.662317
C	2.675860	0.506534	-1.424996
O	3.634564	1.192544	-1.723143

O	1.870018	-0.111148	-2.292282
C	2.175480	0.032969	-3.714913
C	1.537137	1.288895	-4.283299
C	2.946242	1.176136	0.944162
N	4.089515	0.892905	1.413311
C	4.903405	1.640881	2.265680
N	5.857556	2.323375	1.637646
S	4.749433	1.506726	3.937725
C	2.179833	2.470125	1.241989
F	1.613104	2.939896	0.111979
F	1.191064	2.207092	2.119739
F	2.951990	3.438542	1.751867
H	1.886975	-1.853189	-0.379624
H	4.378264	-1.051362	0.640809
H	-0.027615	-0.917712	1.427424
H	-0.845905	-1.558321	3.659824
H	0.673759	-2.687465	5.278833
H	3.026801	-3.169406	4.623436
H	3.846227	-2.529491	2.374987
H	1.761589	-0.870794	-4.165008
H	3.260345	0.036885	-3.836539
H	0.455420	1.285462	-4.119171
H	1.721810	1.329771	-5.361844
H	1.963371	2.187142	-3.828003
H	5.916910	2.337836	0.626961
H	6.563267	2.810606	2.172789

No Aryl Substituent (Benzaldehyde) Intermediate 12 Conformer 2

H	1.195626	0.156834	-0.013880
C	2.279448	0.013984	-0.011158
C	2.569509	-1.455700	0.436603
O	3.938376	-1.775209	0.259698
C	2.044004	-1.746198	1.842632
C	0.692980	-1.524409	2.152640
C	0.197504	-1.819543	3.423448
C	1.045802	-2.343323	4.404326
C	2.388628	-2.573632	4.100418
C	2.883726	-2.279228	2.826469
C	2.761297	0.223420	-1.453582
O	3.698035	0.931280	-1.769759
O	2.014215	-0.490373	-2.299471
C	2.362050	-0.432368	-3.718488
C	1.690792	0.750634	-4.395061
C	2.925909	1.059848	0.873333
N	4.071386	0.861169	1.379254

C	4.832052	1.697488	2.198242
N	5.752193	2.400551	1.542594
S	4.668603	1.639628	3.873587
C	2.089578	2.328072	1.078087
F	1.556010	2.720481	-0.096622
F	1.073512	2.056705	1.921590
F	2.791841	3.353063	1.578062
H	2.026985	-2.091065	-0.271532
H	4.451135	-1.116837	0.760315
H	0.015837	-1.130537	1.398515
H	-0.851519	-1.643763	3.645943
H	0.660468	-2.573151	5.393916
H	3.055332	-2.985663	4.853266
H	3.924208	-2.474824	2.590388
H	2.002679	-1.383097	-4.115556
H	3.449345	-0.388449	-3.805989
H	0.605416	0.711374	-4.262736
H	1.908273	0.722494	-5.467873
H	2.064190	1.696920	-3.993961
H	5.820454	2.366582	0.532947
H	6.423922	2.953842	2.056894

No Aryl Substituent (Benzaldehyde) Intermediate 12 Conformer 3

H	1.203803	0.433004	0.007196
C	2.274848	0.213500	-0.007760
C	2.461944	-1.294158	0.361156
O	3.804749	-1.699951	0.163364
C	1.914938	-1.623028	1.750365
C	0.581265	-1.327418	2.073088
C	0.063436	-1.655799	3.326724
C	0.871615	-2.287772	4.277236
C	2.196470	-2.592280	3.959975
C	2.713791	-2.264188	2.703219
C	2.763179	0.462040	-1.441458
O	3.753573	1.107546	-1.726544
O	1.956607	-0.141288	-2.317877
C	2.301165	-0.033471	-3.734712
C	1.718349	1.231977	-4.340442
C	2.998996	1.167517	0.919032
N	4.109877	0.848035	1.439875
C	4.923664	1.585064	2.301952
N	5.934901	2.199623	1.693256
S	4.695566	1.515716	3.969377
C	2.281721	2.502715	1.152968
F	1.755638	2.953268	-0.004161

F	1.267171	2.313733	2.020312
F	3.084749	3.456633	1.642111
H	1.876741	-1.851049	-0.378507
H	4.361965	-1.113206	0.703773
H	-0.065730	-0.849459	1.341424
H	-0.971885	-1.421521	3.559245
H	0.468988	-2.543578	5.253473
H	2.831661	-3.088619	4.688813
H	3.739089	-2.517663	2.455990
H	1.869047	-0.931477	-4.179218
H	3.388211	-0.066742	-3.829434
H	0.633466	1.266684	-4.203012
H	1.930237	1.247521	-5.414620
H	2.162711	2.123981	-3.890182
H	6.035929	2.177067	0.686067
H	6.641356	2.670443	2.241936

No Aryl Substituent (Benzaldehyde) Intermediate 12 Conformer 4

H	1.162990	0.416526	0.026789
C	2.241056	0.239285	-0.007887
C	2.492529	-1.269168	0.318304
O	3.844003	-1.617747	0.075758
C	1.996914	-1.651380	1.713237
C	0.666798	-1.401184	2.085697
C	0.197651	-1.780167	3.344229
C	1.051567	-2.417563	4.250080
C	2.373324	-2.676387	3.883353
C	2.841976	-2.297838	2.621682
C	2.699834	0.538390	-1.441873
O	3.656489	1.232521	-1.727302
O	1.902917	-0.076323	-2.319660
C	2.238513	0.050339	-3.736709
C	3.240978	-1.012610	-4.152749
C	2.942654	1.193139	0.936213
N	4.092508	0.920244	1.395460
C	4.899398	1.667138	2.255556
N	5.828038	2.387749	1.631664
S	4.771529	1.487818	3.925344
C	2.154515	2.467681	1.258810
F	1.601529	2.962657	0.132864
F	1.154144	2.165818	2.110662
F	2.904010	3.430667	1.810958
H	1.909226	-1.829800	-0.419863
H	4.392725	-1.019615	0.612446
H	-0.015357	-0.919122	1.389677

H	-0.835571	-1.580779	3.615424
H	0.686630	-2.712841	5.229985
H	3.044010	-3.176352	4.577071
H	3.865305	-2.515643	2.335448
H	2.616772	1.059106	-3.912681
H	1.281330	-0.073458	-4.245847
H	4.192266	-0.882012	-3.629295
H	3.426310	-0.931693	-5.228890
H	2.855928	-2.015220	-3.944022
H	5.872973	2.427129	0.620877
H	6.530289	2.877697	2.168892

No Aryl Substituent (Benzaldehyde) Intermediate 13 Conformer 0

C	5.826343	1.255136	-0.955339
C	5.175458	0.023039	-1.563228
O	5.303193	-1.129994	-0.677969
C	4.373253	-1.291944	0.278898
O	3.398061	-0.576610	0.418099
C	4.722042	-2.470381	1.134675
C	3.818235	-3.317184	1.702232
C	2.367094	-3.441928	1.593260
C	1.590531	-2.974877	0.511620
C	0.214369	-3.182501	0.491398
C	-0.419223	-3.850010	1.546601
C	0.336652	-4.331024	2.619207
C	1.716715	-4.142374	2.633041
C	6.156385	-2.725370	1.410417
N	6.577710	-3.923474	1.544170
C	7.847291	-4.386350	1.842321
N	8.016058	-4.800289	3.097442
S	9.006426	-4.576618	0.622760
C	7.097556	-1.519698	1.619947
F	6.402908	-0.396921	1.895459
F	7.920450	-1.746945	2.663122
F	7.864532	-1.280974	0.542540
H	5.786690	2.076004	-1.678748
H	6.873190	1.058691	-0.708869
H	5.302991	1.569806	-0.048479
H	5.681931	-0.290658	-2.477538
H	4.115952	0.183240	-1.771854
H	4.260434	-4.051359	2.373207
H	2.060201	-2.464717	-0.319082
H	-0.368519	-2.825804	-0.352836
H	-1.494477	-4.003144	1.525273
H	-0.145226	-4.861377	3.435209

H	2.306755	-4.531861	3.458482
H	7.291619	-4.684454	3.794510
H	8.880419	-5.250075	3.365052

No Aryl Substituent (Benzaldehyde) Intermediate 13 Conformer 1

C	4.692838	1.873610	1.239984
C	5.555489	0.961736	0.383775
O	5.531154	-0.407421	0.885281
C	4.567199	-1.227364	0.428857
O	3.665356	-0.879195	-0.311585
C	4.794888	-2.608486	0.953177
C	3.827561	-3.525006	1.240687
C	2.368078	-3.483553	1.244406
C	1.710715	-4.734441	1.251978
C	0.320735	-4.817709	1.272923
C	-0.443968	-3.648765	1.316627
C	0.191499	-2.401356	1.339154
C	1.580062	-2.313724	1.299039
C	6.200885	-3.022224	1.171762
N	6.502332	-3.763368	2.168540
C	7.715679	-4.351907	2.483872
N	8.619722	-3.558228	3.057853
S	7.912294	-6.018465	2.271483
C	7.269392	-2.611649	0.136094
F	6.722636	-2.125674	-0.993303
F	8.100132	-1.674805	0.637516
F	8.021288	-3.674892	-0.206713
H	4.797353	2.904836	0.886964
H	5.005475	1.835144	2.287805
H	3.638163	1.592700	1.173406
H	6.609823	1.239759	0.432451
H	5.228871	0.955003	-0.658169
H	4.221716	-4.497565	1.530303
H	2.305268	-5.643952	1.235559
H	-0.163319	-5.789798	1.266876
H	-1.528408	-3.707072	1.342983
H	-0.400211	-1.491982	1.390384
H	2.048406	-1.339204	1.319726
H	8.452875	-2.568334	3.185418
H	9.469322	-3.955752	3.435286

No Aryl Substituent (Benzaldehyde) Intermediate 13 Conformer 2

C	4.436514	1.937712	1.335717
C	5.405940	1.101956	0.517080
O	5.448562	-0.274908	0.995620
C	4.570591	-1.148721	0.474415

O	3.683974	-0.849152	-0.304518
C	4.866132	-2.526476	0.978331
C	3.933023	-3.489592	1.220995
C	2.472898	-3.490875	1.205102
C	1.848118	-4.754509	1.113888
C	0.460306	-4.871969	1.107346
C	-0.333813	-3.727873	1.224038
C	0.269503	-2.470341	1.347373
C	1.655813	-2.347505	1.334402
C	6.287116	-2.885339	1.204715
N	6.598797	-3.698117	2.139270
C	7.837025	-4.199476	2.500213
N	8.070422	-5.454083	2.116943
S	8.863008	-3.318957	3.519887
C	7.358059	-2.328965	0.241215
F	6.807447	-1.893595	-0.910119
F	8.049926	-1.313524	0.783409
F	8.240515	-3.296921	-0.079978
H	4.489137	2.979627	1.003078
H	4.692443	1.902390	2.398912
H	3.408937	1.587179	1.205587
H	6.434346	1.449814	0.629520
H	5.139042	1.092612	-0.541769
H	4.356133	-4.457862	1.482241
H	2.465758	-5.645718	1.040964
H	0.000375	-5.852181	1.024034
H	-1.416680	-3.814080	1.230682
H	-0.345535	-1.581994	1.457876
H	2.099894	-1.366578	1.439172
H	7.423128	-5.953912	1.520802
H	8.904645	-5.928468	2.433257

No Aryl Substituent (Benzaldehyde) Intermediate 13 Conformer 3

C	6.128128	1.045387	-1.019576
C	5.373554	0.923970	0.294457
O	5.414436	-0.445104	0.797658
C	4.469417	-1.301275	0.369091
O	3.546114	-0.995498	-0.363012
C	4.735928	-2.664409	0.925847
C	3.787979	-3.579694	1.272421
C	2.328794	-3.538990	1.321698
C	1.668590	-4.787675	1.343540
C	0.279435	-4.866085	1.406408
C	-0.479407	-3.694806	1.480727
C	0.160778	-2.449685	1.491999

C	1.547850	-2.366861	1.409342
C	6.152406	-3.057228	1.110332
N	6.495450	-3.761538	2.120198
C	7.725245	-4.327269	2.412722
N	8.638878	-3.505617	2.929328
S	7.933645	-5.997544	2.247273
C	7.178042	-2.672384	0.023261
F	6.580963	-2.239353	-1.102915
F	8.009167	-1.701719	0.456525
F	7.936120	-3.734710	-0.305740
H	6.153166	2.096846	-1.323709
H	5.638383	0.469968	-1.809845
H	7.156498	0.690558	-0.909939
H	4.328978	1.224539	0.194189
H	5.845398	1.509131	1.085658
H	4.196763	-4.542275	1.574822
H	2.259534	-5.698854	1.303527
H	-0.208242	-5.836394	1.409533
H	-1.562684	-3.749630	1.540805
H	-0.425798	-1.538792	1.569001
H	2.020870	-1.394107	1.425783
H	8.463819	-2.514219	3.032326
H	9.505200	-3.881584	3.290468

No Aryl Substituent (Benzaldehyde) Intermediate 13 Conformer 4

C	3.912468	0.434614	-1.922731
C	2.974368	-0.049934	-0.829302
O	3.389179	-1.360624	-0.338500
C	4.284474	-1.387975	0.652897
O	4.793628	-0.403006	1.162356
C	4.643206	-2.782083	1.074251
C	3.754027	-3.762857	1.386705
C	2.297355	-3.720529	1.479550
C	1.591508	-4.925854	1.283245
C	0.200990	-4.959473	1.364719
C	-0.507674	-3.795279	1.676548
C	0.181759	-2.599607	1.910864
C	1.569774	-2.558956	1.812454
C	6.084491	-3.071960	1.215261
N	6.485850	-3.935996	2.068510
C	7.762364	-4.420240	2.290545
N	8.568262	-3.635946	3.006568
S	8.158739	-5.986829	1.789242
C	7.080296	-2.333354	0.298168
F	6.480249	-1.831488	-0.798302

F	7.665078	-1.314261	0.959384
F	8.054498	-3.160230	-0.120578
H	3.548634	1.390084	-2.314539
H	3.953181	-0.283351	-2.747328
H	4.923322	0.583529	-1.533628
H	1.963815	-0.215527	-1.206922
H	2.939144	0.644871	0.012107
H	4.195293	-4.732256	1.612351
H	2.144107	-5.832968	1.053463
H	-0.328149	-5.892876	1.196842
H	-1.590939	-3.821335	1.753051
H	-0.364364	-1.700344	2.180344
H	2.091186	-1.634398	2.035611
H	8.279643	-2.714803	3.310740
H	9.463135	-3.987558	3.319354

No Aryl Substituent (Benzaldehyde) Intermediate 14 Conformer 1

C	2.649174	1.712364	0.479208
C	3.047872	0.513589	-0.364187
O	3.336756	-0.646393	0.479328
C	4.571460	-0.764069	0.962861
O	5.488919	0.011918	0.753874
C	4.732552	-1.995468	1.844286
C	4.014401	-3.284028	1.326654
O	4.789456	-4.419231	1.742978
C	2.563542	-3.394059	1.753064
C	1.562111	-3.577409	0.791362
C	0.227844	-3.732357	1.175175
C	-0.118412	-3.711456	2.528786
C	0.875097	-3.533205	3.496294
C	2.207178	-3.372887	3.110129
C	6.223160	-2.260896	2.043281
O	6.775882	-2.206611	3.117714
C	7.105677	-2.615255	0.813940
F	6.398749	-2.682693	-0.333540
F	8.064373	-1.685975	0.658423
F	7.707145	-3.801041	1.004395
H	2.368386	2.539147	-0.181513
H	3.479252	2.043231	1.109533
H	1.791952	1.473891	1.115809
H	3.926330	0.720538	-0.978224
H	2.229019	0.175342	-1.000967
H	4.353503	-1.731490	2.837832
H	4.066053	-3.296288	0.236860
H	4.587988	-4.607868	2.672150

H	1.828518	-3.596635	-0.262240
H	-0.538843	-3.870771	0.417907
H	-1.155660	-3.831926	2.828704
H	0.613693	-3.515291	4.550595
H	2.966074	-3.230219	3.876826

No Aryl Substituent (Benzaldehyde) Intermediate 14 Conformer 2

C	3.020847	1.873595	0.889401
C	2.515935	0.498500	0.487514
O	3.154891	-0.547009	1.287544
C	4.326488	-1.017266	0.859409
O	4.917678	-0.643615	-0.136143
C	4.838099	-2.119427	1.791906
C	3.912101	-3.374995	1.706575
O	3.762956	-3.784026	0.358125
C	4.375817	-4.493350	2.640927
C	4.562140	-4.242836	4.009586
C	4.965389	-5.264828	4.870601
C	5.187824	-6.553892	4.376264
C	4.995784	-6.812227	3.017666
C	4.589519	-5.788732	2.156063
C	6.267320	-2.449216	1.396843
O	6.579075	-3.288011	0.580416
C	7.372182	-1.655130	2.132698
F	7.106365	-0.334740	2.093069
F	7.392304	-2.036646	3.427138
F	8.576579	-1.870394	1.601648
H	2.471843	2.636241	0.327337
H	4.085596	1.982657	0.665319
H	2.861477	2.051193	1.957130
H	2.701943	0.287746	-0.567267
H	1.452821	0.378343	0.701602
H	4.816028	-1.723470	2.811372
H	2.919005	-3.041272	2.024971
H	4.652488	-3.974096	0.016078
H	4.382096	-3.250169	4.414767
H	5.103771	-5.053879	5.927459
H	5.503416	-7.349170	5.045840
H	5.159159	-7.811782	2.623872
H	4.427166	-5.998493	1.104172

No Aryl Substituent (Benzaldehyde) Intermediate 14 Conformer 3

C	2.894794	1.807723	0.847880
C	2.506457	0.418922	0.370728
O	3.130228	-0.612893	1.200287
C	4.361825	-1.002233	0.871405

O	5.019104	-0.563233	-0.053692
C	4.851149	-2.103346	1.816864
C	4.004153	-3.401352	1.623707
O	3.992685	-3.781126	0.258420
C	4.444149	-4.519476	2.569566
C	4.495081	-4.297731	3.954794
C	4.875993	-5.320741	4.824642
C	5.211135	-6.582190	4.322628
C	5.154022	-6.812390	2.946764
C	4.770039	-5.787943	2.076036
C	6.324039	-2.347364	1.535632
O	6.747440	-3.149129	0.732242
C	7.320186	-1.516399	2.378129
F	6.976836	-0.213770	2.364870
F	7.269317	-1.947087	3.656288
F	8.571309	-1.639185	1.932227
H	2.354386	2.554618	0.257071
H	3.967067	1.981290	0.722249
H	2.632421	1.945975	1.900986
H	2.796684	0.248011	-0.667638
H	1.437193	0.234910	0.485711
H	4.723814	-1.738716	2.840545
H	2.971061	-3.127500	1.861879
H	4.917093	-3.916054	-0.009272
H	4.225966	-3.327487	4.364935
H	4.908900	-5.132474	5.894269
H	5.509298	-7.378113	4.999395
H	5.405671	-7.790747	2.546316
H	4.712206	-5.976671	1.009337

No Aryl Substituent (Benzaldehyde) Intermediate 14 Conformer 4

C	3.112491	0.067996	-1.852550
C	2.694156	0.340648	-0.417892
O	3.067775	-0.773405	0.453947
C	4.299528	-0.773071	0.957940
O	5.141957	0.087456	0.763759
C	4.565568	-1.980544	1.847901
C	3.892229	-3.313282	1.386315
O	4.747837	-4.400383	1.772129
C	2.473902	-3.493356	1.891427
C	1.433838	-3.742425	0.987148
C	0.131958	-3.961558	1.443815
C	-0.142599	-3.939367	2.813793
C	0.889935	-3.695247	3.724347
C	2.189290	-3.470979	3.265307

C	6.076127	-2.165953	1.973070
O	6.679611	-2.088337	3.018069
C	6.909368	-2.464225	0.695263
F	6.143456	-2.562916	-0.412501
F	7.804089	-1.481234	0.495539
F	7.584024	-3.615614	0.840864
H	2.762835	0.886023	-2.490857
H	2.672718	-0.865180	-2.216972
H	4.200835	0.005909	-1.939598
H	1.609547	0.399737	-0.315532
H	3.148194	1.252715	-0.026169
H	4.224542	-1.717597	2.855376
H	3.885835	-3.338353	0.295628
H	4.612257	-4.582414	2.714482
H	1.644748	-3.761867	-0.078873
H	-0.665431	-4.150465	0.730441
H	-1.154626	-4.109493	3.170431
H	0.684199	-3.675604	4.790871
H	2.979106	-3.277417	3.988382

No Aryl Substituent (Benzaldehyde) Intermediate 14 Conformer 5

C	1.907235	-0.180963	-0.844356
C	2.698285	0.471662	0.276063
O	3.299185	-0.540157	1.145065
C	4.490795	-1.022977	0.792448
O	5.144049	-0.663784	-0.169084
C	4.939874	-2.112756	1.770236
C	4.008644	-3.360923	1.645057
O	3.927337	-3.776959	0.292904
C	4.410948	-4.478877	2.608095
C	4.560829	-4.218857	3.979480
C	4.904930	-5.243145	4.863336
C	5.103637	-6.543727	4.389888
C	4.947632	-6.811263	3.028489
C	4.600697	-5.785718	2.143902
C	6.386366	-2.461068	1.464408
O	6.736971	-3.310418	0.674961
C	7.454271	-1.671854	2.257991
F	7.215160	-0.348293	2.175358
F	7.382552	-2.026847	3.557906
F	8.687184	-1.917838	1.812130
H	1.420466	0.597127	-1.441444
H	1.133618	-0.841823	-0.442292
H	2.561619	-0.759927	-1.502034
H	2.056024	1.045217	0.946201

H	3.493802	1.114213	-0.105535
H	4.861174	-1.702013	2.780866
H	3.003069	-3.015918	1.907280
H	4.831003	-3.985596	0.002602
H	4.399413	-3.216969	4.369047
H	5.016233	-5.024568	5.921843
H	5.373404	-7.340541	5.077406
H	5.093274	-7.819573	2.650350
H	4.466519	-6.001564	1.089332

No Aryl Substituent (Benzaldehyde) Intermediate 15 Conformer 1

C	2.272362	0.365846	1.327280
C	3.426222	-0.293993	0.591082
O	3.689410	0.342440	-0.699036
C	4.484369	1.418107	-0.703989
O	4.971899	1.930032	0.287985
C	4.741811	1.908901	-2.103393
C	3.791804	2.441949	-2.920278
C	2.387337	2.731941	-2.674282
C	1.840115	2.935720	-1.389670
C	0.486048	3.218822	-1.237839
C	-0.348265	3.299212	-2.359366
C	0.182557	3.118767	-3.640401
C	1.540764	2.854313	-3.797480
C	6.109319	1.814413	-2.659508
O	6.438066	2.232123	-3.758015
C	7.219091	1.139235	-1.806736
F	6.767378	0.019851	-1.198226
F	8.258157	0.797876	-2.577495
F	7.661954	1.982925	-0.855215
H	2.050314	-0.213997	2.229336
H	1.372582	0.390924	0.705731
H	2.524719	1.385202	1.631270
H	3.192494	-1.326006	0.324375
H	4.346627	-0.270473	1.178349
H	4.144189	2.690616	-3.920331
H	2.479784	2.921831	-0.514677
H	0.079853	3.387354	-0.244974
H	-1.404825	3.517515	-2.233716
H	-0.458265	3.195314	-4.513654
H	1.958329	2.725638	-4.792329

No Aryl Substituent (Benzaldehyde) Intermediate 15 Conformer 2

C	2.095284	-0.908720	0.665697
C	3.116534	0.204447	0.783271
O	3.472750	0.613812	-0.571115

C	4.386793	1.582344	-0.681695
O	4.948820	2.112497	0.260385
C	4.647692	1.938835	-2.118554
C	3.715418	2.489895	-2.944692
C	2.346357	2.906400	-2.680277
C	1.847782	3.215447	-1.395760
C	0.524144	3.611494	-1.231088
C	-0.328869	3.702597	-2.337937
C	0.154563	3.418388	-3.618784
C	1.483670	3.039635	-3.789926
C	5.985178	1.695202	-2.698618
O	6.315419	2.010534	-3.830478
C	7.057325	0.983090	-1.828164
F	6.526697	-0.045527	-1.129623
F	8.036497	0.495323	-2.599031
F	7.604264	1.845974	-0.951130
H	1.805827	-1.239282	1.667965
H	2.509450	-1.765810	0.126323
H	1.197523	-0.564472	0.143582
H	4.027268	-0.120657	1.293769
H	2.718244	1.075557	1.312066
H	4.051983	2.643622	-3.968978
H	2.503312	3.194927	-0.532335
H	0.156802	3.858963	-0.239658
H	-1.362001	4.009237	-2.202106
H	-0.499775	3.502286	-4.481269
H	1.864847	2.828662	-4.785342

No Aryl Substituent (Benzaldehyde) Intermediate 15 Conformer 3

C	3.982465	-1.682065	0.432476
C	2.994645	-0.569945	0.120287
O	3.414704	0.178251	-1.062419
C	4.261286	1.197062	-0.875955
O	4.704268	1.549114	0.202943
C	4.613274	1.865354	-2.176138
C	3.723321	2.552704	-2.944561
C	2.318723	2.860257	-2.721921
C	1.696713	2.850324	-1.454144
C	0.346783	3.163316	-1.329858
C	-0.410606	3.487051	-2.462340
C	0.194321	3.519280	-3.722476
C	1.549251	3.223910	-3.848653
C	6.003047	1.796462	-2.674648
O	6.402631	2.348277	-3.687262
C	7.043923	0.967589	-1.871301

F	6.526096	-0.210093	-1.453537
F	8.113971	0.700144	-2.628689
F	7.456958	1.650317	-0.786962
H	3.614838	-2.263083	1.284582
H	4.963644	-1.274527	0.689818
H	4.092740	-2.355696	-0.422567
H	2.889139	0.125646	0.955275
H	2.013709	-0.963791	-0.151111
H	4.133848	2.936529	-3.877413
H	2.274974	2.646029	-0.560269
H	-0.116262	3.164221	-0.347639
H	-1.464778	3.727529	-2.358790
H	-0.386198	3.784524	-4.600965
H	2.024369	3.258410	-4.825264

No Aryl Substituent (Benzaldehyde) Intermediate 15 Conformer 4

C	6.064383	-0.840262	1.339272
C	5.239800	0.435271	1.394060
O	5.312699	1.165644	0.130888
C	4.435063	0.845729	-0.830716
O	3.545296	0.022582	-0.710024
C	4.698580	1.657181	-2.063720
C	3.756926	2.365300	-2.754677
C	2.338384	2.624246	-2.561831
C	1.581768	2.289654	-1.415565
C	0.231868	2.618020	-1.343970
C	-0.398673	3.275899	-2.407482
C	0.333355	3.617627	-3.547938
C	1.687115	3.302376	-3.618967
C	6.069791	1.715713	-2.622719
O	6.442020	2.528313	-3.454143
C	7.121329	0.647895	-2.201070
F	6.547724	-0.528959	-1.865863
F	7.960151	0.414931	-3.220955
F	7.853958	1.072279	-1.153969
H	6.062234	-1.310542	2.328003
H	7.098468	-0.621329	1.059842
H	5.647434	-1.549181	0.619179
H	5.636895	1.140306	2.126323
H	4.190432	0.233065	1.616647
H	4.161359	2.855384	-3.639079
H	2.041840	1.776236	-0.582708
H	-0.334792	2.359311	-0.454397
H	-1.454428	3.523333	-2.343319
H	-0.147141	4.132709	-4.374250

H	2.259360	3.578070	-4.500553

No Aryl Substituent (Benzaldehyde) Intermediate 15 Conformer 5

C	6.310971	-0.622701	1.435630
C	5.419003	0.607759	1.455054
O	5.448083	1.301403	0.169497
C	4.587879	0.903211	-0.778094
O	3.746198	0.035306	-0.628974
C	4.801566	1.689923	-2.036901
C	3.821693	2.338507	-2.733160
C	2.395710	2.541810	-2.528670
C	1.663018	2.187069	-1.372767
C	0.301908	2.462201	-1.291265
C	-0.363969	3.085912	-2.353948
C	0.343532	3.445804	-3.504210
C	1.707888	3.183059	-3.585703
C	6.162031	1.792555	-2.615618
O	6.483790	2.585580	-3.486230
C	7.271204	0.800317	-2.159323
F	6.762252	-0.393239	-1.780661
F	8.121202	0.576808	-3.171691
F	7.979584	1.301442	-1.129650
H	6.339509	-1.059614	2.439152
H	7.330242	-0.357688	1.141971
H	5.929131	-1.376331	0.741946
H	5.779981	1.355669	2.163050
H	4.382868	0.356291	1.689177
H	4.194730	2.824027	-3.633739
H	2.149298	1.697343	-0.540549
H	-0.246056	2.188083	-0.394626
H	-1.428041	3.292032	-2.281783
H	-0.164651	3.933864	-4.330376
H	2.260645	3.472600	-4.475239

No Aryl Substituent (Benzaldehyde) Intermediate 16 Conformer 1

C	8.584651	-0.478433	0.011395
C	8.301225	-0.462456	-1.480266
O	6.890985	-0.759763	-1.746802
C	6.540925	-2.039704	-1.846246
O	7.285766	-2.993785	-1.727260
C	5.038213	-2.169142	-2.141988
C	4.651772	-1.470400	-3.479815
N	5.412243	-2.059932	-4.580708
C	5.707559	-1.448417	-5.751627
N	5.317726	-0.174230	-5.917150
S	6.586288	-2.279562	-6.965316

C	3.137147	-1.447779	-3.694496
C	2.380524	-0.453311	-3.057567
C	0.992882	-0.415571	-3.205206
C	0.347482	-1.370362	-3.996452
C	1.096795	-2.359144	-4.638763
C	2.485514	-2.399315	-4.489162
C	4.681019	-3.649563	-2.093309
O	4.747041	-4.408442	-3.032963
C	4.190683	-4.171657	-0.721502
F	5.034705	-3.788360	0.255863
F	2.980575	-3.634558	-0.461572
F	4.085247	-5.500428	-0.708204
H	9.624624	-0.180712	0.180726
H	8.442414	-1.478918	0.429198
H	7.934506	0.224827	0.540100
H	8.912346	-1.186950	-2.021583
H	8.442280	0.529800	-1.910914
H	4.504254	-1.643140	-1.344398
H	4.996396	-0.439168	-3.353169
H	5.598240	-3.054133	-4.544556
H	4.669327	0.290118	-5.296905
H	5.528461	0.282463	-6.791532
H	2.878628	0.297981	-2.448414
H	0.418919	0.362417	-2.709831
H	-0.731825	-1.339780	-4.116055
H	0.602743	-3.100888	-5.259996
H	3.059323	-3.167504	-4.997954

No Aryl Substituent (Benzaldehyde) Intermediate 16 Conformer 2

C	8.548889	-0.357766	-0.173598
C	8.227786	-0.353723	-1.657782
O	6.818293	-0.682176	-1.887167
C	6.493479	-1.969360	-1.976513
O	7.261879	-2.907082	-1.878000
C	4.986191	-2.133051	-2.228238
C	4.538756	-1.426510	-3.542850
N	5.277242	-1.981855	-4.675660
C	5.536068	-1.342544	-5.840362
N	5.119199	-0.074243	-5.974985
S	6.398507	-2.136988	-7.090615
C	3.017646	-1.439778	-3.707366
C	2.257382	-0.476965	-3.027631
C	0.865062	-0.473438	-3.128552
C	0.218282	-1.431107	-3.915229
C	0.970942	-2.388216	-4.600022

C	2.364548	-2.394203	-4.497286
C	4.670413	-3.623069	-2.189514
O	4.724508	-4.367290	-3.141575
C	4.239692	-4.177323	-0.810103
F	5.100821	-3.781004	0.146678
F	3.022227	-3.681142	-0.506420
F	4.175130	-5.508907	-0.811875
H	9.586149	-0.037171	-0.031661
H	8.438696	-1.359459	0.250997
H	7.897127	0.333413	0.368857
H	8.840950	-1.066920	-2.211645
H	8.337259	0.639604	-2.095133
H	4.464926	-1.633096	-1.405741
H	4.861342	-0.388842	-3.411929
H	5.480455	-2.973239	-4.663672
H	4.505070	0.382655	-5.315841
H	5.320659	0.411289	-6.835762
H	2.755745	0.276863	-2.421844
H	0.288248	0.280456	-2.600299
H	-0.864879	-1.427171	-3.998337
H	0.475704	-3.131892	-5.218017
H	2.940905	-3.137649	-5.038909

No Aryl Substituent (Benzaldehyde) Intermediate 16 Conformer 3

C	8.441762	-0.263896	-0.169541
C	8.158541	-0.357824	-1.658467
O	6.743711	-0.651903	-1.902461
C	6.372952	-1.929804	-1.904090
O	7.102709	-2.883973	-1.713127
C	4.867940	-2.058694	-2.186837
C	4.485373	-1.447986	-3.568081
N	5.230702	-2.124038	-4.628721
C	5.546791	-1.595122	-5.834315
N	5.203098	-0.322058	-6.080836
S	6.385389	-2.534490	-6.997484
C	2.970148	-1.415380	-3.777025
C	2.233128	-0.369540	-3.202710
C	0.845395	-0.318932	-3.345636
C	0.180099	-1.312644	-4.069604
C	0.909864	-2.353262	-4.649500
C	2.298670	-2.406114	-4.504288
C	4.492863	-3.527255	-2.033876
O	4.531016	-4.347920	-2.921670
C	4.016540	-3.950113	-0.623161
F	4.862400	-3.491656	0.319271

F	2.804127	-3.402831	-0.396551
F	3.919432	-5.275142	-0.512363
H	9.486616	0.028220	-0.022033
H	8.282257	-1.226873	0.323408
H	7.803483	0.488714	0.302638
H	8.757867	-1.131453	-2.141812
H	8.315954	0.595948	-2.163903
H	4.343814	-1.472951	-1.424946
H	4.846593	-0.416434	-3.513234
H	5.380948	-3.120409	-4.533652
H	4.629870	0.231818	-5.460429
H	5.458450	0.085254	-6.967477
H	2.746538	0.411674	-2.646142
H	0.287013	0.499070	-2.899308
H	-0.899286	-1.272581	-4.185690
H	0.400387	-3.125950	-5.218366
H	2.857044	-3.215070	-4.964781

No Aryl Substituent (Benzaldehyde) Intermediate 16 Conformer 4

C	9.360974	-3.244200	-2.062252
C	8.432197	-2.890059	-0.914401
O	7.028946	-2.983092	-1.329085
C	6.478931	-1.915430	-1.907892
O	7.037218	-0.863952	-2.136915
C	4.989661	-2.157065	-2.221734
C	4.593566	-1.465336	-3.555399
N	5.302003	-2.092757	-4.670631
C	5.596055	-1.499431	-5.848521
N	5.230398	-0.217743	-6.020920
S	6.443352	-2.357948	-7.066884
C	3.076302	-1.398339	-3.734087
C	2.370915	-0.338271	-3.146774
C	0.981308	-0.263061	-3.260866
C	0.283131	-1.246702	-3.967209
C	0.981371	-2.302318	-4.559334
C	2.371720	-2.379030	-4.444246
C	4.650776	-3.640545	-2.171890
O	4.847034	-4.423071	-3.076346
C	3.961808	-4.136627	-0.878114
F	4.606771	-3.693543	0.215652
F	2.706379	-3.639793	-0.851619
F	3.898283	-5.468460	-0.834779
H	10.394257	-3.237236	-1.700232
H	9.277877	-2.517993	-2.875577
H	9.138634	-4.242391	-2.450708

H	8.609581	-1.881153	-0.537799
H	8.506268	-3.605098	-0.094044
H	4.440844	-1.643139	-1.425037
H	4.980396	-0.446576	-3.446687
H	5.487942	-3.086252	-4.613240
H	4.579999	0.253024	-5.407177
H	5.423329	0.218619	-6.909828
H	2.911176	0.432921	-2.601914
H	0.446994	0.566091	-2.805612
H	-0.797597	-1.187239	-4.060721
H	0.445745	-3.066988	-5.114933
H	2.906355	-3.197117	-4.917547

No Aryl Substituent (Benzaldehyde) Intermediate 16 Conformer 5

C	8.424850	-0.223625	-0.347797
C	8.085140	-0.303633	-1.825630
O	6.667011	-0.619088	-2.019350
C	6.316803	-1.902472	-2.016092
O	7.067702	-2.846373	-1.858450
C	4.804236	-2.052594	-2.245428
C	4.367856	-1.462806	-3.619694
N	5.088744	-2.139533	-4.697326
C	5.337461	-1.624508	-5.924290
N	4.908300	-0.378971	-6.185293
S	6.205539	-2.535794	-7.087227
C	2.845725	-1.454122	-3.777024
C	2.116785	-0.407401	-3.193651
C	0.724275	-0.375594	-3.286143
C	0.045368	-1.389227	-3.968580
C	0.766361	-2.430766	-4.557711
C	2.160010	-2.464666	-4.462758
C	4.451704	-3.523983	-2.064234
O	4.484435	-4.356638	-2.941169
C	4.015899	-3.934183	-0.636662
F	4.888786	-3.464506	0.275025
F	2.809529	-3.386788	-0.380416
F	3.925719	-5.257908	-0.510087
H	9.468832	0.087295	-0.237457
H	8.303490	-1.195871	0.137663
H	7.791198	0.510030	0.159158
H	8.678052	-1.060484	-2.342417
H	8.206239	0.659021	-2.324129
H	4.298801	-1.463567	-1.473909
H	4.713613	-0.424383	-3.590409
H	5.318169	-3.117166	-4.573870

H	4.256403	0.114959	-5.591822
H	5.077110	0.003554	-7.103373
H	2.640663	0.388773	-2.669114
H	0.172566	0.443153	-2.833039
H	-1.037912	-1.363959	-4.045546
H	0.246419	-3.219044	-5.094727
H	2.708518	-3.276031	-4.930621

No Aryl Substituent (Benzaldehyde) Intermediate 2 Conformer 0

H	2.559968	1.037322	-0.354552
C	2.123722	0.129916	0.111709
O	1.056369	-0.319412	-0.284450
C	2.927275	-0.448378	1.204797
C	2.492812	-1.599832	1.884570
C	3.265457	-2.130597	2.913035
C	4.474461	-1.516016	3.268308
C	4.911423	-0.370473	2.595486
C	4.138198	0.163316	1.564175
H	1.553608	-2.061059	1.594351
H	2.932844	-3.020011	3.440309
H	5.075563	-1.932346	4.071964
H	5.848632	0.101744	2.874738
H	4.468668	1.053825	1.034563

No Aryl Substituent (Benzaldehyde) Intermediate 2 Conformer 1

H	2.560167	1.037236	-0.354526
C	2.123727	0.129956	0.111794
O	1.056274	-0.319232	-0.284248
C	2.927248	-0.448386	1.204831
C	2.492701	-1.599724	1.884742
C	3.265364	-2.130496	2.913169
C	4.474459	-1.516023	3.268266
C	4.911516	-0.370601	2.595326
C	4.138268	0.163187	1.564039
H	1.553379	-2.060744	1.594537
H	2.932741	-3.019826	3.440585
H	5.075575	-1.932371	4.071909
H	5.848810	0.101528	2.874458
H	4.468672	1.053598	1.034220

No Aryl Substituent (Benzaldehyde) Intermediate 2 Conformer 2

H	2.574945	1.023002	-0.371643
C	2.119911	0.138300	0.119378
O	1.032171	-0.285857	-0.248188
C	2.925270	-0.444285	1.208985
C	2.468314	-1.569055	1.917952
C	3.243307	-2.104275	2.942183

C	4.476917	-1.520602	3.263725
C	4.936259	-0.401692	2.561877
C	4.160682	0.136719	1.534753
H	1.510050	-2.004912	1.651700
H	2.894497	-2.973090	3.492829
H	5.079723	-1.940707	4.064161
H	5.892468	0.046378	2.815404
H	4.506587	1.006676	0.981384

No Aryl Substituent (Benzaldehyde) Intermediate 2 Conformer 3

H	1.204913	-0.432475	-0.116820
C	2.123642	0.142916	0.119072
O	2.394183	1.163311	-0.500422
C	2.944397	-0.424097	1.205370
C	2.522419	-1.602608	1.840133
C	3.286290	-2.153063	2.869662
C	4.472022	-1.525788	3.264658
C	4.897723	-0.348397	2.633509
C	4.137341	0.202389	1.606571
H	1.598373	-2.081086	1.523939
H	2.961620	-3.064870	3.362122
H	5.067627	-1.953233	4.066596
H	5.820054	0.132150	2.946831
H	4.447597	1.113450	1.103778

No Aryl Substituent (Benzaldehyde) Intermediate 3 Conformer 0

H	1.033018	-0.171623	-0.469367
C	2.103020	-0.038078	-0.265919
O	2.732254	-0.485042	-1.456049
N	2.350384	1.383791	-0.031828
C	1.928857	2.382602	-0.845642
N	1.397140	2.015794	-2.025641
S	2.051601	4.020765	-0.365322
C	2.488353	-0.832625	0.975672
C	3.317169	-1.954919	0.877838
C	3.648141	-2.688008	2.021436
C	3.146243	-2.310661	3.268754
C	2.305942	-1.197358	3.369110
C	1.976495	-0.463077	2.228641
H	3.685172	-0.316403	-1.377921
H	2.808804	1.662966	0.824751
H	1.608447	1.099042	-2.405071
H	1.127492	2.744643	-2.669393
H	3.697945	-2.259070	-0.091460
H	4.297043	-3.554994	1.933875
H	3.403219	-2.881839	4.156444

H	1.904151	-0.901234	4.334056
H	1.315110	0.395328	2.315034

No Aryl Substituent (Benzaldehyde) Intermediate 3 Conformer 1

H	1.076002	-0.185166	-0.511653
C	2.145298	-0.061801	-0.298256
O	2.782026	-0.534878	-1.474324
N	2.407833	1.360379	-0.083232
C	2.008567	2.351600	-0.917276
N	1.484834	1.973321	-2.097192
S	2.147647	3.995524	-0.461168
C	2.507439	-0.842051	0.959270
C	3.314997	-1.981576	0.885270
C	3.624917	-2.701777	2.042816
C	3.122995	-2.294101	3.280549
C	2.303673	-1.163422	3.357193
C	1.995186	-0.442037	2.202704
H	3.736383	-0.379348	-1.386560
H	2.860984	1.647004	0.773680
H	1.687662	1.047950	-2.459897
H	1.231116	2.695322	-2.754981
H	3.695321	-2.308674	-0.076682
H	4.257404	-3.582454	1.973736
H	3.363636	-2.855162	4.179199
H	1.902086	-0.843606	4.314630
H	1.349793	0.430054	2.270874

No Aryl Substituent (Benzaldehyde) Intermediate 3 Conformer 2

H	0.864538	-0.081596	-0.233293
C	1.945780	0.048035	-0.098338
O	2.491554	-0.335643	-1.350187
N	2.204935	1.458086	0.188343
C	1.722894	2.493234	-0.542010
N	1.106210	2.181999	-1.696434
S	1.877148	4.106399	0.008354
C	2.420203	-0.804380	1.072015
C	3.234506	-1.921334	0.858658
C	3.648617	-2.707736	1.937932
C	3.244775	-2.389388	3.236225
C	2.418951	-1.281776	3.452569
C	2.006527	-0.494312	2.376363
H	3.447450	-0.166822	-1.329439
H	2.725424	1.696966	1.021317
H	1.291573	1.287124	-2.136763
H	0.788500	2.940396	-2.281447
H	3.539278	-2.179020	-0.150174

H	4.285506	-3.569904	1.760577
H	3.566513	-3.001646	4.074024
H	2.093342	-1.031373	4.458319
H	1.357278	0.359390	2.553388

No Aryl Substituent (Benzaldehyde) Intermediate 3 Conformer 3

H	0.744044	0.144135	0.047834
C	1.836872	0.075526	-0.024670
O	2.056448	-0.474779	-1.313745
N	2.387700	1.425755	0.081491
C	1.967000	2.489321	-0.645935
N	1.097389	2.235236	-1.640272
S	2.506943	4.073058	-0.283823
C	2.371314	-0.783010	1.114887
C	2.919209	-2.044888	0.862062
C	3.386693	-2.834429	1.916726
C	3.301878	-2.373018	3.232201
C	2.742513	-1.117848	3.490892
C	2.277369	-0.327426	2.438457
H	3.013067	-0.487209	-1.479267
H	3.092499	1.609281	0.782436
H	1.032594	1.296885	-2.020201
H	0.814282	3.004950	-2.228254
H	2.974154	-2.411262	-0.157598
H	3.814631	-3.810952	1.707625
H	3.664304	-2.987915	4.051259
H	2.665050	-0.753917	4.511589
H	1.835548	0.642805	2.651206

No Aryl Substituent (Benzaldehyde) Intermediate 3 Conformer 4

H	0.835430	0.268413	-0.052984
C	1.906949	0.054201	-0.133206
O	2.019625	-0.665376	-1.358931
N	2.655290	1.313288	-0.196141
C	2.408073	2.312073	-1.083521
N	1.632146	2.011848	-2.137178
S	3.060327	3.877168	-0.842953
C	2.361871	-0.755671	1.064697
C	3.721557	-1.037310	1.266125
C	4.123698	-1.820429	2.349699
C	3.172025	-2.328296	3.239554
C	1.816735	-2.050805	3.042420
C	1.413648	-1.267351	1.958121
H	2.950763	-0.908682	-1.485935
H	3.181599	1.583919	0.623401
H	1.466794	1.038117	-2.373185

H	1.493363	2.717251	-2.845027
H	4.468646	-0.642914	0.581352
H	5.178616	-2.032000	2.499528
H	3.486405	-2.936092	4.083364
H	1.073629	-2.441170	3.731989
H	0.359309	-1.051182	1.805511

No Aryl Substituent (Benzaldehyde) Intermediate 4 Conformer 0

H	2.401697	-0.565496	-0.661782
C	2.015606	0.030856	0.173201
N	0.937729	-0.317165	0.787576
C	0.220463	-1.433273	0.352198
N	-0.390685	-2.063091	1.358302
S	0.049271	-1.924910	-1.266171
C	2.785172	1.214494	0.539598
C	2.385986	2.068920	1.587081
C	3.146091	3.191181	1.897251
C	4.311730	3.473881	1.170693
C	4.716674	2.631045	0.131528
C	3.956966	1.504908	-0.183083
H	-0.269924	-1.735127	2.308288
H	-0.956052	-2.882190	1.188081
H	1.483804	1.836006	2.143460
H	2.837509	3.849138	2.704383
H	4.902883	4.351171	1.418003
H	5.619862	2.851069	-0.429656
H	4.264219	0.844584	-0.989951

No Aryl Substituent (Benzaldehyde) Intermediate 4 Conformer 1

H	2.395808	-0.568015	-0.653669
C	2.002677	0.039717	0.169707
N	0.911704	-0.292372	0.770118
C	0.192126	-1.407248	0.332803
N	-0.466161	-2.001324	1.330942
S	0.072474	-1.939249	-1.277467
C	2.777577	1.218994	0.537915
C	2.370407	2.086165	1.571892
C	3.136500	3.203603	1.884595
C	4.316046	3.468865	1.174016
C	4.728914	2.613446	0.148292
C	3.963319	1.492036	-0.168805
H	-0.373979	-1.650599	2.276031
H	-1.036854	-2.816832	1.161224
H	1.457416	1.866661	2.115897
H	2.821926	3.871178	2.681460
H	4.911831	4.342458	1.423275

H	5.642847	2.820092	-0.400428
H	4.276619	0.822125	-0.965396

No Aryl Substituent (Benzaldehyde) Intermediate 4 Conformer 2

H	2.362805	-0.507809	-0.729173
C	1.976938	0.083098	0.109753
N	0.878500	-0.246616	0.697688
C	0.198781	-1.400347	0.299101
N	-1.120598	-1.288234	0.470421
S	0.922531	-2.829597	-0.269761
C	2.766339	1.243614	0.505888
C	2.382199	2.077279	1.575580
C	3.169477	3.169356	1.923660
C	4.345265	3.444505	1.210654
C	4.733120	2.624391	0.147074
C	3.946701	1.528077	-0.205170
H	-1.511868	-0.424040	0.823371
H	-1.738803	-2.051653	0.236871
H	1.468436	1.855619	2.117393
H	2.872748	3.810924	2.748228
H	4.956274	4.299621	1.485820
H	5.643563	2.839530	-0.404189
H	4.242191	0.885880	-1.030810

No Aryl Substituent (Benzaldehyde) Intermediate 4 Conformer 3

H	2.398059	-0.593804	-0.602797
C	1.954996	0.080110	0.139836
N	0.792455	-0.163161	0.639825
C	0.061914	-1.266589	0.193233
N	-0.711405	-1.778634	1.153518
S	0.061893	-1.874410	-1.394436
C	2.750489	1.241511	0.521682
C	2.288614	2.186170	1.460175
C	3.077622	3.282784	1.789418
C	4.334479	3.450063	1.190428
C	4.801740	2.517245	0.259980
C	4.013501	1.416320	-0.073131
H	-0.689323	-1.382471	2.084708
H	-1.306197	-2.573462	0.969034
H	1.315652	2.042860	1.919044
H	2.720679	4.009916	2.512940
H	4.947864	4.307698	1.452161
H	5.775590	2.647931	-0.202231
H	4.369380	0.686924	-0.796189

No Aryl Substituent (Benzaldehyde) Intermediate 4 Conformer 4

H	1.063867	-0.073341	0.724111

C	1.892753	0.165450	0.047493
N	2.078086	-0.512366	-1.032608
C	1.250582	-1.599932	-1.320068
N	1.087253	-1.773239	-2.633852
S	0.572185	-2.656537	-0.173892
C	2.749825	1.279587	0.436378
C	2.458919	1.966704	1.629345
C	3.258632	3.032469	2.040252
C	4.353227	3.420026	1.261801
C	4.649431	2.742120	0.070444
C	3.854364	1.678910	-0.343079
H	1.515690	-1.128130	-3.285660
H	0.521845	-2.530727	-2.988836
H	1.606100	1.659197	2.229009
H	3.030485	3.559140	2.962036
H	4.976980	4.250671	1.579949
H	5.500181	3.049144	-0.530820
H	4.070794	1.147855	-1.264402

No Aryl Substituent (Benzaldehyde) Intermediate 6 Conformer 1

C	8.894478	2.494500	1.120144
C	8.081785	1.414967	0.427192
O	6.662167	1.775690	0.395354
C	6.239133	2.526639	-0.618442
O	6.916682	2.913366	-1.551693
C	4.746281	2.855577	-0.461359
C	4.475211	3.644463	0.855001
N	5.260468	4.877779	0.856449
C	5.699116	5.535627	1.955040
N	5.433483	5.005098	3.159799
S	6.597880	6.985604	1.794627
C	2.979263	3.841416	1.106762
C	2.246828	2.787139	1.671953
C	0.874590	2.916186	1.892849
C	0.220424	4.104619	1.555085
C	0.945989	5.160574	0.998351
C	2.319180	5.030863	0.773961
C	4.296099	3.584215	-1.721246
O	4.345852	4.782004	-1.882931
C	3.725750	2.684783	-2.844054
F	4.548508	1.644940	-3.079519
F	2.536472	2.191745	-2.439478
F	3.548278	3.363615	-3.977614
H	9.936936	2.167725	1.192453
H	8.519263	2.676640	2.131502

H	8.867610	3.431411	0.556637
H	8.098681	0.474437	0.979567
H	8.415219	1.243240	-0.597638
H	4.208352	1.905732	-0.381599
H	4.873629	2.996925	1.642259
H	5.361898	5.370620	-0.021642
H	4.764299	4.260469	3.297447
H	5.744723	5.502769	3.980288
H	2.752452	1.863725	1.946327
H	0.319984	2.093452	2.335169
H	-0.846613	4.208435	1.730693
H	0.445646	6.089282	0.738743
H	2.874805	5.859927	0.347163

No Aryl Substituent (Benzaldehyde) Intermediate 6 Conformer 2

No Aryl Substituent (Benzaldehyde) Intermediate 6 Conformer 3

C	8.940079	2.475065	1.201776
C	8.159139	1.452124	0.395366
O	6.742332	1.817241	0.329573
C	6.361638	2.639334	-0.645167
O	7.077288	3.088796	-1.519893
C	4.863150	2.959371	-0.528989
C	4.529868	3.635586	0.835203
N	5.315434	4.859747	0.978910
C	5.701406	5.422487	2.148214
N	5.398888	4.788554	3.291079
S	6.573399	6.897929	2.150469
C	3.023491	3.818337	1.028750
C	2.260494	2.719547	1.450174
C	0.879047	2.836643	1.612500
C	0.246023	4.057253	1.359143
C	1.001962	5.156847	0.945298
C	2.384754	5.039351	0.779632
C	4.471527	3.793440	-1.742239
O	4.528912	5.000213	-1.800840
C	3.951132	2.993619	-2.960789
F	4.777204	1.966513	-3.238716
F	2.739800	2.483267	-2.657070
F	3.836314	3.763361	-4.043332
H	9.978846	2.141621	1.293918
H	8.521648	2.581397	2.206954
H	8.935427	3.451641	0.709518
H	8.153974	0.472697	0.875769
H	8.537466	1.357445	-0.623902

H	4.322358	2.008053	-0.555704
H	4.888849	2.920285	1.581420
H	5.446848	5.430579	0.153529
H	4.799137	3.977090	3.337231
H	5.699169	5.196132	4.163409
H	2.748909	1.769973	1.659003
H	0.300620	1.978800	1.943678
H	-0.828425	4.151432	1.489231
H	0.518121	6.110136	0.751997
H	2.963671	5.901495	0.463795

No Aryl Substituent (Benzaldehyde) Intermediate 6 Conformer 4

C	8.880737	2.445120	1.105516
C	8.061250	1.405039	0.362131
O	6.646594	1.784372	0.333806
C	6.239904	2.576970	-0.654886
O	6.929205	2.989002	-1.568452
C	4.749495	2.916137	-0.498942
C	4.465785	3.630643	0.856618
N	5.265987	4.850753	0.943260
C	5.704274	5.433912	2.083555
N	5.434962	4.831426	3.251581
S	6.600624	6.893523	2.018395
C	2.968064	3.833334	1.092988
C	2.208098	2.752555	1.563798
C	0.833786	2.887828	1.766793
C	0.204796	4.108749	1.505358
C	0.957701	5.190450	1.042639
C	2.333262	5.054706	0.836157
C	4.326392	3.722179	-1.720265
O	4.395722	4.926273	-1.812504
C	3.754114	2.897630	-2.898222
F	4.560392	1.856913	-3.181973
F	2.550949	2.405652	-2.536524
F	3.603870	3.642187	-3.994195
H	9.918399	2.102673	1.173690
H	8.498347	2.590545	2.120137
H	8.870069	3.404418	0.580411
H	8.062439	0.442291	0.875132
H	8.401496	1.271788	-0.666159
H	4.199764	1.969537	-0.483055
H	4.841520	2.931800	1.609940
H	5.376281	5.398688	0.099421
H	4.816975	4.037194	3.339887
H	5.770573	5.256906	4.102212

H	2.693695	1.803110	1.779081
H	0.257792	2.043970	2.135969
H	-0.863970	4.217139	1.667197
H	0.477118	6.144034	0.842718
H	2.910037	5.903653	0.482788

No Aryl Substituent (Benzaldehyde) Intermediate 6 Conformer 5

C	8.888982	3.111596	1.192815
C	8.184374	1.930433	0.548777
O	6.738278	2.156834	0.494658
C	6.256202	2.815706	-0.556250
O	6.902868	3.217347	-1.504906
C	4.737804	3.012634	-0.420581
C	4.386240	3.844075	0.850001
N	5.057692	5.141492	0.785919
C	5.406826	5.905420	1.847902
N	5.170726	5.426764	3.079021
S	6.153381	7.429399	1.609264
C	2.876449	3.919141	1.084770
C	2.236059	2.837154	1.706188
C	0.855830	2.854922	1.913933
C	0.101286	3.959088	1.506615
C	0.734720	5.042729	0.893490
C	2.115974	5.024083	0.682091
C	4.230618	3.625597	-1.719442
O	4.171380	4.811968	-1.948125
C	3.754548	2.616031	-2.791409
F	4.677365	1.651025	-2.970470
F	2.617811	2.030712	-2.361175
F	3.517749	3.212428	-3.959949
H	9.956764	2.886837	1.282162
H	8.492430	3.304045	2.194091
H	8.778355	4.015039	0.586459
H	8.283897	1.022331	1.144993
H	8.540845	1.742622	-0.465448
H	4.290261	2.022573	-0.288923
H	4.834512	3.277410	1.672191
H	5.124877	5.590497	-0.118642
H	4.620138	4.598605	3.256309
H	5.434457	5.988756	3.874017
H	2.819653	1.979718	2.034493
H	0.373092	2.011846	2.400023
H	-0.972274	3.976862	1.671843
H	0.156012	5.906900	0.579566
H	2.599122	5.874280	0.211032

No Aryl Substituent (Benzaldehyde) Intermediate 7 Conformer 0

H	1.063252	0.007742	-0.077673
C	2.151657	-0.114713	-0.017443
N	2.799285	0.800380	-0.965824
C	2.963552	0.554389	-2.275098
S	3.560815	1.730739	-3.352744
N	2.636434	-0.691433	-2.716653
C	2.118131	-1.785797	-1.922541
O	0.738444	-1.986168	-2.112026
C	2.518736	-1.555539	-0.437573
H	3.602389	-1.668725	-0.365873
C	1.924651	-2.606174	0.499706
O	2.578843	-3.525842	0.952551
O	0.635272	-2.379475	0.766342
C	-0.051335	-3.340558	1.622144
C	-0.581207	-4.508358	0.806974
C	2.755263	-3.086863	-2.476124
F	2.584334	-3.151355	-3.813982
F	4.076641	-3.129790	-2.229315
F	2.189029	-4.177679	-1.937474
C	2.594225	0.209510	1.401214
C	1.639230	0.424512	2.401841
C	2.039127	0.706345	3.710939
C	3.397680	0.779843	4.027840
C	4.356406	0.570943	3.031259
C	3.957132	0.286334	1.724426
H	3.007868	1.739329	-0.652984
H	2.703033	-0.839606	-3.716058
H	0.288837	-1.129191	-2.068868
H	-0.861702	-2.764685	2.072351
H	0.638578	-3.665660	2.403571
H	-1.234316	-4.152941	0.004378
H	-1.157993	-5.174277	1.456805
H	0.236269	-5.083489	0.363247
H	0.581352	0.371404	2.159988
H	1.289842	0.872526	4.479677
H	3.709136	1.002293	5.044589
H	5.414379	0.629402	3.270728
H	4.708731	0.131628	0.954686

No Aryl Substituent (Benzaldehyde) Intermediate 7 Conformer 1

H	1.107863	0.146651	-0.200731
C	2.183740	-0.007810	-0.055607
N	2.923305	0.819292	-1.013863
C	3.198123	0.482109	-2.284185

S	3.926525	1.573229	-3.372170
N	2.899730	-0.787763	-2.669655
C	2.169151	-1.744813	-1.873254
O	0.796705	-1.639295	-2.157674
C	2.489805	-1.495781	-0.375813
H	3.548204	-1.694079	-0.199377
C	1.649487	-2.383064	0.533236
O	0.433713	-2.491133	0.417385
O	2.373057	-2.984230	1.462316
C	1.655471	-3.819523	2.429344
C	2.681699	-4.363374	3.400780
C	2.660271	-3.148627	-2.315498
F	2.513364	-3.300773	-3.645805
F	3.957953	-3.346456	-2.016254
F	1.943896	-4.114203	-1.704966
C	2.545361	0.369539	1.369853
C	1.527174	0.590939	2.305290
C	1.842204	0.909056	3.629113
C	3.177636	1.015302	4.023924
C	4.198239	0.801072	3.091810
C	3.884788	0.477171	1.771209
H	3.126198	1.774809	-0.750263
H	3.017690	-0.991264	-3.654045
H	0.305453	-1.999246	-1.389433
H	1.144053	-4.611095	1.875705
H	0.904928	-3.195735	2.921507
H	3.429977	-4.970275	2.882674
H	2.176032	-4.994104	4.138250
H	3.189111	-3.552056	3.930880
H	0.487162	0.518706	1.997257
H	1.044701	1.080844	4.346394
H	3.423618	1.267937	5.051520
H	5.238488	0.886097	3.393152
H	4.682126	0.314947	1.050593

No Aryl Substituent (Benzaldehyde) Intermediate 7 Conformer 2

H	1.043019	-0.068687	0.016333
C	2.138452	-0.113067	0.038383
N	2.679021	0.850557	-0.926670
C	2.848074	0.627472	-2.239566
S	3.346782	1.856937	-3.309784
N	2.633491	-0.638487	-2.688113
C	2.096736	-1.717698	-1.894008
O	0.696932	-1.741316	-2.022031
C	2.559017	-1.533288	-0.423705

H	3.645564	-1.622835	-0.379140
C	1.926003	-2.572454	0.492769
O	0.719541	-2.789154	0.510612
O	2.820852	-3.182985	1.253655
C	2.340725	-4.194686	2.201592
C	1.924548	-3.554934	3.514107
C	2.670849	-3.029083	-2.492399
F	2.393221	-3.106105	-3.808220
F	4.006570	-3.108403	-2.344724
F	2.124091	-4.103947	-1.888965
C	2.613465	0.216067	1.442277
C	1.678596	0.345166	2.476430
C	2.101037	0.624342	3.779174
C	3.461011	0.783018	4.054032
C	4.398652	0.662684	3.022743
C	3.978140	0.379036	1.722955
H	2.821874	1.801196	-0.611516
H	2.664242	-0.765575	-3.691674
H	0.334270	-2.200782	-1.235912
H	3.199429	-4.855657	2.325438
H	1.521464	-4.742149	1.732404
H	2.744776	-2.971424	3.941750
H	1.654252	-4.343958	4.223398
H	1.058386	-2.901875	3.379335
H	0.619031	0.231202	2.262136
H	1.367362	0.723779	4.574027
H	3.790456	1.004196	5.065374
H	5.457228	0.789959	3.230925
H	4.710738	0.291041	0.924875

No Aryl Substituent (Benzaldehyde) Intermediate 7 Conformer 3

H	1.088393	0.023929	0.030352
C	2.180059	-0.081433	0.064442
N	2.792375	0.796236	-0.939266
C	2.978510	0.487049	-2.230774
S	3.573409	1.616008	-3.360285
N	2.679716	-0.785404	-2.615382
C	2.113536	-1.817234	-1.774463
O	0.721943	-1.951204	-1.942616
C	2.529854	-1.541338	-0.301468
H	3.610207	-1.679243	-0.225877
C	1.842409	-2.492753	0.675244
O	0.681896	-2.391719	1.021735
O	2.698900	-3.422287	1.113529
C	2.186446	-4.424266	2.044381

C	1.561107	-5.592288	1.299826
C	2.686150	-3.170308	-2.271159
F	2.511862	-3.287472	-3.603351
F	4.002565	-3.273382	-2.018648
F	2.061662	-4.204585	-1.681410
C	2.660403	0.302273	1.455010
C	1.732327	0.498237	2.484998
C	2.164762	0.832324	3.770963
C	3.528696	0.977650	4.035476
C	4.460015	0.786739	3.009897
C	4.028911	0.449160	1.725909
H	2.987903	1.752885	-0.674808
H	2.736672	-0.973916	-3.608540
H	0.334337	-1.073249	-2.072569
H	1.473430	-3.941151	2.715442
H	3.067378	-4.731765	2.610320
H	0.671759	-5.276914	0.747542
H	1.264072	-6.360173	2.021587
H	2.274675	-6.033859	0.598362
H	0.671280	0.386771	2.280084
H	1.435762	0.983106	4.562129
H	3.865310	1.241213	5.034245
H	5.521894	0.900849	3.208740
H	4.757817	0.308013	0.932102

No Aryl Substituent (Benzaldehyde) Intermediate 7 Conformer 4

H	1.042396	0.213275	-0.059487
C	2.121665	0.057883	0.042354
N	2.825428	0.854965	-0.970953
C	3.163140	0.452693	-2.204494
S	3.914697	1.494031	-3.326387
N	2.905264	-0.845093	-2.526246
C	2.112090	-1.744370	-1.712867
O	0.729902	-1.610723	-1.925624
C	2.404425	-1.438387	-0.223996
H	3.460651	-1.650890	-0.048454
C	1.571074	-2.293162	0.731287
O	0.404202	-2.082621	0.997467
O	2.310851	-3.282036	1.244584
C	1.655510	-4.196784	2.176047
C	1.739753	-3.670650	3.599115
C	2.541813	-3.181458	-2.103708
F	2.414853	-3.338275	-3.443427
F	3.820350	-3.436375	-1.778651
F	1.763227	-4.099758	-1.510661

C	2.553516	0.487008	1.436268
C	1.586405	0.772512	2.407944
C	1.970498	1.146787	3.698306
C	3.325137	1.243956	4.025201
C	4.295507	0.964542	3.057781
C	3.912289	0.586530	1.769961
H	3.002529	1.827762	-0.757041
H	3.097865	-1.109824	-3.484181
H	0.551662	-1.654380	-2.878745
H	2.204030	-5.132722	2.057556
H	0.621643	-4.337434	1.855019
H	2.780984	-3.512458	3.895458
H	1.294574	-4.402374	4.281238
H	1.195015	-2.728101	3.702166
H	0.532763	0.699679	2.153362
H	1.211510	1.367729	4.443507
H	3.624585	1.539146	5.026991
H	5.350559	1.041728	3.305127
H	4.671839	0.377854	1.020884

Stereoisomer 1

S	1.28000	-0.21300	0.58300
C	2.76700	-0.14300	-0.14500
N	3.36900	1.04200	-0.40700
C	4.64500	1.18500	-1.11100
O	4.25800	1.15700	-2.50400
C	5.38900	2.54800	-0.82600
F	4.92200	3.16600	0.28200
F	6.72800	2.40700	-0.71800
F	5.16900	3.41800	-1.85200
C	5.54200	-0.04300	-0.86200
H	6.41800	0.01200	-1.52100
C	6.04300	-0.03300	0.57000
O	6.90600	-1.05500	0.81200
C	7.58000	-1.02100	2.08200
C	6.70800	-1.56200	3.20300
O	5.78000	0.84400	1.38100
N	3.44400	-1.27400	-0.46500
C	4.68700	-1.26700	-1.22100
H	4.34100	-1.16200	-2.25900
C	5.41400	-2.60200	-1.22600
C	5.23000	-3.57600	-0.23400
C	5.89700	-4.79200	-0.32300
O	5.71500	-5.77300	0.61400
C	6.77300	-5.06300	-1.36800

C	6.97600	-4.10500	-2.35300
C	6.30100	-2.88200	-2.28600
H	2.92300	1.86900	-0.02000
H	3.51900	1.78800	-2.57600
H	7.92500	-0.00400	2.30400
H	8.47300	-1.64600	1.98300
H	5.76700	-1.01000	3.28500
H	7.23400	-1.49300	4.16000
H	6.45100	-2.61000	3.02100
H	2.89500	-2.12600	-0.43900
H	4.56900	-3.37800	0.60900
H	5.01700	-5.49000	1.22500
H	7.28100	-6.01700	-1.42000
H	7.65400	-4.30600	-3.18000
H	6.46500	-2.15200	-3.07600

Stereoisomer 2

S	1.62000	0.38000	1.41100
C	2.86400	0.25400	0.32200
N	3.57200	1.34000	-0.05600
C	4.59900	1.35100	-1.09500
O	3.85600	1.64900	-2.29600
C	5.68400	2.48300	-0.90300
F	5.46300	3.18600	0.23600
F	6.95600	2.03600	-0.88300
F	5.60700	3.38800	-1.91600
C	5.22100	-0.03600	-1.29500
H	5.78800	-0.02400	-2.23600
C	6.22500	-0.46600	-0.23600
O	6.19600	0.30000	0.88700
C	7.10200	-0.06200	1.94800
C	8.40900	0.70500	1.83400
O	6.96800	-1.42300	-0.40000
N	3.22300	-0.94300	-0.19900
C	4.10400	-1.09900	-1.35200
H	4.53700	-2.10100	-1.22900
C	3.31500	-1.16400	-2.66900
C	3.98100	-1.41500	-3.88300
C	3.26900	-1.45100	-5.08600
O	3.89300	-1.67600	-6.27900
C	1.89400	-1.25700	-5.11700
C	1.21400	-1.03100	-3.92700
C	1.91600	-0.98800	-2.71900
H	3.28300	2.22100	0.35600
H	3.46800	2.53300	-2.18500

H	7.28300	-1.14200	1.97100
H	6.61100	0.20100	2.89000
H	8.89100	0.52900	0.86700
H	9.10200	0.41000	2.62900
H	8.23400	1.78300	1.91400
H	2.62900	-1.72800	0.04800
H	5.05500	-1.57400	-3.89000
H	4.84100	-1.80200	-6.11800
H	1.35700	-1.28200	-6.06000
H	0.13700	-0.87900	-3.93800
H	1.33700	-0.79400	-1.81900

Stereoisomer 3

S	1.62500	0.17500	1.23100
C	2.90700	0.06700	0.18700
N	3.57400	1.16800	-0.21300
C	4.48900	1.19800	-1.35300
O	3.59000	1.26500	-2.47400
C	5.45200	2.45200	-1.27500
F	5.04900	3.29200	-0.28300
F	6.73600	2.12600	-1.01500
F	5.40900	3.18200	-2.41300
C	5.30000	-0.11200	-1.41600
H	5.98600	-0.13300	-0.55800
C	6.14900	-0.12100	-2.67600
O	6.91200	-1.23800	-2.76700
C	7.86700	-1.27600	-3.84300
C	7.21800	-1.66000	-5.16000
O	6.21300	0.81200	-3.46900
N	3.35500	-1.12500	-0.26500
C	4.32400	-1.29400	-1.34400
H	3.72900	-1.34000	-2.26500
C	5.01100	-2.63900	-1.14100
C	5.74700	-2.91400	0.02700
C	6.44100	-4.11500	0.14500
O	7.19400	-4.39600	1.24700
C	6.40300	-5.06400	-0.86800
C	5.65200	-4.81700	-2.01500
C	4.95500	-3.61500	-2.15000
H	3.16800	2.04800	0.08700
H	3.45700	2.20700	-2.68700
H	8.61900	-2.02600	-3.57600
H	8.38800	-0.31400	-3.92100
H	6.82700	-2.68200	-5.11400
H	7.94500	-1.60300	-5.97500

H	6.37300	-1.00700	-5.40100
H	2.78700	-1.92600	-0.00800
H	5.78000	-2.18200	0.82900
H	7.16300	-3.63700	1.85100
H	6.95900	-5.99100	-0.76600
H	5.62100	-5.55700	-2.81100
H	4.39000	-3.43500	-3.06300

Stereoisomer 4

S	1.21000	-0.29700	0.63500
C	2.68300	-0.15200	-0.11000
N	3.31700	1.04300	-0.20900
C	4.41900	1.27100	-1.13900
O	5.01300	2.50000	-0.70600
C	3.91400	1.46500	-2.63500
F	2.70400	0.89500	-2.84600
F	3.79300	2.77100	-2.96900
F	4.77100	0.90700	-3.52500
C	5.37400	0.06100	-1.06500
H	6.18200	0.16800	-1.80300
C	6.08800	0.08100	0.26800
O	5.77700	-0.92800	1.10000
C	6.42600	-0.92700	2.38400
C	5.60800	-0.14600	3.39800
O	6.85600	1.00500	0.55000
N	3.32300	-1.24000	-0.61700
C	4.55200	-1.19500	-1.38900
H	4.18100	-1.11500	-2.41800
C	5.34300	-2.49600	-1.39300
C	5.16800	-3.49600	-0.42300
C	5.91300	-4.67300	-0.48600
O	5.75400	-5.66400	0.43800
C	6.84300	-4.87500	-1.49700
C	7.03500	-3.89300	-2.46200
C	6.28800	-2.71300	-2.41300
H	2.79300	1.86000	0.09400
H	5.73200	2.32100	-0.06600
H	7.44700	-0.53300	2.30900
H	6.51000	-1.97100	2.70300
H	5.45100	0.88500	3.06800
H	6.11200	-0.13300	4.37000
H	4.62000	-0.59800	3.52100
H	2.77100	-2.09300	-0.63300
H	4.46400	-3.34900	0.39200
H	5.03900	-5.41500	1.04500

H	7.41600	-5.80000	-1.53000
H	7.76100	-4.05100	-3.25600
H	6.45100	-1.96700	-3.19000

5. References

1. O'Boyle, N.-M.; Banck, M.; James, C.-A.; Morley, C.; Vandermeersch, T.; Hutchison, G.-R. Open Babel: An open chemical toolbox. *J Cheminform.* **2011**, *3*. https://doi.org/10.1186/1758-2946-3-33.
2. Neese, F. The ORCA program system. *Wiley Interdiscip. Rev. Comput. Mol. Sci.* **2012**, *2*, 73-78.
3. Hanwell, M.-D.; Curtis, D.-E.; Lonie, D.-C.; Vandermeersch, T.; Zurek, E.; Hutchison, G.-R. Avogadro: an advanced semantic chemical editor, visualization, and analysis platform. *J Cheminform.* **2012**, *4*. doi: 10.1186/1758-2946-4-17.
4. Kemmer, G.; Keller, S. Nonlinear least-squares data fitting in Excel spreadsheets. *Nat. Protoc.* **2010**, *5*, 267–281. doi: 10.1038/nprot.2009.182
5. Willcott, Mark Robert. "MestRe Nova." *J. Am. Chem. Soc*. vol. 131, no. 36, 2009, pp. 13180-13180, doi: 10.1021/ja906709t.
